# Structure-based scaffold hopping reveals strategies to overcome oncogenic KIT and PDGFRA mutation-driven drug-resistance in GIST

**DOI:** 10.1038/s41467-026-76340-7

**Published:** 2026-08-06

**Authors:** T. Schulz, M. Beerbaum, A. Scrima, H. Jantzen, A. Teuber, T. Mühlenberg, L. Ebel, F. Garcia-Fossa, A. George, N. Berner, J. Weisner, M. P. Müller, S. Wilhelm, S. Sievers, S. Bauer, D. Rauh

**Affiliations:** 1 Department of Chemistry and Chemical Biology, TU Dortmund University and Drug Discovery Hub Dortmund (DDHD, Zentrum für Integrierte Wirkstoffforschung (ZIW), Dortmund, Germany; 2 Department of Medical Oncology and Sarcoma Center, West German Cancer Center, DKTK partner site Essen, German Cancer Consortium (DKTK), University Duisburg-Essen, Medical School, Essen, Germany; 3 Max Planck Institute of Molecular Physiology, Compound Management and Screening Center (COMAS), Dortmund, Germany; 4 Chair of Proteomics and Bioanalytics, TUM School of Life Sciences, Technical University of Munich (TUM), Freising, Germany; 5 Present Address: Department of Biological Engineering, Massachusetts Institute of Technology (MIT), Koch Institute for Integrative Cancer Research at MIT, Broad Institute of MIT and Harvard, Cambridge, MA USA

**Keywords:** Kinases, Structure-based drug design, X-ray crystallography

## Abstract

Gastrointestinal stromal tumors (GIST) are the most common mesenchymal tumors of the gastrointestinal tract. Current tyrosine kinase inhibitors (TKIs) targeting oncogenic KIT and PDGFRA have improved patient outcomes, yet off-target toxicities and drug resistance mutations remain major clinical challenges. Many approved TKIs, often repurposed from other cancer indications, harbor diverse hinge-binding motifs that limit activity against resistance mutations clustering in the ATP-binding pocket of the kinase domain. Here, we describe a structure-based scaffold-hopping strategy to design kinase inhibitors with selectivity for mutant KIT/PDGFRA. Using structure-activity relationship (SAR) studies and 14 determined co-crystal structures, including a structure of the PDGFRA-G680R solvent-front mutation, we define key molecular interactions underlying resistance and inhibitor selectivity. Our lead 6,7-quinazoline-based inhibitors show high potency against clinically relevant KIT/PDGFRA mutations and effectively suppress downstream signaling. These compounds provide selective chemical tools to interrogate resistance mechanisms, and the PDGFRA-G680R structure shows the molecular basis for targeting solvent-front mutations across oncogenic kinases.

## Abstract (web summary)

In this work, the authors develop and characterize KIT/PDGFRA inhibitors to define key molecular interactions underlying inhibitor selectivity and drug-resistance, reporting structural insights for targeting solvent-front mutations across oncogenic kinases.

## Introduction

**Fig. 1:**
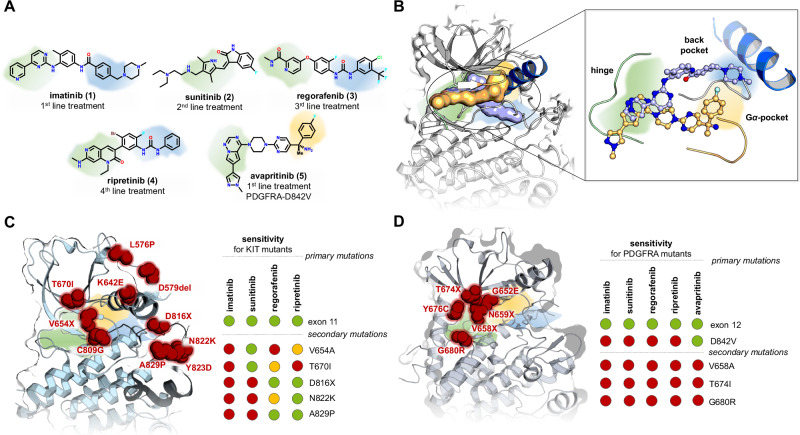
Current treatment landscape and molecular challenges in GIST therapy. **A** Approved TKI for the treatment of GIST. **B** Comparison of the binding modes of the type II inhibitor imatinib (blue) and the type 1.5 inhibitor avapritinib (orange). **C**, **D** Overview of secondary KIT drug resistance mutations and drug sensitivity panel for selected imatinib resistance mutations (**C**) and of secondary PDGFRA drug resistance mutations and drug sensitivity panel for selected avapritinib resistance mutations (**D**). Colors indicate drug sensitivity (green: sensitive; orange: intermediate sensitivity; red: resistant). X denotes any amino acid substitution observed in patients at the respective residue. Throughout panels (**A**–**D**), the hinge-binding region is highlighted in pale green, the back-pocket region in blue, and the Gα-pocket in orange.

Gastrointestinal stromal tumors (GIST) are driven by receptor tyrosine kinases KIT and PDGFRA in ~85 % of cases^1,2^. Although GIST is classified as an orphan disease, the number of patients with active disease is increasing, partly due to improved survival^3,4^. Historically, GIST was difficult to treat, but the introduction of targeted therapies against its oncogenic drivers has revolutionized disease management^5,6^. Currently, five tyrosine kinase inhibitors (TKIs) are approved for the treatment of GIST, many of which were repurposed from other oncological indications (Fig. 1A,B)^7,8^. Imatinib was developed to treat chronic myelogenous leukemia (CML), targeting BCR-ABL. It was subsequently found to also potently inhibit primary KIT and (non-D842V)-PDGFRA mutations and is now the established first-line therapy in GIST (Fig. 1C,D)^7,9^. However, despite the efficacy of these TKIs for GIST, the development of acquired drug resistance across different kinase regions represents a significant clinical challenge (Fig. 1C,D)^10,11^. Efforts to overcome drug resistance led to the repurposing of sunitinib and regorafenib as second and third-line therapies, respectively. Sunitinib is effective against ATP-binding pocket (AP) mutations, while regorafenib is more potent against activation loop (AL) mutations (Fig. 1C). Yet, both show moderate clinical activity with median PFS of 5-6 months and are associated with unfavorable side effect profiles due to their broad multikinase activity^12,13^. Approved in 2020 as a fourth-line treatment for KIT-mutated GIST, ripretinib inhibits a range of primary and secondary mutations, particularly in the AL (Fig. 1C)^14^. Nonetheless, recent studies have already revealed compound escape mechanisms, like AP/AL mutations, limiting therapeutic durability^10^. While different TKIs are approved to counteract resistance mutations in KIT-driven GIST, there are currently no effective therapies for secondary PDGFRA mutations beyond the predominant D842V mutation (Fig. 1D). Localized within the AL, PDGFRA-D842V stabilizes the active kinase conformation, rendering all approved type II inhibitors ineffective (Fig. 1B)^15,16^. The type 1.5 inhibitor avapritinib is currently the only approved therapy for PDGFRA-D842V-driven GIST and has demonstrated remarkable response rates^17^. It also has clinical relevance in advanced systemic mastocytosis due to its high potency against AL-mutant KIT^18^. However, its use is limited by cognitive side effects^19^ and the emergence of resistance mutations^16^. Notably, secondary resistance mutations cluster in the AP region, such as the prominent solvent-front mutation PDGFRA-G680R, which has been clinically observed in patients and demonstrates the highest level of resistance to avapritinib (Fig. 1D)^16,20^. In such cases, no alternative therapies are available, and prognosis remains poor. Solvent-front mutations like PDGFRA-G680R constitute a recurrent resistance mechanism across multiple oncogenic kinases, including ALK-G1202R, ROS1-G2032R, TrkA-G595R and RET-G810R^21^. Despite their clinical significance, there is a lack of structural insights into solvent-front resistance in PDGFRA, hindering rational design efforts.

In summary, many approved GIST therapies were not specifically designed to target KIT/PDGFRA, possess non-tailored hinge-binding elements, and are limited by side effects and drug resistance. Accordingly, there is a clear need for inhibitors tailored to mutant KIT/PDGFRA that can elucidate current resistance mechanisms and thus guide the design of next-generation inhibitors to overcome drug resistance. Recently, we reported the crystal structure of avapritinib in complex with KIT and PDGFRA, providing structural insights into its binding mode and the impact of resistance mutations^15^. The crystal structures in that work revealed a previously undescribed pocket (G*α*-pocket), located in the N-lobe of the kinase domain, bordered by regulatory elements including the Gly-rich loop and the *α*C-helix, and demonstrated its importance for potent ligand engagement (Fig. 1).

In this work, we report the design, synthesis, biological, and structural characterization of inhibitors that exploit G*α*-pocket binding in combination with a structure-based scaffold-hopping approach targeting drug-resistant KIT/PDGFRA.

## Results

### Avapritinib resistance motivates structure-based scaffold hopping

**Fig. 2:**
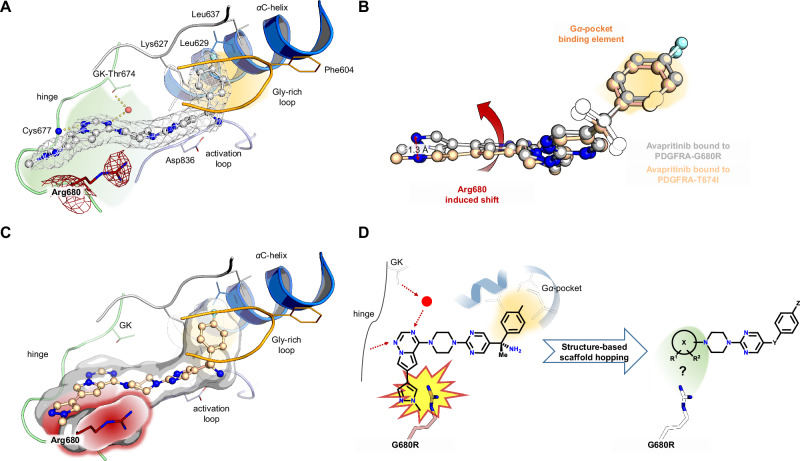
Structural insights into the solvent-front mutation PDGFRA-G680R motivates the structure-based scaffold hopping of avapritinib. **A** Complex crystal structure of avapritinib bound to PDGFRA-G680R (PDB-ID: 9TF9). Avapritinib and Arg680 are shown with their |2Fo-Fc|-electron density map at σ-level = 1.0. **B** Comparison of the ligand orientation of avapritinib in PDGFRA-T674I (orange, PDB-ID: 8PQH) and PDGFRA-G680R (gray, PDB-ID: 9TF9). The methylpyrazole group of avapritinib in PDGFRA-G680R is displaced relative to its binding in PDGFRA-T674I, indicating steric interference with Arg680. **C** Alignment of co-crystal structures of avapritinib bound to PDGFRA-T674I (orange ligand, protein not shown, PDB-ID: 8PQH) and to PDGFRA-G680R (protein shown, ligand omitted, PDB-ID: 9TF9). The overlay underlines the conformational incompatibility caused by the proximity of the methylpyrazole and Arg680. **D** Schematic overview of key protein-ligand interactions (left) and opportunities for optimization to address ATP-binding pocket mutations (right), such as PDGFRA-G680R, forming the basis for a structure-based scaffold hopping approach. X = H or diverse mono- or bicyclic N-heteroaromatic ring systems, R^1^ = H or methyl, nitrile, ether, ester, hydroxy, halogen, nitro, amine, methyl-1H-pyrazole, R^2^ = H or nitro, amine, ether, Y = CH_2_ or (S)-ethan-1-amine, Z = H or F. GK gatekeeper.

The most challenging resistance mutation for avapritinib is the solvent-front mutation PDGFRA-G680R, which is positioned at the entrance of the ATP-binding pocket (Fig. 1D). To elucidate the molecular basis of resistance, we sought to determine the crystal structure of this mutant in complex with avapritinib. Extensive efforts in protein expression, purification, and crystallization ultimately yielded the co-crystal structure of the PDGFRA-G680R resistance mutation, revealing well-defined electron density for avapritinib. However, while the electron density for the guanidinium group of Arg680 is well defined, the density for Cγ and Cδ of the side chain of Arg680 is less well resolved, indicating conformational flexibility. Despite this, the structure provides key insights into the resistance mechanism (Fig. 2A). The overall binding mode of avapritinib remains largely consistent with the wild-type, particularly in regions distal to the hinge, such as the G*α*-pocket. However, near the mutation site, the methylpyrazole and triazine scaffold exhibit a pronounced shift, indicating an induced reorientation of the inhibitor. The methylpyrazole moiety of avapritinib is shifted by 1.3 Å and tilted by 20° compared to avapritinib bound to the PDGFRA gatekeeper mutant T674I (Fig. 2B). This suggests steric interference between Arg680 and the inhibitor, which in turn prevents avapritinib from adopting its preferred binding conformation (Fig. 2C). Given that other key protein-ligand interactions are preserved, our findings strongly support the need for rational scaffold modifications to circumvent steric hindrance imposed by this bulky, basic amino acid. Given that most avapritinib resistance mutations cluster near the ATP-binding pocket and that analogous AP mutations in KIT pose a significant challenge to many approved drugs, we employed a structure-based scaffold hopping approach to design inhibitors capable of effectively targeting mutant KIT and PDGFRA. The co-crystal structure of avapritinib bound to PDGFRA-G680R showed a direct hydrogen bond between the N2 nitrogen of the pyrrolo[2,1-f][1,2,4] triazine scaffold and the backbone of Cys677 (Cys673 in KIT) in the hinge region (Fig. 2A). Additionally, a water-mediated contact with the gatekeeper residue Thr674 was observed via the N4 nitrogen (Fig. 2A). Thus, the hypothesis was that these two polar interactions are important for effective binding to the hinge region of KIT and PDGFRA. Accordingly, particular emphasis was paid to heterocyclic scaffolds mimicking the 2,4-nitrogen motif of the triazine, like, for example, pyrimidines or quinazolines (Fig. 2D). In the case of the solvent-front mutation G680R, the methylpyrazole moiety seems unable to accommodate the increased steric demand imposed by the arginine substitution. Hence, smaller scaffolds were thought to be beneficial to address this mutant. Accordingly, inhibitors that do not contain a hinge-binding element also appear to be of interest. Further, the phenyl moiety binding to the G*α*-pocket was maintained since we demonstrated the contribution of this motif to potent ligand binding^15^. The piperazinyl-pyrimidine moiety was also retained to link the G*α*-pocket binding element to various known hinge binding motifs. To improve synthetic accessibility and to systematically assess the contribution of the stereogenic center to potency and selectivity, this feature was temporarily omitted during early SAR exploration. The resulting 5-benzyl-2-(piperazin-1-yl)pyrimidine scaffold served as a simplified core for subsequent decoration, thereby enabling rapid analogue synthesis while decoupling stereochemical effects from core hinge-binding interactions (Fig. 2D).

### Synthesis of inhibitors for mutant KIT/PDGFRA

**Fig. 3:**
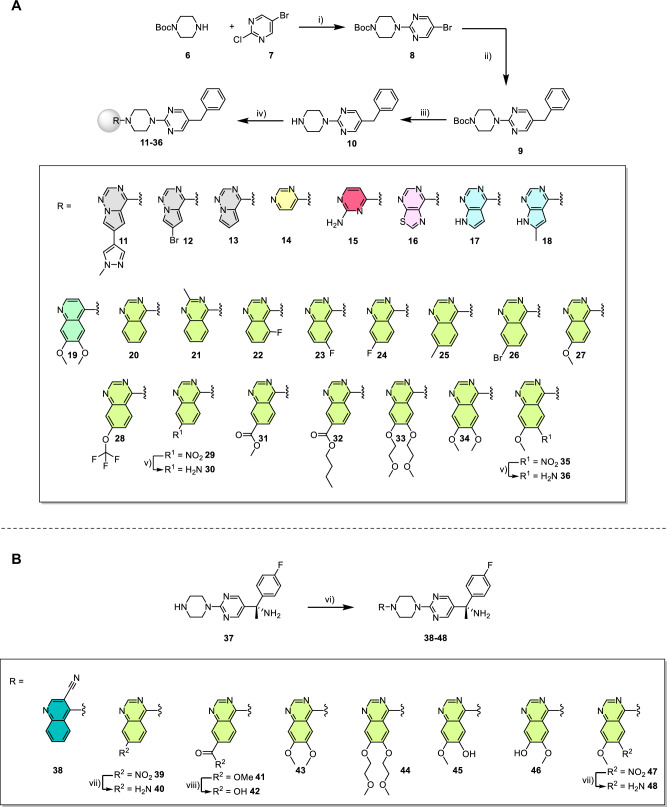
Synthesis and overview of type 1.5 KIT/PDGFRA inhibitors. **A** Linear four-step synthesis of 5-benzyl-2-(piperazin-1-yl)pyrimidine-based inhibitors. **B** Synthesis of (S)-1-(4-fluorophenyl)-1-(2-(piperazin-1-yl)pyrimidin-5-yl)ethan-1-amine-based KIT/PDGFRA inhibitors. Reaction and conditions: (i) TEA, dry THF, rt, 16 h (90 %); (ii) 2-benzyl-4,4,5,5-tetramethyl-1,3,2-dioxaborolane, Pd(PPh_3_)_4_, Cs_2_CO_3_, dioxane/water (5:1), 80 °C, 72 h in a sealed pressure flask (53 %); (iii) TFA/DCM (1:5), rt, ovn. (90 %); (iv) different chloro-pyrimidines, K_2_CO_3_, dry THF/dry dioxane (2:1), rt, 2-4 d (22 %-quant.); (v) Iron, NH_4_Cl, 80 °C, 2 d (66 %). (vi) Chloro-pyrimidines, K_2_CO_3_, dry THF/dry dioxane (2:1), rt, 2-4 d (31-76 %); (vii) Iron, NH_4_Cl, 80 °C, 2 d (25-70 %).; (viii) NaOH, rt, 1 d (11 %).

**Fig. 4:**
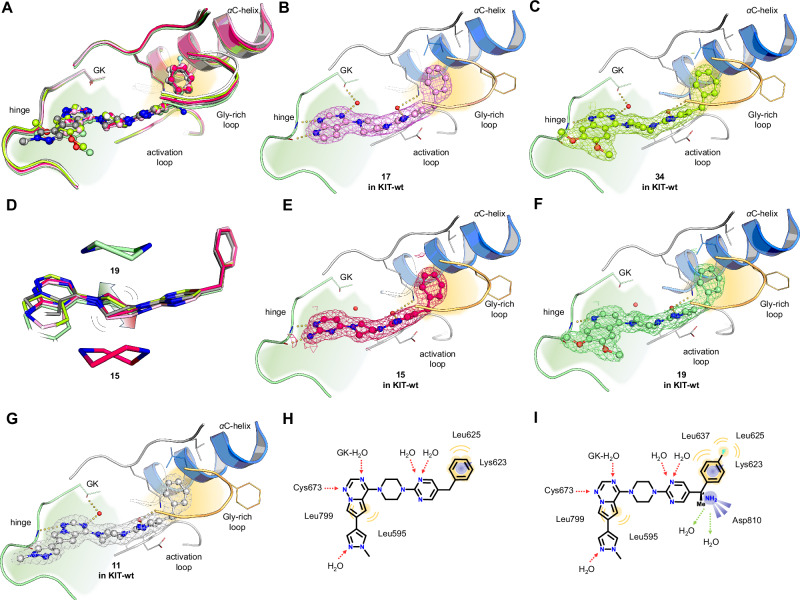
Structural insights into different hinge-binding elements reveal protein-ligand interactions and ligand conformations of avapritinib-based inhibitors. **A** Comparison of complex crystal structures of avapritinib (PDB-ID: 8PQ9, white), **11** (PDB-ID: 9TFD, grey), **15** (PDB-ID: 9TFE, red), **17** (PDB-ID: 9TFF, pink), **19** (PDB-ID: 9TFG, pale green) and **34** (PDB-ID: 9TFJ, lime green) bound to KIT-wt. **B** Complex crystal structure of **17** bound to KIT-wt (PDB-ID: 9TFF). **C** Complex crystal structure of **34** bound to KIT-wt (PDB-ID: 9TFJ). **D** Close-up of ligand conformations in co-crystal structures, illustrating how the hinge-binding element affects piperazine orientation. **E** Complex crystal structure of **15** bound to KIT-wt (PDB-ID: 9TFE). **F** Complex crystal structure of **19** bound to KIT-wt (PDB-ID: 9TFG). **G** Complex crystal structure of **11** bound to KIT-wt (PDB-ID: 9TFD). **H** Visualization of the pharmacophore model of **11** in KIT-wt (PDB-ID 9TFD). **I** Visualization of the pharmacophore model of avapritinib in KIT-wt (PDB-ID 8PQ9). Selected residues involved in direct polar (dotted yellow lines), hydrophobic, and water-mediated key interactions between KIT-wt and the compounds are shown. Pharmacophore models were generated with LigandScout and illustrated in Microsoft PowerPoint. The |2F_o_-F_c_|-electron density maps are shown at σ-level = 1.0. GK gatekeeper, wt wild-type.

To generate a library of analogs with different scaffolds, late-stage modifications were prioritized to efficiently introduce structural variations. A linear four-step synthesis route was established (Fig. 3A). The first step was the nucleophilic substitution of the commercially available 5-bromo-2-chloropyrimidine with *tert*-butyl piperazine-1-carboxylate, affording *tert*-butyl 4-(5-bromopyrimidin-2-yl)piperazine-1-carboxylate **8** in excellent yield of 90 %. Next, **8** was coupled to 2-benzyl-4,4,5,5-tetramethyl-1,3,2-dioxaborolane in a Suzuki-Miyaura reaction. Subsequently, an acid-mediated carbamate cleavage yielded the key compound **10** in a very good yield of 90 %. Notably, this compound follows a type III inhibitor approach, featuring only a proton in the hinge-binding region. Lastly, nucleophilic substitution reactions of **10** with various halogenated heterocycles, like pyrimidines or quinazolines, afforded the inhibitors **11**-**36**. For the synthesis of derivatives including the fluoro group and stereogenic motif, (S)-1-(4-fluorophenyl)-1-(2-(piperazin-1-yl)pyrimidin-5-yl)ethan-1-amine was directly coupled with selected substituted heterocycles (Fig. 3B). In total, this synthetic strategy enabled the efficient preparation of 39 inhibitors, encompassing various scaffolds. The successfully synthesized inhibitors were evaluated for their inhibitory potency against both wild-type and mutant KIT and PDGFRA. Biochemical assessments were conducted using a homogeneous time-resolved fluorescence (HTRF) assay to determine structure-activity relationships (SAR). Specifically, the half maximal inhibitory concentration (IC_50_) was measured against PDGFRA-wt, PDGFRA-D842V, PDGFRA-G680R, KIT-wt, and KIT-D816H (Table 1). We note that ATP concentrations were adjusted to the respective K_M_(ATP) values for each kinase, minimizing bias in IC_50_ comparisons. Accordingly, comparisons across different kinase variants were interpreted qualitatively, while ligand-to-ligand comparisons within a given kinase are therefore directly comparable. In addition, we aimed to complement and validate the findings with protein X-ray crystallography. In total, 10 structures were determined with five different scaffolds elucidating their binding mode in KIT (Fig. 4, Supplementary Fig. 1). Notably, this effort also yielded co-crystal structures of KIT in complex with inhibitors incorporating aminopyrimidine or pyrrolopyrimidine hinge-binding motifs, expanding the structural understanding of KIT-inhibitor interactions.

**Table 1:** Determined IC_50_ values for inhibitors 10-48 and reference compounds against KIT-wt (200 µM ATP), -D816H (12 µM ATP) and PDGFRA-wt (57 µM ATP), -D842V (14 µM ATP), and -G680R (11 µM ATP)

Cpd	HTRF IC_50_ [nM]				
	KIT- wt	KIT- D816H	PDGFRA- wt	PDGFRA-D842V	PDGFRA-G680R
**imatinib**	51 ± 2	850 ± 50	43 ± 8	2100 ± 400	110 ± 40
**sunitinib**	8.0 ± 2	330 ± 20	6.3 ± 4	1600 ± 600	890 ± 400
**regorafenib**	9.4 ± 2	210 ± 60	4.7 ± 0.4	220 ± 10	320 ± 40
**ripretinib**	2.2 ± 1	2.0 ± 0.3	4.2 ± 0.7	2.6 ± 2	250 ± 90
**avapritinib**	11 ± 2	0.4 ± 0.1	0.4 ± 0.2	≤0.1	95 ± 30
**10**	≥20,000	≥20,000	≥20,000	≥20,000	18,000 ± 2000
**11**	2200 ± 1000	9 ± 3	18 ± 7	1.4 ± 1	15,000 ± 7000
**12**	17,000 ± 3000	2200 ± 700	17,000 ± 4000	8000 ± 3000	≥20,000
**13**	13,000 ± 4000	200 ± 40	1100 ± 500	220 ± 100	6500 ± 3000
**14**	≥20000	690 ± 80	14,000 ± 6000	2100 ± 500	15,000 ± 1000
**15**	18,000 ± 900	3900 ± 2000	9600 ± 2000	17,000 ± 3000	19,000 ± 700
**16**	≥20,000	1380 ± 100	14,000 ± 8000	1700 ± 30	≥20,000
**17**	≥20,000	21 ± 3	1600 ± 1000	24 ± 5	≥20,000
**18**	≥20,000	22 ± 2	500 ± 500	16 ± 2	≥20,000
**19**	4900 ± 2000	68 ± 24	160 ± 90	96 ± 17	6100 ± 2000
**20**	15,000 ± 300	260 ± 29	2400 ± 2000	190 ± 2	8500 ± 500
**21**	≥20,000	≥20,000	16,000 ± 5000	≥20,000	≥20,000
**22**	17,000 ± 4000	860 ± 100	5100 ± 3000	650 ± 90	18,000 ± 1300
**23**	18,000 ± 3000	620 ± 90	4400 ± 3000	490 ± 80	19,000 ± 2000
**24**	14,000 ± 6000	280 ± 50	760 ± 400	270 ± 80	13,000 ± 2000
**25**	1700 ± 1000	19 ± 3	100 ± 50	15 ± 4	5700 ± 500
**26**	1600 ± 500	60 ± 13	180 ± 70	88 ± 20	≥2000
**27**	12,000 ± 6000	14 ± 1	1100 ± 600	19 ± 1	1100 ± 40
**28**	18,000 ± 2000	470 ± 70	9600 ± 7000	540 ± 100	≥20,000
**29**	≥2000	210 ± 20*	1500 ± 600	500 ± 100	≥2000
**30**	3300 ± 2000	53 ± 5	170 ± 80	36 ± 9	2000 ± 400
**31**	2400 ± 200*	63 ± 8	970 ± 600	110 ± 20	≥20,000
**32**	≥2000	1300 ± 500	≥2000	1500 ± 300	≥2000
**33**	92 ± 56	2.6 ± 0.4	4.3 ± 1.5	0.5 ± 0.2	1200 ± 100
**34**	140 ± 60	4.1 ± 0.5	5.4 ± 0.2	3.3 ± 0.3	150 ± 50
**35**	≥20,000	160 ± 10	300 ± 100	77 ± 30	≥20,000
**36**	110 ± 10	6.5 ± 1.0	4.4 ± 2.5	1.0 ± 0.3	460 ± 30
**37**	≥20,000	8300 ± 500	5900 ± 500	4900 ± 4000	≥20,000
**38**	9400 ± 4000	110 ± 5	190 ± 80	62 ± 23	4500 ± 2000
**39**	5700 ± 3000	34 ± 2	220 ± 100	42 ± 20	18,000 ± 2000
**40**	2800 ± 500	29 ± 5	43 ± 30	6.6 ± 1	10,000 ± 2000
**41**	2400 ± 1000	16 ± 2	27 ± 9	13 ± 3	14,000 ± 3000
**42**	≥20,000	4700 ± 400	7600 ± 2000	2700 ± 2000	≥20,000
**43**	100 ± 50	1.1 ± 0.2	2.3 ± 1	0.3 ± 0.2	74 ± 6
**44**	37 ± 20	0.7 ± 0.1	0.8 ± 0.3	≤0.1	290 ± 70
**45**	62 ± 30	0.8 ± 0.1	0.8 ± 0.2	≤0.1	37 ± 20
**46**	6400 ± 900	83 ± 9	180 ± 80	36 ± 20	8800 ± 1000
**47**	740 ± 200	23 ± 4	32 ± 5	16 ± 8	2500 ± 200
**48**	23 ± 3	2.0 ± 1	1.0 ± 1	≤0.1	280 ± 200

Data presented as mean values ± s.d; if not otherwise stated, *n* ≥ 3, where *n* represents the number of independent experiments. *n = 2. Certain compounds display a different concentration range depending on the stock concentration (20,000 nM for 10 mM stock vs. 2000 nM for 1 mM stocks). Source data are provided as a Source Data file.

### Structure-based scaffold hopping reveals determinants of potency and resistance in KIT/PDGFRA inhibition

All inhibitors adopt a binding mode very similar to avapritinib, regardless of their hinge-binding element (Fig. 4A). Accordingly, key protein-ligand interactions, such as binding to the G*α*-pocket or engagement with the catalytic lysine, are preserved. However, distinct differences emerge in the interaction with the hinge region. Some scaffolds facilitate a single hydrogen bond with the hinge region, whereas others, like the pyrrolopyrimidine, enable two hydrogen-bond interactions (Fig. 4B). Conversely, quinazoline scaffolds promote more pronounced hydrophobic interactions at the kinase entrance (Fig. 4C, Supplementary Fig. 2). Additionally, variations in the piperazine conformation are observed depending on the scaffold. Specifically, fused 6- and 5-membered ring systems tend to adopt a boat conformation, while 6- and 6-membered systems favor a chair conformation (Fig. 4D). This conformational flexibility of the piperazine moiety is particularly important, as it serves as the key linker that stabilizes the bioactive conformation of avapritinib by enabling both hinge engagement and optimal positioning within the G*α*-pocket, as highlighted in prior SAR studies^15^. Beyond providing structural insights into the binding mode of various scaffolds to KIT, these structures also serve as a valuable foundation for the SAR evaluation.

Compound **10**, designed as a type III inhibitor, exhibited no apparent inhibition across all tested kinase variants (IC_50_ ≥ 10,000 nM). This finding underscores the necessity of a suitable hinge-binding element for potent KIT/PDGFRA inhibition and indicates that occupying the G*α*-pocket alone is insufficient for strong activity. This first observation is consistent with findings from a recent study employing a different class of inhibitors^22^. To assess the impact of the stereogenic center in greater depth, we investigated **11** alongside avapritinib. Consistent with our previous report^15^, the re-synthesized **11** displayed reduced potency, underscoring the importance of the stereogenic center for potent inhibition (Fig. 4G-I). This consideration is essential when analyzing simplified derivatives, as reintroduction of the stereocenter is likely to increase potency. Stepwise truncation of the methylpyrazole moiety in **11** to a bromine (**12**) and subsequently to hydrogen (**13**) led to a general loss of potency against both wild-type and AL mutant KIT/PDGFRA. While the IC_50_ against the G680R mutant showed some improvement, it remained above 6000 nM, indicating that simple size reduction alone is not sufficient to effectively overcome the solvent-front mutation. A comparison of the different scaffolds **14**-**20** revealed significant differences in potency, despite all having the pyrimidine motif, which retains key polar interactions with the hinge region. Pyrimidine (**14**) and aminopyrimidine (**15**), representing the smallest scaffolds in this series, exhibited IC_50_ values consistently above 1000 nM, with no notable inhibition of PDGFRA-G680R. However, the drop in potency between D842V and G680R is less pronounced. The crystal structure of **15** revealed that its amino group projects toward the solvent-exposed region, thereby preventing the pyrimidine nitrogen from engaging in the water-mediated interaction with the gatekeeper Thr670 observed previously (Fig. 4E). This contrasts with the ADP-bound structure (PDB-ID: 1PKG), in which the adenine amino group forms a direct hydrogen bond with the backbone carbonyl of Glu671 (Supplementary Fig. 3). Notably, in the binding mode observed for **15**, if the heterocycle adopted an approximately 180° flipped orientation, the amino group would be expected to sterically clash with the backbone of Glu671. The thiazolo (**16**) and pyrrolo (**17**) scaffolds share a similar overall shape, yet **17** demonstrated significantly higher potency against AL mutants, with IC_50_ values around 20 nM. Notably, compound **17** exhibited selectivity over the respective wild-type kinases (Table 1). To address this, a methyl group was introduced at the pyrrolo-pyrimidine scaffold (**18**) to reduce the protonation of the basic hydrogen bond donor, potentially enhancing cellular permeability. However, while **18** retained similar biochemical potency, it did not yield improved cellular activity (Supplementary Table 2). Furthermore, both **17** and **18** displayed IC_50_ values exceeding 20,000 nM against PDGFRA-G680R, rendering this scaffold less promising against this type of resistance mutation. In contrast, quinazoline **20** showed an IC_50_ of 190 nM on PDGFRA-D842V and about 8000 nM on the G680R. Thus, this represents a slight improvement over the truncated avapritinib analog **13** in targeting PDGFRA-D842V, as well as the highest potency on the G680R out of the non-decorated scaffold-hopping derivatives. Similarly, the disubstituted quinoline derivative (**19**) showed promising inhibition of AL mutants, with IC_50_ values below 100 nM. However, the respective crystal structure revealed that the quinoline scaffold alone cannot engage in the water-mediated interaction with the gatekeeper, likely reducing its overall potency (Fig. 4F). This suggests that modifications at the ether-substituted positions could provide an opportunity to further modulate potency. Based on these findings, the quinazoline core was selected for further SAR investigations.

### Quinazoline SAR reveals electronic and spatial preferences for targeting KIT/PDGFRA mutants

First, a series of substitutions were introduced at various positions around the quinazoline scaffold to evaluate potential exit vectors for enhancing potency against mutant KIT/PDGFRA. Modification at the 2′ position (**21**) was not tolerated, yielding IC_50_ values ≥ 20,000 nM against AL mutants, likely due to steric interference near the gatekeeper with the backbone of Glu671 in KIT (Glu675 in PDGFRA). This is consistent with the structural observation of the aminopyrimidine, which flipped relative to the adenine-group of ADP due to interference (Supplementary Fig. 3). Fluorine substitutions also proved unfavorable, as all positions tested (**22**-**24**) showed reduced potency. However, among them, the 7′-fluoro derivative **24** exhibited comparatively better potency, suggesting it as the most permissive site for further modification. A subseries of eight 7′-substituted inhibitors (**25**-**32**) was analyzed to investigate the effect on the inhibition of KIT and PDGFRA variants. This revealed clear SAR trends, particularly for PDGFRA-D842V. The most potent inhibitors featured electron-rich groups with moderate steric bulk, with methyl (**25**) and methoxy (**27**) yielding IC_50_ values of 15 nM and 19 nM, respectively. The amino derivative (**30**) retained a potency of around 36 nM. In contrast, analogs bearing strongly electron-withdrawing substituents such as nitro (**29**) and trifluoromethyl (**28**) exhibited a marked reduction in potency (IC_50_ > 500 nM), corresponding to a 30-fold drop in potency. These results suggest that electronic enrichment at the 7′-position, when paired with a distinct size, enhances binding affinity; potentially by favoring the bioactive ligand conformation or strengthening interactions near the hinge region within the D842V mutant active site. Supporting this, a co-crystal structure of **25** bound to KIT revealed enhanced hydrophobic contacts at the kinase entrance, particularly with Tyr672 and Leu595 (Supplementary Fig. 2). A similar SAR trend was observed for the corresponding AL mutation KIT-D816H. Again, methyl (**25**) and methoxy (**27**) emerged as the most potent compounds (IC_50_ = 19 nM and 14 nM, respectively), while trifluoromethoxy **28** was significantly less active (IC_50_ = 470 nM). These parallels point to a shared sensitivity of both mutant kinases to subtle electronic effects within the pharmacophore. Thus, these results indicate a preferential interaction of electron-dense substituents with the mutant binding pocket profile and can be used for further optimization. Notably, the solvent-front mutation PDGFRA-G680R also showed increased sensitivity to electron-rich analogs (e.g., methoxy **27**, IC_50_ = 1.1 µM), whereas electron-deficient compounds such as nitro **29** were inactive (IC_50_ > 20 µM). Consistent with the observed SAR trends, the ester analogs **31** and **32** exhibited reduced potency against AL mutant KIT/PDGFRA compared to **25** or **27** (Table 1). Interestingly, wild-type KIT and PDGFRA displayed lower sensitivity overall, indicating a favorable selectivity profile. In particular, the methoxy group conferred high selectivity with up to 56-fold and 850-fold preference for PDGFRA-D842V and KIT-D816H over their wild-type counterparts, respectively. Building on the promising results of electron-rich 7′-substituents, we next explored dual substitution at positions 6 and 7 of the quinazoline core to further enhance potency. The introduction of ether chains at the 6,7-positions emerged as a particularly effective strategy, especially against the PDGFRA-D842V mutation.

A clear potency trend was observed: bis(2-methoxyethoxy) (**33**) > dimethyl ether (**34**) > monoether (**27**), with IC_50_ values of 0.5 nM, 3.3 nM, and 19 nM, respectively. The co-complex structure of KIT-wt and **33** reveals an additional hydrogen bond between the backbone nitrogen atom of Asp677 and the distal oxygen of one of the two methoxyethoxy groups, which aligns with the observed increase in potency. In the case of the solvent-front mutation PDGFRA-G680R, the dimethyl ether analog (**32**) showed the greatest improvement over the monoether (**27**), achieving a sevenfold increase in potency (IC_50_ ≈ 150 nM vs. 1100 nM). However, the extended bis(2-methoxyethoxy) group in compound **33**, while highly potent against D842V, failed to translate this advantage to the G680R mutant (IC_50_ ≈ 1200 nM). This suggests that the elongated chain may introduce steric constraints or misalignment within the altered binding pocket, particularly in proximity to Arg680, as described further below. Further supporting these observations, **36** (7-methoxyquinazolin-6-amine) displayed comparable potency to compound **34** on both wild-type kinases and AL mutants. However, it exhibited a threefold reduction in potency against PDGFRA-G680R. This suggests that substitution patterns around the 6- and 7-positions may differentially impact engagement with the solvent-front residue and highlight the nuanced balance between interaction potential and steric accommodation in this region.

### Lead optimization yields sub-nanomolar, mutant-selective KIT/PDGFRA inhibitors

To efficiently derive the initial SAR, we first employed a simpler parent compound, already described earlier in the manuscript. We then applied the more complex (S)-1-(4-fluorophenyl)-1-(2-(piperazin-1-yl)pyrimidin-5-yl)ethan-1-amine to investigate further and optimize the most promising leads and SAR trends. One approach, inspired by the water-mediated interaction observed with the quinazoline scaffold, involved retaining the core structure while replacing the bridging water molecule with a nitrile group (**38**). This scaffold did not show significant improvement in inhibition across the investigated kinase variants, with IC_50_ values of ~60 and 100 nM against PDGFRA-D842V and KIT-D816H, respectively, and 4500 nM against PDGFRA-G680R. However, it should be noted that it is a non-decorated scaffold. The co-complex structure of **38** revealed a well-aligned binding pose, suggesting that neither conformational rigidity nor nitrile positioning hindered the protein-ligand interaction (Supplementary Fig. 1). These findings imply that the bridging water molecule is an integral component of the interaction network and its displacement seems energetically unfavorable compared to maintaining the polar interaction. The other key trends described earlier remained consistent in this advanced series. For instance: (i) hinge binding proved essential, as demonstrated by the marked loss of potency in compound **37**, and (ii) substituents with higher electron density and favorable steric properties were favored, with a potency ranking of ether **43** > amine **40** > ester **41** > nitro **39**. Furthermore, indeed, reinstallation of the stereogenic center and the fluoro group led to an overall increase in potency, as demonstrated across matched compound sets (e.g., **33** vs. **44,** **34** vs. **43**, and **36** vs. **48**). Interestingly, this enhancement was more pronounced for PDGFRA variants than for KIT in the case of ether derivatives, while the effect was comparable for the amine analogs and slightly reversed for nitro-containing inhibitors. The most promising inhibitors, like **43,** **44,** **45** and **48**, demonstrated higher biochemical potency (≤2 nM) against KIT-D816H compared to imatinib, sunitinib and regorafenib (>200 nM) as well as comparable potency compared to the AL inhibitors ripretinib and avapritinib (≤2 nM). Importantly, they showed significantly reduced sensitivity against KIT-wt, with up to 90-fold selectivity favoring the mutant kinase, representing a high degree of KIT-wt sparing. For PDGFRA-D842V, the data recapitulate the clinically observed resistance to approved type II inhibitors (>200 nM), whereas, e.g., compounds **45** and **48** exhibited sub-nanomolar inhibition (IC_50_ ≤ 0.1 nM), matching the potency of avapritinib. Notably, compound **45** also demonstrated improved inhibition of the solvent-front PDGFRA-G680R mutation compared to avapritinib, with an IC_50_ of 37 nM. This represents a step forward in the development of next-generation TKIs with the capacity to overcome this challenging type of resistance mutation.

### Structural basis of solvent-front mutation resistance: insights from the PDGFRA-G680R mutation

**Fig. 5:**
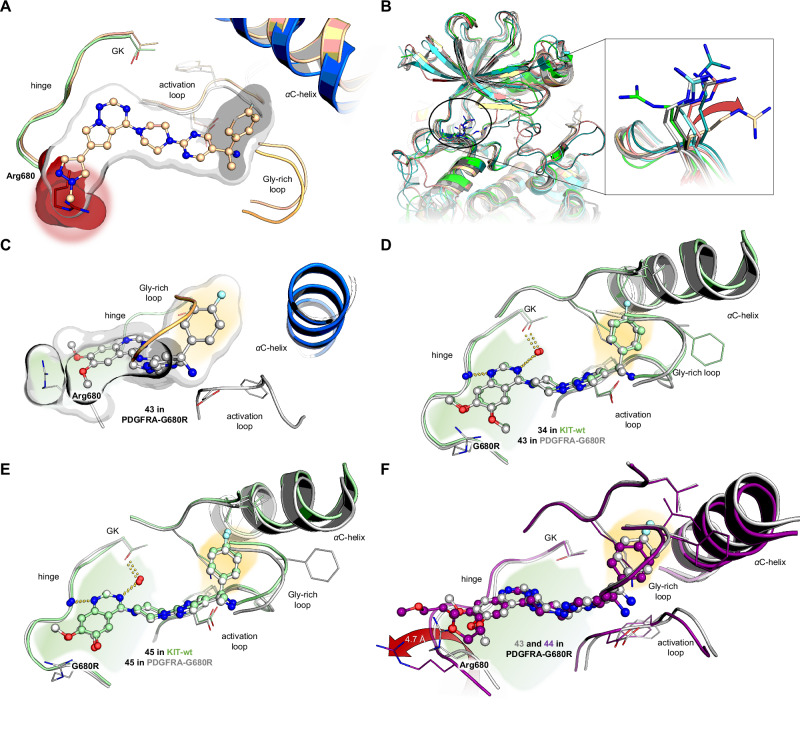
Structural analysis of the avapritinib-resistant PDGFRA-G680R solvent-front mutation, and quinazoline-based inhibitors. **A** Comparison of co-crystal structures of avapritinib bound to KIT-wt (PDB-ID: 8PQ9, wheat) and **43** bound to PDGFRA-G680R (PDB-ID: 9TFA). The binding mode of avapritinib, as observed in the wild-type structure, clashes with the commonly observed conformation of Arg680 in the PDGFRA-G680R mutant, demonstrating significant steric interference at the solvent-front. **B** Comparison of crystal structures avapritinib (wheat), **43** (grey) and **45** (white) bound to PDGFRA-G680R (PDB-ID: 9TF9, 9TFA, 9TFM) and ALK-G1202R (green, PDB-ID: 9GBE), ROS1-G2032R (cyan, PDB-ID: 9QEK) and TrkA-G595R (pink, PDB-ID: 7VKM; deepteal, PDB-ID: 8J5X (ligand bound)). The structures show a similar orientation of the respective arginine residue, highlighting conserved conformational features at the solvent-front across different kinases; except for the co-complex structure of avapritinib, suggesting an induced and favorable conformation of Arg680. **C** Complex crystal structure of **43** bound to PDGFRA-G680R (PDB-ID: 9TFA). The structure shows a well-defined shape complementarity between the di-methoxy group of **43** and Arg680. **D** Comparison of co-crystal structures of **34** bound to KIT-wt (PDB-ID: 9TFJ, pale green) and **43** bound to PDGFRA-G680R (PDB-ID: 9TFA, grey). The structures reveal similar binding modes between the wild-type and the G680R structure. **E** Comparison of co-crystal structures of **45** bound to KIT-wt (PDB-ID: 9TFM, pale green) and bound to PDGFRA-G680R (PDB-ID: 9TFC, grey). The observed binding mode is similar for the wild-type and G680R structures, suggesting that the G680R mutation does not significantly alter ligand orientation. **F** Comparison of co-crystal structures of **43** (PDB-ID: 9TFA, grey) and **44** (PDB-ID: 9TFB, purple) bound to PDGFRA-G680R. The extended ether chain of **44** induces a displacement of approximately 4.7 Å in the amidine head group of Arg680 relative to its position in the **43**-bound structure. GK gatekeeper, wt wild-type.

To deepen our understanding of solvent-front resistance and follow up on this promising result, we performed comprehensive X-ray crystallography studies on the lead compounds. This effort resulted in the successful determination of three additional co-crystal structures of PDGFRA-G680R in complex with **43,** **44** and **45** (Fig. 5A-F, Supplementary Fig. 4). These structures provide insights into protein-ligand interactions, particularly with the Arg680 residue, and reveal ligand-induced conformational changes of Arg680 (Supplementary Fig. 5). To better understand the implications for inhibitor binding and resistance, we first compared these structures with those of avapritinib and other solvent-front mutated kinases. An alignment of the avapritinib co-complex structure with the crystal structures of the quinazoline derivative **43** in complex with PDGFRA-G680R clearly demonstrates the steric clash between avapritinib’s methylpyrazole moiety with the enlarged and charged side chain of Arg680 (Fig. 5A). Hence, this solvent-front mutation introduces a barrier to inhibitor binding by disrupting the favored binding pose and introducing a high steric demand paired with different electrostatic properties. In general, solvent-front mutations often involve G-to-X substitutions, where the small, flexible glycine is replaced by sterically demanding and charged residues such as arginine^21^. Structural alignments of solvent-front G-to-R mutations reveal that these changes disrupt a key feature of kinase inhibitors: the hinge-binding scaffold. Similar to PDGFRA-G680R, these mutations seem to predominantly confer drug resistance through steric and electrostatic alterations at the entrance of the ATP-binding pocket (Fig. 5B). The resulting changes in the spatial and physicochemical properties of this region create a mismatch with the tailored binding motif of previously potent inhibitors. Beyond local effects, hydrophobic-to-charged substitutions at regulatory hotspots, such as the hinge region or AL, can fundamentally alter kinase activity. Taking EGFR-L858R as a paradigm, such mutations disrupt the native geometry that stabilizes the inactive conformation, thereby favoring more active kinase conformations. Additionally, the introduced positive charge of Arg680 might enhance ATP binding affinity through electrostatic guidance, thereby contributing to the resistance profile observed with these mutations. Hence, although the sequence and structure of the target kinases differ in all cases, the seemingly generalizable resistance mechanism suggests that similar design principles could be applied across different kinases bearing solvent-front mutations. Nevertheless, the effectiveness of different starting strategies depends on the specific situation. A common approach is to avoid the introduced steric conflict. However, our SAR studies revealed that reducing the size of the ligands at this position was insufficient to restore potency, as this strategy comes at the cost of critical binding interactions, leading to a net loss of affinity. Therefore, overcoming this resistance mutation necessitates gaining binding energy through alternative interactions beyond alleviating steric conflict. Another established approach is the use of macrocyclization strategies, as exemplified by emerging ALK inhibitors such as lorlatinib^21,23^. However, challenges remain, including synthetic accessibility, off-target toxicity and the difficulty of maintaining drug-like physicochemical properties without interfering with key pharmacophore elements^24,25^. Meanwhile, the structure-based scaffold hopping strategy provides a rational and flexible framework for addressing solvent-front mutations. If available, this approach leverages prior knowledge and structural insights, enabling targeted modification of mutation-affected regions while preserving key pharmacophoric interactions. Thus, it enables the development of advanced inhibitors by building on existing molecules rather than starting from scratch, accelerating the process. In the case of PDGFRA, our SAR analysis underscored the critical importance of optimized pharmacophoric features at the solvent-accessible entrance of the binding site and established quinazolines as a privileged scaffold. Notably, ether-containing substituents at this position consistently yielded higher potency, provided they adopt a precise orientation that complements the topography of the G680R variant. Indeed, the extended ether chain of **44** was observed to induce a shift of approximately 4.7 Å compared to the **43**-bound structure (Fig. 5F). This structural displacement illustrates that the increased ether length, especially at the 6′-position, does not effectively engage the solvent-front mutation, which explains the reduced potency observed (Table 1). In addition, the previously observed hydrogen bond between the methoxyethoxy group and the backbone of Asp677 in KIT-wt is absent in the PDGFRA-G680R co-complex structure (Supplementary Fig. 6). In contrast, the crystal structure of **43** showed that the di-methoxy ether moiety achieves an excellent shape complementarity with the altered solvent front, contributing to its improved potency against this mutation (Fig. 5C). Notably, a comparison of the co-crystal structures of compound **43** bound to PDGFRA-G680R and its close analogue, compound **34**, bound to KIT-wt, revealed no differences in binding mode (Fig. 5D). The same is true for methoxy hydroxy **45**, whose interaction pattern remained unchanged between the KIT-wt and PDGFRA-G680R structures (Fig. 5E). These structural findings also support the hypothesis that this arginine conformation is energetically favored, especially when compared to the conformer observed in the avapritinib co-complex structure. Despite the substantial alteration introduced by the Arg680 side chain, the inhibitors do not undergo major reorientation to accommodate the mutation. Rather, their potency appears to stem from a preconfigured pharmacophore that engages the mutant PDGFRA-G680R solvent front effectively without requiring substantial induced fit or adaptive binding.

Overall, the structural characterization of the PDGFRA-G680R solvent-front mutation reveals steric and electrostatic changes at the ATP-binding site that mirror resistance-conferring features observed in other kinases. The combination of structure-based scaffold hopping with late-stage derivatization has enabled a targeted tuning of both binding and physicochemical properties. This strategy offers a flexible and rational framework to design next-generation inhibitors capable of overcoming the challenges posed by solvent-front mutations while allowing adjustment of pharmacokinetic parameters. Notably, emerging strategies such as covalent targeting of non-cysteine residues, including recent reports of chemotypes capable of covalently engaging arginine side chains,^26,27^ will greatly benefit from the SAR and structural insights presented here. Together, these data contribute to the design of mutation-specific inhibitors to tackle solvent-front resistance and provide an approach that may extend to other oncogenic kinases bearing solvent-front mutations.

### Cellular evaluation confirms potent, selective inhibition of oncogenic PDGFRA signaling

**Fig. 6:**
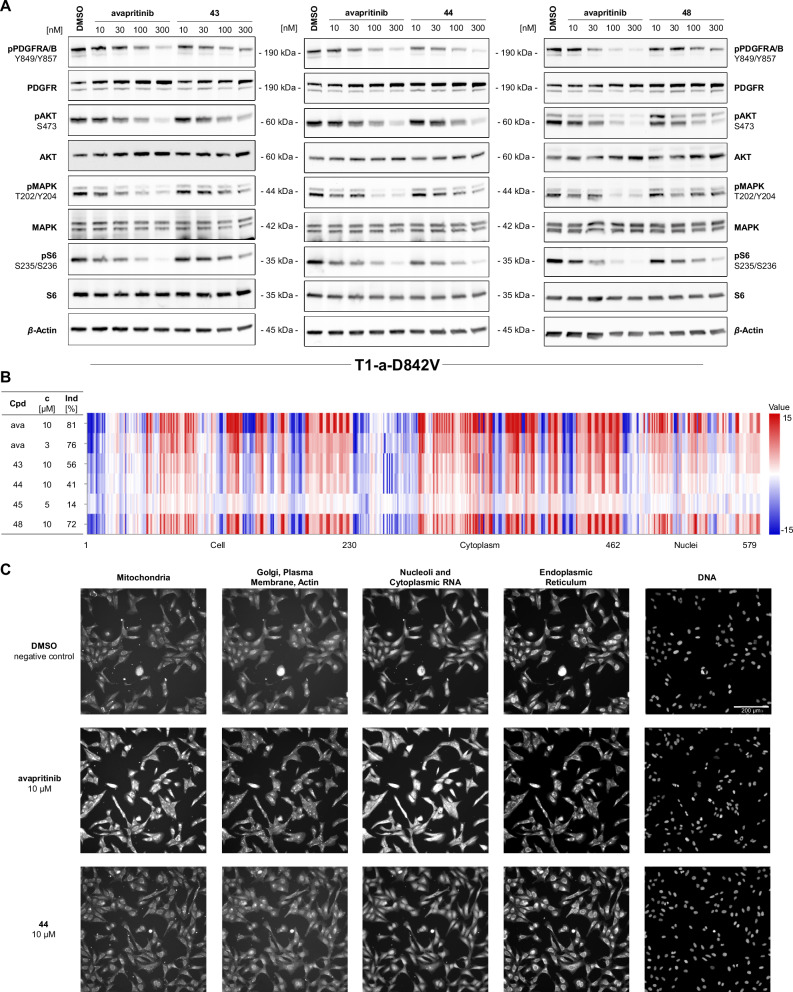
Cellular selectivity profile, target engagement and downstream inhibition of lead compounds compared to avapritinib. **A** Western blot analysis to elucidate dose-dependent inhibition of phospho-PDGFRA-D842V and downstream pathway in T1-a-D842V cells. The cells were treated with DMSO (negative control) or avapritinib, **44, 45** or **48**, respectively for 24 h, lysed, blotted and incubated with PDGFRA and pPDGFRA/B specific antibodies as well as downstream protein-specific antibodies (*n* = 1). **B** Heat map of cell painting profile (Z-scores) performed in U2OS cells treated for 20 h with avapritinib, **43**-**45** and **48**. Each feature was normalized to the median of the DMSO 0.5 % (negative control). Blue indicates a decrease in feature median absolute deviation compared to the negative control, and red indicates an increase in feature median absolute deviation compared to the negative control. The set of 579 features is divided in features related to the compartments: cell (1-229), cytoplasm (230-461) and nuclei (462-579). The bar plot indicates the induction value, calculated as the fraction of features with changes greater than three median absolute deviations from the control. Representative profiles were generated from avapritinib (10 µM, *n* = 4), compound **45** (5 µM, *n* = 2), and compounds **43** (10 µM), **44** (10 µM), and **48** (10 µM) (each *n* = 1) biological experiments. Each biological replicate consisted of three technical replicate wells. **C** Representative images of U2OS cells stained with the cell painting dyes (Mitochondria; Golgi, plasma membrane, and actin; Nucleoli and cytoplasmic RNA; Endoplasmic reticulum; DNA), which were treated with avapritinib (10 µM), **44** (10 µM) or DMSO (0.5 %). Scale bar = 200 µm. Source data are provided as a Source Data file.

Ultimately, thorough cellular evaluation was conducted using CRISPR/Cas9-engineered isogenic GIST cell models harboring clinically relevant primary (T1-a-D842V) and secondary (T1-a-DV-G680R) PDGFRA mutations, alongside a non-KIT/PDGFRA-dependent control cell line (GIST-48B) to assess off-target effects. The SAR trends observed in biochemical assays translated to the cellular setting (Supplementary Table 2). For instance, type III inhibitors lacking hinge-binding motifs exhibited minimal activity, underscoring the importance of hinge interactions. Similarly, stepwise truncation of avapritinib to alleviate the steric conflict with Arg680 failed to restore potency, highlighting that steric compatibility alone is insufficient to achieve meaningful inhibition. While this strategy modestly improved activity against G680R, it led to a ca. 10-fold loss of potency against D842V, underscoring the challenge of optimizing spatial fit without compromising critical molecular interactions. The quinazoline-based inhibitors consistently showed higher cellular potency, particularly methoxy substitutions further enhancing inhibition across mutant models. Based on these findings, the four most promising inhibitors (**43**-**45** and **48**) were selected for direct comparison with clinically relevant reference compounds (Table 2). The isogenic GIST cell models recapitulated the clinically observed resistance against the type II inhibitors imatinib, sunitinib, regorafenib and ripretinib, all of which failed to achieve meaningful inhibition against T1-a-D842V. Similarly, recently reported next-generation inhibitors NB003 and IDRx42 exhibited limited activity, with GR_50_ values exceeding 100 nM and 1000 nM, respectively. In contrast, avapritinib exhibited a GR_50_ of 17 nM against D842V, reflecting its best-in-class status against this genetic lesion. The four lead quinazoline-based inhibitors (**43**-**45** and **48**) matched avapritinib, with GR_50_ values ranging from 9-16 nM, establishing them among the most potent compounds against this activating mutation. The drug-resistant double mutant (D842V/G680R) remained unsensitive for all approved drugs again showing GR_50_ values > 2000 nM. Among the tested quinazolines, compound **45** achieved sub-micromolar inhibition (GR_50_ < 1000 nM), marking a notable, though not yet clinically sufficient, advance in overcoming this secondary resistance mutation. Nevertheless, the biochemical and cellular evaluation suggests that compound **45** exhibits best-in-class inhibitory potency against PDGFRA-D842V and D842V-G680R. Furthermore, the quinazoline inhibitors demonstrated markedly reduced sensitivity in the non-KIT/PDGFRA-dependent GIST-48B control line, suggesting an improved and excellent selectivity profile (Table 2, Supplementary Fig. 7). To further investigate kinome selectivity of the lead compounds, a chemical proteomics approach was performed^28^. Kinobead-based affinity enrichment was conducted using a mixed lysate from five cancer cell lysates, providing coverage of more than 350 kinases, while exhibiting low KIT and PDGFRA expression. Dose-dependent apparent K_d_ values were determined to identify potential off-targets. PDGFRB emerged as the only high-affinity target, with apparent binding below 1 μM. Additional kinases, including FLT3, CSF1R, and RET, displayed moderate affinity in the low-micromolar range, whereas all other detected kinases exhibited weak binding with apparent affinities above 1 μM (Supplementary Figs. 8 and 9). Encouraged by the strong biochemical and cellular profile, we further evaluated three lead inhibitors for cellular target engagement using Western blot analysis in T1-a-D842V cells, alongside avapritinib and a DMSO control (Fig. 6A). These studies demonstrated dose-dependent suppression of phospho-PDGFRA, confirming effective on-target inhibition. Compounds **43** and **48** exhibited comparable potency to avapritinib at 30-100 nM, with **44** achieving greater inhibition of phospho-PDGFR at 10-30 nM. Importantly, the observed inhibition propagates to block downstream signaling pathways as evidenced by reduced phosphorylation of AKT, MAPK and S6. These findings corroborate the CTG cell viability data and further support the ability of these compounds to act as selective chemical probes for investigating resistance mechanisms while highlighting their therapeutic potential. To substantiate the enhanced selectivity of our lead inhibitors, we evaluated them along with avapritinib in non-KIT/PDGFRA dependent human osteosarcoma (U2OS) cells using the cell painting assay (CPA). CPA is a multiparametric phenotypic screening method that evaluates cellular morphology to simultaneously probe multiple biological pathways, making it highly sensitive to subtle changes in cell architecture induced by compound treatment^29^. A heatmap generated from the high-content imaging data revealed distinct morphological signatures between avapritinib and the lead compounds (Fig. 6B). While avapritinib induces a spectrum of cellular changes, in particular, correlating to interference with the lysosomotropic cholesterol homeostasis (LCH), **43**-**45** showed a more restrained effect profile, with fewer and less pronounced morphological shifts. Avapritinib induces changes in the Golgi highlighted by the higher values of intensity and texture on the cytoplasm (Supplementary Table 3 for feature values), consistent with the LCH profile. At the same time, the area of the cytoplasm and nuclei is reduced compared to the control, suggesting reduction in the overall cell size, also noticeable in the representative images (Fig. 6C). For the lead compounds tested at the same concentration as avapritinib, subtle changes in similar features, but at a smaller scale, were observed. This effect was quantified via the induction value, which indicates the percentage of significantly changed features relative to the DMSO control (MAD > 3), as presented in the heat map (Fig. 6B). For example, avapritinib induced higher intensity values than the negative control in DNA-, RNA-, and cytoskeletal-associated features, with Z-scores ranging from 5 to 12, suggesting a broad perturbation of nuclear and cytoplasmic organization. In contrast, compound 44 remained close to baseline for these same features with Z-scores around 0 (Supplementary Table 3). Consistent with the heatmap analysis, PCA visualization of the cell painting profiles demonstrated that compound 43-45 clustered closely, 48 showed larger intra-group distances, while avapritinib remained the most distant profile, indicative of a more restrained off-target morphological impact (Supplementary Fig. 10, Supplementary Table 4). For example, **44** induced a minor shift in nuclear size of six Z-score units compared to avapritinib, which induced a drastic reduction in nuclear size of 23 Z-score units. Overall, the lead compounds induced less changes in the control cells than avapritinib as further underlined by the lower induction values (Fig. 6B). Notably, the concentration of a significant morphological effect induced by the compounds aligns with the GR_50_ values obtained from the CTG in the 48B control cell line (Supplementary Fig. 11).

**Table 2:** Determined GR_50_ values for the lead compounds and references against primary (D842V) and secondary (G680R) as well as control (48B) cell lines

Cpd	CTG GR_50_ [nM]			
	T1-a-DV	T1-a-DV + GR	GIST-48B	Selectivity ratio (48B/DV)
**imatinib**	1200 ± 40	9500 ± 400	>10,000	8
**sunitinib**	1100 ± 80	3600 ± 500	2900 ± 200	3
**regorafenib**	380 ± 90	3200 ± 200	3000 ± 500	8
**ripretinib**	840 ± 100	4600 ± 200	1600 ± 50	2
**avapritinib**	17 ± 2	2200 ± 300	1900 ± 200	112
**NB003**	190 ± 10	>10,000	>10,000	53
**IDRx42**	1700 ± 200	4200 ± 90	3500 ± 100	2
**43**	16 ± 6	1900 ± 400	>10,000	625
**44**	9 ± 1	5400 ± 600	>10,000	1111
**45**	10 ± 1	860 ± 100	>1000	n.a.
**48**	11 ± 3	1800 ± 100	3400 ± 100	309

Data presented as mean values ± s.d; *n* ≥ 3, where *n* represents the number of independent experiments. Certain compounds display a different concentration range depending on the stock concentration (10,000 nM for 10 mM stock vs. 1000 nM for 1 mM stocks). GR_50_ values are reported with up to two significant figures, and the associated standard deviation with one significant figure. n.a. not applicable. Source data are provided as a Source Data file.

These findings further underline that our inhibitors exhibit an improved selectivity profile, likely minimizing undesired off-target toxicity while still maintaining efficacy through their intended mechanism of action. This selectivity is not only important for the therapeutic application, as the reduced off-target toxicity could translate into a more favorable therapeutic index, but also for the application as chemical tools to study the biological principles and effects of mutant-PDGFRA-dependent GIST.

## Discussion

The identification of KIT and PDGFRA as primary oncogenic drivers of GIST has enabled targeted therapies and has transformed patient care. However, off-target toxicities and the emergence of acquired resistance mutations continue to pose major clinical challenges. In this study, we employed a structure-based scaffold-hopping strategy to design type 1.5 inhibitors targeting mutant KIT and PDGFRA, yielding deeper insights into the structural determinants of inhibitor binding and resistance. We identified 6,7-substituted quinazoline inhibitors targeting the G*α*-pocket as a highly promising chemotype for selective mutant KIT and PDGFRA inhibition. The SAR revealed distinct trends, providing insights into protein-ligand binding to mutant-KIT and PDGFRA, as well as insights into the impact of the solvent-front mutation. It appears that a truncated hinge-binding element or bypassing the steric conflict alone is not sufficient to effectively overcome this mutation. Initial scaffold comparisons revealed that hinge-binding geometry and hydrogen bonding capacity are critical for potency, with quinazolines offering enhanced hydrophobic interactions and favorable kinase entrance engagement. Subsequent SAR investigations demonstrated that electron-rich and sterically balanced substituents at the 7′-position, especially methoxy and ether groups, improve potency against mutants such as KIT-D816H, PDGFRA-D842V or -G680R. Importantly, substitutions at the 6,7-positions further increased potency and selectivity, and led to compounds with sub-nanomolar mutant potency and wild-type selectivity. Comprehensive SAR profiling and intensive structural biology efforts, comprising 14 co-crystal structures, including the crystal structure of the solvent-front PDGFRA-G680R mutation, identified critical interactions governing potency and selectivity. Most notably, the four co-crystal structures of PDGFRA-G680R provide a framework for understanding and targeting solvent-front mutations across oncogenic kinases. The resulting 6,7-quinazoline-based inhibitors demonstrated sub-nanomolar cellular potency against PDGFRA-D842V and effective suppression of downstream signaling, confirming on-target activity. In addition to their high potency, compounds **43**-**45** exhibited a more restrained phenotypic impact in non-KIT/PDGFRA-dependent U2OS cells and reduced cytotoxicity in the KIT-independent control line GIST-48B compared to currently approved TKIs. While further in vivo and pharmacokinetic studies are required to define their translational potential, these compounds represent valuable selective tools to investigate drug resistance in KIT/PDGFRA-driven cancers such as GIST or mastocytosis. Collectively, our findings offer mechanistic and structural insights into drug resistance and establish a promising foundation for the development of next-generation inhibitors targeting mutant KIT and PDGFRA, as well as other oncogenic kinases bearing solvent-front mutations.

## Methods

### HTRF assay

#### Reagents and materials

White, F-bottom, 384-well small volume plates (25 μL fill volume) were obtained from Greiner Bio-One GmbH (Solingen, Germany). Revvity (Waltham, MA, United States of America) was the source of all supplies for the KIT and PDGFRA HTRF assay kit and the active enzymes were purchased from Invitrogen (PDGFRA-D842V (PV4203, #2343231B), ProQinase (KIT-wt (0997-0000-1 (010)), KIT-D816H (#1041-0000-1 (002)), PDGFRA-wt (#1057-0000-1)), and SignalChem (PDGFRA-G680R (#X3408-18))).

#### Activity-based assay

The half-maximal inhibitory concentrations (IC_50_) were determined biochemically using the HTRF KinEASE TK assay (Revvity), following the manufacturer’s protocol. Assay conditions for KIT-wt, KIT-D816H, PDGFRA-wt, PDGFRA-D842V and PDGFRA-G680R were based on previously published parameters^15,22^ and are summarized in Supplementary Table 5.

In brief, 5 µL kinase solution and 2.5 µL inhibitor solution (8 % DMSO in HTRF buffer) were incubated for 0.5 h. Subsequently, 2.5 µL of the starting solution containing ATP and substrate was added, and the reaction was allowed to proceed. After reaction completion, 10 µL of stop solution was added. FRET signals were measured using an EnVision plate reader (PerkinElmer, Waltham, MA, USA) and the ratio of the intensities (λ ex 665 nm/λ em 620 nm) was recorded across eight different inhibitor concentrations. IC_50_ values were determined by fitting the data to a Hill 4-parameter equation using the Quattro software (Quattro Research GmbH, Martinsried, Germany). All reactions were performed in duplicate, with at least three independent experiments conducted for each IC_50_ determination. Exemplary dose-response curve fits for the lead compounds, which were used to determine the IC_50_ values against KIT and PDGFRA variants, are shown in Supplementary Fig. 12.

### Protein production, crystallization and structure determination

Construct design, protein expression and purification were done as published previously,^15^ unless otherwise stated.

#### Construct design, protein expression and purification of PDGFRA-G680R

For crystallization studies, codon-optimized DNA encoding residues 550-973 of human PDGFRA (Uniprot-ID: P16234) including an N-terminal His_10_-tag and a TEV protease cleavage site was cloned into a pIEX/Bac-3 expression vector. The construct is based on the published crystal structure of the PDGFRA kinase domain (PDB-ID: 5GRN), including a deletion of the kinase insert loop within the C-terminal sub domain (amino acids 697-768). The mutation was introduced into a WT construct using PCR mediated mutagenesis (forward mutagenesis primer CCGAGTACTGCTTCTACCGTGACCTGGTCAACTACCTG; reverse mutagenesis primer CAGGTAGTTGACCAGGTCACGGTAGAAGCAGTACTCGG).

Transfection, virus generation, amplification and protein expression were carried out in *S. frugiperda* (Sf9) cells (Thermo Fisher Scientific). After expression, cells were harvested (4 °C, 3000 g, 20 min), resuspended in lysis buffer (20 mM Tris pH 8.0, 150 mM NaCl, 20 mM imidazole, 0.1 % Triton X-100, 5 mM β-mercaptoethanol (BME), protease inhibitor cocktail complete EDTA-free (Roche)) and lysed by sonication. After centrifugation (4 °C, 75,000 g, 1 h), the supernatant was loaded onto a HisTrap FF crude (Cytiva) column and eluted with an imidazole gradient (Buffer A: 20 mM Tris pH 8.0, 150 mM NaCl, 5 mM BME; Buffer B: 20 mM Tris pH 8.0, 150 mM NaCl, 500 mM imidazole, 5 mM BME). Fractions containing PDGFRA-G680R were combined, and for removal of the imidazole, the protein solution was dialysed (20 mM Tris pH 8.0, 150 mM NaCl, 5 mM BME) and simultaneously incubated with TEV protease (ratio protein 5:1 protease) to remove the His_10_-tag, followed by a second nickel-affinity chromatography collecting the flow through. In a final step, the protein was purified using a S75 16/600 size exclusion chromatography column (20 mM Tris pH 8.0, 200 mM NaCl, 1 mM TCEP) and concentrated to 13.5 mg/mL. The mass of the protein was confirmed by ESI-MS analysis.

#### Construct design, protein expression and purification of KIT-wt

Codon-optimized DNA encoding residues 551-934 of human KIT (Uniprot-ID: P10721) including an N-terminal His_6_-tag and a thrombin or a TEV (tobacco etch virus) protease cleavage site was cloned into a pCDFDuet-1 expression vector together with the coding sequence for the phosphatase YopH (Uniprot-ID: P15273, residues 164-468). The thrombin cleavage site containing and the TEV cleavage site containing KIT-constructs only differ by one amino acid at the N-terminus after tag-removal (GSM vs. GS), which has no detectable impact on the crystallization behavior or the resulting structures (9TFK has been obtained using the construct containing a TEV cleavage site, all other KIT structures reported here have been obtained using the construct containing a thrombin cleavage site). The constructs are based on the published crystal structure of the KIT kinase domain (PDB-ID: 6GQK) and include several amino acid substitutions and a deletion of the kinase insert loop within the C-terminal sub domain (amino acids 688-765) that is replaced by EFVPYKVAPEDLYKDFLT.

For protein production, the construct containing the thrombin cleavage site has been expressed and purified as described previously^15^. The expression and purification of the KIT construct containing the TEV cleavage site was performed accordingly, with the following exceptions: The incubation step with 1 % CHAPS has been omitted and for the lysis and the Ni-NTA affinity chromatography 50 mM Tris pH 8.0, 10 % glycerol, 300 mM NaCl and 5 mM BME has been used as buffer (instead of 40 mM HEPES pH 8.0, 10 % glycerol, 300 mM NaCl and 1 mM TCEP). TEV protease (ratio protein 20:1 protease) has been added to the eluted protein for the removal of the His_6_-tag and simultaneously, protein has been dialyzed against 20 mM Tris pH 8.0, 300 mM NaCl, 10 % glycerol, 5 mM BME. All subsequent steps (size exclusion chromatography, ESI-MS analysis) have been performed as described previously.

#### Crystallization experiments of PDGFRA-G680R and KIT-wt

Before crystallization, the proteins were incubated with a 2-fold excess for PDGFRA-G680R and 3-fold excess for KIT-wt of the corresponding ligands for 3 h at 4 °C. The proteins were crystallized at a concentration of 5–10 mg/ml for PDGFRA-G680R and 3 mg/ml for KIT-wt using the vapour diffusion hanging drop method at 285.15 K or 293.15 K, using 1 µl of the protein+ligand solution and 1 µl of the reservoir solution. For data collection, crystals were usually cryoprotected by increasing the PEG3350 concentration to 35 % or by adding 20-25 % ethylene glycol or glycerol before freezing. The crystallization conditions for PDGFRA-G680R and KIT-wt can be found in Supplementary Tables 6 and 7.

#### Structure determination and refinement

Diffraction data were processed using XDS^30^. Structures of PDGFRA-G680R and KIT-wt in complex with ligands were determined by molecular replacement with PHASER^31^ using structure PDB-ID: 8PQH (PDGFRA) and PDB-ID: 8PQ9 (KIT) as a search model. Molecules in the asymmetric unit were manually adjusted using COOT^32^ and the structures were refined with Phenix.refine. Inhibitor topology files were generated using eLBOW of the Phenix program package^33^. The refined structures were validated with the PDB validation server. PyMOL (W.L. DeLano, The PyMOL Molecular Graphics System) was used for generating figures.

### In silico analysis of novel type 1.5 inhibitors

#### In silico calculation of physicochemical properties

MarvinSketch was used to draw, display and characterize chemical structures in terms of their physicochemical parameters Marvin 22.13.0, Chemaxon (https://www.chemaxon.com).

#### Generation of pharmacophore models

To investigate protein-ligand interactions between crystallized ligands and target proteins, pharmacophore models were generated using LigandScout (v.4.4.8)^34^. Two-dimensional pharmacophore models were created for each ligand bound to either KIT-wt or PDGFRA-G680R using the standard settings.

#### Generation of schematic 2D protein-ligand interactions representations

Schematic two-dimensional depictions of protein–ligand interactions were generated using LIGPLOT (v2.3.1)^35^. For each crystallized ligand, interaction maps were produced using default parameters with automated water molecule detection enabled. Interaction diagrams were generated separately for complexes with KIT-wt and PDGFRA-G680R.

### Cell culture and CTG assay

Primary (T1-a-D842V) and secondary (T1-a-D842V + G680R) on-target mutant PDGFRA-dependent cell lines, along with non-target dependent control cell lines GIST-48B (RRID: CVCL_M441)) and U2OS (RRID: CVCL_0042) were used in this study. Gene editing by CRISPR-Cas9 has been performed as described^16^ to afford the T1-a-D842V (PDGFRA exon 18, D842V) and the T1-a-D842V + G680R (PDGFRA exon 18, D842V, and solvent-front G680R). The GIST-48B exhibits minimal expression of KIT transcript and protein, with levels that are essentially undetectable^36^.

The cell lines were cultured in IMDM containing 10 % FBS and 1 % Penicillin/Streptomycin and incubated at 37 °C and 5 % CO_2_. In the course of this study, all cell lines were subjected to routine testing for mycoplasma contamination using PCR and the MycoAlert Mycoplasma Detection Kit (Lonza) as well as an evaluation of the response to established GIST inhibitor treatment.

The cell viability was assessed via the CellTiter-Glo (CTG) assay (Promega, Madison, WI), following the manufacturer’s protocol. Luminescence was measured with an Envision plate reader (Revvity). Curve fitting was performed, and GR_50_ values were calculated using the Quattro Workflow software (Quattro Research GmbH), based on the average of more than two independent experiments.

### Kinobeads selectivity profiling

*Cell culture and lysis*. K-562, COLO-205 and MV-4-11 cells were cultured in Roswell Park Memorial Institute 1640 medium (Biochrom). SK-N-BE(2) cells were cultured in DMEM/Ham’s F-12 (1:1), and OVCAR-8 cells in Iscove’s modified Dulbecco’s medium (Biochrom GmbH). All media were supplemented with 10 % (v/v) fetal bovine serum (Biochrom GmbH). Cells were lysed in 0.8% IGEPAL, 50 mM Tris-HCl pH 7.5, 5 % glycerol, 1.5 mM MgCl_2_, 150 mM NaCl, 1 mM Na_3_VO_4_, 25 mM NaF and 1 mM dithiothreitol (DTT), supplemented with protease inhibitors (SigmaFast, Sigma) and phosphatase inhibitors prepared in house according to Sigma-Aldrich phosphatase inhibitor cocktails 1, 2 and 3. Lysates were clarified by ultracentrifugation and diluted in lysis buffer lacking IGEPAL to a final protein concentration of 5 mg ml^−1^, as determined by Bradford assay, before mixing in a 1:1:1:1:1 ratio.

*Kinobeads pulldowns*. Kinobeads pulldown experiments were performed as previously described^28,37,38^. Briefly, cell lysates (2.5 mg total protein per pulldown) were pre-incubated with compound at increasing concentrations (DMSO, 3, 10, 30, 100, 300 and 1000 nM, and 3 and 30 µM) for 45 min at 4 °C. Lysates were then incubated with Kinobeads epsilon (17 µl settled beads) for 30 min at 4 °C. To assess protein depletion, the flow-through from the DMSO control pulldown was subjected to a second pulldown with fresh Kinobeads. Beads were washed sequentially with buffers containing 0.4 %, 0.2 % or no IGEPAL. Bound proteins were reduced in 50 mM DTT in 8 M urea, 40 mM Tris-HCl pH 7.4 for 30 min at room temperature and alkylated with 55 mM chloroacetamide. Urea concentration was then reduced to 1 M and proteins were digested overnight with trypsin. Peptides were acidified with 6 µl 10 % formic acid (FA) and desalted using self-packed StageTips containing SCX material (Empore Cation Extraction Disks, Supelco). StageTips were equilibrated with 0.1 % FA in 50 % acetonitrile, washed three times with 150 µl 0.1 % FA in 50% acetonitrile, and peptides were eluted with 100 µl 4 % ammonium formate (pH 6.5) in 50 % acetonitrile. Eluates were lyophilized and dried peptides were stored at −20 °C until LC-MS/MS analysis.

*LC-MS/MS of Kinobeads pulldowns*. Peptides were delivered to a trap column (300 µm × 5 mm, PepMap Neo Trap Cartridge, Thermo Fisher Scientific) and separated on an analytical column (75 µm ID × 8 cm, Aurora Rapid, IonOpticks) using a Vanquish Neo UHPLC system coupled to an Orbitrap Astral mass spectrometer with a Nanospray Flex source (Thermo Fisher Scientific). Solvent A was 0.1 % formic acid in water and solvent B was 0.1 % formic acid in acetonitrile. Peptides were separated at 300 nl min^−1^ using a 15 min gradient from 2 to 32 % B, with an initial step from 2 to 6 % B over 0.5 min at 750 nl min^−1^ and from 6 to 9.5 % B over 2 min while reducing the flow rate to 300 nl min^−1^. The column was washed with 80 % B for 3.5 min and re-equilibrated at 2 % B. The mass spectrometer was operated in data-independent acquisition and positive ionization mode. MS1 spectra were acquired in the Orbitrap mass analyser over a mass-to-charge (m/z) range of 360 –1000 at a resolution of 240,000 every 0.6 s using an automatic gain control (AGC) target value of 3 × 10^6^ charges or a maximum injection time of 5 ms. In parallel, precursor ions were fragmented using 28 % normalized collision energy (NCE), and MS2 spectra were acquired in the Astral mass analyzer across a scan range of 150-2,000 m/z using a 2 Th isolation window, an AGC target of 3 × 10^4^ charges and a maximum injection time of 3 ms.

*Data processing and analysis of Kinobeads pulldowns*. Raw files were analyzed using FragPipe v23, using the DIA_SpecLib_Quant workflow, against all canonical protein sequences as annotated in the UniProtKB reference database of human (20,435 entries, downloaded 28 June 2024). Carbamidomethylated cysteine was set as fixed modification and oxidation of methionine and N-terminal protein acetylation as variable modifications. Stricttrypsin was specified as the proteolytic enzyme and up to two missed cleavages were allowed. The minimum length of amino acids was set to seven and all data were adjusted to 1 % PSM and 1 % protein FDR, and proteins with at least two unique peptides retained for downstream analysis. Dose-response data was evaluated using CurveCurator^39^. Kinobeads selectivity profiling was performed once as a chemical proteomics profiling experiment.

### Cell painting assay

The Cell Painting assay^40^ was performed as described previously^29,41^.

Briefly, U2OS medium (5 μL) was added to each well of a 384-well plate (PerkinElmer CellCarrier-384 Ultra) prior to addition of 1600 U2OS cells per well and incubation for 4 h at 37 °C. Compounds were added to the cells using the Echo 520 acoustic dispenser (Labcyte) followed by incubation for 20 h at 37 °C. Subsequently, mitochondria were stained with 0.1 μg/μL Mito Tracker Deep Red for 30 min at 37 °C in the dark. Cells were fixed using 3.7 % formaldehyde in PBS for 20 min at 37 °C in the dark. Cells were washed three times using PBS before permeabilization using Triton X-100 for 15 min 37 °C in the dark. After three additional washing steps, 25 μL of a staining solution were added to each well, which contained 1 % BSA, 5 μL/mL Phalloidin (Alexa594 conjugate, Thermo Fisher Scientific, A12381), 25 μg/mL Concanavalin A (Alexa488 conjugate, Thermo Fisher Scientific, Cat. No. C11252), 5 μg/mL Hoechst 33342 (Sigma, Cat. No. B2261-25 mg), 1.5 μg/mL WGA-Alexa594 conjugate (Thermo Fisher Scientific, Cat. No. W11262) and 1.5 μM SYTO 14 solution (Thermo Fisher Scientific, Cat. No. S7576). Plates were incubated for 30 min at 37 °C in the dark and washed three times with PBS. Plates were sealed and centrifuged for 1 min at 46 g. The plates were prepared in triplicates with shifted layouts to reduce plate effects and imaged using a Micro XL High-Content Screening System (Molecular Devices) in five channels (DAPI: Ex350-400/Em410-480; FITC: Ex470-500/Em510-540; Spectrum Gold: Ex520-545/Em560-585; TxRed: Ex535-585/Em600-650; Cy5: Ex605-650/Em670-715) with nine sites per well and 20× Ph1 S Plan Fluor ELWD ADM objective (NA 0.45). Images were acquired as 16-bit monochrome TIFF files (1080 × 1080 pixels, binning 2, calibrated pixel size 0.6636 × 0.6636 µm). Representative microscopy images (Fig. 6C) were generated from the raw TIFF images using ImageMagick. Images were resized for figure layout, and linear brightness/contrast adjustments were applied uniformly to all images within each fluorescence channel. Raw images used for quantitative analysis were otherwise unmodified.

Generated images were processed with the CellProfiler package (https://cellprofiler.org, version 3.0.0) on a computing cluster of the Max Planck Society to extract 1716 cell features. The data was then further aggregated as medians per well (9 sites→1 well), then over the three replicates. Further analysis was performed with custom Python (https://www.python.org) scripts using the Pandas (https://pandas.pydata.org) and Dask (https://dask.org) data processing libraries as well as the Scientific Python (https://scipy.org) package. The phenotypic fingerprints were compiled from the Z-scores of all individual cellular features, where the Z-score is a measure of how far a data point is from a median value. Specifically, Z-scores of test compounds were calculated relative to the median of DMSO controls. Thus, the Z-score of a test compound defines how many MADs (Median Absolute Deviations) the measured value is from the median of the controls^42^. The phenotypic compound fingerprint is then determined as the list of Z-scores of all 579 features for one compound.

The induction (in percent) was determined using Eq. (1) as a measure of bioactivity of each compound as the fraction of significantly changed features:

1 $${\mbox{Induction}}\left[\%\,\right]=\frac{{number}\,{of}\,{features}\,{with}\left|.\right|\,{values}\, > 3}{{total}\,{number}\,{of}\,{features}}$$

### PCA and Euclidean distance between cell painting profiles

Morphological profiles were built as described in the Cell Painting Assay section, resulting in Z-scores for each compound across 579 features. Principal component analysis (PCA) was run on the Z-scored matrix with Scikit-learn,^43^ resulting in 2 principal component vectors that together explained 90 % of the total variance. To obtain a single profile per compound, PCA values were averaged across concentrations to generate compound-level centroids. The pairwise Euclidean distance between avapritinib and compounds was calculated between the centroid pairs using the SciPy spatial distance function,^44^ an approach commonly used to quantify distance between Cell Painting fingerprints^45^.

### Western blotting

The western blot experiments were conducted as previously described^10,15^.

In brief, T1-a-D842V cells were plated in 6-well plates and treated the following day with the respective inhibitors or a DMSO control. After 3 h of treatment, cells were lysed using NP-40-containing buffer (1 % NP-40, 50 mmol/L Tris-HCl pH 8.0, 100 mmol/L sodium fluoride, 30 mmol/L sodium pyrophosphate, 2 mmol/L sodium molybdate, 5 mmol/L EDTA and 2 mmol/L sodium vanadate; freshly adding 0.1% 10 mg/mL aprotinin and leupeptin as well as 1 % 100 mmol/L PMSF and 200 mmol/L sodium vanadate). The cells were scraped off and lysed while rotating at 4 °C for 1 h. Protein concentration was adjusted to 2 μg/μL, and SDS-loading buffer (0.5 M Tris-HCl pH 6.7, 10 % SDS, 2.5% DTT, 50 % glycerol, and 0.05 % bromophenol blue) was added. Lysates were incubated at 95 °C for 5 min. Equal protein amounts (30 µg) per lane were separated by SDS/PAGE (NuPAGE, Life Technologies) and transferred to Hybond-P nitrocellulose membranes (Amersham Pharmacia Biotech). Membranes were blocked with Net-G buffer (1.5 M NaCl, 50 mM EDTA, 500 mM Tris, 0.5 % Tween 20, 0.4 % gelatin) and incubated at 4 °C overnight with corresponding primary antibodies. After washing with Net-G, membranes were incubated with secondary antibodies for 2 h at room temperature and washed again. Protein expression and phosphorylation changes were detected by chemiluminescence (WesternBright ECL, Advansta Inc.) and images were captured and quantified using a FUJI LAS-3000 system with Science Lab 2001 ImageGauge 4.0 software (Fujifilm Medical Systems). 2 to 4 gels/membranes were prepared from the same experiment to ensure distinct detection of proteins with similar molecular weights, as well as their total and phosphorylated forms. β-Actin was used as a loading control for each membrane; a representative stain is shown.

### Reporting summary

Further information on research design is available in the Nature Portfolio Reporting Summary linked to this article.

## Supplementary information

Supplementary Information

Reporting Summary

Transparent Peer Review file

## Source data

Source Data

## Data Availability

The data supporting the findings of this study are available in the paper and its Supplementary Information. The crystal structure data generated in this study have been deposited in the PDB database under accession codes 9TF9, 9TFA, 9TFB, 9TFC, 9TFD, 9TFE, 9TFF, 9TFG, 9TFH, 9TFI, 9TFJ, 9TFK, 9TFL and 9TFM. Diffraction data were measured at the ESRF and are available via [10.15151/esrf-dc-2423339802]. Previously reported structures that were used in this publication are deposited under the following accession codes: 1PKG, 7VKM, 8J5X, 8PQ9, 9GBE and 9QEK. The kinobeads mass spectrometry proteomics data have been deposited to the PRIDE repository with the data set identifier: PXD077574. Source data are provided with this paper as a Source Data file. Source data are provided with this paper.

## Code availability

The Cell Painting image analysis and data processing pipeline used in this study is available at 10.5281/zenodo.21492040. The custom scripts used to generate the Cell Painting analyses and figures presented in this manuscript are publicly available under the MIT License under 10.5281/zenodo.21068942.
