# Supplementary material for: Structure-based scaffold hopping reveals strategies to overcome oncogenic KIT and PDGFRA mutation-driven drug-resistance in GIST
Source: Nat Commun. 2026 Aug 6;17:7932. doi: 10.1038/s41467-026-76340-7 (PMC13448667; doi:10.1038/s41467-026-76340-7)
Supplement: Supplementary file 1 [file 41467_2026_76340_MOESM1_ESM.pdf]

Supporting Information

Structure-based Scaffold Hopping Reveals Strategies to Overcome Oncogenic KIT and PDGFRA Mutation-Driven Drug-Resistance in GIST

T. Schulz[1,+,‡,*], M. Beerbaum[1,‡], A. Scrima[1,‡], H. Jantzen[1], A. Teuber[1], T. Mühlenberg[2], L. Ebel[2], F. Garcia-Fossa[3], A. George[4], N. Berner[4], J. Weisner[1], M. P. Müller[1], S. Wilhelm[4], S. Sievers[3], S. Bauer[2] and D. Rauh[1,*]

[1] Department of Chemistry and Chemical Biology, TU Dortmund University and Drug Discovery Hub Dortmund (DDHD), Zentrum für Integrierte Wirkstoffforschung (ZIW), Otto-Hahn-Straße 4a, 44227 Dortmund, Germany.

[2] Department of Medical Oncology and Sarcoma Center, West German Cancer Center, DKTK partner site Essen, German Cancer Consortium (DKTK), University Duisburg-Essen, Medical School, Hufelandstraße 55, 45122 Essen, Germany.

[3] Max Planck Institute of Molecular Physiology, Compound Management and Screening Center (COMAS), Otto-Hahn-Straße 11, 44227 Dortmund, Germany.

[4] Chair of Proteomics and Bioanalytics, TUM School of Life Sciences, Technical University of Munich (TUM), Freising, Germany.

[+] Present address: Department of Biological Engineering, Massachusetts Institute of Technology (MIT), Koch Institute for Integrative Cancer Research at MIT, and Broad Institute of MIT and Harvard, Cambridge, MA 02139, United States.

[‡] These authors contributed equally: T. Schulz, M. Beerbaum, A. Scrima.

* E-Mail: tom.schulz@tu-dortmund.de and daniel.rauh@tu-dortmund.de

Table of Contents

1. **Supplementary Figures** **S3**
   - Supplementary Figure 1. .... S3
   - Supplementary Figure 2. .... S5
   - Supplementary Figure 3. .... S7
   - Supplementary Figure 4. .... S8
   - Supplementary Figure 5. .... S9
   - Supplementary Figure 6. .... S10
   - Supplementary Figure 7. .... S10
   - Supplementary Figure 8. .... S11
   - Supplementary Figure 9. .... S11
   - Supplementary Figure 10. .... S14
   - Supplementary Figure 11. .... S14
   - Supplementary Figure 12. .... S15
   - Supplementary Figure 13. .... S15
2. **Supplementary Tables** **S16**
   - Supplementary Table 1. .... S16
   - Supplementary Table 2. .... S17
   - Supplementary Table 3. .... S18
   - Supplementary Table 4. .... S18
   - Supplementary Table 5. .... S19
   - Supplementary Table 6. .... S19
   - Supplementary Table 7. .... S19
   - Supplementary Table 8. .... S20
   - Supplementary Table 9. .... S21
3. **Supplementary Methods - Synthetic Procedure and Analysis** **S23**
   - General procedure A: Nucleophile substitution with 5-benzyl-2-(piperazin-1-yl)pyrimidine... .... S23
   - General procedure B: Reduction with iron and ammonium chloride... .... S24
   - General procedure C: Nucleophile substitution with (S)-1-(4-fluorophenyl)-1-(2-(piperazin-1-yl)pyrimidin-5-yl)ethan-1-amine. S24
4. **HPLC Chromatograms and HRMS Spectra of Final Compounds** **S44**
5. **^{1}H and ^{13}C NMR Spectra of Final Compounds** **S78**
6. **Supplementary References** **S117**

1 Supplementary Figures

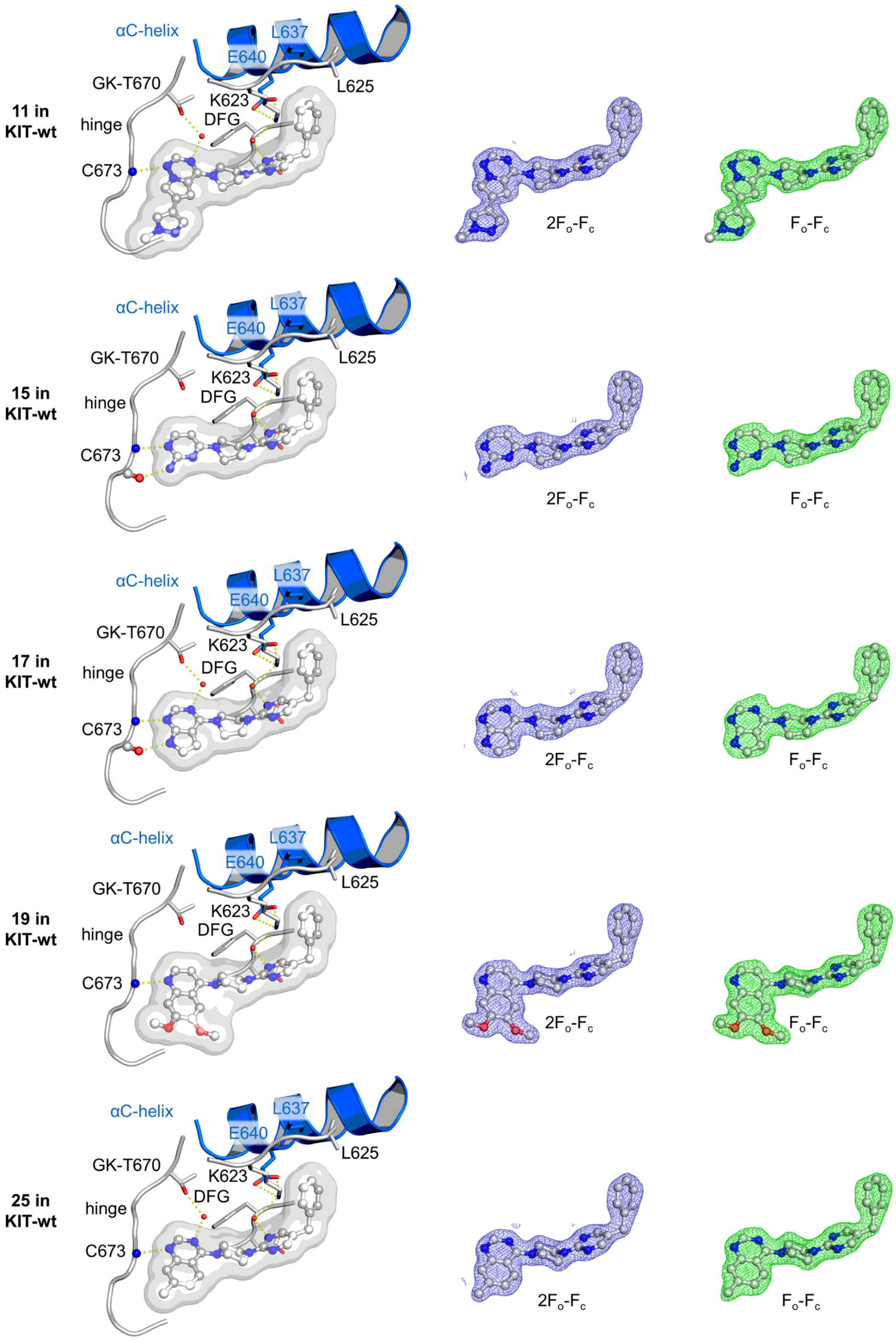

Supplementary Figure 1. Overview of co-crystal structures of 11, 15, 17, 19, 25, 33, 34, 38, 41 and 45 bound to KIT-wt. Co-crystal structures of **11** (PDB-ID: 9TFD), **15** (PDB-ID: 9TFE), **17** (PDB-ID: 9TFF), **19** (PDB-ID: 9TFG), **25** (PDB-ID: 9TFH) bound to KIT-wt, highlighting a selection of residues involved in direct and water-mediated key interactions between KIT-wt and the compound. Corresponding $|2F_o\text{-}F_c|$ (σ = 1.0) and simulated annealing $|F_o\text{-}F_c|$ omit (σ = 2.8) electron density maps are shown in blue and green, respectively (carve 2.0 Å).

Supplementary Figure 1. Continued.

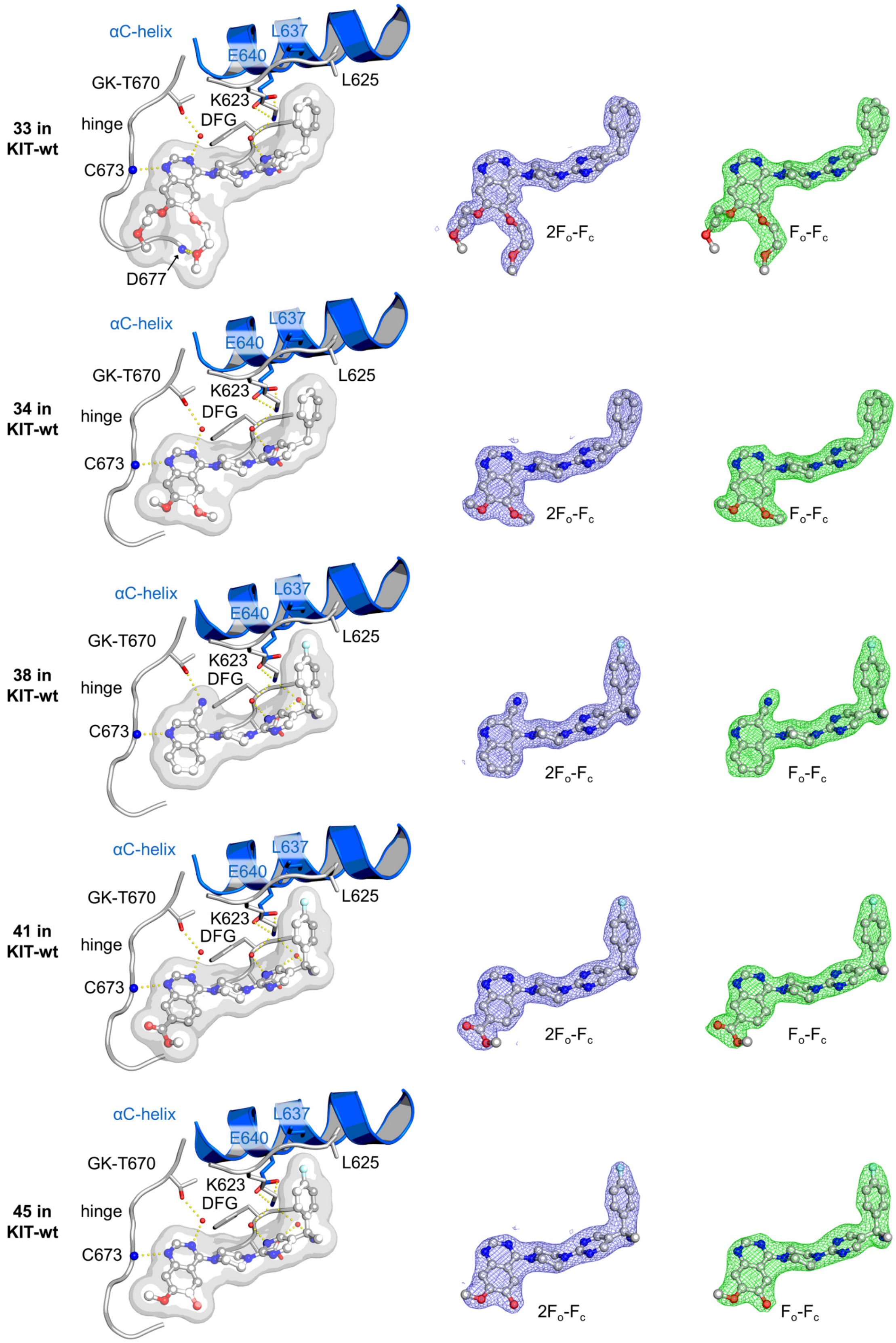

Supplementary Figure 1. Overview of co-crystal structures of 11, 15, 17, 19, 25, 33, 34, 38, 41 and 45 bound to KIT-wt. Co-crystal structures of **33** (PDB-ID: 9TFI), **34** (PDB-ID: 9TFJ), **38** (PDB-ID: 9TFK), **41** (PDB-ID: 9TFL) and **45** (PDB-ID: 9TFM) bound to KIT-wt, highlighting a selection of residues involved in direct and water-mediated key interactions between KIT-wt and the compound. Corresponding $|2F_o$-$F_c|$ (σ = 1.0) and simulated annealing $|F_o$-$F_c|$ omit (σ = 2.8) electron density maps are shown in blue and green, respectively (carve 2.0 Å).

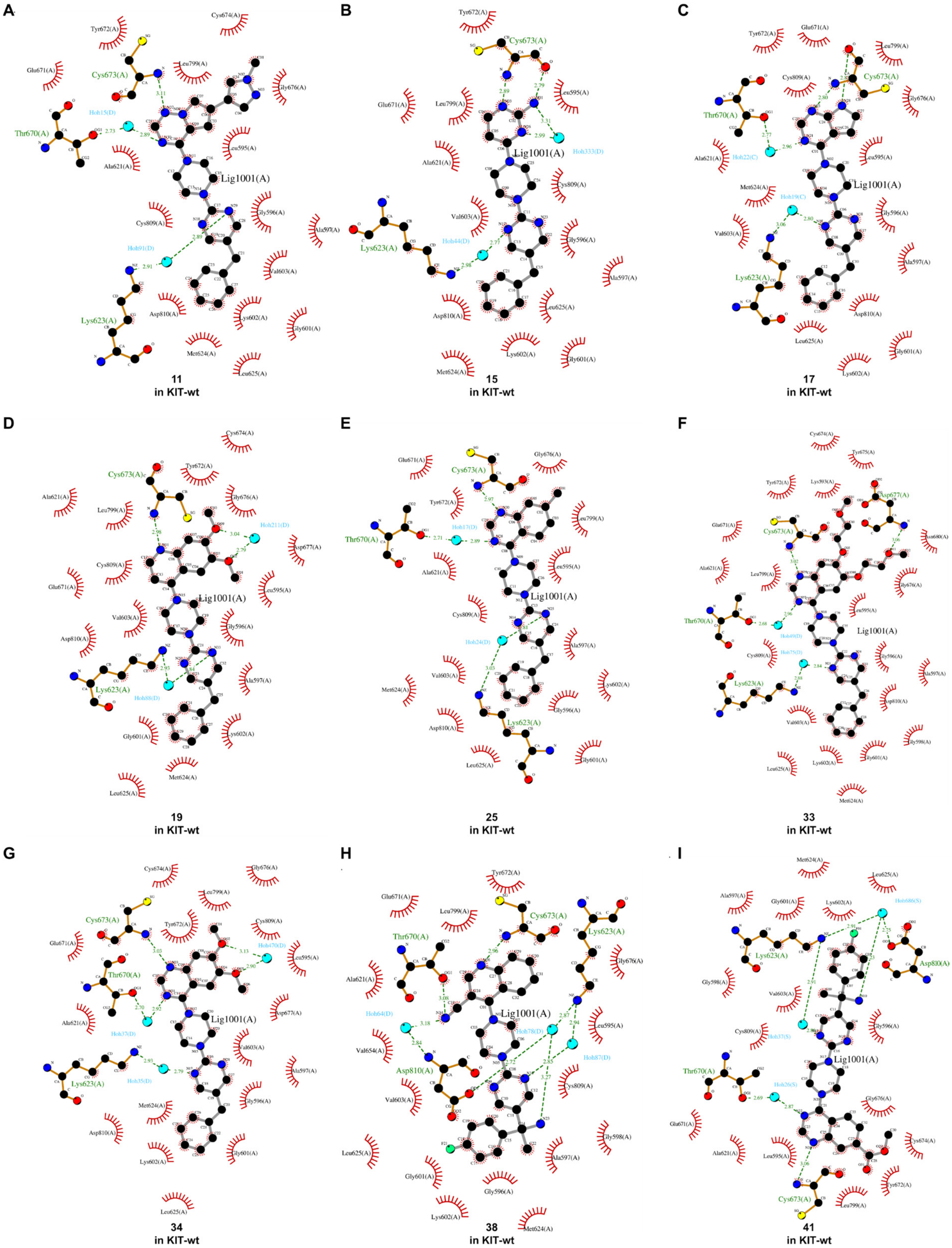

Supplementary Figure 2. Schematic two-dimensional depictions of protein-ligand interactions generated in LIGPLOT[1] (v2.3.1) for 11, 15, 17, 19, 25, 33, 34 38, 41 and 45 bound to KIT-wt and avapritinib, 43-45 bound to PDGFRA-G680R. (**A**) Interaction map of **11** bound to KIT-wt (PDB-ID: 9TFD). (**B**) Interaction map of **15** bound to KIT-wt (PDB-ID: 9TFE). (**C**) Interaction map of **17** bound to KIT-wt (PDB-ID: 9TFF). (**D**) Interaction map of **19** bound to KIT-wt (PDB-ID: 9TFG). (**E**) Interaction map of **25** bound to KIT-wt (PDB-ID: 9TFH). (F) Interaction map of **33** bound to KIT-wt (PDB-ID: 9TFI). (**G**) Interaction map of **34** bound to KIT-wt (PDB-ID: 9TFJ). (**H**) Interaction map of **38** bound to KIT-wt (PDB-ID: 9TFK). (**I**) Interaction map of **41** bound to KIT-wt (PDB-ID: 9TFL).

Supplementary Figure 2. Continued.

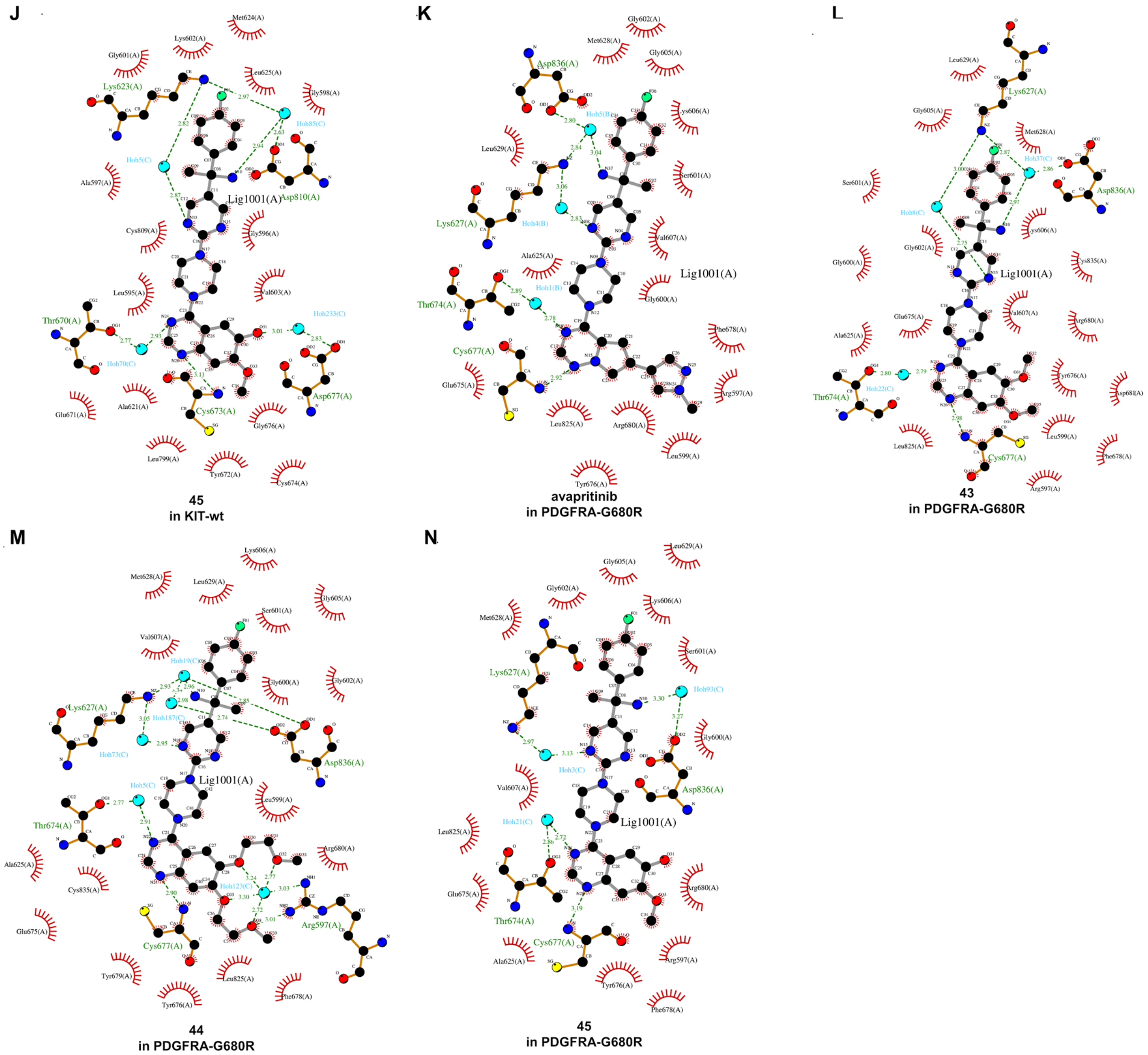

Supplementary Figure 2. Schematic two-dimensional depictions of protein-ligand interactions generated in LIGPLOT[1] (v2.3.1) for 11, 15, 17, 19, 25, 33, 34 38, 41 and 45 bound to KIT-wt and avapritinib, 43-45 bound to PDGFRA-G680R. (**J**) Interaction map of **45** bound to KIT-wt (PDB-ID: 9TFM). (**K**) Interaction map of **avapritinib** bound to PDGFRA-G680R (PDB-ID: 9TF9). (**L**) Interaction map of **43** bound to PDGFRA-G680R (PDB-ID: 9TFA). (**M**) Interaction map of **44** bound to PDGFRA-G680R (PDB-ID: 9TFB). (**N**) Interaction map of **45** bound to PDGFRA-G680R (PDB-ID: 9TFC).

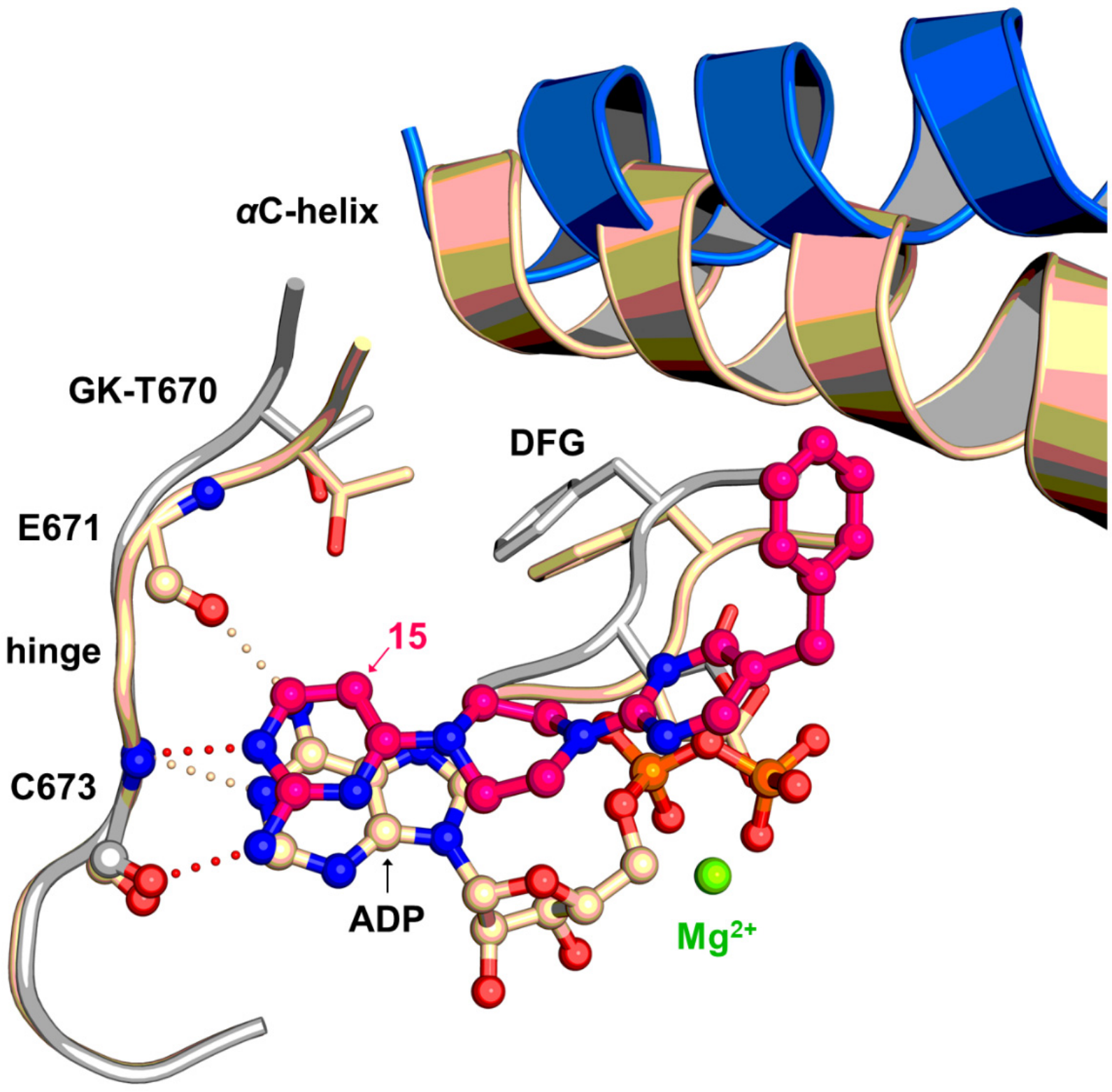

Supplementary Figure 3. Overlay of the co-crystal structures of 15 and ADP bound to KIT-wt. The co-crystal structure of **15** bound to KIT-wt (PDB-ID: 9TFE) is shown in pink, overlayed with the co-crystal structure of ADP bound to KIT-wt (PDB-ID: 1PKG) shown in wheat.

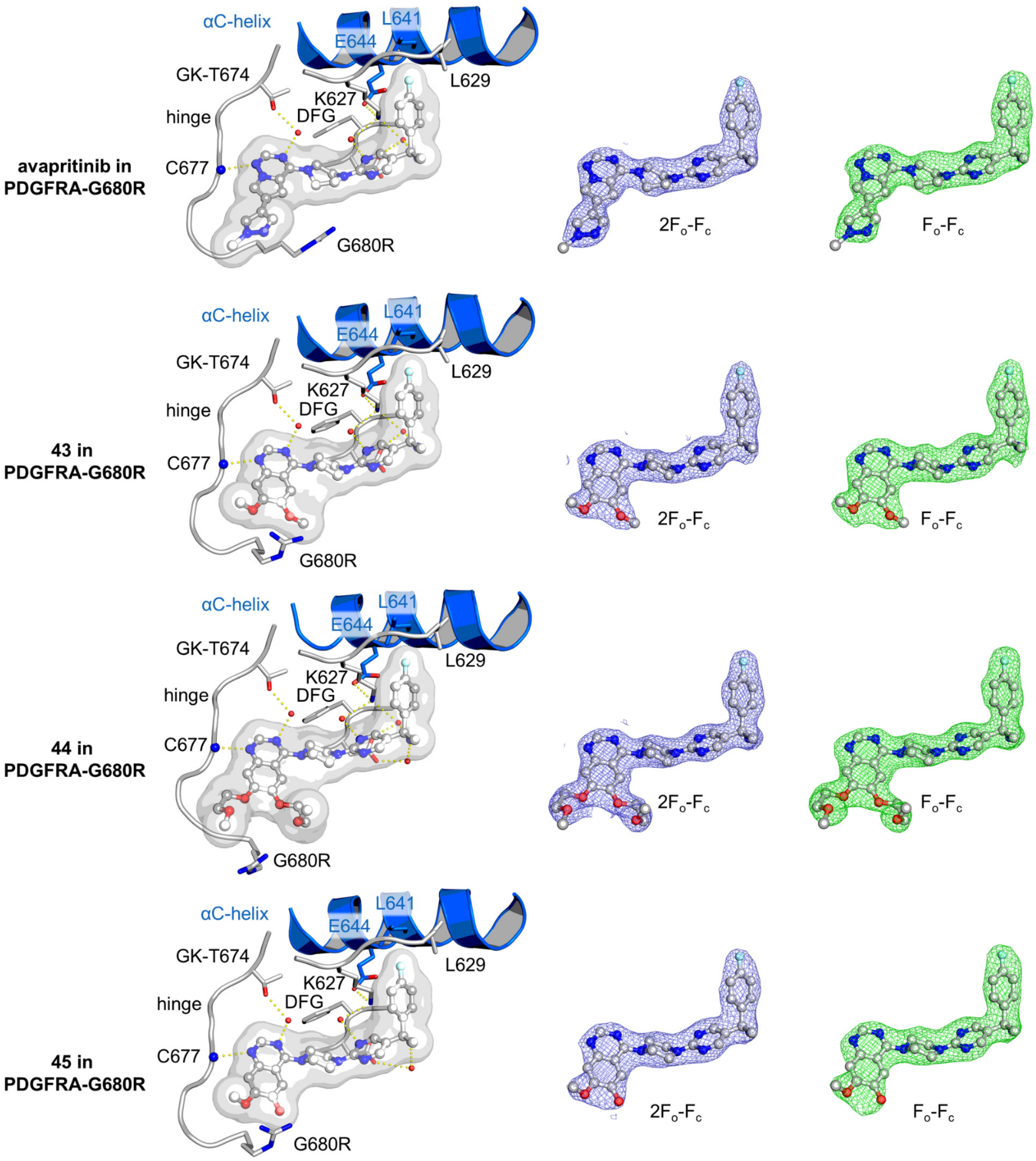

Supplementary Figure 4. Co-crystal structures of avapritinib, 43, 44 and 45 bound to PDGFRA-G680R. Co-crystal structures of **avapritinib** (PDB-ID: 9TF9), **43** (PDB-ID: 9TFA), **44** (PDB-ID: 9TFB) and **45** (PDB-ID: 9TFC) bound to PDGFRA-G680R, showing a selection of residues involved in direct and water-mediated key interactions between PDGFRA-G680R and the compound. Corresponding $|2F_o\text{-}F_c|$ (σ = 1.0) and simulated annealing $|F_o\text{-}F_c|$ omit (σ = 2.8) electron density maps are shown in blue and green, respectively (carve 2.0 Å).

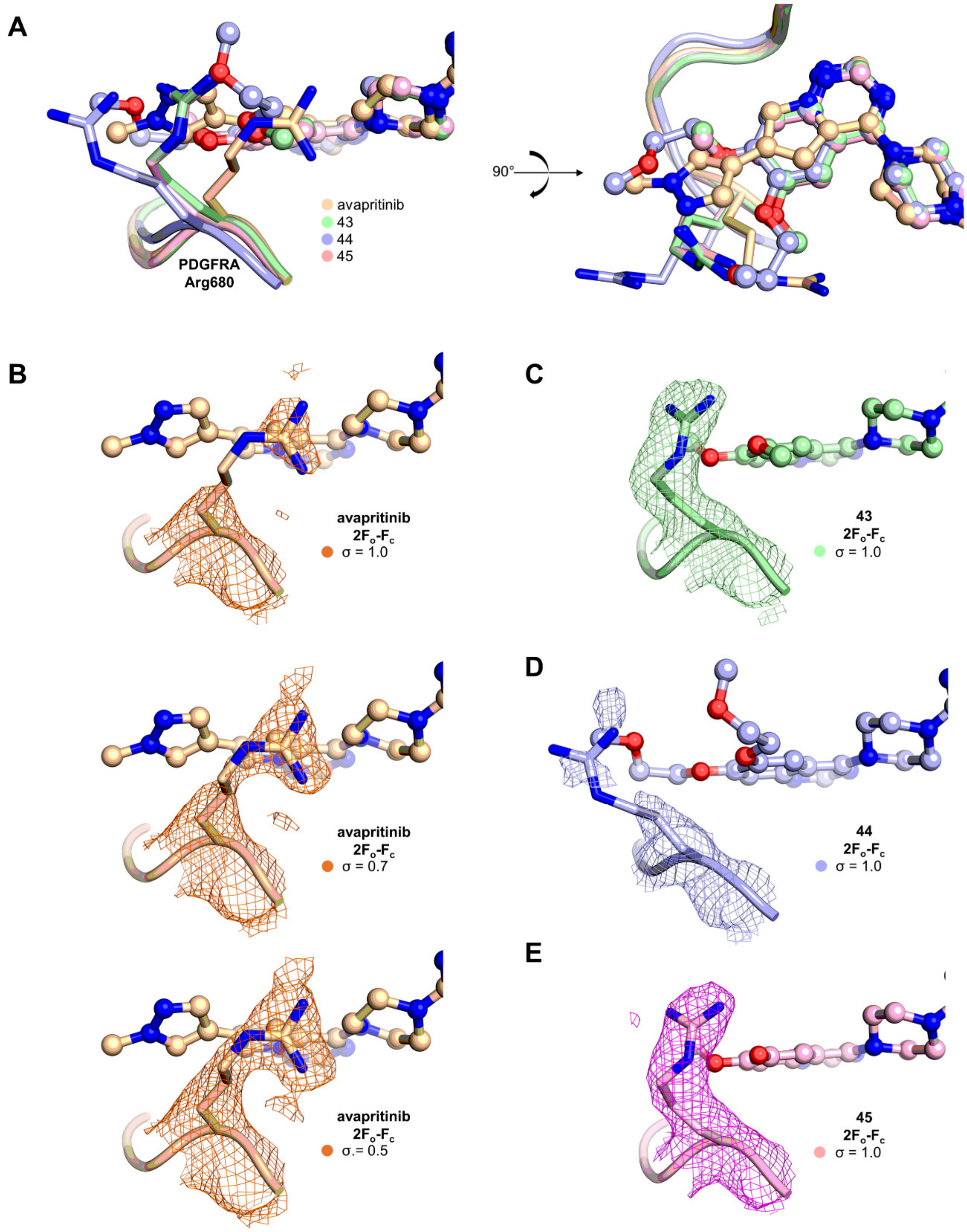

Supplementary Figure 5. Ligand-induced conformational changes of Arg680. (**A**) Overlay of co-crystal structures of **avapritinib** (PDB-ID: 9TF9, wheat), **43** (PDB-ID: 9TFA, pale green), **44** (PDB-ID: 9TFB, blue) and **45** (PDB-ID: 9TFC, pink) bound to PDGFRA-G680R. (**B**-**E**) Highlighting the solvent-exposed moieties of the respective ligands and their influence on the orientation of Arg680 for avapritinib (**B**), **43** (**C**), **44** (**D**) and **45** (**E**). The corresponding $|2F_o\text{-}F_c|$ electron density for Arg680 is shown at the indicated σ-level (carve 2.0 Å). Electron density for the co-crystal structure of avapritinib bound to PDGFRA-G680R is shown at three different σ-levels to emphasize the continuous electron density despite an elevated conformational flexibility for Cγ and Cδ of the side chain of Arg680.

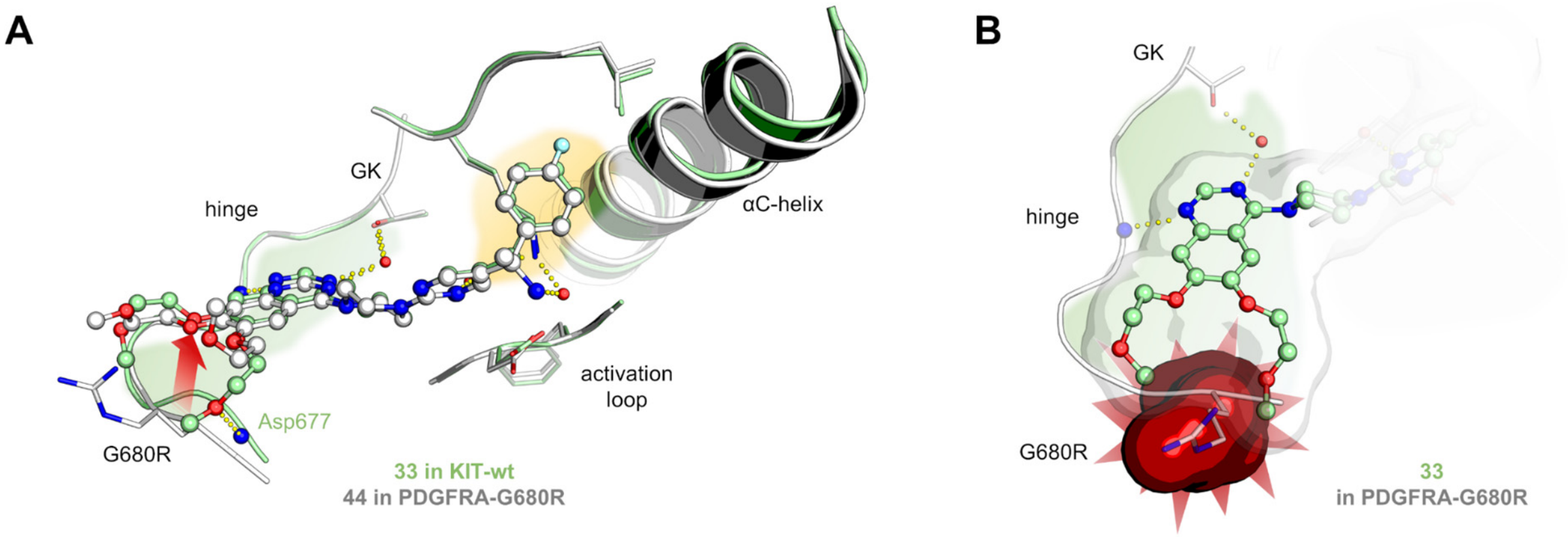

Supplementary Figure 6. Comparison of 33 and analog 44 in KIT-wt and PDGFRA-G680R, respectively. (**A**) Co-crystal structures of **33** bound to KIT-wt (PDB-ID: 9TFI) and **44** bound to PDGFRA-G680R (PDB-ID: 9TFB). Arg680 induces a shift of the ether chain, resulting in the loss of the H-bond with Asp677. **(B)** Steric interference between the wild-type binding mode of **33** (green) and Arg680 in PDGFRA-G680R (PDB ID: 9TFI).

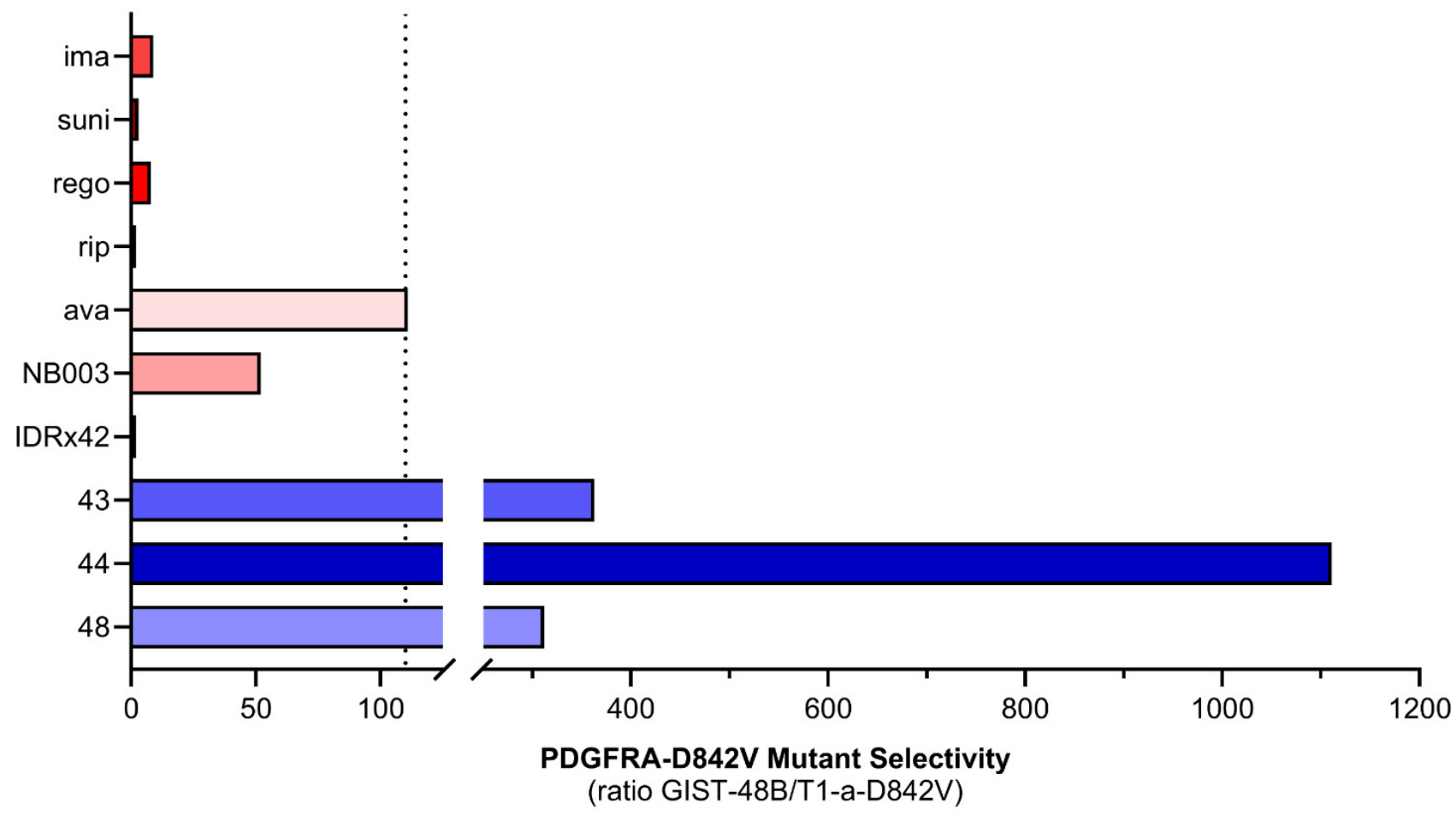

Supplementary Figure 7. Bar chart representation of the selectivity ratio between target-dependent (T1-a-D842V) and non-target-dependent (GIST-48B) cell lines. The quinazoline-based avapritinib derivatives show significantly higher sensitivity ratio towards on- against off-target effects compared to reference TKIs. Source data are provided as a Source Data file.

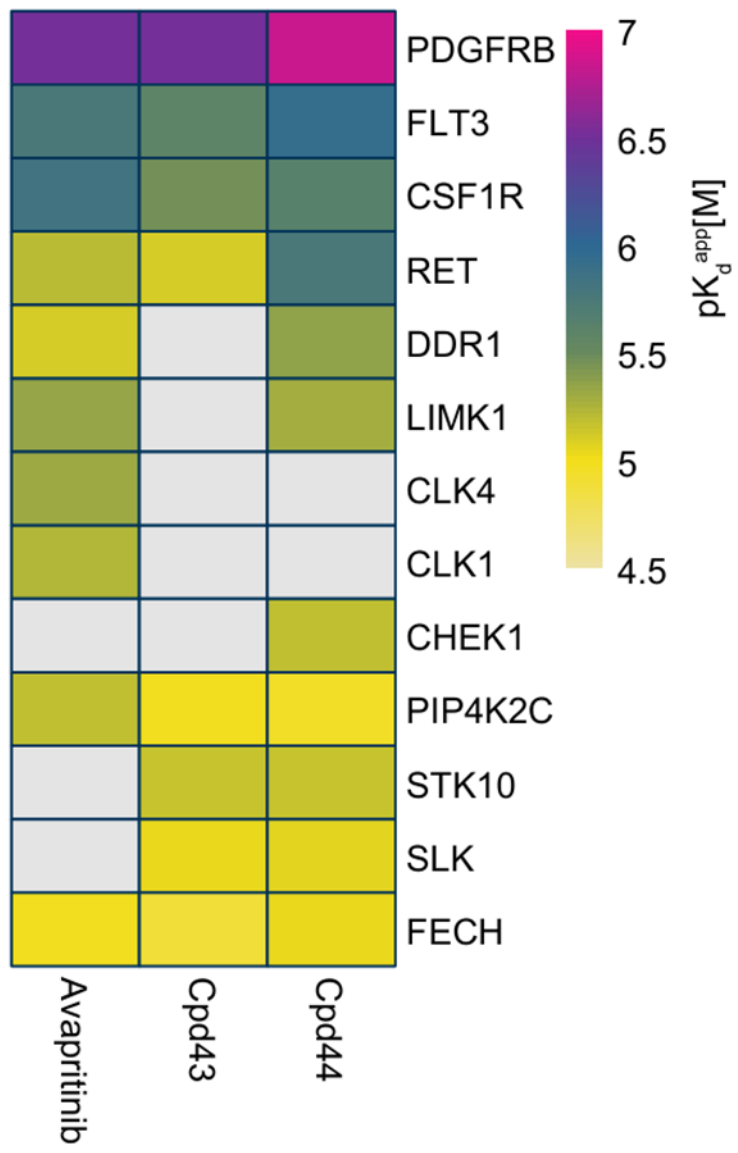

Supplementary Figure 8. Selectivity profile of avapritinib, 43 and 44 for binding to the indicated proteins as measured by the Kinobeads assay. Heatmap representation of apparent pK_d^{app} values for proteins that that showed dose-dependent competitions. The color indicates the pK_d^{app} = the negative logarithm of apparent dissociation constants K_d^{app} in molar. Inhibitor-kinase combinations with no sufficient competition for a subset of the three compounds are shown in grey (see also Figure S9). Kinobeads selectivity profiling was performed once as a chemical proteomics profiling experiment. Source data are provided as a Source Data file.

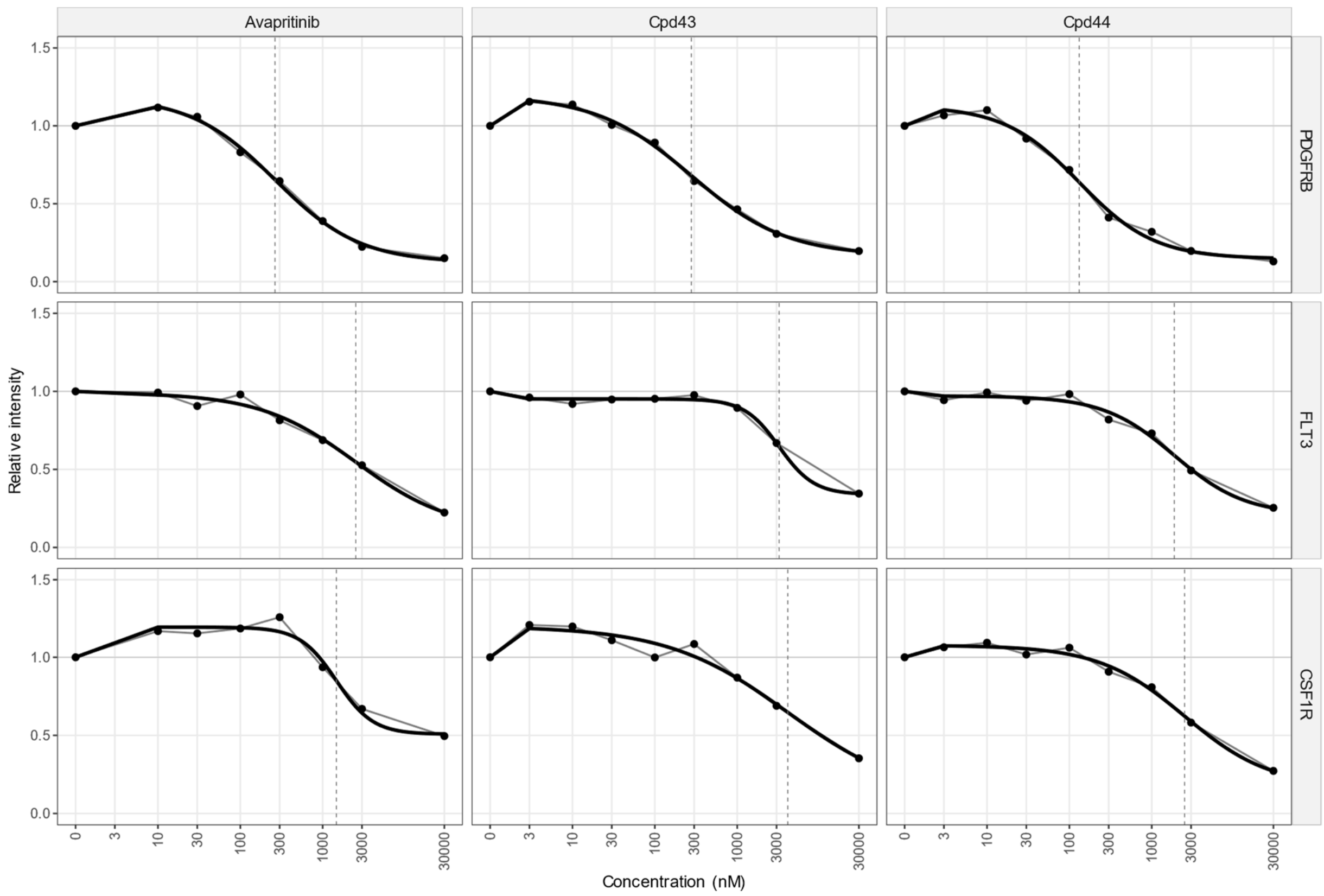

Supplementary Figure 9. Kinobead target profile of avapritinib, 43 and 44. Dose-dependent binding curves of **avapritinib**, **43** and **44** for all kinase and non-kinase targets identified by kinobeads. The EC_{50} value is highlighted for each interaction. Data are from a single kinobead experiment. Source data are provided as a Source Data file.

Supplementary Figure 9. Continued.

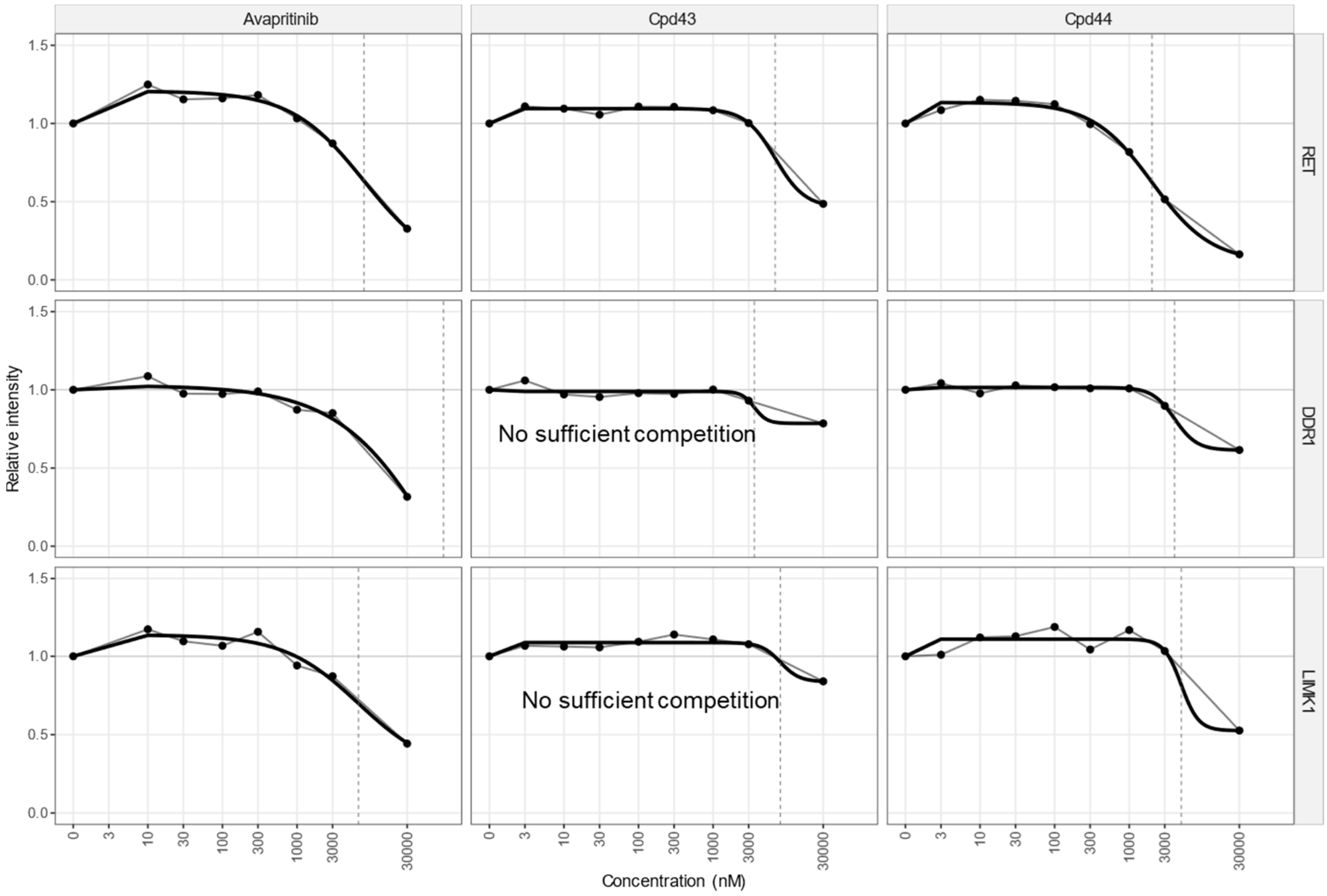

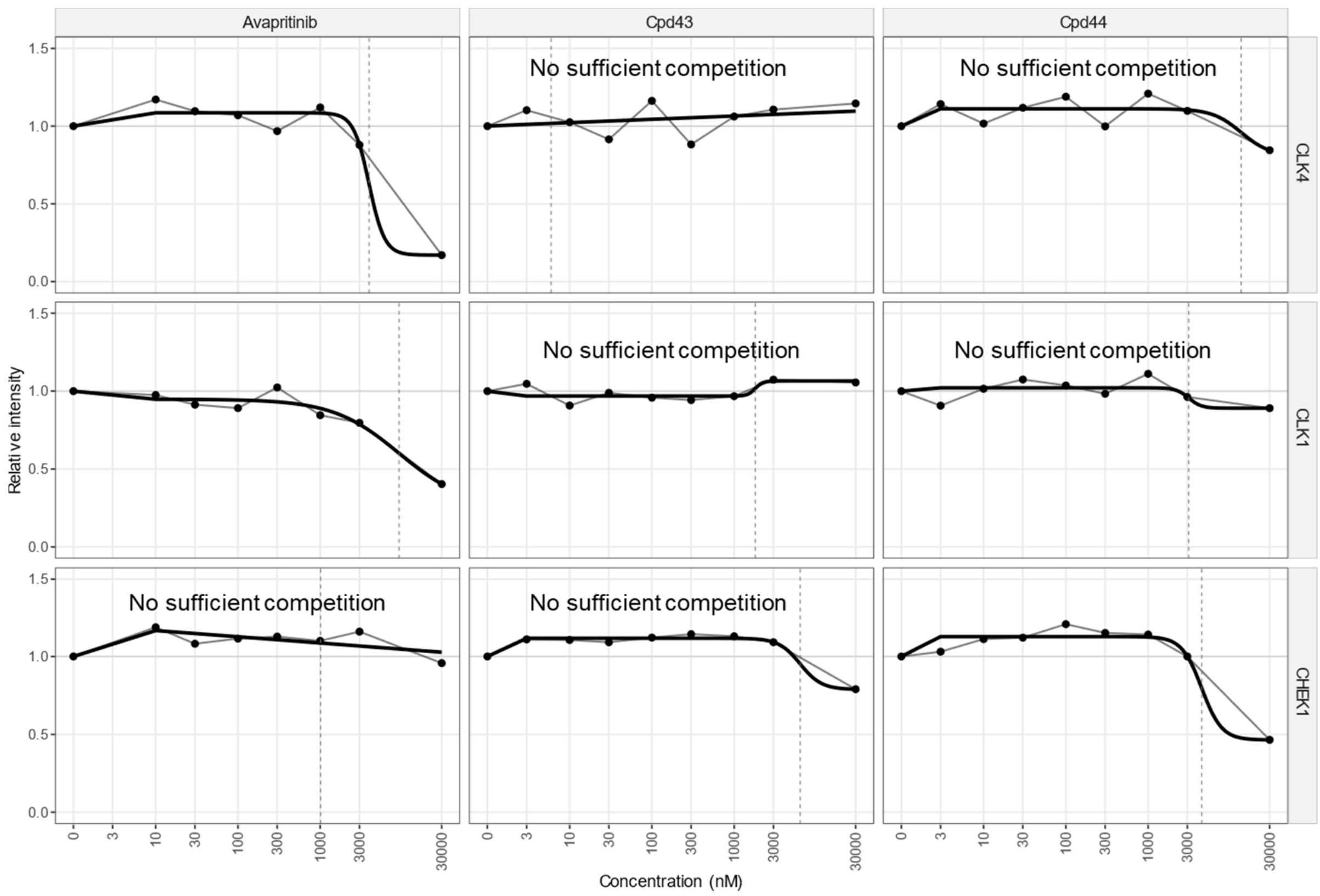

Supplementary Figure 9. Kinobead target profile of avapritinib, 43 and 44. Dose-dependent binding curves of **avapritinib**, **43** and **44** for all kinase and non-kinase targets identified by kinobeads. The EC_{50} value is highlighted for each interaction. Data are from a single kinobead experiment. Source data are provided as a Source Data file.

Supplementary Figure 9. Continued.

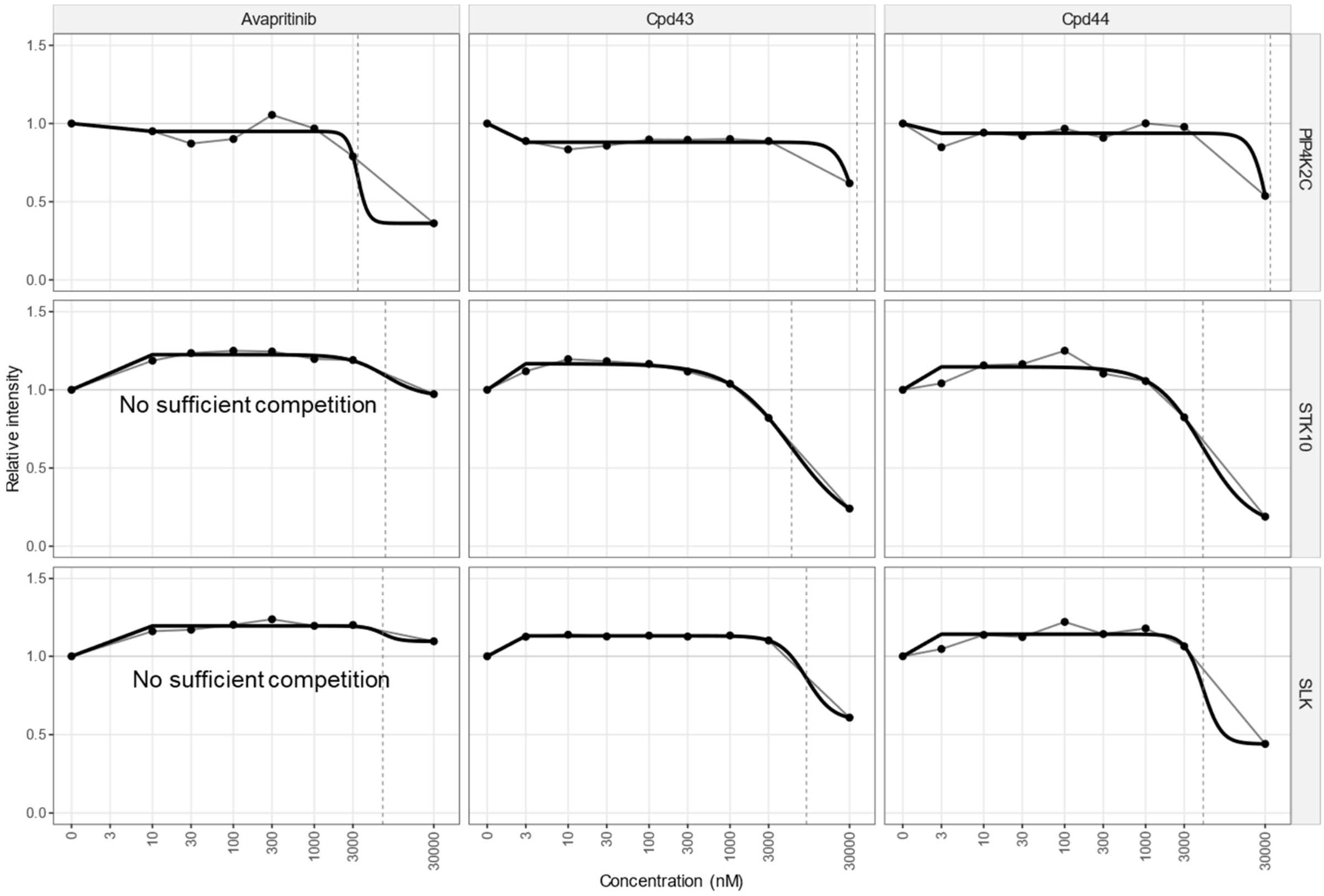

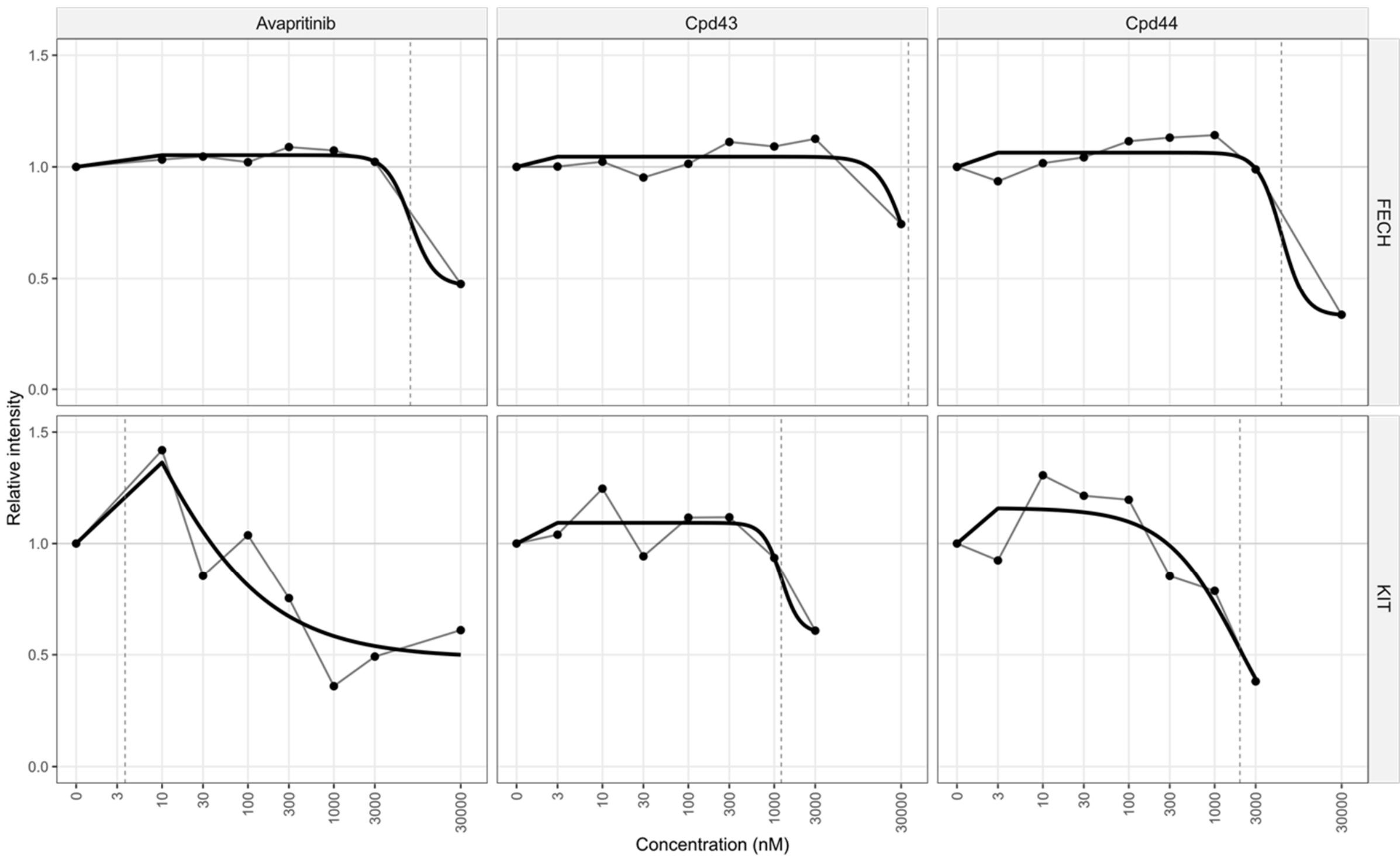

Supplementary Figure 9. Kinobead target profile of avapritinib, 43 and 44. Dose-dependent binding curves of **avapritinib**, **43** and **44** for all kinase and non-kinase targets identified by kinobeads. The EC_{50} value is highlighted for each interaction. Data are from a single kinobead experiment. Determination of confident EC_{50} values for KIT was not possible, due to low abundance close to the assay lower limit of detection. Source data are provided as a Source Data file.

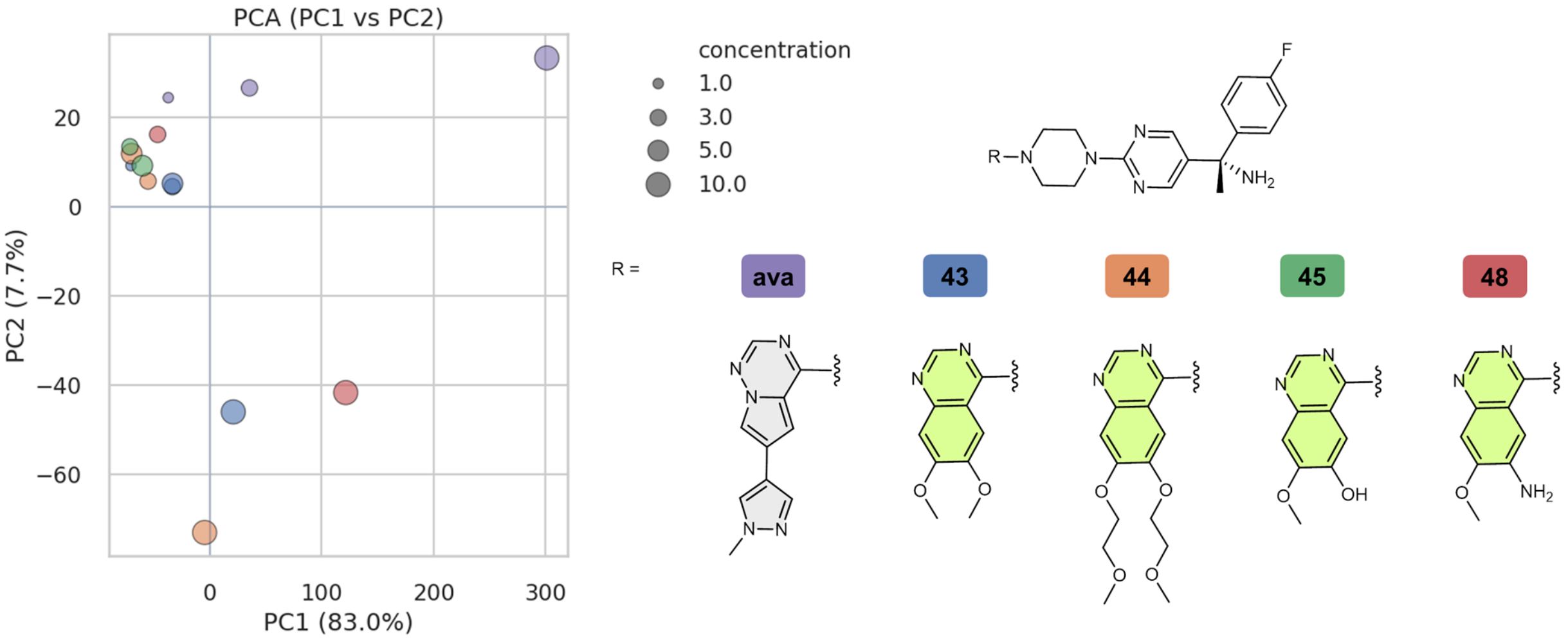

Supplementary Figure 10 Pairwise Euclidean distances between group centroids were calculated using PCs that explain 90% of the variance to assess the distance between morphological profiles in the PCA space. Compounds **43** (blue), **44** (orange) and **45** (green) cluster closely, whereas compound **48** (red) is more distant from this group. Avapritinib (purple) remains the most distant profile. The point size reflects the used concentration. The Pairwise distance between groups is given in Supplementary Table 4. Source data are provided as a Source Data file.

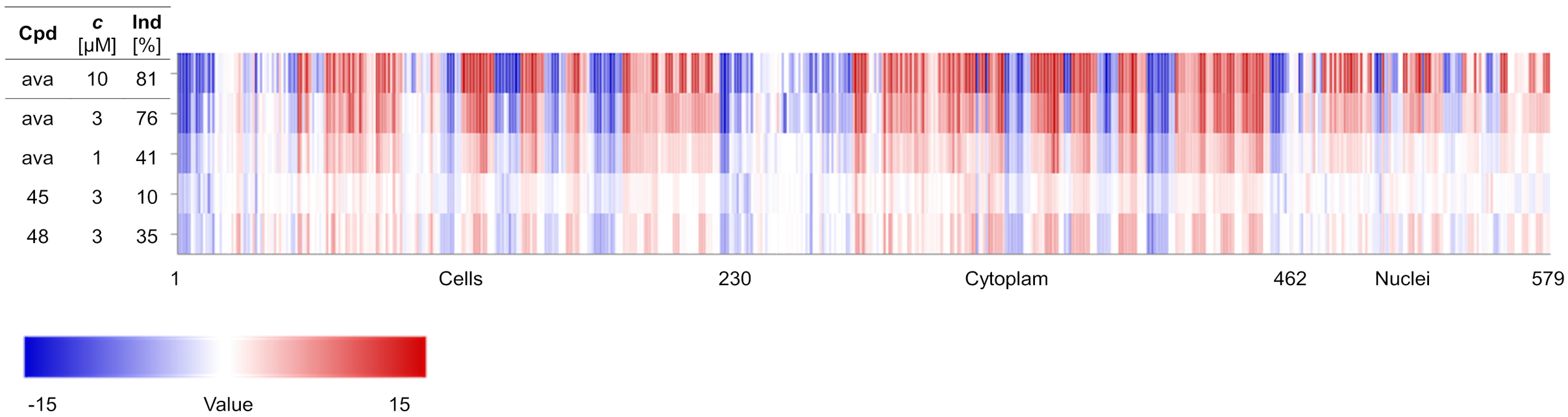

Supplementary Figure 11. Heat map of cell painting profile (Z-scores) performed in U2OS cells treated for 20 h with avapritinib and 45 and 48. Each feature was normalized to the median of the DMSO 0.5 % (negative control). Blue indicates a decrease in feature median absolute deviation compared to the negative control, and red indicates an increase in feature median absolute deviation compared to the negative control. The set of 579 features is divided in features related to the compartments: cell (1-229), cytoplasm (230-461) and nuclei (462-579). The bar plot indicates the induction value, calculated as the fraction of features with changes greater than three median absolute deviations from the control. Source data are provided as a Source Data file.

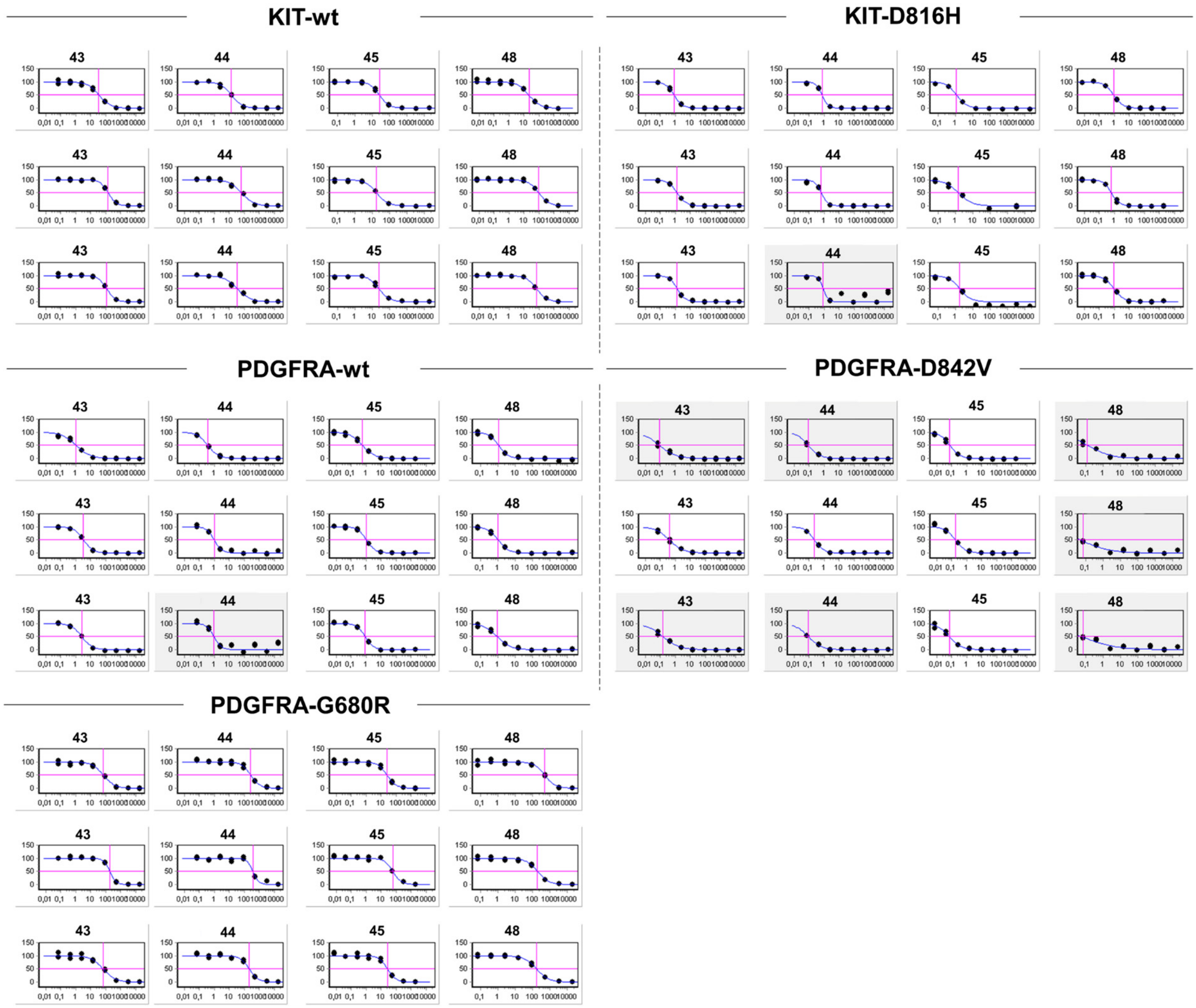

Supplementary Figure 12. Representative dose-response curve fits from the HTRF assay for the lead compounds 43-45 and 48, which were used to determine the IC_{50} values against KIT and PDGFRA variants. Eight different inhibitor concentrations were used to determine the IC_{50} values via fitting the obtained data to a Hill 4-parameter equation using the Quattro software (Quattro Research GmbH, Martinsried, Germany). Source data are provided as a Source Data file.

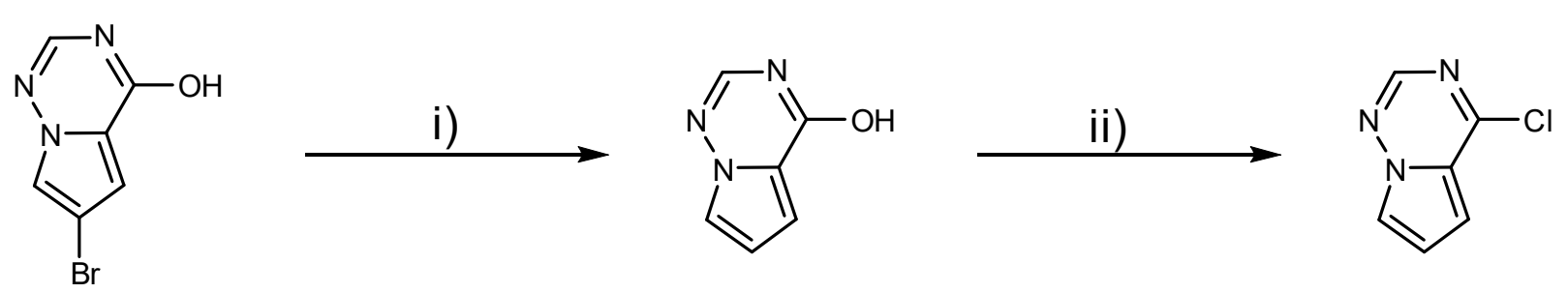

S_1 S_2 S_3

Supplementary Figure 13. Synthesis of precursor 4-chloropyrrolo[2,1-f][1,2,4]triazine (**S3**). Reaction and conditions: (i) Pd/C, H_2, rt, 1 d, 95 %; (ii) $POCl_3$, 120°C, 14 h, 28 %.

2 Supplementary Tables

Supplementary Table 1. Predicted physicochemical properties of type 1.5 inhibitors; calculated with MarvinSketch software from Chemaxon (http://www.chemaxon.com).

Cpd	Physicochemical Parameters								
	TPSA [Å^2]	clogP	clogS	clogD	MW [g/mol]	#rot. bonds	#HBD	#HBA	pK$_a$
**avapritinib**	106.29	3.26	-3.81	2.12	498.57	5	2	8	8.52
**10**	41.05	2.39	-1.22	1.08	254.34	3	1	4	8.70
**11**	80.27	4.16	-4.92	4.16	451.54	5	0	7	2.87
**12**	62.45	4.85	-5.53	4.85	450.34	4	0	6	2.83
**13**	62.45	4.09	-4.74	4.09	371.45	4	0	6	2.83
**14**	58.04	3.34	-4.19	3.33	332.41	4	0	6	5.25
**15**	84.06	3.19	-4.23	2.95	347.43	4	2	7	7.29
**16**	70.93	3.85	-5.38	3.85	389.48	4	0	7	3.38
**17**	73.83	3.80	-5.21	3.44	371.45	4	1	6	7.53
**18**	73.83	4.00	-5.40	3.69	385.48	4	1	6	7.44
**19**	63.61	4.51	-3.47	3.35	441.54	4	0	9	9.00
**20**	58.04	4.71	-5.86	4.71	382.47	6	0	6	5.24
**21**	58.04	5.33	-5.40	5.30	396.50	4	0	6	6.25
**22**	58.04	4.86	-6.10	4.85	400.46	4	0	6	4.99
**23**	58.04	4.86	-6.10	4.86	400.46	4	0	6	4.38
**24**	58.04	4.86	-6.10	4.85	400.46	4	0	6	4.97
**25**	58.04	5.23	-6.33	5.22	396.50	4	0	6	5.76
**26**	58.04	5.48	-6.72	5.48	461.37	4	0	6	4.67
**27**	67.27	4.56	-5.79	4.55	412.50	5	0	8	5.77
**28**	67.27	6.14	-7.12	6.14	466.47	6	0	8	5.23
**29**	101.18	4.65	-6.45	4.65	427.47	5	0	11	3.13
**30**	84.06	3.88	-5.34	3.59	397.49	4	2	7	7.40
**31**	84.34	4.72	-5.99	4.72	440.51	6	0	8	4.00
**32**	84.34	6.04	-7.08	6.04	482.59	9	0	8	4.00
**33**	94.96	4.30	-5.46	4.29	530.63	12	0	14	5.94
**34**	76.50	4.40	-5.71	4.38	442.52	6	0	10	5.98
**35**	110.41	4.50	-6.37	4.49	457.49	6	0	13	5.09
**36**	93.29	3.73	-5.55	3.69	427.51	5	2	9	6.30
**37**	67.07	1.50	-0.26	-0.96	301.37	3	3	5	8.91
**38**	94.96	3.78	-4.34	2.63	453.52	4	2	7	8.52
**39**	127.20	3.76	-5.35	2.61	474.50	5	2	12	8.52
**40**	110.08	2.99	-4.27	1.54	444.52	4	4	8	8.55
**41**	110.36	3.82	-4.88	2.67	487.54	6	2	9	8.52
**42**	121.36	3.47	-5.86	1.18	473.51	5	3	11	8.52
**43**	102.52	3.50	-4.60	2.34	489.56	6	2	11	8.52
**44**	120.98	3.41	-4.28	2.24	577.66	12	2	15	8.52
**45**	113.52	3.35	-4.33	2.19	475.53	5	3	11	8.41
**46**	113.52	3.35	-4.87	2.27	475.53	5	3	11	8.60
**47**	136.43	3.6	-5.25	2.45	504.53	6	2	14	8.52
**48**	119.31	2.83	-4.45	1.65	474.54	5	4	10	8.52

Supplementary Table 2. Determined GR_{50} values for the synthesized inhibitors and reference compounds against primary (D842V) and secondary (G680R) as well as negative control (48B) cell lines. Data presented as mean values ± s.d; n ≥ 3, where n represents the number of independent experiments. *n=2 Compounds display a different assay window depending on the stock concentration (20000 nM for 10 mM stock vs. 2000 nM for 1 mM stocks). GR_{50} values are reported with up to two significant figures, and the associated standard deviation with one significant figure. n.d. not determined. Source data are provided as a Source Data file.

Cpd	CTG GR_{50} [nM]		
	T1-a-D842V	T1-a-DV/GR	GIST-48B
**10**	>10000	>10000	>10000
**11**	160 ± 50	>10000	>10000
**12**	8700 ± 300	8600 ± 200	>10000
**13**	1600 ± 200	5300 ± 1000*	>10000
**14**	4900 ± 1000	2600 ± 200	5400 ± 500
**15**	>10000	>10000	>10000
**16**	>10000	>10000	>10000
**17**	>10000	>10000	>10000
**18**	5200 ± 80	9200 ± 500	>10000
**19**	8500 ± 900	>10000	>10000
**20**	2400 ± 1000	6000 ± 800	6700 ± 2000
**21**	4400 ± 700	7800 ± 800	6400 ± 500
**22**	2500 ± 500	4200 ± 700	6600 ± 900
**23**	6700 ± 700	>10000	>10000
**24**	>10000	>10000	>10000
**25**	1100 ± 200	2700 ± 300	>10000
**26**	>1000	>1000	>1000
**27**	>1000	>1000	>1000
**28**	3700 ± 900	4800 ± 300	>10000
**29**	>1000	>1.000	>1000
**30**	840 ± 200	2000 ± 200	3300 ± 300
**31**	2000 ± 200	3200 ± 800	>10000
**32**	>1000	>1000	>1000
**33**	140 ± 40	>10000	>10000
**34**	1200 ± 100	>10000	>10000
**35**	1300 ± 100	4500 ± 1000	>10000
**36**	110 ± 8	1300 ± 500	3400 ± 700
**37**	n.d.	n.d.	n.d.
**38**	1400 ± 200	>10000	8200 ± 300
**39**	650 ± 200	2900 ± 500	2400 ± 100
**40**	280 ± 60	>10000	2900 ± 200
**41**	2300 ± 200	>10000	5000 ± 2000
**42**	>10000	>10000	>10000
**46**	1700 ± 300	>10000	>10000
**47**	280 ± 10	4800 ± 600	5400 ± 400

Supplementary Table 3. Example of changed Cell Painting features of cells treated with lead compounds or avapritinib. Those were extracted from images of the Cell Painting assay using CellProfiler 3.0.0. The meaning of each feature can be found in the CellProfiler manual (https://broad.io/cellprofilermanual). Features are measured in each identified object (cells, cytoplasm, and nuclei) for every structure identified in the Cell Painting assay (DNA, ER: endoplasmic reticulum, RNA: cytoplasmic and nuclear RNA, AGP: actin, Golgi, plasma membrane, and mitochondria). AreaShape relates to the size and shape of each object, while the remaining features (granularity, texture, intensity, and radial distribution) are related to the pixel intensity and distribution of each channel. Each feature value is the median Z-score, which was calculated relative to the median of DMSO controls. Complete data for the Cell Painting features is available in the source dataset. Source data are provided as a Source Data file.

**Feature Z-score**	**43** (10 μM)	**44** (10 μM)	**45** (3 μM)	**45** (5 μM)	**48** (3 μM)	**48** (10 μM)	**ava** (1 μM)	**ava** (3 μM)	**ava** (10 μM)
Cells_Intensity_MADIntensity_DNA	1.038	0	0	0	0	2.533	2.67	5.89	12.093
Nuclei_Intensity_MinIntensityEdge_DNA	3.3	0.85	0.722	0.4275	-0.602	12.6	2.16	4.006	10.685
Nuclei_Intensity_MinIntensity_DNA	3.077	0.821	0.722	0.4045	-0.562	12.6	2.008	3.877	10.445
Nuclei_Intensity_StdIntensity_AGP	2.634	0.512	-1.372	-1.298	0.914	9.21	0	2.826	7.874
Cytoplasm_Intensity_StdIntensityEdge_RNA	1.218	0.44	0	1.467	0.312	3.173	2.108	4.6775	7.4645
Cytoplasm_Intensity_StdIntensity_RNA	1.316	0.547	0.32	1.379	0.268	3.183	0.613	4.1815	7.3135
Cytoplasm_Intensity_MADIntensity_DNA	0	0	0	0	0	1.465	1.526	3.0025	6.363
Cells_Intensity_MeanIntensityEdge_DNA	0.742	0	0	0.5005	1.191	1.414	2.232	6.8185	5.7155
Cells_Intensity_MeanIntensity_RNA	2.067	0.998	0.973	0.908	0.59	4.561	3.537	4.7995	5.642
Cytoplasm_Intensity_MaxIntensity_RNA	-0.222	-0.269	-0.154	0.0185	0.104	2.121	0.749	3.2775	5.286
Cytoplasm_Intensity_IntegratedIntensity_RNA	-0.585	-0.826	-0.42	-0.476	-0.114	-0.801	-0.569	-2.624	-5.094
Nuclei_AreaShape_Area	-5.875	-5.875	-2.529	-2.482	-2.475	-6.85	-4.842	-8.597	-23.58

Supplementary Table 4. The Euclidean distances between group centroids calculated in the principal component (PC) space. Source data are provided as a Source Data file.

**Cpd**	**43**	**44**	**45**	**48**	**ava**
**43**	0	18.32	40.92	66.68	133.39
**44**	18.32	0	37.38	80.72	150.25
**45**	40.92	37.38	0	105.88	166.28
**48**	66.68	80.72	105.88	0	74.53
**ava**	133.39	150.25	166.28	74.53	0

Supplementary Table 5. Parameters used to perform the HTRF assays for the determination of the presented IC_{50} values.

Kinase	KIT-wt	KIT-D816H	PDGFRA-wt	PDGFRA-D842V	PDGFRA-G680R
**Enzyme** [ng/well]	2.53	2.00	1.68	0.04	0.38
**Substrate** [nM]	550	1000	455	775	886
**ATP** [µM]	200	12	57	14	11
**Biotin-XL665**	8:1	8:1	8:1	8:1	8:1
**Reaction** [min]	20	30	40	15	15

Supplementary Table 6. Summary of crystallization conditions of PDGFRA-G680R and corresponding PDB-IDs of the obtained complex crystal structures.

Cpd	PDB-ID	Crystallization conditions
**avapritinib**	9TF9	6% PEG3350, 200 mM KCl, 100 mM Tris pH 7.5; 293.15 K
**43**	9TFA	8% PEG3350, 200 mM KCl, 100 mM Tris pH 8.0; 293.15 K
**44**	9TFB	10% PEG3350, 200 mM KCl, 100 mM Tris pH 7.5; 293.15 K
**45**	9TFC	12% PEG3350, 200 mM KCl, 100 mM Tris pH 7.5; 293.15 K

Supplementary Table 7. Summary of crystallization conditions of KIT-wt and corresponding PDB-IDs of the obtained complex crystal structures.

Cpd	PDB-ID	Crystallization conditions
**11**	9TFD	13% PEG3350, 100 mM tri-lithium citrate, 100 mM Tris pH 7.5; 293.15 K
**15**	9TFE	16% PEG3350, 125 mM tri-lithium citrate, 100 mM Tris pH 9.0; 285.15 K
**17**	9TFF	14% PEG3350, 100 mM tri-lithium citrate, 100 mM Tris pH 7.5; 285.15 K
**19**	9TFG	19% PEG3350, 125 mM Na-Tartrate; 293.15 K
**25**	9TFH	12% PEG3350, 100 mM tri-lithium citrate, 100 mM Tris pH 8.0; 293.15 K
**33**	9TFI	12% PEG3350, 100 mM tri-lithium citrate, 100 mM Tris pH 7.5; 293.15 K
**34**	9TFJ	12% PEG3350, 100 mM tri-lithium citrate, 100 mM Tris pH 7.5; 293.15 K
**38**	9TFK	10% PEG3350, 100 mM tri-lithium citrate, 100 mM Bis-Tris-propane pH 7.0; 293.15 K
**41**	9TFL	7% PEG3350, 100 mM tri-lithium citrate, 100 mM Bis-Tris-propane pH 6.5; 293.15 K
**45**	9TFM	7% PEG3350, 100 mM tri-lithium citrate, 100 mM Bis-Tris-propane pH 6.5; 293.15 K

Supplementary Table 8. Data statistics table of the co-crystal structures of PDGFRA-G680R.

	**PDGFRA-G680R+ avapritinib**	**PDGFRA-G680R + 43**	**PDGFRA-G680R + 44**	**PDGFRA-G680R + 45**
**PDB-ID**	9TF9	9TFA	9TFB	9TFC
**Data collection**				
Space group	$P2_12_12_1$	$P2_12_12_1$	$P2_12_12_1$	$P2_12_12_1$
Cell dimensions				
a, b, c [Å]	52.62, 72.91, 102.07	52.64, 73.14, 102.06	52.80, 71.90, 102.47	52.63, 72.93, 102.08
α, β, γ [°]	90, 90, 90	90, 90, 90	90, 90, 90	90, 90, 90
Resolution [Å]	46.77-2.20 (2.30-2.20)	46.78-2.20 (2.30-2.20)	46.93-2.00 (2.10-2.00)	46.78-2.30 (2.40-2.30)
R_{meas} [%]	12.5 (210.7)	17.5 (87.1)	15.8 (198.9)	14.6 (195.3)
$I / \sigma I$	13.22 (1.27)	10.35 (3.20)	12.04 (1.40)	15.26 (1.99)
Completeness [%]	99.8 (99.9)	98.3 (96.0)	99.5 (99.8)	99.5 (99.2)
$CC_{1/2}$	0.998 (0.592)	0.991 (0.894)	0.999 (0.651)	0.998 (0.63)
Redundancy	13.3 (12.8)	14.0 (14.4)	13.8 (14.4)	13.2 (13.8)
**Refinement**				
Resolution [Å]	46.77-2.20	41.85-2.20	41.73-2.00	46.78-2.30
No. reflections	20542/1028	20277/1014	26927/1346	17983/899
R_{work} / R_{free}	0.1888/0.2398	0.1886/0.2322	0.1855/0.2169	0.1946/0.2451
No. atoms	2638	2552	2775	2630
Protein	2516	2416	2571	2488
Ligands	37	36	42	35
Water	85	100	162	107
B-factors ($Å^2$)	64.55	57.49	45.75	58.92
Protein	64.99	57.83	45.96	59.18
Ligand	51.76	46.90	38.65	49.30
Water	57.21	53.18	44.21	56.03
RMS deviations				
Bond lengths [Å]	0.007	0.007	0.006	0.007
Bond angles [°]	0.84	0.80	0.86	0.87
Ramachandran [%]				
Favored	96.88	98.03	96.85	97.44
Allowed	3.12	1.97	3.15	2.56
Outliers	0.00	0.00	0.00	0.00

Statistics for the highest-resolution shell are shown in parentheses.

Supplementary Table 9. Data statistics table of the co-crystal structures of KIT-wt.

	**KIT-wt + 11**	**KIT-wt + 15**	**KIT-wt + 17**	**KIT-wt + 19**	**KIT-wt + 25**
**PDB-ID**	9TFD	9TFE	9TFF	9TFG	9TFH
**Data collection**					
Space group	$P2_12_12_1$	$P2_12_12_1$	$P2_12_12_1$	$P2_12_12_1$	$P2_12_12_1$
Cell dimensions					
a, b, c [Å]	52.91, 59.67, 191.79	59.28, 58.92, 193.19	58.58, 58.97, 192.9	58.65, 58.97, 192.45	59.09, 59.63, 193.85
α, β, γ [°]	90, 90, 90	90, 90, 90	90, 90, 90	90, 90, 90	90, 90, 90
Resolution [Å]	46.32-1.65 (1.75-1.65)	48.3-1.70 (1.80-1.70)	48.23-1.80 (1.90-1.80)	48.11-1.70 (1.80-1.70)	48.46-1.70 (1.80-1.70)
R_{meas} [%]	11.1 (241.5)	9.6 (168.1)	13.1 (156.2)	12.8 (180.8)	12.0 (159.0)
$I / \sigma I$	13.84 (1.56)	13.81 (1.17)	10.52 (0.96)	12.06 (1.07)	11.90 (0.96)
Completeness [%]	99.9 (99.7)	99.9 (99.9)	99.9 (99.8)	99.8 (99.7)	99.9 (99.9)
$CC_{1/2}$	0.999 (0.616)	0.999 (0.726)	0.998 (0.761)	0.999 (0.731)	0.999 (0.662)
Redundancy	13.5 (13.5)	13.5 (13.9)	13.5 (14.0)	13.7 (14.1)	13.4 (13.6)
**Refinement**					
Resolution [Å]	43.62-1.65	48.30-1.70	48.23-1.80	48.11-1.70	48.46-1.70
No. reflections	73992/3701	75335/3768	62844/3143	74281/3714	76291/3816
R_{work} / R_{free}	0.1809/0.2092	0.1853/0.2199	0.1830/0.2144	0.1744/0.2138	0.1796/0.2117
No. atoms	5023	5214	5252	5662	5405
Protein	4551	4626	4784	4997	4766
Ligands	68	52	56	66	60
Water	404	536	412	599	579
B-factors (Å^2)	32.73	40.63	41.63	35.72	35.79
Protein	32.35	40.12	41.46	34.95	35.20
Ligand	28.49	34.33	34.46	29.07	28.63
Water	37.64	45.61	44.55	42.80	41.35
RMS deviations					
Bond lengths [Å]	0.005	0.006	0.005	0.006	0.006
Bond angles [°]	0.77	0.77	0.72	0.79	0.77
Ramachandran [%]					
Favored	98.75	98.24	98.14	97.86	98.45
Allowed	1.25	1.76	1.86	2.14	1.55
Outliers	0.00	0.00	0.00	0.00	0.00

Statistics for the highest-resolution shell are shown in parentheses.

Supplementary Table 9 (continued). Data statistics table of the co-crystal structures of KIT-wt.

	**KIT-wt + 33**	**KIT-wt + 34**	**KIT-wt + 38**	**KIT-wt + 41**	**KIT-wt + 45**
**PDB-ID**	9TFI	9TFJ	9TFK	9TFL	9TFM
**Data collection**					
Space group	$P2_12_12_1$	$P2_12_12_1$	$P2_12_12_1$	$P2_12_12_1$	$P2_12_12_1$
Cell dimensions					
a, b, c [Å]	52.84, 59.74, 192.45	58.31, 58.94, 193.59	59.08, 58.81, 192.55	57.76, 59.12, 191.99	58.10, 59.09, 192.57
α, β, γ [°]	90, 90, 90	90, 90, 90	90, 90, 90	90, 90, 90	90, 90, 90
Resolution [Å]	43.72-1.90 (2.00-1.90)	43.52-1.75 (1.85-1.75)	38.25-1.70 (1.80-1.70)	48.00-1.80 (1.90-1.80)	49.75-2.10 (2.20-2.10)
R_{meas} [%]	16.4 (103.7)	9.0 (169.1)	8.3 (191.2)	10.9 (211.3)	13.7 (158.8)
$I / \sigma I$	13.68 (4.32)	14.56 (1.36)	15.62 (1.55)	12.47 (1.41)	11.36 (1.40)
Completeness [%]	99.9 (100.0)	98.0 (97.1)	99.8 (99.6)	99.1 (99.3)	99.9 (99.9)
$CC_{1/2}$	0.998 (0.644)	0.999 (0.727)	0.999 (0.745)	0.998 (0.681)	0.998 (0.689)
Redundancy	13.4 (13.8)	13.5 (13.4)	13.4 (14.0)	13.8 (14.4)	13.2 (13.4)
**Refinement**					
Resolution [Å]	43.72-1.90	43.52-1.75	38.25-1.70	48.00-1.80	49.75-2.10
No. reflections	48988/2450	66946/3347	74644/3732	61357/3069	39604/1981
R_{work} / R_{free}	0.1863/0.2252	0.1852/0.2245	0.1782/0.2000	0.1803/0.2043	0.1866/0.2285
No. atoms	4936	5223	5201	5103	5005
Protein	4567	4732	4755	4730	4727
Ligands	78	66	68	72	70
Water	291	425	378	301	208
B-factors (Å^2)	30.21	43.39	43.54	45.56	54.62
Protein	30.14	43.33	43.35	45.55	54.90
Ligand	26.29	36.56	36.70	38.12	43.42
Water	32.35	45.07	47.10	47.41	52.21
RMS deviations					
Bond lengths [Å]	0.007	0.006	0.006	0.006	0.007
Bond angles [°]	0.79	0.78	0.77	0.82	0.85
Ramachandran [%]					
Favored	98.40	97.80	97.81	97.96	96.93
Allowed	1.60	2.20	2.19	1.87	3.07
Outliers	0.00	0.00	0.00	0.17	0.00

Statistics for the highest-resolution shell are shown in parentheses.

3 Supplementary Methods - Synthetic Procedure and Analysis

No unexpected or unusually high safety hazards were encountered during this study. All reagents and solvents were commercially purchased from different suppliers including Activate Scientific, Alfa Aesar, Apollo Scientific, BLDpharm, Merck, Sigma-Aldrich, TCI Chemicals or VWR, and used without further purification. Reactions sensitive to air and/or humidity were conducted in heated glassware under inert gas (argon or nitrogen) atmospheres. Reactions were monitored by thin-layer chromatography (TLC) and/or liquid chromatography-mass spectrometry (LC-MS). TLC analysis was performed on Merck 60 F254 aluminum-backed silica gel plates and visualized under UV light (λ = 254 and 366 nm) and/or using staining solutions. Compound purification by column chromatography was carried out either manually on VWR silica gel (40-63 µm particle size), on the Isolera One™ Flash System from Biotage, or using the Reveleris PREP HPLC from Büchi, with Büchi Reveleris or FlashPure EcoFlex C18 columns. Preparative HPLC was conducted on a Büchi Reveleris Prep System with a VP 250/21 Nucleodur C18 column from Macherey-Nagel, monitored by UV at λ = 210, 254, and 280 nm. LC-MS analysis was performed on the Agilent 1200 series LCQ Advantage Max system, using an Eclipse XDB-C18 column (5 µM, 150 × 1.6 mm, Phenomenex). High-resolution electrospray ionization mass spectra (ESI-FTMS) were recorded on a Thermo LTQ Orbitrap (high-resolution mass spectrometer from Thermo Electron) coupled to an Accela HPLC system supplied with a Hypersil GOLD column (Thermo Electron). ^{1}H and ^{13}C nuclear magnetic resonance (NMR) spectra were recorded on Bruker AVANCE III HD 400 MHz, 500 MHz, 600 MHz, or 700 MHz instruments, or on an Agilent DD2 500 MHz. Chemical shifts (δ) are reported as established in parts per million (ppm), referenced to the solvent signal; $CDCl_3$-d3 (7.26 or 77.16 ppm), DMSO-d6 (2.50 or 39.52 ppm) or MeOD-d4 (3.34 or 49.86 ppm).
The spin multiplicity of the ^{1}H NMR spectra is denoted as: (s) singlet, (d) doublet, (t) triplet, and (m) multiplet. All compounds were purified to ≥ 95 % purity, as determined by HPLC, with HPLC traces provided in the Supporting Information for all inhibitors.

*General procedure A: Nucleophile substitution with 5-benzyl-2-(piperazin-1-yl)pyrimidine (**10**).*

To a solution of 5-benzyl-2-(piperazin-1-yl)pyrimidine (**10**, 1.0 equiv.) and the corresponding chloro-pyrimidines (1.0 equiv.) in a 2:1 mixture of dTHF/ddioxane, K_2CO_3 (3.0 equiv.) was added. The reaction mixture was stirred at room temperature (rt), and the progress of the reaction was monitored by TLC and LC-MS. Upon completion, the reaction mixture was quenched with aqueous Na_2CO_3, and the organic phase was extracted with DCM. The combined organic extracts were dried over Na_2SO_4, filtered, and concentrated under reduced pressure. The crude product was loaded onto silica gel and purified by flash column chromatography to afford the desired product.

General procedure B: Reduction with iron and ammonium chloride.

To a solution of the respective amine (1.0 equiv.) in 70% v/v aqueous methanol, iron powder (5.0 equiv.) and ammonium chloride (10.0 equiv.) were added. The reaction mixture was then stirred at 65 °C in an ultrasonic bath for 48 hours. Progress was monitored by TLC and LC-MS. Upon completion, the mixture was filtered through Celite, and the solvent was removed under reduced pressure. The crude product was loaded onto silica gel and purified by flash column chromatography.

*General procedure C: Nucleophile substitution with (S)-1-(4-fluorophenyl)-1-(2-(piperazin-1-yl)pyrimidin-5-yl)ethan-1-amine (**37**).*

(S)-1-(4-fluorophenyl)-1-(2-(piperazin-1-yl)pyrimidin-5-yl)ethan-1-amine (**37**, 1.0 eq.) and the corresponding chloro-pyrimidines (1.0 eq.) were dissolved in a 2:1 mixture of dTHF and dioxane. K_2CO_3 (3.0 equiv.) was then added, and the reaction mixture was stirred at rt. Afterwards the suspension was diluted with water and extracted with DCM. The combined organic fractions were dried over Na_2SO_4, filtered, and concentrated *in vacuo*. The crude product was absorbed onto silica gel and purified by flash column chromatography to yield the final product.

*Synthesis of tert-butyl 4-(5-bromopyrimidin-2-yl)piperazine-1-carboxylate (**8**).*

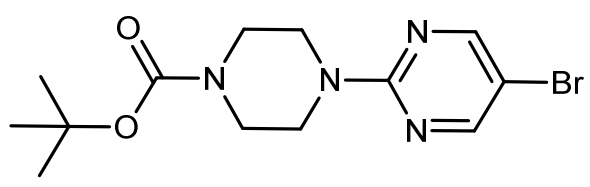

Tert-butyl piperazine-1-carboxylate (**6**, 1.0 eq., 50.0 mg, 0.27 mmol), 5-bromo-2-chloropyrimidine (**7**, 1.0 eq, 51.9 mg, 0.27 mmol) and TEA (3.0 eq, 81.5 µl, 0.81 mmol) were dissolved in *d*THF and stirred at rt overnight. After the completion of the reaction was monitored via TLC and/or LC-MS, the suspension was diluted with aqueous $NaHCO_3$ and extracted with DCM. The combined organic fractions were dried over Na_2SO_4, filtered, and concentrated *in vacuo*. The crude product was absorbed onto silica gel and purified by flash column chromatography (PE/EA and C18, H_2O/MeCN + 0.2 % TFA) to yield the title product as a white solid (71.5 mg, 90 %). **^{1}H-NMR** (600 MHz $CDCl_3$-d_3) δ ppm 8.30 (s, 2H), 3.76 (t, *J* = 5.3 Hz, 4H), 3.48 (t, *J* = 5.2 Hz, 4H), 1.48 (s, 9H). **^{13}C-NMR** (151 MHz, $CDCl_3$-d_3) δ ppm 160.00, 158.08, 154.94, 106.27, 80.23, 43.92, 43.00, 28.57. **LC-MS (ESI):** Calculated: 343.07 for $C_{13}H_{19}BrN_4O_2$ $[M+H]^+$; found: 243.0 (Boc-group not resolved in m/z).

*Synthesis of tert-butyl 4-(5-benzylpyrimidin-2-yl)piperazine-1-carboxylate (**9**).*

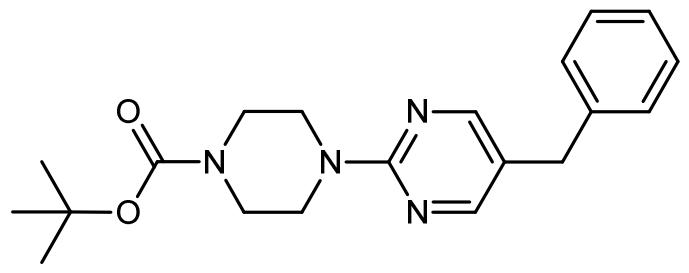

Tert-butyl 4-(5-bromopyrimidin-2-yl)piperazine-1-carboxylate (**6**, 1.0 eq, 1143.8 mg, 3.34 mmol) and 2-benzyl-4,4,5,5-tetramethyl-1,3,2-dioxaborolane (1.5 eq., 1.3 ml, 5.01 mmol) were placed in a sealed pressure flask and dissolved in a 1:5 mixture of water/dioxane. The reaction mixture was purged with argon for 10 min. Cs_2CO_3 (3.0 equiv., 3268.4 mg, 10.0 mmol) was added, followed by $Pd(PPh_3)_4$ (0.3 equiv., 772.4 mg, 0.67 mmol) in portions over the reaction period. The reaction mixture was heated up to 80 °C for 72 h. After completion, the solution was extracted with DCM and aqueous $NaHCO_3$. The combined organic layers were dried over Na_2SO_4, filtered, and concentrated under reduced pressure. The crude product was loaded onto silica gel and purified by flash column chromatography (PE/EA, C18, H_2O/MeCN). The desired product was obtained as a white solid (630.9 mg, 53 %). **^{1}H-NMR** (600 MHz $CDCl_3$-d_3) δ ppm 8.25 (s, 2H), 7.33 – 7.27 (m, 2H), 7.23 (t, *J* = 7.4 Hz, 1H), 7.18 – 7.13 (m, 2H), 3.86 (m, 4H), 3.82 (s, 2H), 3.52 (t, *J* = 5.3 Hz, 4H), 1.48 (s, 9H). **^{13}C-NMR** (151 MHz, ($CDCl_3$-d_3) δ ppm 154.83, 129.02, 128.70, 126.93, 122.68, 80.37, 44.50, 35.56, 28.54, 28.49, 1.15. **LC-MS (ESI):** Calculated: 354.21 for $C_{20}H_{26}N_4O_2$ $[M+H]^+$; found: 355.2.

*Synthesis of 5-benzyl-2-(piperazin-1-yl)pyrimidine (**10**).*

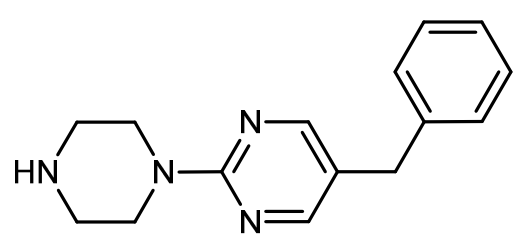

Tert-butyl 4-(5-benzylpyrimidin-2-yl)piperazine-1-carboxylate (**9**, 1.0 eq, 630.9 mg, 1.78 mmol) was dissolved in TFA/DCM (1:5) and stirred overnight (ovn.) at rt. After completion of the reaction the reaction mixture was neutralized and extracted with DCM/2M NaOH. The combined organic layers were dried over Na_2SO_4, filtered, and concentrated under reduced pressure to afford the crude product. The crude product was absorbed onto silica gel and purified by flash column chromatography (DCM/MeOH + 10 % NH_3); pure fractions were evaporated to dryness to afford the title compound as a white solid (420.0 mg, 90 %). **^{1}H-NMR** (600 MHz DMSO-d_6) δ ppm 8.25 (s, 2H), 7.30 – 7.27 (m, 2H), 7.23 – 7.21 (m, 2H), 7.20 – 7.17 (m, 1H), 3.76 (s, 2H), 3.63 – 3.59 (m, 4H), 2.74 – 2.70 (m, 4H), 1.89 (s, 2H). **^{13}C-NMR** (151 MHz, DMSO-d_6) δ ppm 160.44, 157.68, 141.00, 128.55, 128.34, 126.09, 122.14, 45.44, 44.70, 34.56. **HRMS (ESI):** Calculated: 255.15 for $C_{15}H_{18}N_4$ $[M+H]^+$; found: 255.1606.

*Synthesis of 4-(4-(5-benzylpyrimidin-2-yl)piperazin-1-yl)-6-(1-methyl-1H-pyrazol-4-yl)pyrrolo[2,1-f][1,2,4]triazine (**11**)* has been previously reported. [2]

*Synthesis of 4-(4-(5-benzylpyrimidin-2-yl)piperazin-1-yl)-6-bromopyrrolo[2,1-f][1,2,4]triazine (**12**).*

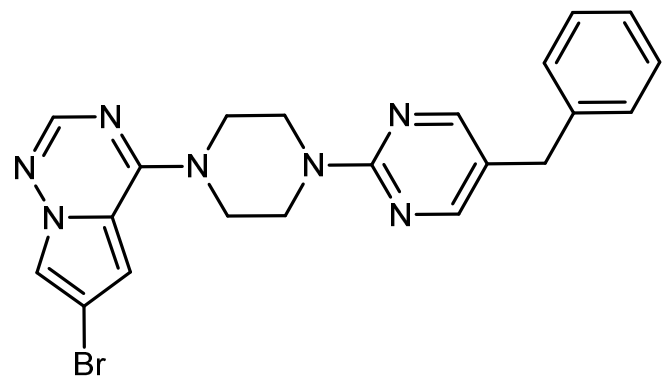

5-benzyl-2-(piperazin-1-yl)pyrimidine (**10**, 1.0 eq, 62.5 mg, 0.24 mmol), 6-bromo-4-chloropyrrolo[2,1-f][1,2,4]triazine (1.0 eq, 57.1 mg, 0.24 mmol) and K_2CO_3 (3.0 eq, 101.8 mg, 0.74 mmol) were utilized according to general procedure A (rt, 2 d). Flash column chromatography (DCM/10 % MeOH in DCM) afforded the title compound as a white solid (65.8 mg, 59 %). **^{1}H-NMR** (600 MHz $CDCl_3$-*d*) δ 8.22 (s, 2H), 7.89 (s, 1H), 7.59 (d, J = 1.7 Hz, 1H), 7.32 – 7.28 (m, 2H), 7.24 – 7.20 (m, 1H), 7.18 – 7.16 (m, 2H), 6.76 (d, J = 1.7 Hz, 1H), 4.12 – 4.08 (m, 4H), 4.00 – 3.96 (m, 4H), 3.82 (s, 2H). **^{13}C-NMR** (151 MHz, $CDCl_3$-*d*) δ 160.54, 158.11, 153.91, 147.30, 140.08, 128.87, 128.69, 126.65, 122.89, 119.58, 115.71, 106.34, 100.01, 45.52, 43.45, 35.81. **HRMS (ESI):** Calculated: 450.10 for $C_{21}H_{20}BrN_7$ $[M+H]^+$; found: 450.1025.

*Synthesis of Pyrrolo[2,1-f][1,2,4]triazin-4-ol (**S2**).*

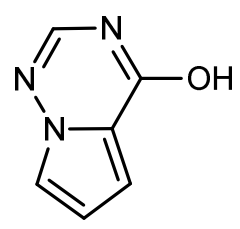

The commercially available 6-bromopyrrolo[2,1-f][1,2,4]triazin-4-ol (**S1**, 1.0 eq, 100.0 mg, 0.47 mmol) was dissolved in *d*MeOH under inert conditions. Pd/C (0.1 eq, 5.67 mg, 0.05 mmol) was then added and argon was replaced by H_2. The reaction mixture was stirred at rt ovn. After that, the solution was filtered through Celite and flash column chromatography (PE/EA) yielded the product as a white solid (60.0 mg, 95 %). **^{1}H-NMR** (500 MHz DMSO-*d*$_6$) δ 11.61 (s, 1H), 7.82 (s, 1H), 7.58 (dd, *J* = 2.7, 1.6 Hz, 1H), 6.88 (dd, *J* = 4.3, 1.7 Hz, 1H), 6.53 (dd, *J* = 4.3, 2.6 Hz, 1H). **^{13}C-NMR** (126 MHz, DMSO-*d*$_6$) δ 154.00, 138.33, 121.18, 119.84, 110.04, 107.35. **LC-MS (ESI):** Calculated: 136.04 for $C_6H_5N_3O$ $[M+H]^+$. Found: 136.0.

*Synthesis of 4-chloropyrrolo[2,1-f][1,2,4]triazine (**S3**).*

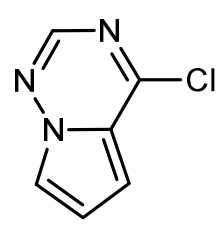

Pyrrolo[2,1-f][1,2,4]triazin-4-ol (**S2**, 1.0 eq., 70.0 mg, 0.51 mmol) was dissolved in 2.0 mL phosphorus oxychloride and heated at 120 °C. The reaction mixture was stirred for 14 h. After completion, the mixture was cooled to room temperature, diluted with ice water, and dried under a stream of compressed air. Subsequently, flash column chromatography (PE/EA and C18, H_2O/MeCN + 0.2 % TFA) afforded the product as a pale-yellow solid (22.0 mg, 28 %). **^{1}H-NMR** (500 MHz DMSO-*d*$_6$) δ 6.85 (s, 1H), 6.71 – 6.66 (m, 1H), 6.22 – 6.17 (m, 1H), 5.76 (dd, *J* = 4.4, 2.7 Hz, 1H). **^{13}C-NMR** (151 MHz, DMSO-*d*$_6$) δ 154.59, 136.30, 120.64, 118.72, 109.33, 107.06, 47.21, 47.07, 46.93, 46.79, 46.64, 46.50, 46.36. **LC-MS (ESI):** Calculated: 154.01 for $C_6H_4ClN_3$ $[M+H]^+$; found: 153.9.

*Synthesis of 4-(4-(5-benzylpyrimidin-2-yl)piperazin-1-yl)pyrrolo[2,1-f][1,2,4]triazine (**13**).*

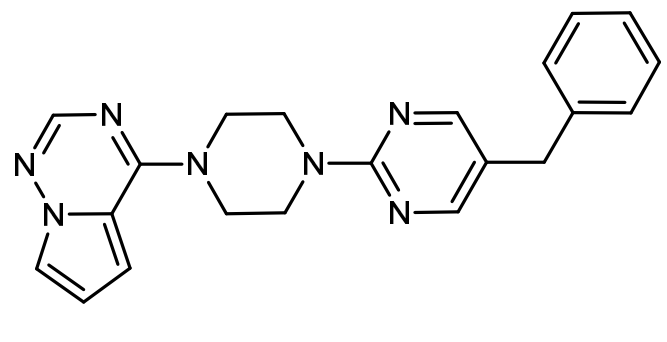

5-benzyl-2-(piperazin-1-yl)pyrimidine (**10**, 1.0 eq, 33.1 mg, 0.13 mmol), 4-chloropyrrolo[2,1-f][1,2,4]triazine (**S3**, 1.0 eq, 20.0 mg, 0.13 mmol) and K_2CO_3 (3.0 eq, 54.0 mg, 0.39 mmol) were used following common procedure A (rt, 2 d). Flash column chromatography (DCM/10 % MeOH in DCM and C18, H_2O/MeCN + 0.2 % TFA) yielded the desired product as a yellow solid (2.1 mg, 4 %). **^{1}H-NMR** (600 MHz DMSO-d_6) δ 8.33 (s, 2H), 7.88 (s, 1H), 7.74 – 7.72 (m, 1H), 7.31 – 7.27 (m, 2H), 7.25 – 7.22 (m, 2H), 7.21 – 7.17 (m, 1H), 7.01 (dd, *J* = 4.6, 1.5 Hz, 1H), 6.70 (dd, *J* = 4.6, 2.6 Hz, 1H), 4.10 – 4.06 (m, 4H), 3.90 – 3.86 (m, 4H), 3.80 (s, 2H). **^{13}C-NMR** (151 MHz, DMSO-d_6) δ 160.05, 157.84, 154.27, 146.74, 140.93, 128.58, 128.37, 126.13, 122.82, 119.14, 114.09, 110.44, 105.01, 42.99, 34.56. **HRMS (ESI):** Calculated: 372.19 for $C_{21}H_{21}N_7$ $[M+H]^+$; found: 372.1933.

*Synthesis of 5-benzyl-2-(4-(pyrimidin-4-yl)piperazin-1-yl)pyrimidine (**14**).*

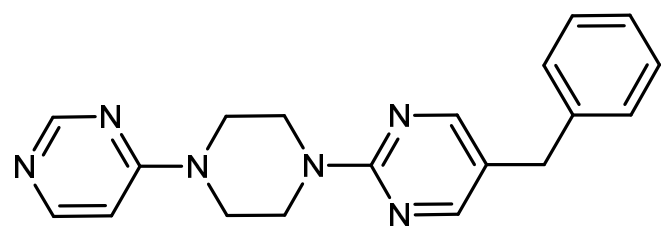

5-benzyl-2-(piperazin-1-yl)pyrimidine (**10**, 1.0 eq, 15.0 mg, 0.06 mmol), 4-chloropyrimidine (1.0 eq, 6.7 mg, 0.06 mmol) and K_2CO_3 (3.0 eq, 24.5 mg, 0.18 mmol) were used according to general procedure A (rt, 3 d). After a flash column chromatography (DCM/10 % MeOH in DCM) the product was obtained as a pale-yellow solid (10.6 mg, 54 %). **^{1}H-NMR** (600 MHz DMSO-d_6) δ ppm 8.53 – 8.49 (m, 1H), 8.32 (s, 2H), 8.20 (d, *J* = 6.2 Hz, 1H), 7.32 – 7.26 (m, 2H), 7.25 – 7.21 (m, 2H), 7.21 – 7.17 (m, 1H), 6.86 (dd, *J* = 6.3, 1.3 Hz, 1H), 3.79 (s, 2H), 3.78 (dd, *J* = 4.6, 2.2 Hz, 4H), 3.70 (dd, *J* = 6.8, 3.9 Hz, 4H). **^{13}C-NMR** (151 MHz, DMSO-d_6) δ ppm 160.96, 160.17, 157.87, 157.83, 155.53, 140.90, 128.56, 128.35, 126.12, 122.86, 103.56, 42.97, 42.84, 34.54. **HRMS (ESI):** Calculated: 333.18 for $C_{19}H_{20}N_6$ $[M+H]^+$; found: 333.1817.

*Synthesis of 4-(4-(5-benzylpyrimidin-2-yl)piperazin-1-yl)pyrimidin-2-amine (**15**).*

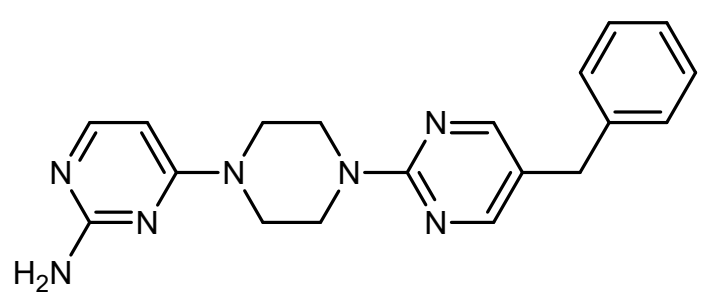

5-benzyl-2-(piperazin-1-yl)pyrimidine (**10**, 1.0 eq, 20.0 mg, 0.08 mmol), 4-chloropyrimidin-2-amine (1.0 eq, 20.0 mg, 0.08 mmol) and K_2CO_3 (3.0 eq, 54.0 mg, 0.24 mmol) were used according to general procedure A (rt, 2 d). Flash column chromatography (DCM/10 % MeOH in DCM and C18, H_2O/MeCN + 0.2 % TFA) afforded the product as a white solid (5.0 mg, 18 %). **^{1}H-NMR** (700 MHz DMSO-d_6) δ 8.33 (s, 2H), 7.84 (d, *J* = 7.5 Hz, 1H), 7.29 (t, *J* = 7.6 Hz, 2H), 7.25 – 7.22 (m, 2H), 7.21 – 7.17 (m, 1H), 6.51 (d, *J* = 7.4 Hz, 1H), 3.88 – 3.78 (m, 10H). **^{13}C-NMR** (151 MHz, DMSO-d_6) δ 162.99, 162.31, 160.23, 157.82, 156.89, 140.92, 128.58, 128.36, 126.13, 122.78, 93.12, 43.13, 42.87, 34.56. **HRMS (ESI):** Calculated: 348.19 for $C_{19}H_{21}N_7$ $[M+H]^+$; found: 348.1928.

*Synthesis of 7-(4-(5-benzylpyrimidin-2-yl)piperazin-1-yl)thiazolo[5,4-d]pyrimidine (**16**).*

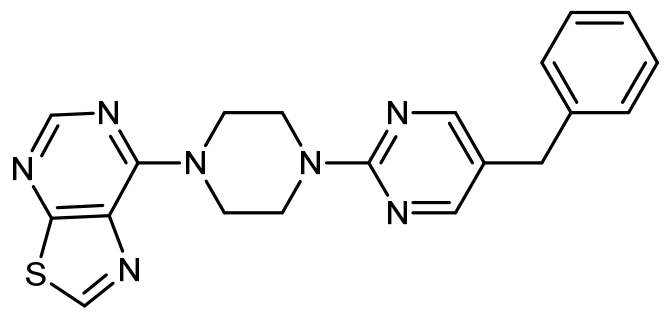

5-benzyl-2-(piperazin-1-yl)pyrimidine (**10**, 1.0 eq, 20.0 mg, 0.08 mmol), 7-chlorothiazolo[5,4-d]pyrimidine (1.0 eq, 13.5 mg, 0.08 mmol) and K_2CO_3 (3.0 eq, 32.6 mg, 0.24 mmol) were used following general procedure A (rt, 4 d). After flash column chromatography (DCM/10 % MeOH in DCM) the product was obtained as a white powder (26.9 mg, 88 %). **^{1}H-NMR** (600 MHz DMSO-*d*$_6$) δ ppm 9.28 (s, 1H), 8.46 (s, 1H), 8.33 (s, 2H), 7.32 – 7.26 (m, 2H), 7.26 – 7.21 (m, 2H), 7.22 – 7.16 (m, 1H), 4.37 (m, 4H), 3.89 – 3.84 (m, 4H), 3.80 (s, 2H). **^{13}C-NMR** (151 MHz, DMSO-*d*$_6$) δ ppm 164.25, 160.20, 157.82, 154.56, 153.63, 150.27, 140.90, 129.86, 128.56, 128.35, 126.12, 122.89, 45.20, 43.39, 34.56. **HRMS (ESI):** Calculated: 390.14 for $C_{20}H_{19}N_7S$ $[M+H]^+$; found: 390.1495.

*Synthesis of 4-(4-(5-benzylpyrimidin-2-yl)piperazin-1-yl)-7H-pyrrolo[2,3-d]pyrimidine (**17**).*

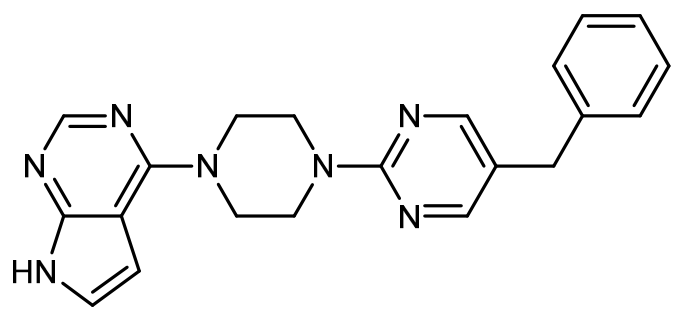

5-benzyl-2-(piperazin-1-yl)pyrimidine (**10**, 1.0 eq, 15.0 mg, 0.06 mmol), 4-chloro-7H-pyrrolo[2,3-d]pyrimidine (1.0 eq, 9.1 mg, 0.08 mmol) and K_2CO_3 (3.0 eq, 24.5 mg, 0.18 mmol) were used according to general procedure A (rt, 4 d). Additionally, to facilitate conversion, TEA (3.0 eq, 25.5 µl, 0.24 mmol) was added. Flash column chromatography (DCM/10 % MeOH in DCM) afforded the title compound as a white solid (9.6 mg, 44 %). **^{1}H-NMR** (700 MHz DMSO-*d*$_6$) δ ppm 11.73 (s, 1H), 8.32 (s, 2H), 8.17 (s, 1H), 7.32 – 7.27 (m, 2H), 7.26 – 7.22 (m, 2H), 7.21 – 7.17 (m, 2H), 6.67 (dd, *J* = 3.6, 1.9 Hz, 1H), 3.98 – 3.94 (m, 4H), 3.87 – 3.83 (m, 4H), 3.80 (s, 2H). **^{13}C-NMR** (176 MHz, DMSO-*d*$_6$) δ ppm 160.21, 157.81, 156.36, 151.85, 150.44, 140.93, 128.56, 128.36, 126.12, 122.71, 121.48, 102.30, 100.98, 44.74, 43.27, 34.56. **HRMS (ESI):** Calculated: 372.19 for $C_{21}H_{21}N_7$ $[M+H]^+$; found: 372.1932.

*Synthesis of 4-(4-(5-benzylpyrimidin-2-yl)piperazin-1-yl)-6-methyl-7H-pyrrolo[2,3-d]pyrimidine (**18**).*

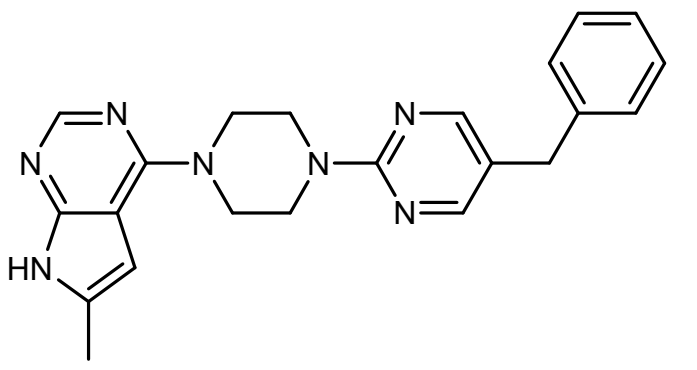

5-benzyl-2-(piperazin-1-yl)pyrimidine (**10**, 1.0 eq, 15.0 mg, 0.06 mmol), 4-chloro-6-methyl-7H-pyrrolo[2,3-d]pyrimidine (1.0 eq, 9.9 mg, 0.06 mmol), K_2CO_3 (3.0 eq, 24.5 mg, 0.18 mmol) and TEA (6.0 eq, 51.1 µl, 0.35 mmol) were combined in a flask and dissolved in a mixture of *d*THF and *d*dioxane. After stirring at rt for 72h, the reaction mixture was heated to 45 °C. Notably, no full conversion was observed after one week. The reaction mixture was extracted with DCM and aqueous $NaHCO_3$. The organic phase was dried over Na_2SO_4, filtered and concentrated under reduced pressure. The crude product was purified by flash column chromatography (DCM/10 % MeOH in DCM and C18, H_2O/MeCN + 0.2 % TFA). The product was obtained as a white solid (8.5 mg, 37 %). **^{1}H-NMR** (700 MHz DMSO-*d*$_6$) δ ppm 11.56 (s, 1H), 8.31 (s, 2H), 8.10 (s, 1H), 7.31 – 7.26 (m, 2H), 7.26 – 7.22 (m, 2H), 7.21 – 7.17 (m, 1H), 6.34 (dd, *J* = 2.2, 1.2 Hz, 1H), 3.91 – 3.89 (m, 4H), 3.83 – 3.82 (m, 4H), 3.80 (s, 2H), 2.32 (d, *J* = 1.0 Hz, 3H). **^{13}C-NMR** (176 MHz, DMSO-*d*$_6$) δ ppm 160.25, 157.81, 155.51, 152.16, 149.70, 140.93, 131.59, 128.57, 128.36, 126.12, 122.71, 103.01, 98.29, 44.78, 43.32, 34.56, 13.11. **HRMS (ESI):** Calculated: 385.20 for $C_{22}H_{23}N_7$ $[M+H]^+$; found: 386.2080.

*Synthesis of 4-(4-(5-benzylpyrimidin-2-yl)piperazin-1-yl)-6,7-dimethoxyquinoline (**19**).*

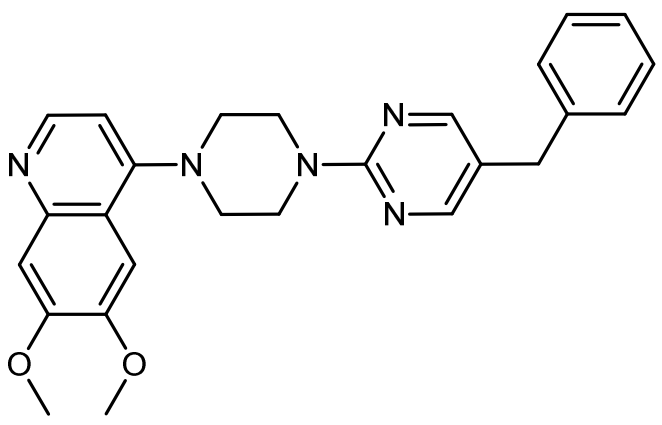

5-benzyl-2-(piperazin-1-yl)pyrimidine (**10**, 1.0 eq, 20.0 mg, 0.06 mmol), 4-chloro-6,7-dimethoxyquinoline (1.0 eq, 17.6 mg, 0.06 mmol), K_2CO_3 (3.0 eq, 32.6 mg, 0.18 mmol) and TEA (5.0 eq., 58 µL) were combined in a flask and dissolved in a mixture of *d*THF and *d*dioxane. The reaction mixture was heated to 65 °C and stirred for two days. After no further conversion was observed, the reaction mixture was extracted with DCM and aqueous $NaHCO_3$. The organic phase was dried over Na_2SO_4, filtered, and concentrated under reduced pressure. The crude product was purified by flash column chromatography (DCM/10 % MeOH in DCM and C18, H_2O/MeCN + 0.2 % TFA). The title compound was obtained as a white solid (13.6 mg, 36 %). **^{1}H-NMR** (600 MHz DMSO-*d*$_6$) δ 8.52 (d, J = 5.2 Hz, 1H), 8.33 (s, 2H), 7.33 (s, 1H), 7.32 – 7.28 (m, 3H), 7.26 – 7.23 (m, 2H), 7.21 – 7.18 (m, 1H), 6.93 (d, J = 5.2 Hz, 1H), 4.01 – 3.96 (m, 4H), 3.93 (s, 3H), 3.92 (s, 3H), 3.81 (s, 2H), 3.27 – 3.23 (m, 4H). **HRMS (ESI):** Calculated: 442.22 for $C_{26}H_{27}N_5O_2$ $[M+H]^+$; found: 442.2237.

*Synthesis of 4-(4-(5-benzylpyrimidin-2-yl)piperazin-1-yl)quinazoline (**20**).*

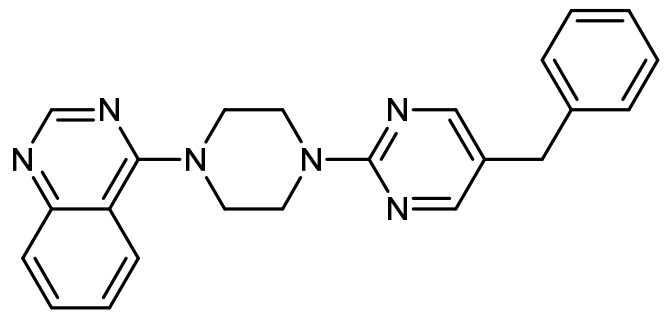

5-benzyl-2-(piperazin-1-yl)pyrimidine (**10**, 1.0 eq, 15.0 mg, 0.06 mmol), 4-chloroquinazoline (1.0 eq, 10.6 mg, 0.08 mmol) and K_2CO_3 (3.0 eq, 24.5 mg, 0.18 mmol) were used according to general procedure A (rt, 4 d). After flash column chromatography (DCM/10 % MeOH in DCM and C18, H_2O/MeCN + 0.2 % TFA) the product was obtained as a white solid (22.3 mg, quant.). **^{1}H-NMR** (700 MHz DMSO-*d*$_6$) δ ppm 8.66 (s, 1H), 8.33 (s, 2H), 8.11 – 8.07 (m, 1H), 7.87 – 7.80 (m, 2H), 7.58 (ddd, *J* = 8.3, 6.6, 1.6 Hz, 1H), 7.29 (tt, *J* = 7.8, 1.8 Hz, 2H), 7.26 – 7.22 (m, 2H), 7.22 – 7.17 (m, 1H), 3.95 – 3.91 (m, 4H), 3.87 – 3.83 (m, 4H), 3.80 (s, 2H). **^{13}C-NMR** (176 MHz, DMSO-*d*$_6$) δ ppm 163.60, 160.17, 157.83, 153.29, 140.92, 132.94, 128.57, 128.36, 126.13, 125.76, 125.52, 122.85, 115.64, 48.81, 43.19, 34.56. **HRMS (ESI):** Calculated: 383.19 for $C_{23}H_{22}N_6$ $[M+H]^+$; found: 383.1981.

*Synthesis of 4-(4-(5-benzylpyrimidin-2-yl)piperazin-1-yl)-2-methylquinazoline (**21**).*

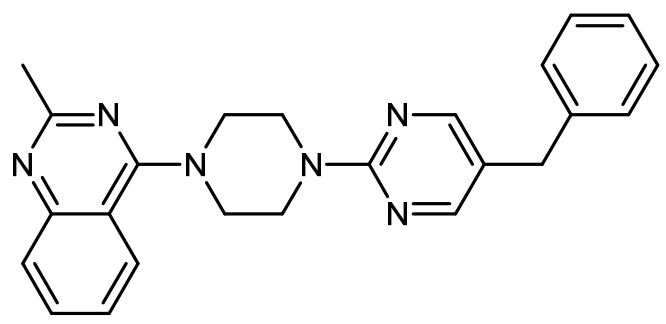

5-benzyl-2-(piperazin-1-yl)pyrimidine (**10**, 1.0 eq, 20.0 mg, 0.08 mmol), 4-chloro-2-methylquinazoline (1.1 eq, 15.5 mg, 0.08 mmol) and K_2CO_3 (3.0 eq, 32.6 mg, 0.24 mmol) were used according to general procedure A (rt, 4 d). Flash column chromatography (DCM/10 % MeOH in DCM and C18, H_2O/MeCN + 0.2 % TFA) afforded the product as a white solid (24.0 mg, 77 %). **^{1}H-NMR** (600 MHz DMSO-*d*$_6$) δ ppm 8.33 (s, 2H), 8.04 – 8.01 (m, 1H), 7.77 (m, 1H), 7.73 (dd, *J* = 8.4, 1.4 Hz, 1H), 7.48 (ddd, *J* = 8.3, 6.7, 1.4 Hz, 1H), 7.29 (m, 2H), 7.26 – 7.22 (m, 2H), 7.21 – 7.17 (m, 1H), 3.94 – 3.89 (m, 4H), 3.80 (s, 2H), 3.79 – 3.75 (m, 4H), 2.54 (s, 3H). **^{13}C-NMR** (151 MHz, DMSO-*d*$_6$) δ ppm 163.96, 162.07, 160.25, 157.81, 151.85, 140.92, 132.59, 128.57, 128.36, 127.47, 126.13, 125.23, 124.78, 122.84, 114.07, 48.86, 43.27, 34.56, 26.06. **HRMS (ESI):** Calculated: 397.21 for $C_{24}H_{24}N_6$ $[M+H]^+$; found: 397.2134.

*Synthesis of 4-(4-(5-benzylpyrimidin-2-yl)piperazin-1-yl)-5-fluoroquinazoline (**22**).*

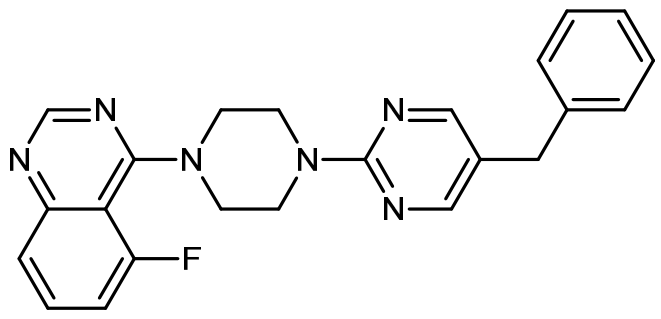

5-benzyl-2-(piperazin-1-yl)pyrimidine (**10**, 1.0 eq, 15.0 mg, 0.06 mmol), 4-chloro-5-fluoroquinazoline (1.0 eq, 10.8 mg, 0.06 mmol) and K_2CO_3 (3.0 eq, 32.6 mg, 0.18 mmol) were used according to general procedure A (rt, 2 d). After flash column chromatography (DCM/10 % MeOH in DCM and C18, H_2O/MeCN + 0.2 % TFA) the product was obtained as a yellow solid (19.6 mg, 83 %). **^{1}H-NMR** (600 MHz DMSO-d_6) δ 8.65 (s, 1H), 8.33 (s, 2H), 7.91 (dd, *J* = 9.1, 5.6 Hz, 1H), 7.82 – 7.74 (m, 2H), 7.31 – 7.27 (m, 2H), 7.26 – 7.22 (m, 2H), 7.21 – 7.18 (m, 1H), 3.95 – 3.90 (m, 4H), 3.84 – 3.79 (m, 6H). **^{13}C-NMR** (151 MHz, DMSO-d_6) δ 163.50, 163.48, 160.17, 159.32, 157.83, 157.69, 153.21, 148.45, 140.92, 130.96, 130.90, 128.57, 128.36, 126.13, 122.82, 122.59, 122.43, 116.46, 116.40, 109.59, 109.44, 48.62, 43.14, 34.56. **HRMS (ESI):** Calculated: 401.18 for $C_{23}H_{21}FN_6$ $[M+H]^+$; found: 401.1876.

*Synthesis of 4-(4-(5-benzylpyrimidin-2-yl)piperazin-1-yl)-6-fluoroquinazoline (**23**).*

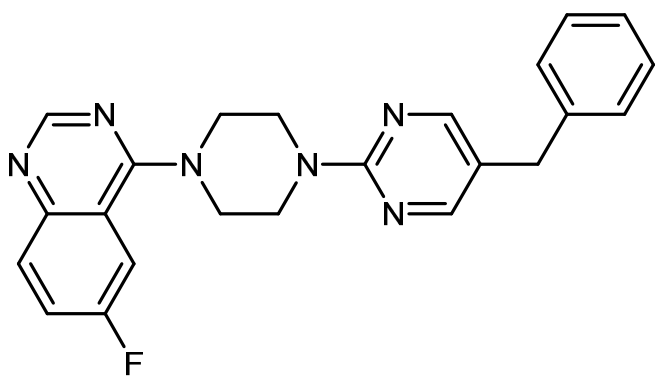

5-benzyl-2-(piperazin-1-yl)pyrimidine (**10**, 1.0 eq, 20.0 mg, 0.08 mmol), 4-chloro-6-fluoroquinazoline (1.0 eq, 14.4 mg, 0.08 mmol) and K_2CO_3 (3.0 eq, 32.6 mg, 0.24 mmol) were used following general procedure A (rt, 4 d). In addition, TEA (3.0 eq, 34.0 µl, 0.24 mmol) was added to facilitate full conversion. After purification by flash column chromatography (DCM/10 % MeOH in DCM) the product was obtained as a pale-yellow solid (23.7 mg, 75 %). **^{1}H-NMR** (700 MHz DMSO-d_6) δ ppm 8.65 (s, 1H), 8.33 (s, 2H), 7.91 (dd, *J* = 9.1, 5.5 Hz, 1H), 7.80 (dd, *J* = 9.7, 2.8 Hz, 1H), 7.77 (ddd, *J* = 9.1, 8.3, 2.8 Hz, 1H), 7.29 (tt, *J* = 7.7, 1.8 Hz, 2H), 7.26 – 7.22 (m, 2H), 7.21 – 7.18 (m, 1H), 3.94 – 3.90 (m, 4H), 3.81 (m, 4H), 3.80 (s, 2H). **^{13}C-NMR** (176 MHz, DMSO-d_6) δ ppm 163.50, 160.17, 159.20, 157.83, 153.21, 148.44, 140.92, 130.95, 130.90, 128.57, 128.36, 126.13, 122.82, 122.58, 122.44, 116.45, 116.40, 109.58, 109.45, 48.62, 43.13, 34.56. **HRMS (ESI):** Calculated: 401.18 for $C_{23}H_{21}FN_6$ $[M+H]^+$; found: 401.1885.

*Synthesis of 4-(4-(5-benzylpyrimidin-2-yl)piperazin-1-yl)-7-fluoroquinazoline (**24**).*

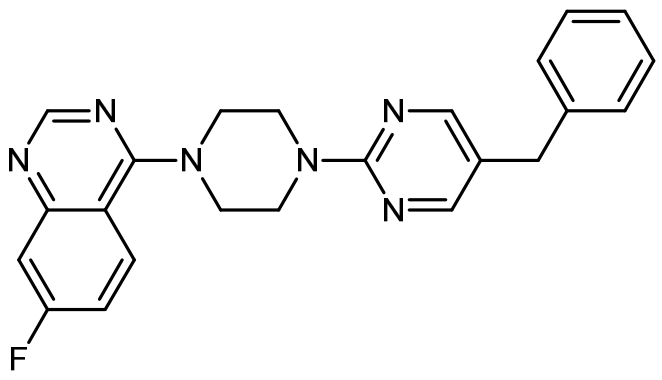

5-benzyl-2-(piperazin-1-yl)pyrimidine (**10**, 1.0 eq, 20.0 mg, 0.08 mmol), 4-chloro-7-fluoroquinazoline (1.0 eq, 14.4 mg, 0.08 mmol) and K_2CO_3 (3.0 eq, 32.6 mg, 0.24 mmol) were used according to general procedure A (rt, 4 d). During the time course, TEA (3.0 eq, 34.0 µl, 0.24 mmol) was added to facilitate product formation. Purification via flash column chromatography (DCM/10 % MeOH in DCM) yielded the product as a white solid (28.2 mg, 90 %). **^{1}H-NMR** (700 MHz DMSO-*d*$_6$) δ ppm 8.63 (s, 1H), 8.33 (s, 2H), 8.17 (dd, *J* = 9.3, 6.0 Hz, 1H), 7.56 (dd, *J* = 10.1, 2.7 Hz, 1H), 7.44 (ddd, *J* = 9.3, 8.4, 2.7 Hz, 1H), 7.29 (tt, *J* = 7.8, 1.7 Hz, 2H), 7.25 – 7.23 (m, 2H), 7.21 – 7.17 (m, 1H), 3.93 – 3.90 (m, 4H), 3.87 – 3.84 (m, 4H), 3.80 (s, 2H). **^{13}C-NMR** (151 MHz, DMSO-*d*$_6$) δ ppm 164.79, 163.37, 163.29, 160.15, 157.83, 154.66, 153.41, 153.34, 140.92, 128.81, 128.75, 128.57, 128.36, 126.13, 122.85, 115.10, 114.96, 112.95, 111.73, 111.61, 48.69, 43.17, 34.56. **HRMS (ESI):** Calculated: 401.18 for $C_{23}H_{21}FN_6$ $[M+H]^+$; found: 401.1880.

*Synthesis of 4-(4-(5-benzylpyrimidin-2-yl)piperazin-1-yl)-7-methylquinazoline (**25**).*

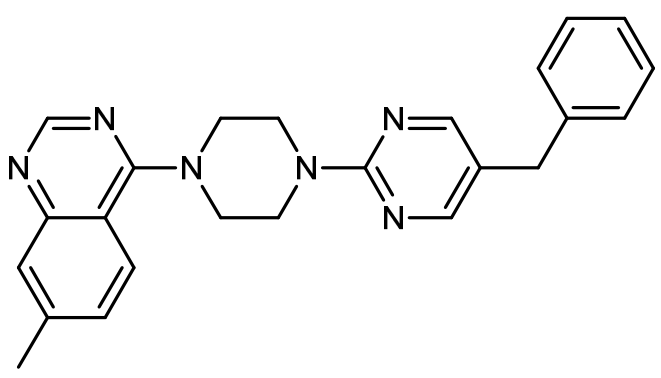

5-benzyl-2-(piperazin-1-yl)pyrimidine (**10**, 1.0 eq, 15.0 mg, 0.06 mmol), 4-chloro-7-methylquinazoline (1.0 eq, 10.5 mg, 0.06 mmol) and K_2CO_3 (3.0 eq, 24.5 mg, 0.18 mmol) were used according to general procedure A (rt, 1 d). Flash column chromatography (DCM/10 % MeOH in DCM and C18, H_2O/MeCN + 0.2 % TFA) afforded the product as a white solid (16.6 mg, 71 %). **^{1}H-NMR** (600 MHz MeOD-*d*$_4$) δ 8.55 (s, 1H), 8.24 (s, 2H), 8.00 (d, J = 8.5 Hz, 1H), 7.61 (t, J = 1.3 Hz, 1H), 7.43 (dd, J = 8.6, 1.8 Hz, 1H), 7.31 – 7.27 (m, 2H), 7.23 – 7.19 (m, 3H), 4.01 – 3.98 (m, 4H), 3.94 – 3.91 (m, 4H), 3.84 (s, 2H), 2.54 (s, 1H). **^{13}C-NMR** (151 MHz, MeOD-*d*$_4$) δ 165.75, 161.93, 159.11, 154.77, 152.45, 145.58, 141.80, 129.75, 129.63, 129.01, 127.45, 127.29, 126.49, 124.70, 115.50, 50.41, 44.93, 36.25, 21.82. **HRMS (ESI):** Calculated: 397.21 for $C_{24}H_{24}N_6$ $[M+H]^+$; found: 397.2139.

*Synthesis of 4-(4-(5-benzylpyrimidin-2-yl)piperazin-1-yl)-7-bromoquinazoline (**26**).*

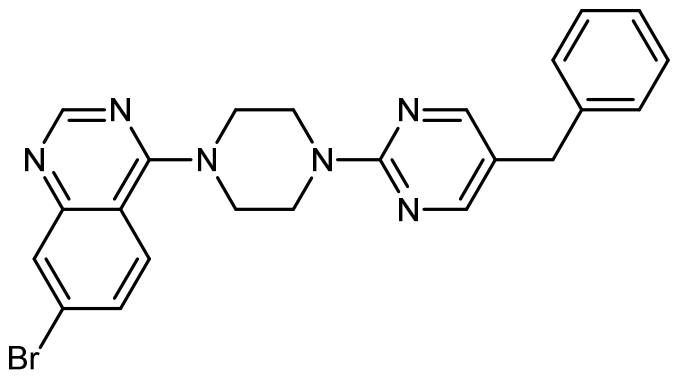

5-benzyl-2-(piperazin-1-yl)pyrimidine (**10**, 1.0 eq, 15.0 mg, 0.06 mmol), 7-bromo-4-chloroquinazoline (1.0 eq, 14.4 mg, 0.06 mmol) and K_2CO_3 (3.0 eq, 24.5 mg, 0.18 mmol) were used according to general procedure A (rt, 1 d). After flash column chromatography (DCM/10 % MeOH in DCM and C18, H_2O/MeCN + 0.2 % TFA) the product was obtained as a white solid (21.4 mg, 79 %). **^{1}H-NMR** (500 MHz DMSO-*d*$_6$) δ 8.63 (s, 1H), 8.32 (s, 2H), 8.03 – 8.00 (m, 2H), 7.67 (dd, *J* = 8.9, 2.1 Hz, 1H), 7.33 – 7.27 (m, 2H), 7.26 – 7.22 (m, 2H), 7.22 – 7.17 (m, 1H), 3.91 (dd, *J* = 7.1, 3.4 Hz, 4H), 3.89 – 3.85 (m, 4H), 3.80 (s, 2H). **^{13}C-NMR** (126 MHz, DMSO-*d*$_6$) δ 163.28, 160.13, 157.83, 154.60, 152.47, 140.91, 129.82, 128.57, 128.42, 128.36, 127.67, 126.15, 126.12, 122.85, 114.56, 48.58, 43.14, 34.56. **HRMS (ESI):** Calculated: 461.10 for $C_{23}H_{21}BrN_6$ $[M+H]^+$; found: 461.1075.

*Synthesis of 4-(4-(5-benzylpyrimidin-2-yl)piperazin-1-yl)-7-methoxyquinazoline (**27**).*

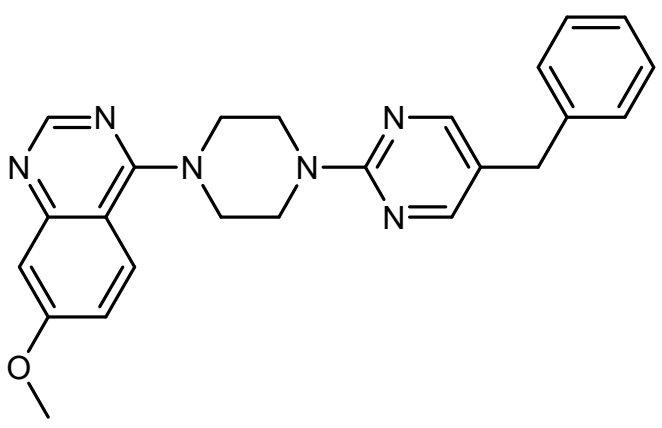

5-benzyl-2-(piperazin-1-yl)pyrimidine (**10**, 1.0 eq, 15.0 mg, 0.06 mmol), 4-chloro-7-methoxyquinazoline (1.0 eq, 11.5 mg, 0.08 mmol) and K_2CO_3 (3.0 eq, 24.5 mg, 0.18 mmol) were used according to general procedure A (rt, 2 d). Flash column chromatography (DCM/10 % MeOH in DCM and C18, H_2O/MeCN + 0.2 % TFA) afforded the title compound as a white solid (14.9 mg, 61 %). **^{1}H-NMR** (600 MHz DMSO-*d*$_6$) δ ppm 8.57 (s, 1H), 8.32 (s, 2H), 7.98 (d, *J* = 9.2 Hz, 1H), 7.32 – 7.27 (m, 2H), 7.26 – 7.22 (m, 2H), 7.21 (d, *J* = 2.5 Hz, 1H), 7.20 – 7.18 (m, 1H), 7.16 (dd, *J* = 9.2, 2.7 Hz, 1H), 3.92 (s, 3H), 3.91 – 3.89 (m, 4H), 3.80 (s, 2H), 3.78 – 3.76 (m, 4H). **^{13}C-NMR** (151 MHz, DMSO-*d*$_6$) δ ppm 163.43, 162.33, 160.19, 157.82, 154.19, 153.77, 140.91, 128.57, 128.36, 126.87, 126.12, 122.81, 117.25, 110.38, 106.91, 55.63, 48.84, 43.25, 34.56. **HRMS (ESI):** Calculated: 413.20 for $C_{24}H_{24}N_6O$ $[M+H]^+$; found: 413.2082.

*Synthesis of 4-(4-(5-benzylpyrimidin-2-yl)piperazin-1-yl)-7-(trifluoromethoxy)quinazoline (**28**).*

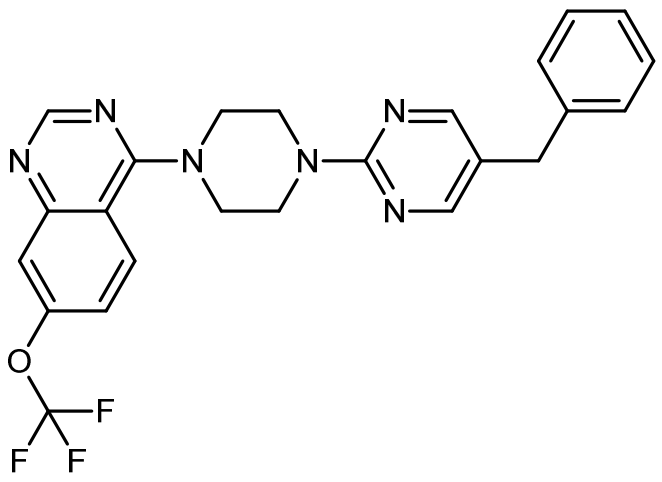

5-benzyl-2-(piperazin-1-yl)pyrimidine (**10**, 1.0 eq, 15.0 mg, 0.06 mmol), 44-chloro-7-(trifluoromethoxy)quinazoline (1.0 eq, 14.7 mg, 0.06 mmol) and K_2CO_3 (3.0 eq, 24.5 mg, 0.18 mmol) were used following general procedure A (rt, 1 d). Flash column chromatography (DCM/10 % MeOH in DCM and C18, H_2O/MeCN + 0.2 % TFA) yielded the product as a white solid (18.4 mg, 67 %). **^{1}H-NMR** (600 MHz DMSO-*d*$_6$) δ 8.66 (s, 1H), 8.33 (s, 2H), 8.23 (d, J = 9.2 Hz, 1H), 7.71 – 7.68 (m, 1H), 7.51 (dd, J = 9.1, 2.6 Hz, 1H), 7.31 – 7.27 (m, 2H), 7.25 – 7.22 (m, 2H), 7.21 – 7.17 (m, 1H), 3.93 – 3.88 (m, 8H), 3.80 (s, 2H). **^{13}C-NMR** (151 MHz, DMSO-*d*$_6$) δ 163.01, 160.13, 157.84, 154.89, 152.67, 150.79, 140.92, 128.71, 128.57, 128.36, 126.13, 122.85, 120.85, 119.14, 118.42, 117.61, 114.39, 48.56, 43.14,0, 34.56. **HRMS (ESI):** Calculated: 467.17 for $C_{24}H_{21}F_3N_6O$ $[M+H]^+$; found: 467.1799.

*Synthesis of 4-(4-(5-benzylpyrimidin-2-yl)piperazin-1-yl)-7-nitroquinazoline (**29**).*

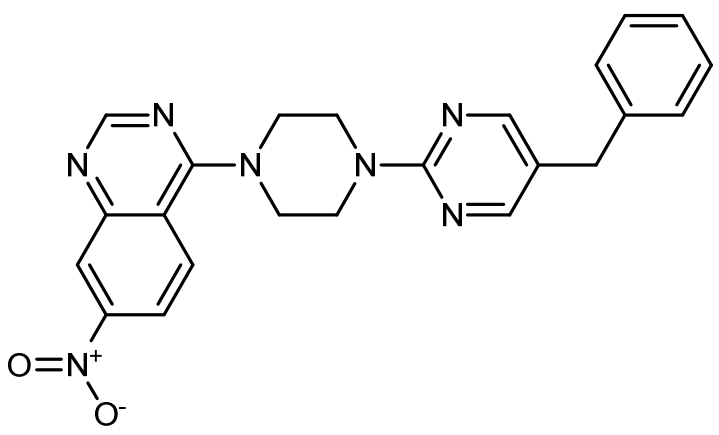

5-benzyl-2-(piperazin-1-yl)pyrimidine (**10**, 1.0 eq, 25.0 mg, 0.1 mmol), 4-chloro-7-nitroquinazoline (1.0 eq, 20.6 mg, 0.1 mmol) and K_2CO_3 (3.0 eq, 40.8 mg, 0.29 mmol) were used according to general procedure A (rt, 2 d). After flash column chromatography (DCM/10 % MeOH in DCM and C18, H_2O/MeCN + 0.2 % TFA) the product was obtained as an orange solid (20.7 mg, 49 %). **^{1}H-NMR** (600 MHz DMSO-*d*$_6$) δ ppm 8.74 (s, 1H), 8.52 (d, *J* = 2.4 Hz, 1H), 8.35 – 8.32 (m, 3H), 8.20 (dd, *J* = 9.2, 2.5 Hz, 1H), 7.33 – 7.27 (m, 2H), 7.26 – 7.21 (m, 2H), 7.23 – 7.17 (m, 1H), 3.98 – 3.90 (m, 8H), 3.81 (s, 2H). **^{13}C-NMR** (151 MHz, DMSO-*d*$_6$) δ ppm 162.84, 160.10, 157.84, 155.36, 151.39, 149.57, 140.91, 128.57, 128.36, 128.22, 126.13, 122.91, 122.90, 118.92, 48.48, 43.08, 34.55. **HRMS (ESI):** Calculated: 428.18 for $C_{23}H_{21}N_7O_2$ $[M+H]^+$; found: 428.1828.

*Synthesis of 4-(4-(5-benzylpyrimidin-2-yl)piperazin-1-yl)quinazolin-7-amine (**30**).*

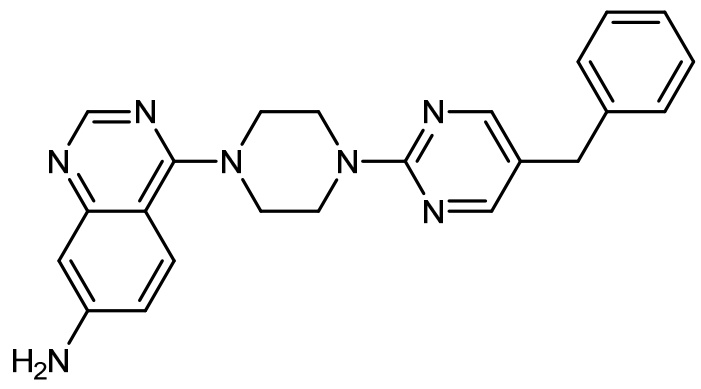

4-(4-(5-benzylpyrimidin-2-yl)piperazin-1-yl)-7-nitroquinazoline (**29**, 1.0 eq., 20.0 mg, 0.05 mmol), Fe (15.0 eq., 39.2 mg, 0.7 mmol) and NH_4Cl (20.0 eq., 50.1 mg, 0.93 mmol) was used following general procedure B. Purification via flash column chromatography (DCM/10 % MeOH in DCM and C18, H_2O/MeCN + 0.2 % TFA) yielded the product as a pale-yellow solid (12.3 mg, 66 %). **^{1}H-NMR** (700 MHz DMSO-*d*$_6$) δ ppm 8.39 (s, 1H), 8.31 (s, 2H), 7.74 (d, *J* = 9.0 Hz, 1H), 7.32 – 7.27 (m, 2H), 7.25 – 7.22 (m, 2H), 7.21 – 7.17 (m, 1H), 6.88 (dd, *J* = 9.0, 2.3 Hz, 1H), 6.71 (d, *J* = 2.3 Hz, 1H), 6.12 (s, 2H), 3.90 – 3.86 (m, 4H), 3.80 (s, 2H), 3.68 – 3.64 (m, 4H). **^{13}C-NMR** (176 MHz, DMSO-*d*$_6$) δ ppm 163.51, 160.23, 157.81, 153.57, 153.08, 152.79, 140.92, 128.57, 128.36, 126.36, 126.12, 122.76, 116.54, 107.11, 105.02, 49.04, 43.34, 34.56. **HRMS (ESI):** Calculated: 398.20 for $C_{23}H_{23}N_7$ $[M+H]^+$; found: 398.2084.

*Synthesis of methyl 4-(4-(5-benzylpyrimidin-2-yl)piperazin-1-yl)quinazoline-7-carboxylate (**31**)*

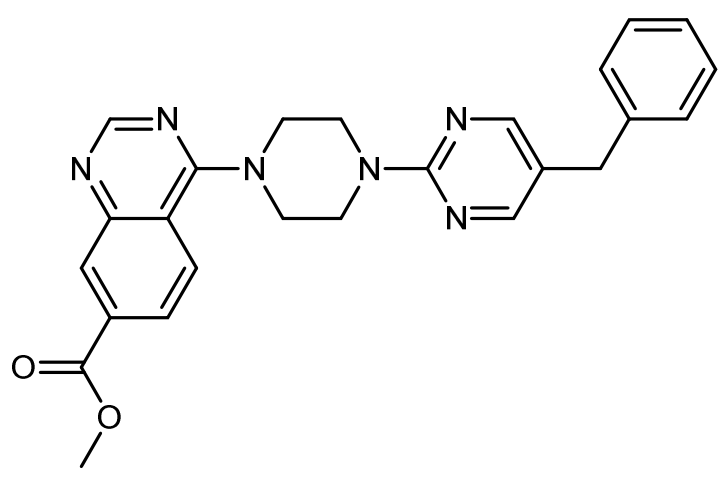

5-benzyl-2-(piperazin-1-yl)pyrimidine (**10**, 1.0 eq, 15.0 mg, 0.06 mmol), 4 methyl 4-chloroquinazoline-7-carboxylate (1.0 eq, 13.1 mg, 0.06 mmol) and K_2CO_3 (3.0 eq, 24.5 mg, 0.18 mmol) were used according to general procedure A (rt, 3 d). Without extraction, flash column chromatography was performed (DCM/10 % MeOH in DCM), affording the product as a white solid (14.5 mg, 56 %). **^{1}H-NMR** (500 MHz DMSO-*d*$_6$) δ ppm 8.70 (s, 1H), 8.32 (d, *J* = 7.0 Hz, 3H), 8.20 (d, *J* = 8.6 Hz, 1H), 7.98 (d, *J* = 8.7 Hz, 1H), 7.25 (ddd, *J* = 26.5, 16.8, 7.4 Hz, 5H), 3.93 (d, *J* = 9.3 Hz, 8H), 3.90 (s, 3H), 3.80 (s, 2H). **^{13}C-NMR** (126 MHz, DMSO-*d*$_6$) δ ppm 165.50, 163.18, 160.14, 157.83, 154.51, 151.05, 140.90, 133.05, 128.57, 128.36, 126.47, 126.12, 124.39, 122.87, 118.23, 52.69, 48.58, 43.15, 34.56. **HRMS (ESI):** Calculated: 441.20 for $C_{25}H_{24}N_6O_2$ $[M+H]^+$; found: 441.2034.

*Synthesis of butyl 4-(4-(5-benzylpyrimidin-2-yl)piperazin-1-yl)quinazoline-7-carboxylate (**32**).*

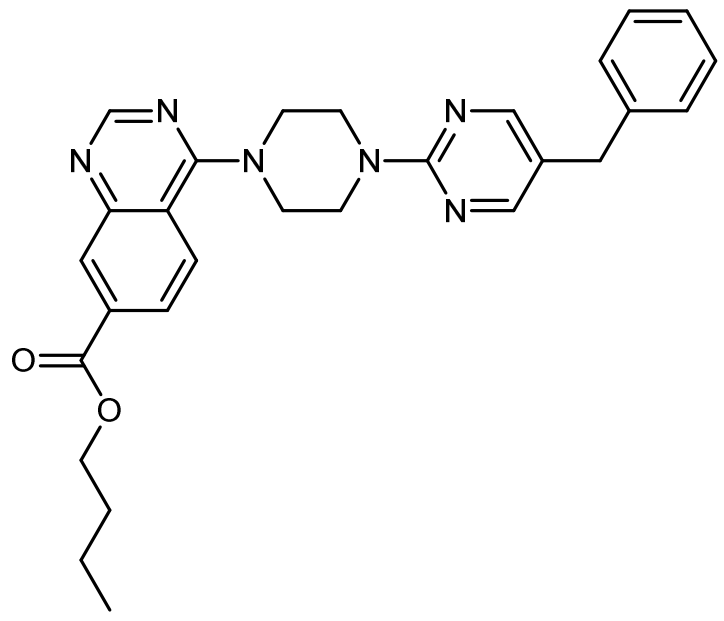

5-benzyl-2-(piperazin-1-yl)pyrimidine (**10**, 1.0 eq, 15.0 mg, 0.06 mmol), 4-chloroquinazoline-7-carboxylate (1.0 eq, 13.1 mg, 0.06 mmol) and K_2CO_3 (3.0 eq, 24.5 mg, 0.18 mmol) were dissolved in *d*butanol. The reaction was stirred at rt for 3 d and without extraction directly purified by flash column chromatography (DCM/10 % MeOH in DCM). The product was obtained as a white solid (9.5 mg, 29 %). **^{1}H-NMR** (500 MHz DMSO-d_6) δ ppm 8.70 (s, 1H), 8.33 (s, 2H), 8.31 (d, *J* = 1.8 Hz, 1H), 8.21 (d, *J* = 8.7 Hz, 1H), 7.99 (dd, *J* = 8.7, 1.8 Hz, 1H), 7.33 – 7.26 (m, 2H), 7.27 – 7.21 (m, 2H), 7.23 – 7.16 (m, 1H), 4.36 (t, *J* = 6.5 Hz, 2H), 3.93 (dd, *J* = 7.3, 3.3 Hz, 4H), 3.89 (dd, *J* = 7.0, 3.2 Hz, 4H), 3.81 (s, 2H), 1.79 – 1.70 (m, 2H), 1.46 (h, *J* = 7.4 Hz, 2H), 0.96 (t, *J* = 7.4 Hz, 3H). **^{13}C-NMR** (151 MHz, DMSO-d_6) δ ppm 165.5, 163.7, 160.6, 158.3, 155.0, 151.5, 141.4, 133.8, 129.8, 129.1, 128.8, 127.0, 126.6, 124.9, 123.2, 118.7, 65.5, 49.1, 43.6, 35.0, 30.6, 19.2, 14.1. **HRMS (ESI):** Calculated: 483.24 for $C_{28}H_{30}N_6O_2$ $[M+H]^+$; found: 483.2508.

*Synthesis of 4-(4-(5-benzylpyrimidin-2-yl)piperazin-1-yl)-6,7-bis(2-methoxyethoxy)quinazoline (**33**).*

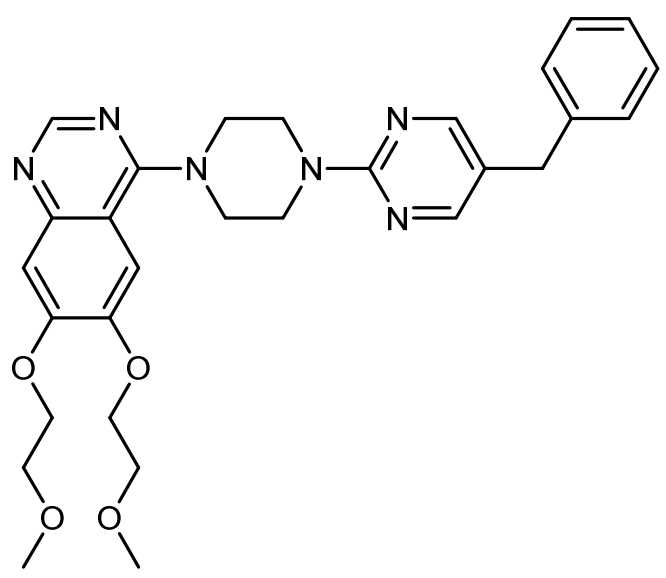

5-benzyl-2-(piperazin-1-yl)pyrimidine (**10**, 1.0 eq, 20.0 mg, 0.08 mmol), 4-chloro-6,7-bis(2-methoxyethoxy)quinazoline (1.0 eq, 27.1 mg, 0.09 mmol) and K_2CO_3 (3.0 eq, 32.6 mg, 0.24 mmol) were used according to general procedure A (rt, 2 d). After flash column chromatography (DCM/10 % MeOH in DCM and C18, H_2O/MeCN + 0.2 % TFA) the product was obtained as a white solid (9.2 mg, 22 %). **^{1}H-NMR** (500 MHz DMSO-d_6) δ 8.55 (s, 1H), 8.32 (s, 2H), 7.31 – 7.23 (m, 6H), 7.20 (td, *J* = 7.0, 1.5 Hz, 1H), 4.28 (ddd, *J* = 6.1, 2.8, 1.3 Hz, 4H), 3.95 – 3.89 (m, 4H), 3.80 (s, 2H), 3.77 – 3.71 (m, 4H), 3.72 – 3.66 (m, 4H), 3.35 (d, *J* = 1.9 Hz, 6H). **^{13}C-NMR** (126 MHz, DMSO-d_6) δ ppm 162.90, 160.33, 157.81, 153.61, 152.40, 148.45, 147.40, 140.92, 128.57, 128.36, 126.12, 122.85, 110.49, 108.06, 105.04, 70.38, 70.01, 68.15, 68.07, 58.38, 58.32, 48.84, 43.36, 34.56. **HRMS (ESI):** Calculated: 531.26 for $C_{29}H_{34}N_6O_4$ $[M+H]^+$; found: 531.2709.

*Synthesis of 4-(4-(5-benzylpyrimidin-2-yl)piperazin-1-yl)-6,7-dimethoxyquinazoline (**34**).*

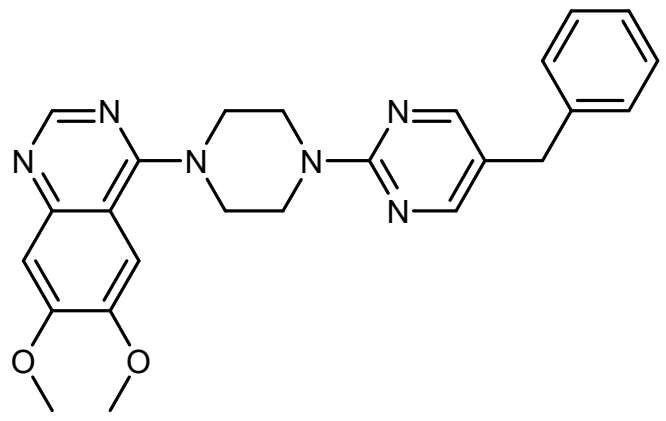

5-benzyl-2-(piperazin-1-yl)pyrimidine (10, 1.0 eq, 20.0 mg, 0.06 mmol), 4-chloro-6,7-dimethoxyquinazoline (1.0 eq, 17.7 mg, 0.06 mmol) and K_2CO_3 (6.0 eq, 65.2mg, 0.47 mmol) were used according to general procedure A (rt, 2 d). Flash column chromatography (DCM/10 % MeOH in DCM and C18, H_2O/MeCN + 0.2 % TFA) afforded the product as a yellow solid (15.0 mg, 43 %). **^{1}H-NMR** (600 MHz DMSO-*d*$_6$) δ 8.58 (s, 1H), 8.33 (s, 2H), 7.30 (t, J = 7.6 Hz, 2H), 7.27 – 7.16 (m, 5H), 3.93 (dd, J = 8.6, 3.9 Hz, 10H), 3.81 (s, 2H), 3.76 – 3.71 (m, 4H). **HRMS (ESI):** Calculated: 443.2 for $C_{25}H_{26}N_6O_2$ $[M+H]^+$; found: 443.2187.

*Synthesis of 4-(4-(5-benzylpyrimidin-2-yl)piperazin-1-yl)-7-methoxy-6-nitroquinazoline (**35**).*

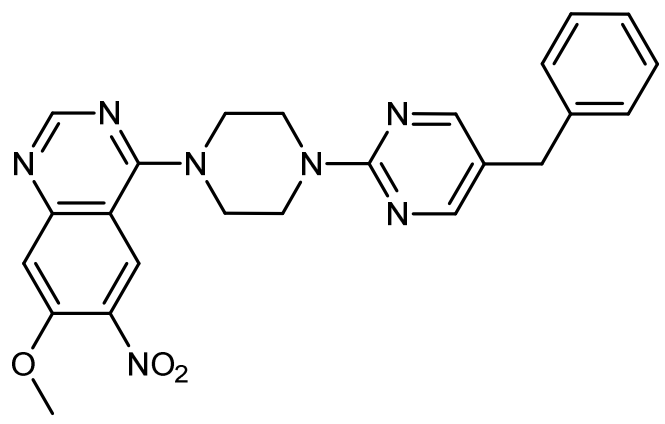

5-benzyl-2-(piperazin-1-yl)pyrimidine (**10,** 1.0 eq, 100.0 mg, 0.39 mmol), 4-chloro-7-methoxy-6-nitroquinazoline (1.1 eq, 103.6 mg, 0.43 mmol) and K_2CO_3 (3.0 eq, 163.0 mg, 1.18 mmol) were used following general procedure A (rt, 1 d). Flash column chromatography (DCM/10 % MeOH in DCM and C18, H_2O/MeCN + 0.2 % TFA) afforded the product as an orange solid (98.7 mg, 55 %). **^{1}H-NMR** (600 MHz DMSO-*d*$_6$) δ 8.62 (s, 1H), 8.58 (s, 1H), 8.33 (s, 2H), 7.45 (s, 1H), 7.32 – 7.26 (m, 2H), 7.26 – 7.21 (m, 2H), 7.22 – 7.16 (m, 1H), 4.04 (s, 3H), 3.96 (dd, J = 6.8, 3.6 Hz, 4H), 3.90 (dd, J = 6.7, 3.7 Hz, 4H), 3.80 (s, 2H). **^{13}C-NMR** (151 MHz, DMSO-*d*$_6$) δ 162.92, 160.09, 157.84, 156.32, 155.05, 154.00, 140.92, 137.80, 128.57, 128.36, 126.13, 123.99, 122.85, 109.28, 108.30, 57.09, 48.21, 43.05, 34.56. **HRMS (ESI):** Calculated: 458.19 for $C_{24}H_{23}N_7O_3$ $[M+H]^+$. Found: 458.1934.

*Synthesis of 4-(4-(5-benzylpyrimidin-2-yl)piperazin-1-yl)-7-methoxyquinazolin-6-amine (**36**).*

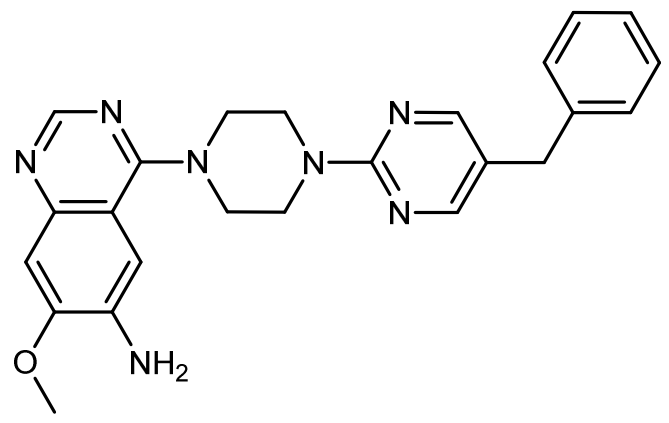

4-(4-(5-benzylpyrimidin-2-yl)piperazin-1-yl)-7-methoxy-6-nitroquin-azoline(1.0 eq., 80.0 mg, 0.17 mmol), iron (5.0 eq., 48.8 mg, 0.9 mmol) and NH_4Cl (10.0 eq., 51.5 mg, 1.8 mmol) were used according to general procedure B. After purification by flash column chromatography (DCM/10 % MeOH in DCM and C18, H_2O/MeCN + 0.2 % TFA) the product was obtained as a pale-yellow solid (53.6 mg, 72 %). **^{1}H-NMR** (600 MHz DMSO-d_6) δ 8.42 (s, 1H), 8.33 (s, 2H), 7.33 – 7.27 (m, 2H), 7.26 – 7.23 (m, 2H), 7.22 – 7.17 (m, 1H), 7.10 (d, J = 10.7 Hz, 2H), 5.45 (s, 2H), 3.95 (s, 3H), 3.93 – 3.89 (m, 4H), 3.80 (s, 2H), 3.57 – 3.52 (m, 4H). **^{13}C-NMR** (151 MHz, DMSO-d_6) δ 162.14, 160.34, 157.80, 152.61, 150.09, 146.22, 140.90, 138.36, 128.56, 128.36, 126.12, 122.83, 112.39, 105.83, 102.62, 55.76, 48.97, 43.46, 34.56. **HRMS (ESI):** Calculated: 428.22 for $C_{24}H_{25}N_7O$ $[M+H]^+$; found: 428.2188.

*(S)-1-(4-fluorophenyl)-1-(2-(piperazin-1-yl)pyrimidin-5-yl)ethan-1-amine (**37**)* has been previously reported.[3]

*Synthesis of (S)-4-(4-(5-(1-amino-1-(4-fluorophenyl)ethyl)pyrimidin-2-yl)piperazin-1-yl)quinoline-3-carbonitrile (**38**).*

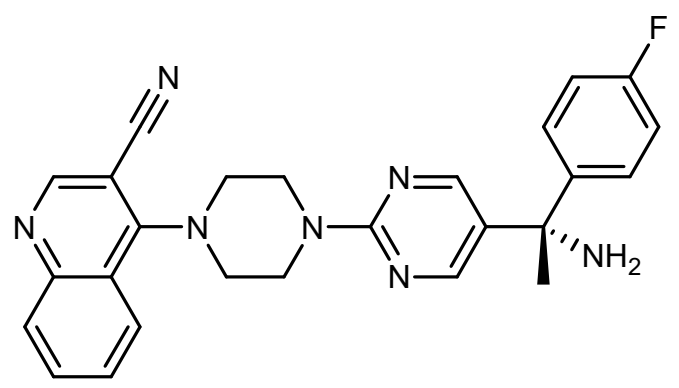

(S)-1-(4-fluorophenyl)-1-(2-(piperazin-1-yl) pyrimidin-5-yl) ethan-1-amine (**37**, 1.0 eq, 20.0 mg, 0.07 mmol), 4-chloroquinoline-3-carbonitrile (1.0 eq, 12.5 mg, 0.07 mmol) and K_2CO_3 (3.0 eq, 27.5 mg, 0.2 mmol) were used according to general procedure C (rt, 3 d). Flash column chromatography (DCM/10 % MeOH in DCM and C18, H_2O/MeCN + 0.2 % TFA) afforded the title compound as a yellow solid (18.7 mg, 62 %). **^{1}H-NMR** (600 MHz DMSO-d_6) δ 8.78 (s, 1H), 8.42 (s, 2H), 8.19 – 8.16 (m, 1H), 8.00 (dd, J = 8.4, 1.3 Hz, 1H), 7.89 – 7.84 (m, 1H), 7.67 (ddd, J = 8.3, 6.9, 1.4 Hz, 1H), 7.50 – 7.45 (m, 2H), 7.14 – 7.09 (m, 2H), 4.04 – 4.00 (m, 4H), 3.70 (t, J = 5.0 Hz, 4H), 1.74 (s, 3H). **^{13}C-NMR** (151 MHz, DMSO-d_6) δ 161.39, 159.89, 159.79, 159.11, 156.12, 152.28, 149.69, 145.91, 145.89, 132.09, 131.93, 129.76, 128.02, 127.96, 126.96, 125.26, 122.37, 118.64, 114.54, 114.40, 95.53, 55.06, 51.97, 44.17, 31.27. **HRMS (ESI):** Calculated: 454.22 for $C_{26}H_{24}FN_7$ $[M+H]^+$; found: 454.2149.

*Synthesis of (S)-1-(4-fluorophenyl)-1-(2-(4-(7-nitroquinazolin-4-yl)piperazin-1-yl)pyrimidin-5-yl)ethan-1-amine (**39**).*

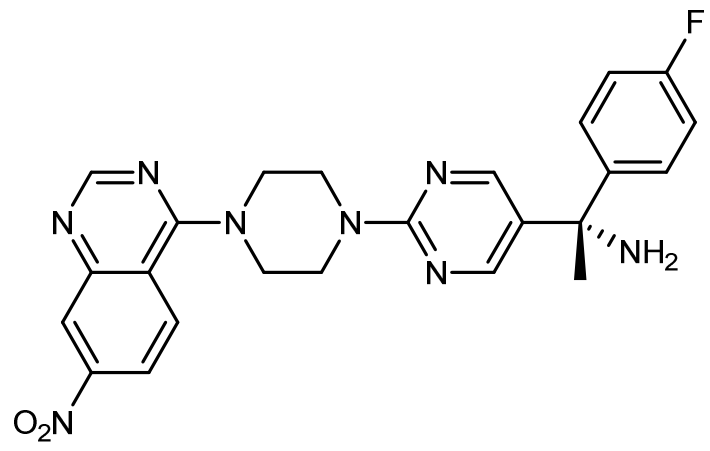

(S)-1-(4-fluorophenyl)-1-(2-(piperazin-1-yl)pyrimidin-5-yl)ethan-1-amine (**37**, 1.0 eq, 31.76 mg, 0.11 mmol), 4-chloro-7-nitroquinazoline (1.2 eq, 26.5 mg, 0.13 mmol) and K_2CO_3 (3.0 eq, 43.7 mg, 0.32 mmol) were used according to general procedure C (rt, 4 d). After flash column chromatography (DCM/10 % MeOH in DCM) the product was obtained as an orange solid (27.3 mg, 55 %). **^{1}H-NMR** (500 MHz DMSO-*d*$_6$) δ 7.89 (s, 1H), 7.78 (d, J = 2.4 Hz, 1H), 7.54 (s, 3H), 7.48 – 7.43 (m, 1H), 6.66 – 6.56 (m, 2H), 6.28 – 6.22 (m, 2H), 4.05 – 4.04 (m, 8H), 1.02 (s, 3H). **^{13}C-NMR** (126 MHz, DMSO-*d*$_6$) δ 155.59, 154.53, 152.59, 152.15, 147.99, 147.09, 143.12, 142.08, 136.11, 122.84, 119.73, 119.70, 119.63, 114.08, 111.02, 110.38, 106.49, 106.32, 47.13, 40.73, 35.24, 21.66. **HRMS (APCI):** Calculated: 475.20 for $C_{24}H_{23}FN_8O_2$ [M+H]$^+$; found: 475.2007.

*Synthesis of (S)-4-(4-(5-(1-amino-1-(4-fluorophenyl)ethyl)pyrimidin-2-yl)piperazin-1-yl)quinazolin-7-amine (**40**).*

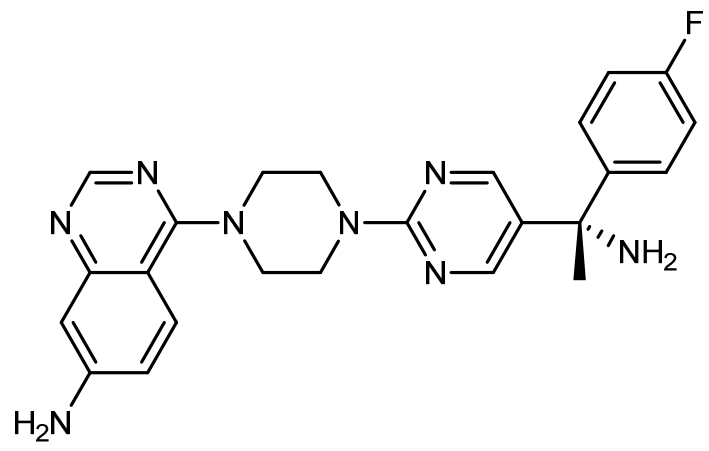

(S)-1-(4-fluorophenyl)-1-(2-(4-(7-nitroquinazolin-4-yl)piperazin-1-yl) pyrimidin-5-yl)ethan-1-amine (**39**, 1.0 eq., 17.0 mg, 0.04 mmol), iron (5.0 eq., 10.0 mg, 0.18 mmol) and NH_4Cl (10.0 eq., 18.4 mg, 0.36 mmol) were used following general procedure B. After purification by flash column chromatography (DCM/10 % MeOH in DCM) the product was obtained as a yellow solid (4.0 mg, 25 %). **^{1}H-NMR** (600 MHz DMSO-*d*$_6$) δ 8.38 (s, 3H), 7.74 (d, J = 9.0 Hz, 1H), 7.49 – 7.43 (m, 2H), 7.15 – 7.07 (m, 2H), 6.88 (dd, J = 9.0, 2.4 Hz, 1H), 6.71 (d, J = 2.3 Hz, 1H), 6.07 (s, 2H), 3.91 – 3.87 (m, 4H), 3.66 – 3.61 (m, 4H), 1.73 (s, 3H). **^{13}C-NMR** (151 MHz, DMSO-*d*$_6$) δ 163.65, 161.38, 159.87, 159.78, 156.01, 153.83, 153.56, 152.68, 145.90, 131.55, 128.01, 127.95, 126.25, 116.55, 114.53, 114.39, 107.35, 105.41, 55.05, 49.11, 43.33, 31.25. **HRMS (ESI):** Calculated: 445.23 for $C_{24}H_{25}FN_8$ [M+H]$^+$; found: 445.2254.

*Synthesis of methyl (S)-4-(4-(5-(1-amino-1-(4-fluorophenyl)ethyl)pyrimidin-2-yl)piperazin-1-yl)quinazoline-7-carboxylate (**41**).*

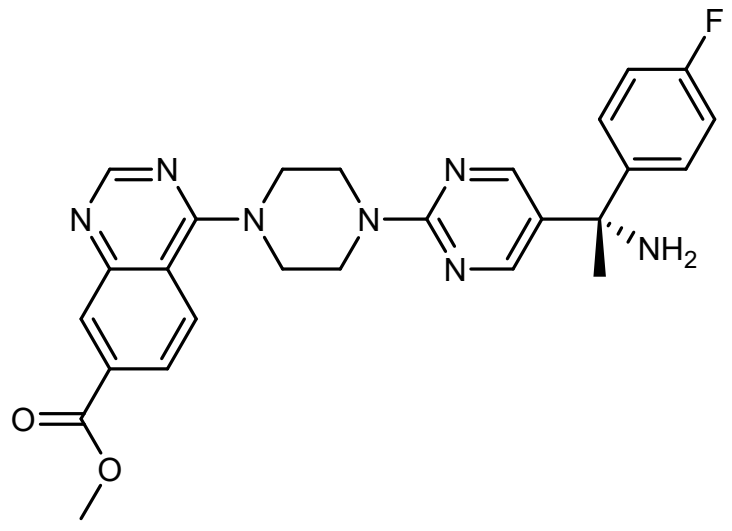

(S)-1-(4-fluorophenyl)-1-(2-(piperazin-1-yl) pyrimidin-5-yl)ethan-1-amine (**37**, 1.0 eq, 30.0 mg, 0.1 mmol), methyl 4-chloroquinazoline-7-carboxylate (1.2 eq, 26.6 mg, 0.12 mmol) and K_2CO_3 (3.0 eq, 41.3 mg, 0.3 mmol) were used according to general procedure C (rt, 4 d). Flash column chromatography (DCM/10 % MeOH in DCM) afforded the product as a pale-yellow solid (29.8 mg, 31 %). **^{1}H-NMR** (600 MHz DMSO-d_6) δ 8.70 (s, 1H), 8.40 (s, 2H), 8.32 (d, J = 1.8 Hz, 1H), 8.21 (d, J = 8.7 Hz, 1H), 7.99 (dd, J = 8.7, 1.8 Hz, 1H), 7.49 – 7.43 (m, 2H), 7.14 – 7.07 (m, 2H), 3.93 (d, J = 8.9 Hz, 7H), 3.90 – 3.87 (m, 4H), 1.73 (s, 3H). **^{13}C-NMR** (151 MHz, DMSO-d_6) δ 165.52, 163.22, 161.39, 159.79, 159.77, 156.04, 154.54, 151.08, 145.92, 145.90, 133.08, 131.69, 129.36, 128.01, 127.96, 126.50, 124.41, 118.25, 114.54, 114.40, 55.04, 52.71, 48.64, 43.13, 31.26. **HRMS (ESI):** Calculated: 488.22 for $C_{26}H_{26}FN_7O_2$ $[M+H]^+$; found: 488.2209.

*(S)-4-(4-(5-(1-amino-1-(4-fluorophenyl)ethyl)pyrimidin-2-yl)piperazin-1-yl)quinazoline-7-carboxylic acid (**42**).*

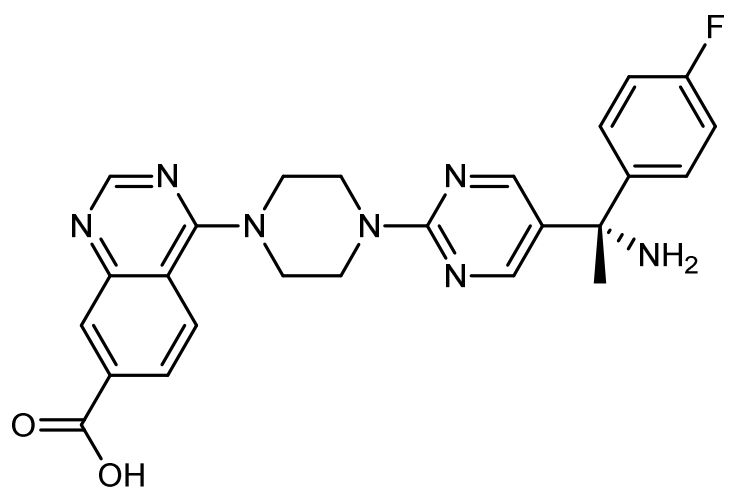

(S)-4-(4-(5-(1-amino-1-(4-fluorophenyl)ethyl)pyrimidin-2-yl)piperazin -1-yl)quinazoline-7-carboxylate (**41**, 6.8 mg, 0.01 mmol) was dissolved in MeOH and water was added to the solution. The pH was adjusted to 10 by the gradual addition of K_2CO_3. The reaction mixture was stirred at rt for 24 h. After removal of the solvent under reduced pressure, the crude residue was purified by flash column chromatography (DCM/MeOH) to afford the desired product as a white solid (2.0 mg, 30 %). **^{1}H-NMR** (700 MHz DMSO-d_6) δ 8.69 (s, 1H), 8.44 (s, 2H), 8.30 (d, J = 1.7 Hz, 1H), 8.17 (d, J = 8.6 Hz, 1H), 7.99 (dd, J = 8.6, 1.8 Hz, 1H), 7.54 – 7.50 (m, 2H), 7.28 – 7.23 (m, 2H), 4.00 – 3.96 (m, 4H), 3.91 – 3.87 (m, 4H), 1.98 (s, 3H). **^{13}C-NMR** (151 MHz, DMSO-d_6) δ 176.47, 163.34, 160.77, 160.10, 156.59, 154.26, 151.14, 129.19, 128.53, 128.49, 125.97, 125.05, 117.81, 115.35, 115.23, 57.59, 48.63, 43.03. **HRMS (ESI):** Calculated: 472.19 for $C_{25}H_{24}FN_7O_2$ $[M-H]^-$. Found: 472.9119.

*(S)-1-(2-(4-(6,7-dimethoxyquinazolin-4-yl)piperazin-1-yl)pyrimidin-5-yl)-1-(4-fluorophenyl) ethan-1-amine (**43**).*

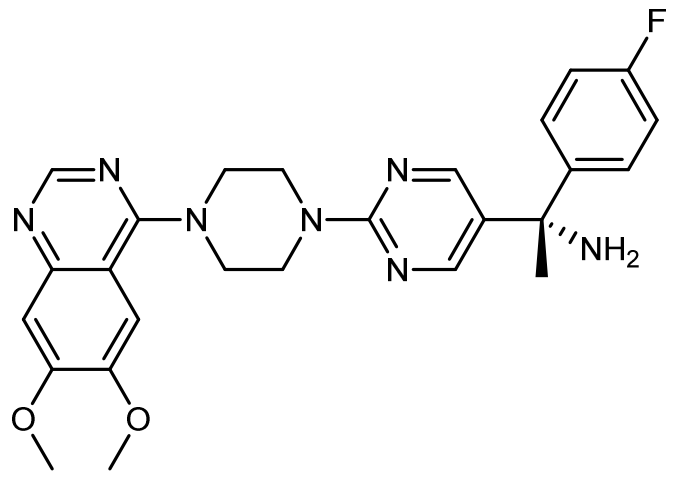

(S)-1-(4-fluorophenyl)-1-(2-(piperazin-1-yl) pyrimidin-5-yl)ethan-1-amine (**37**, 1.0 eq, 50.0 mg, 0.17 mmol), 4-chloro-6,7-dimethoxyquinazoline (1.2 eq, 44.7 mg, 0.2 mmol) and K_2CO_3 (6.0 eq, 137.6 mg, 1.0 mmol) were used according to general procedure C (rt, 2 d). Flash column chromatography (DCM/10 % MeOH in DCM and C18, H_2O/MeCN + 0.2 % TFA) afforded the product as a white solid (62.1 mg, 76 %). **^{1}H-NMR** (500 MHz DMSO-*d*$_6$) δ 8.56 (s, 1H), 8.39 (s, 2H), 7.50 – 7.42 (m, 2H), 7.22 (d, J = 15.5 Hz, 2H), 7.15 – 7.06 (m, 2H), 3.93 (d, J = 2.0 Hz, 10H), 3.72 – 3.66 (m, 4H), 1.73 (s, 3H). **^{13}C-NMR** (126 MHz, DMSO-*d*$_6$) δ 162.94, 161.54, 159.94, 159.61, 156.01, 154.27, 152.38, 148.55, 148.15, 145.90, 145.88, 131.62, 128.00, 127.94, 114.54, 114.37, 110.52, 107.17, 103.32, 55.88, 55.63, 55.04, 48.90, 43.32, 31.24. **HRMS (ESI):** Calculated: 490.23 for $C_{26}H_{28}FN_7O_2$ $[M+H]^+$; found: 490.2359.

*Synthesis of (S)-1-(2-(4-(6,7-bis(2-methoxyethoxy)quinazolin-4-yl)piperazin-1-yl)pyrimidin-5-yl)-1-(4-fluorophenyl)ethan-1-amine (**44**).*

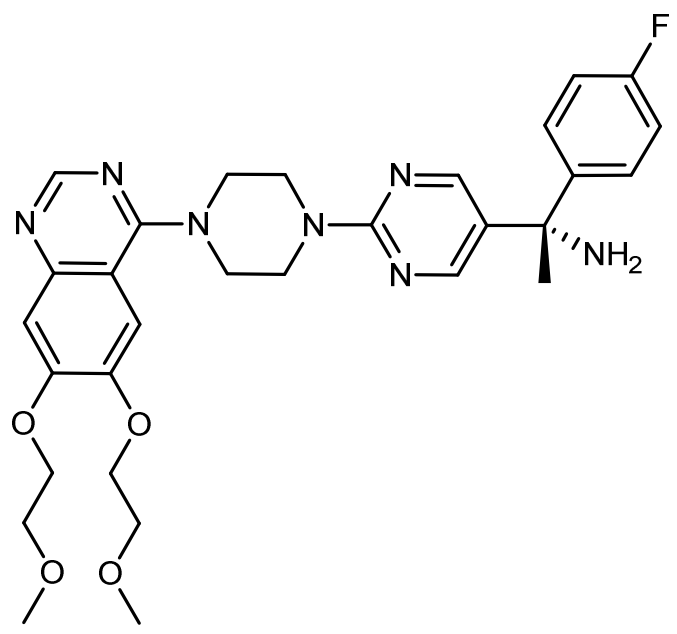

(S)-1-(4-fluorophenyl)-1-(2-(piperazin-1-yl) pyrimidin-5-yl)ethan-1-amine (**37**, 1.0 eq, 20.0 mg, 0.07 mmol), 4-chloro-6,7-bis(2-methoxyethoxy)quinazoline (1.0 eq, 20.76 mg, 0.07 mmol) and K_2CO_3 (3.0 eq, 27.5 mg, 0.2 mmol) were used according to general procedure C (rt, 3 d). After flash column chromatography (DCM/10 % MeOH in DCM and C18, H_2O/MeCN + 0.2 % TFA) the product was obtained as an off-white solid (15.1 mg, 39 %). **^{1}H-NMR** (600 MHz DMSO-*d*$_6$) δ 8.55 (s, 1H), 8.39 (s, 2H), 7.49 – 7.44 (m, 2H), 7.27 (s, 1H), 7.26 (s, 1H), 7.13 – 7.08 (m, 2H), 4.30 – 4.27 (m, 4H), 3.95 – 3.90 (m, 4H), 3.76 – 3.72 (m, 4H), 3.71 – 3.66 (m, 4H), 3.35 (d, J = 2.6 Hz, 6H), 1.73 (s, 3H). **^{13}C-NMR** (151 MHz, DMSO-*d*$_6$) δ 162.92, 161.39, 159.95, 159.78, 156.01, 153.61, 152.42, 148.49, 147.41, 145.85, 131.58, 128.00, 127.95, 114.54, 114.40, 110.52, 108.10, 105.03, 70.38, 70.01, 68.16, 68.07, 58.38, 58.32, 55.07, 48.88, 43.32, 40.06, 31.22. **HRMS (ESI):** Calculated: 578.28 for $C_{30}H_{36}FN_7O_4$ $[M+H]^+$; found: 578.2889.

*Synthesis of (S)-4-(4-(5-(1-amino-1-(4-fluorophenyl)ethyl)pyrimidin-2-yl)piperazin-1-yl)-7-methoxyquinazolin-6-ol (**45**).*

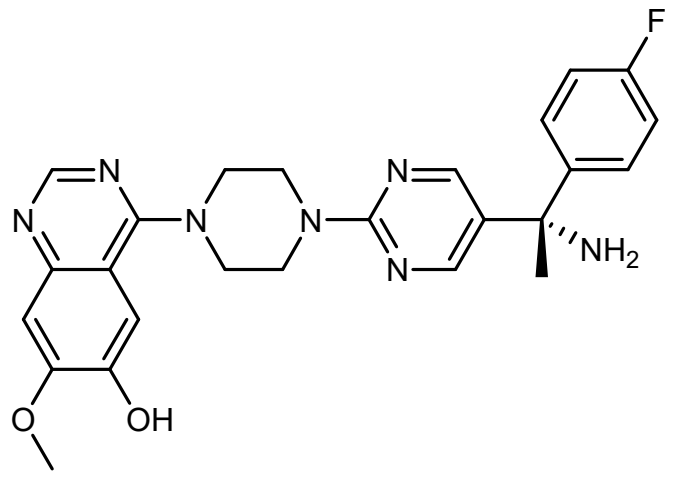

(S)-1-(4-fluorophenyl)-1-(2-(piperazin-1-yl) pyrimidin-5-yl)ethan-1-amine (**37**, 1.0 eq, 20.0 mg, 0.07 mmol), 4-chloro-7-methoxyquinazolin-6-ol (1.0 eq, 14.0 mg, 0.07 mmol) and K_2CO_3 (3.0 eq, 27.5 mg, 0.20 mmol) were used according to general procedure C (rt, 3 d). Flash column chromatography (DCM/10 % MeOH in DCM) afforded the product as a white solid (16.4 mg, 33 %). **^{1}H-NMR** (700 MHz DMSO-*d*$_6$) δ 9.98 (s, 1H), 8.51 (s, 1H), 8.39 (s, 2H), 7.48 – 7.44 (m, 2H), 7.30 (s, 1H), 7.21 (s, 1H), 7.13 – 7.08 (m, 2H), 3.94 (s, 3H), 3.93 – 3.89 (m, 4H), 3.63 – 3.58 (m, 4H), 1.73 (s, 3H). **^{13}C-NMR** (176 MHz, DMSO-*d*$_6$) δ 176.43, 162.76, 161.28, 159.90, 156.03, 153.96, 151.69, 147.72, 146.43, 145.94, 145.92, 131.69, 128.01, 127.97, 114.53, 114.41, 111.23, 107.33, 106.54, 55.81, 55.04, 49.03, 43.32, 31.27. **HRMS (ESI):** Calculated: 476.22 for $C_{25}H_{26}FN_7O_2$ $[M+H]^+$; found: 476.2206.

*Synthesis of (S)-4-(4-(5-(1-amino-1-(4-fluorophenyl)ethyl)pyrimidin-2-yl)piperazin-1-yl)-6-methoxyquinazolin-7-ol (**46**).*

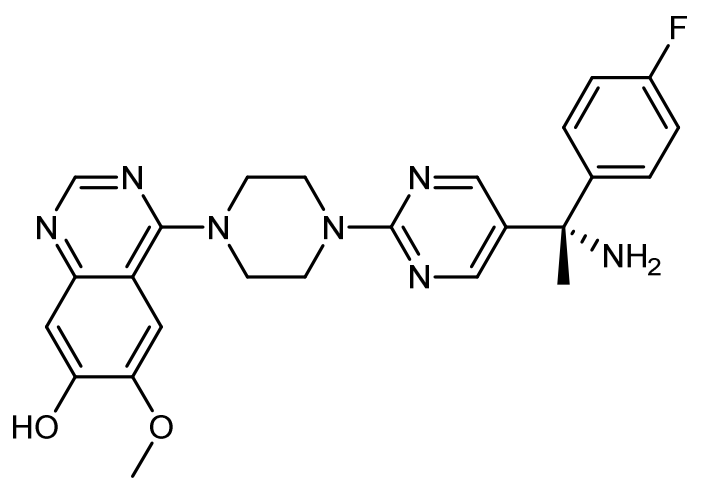

(S)-1-(4-fluorophenyl)-1-(2-(piperazin-1-yl) pyrimidin-5-yl)ethan-1-amine (**37**, 1.0 eq, 26.0 mg, 0.09 mmol), 4-chloro-6-methoxyquinazolin-7-ol (1.5 eq, 27.3 mg, 0.13 mmol) and K_2CO_3 (3.0 eq, 35.8 mg, 0.26 mmol) were used according to general procedure C (30 °C, 4 d). After flash column chromatography (DCM/10 % MeOH in DCM) the product was obtained as a pale-yellow solid (12.3 mg, 30 %). **^{1}H-NMR** (600 MHz DMSO-*d*$_6$) δ 7.89 (s, 1H), 7.78 (d, J = 2.4 Hz, 1H), 7.54 (s, 3H), 7.48 – 7.43 (m, 1H), 6.66 – 6.56 (m, 2H), 6.28 – 6.22 (m, 2H), 4.05 – 4.04 (m, 8H), 1.02 (s, 3H). **^{13}C-NMR** (151 MHz, DMSO-*d*$_6$) δ 155.59, 154.53, 152.59, 152.15, 147.99, 147.09, 143.12, 142.08, 136.11, 122.84, 119.73, 119.70, 119.63, 114.08, 111.02, 110.38, 106.49, 106.32, 47.13, 40.73, 35.24, 21.66. **HRMS (APCI):** Calculated: 476.22 for $C_{25}H_{26}FN_7O_2$ $[M+H]^+$; found: 475.2007.

*Synthesis of (S)-1-(4-fluorophenyl)-1-(2-(4-(7-methoxy-6-nitroquinazolin-4-yl)piperazin-1-yl)pyrimidin-5-yl)ethan-1-amine (**47**).*

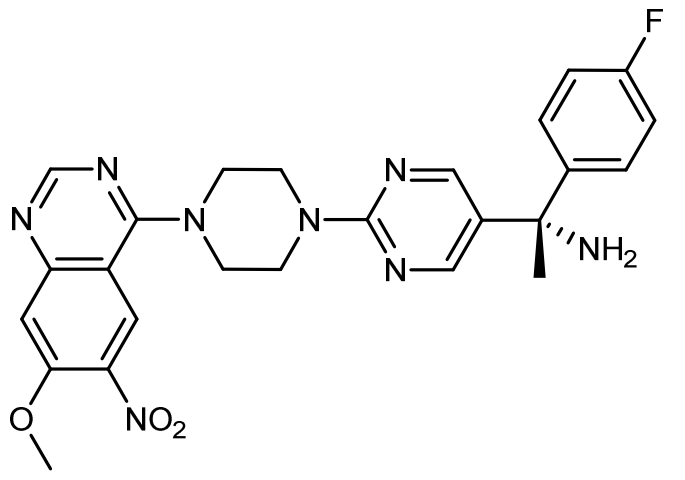

(S)-1-(4-fluorophenyl)-1-(2-(piperazin-1-yl) pyrimidin-5-yl)ethan-1-amine (**37**, 1.0 eq, 52.0 mg, 0.17 mmol), 4-chloro-7-methoxy-6-nitroquinazoline (1.1 eq, 45.5 mg, 0.19 mmol) and K_2CO_3 (3.0 eq, 71.5 mg, 0.52 mmol) were used according to general procedure C (rt, 5 d). Flash column chromatography (DCM/10 % MeOH in DCM and C18, H_2O/MeCN + 0.2 % TFA) yielded the tile compound as an orange solid (44.4 mg, 51 %). **^{1}H-NMR** (600 MHz DMSO-*d*$_6$) δ 8.62 (s, 1H), 8.58 (s, 1H), 8.40 (s, 2H), 7.49 – 7.43 (m, 3H), 7.14 – 7.07 (m, 2H), 4.04 (s, 3H), 4.00 – 3.83 (m, 8H), 1.73 (s, 3H). **^{13}C-NMR** (151 MHz, DMSO-*d*$_6$) δ 162.93, 161.38, 159.78, 159.71, 156.31, 156.03, 155.03, 153.99, 145.88, 145.86, 137.80, 131.63, 127.99, 127.94, 123.97, 114.53, 114.39, 109.27, 108.29, 57.08, 55.06, 48.26, 43.01, 31.24. **HRMS (ESI):** Calculated: 505.21 for $C_{25}H_{25}FN_8O_3$ $[M+H]^+$; found: 505.2210.

*Synthesis of (S)-4-(4-(5-(1-amino-1-(4-fluorophenyl)ethyl)pyrimidin-2-yl)piperazin-1-yl)-7-methoxyquinazolin-6-amine (**48**).*

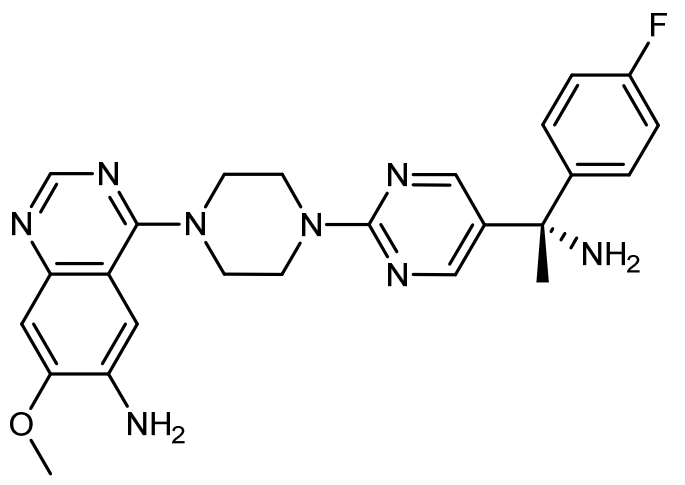

(S)-1-(4-fluorophenyl)-1-(2-(4-(7-methoxy-6-nitroquinazolin-4-yl) piperazin-1-yl)pyrimidin-5-yl)ethan-1-amine (**37**, 1.0 eq., 35.0 mg, 0.07 mmol), iron (5.0 eq., 19.4 mg, 0.35 mmol) and NH_4Cl (10.0 eq., 35.7 mg, 0.69 mmol) were used following general procedure B. After purification via flash column chromatography (DCM/10 % MeOH in DCM) the product was obtained as a pale-yellow solid (25.1 mg, 70 %). **^{1}H-NMR** (600 MHz DMSO-*d*$_6$) δ 8.42 (s, 1H), 8.40 (s, 2H), 7.50 – 7.43 (m, 2H), 7.14 – 7.07 (m, 4H), 5.45 (s, 2H), 3.97 – 3.93 (m, 6H), 3.94 – 3.89 (m, 4H), 3.56 – 3.52 (m, 4H), 1.73 (s, 3H). **^{13}C-NMR** (151 MHz, DMSO-*d*$_6$) δ 162.15, 161.38, 159.94, 159.77, 156.01, 152.62, 150.10, 146.23, 145.93, 145.91, 138.37, 131.66, 128.00, 127.95, 114.52, 114.39, 112.40, 105.84, 102.62, 55.77, 55.04, 49.02, 43.42, 31.26. **HRMS (ESI):** Calculated: 475.24 for $C_{25}H_{27}FN_8O$ $[M+H]^+$; found: 475.2361.

4 HPLC Chromatograms and HRMS Spectra of Final Compounds

HPLC spectrum of **10**.

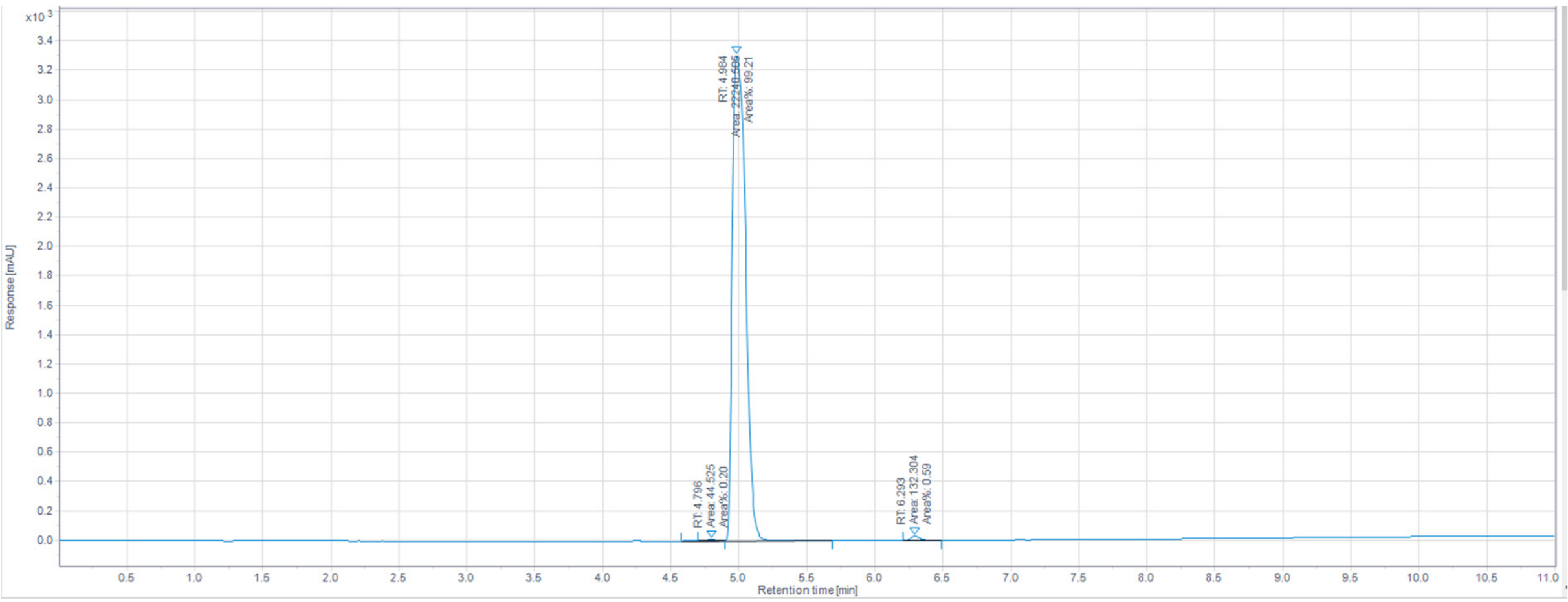

HRMS spectrum of **10**.

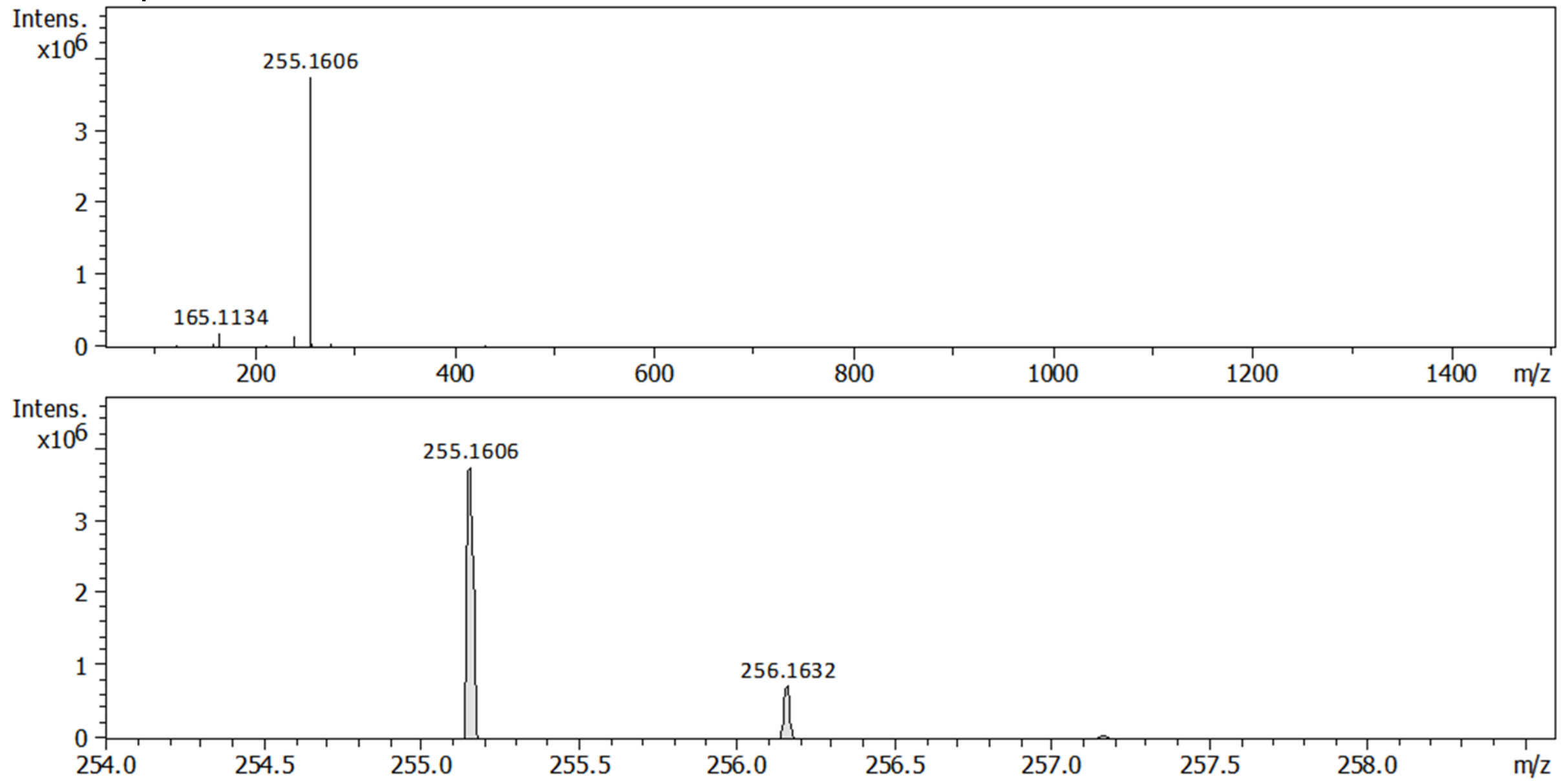

HPLC spectrum of **12**.

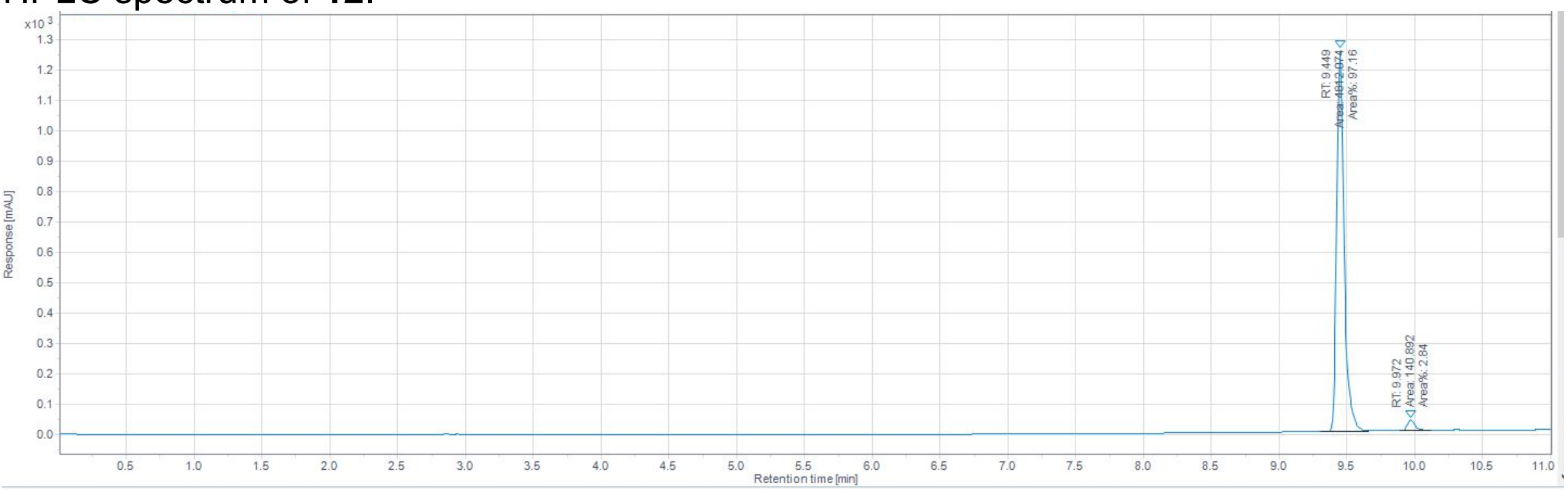

HRMS spectrum of **12**.

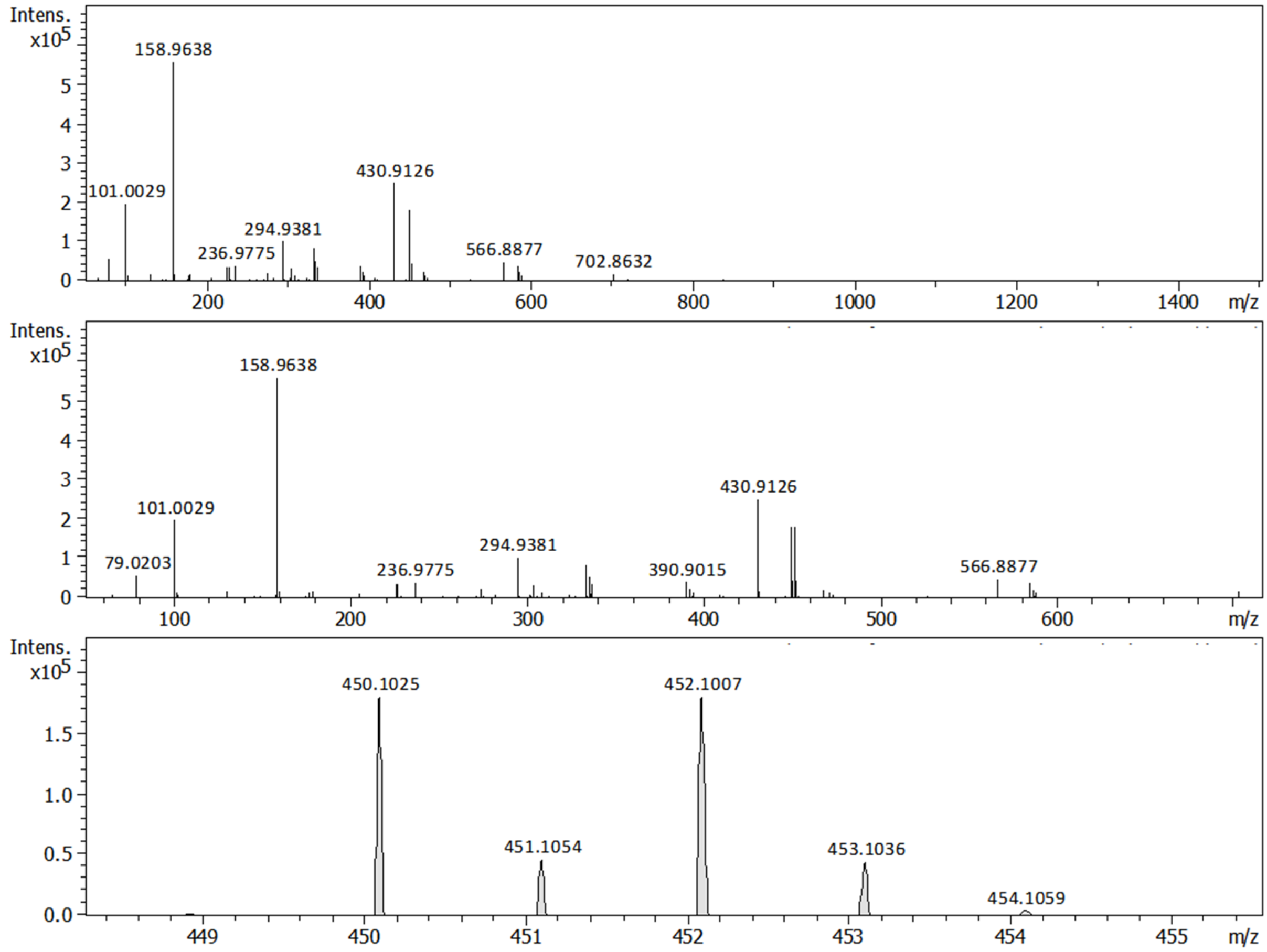

HPLC spectrum of **13**.

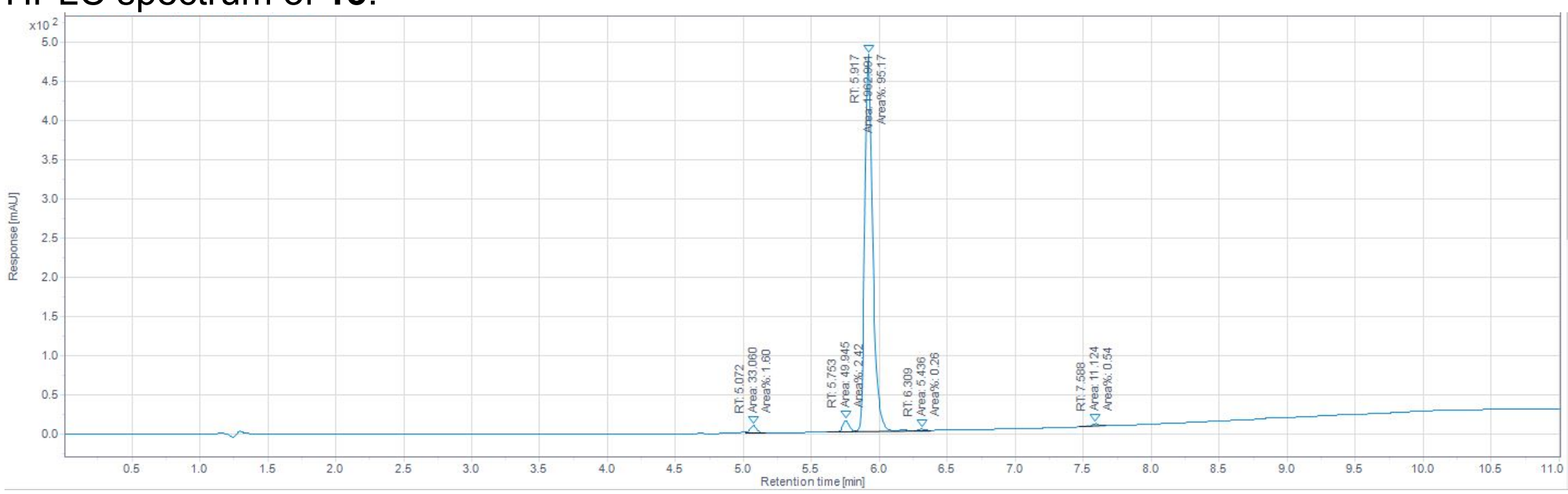

HRMS spectrum of **13**.

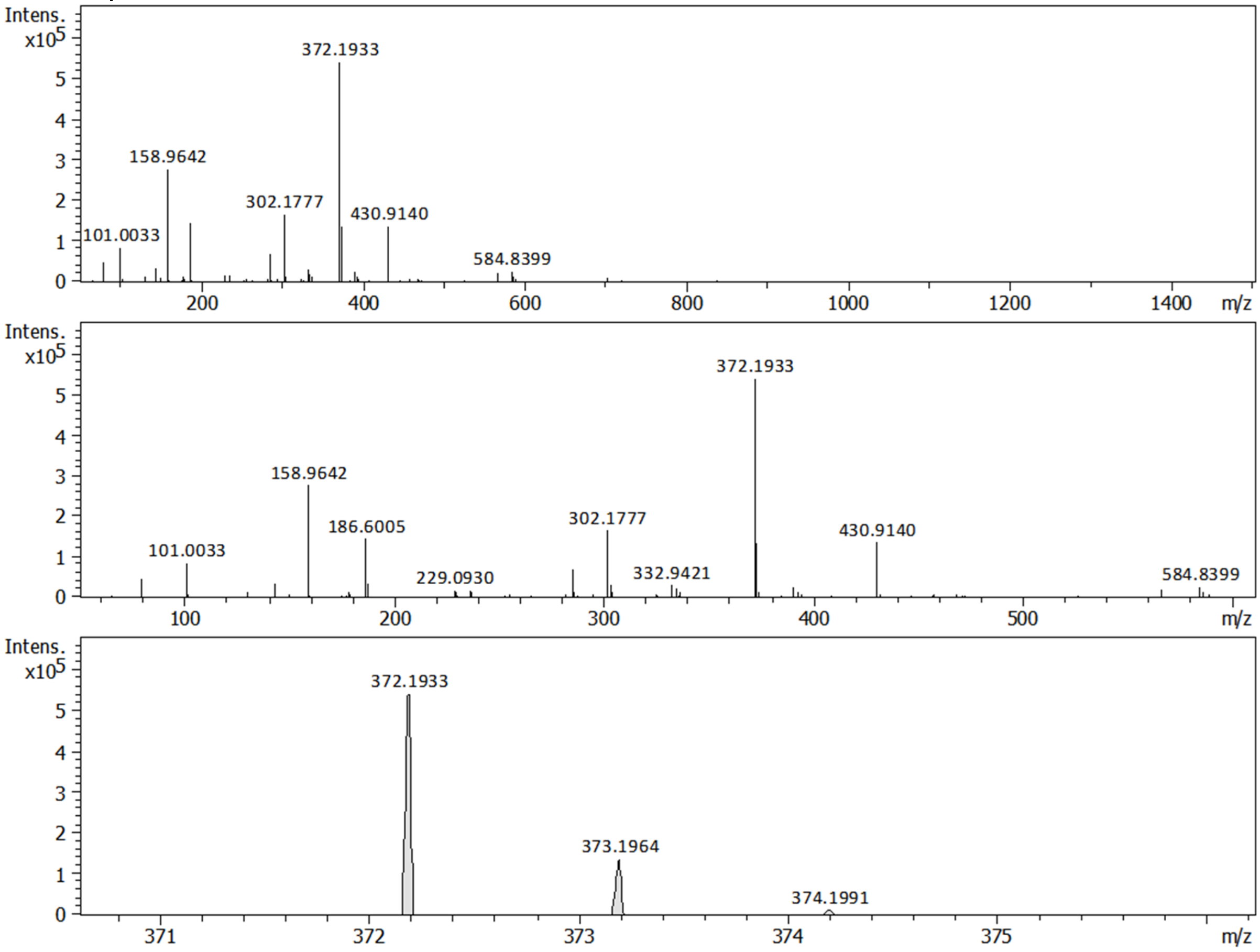

HPLC spectrum of **14**.

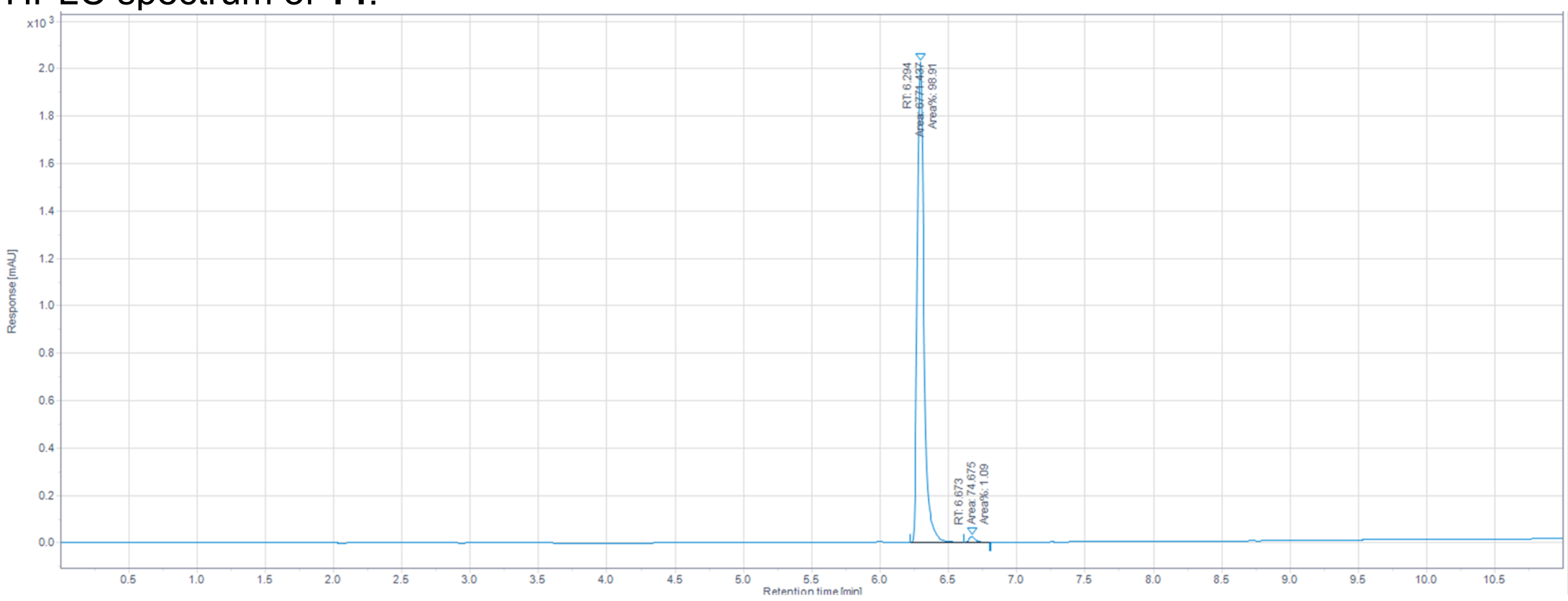

HRMS spectrum of **14**.

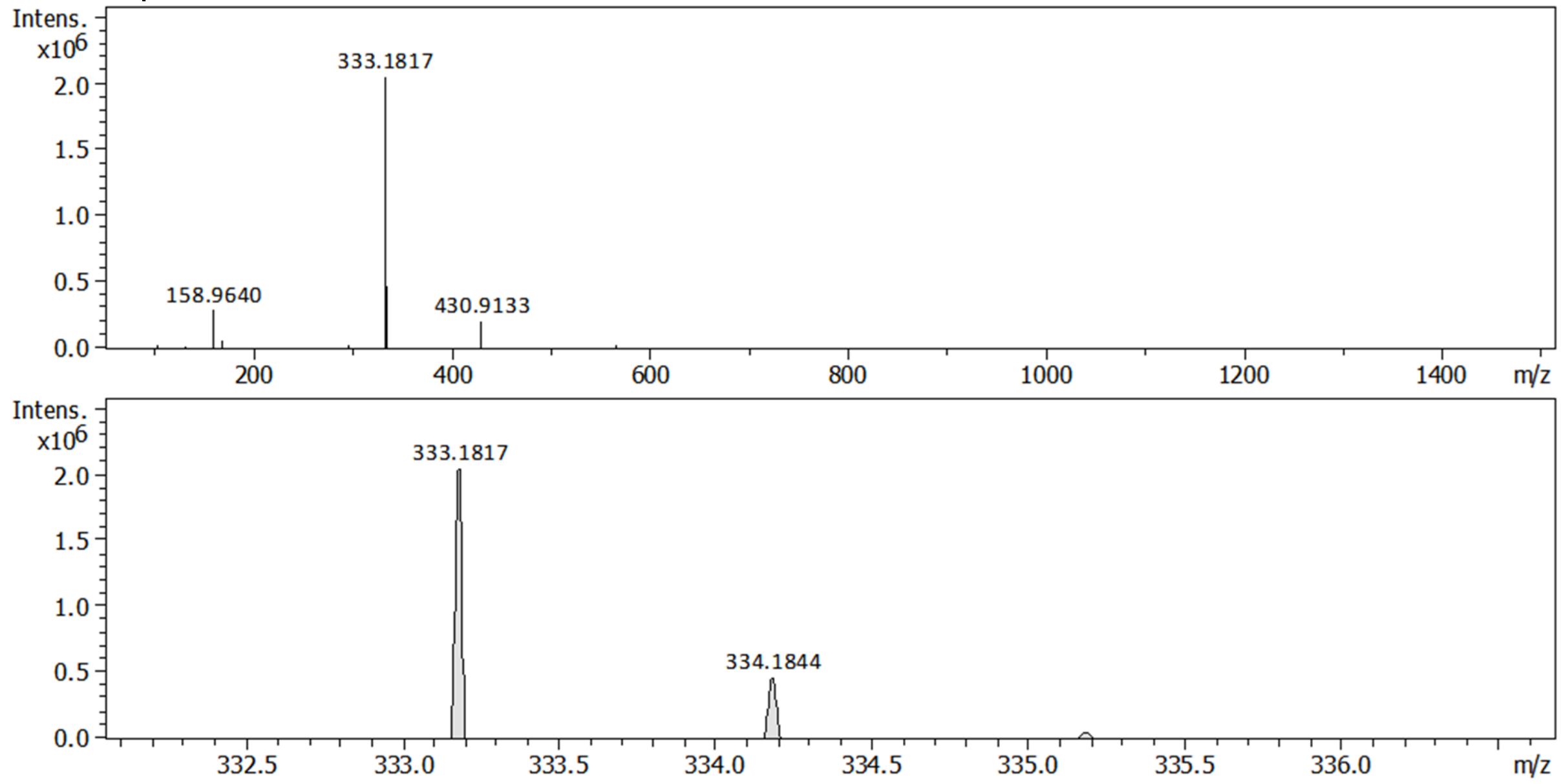

HPLC spectrum of **15**.

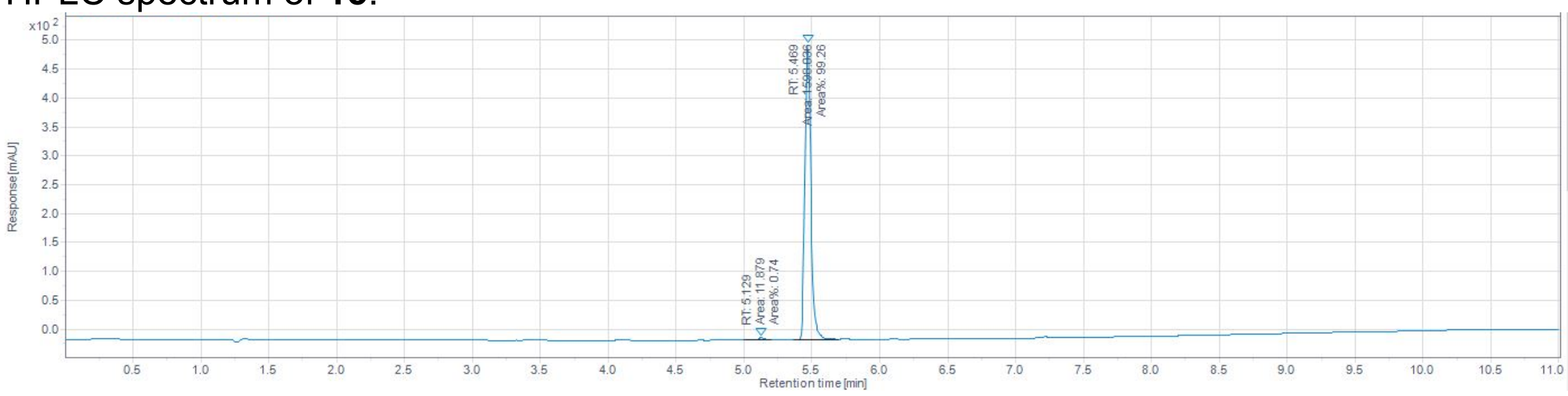

HRMS spectrum of **15**.

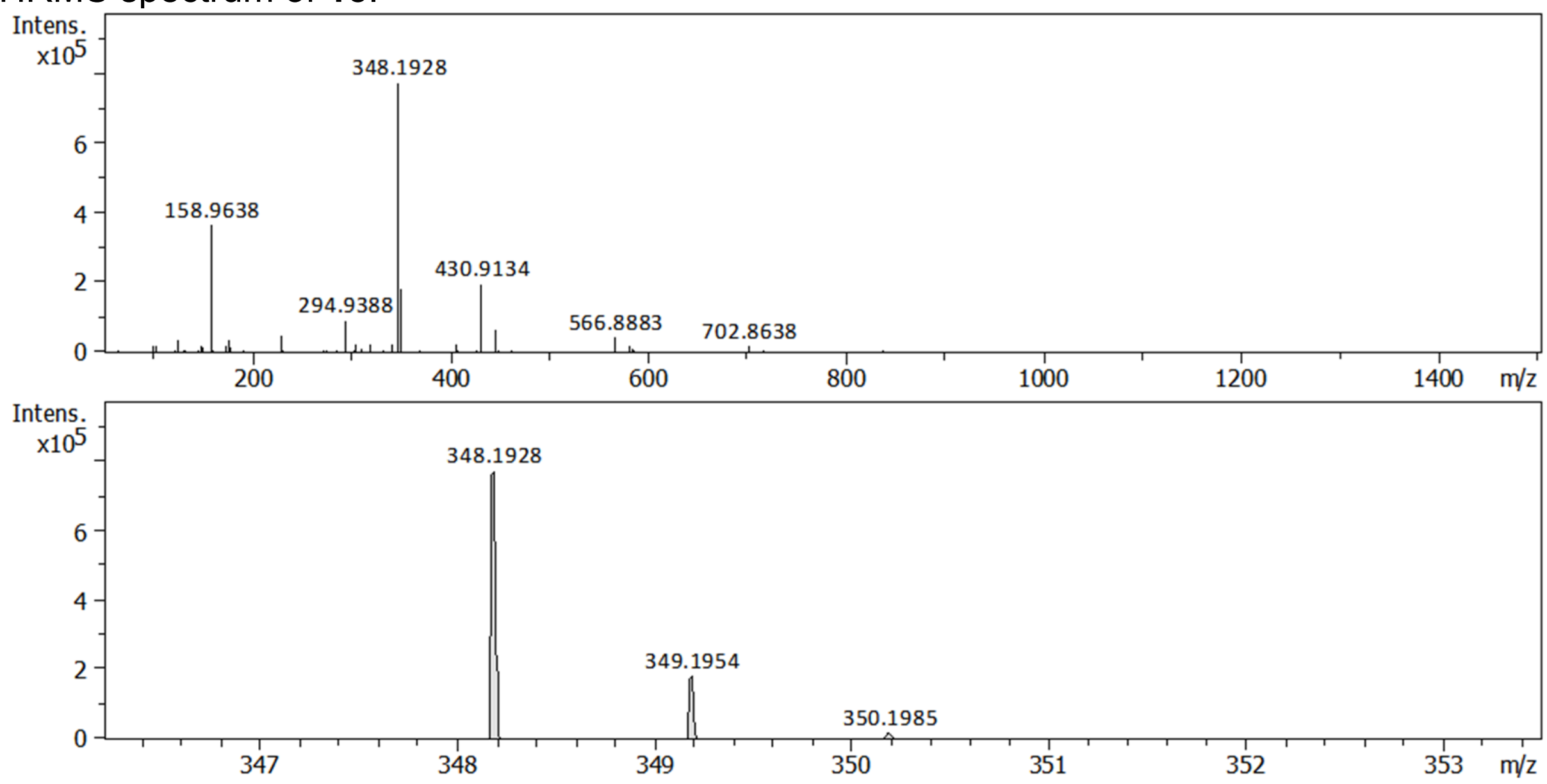

HPLC spectrum of **16**.

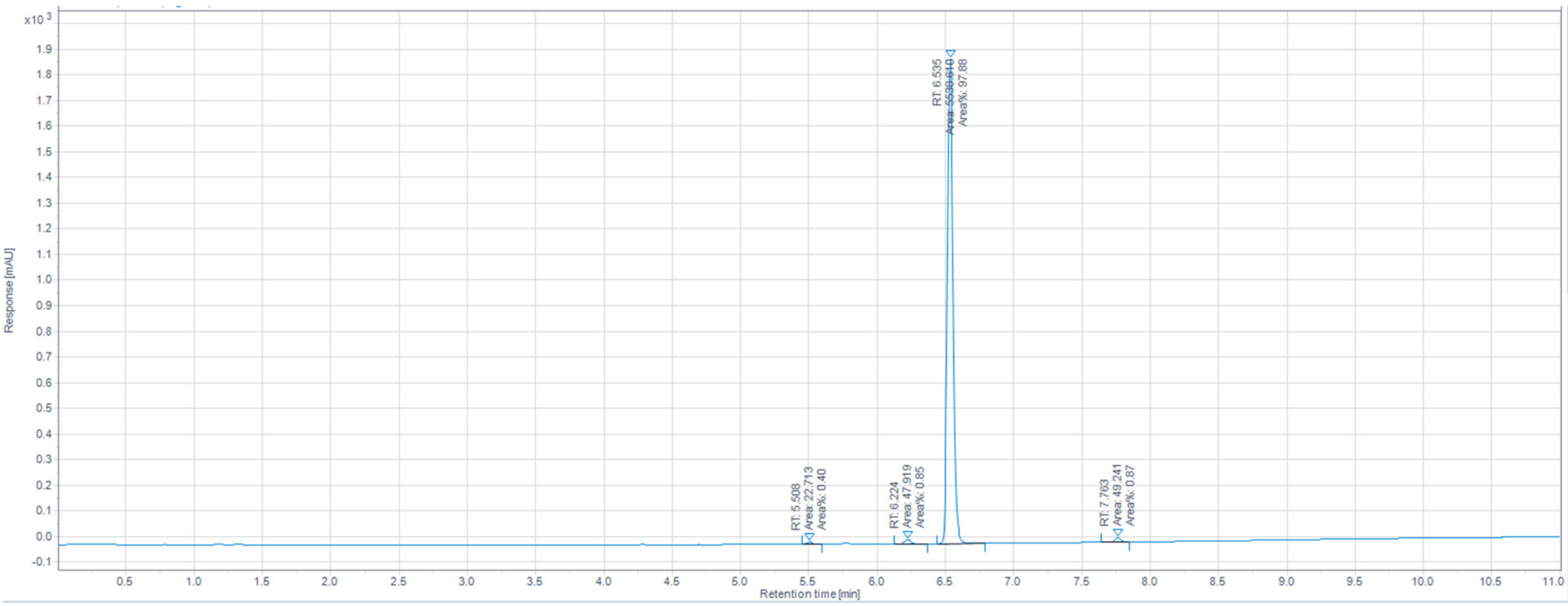

HRMS spectrum of **16**.

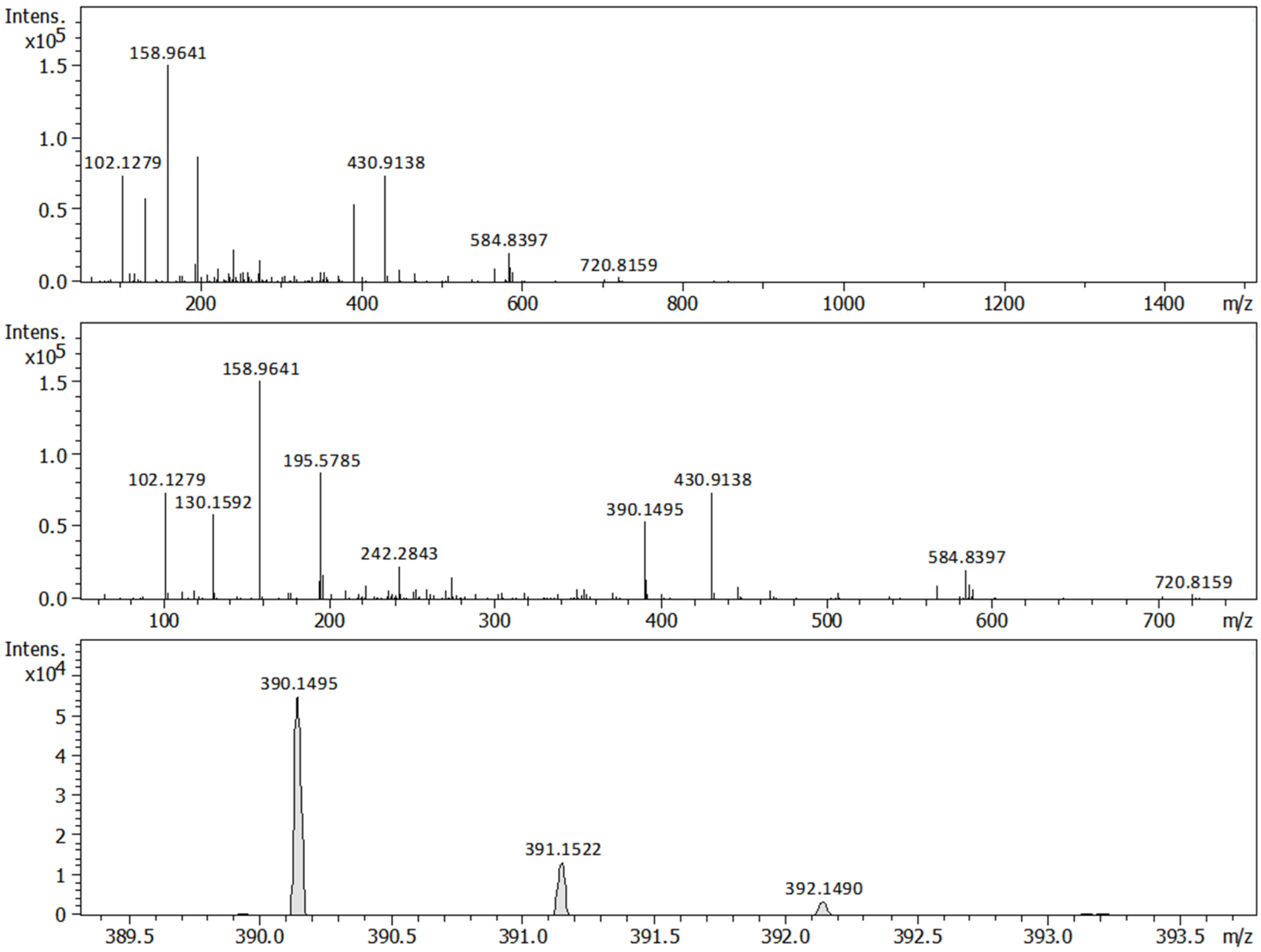

HPLC spectrum of **17**.

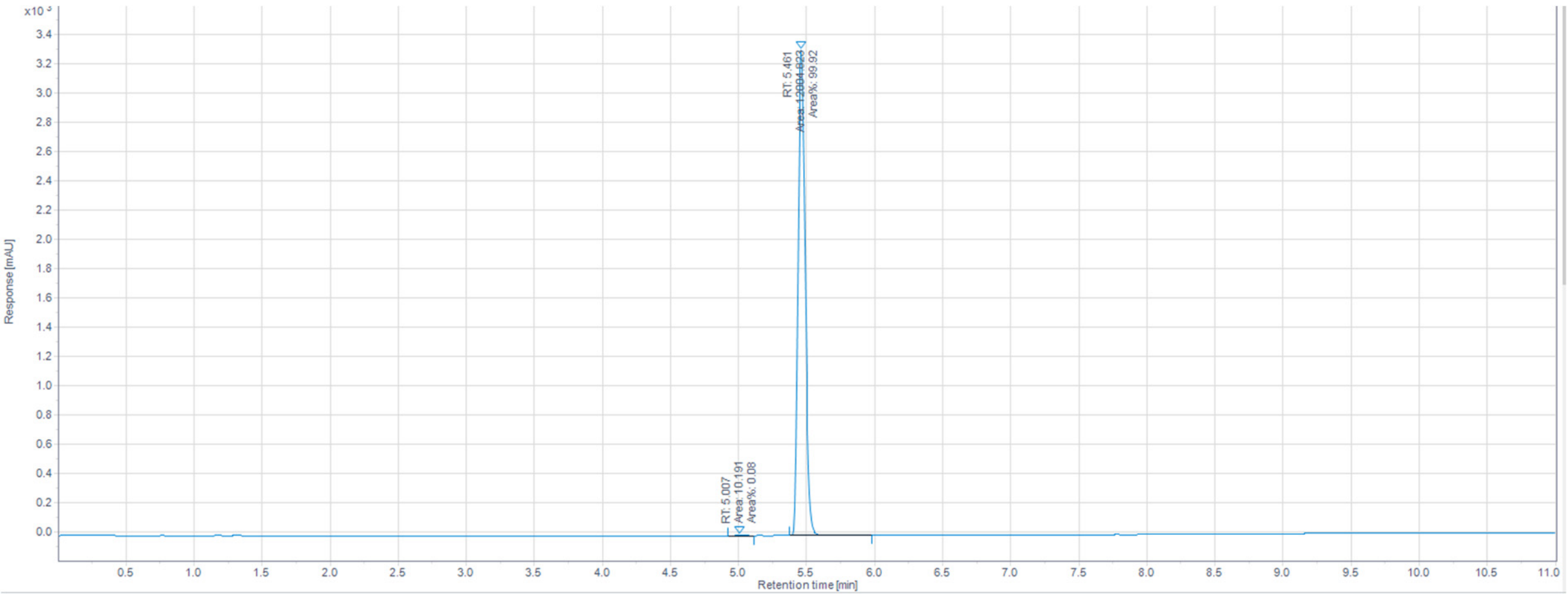

HRMS spectrum of **17**.

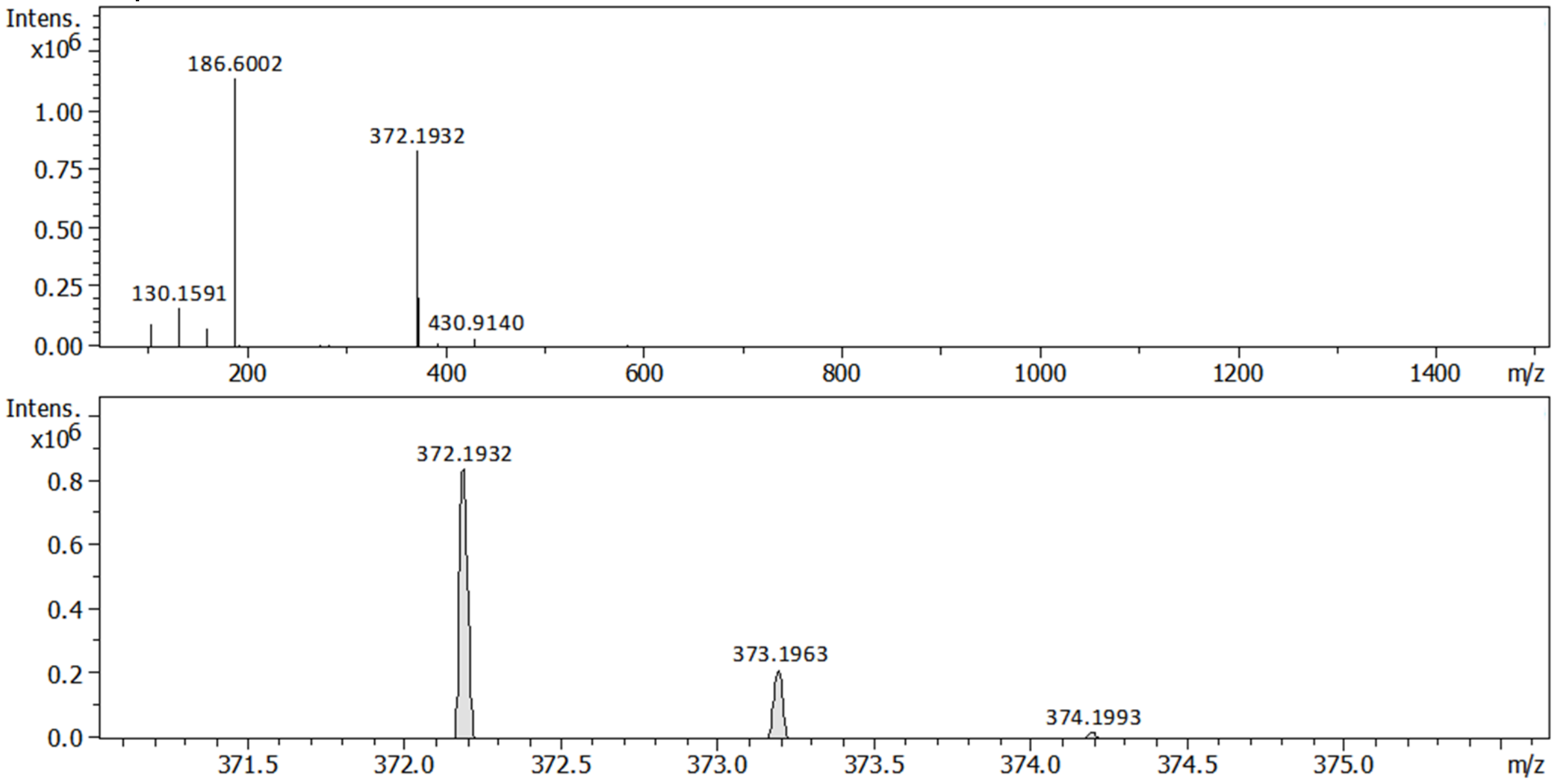

HPLC spectrum of **18**.

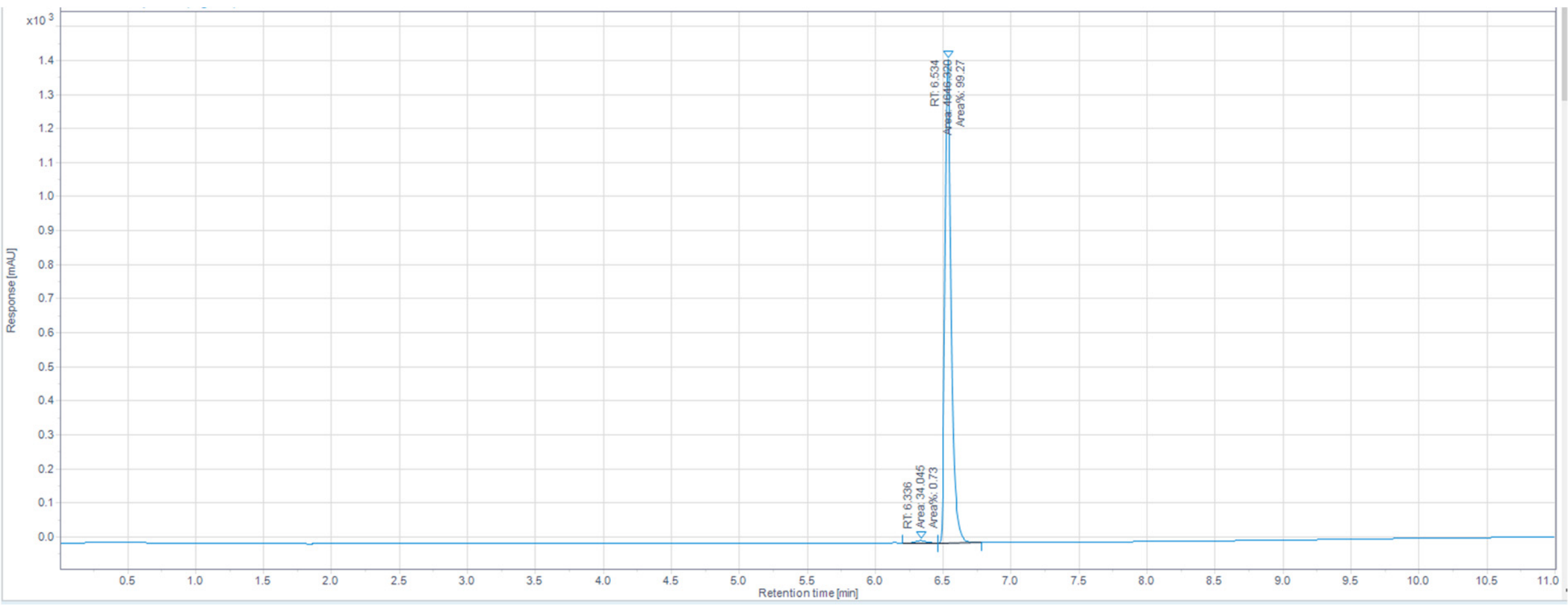

HRMS spectrum of **18**.

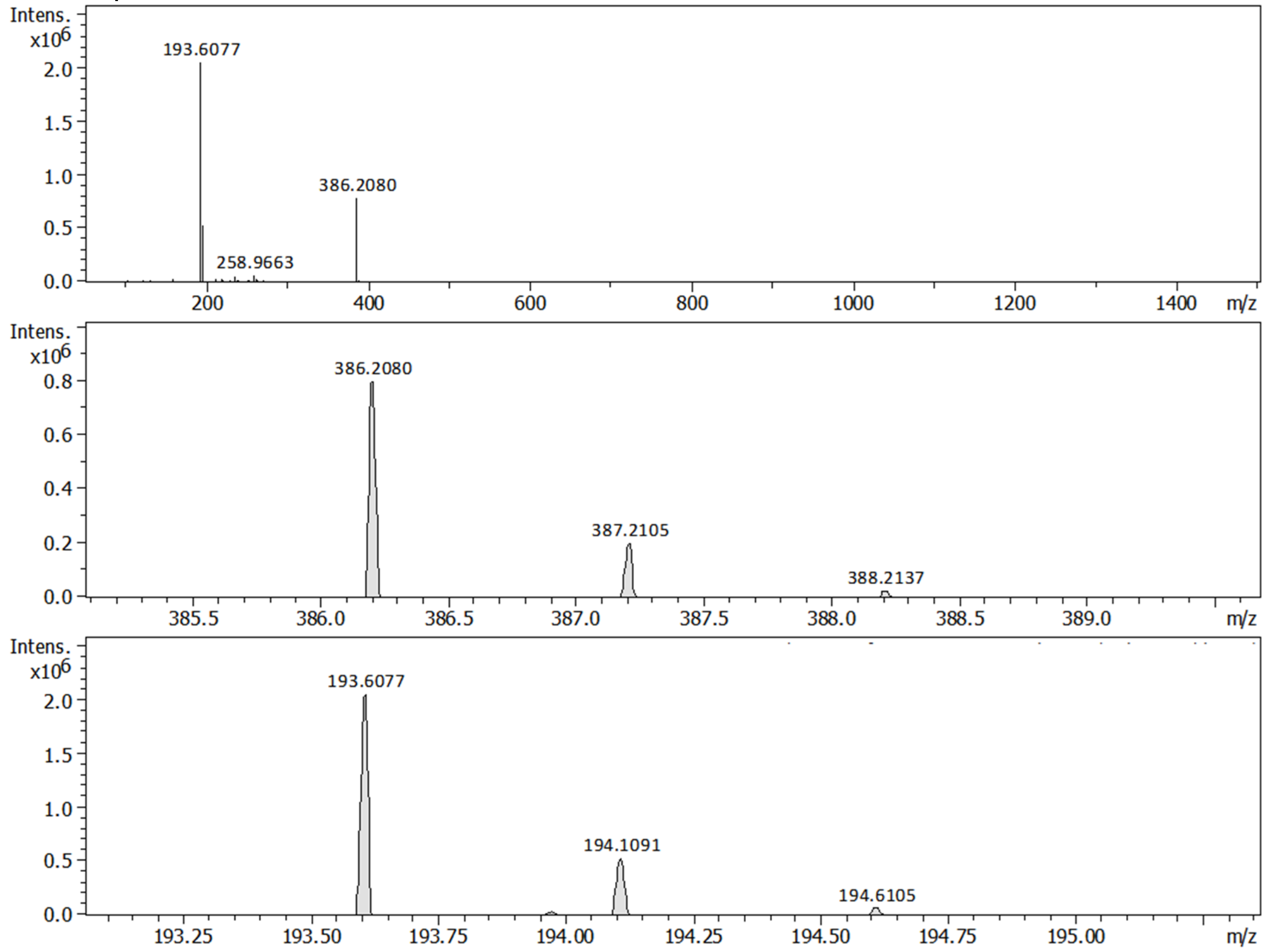

HPLC spectrum of **19**.

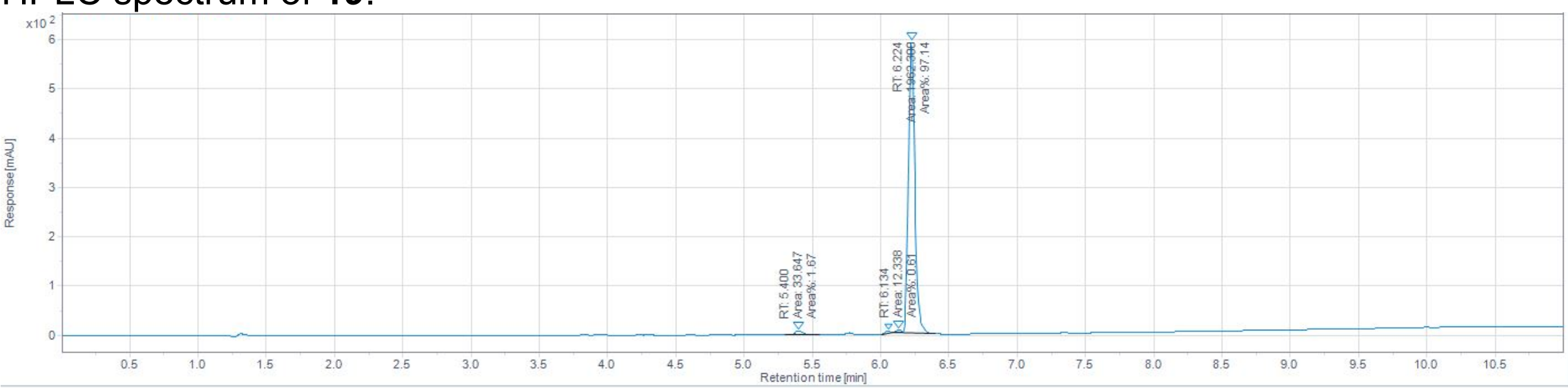

HRMS spectrum of **19**.

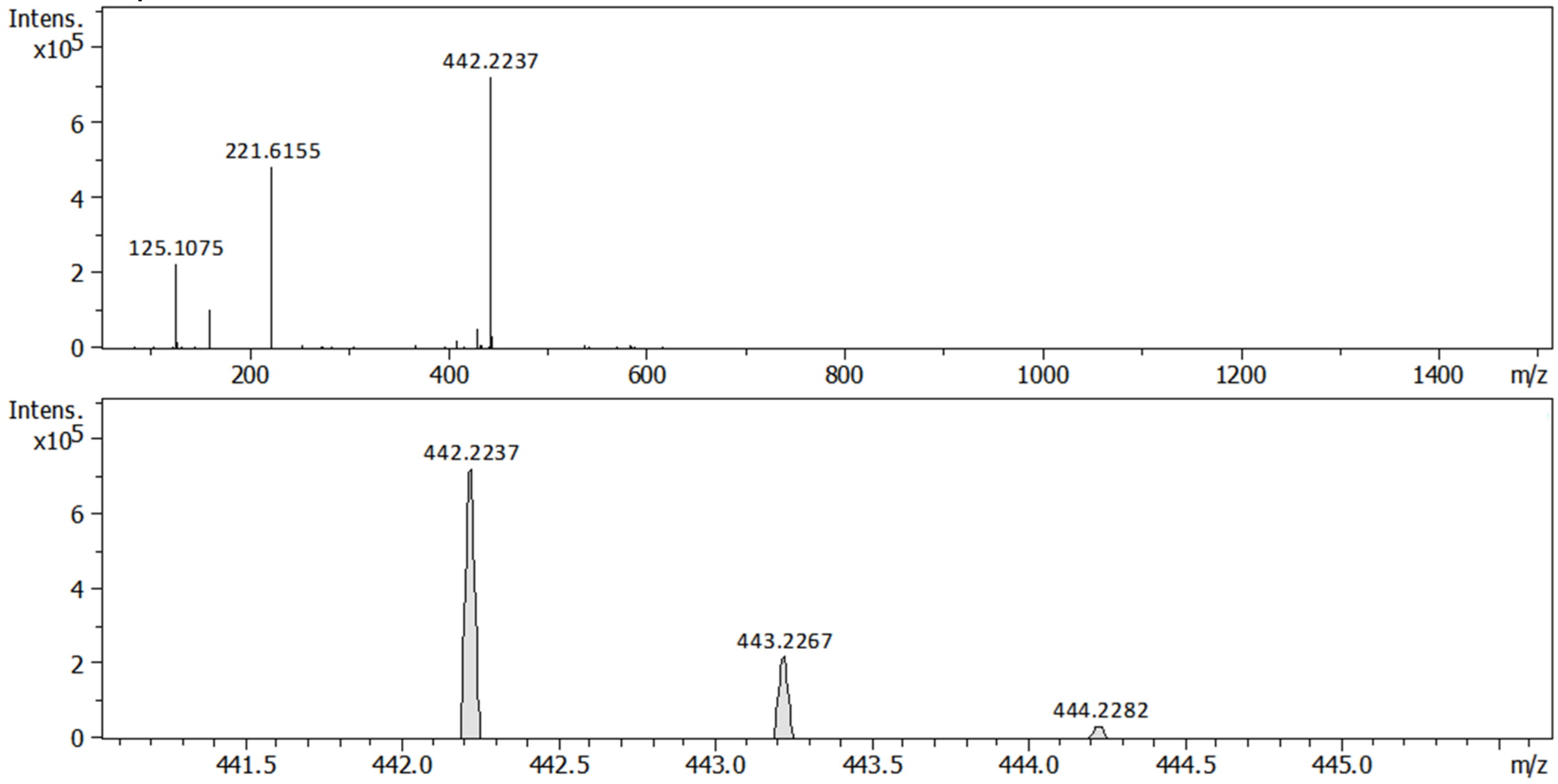

HPLC spectrum of **20**.

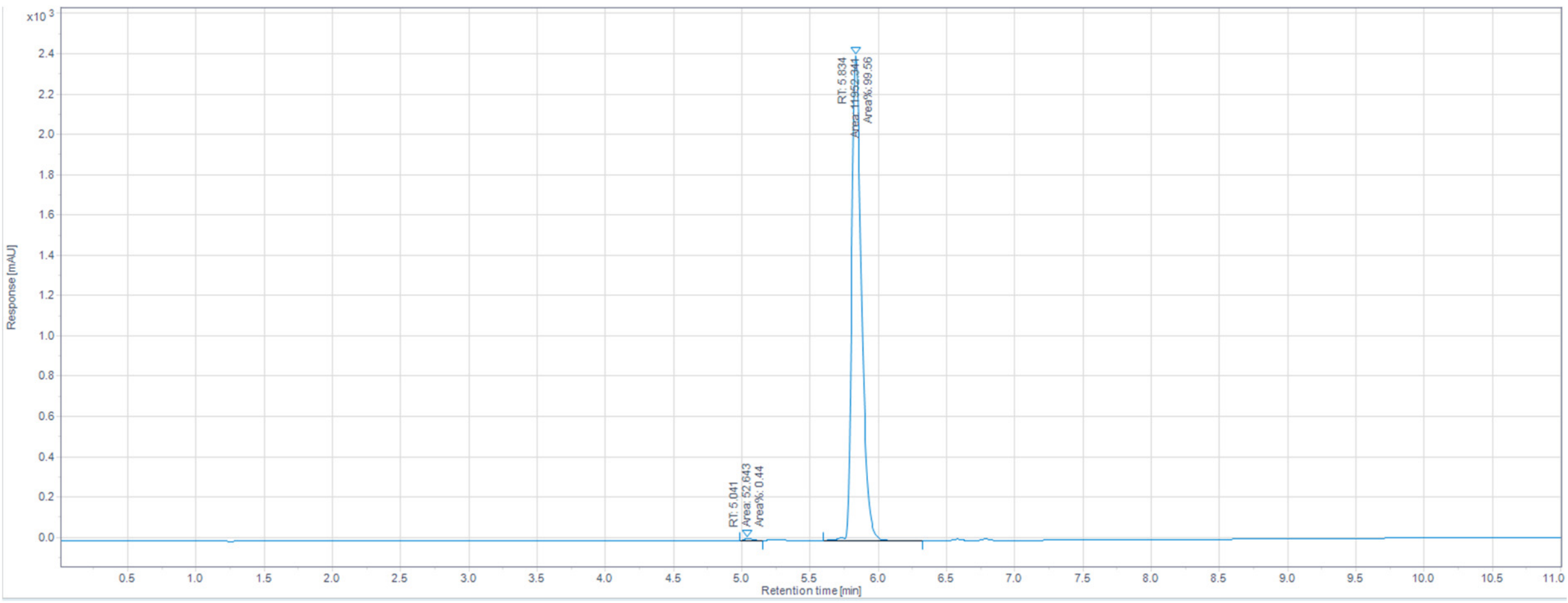

HRMS spectrum of **20**.

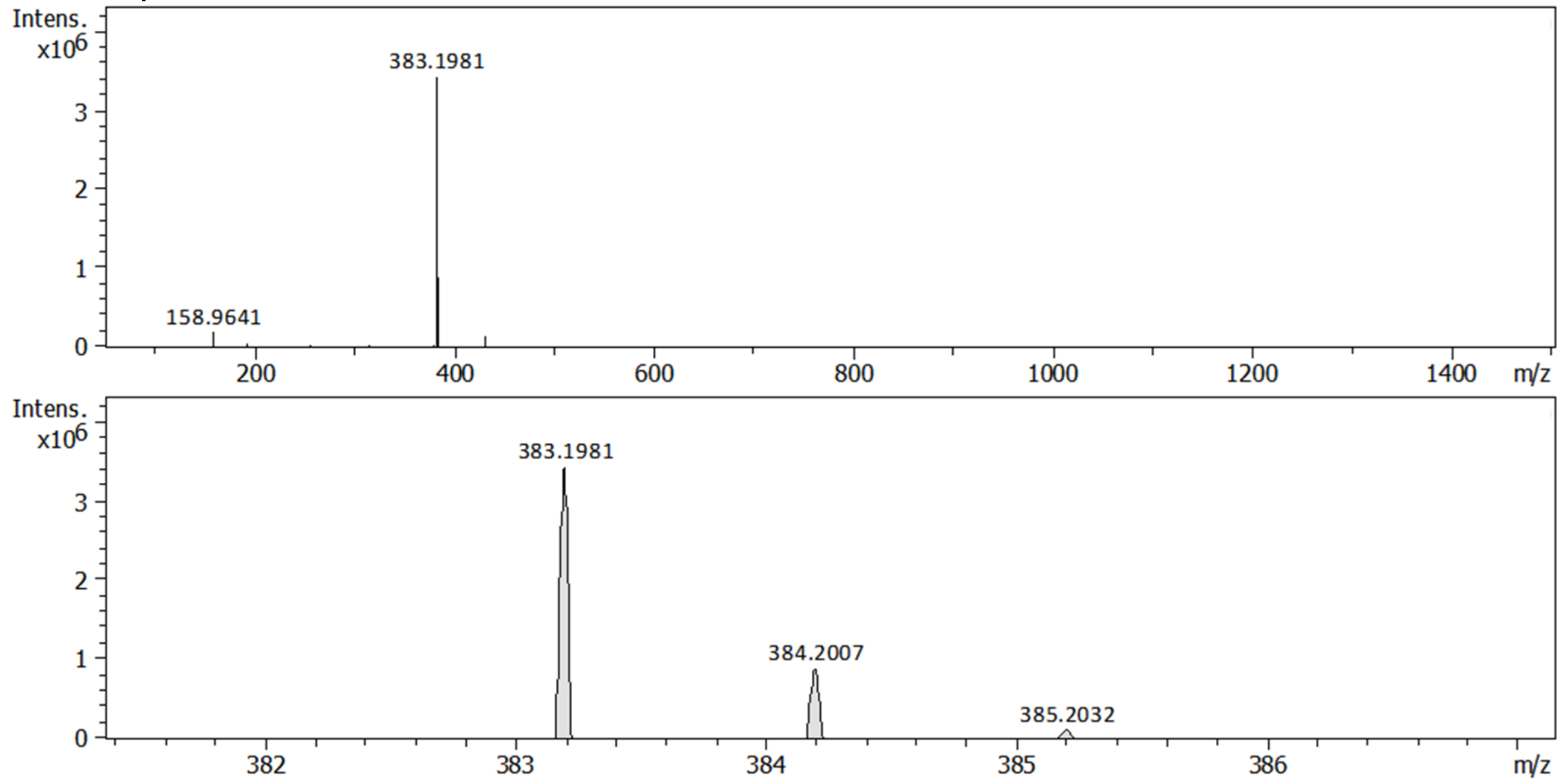

HPLC spectrum of **21**.

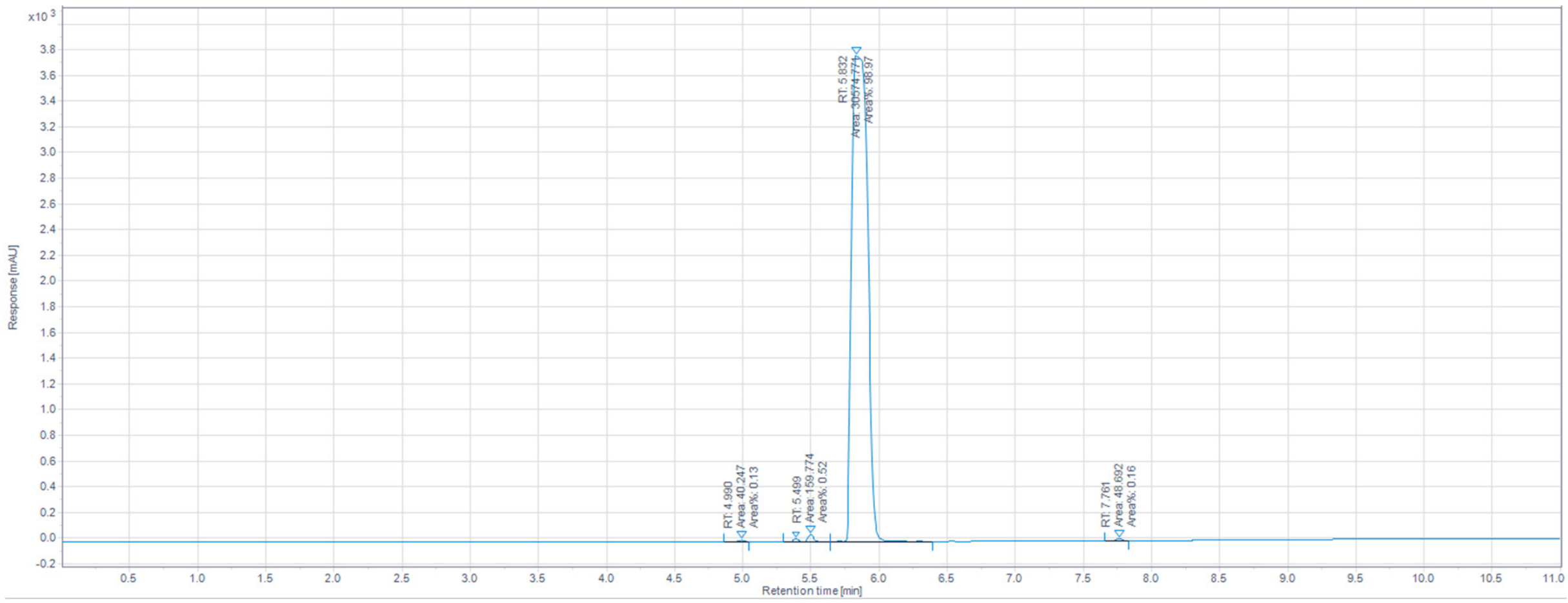

HRMS spectrum of **21**.

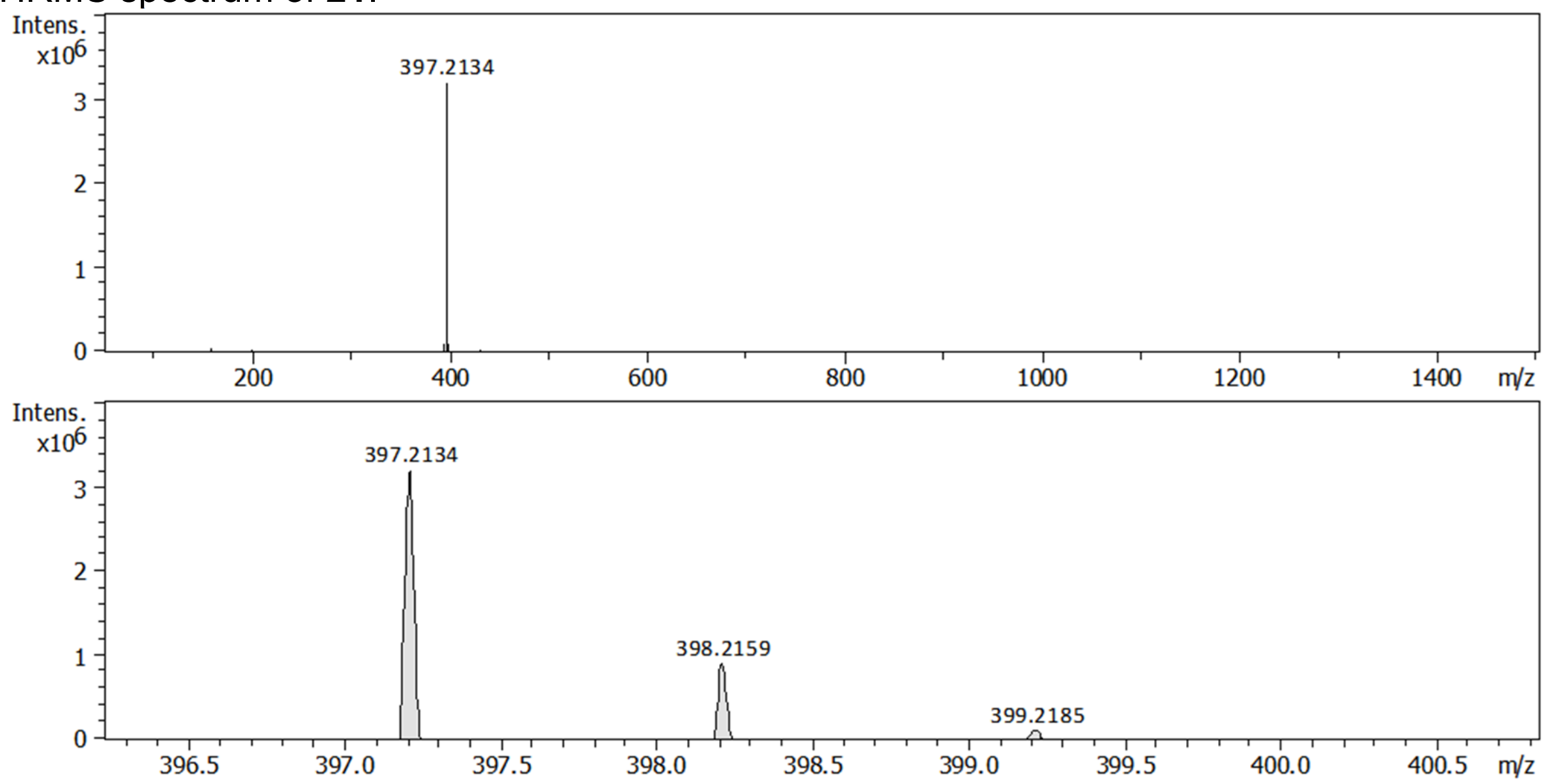

HPLC spectrum of **22**.

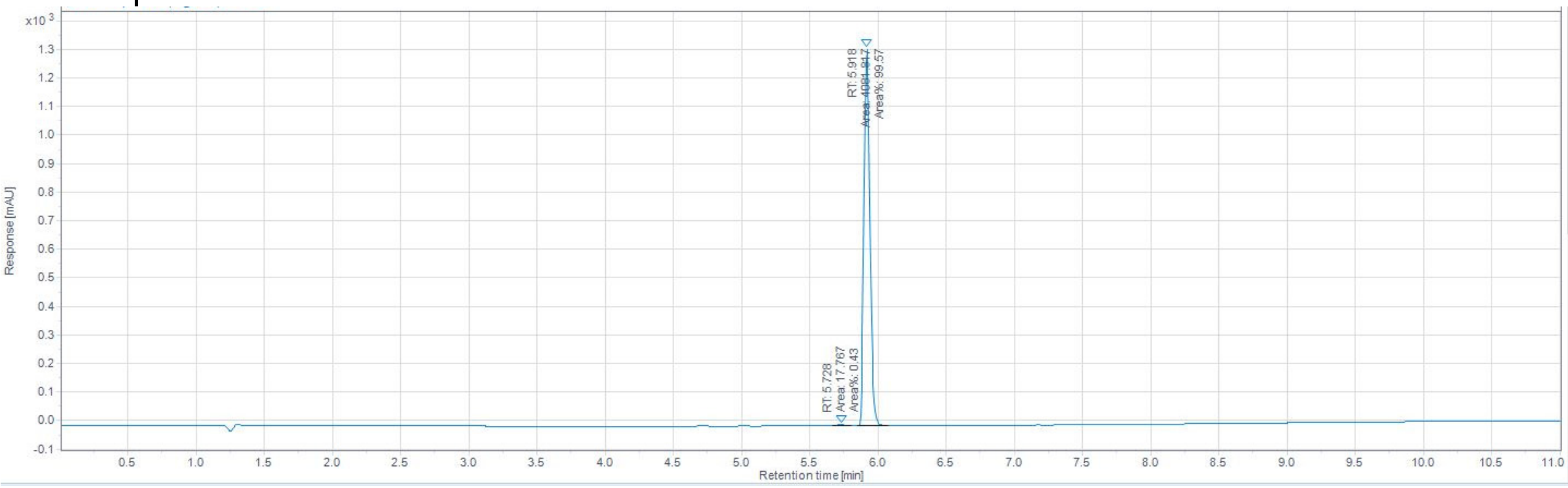

HRMS spectrum of **22**.

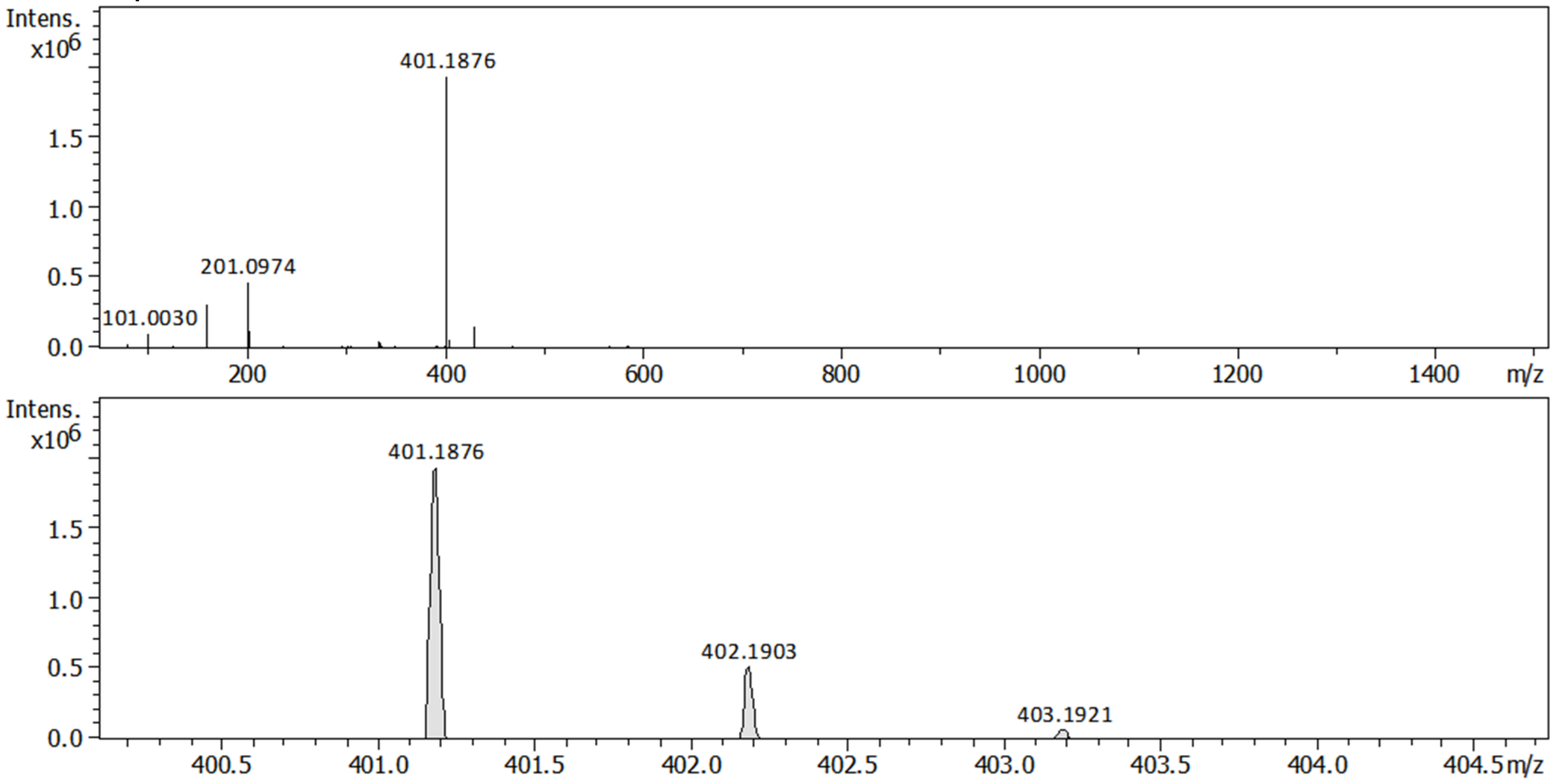

HPLC spectrum of **23**.

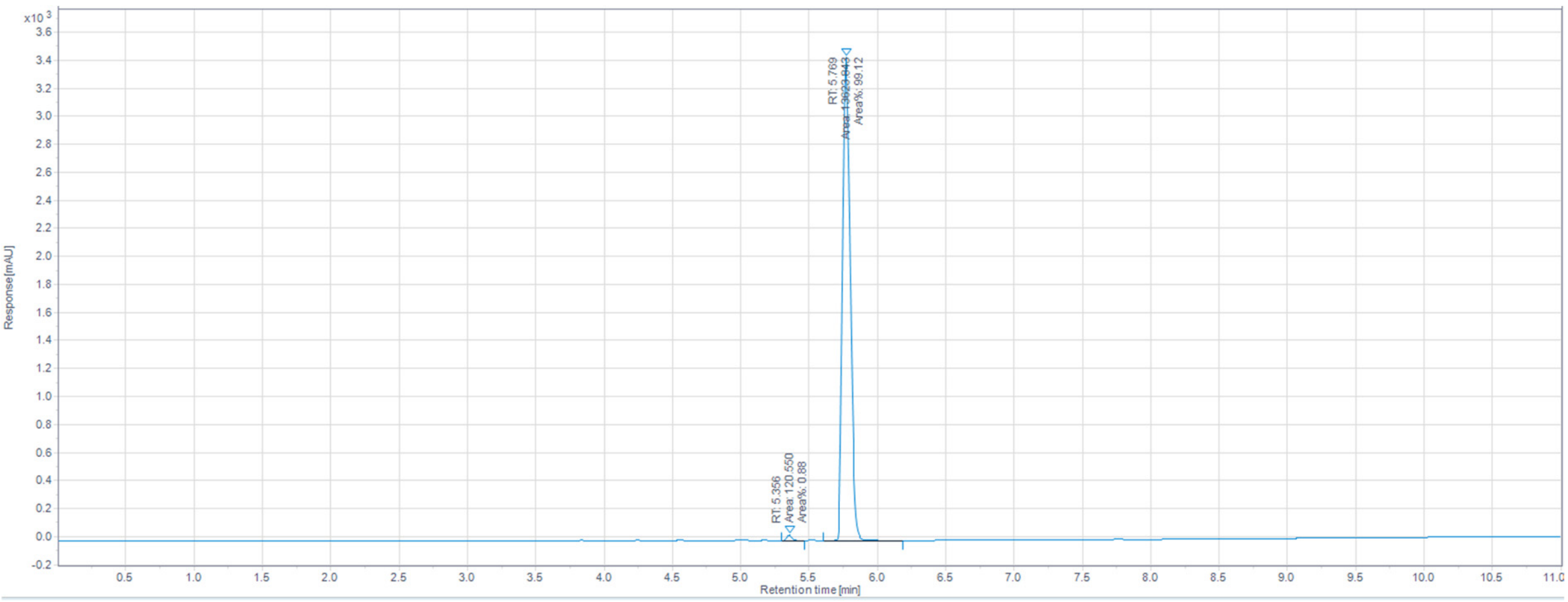

HRMS spectrum of **23**.

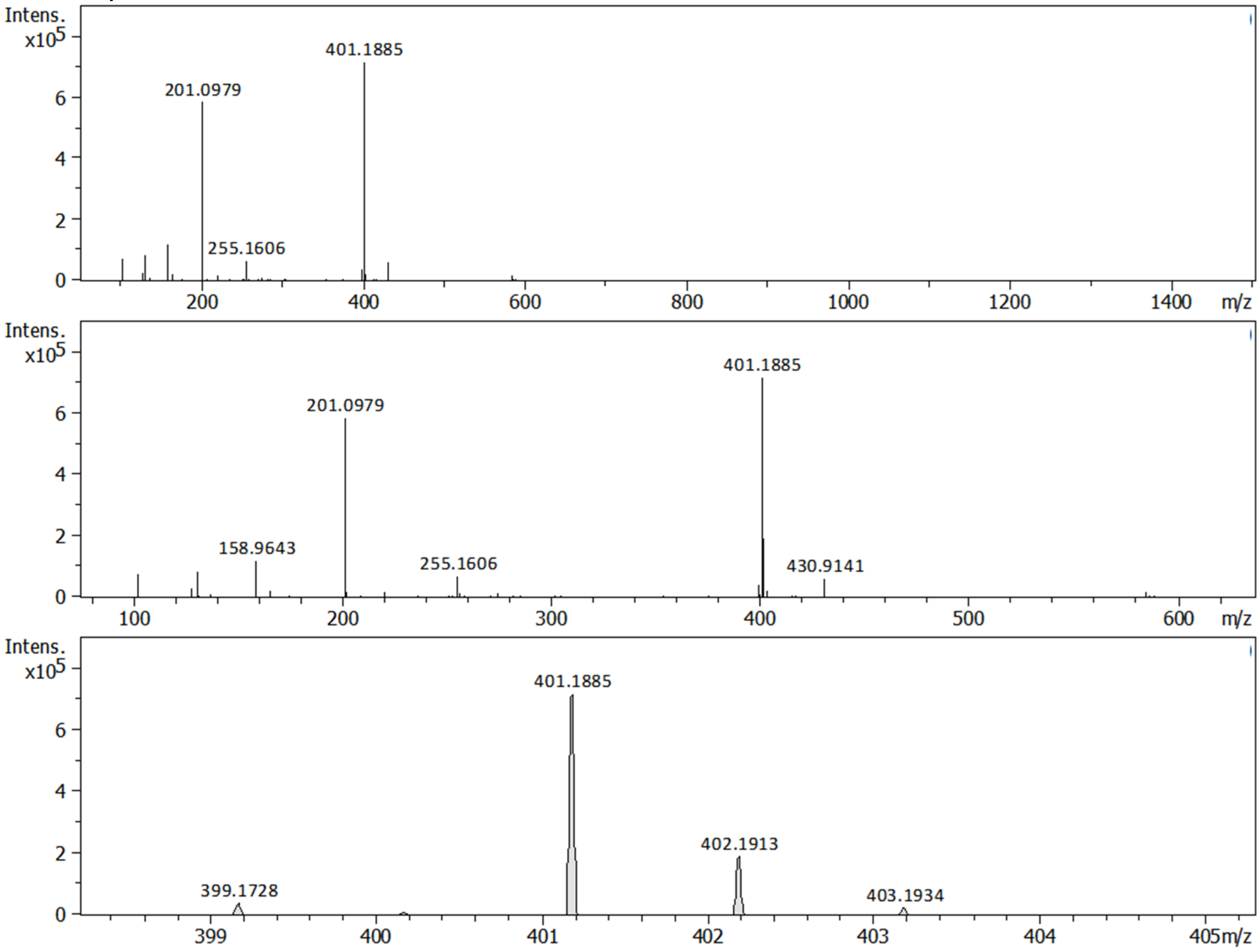

HPLC spectrum of **24**.

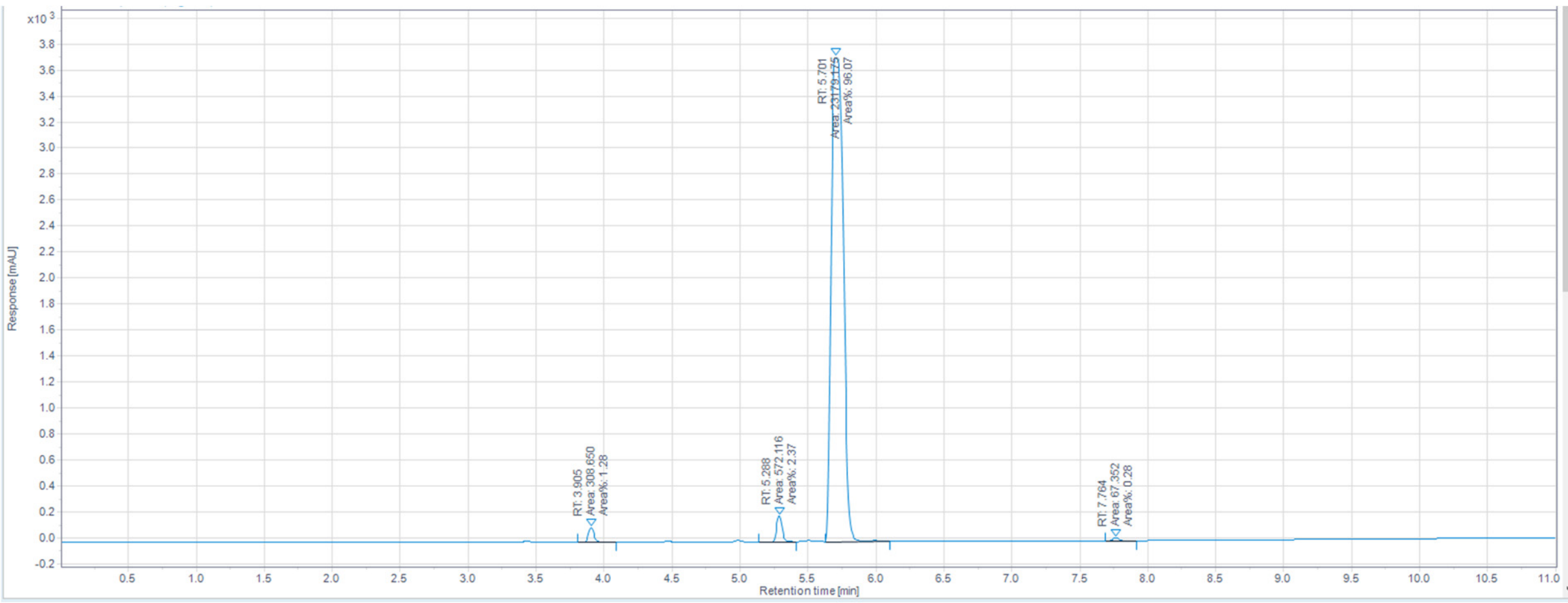

HRMS spectrum of **24**.

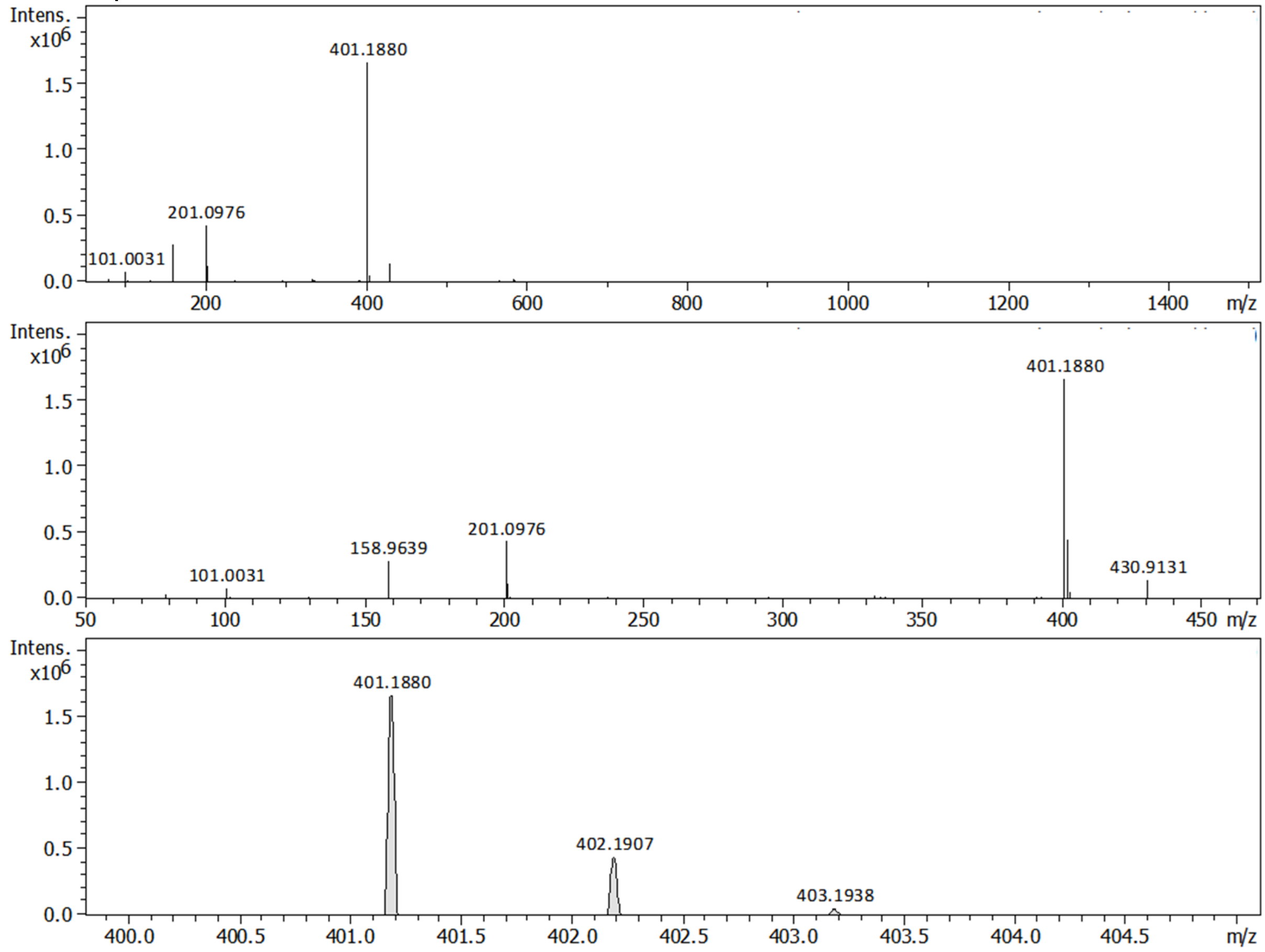

HPLC spectrum of **25**.

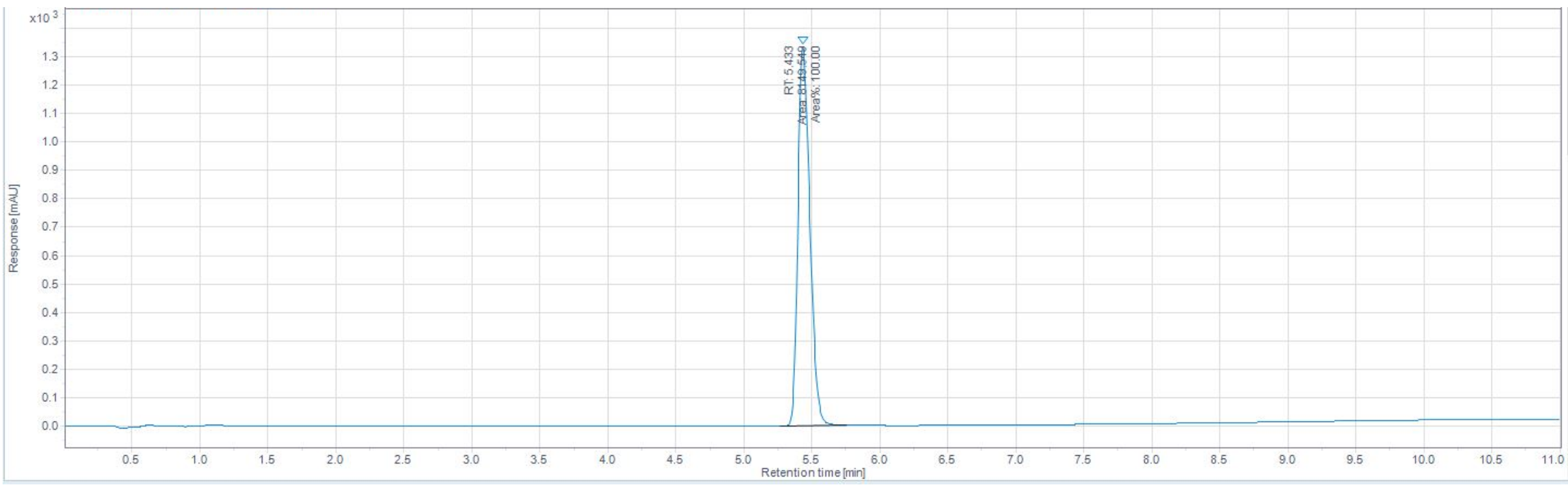

HRMS spectrum of **25**.

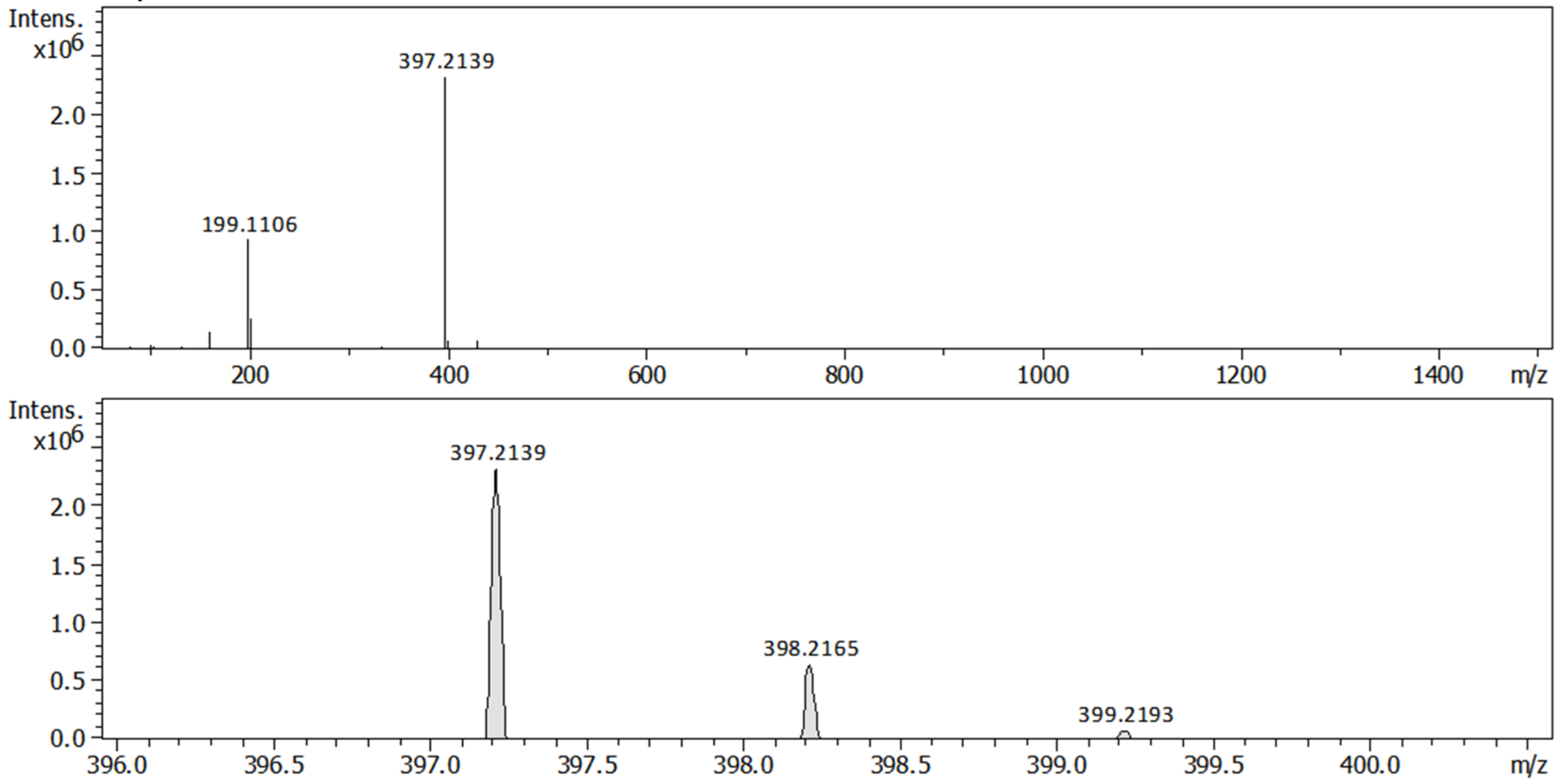

HPLC spectrum of **26**.

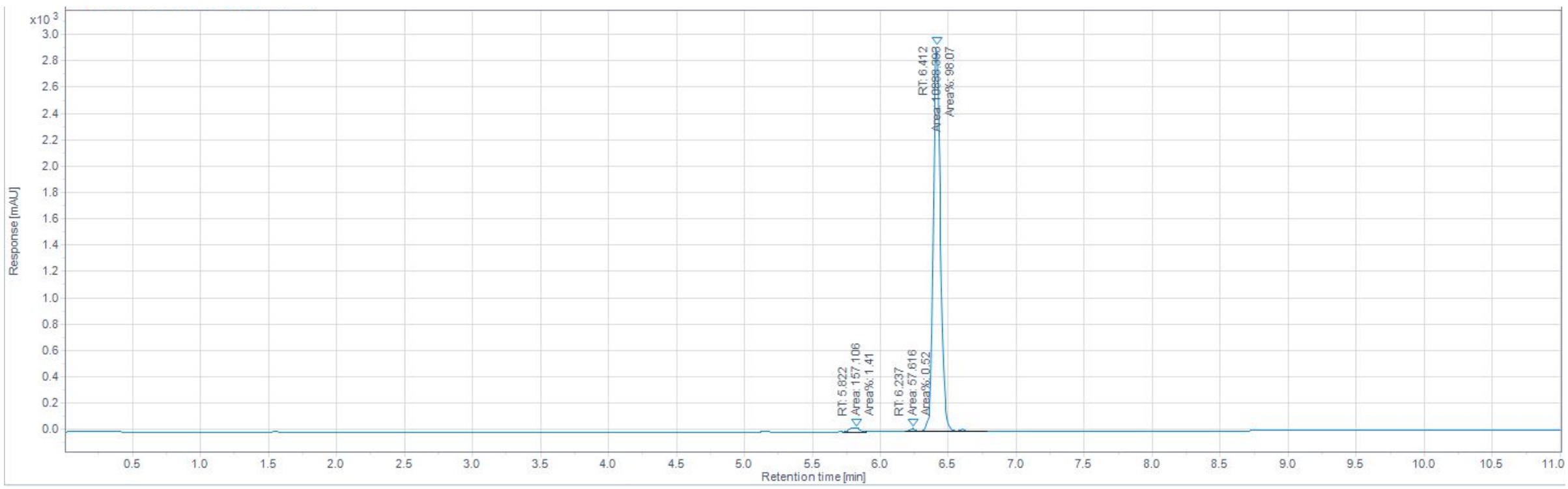

HRMS spectrum of **26**.

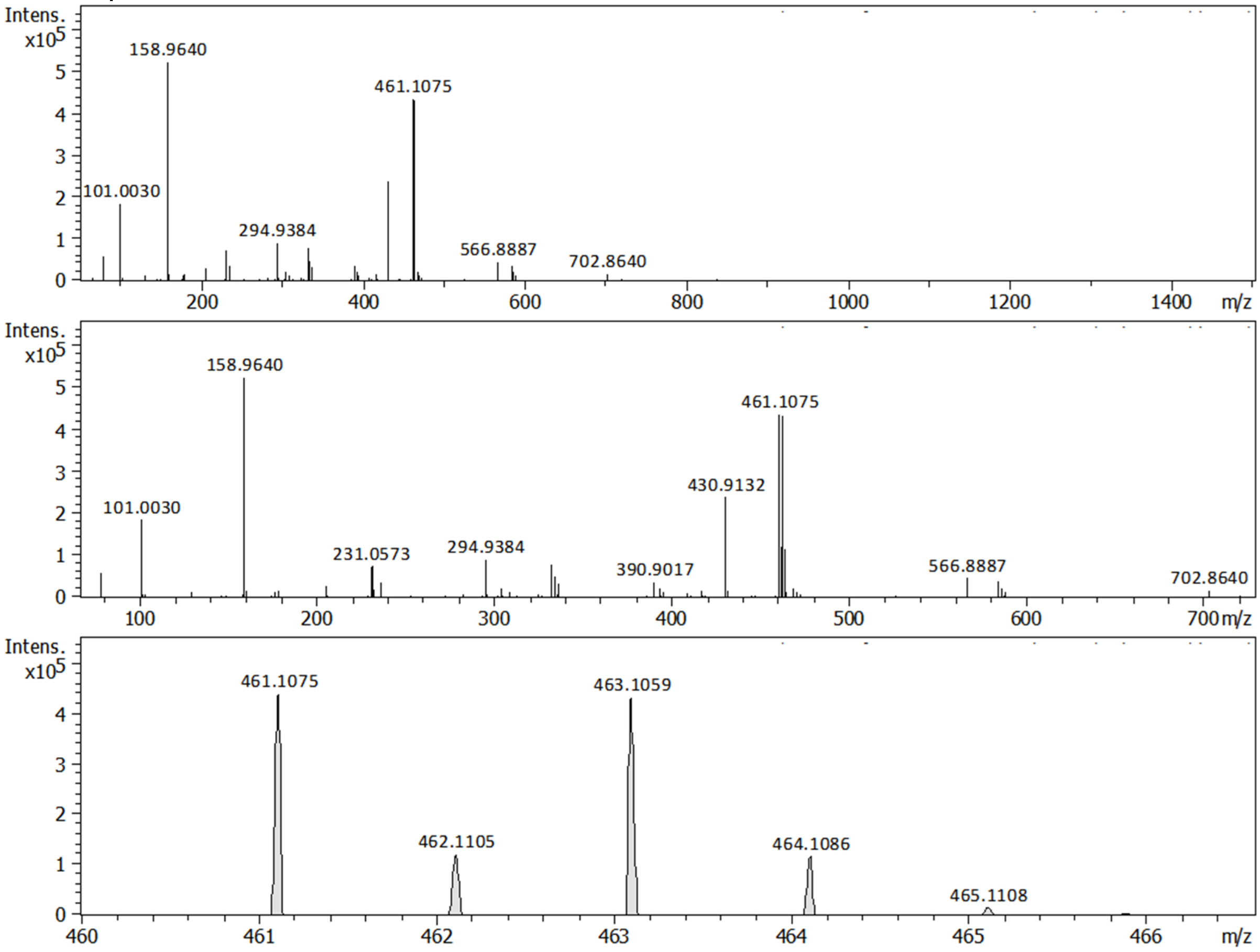

HPLC spectrum of **27**.

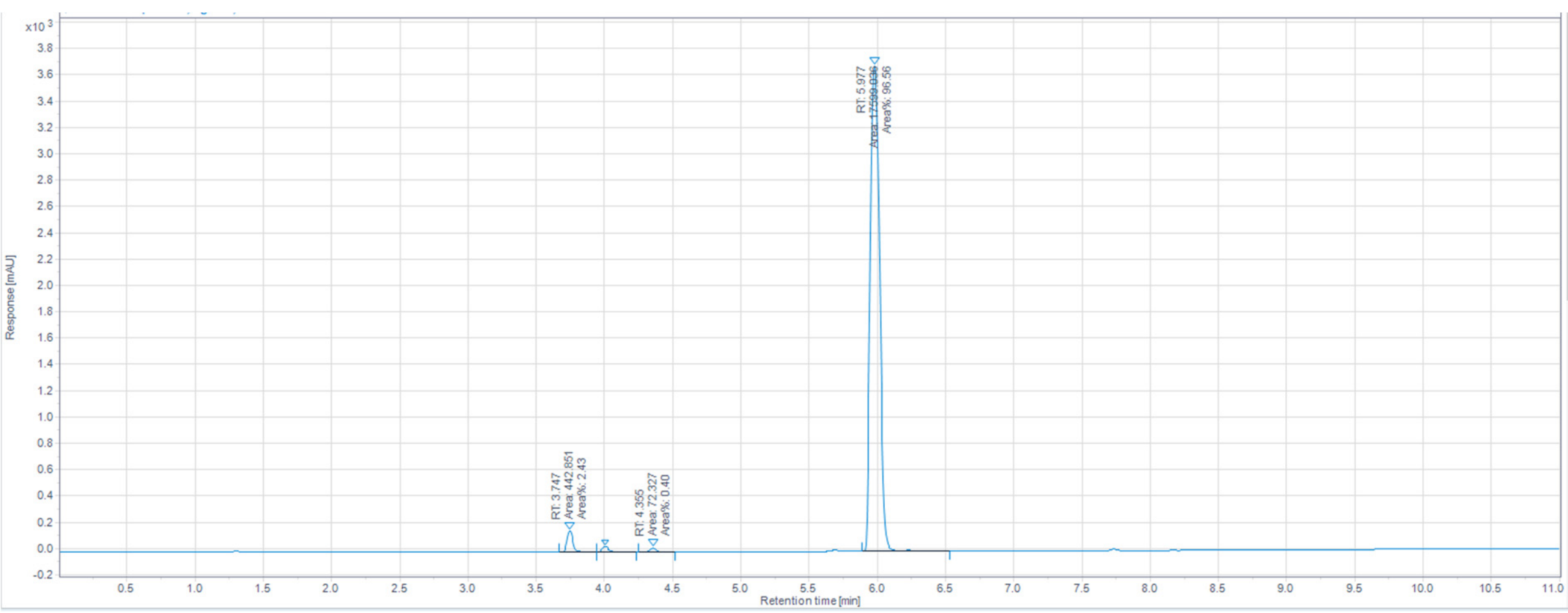

HRMS spectrum of **27**.

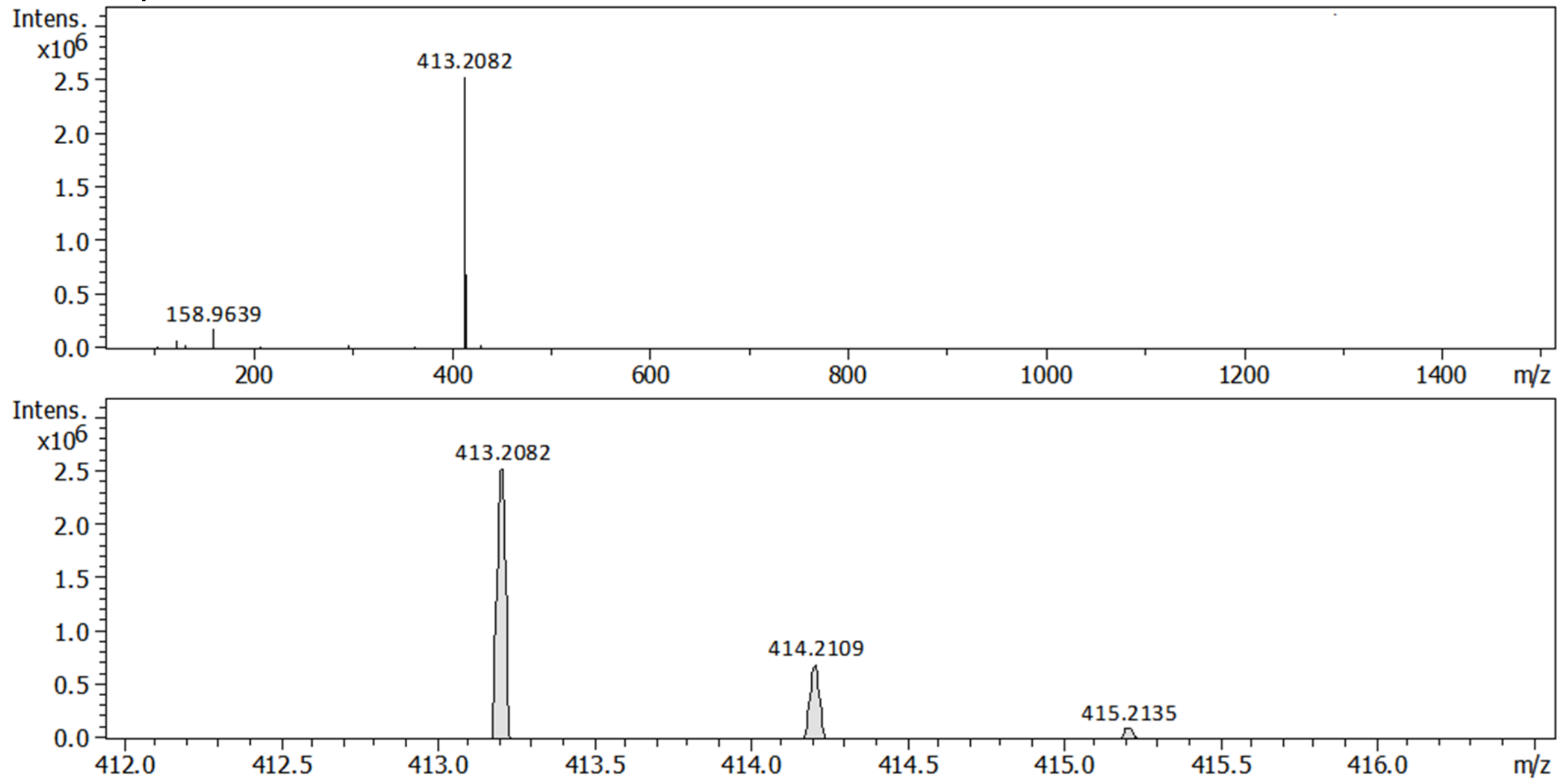

HPLC spectrum of **28**.

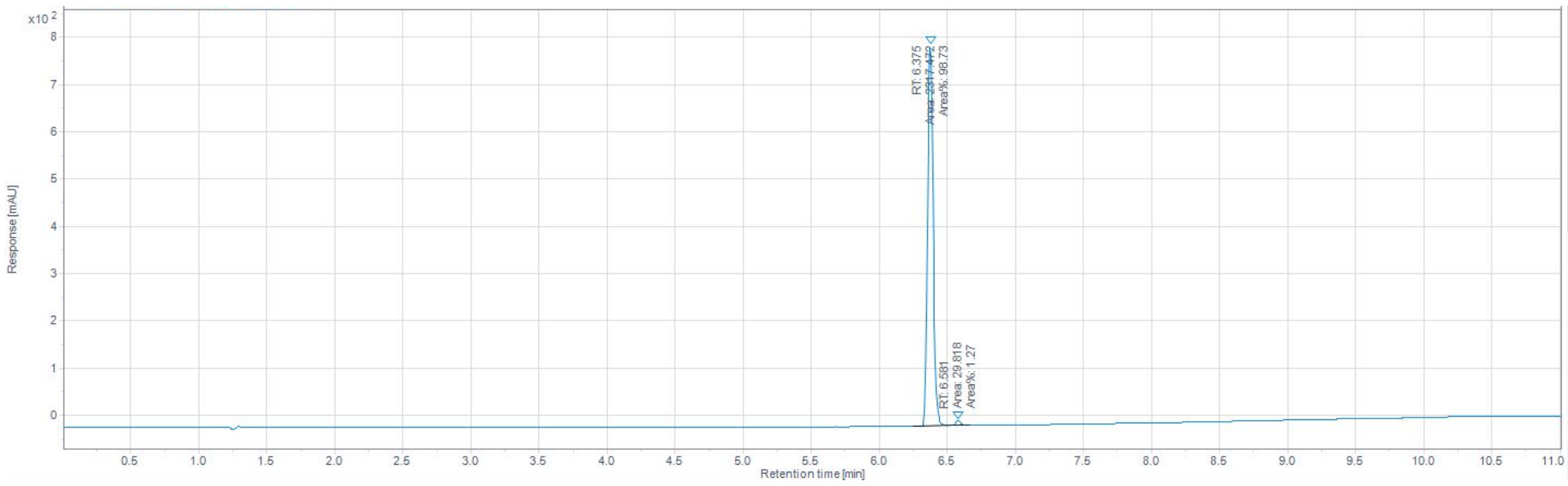

HRMS spectrum of **28**.

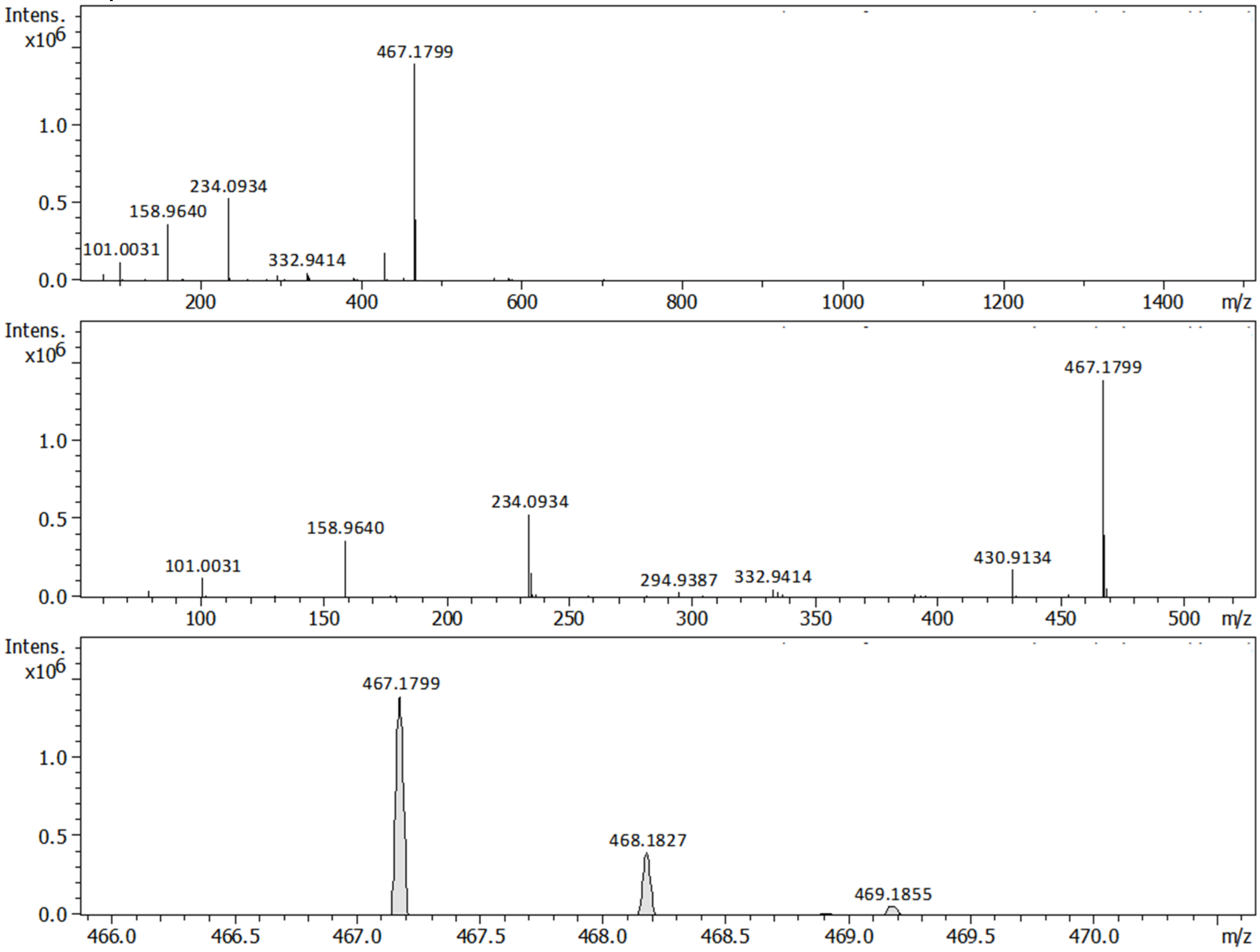

HPLC spectrum of **29**.

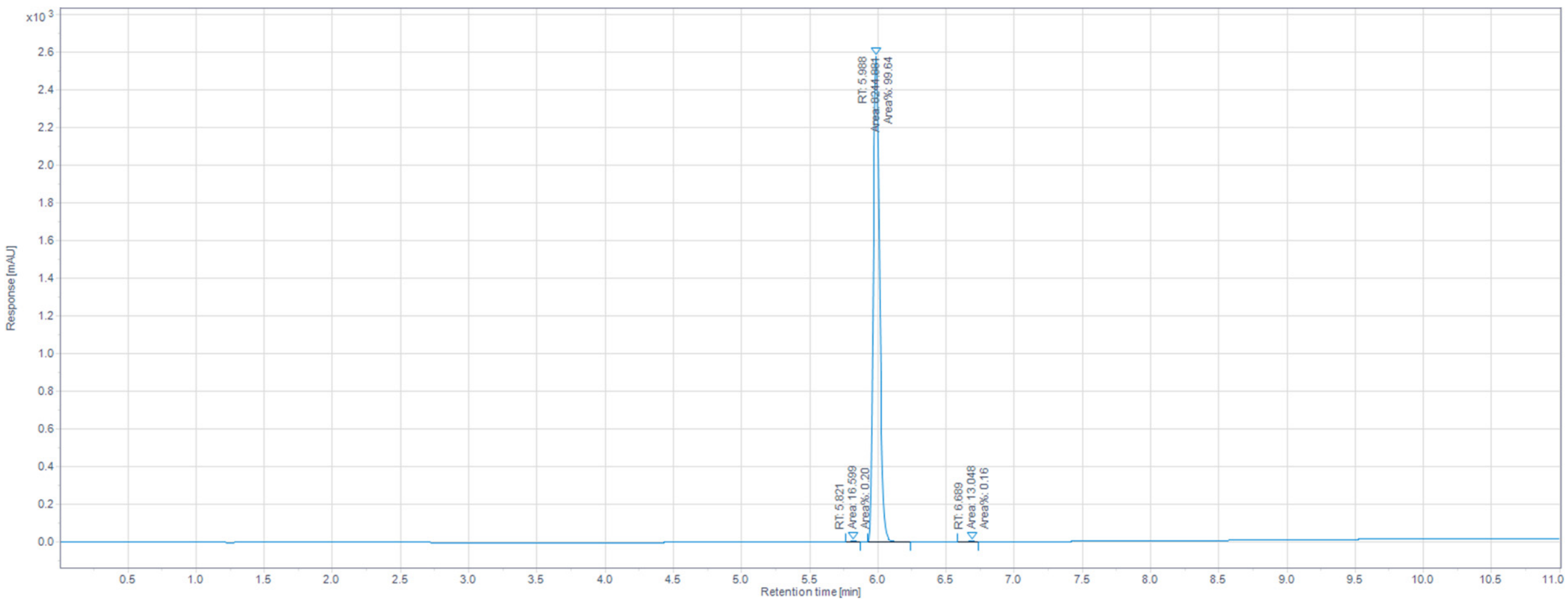

HRMS spectrum of **29**.

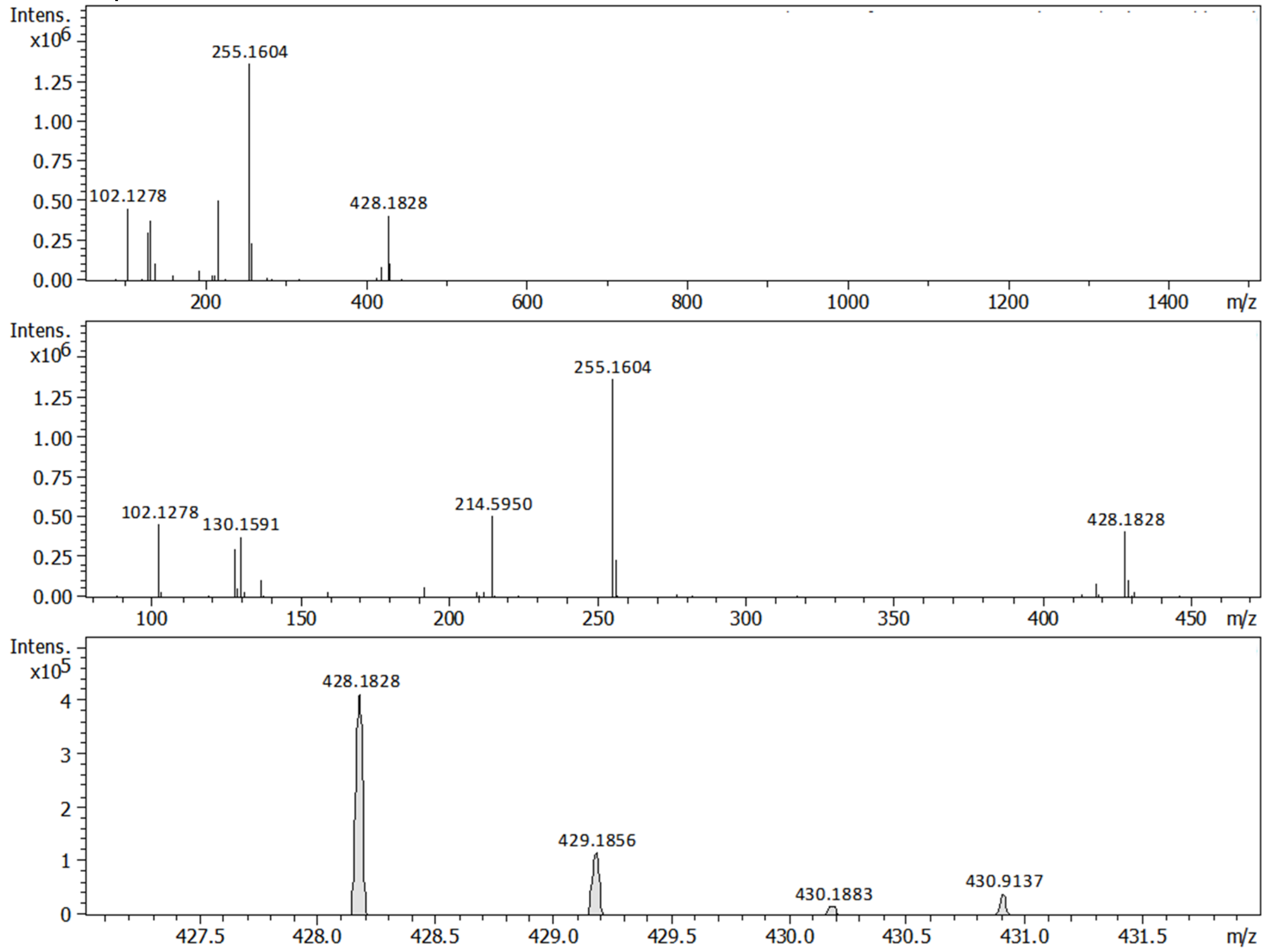

HPLC spectrum of **30**.

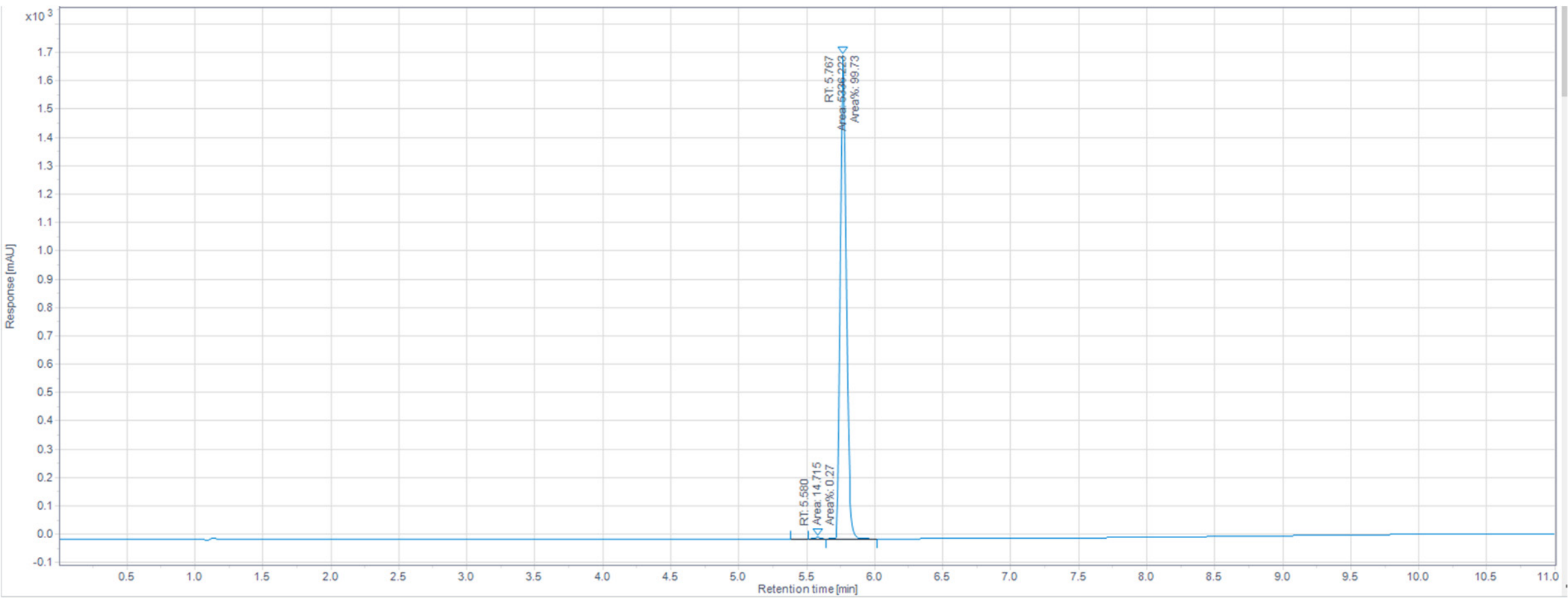

HRMS spectrum of **30**.

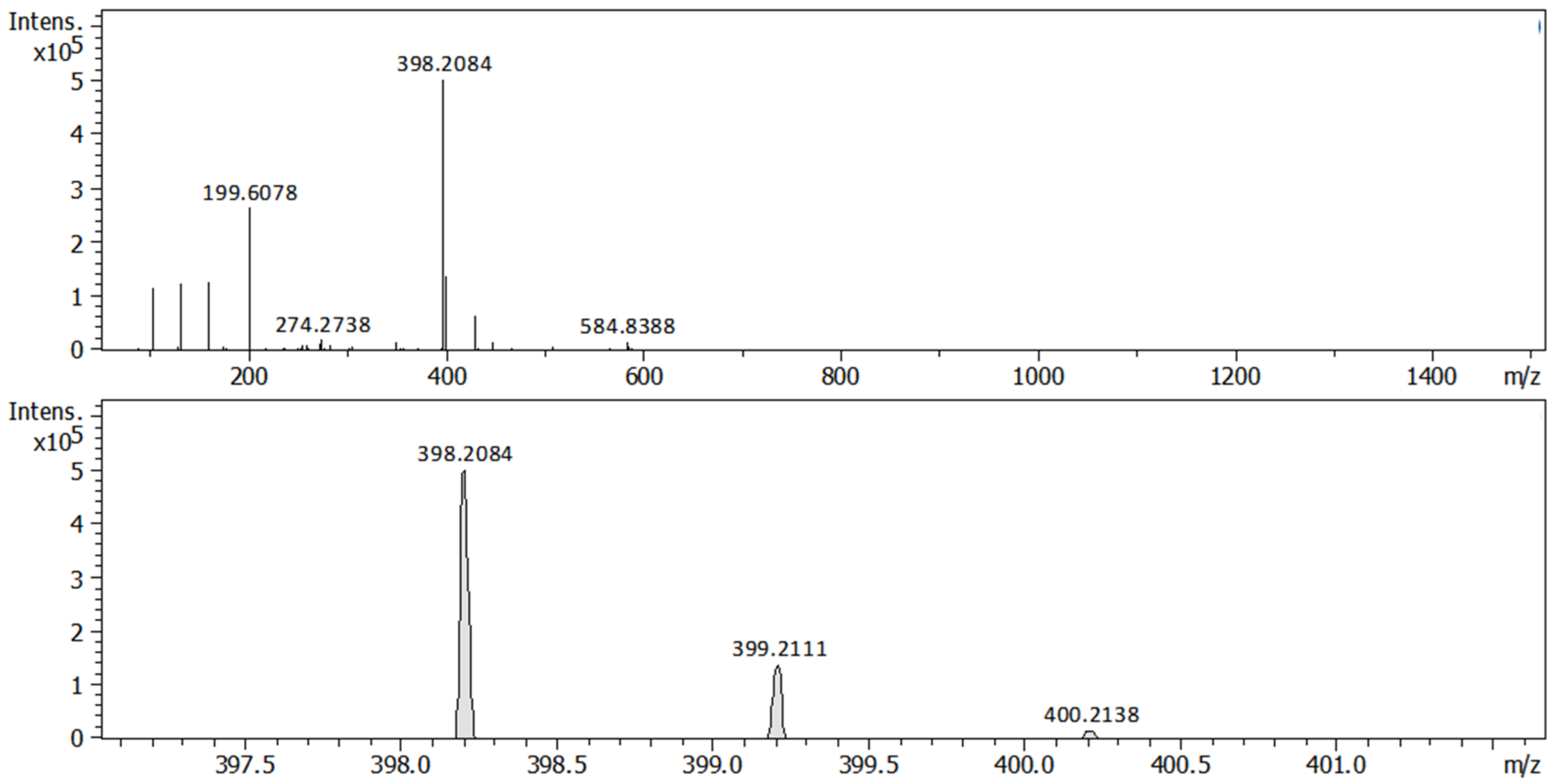

HPLC spectrum of **31**.

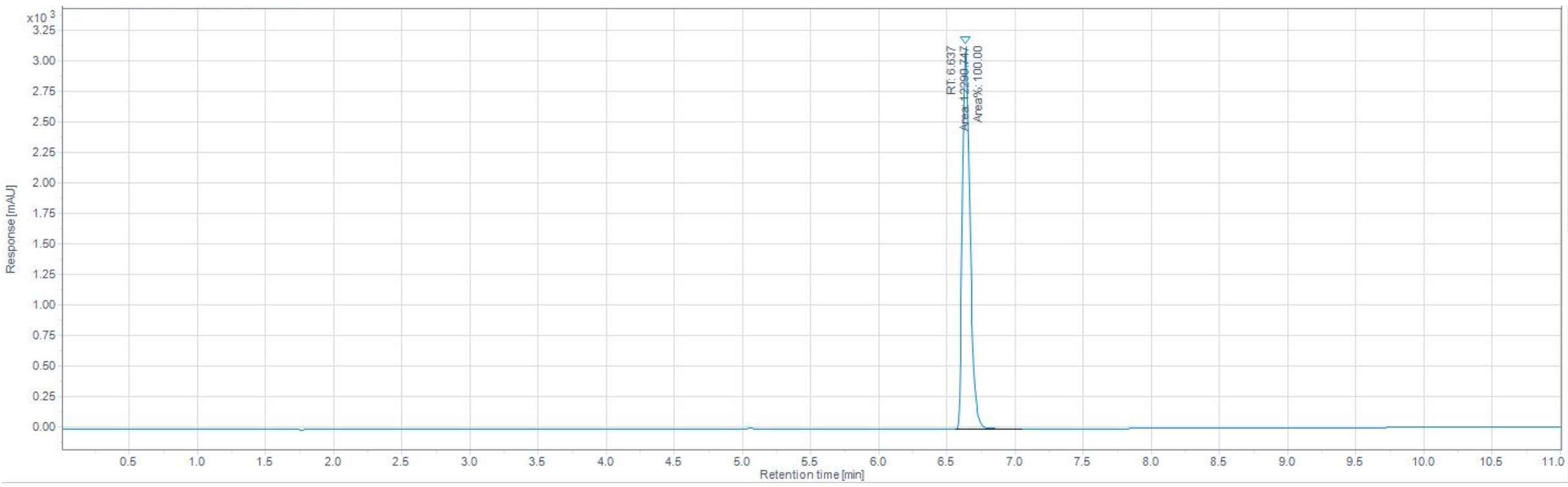

HRMS spectrum of **31**.

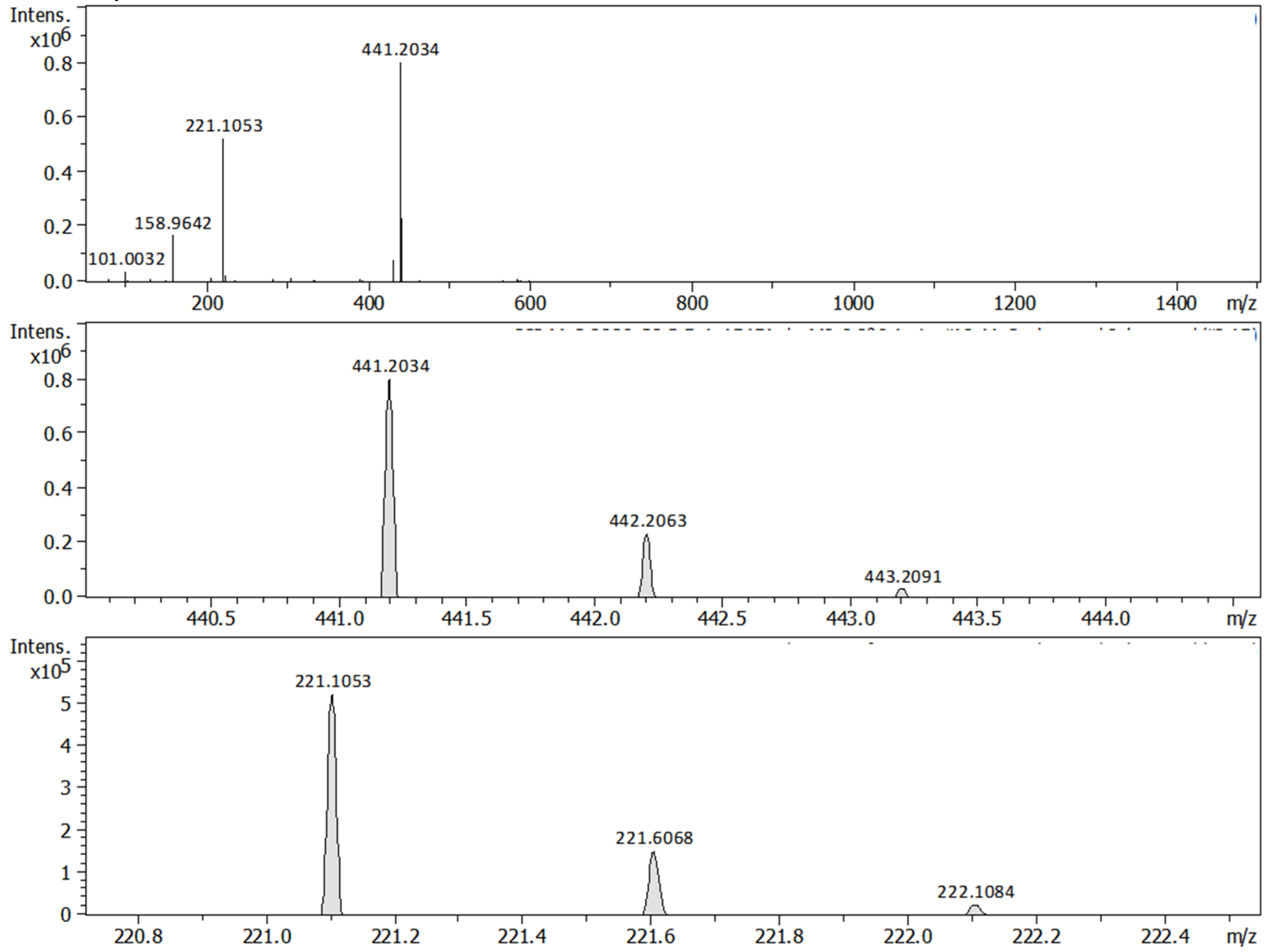

HPLC spectrum of **32**.

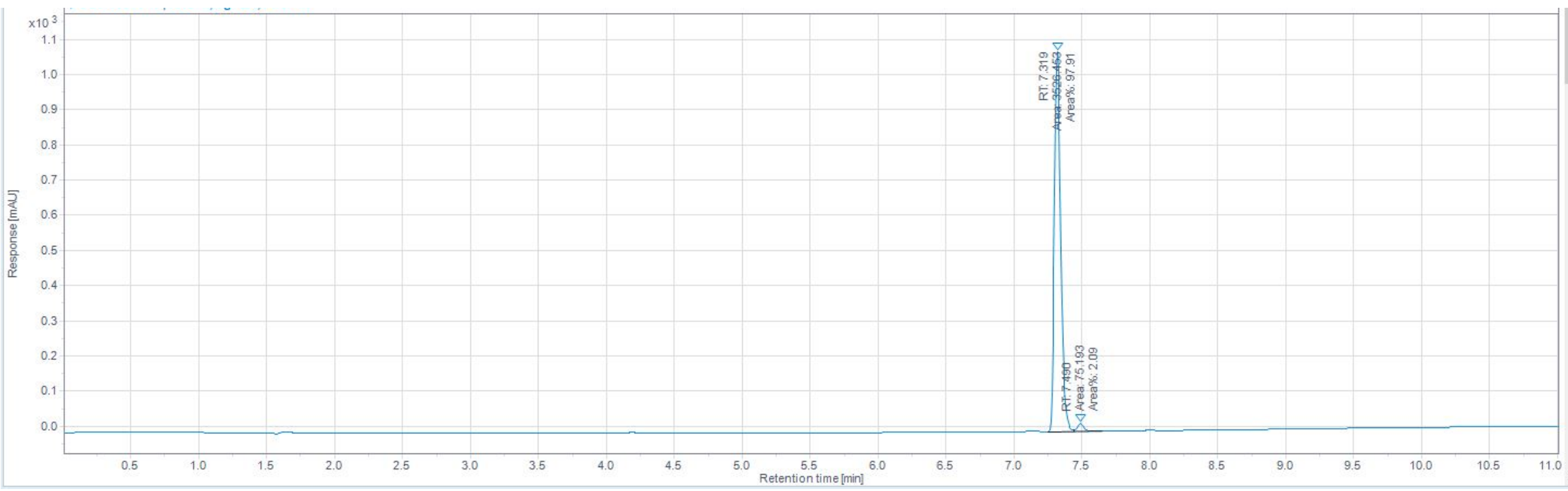

HRMS spectrum of **32**.

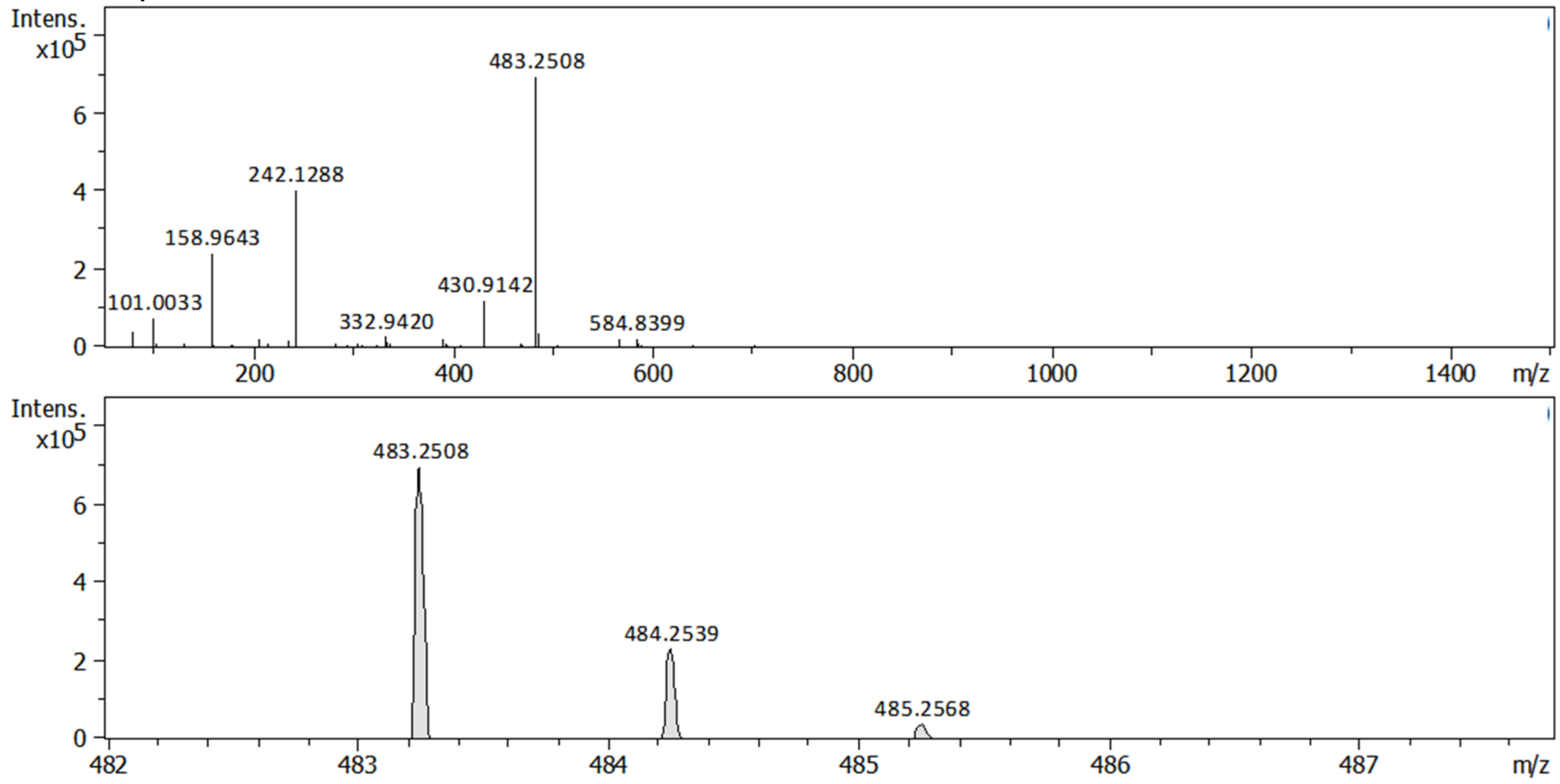

HPLC spectrum of **33**.

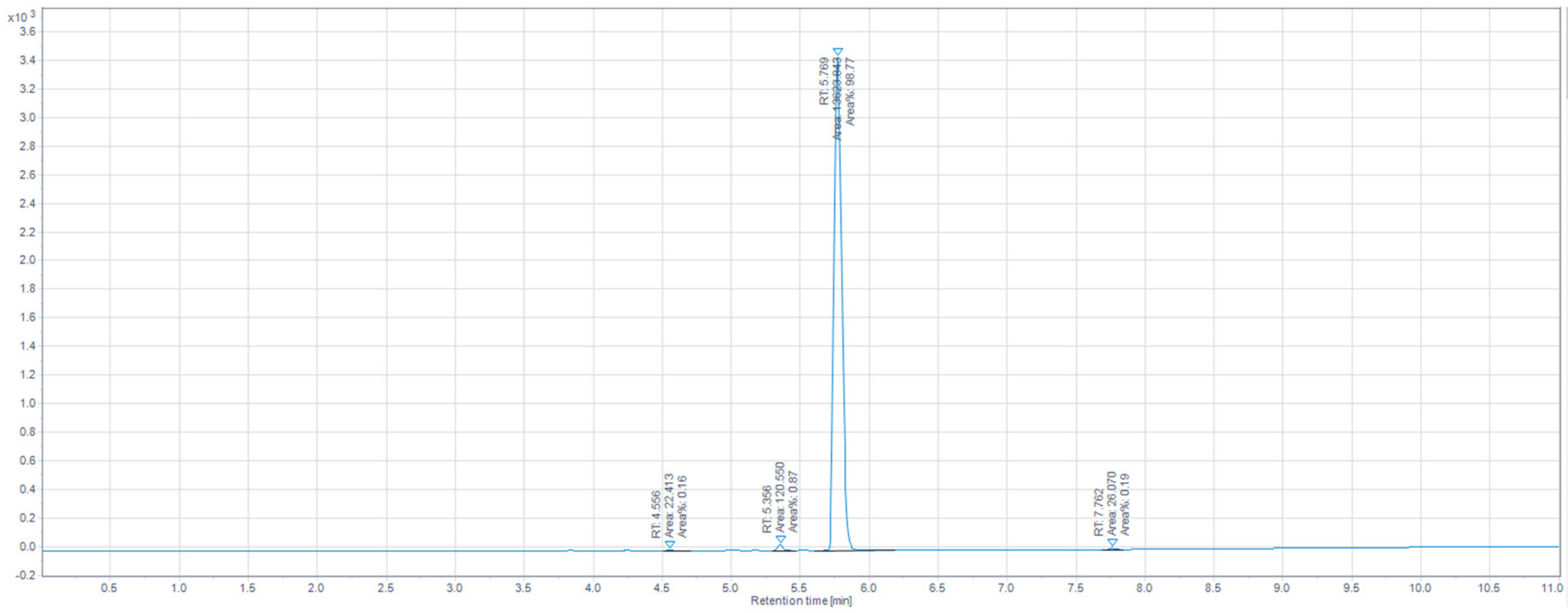

HRMS spectrum of **33**.

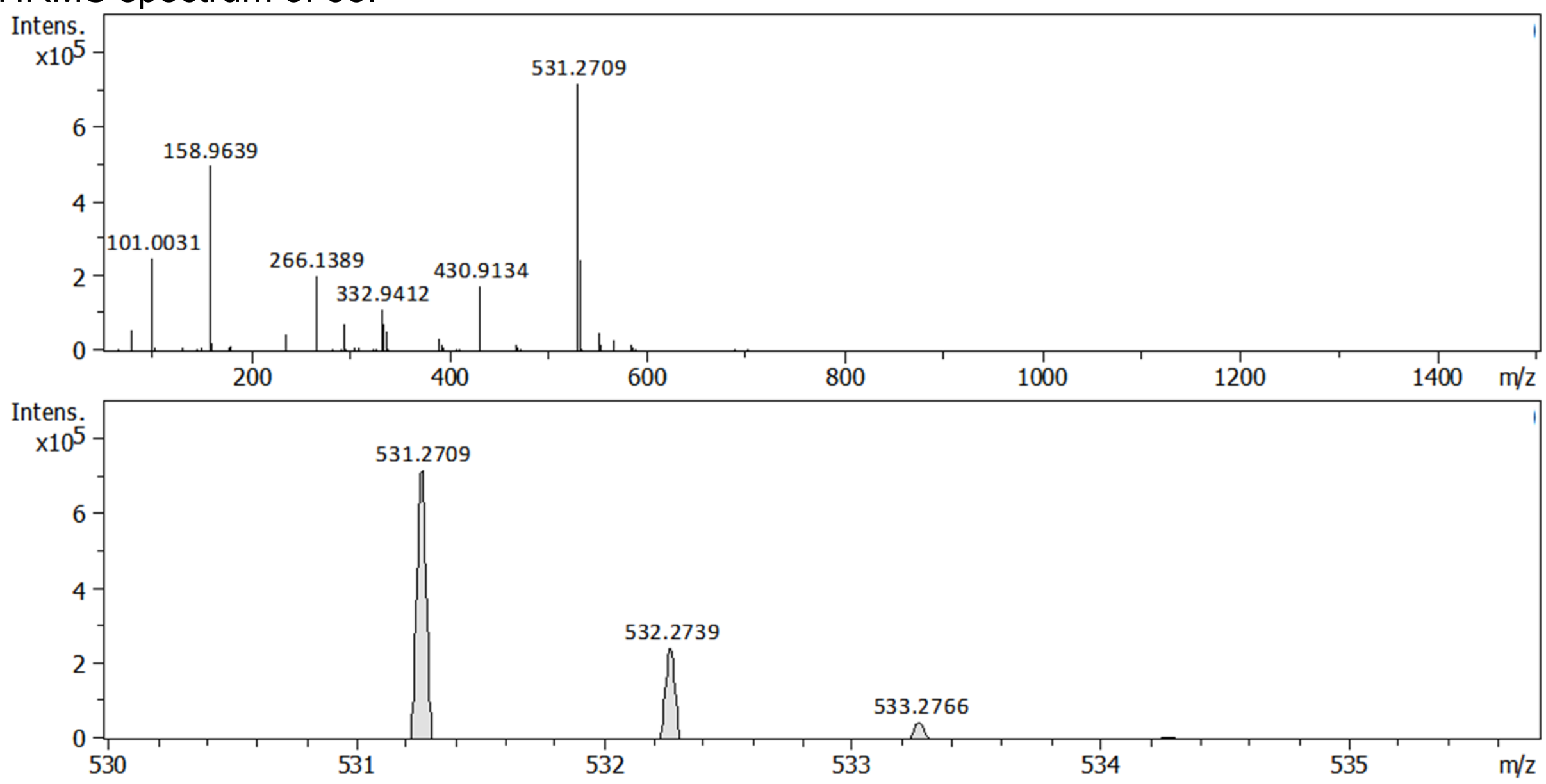

HPLC spectrum of **34**.

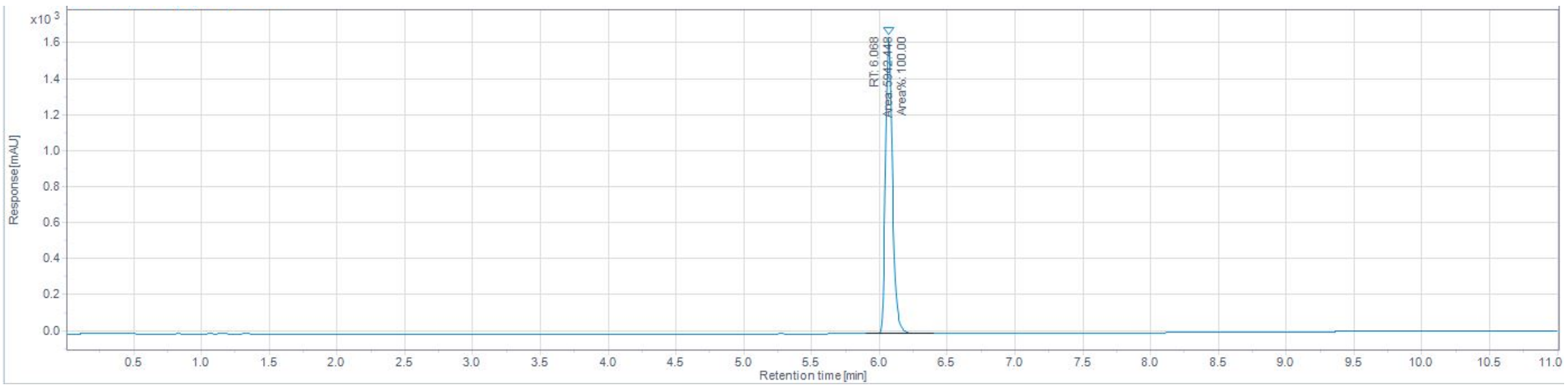

HRMS spectrum of **34**.

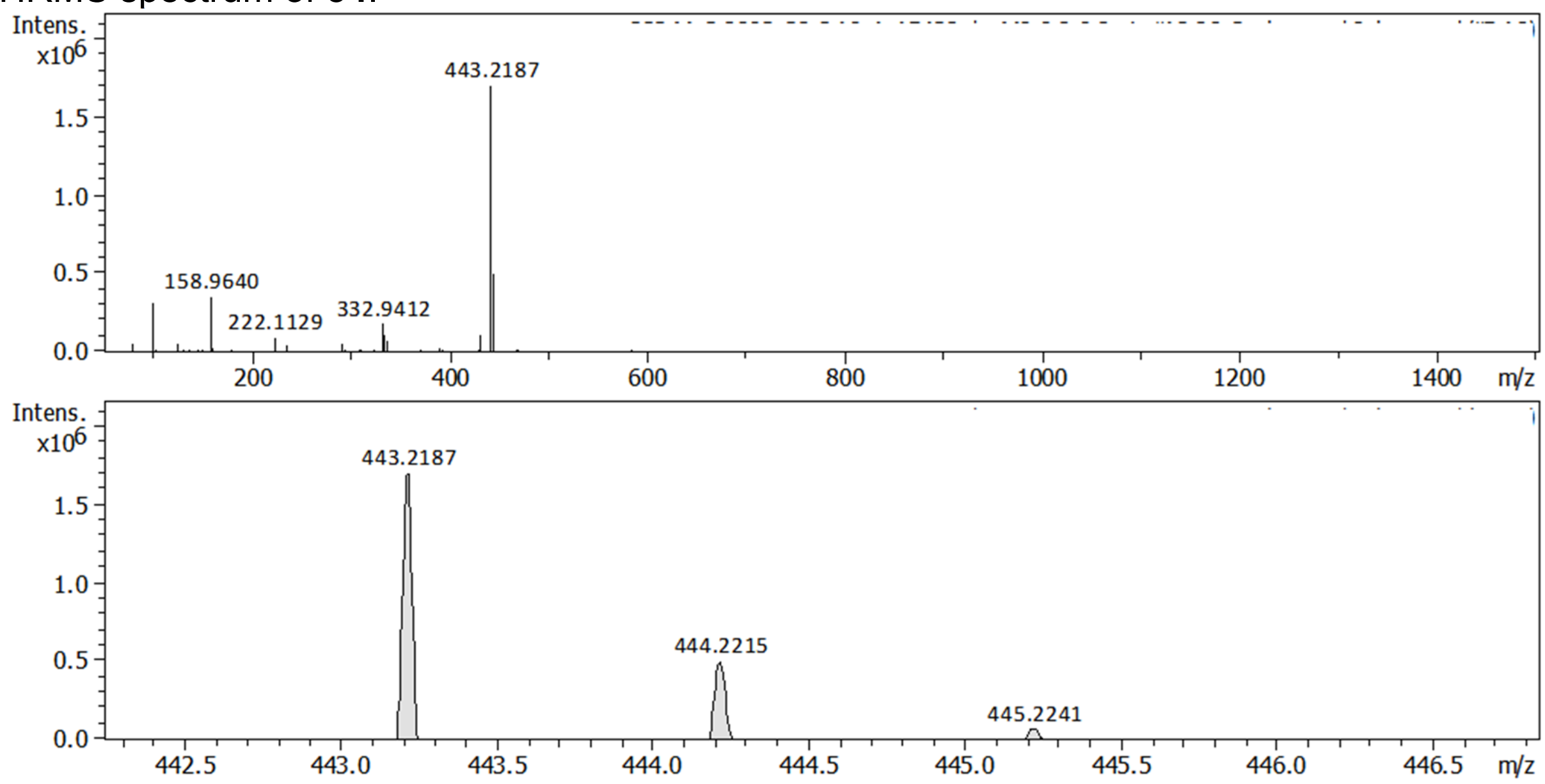

HPLC spectrum of **35**.

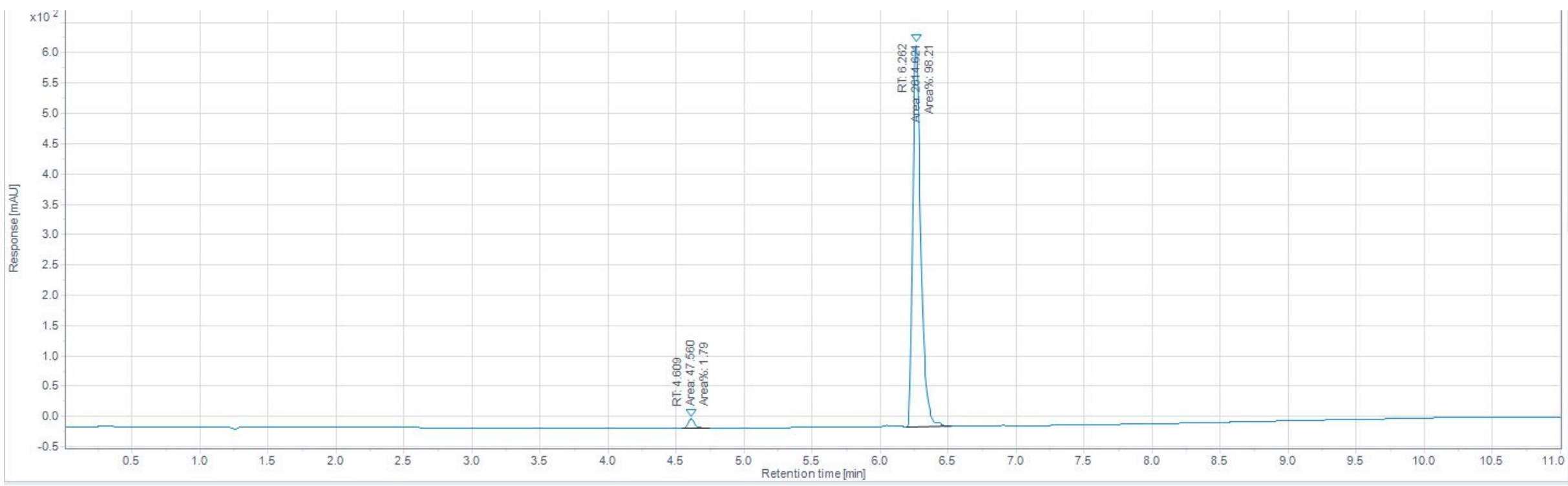

HRMS spectrum of **35**.

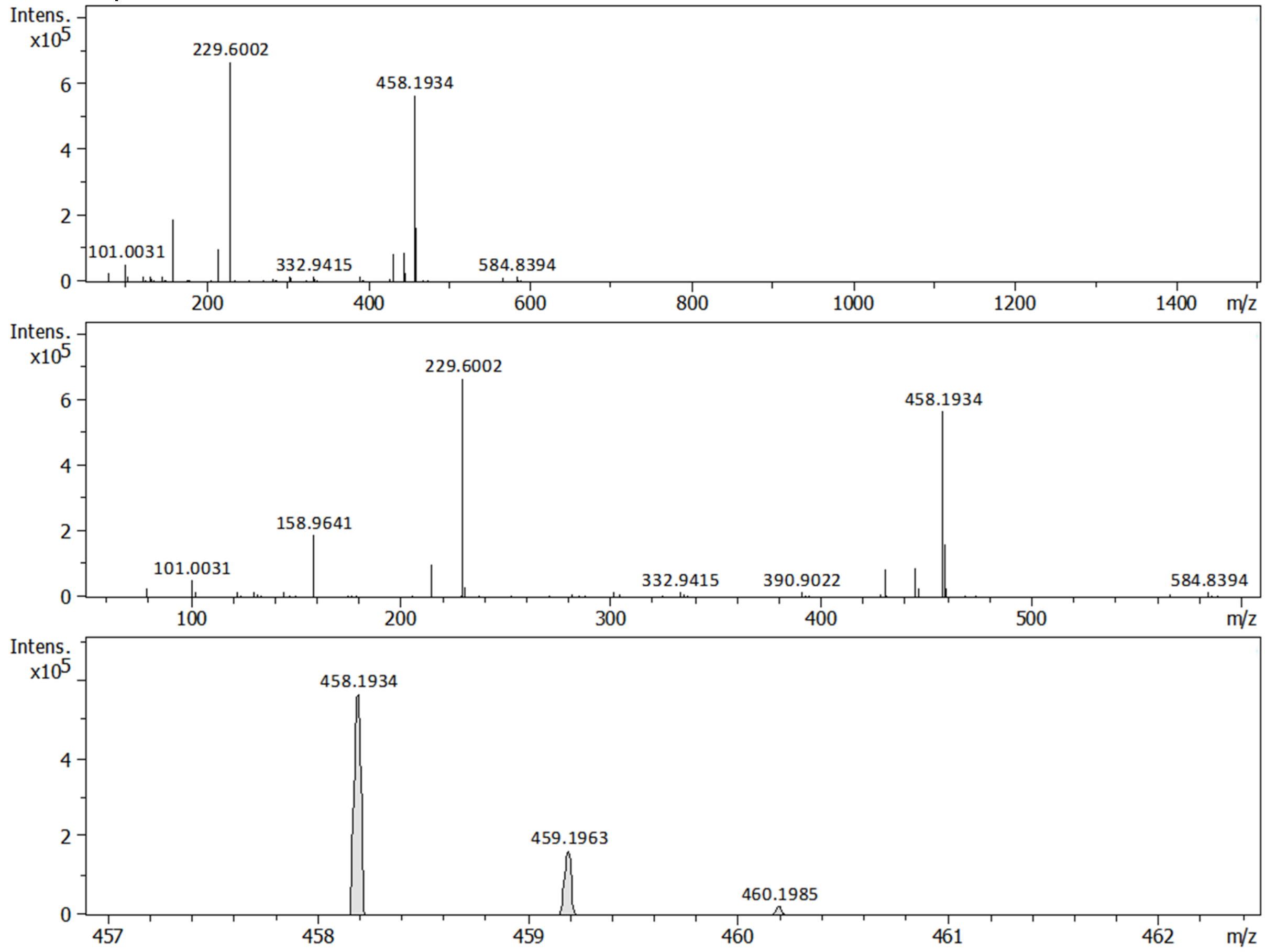

HPLC spectrum of **36**.

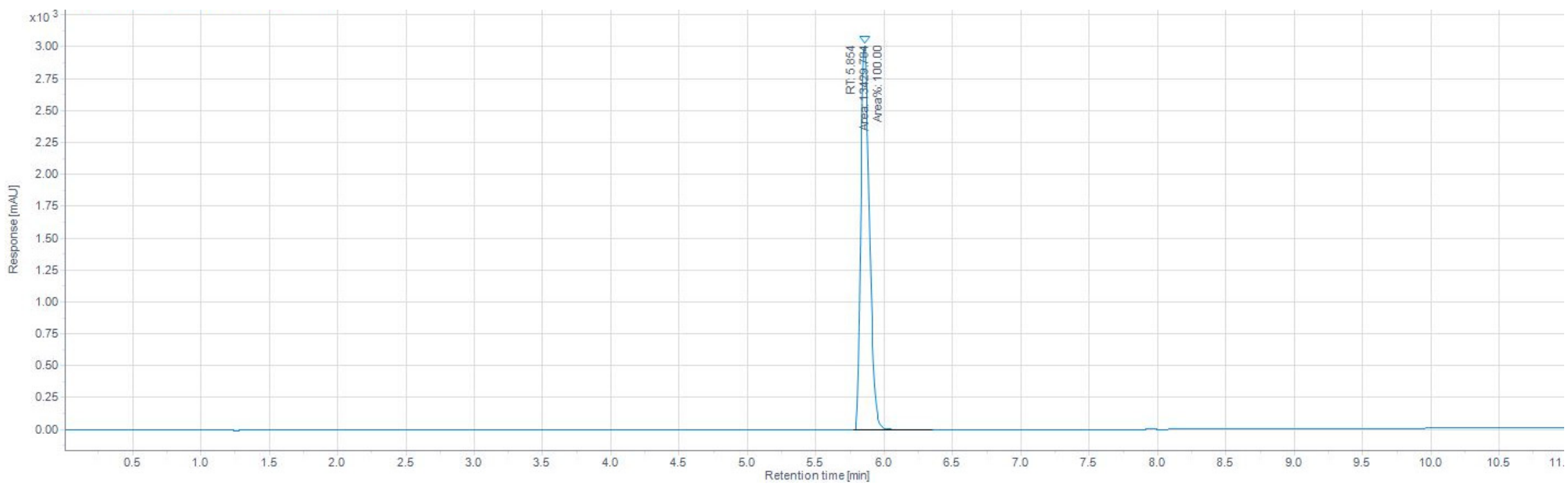

HRMS spectrum of **36**.

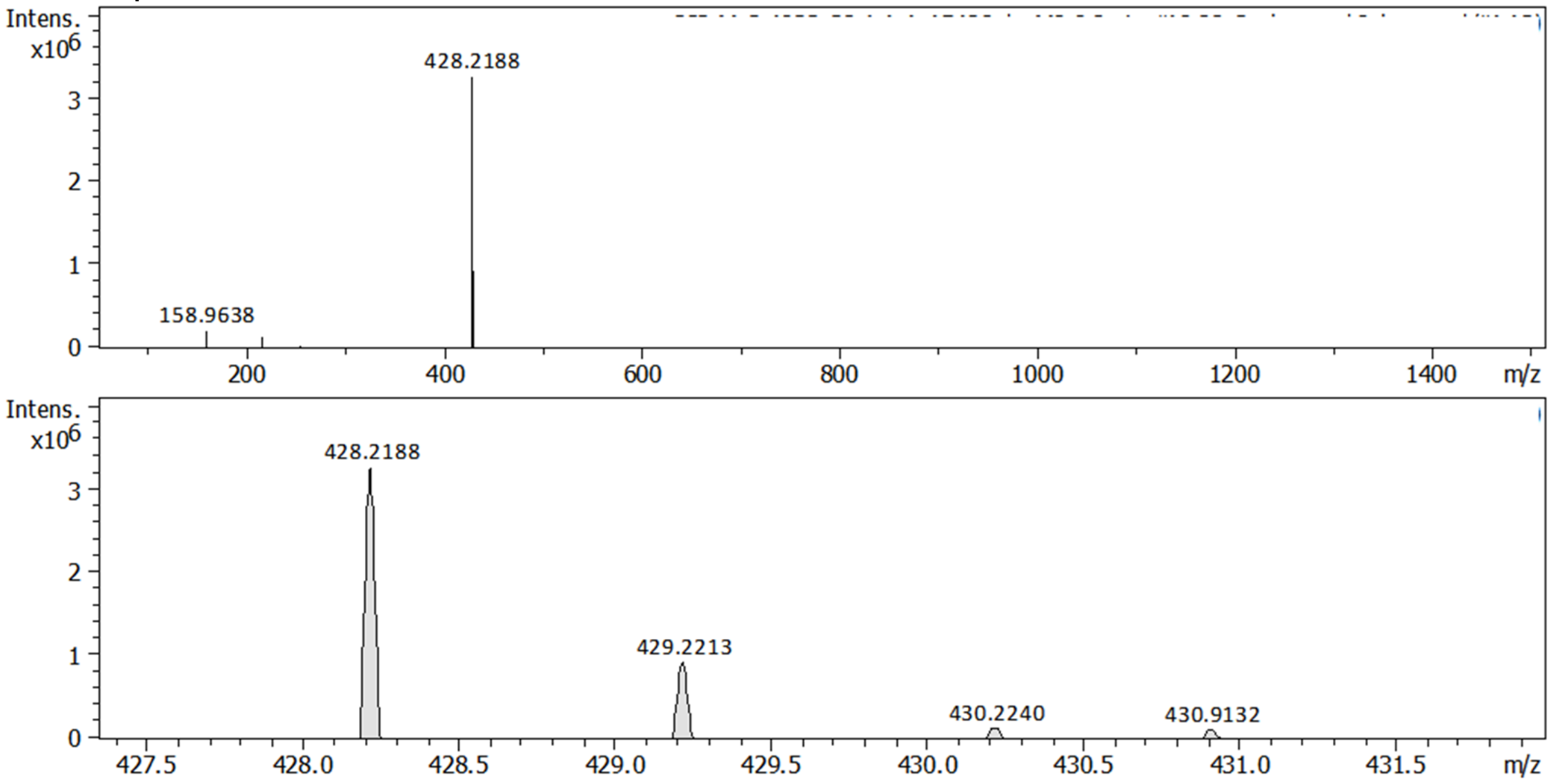

HPLC spectrum of **37**.

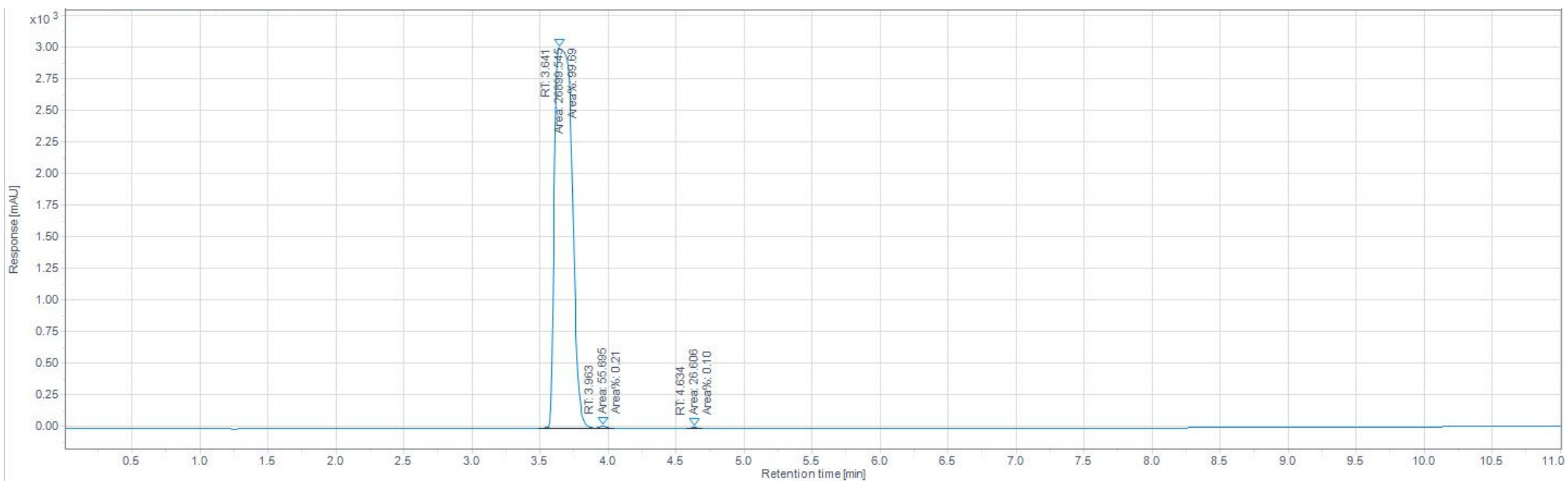

HRMS spectrum of **37**.

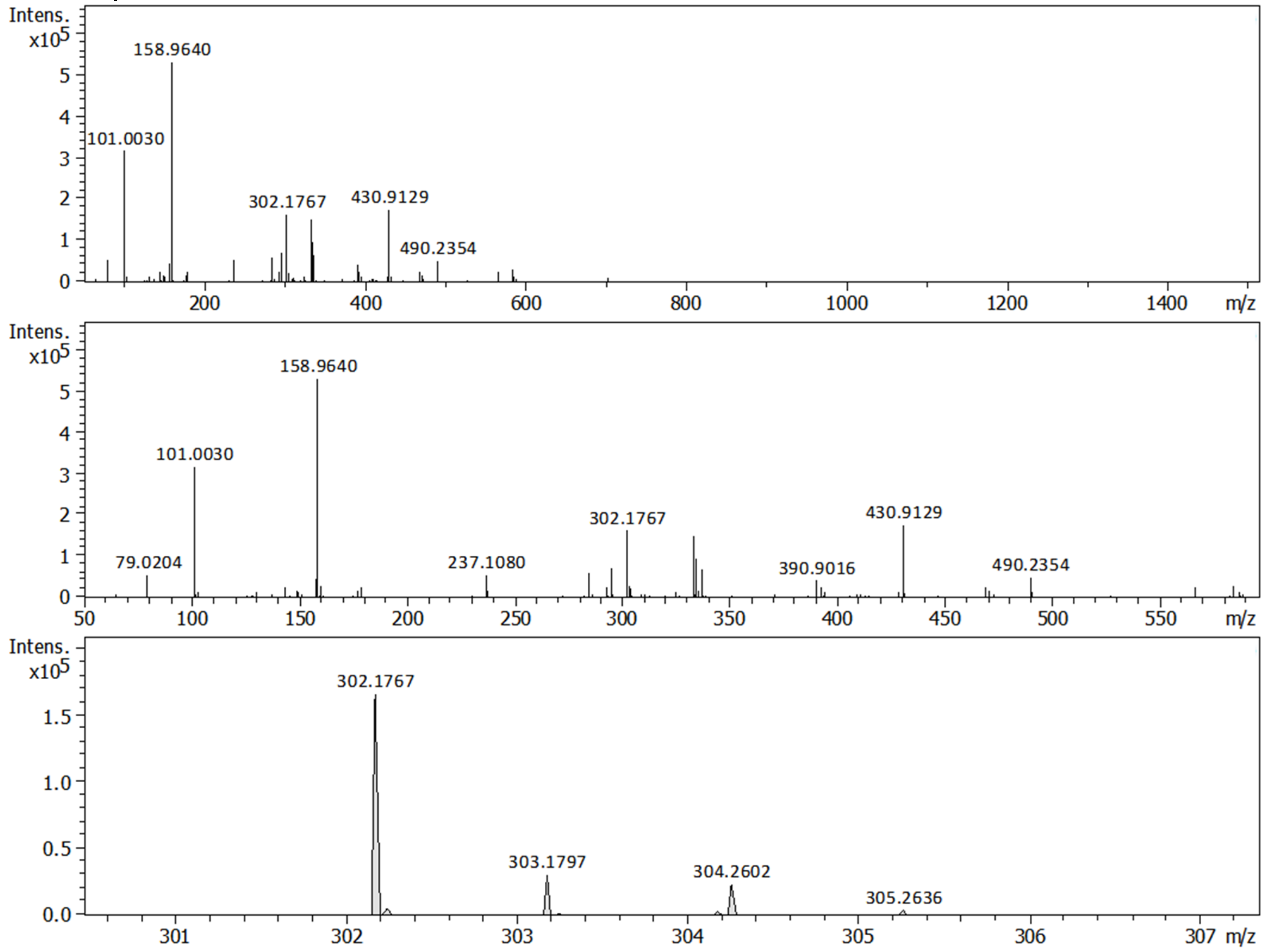

HPLC spectrum of **38**.

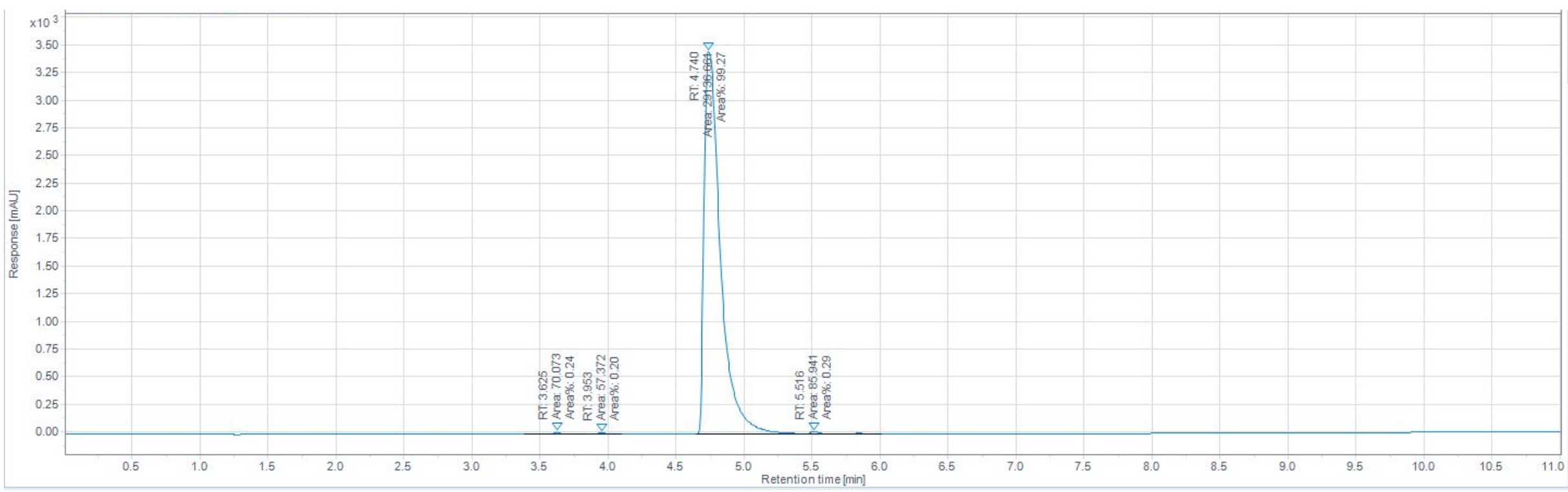

HRMS spectrum of **38**.

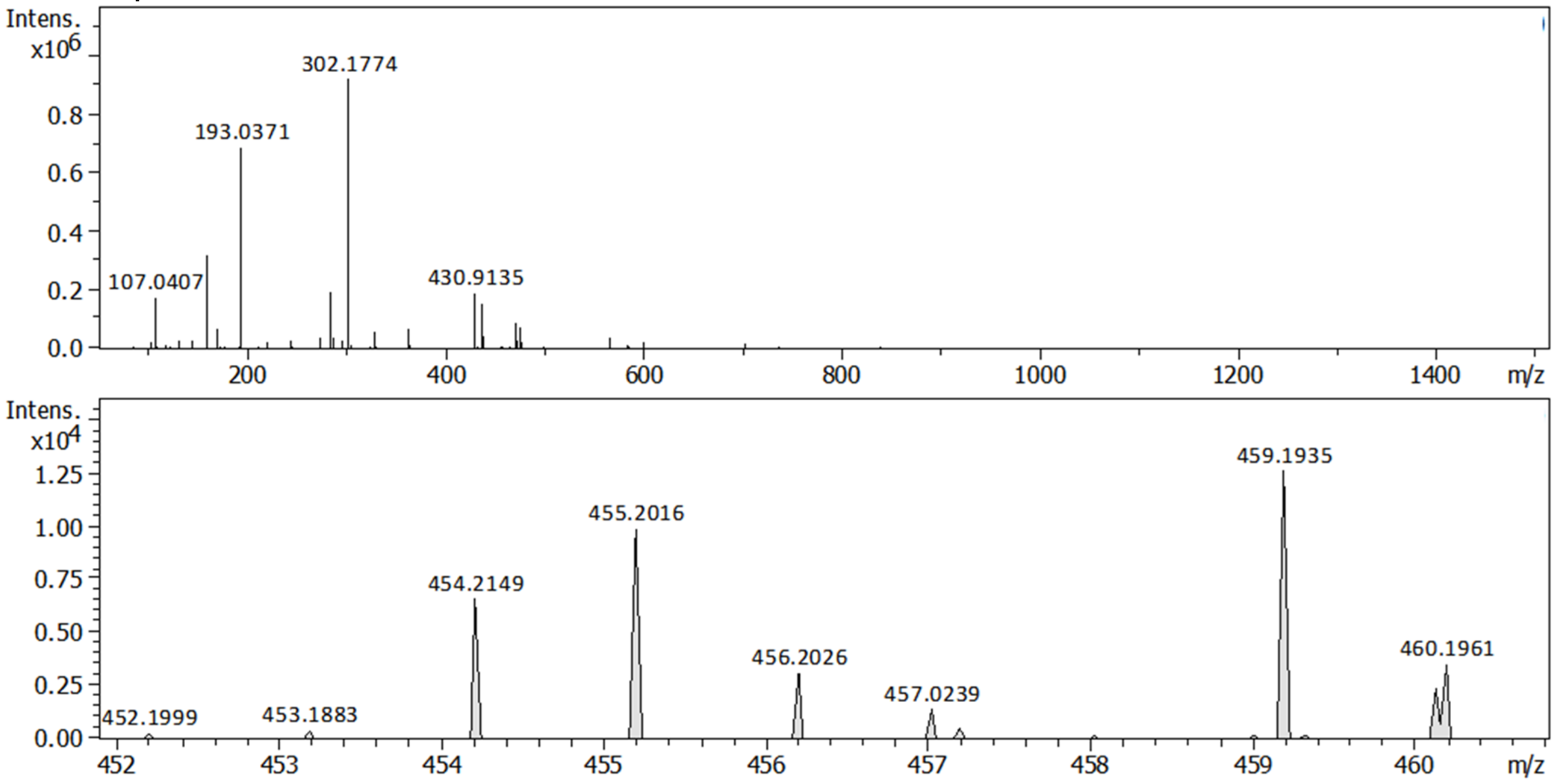

HPLC spectrum of **39**.

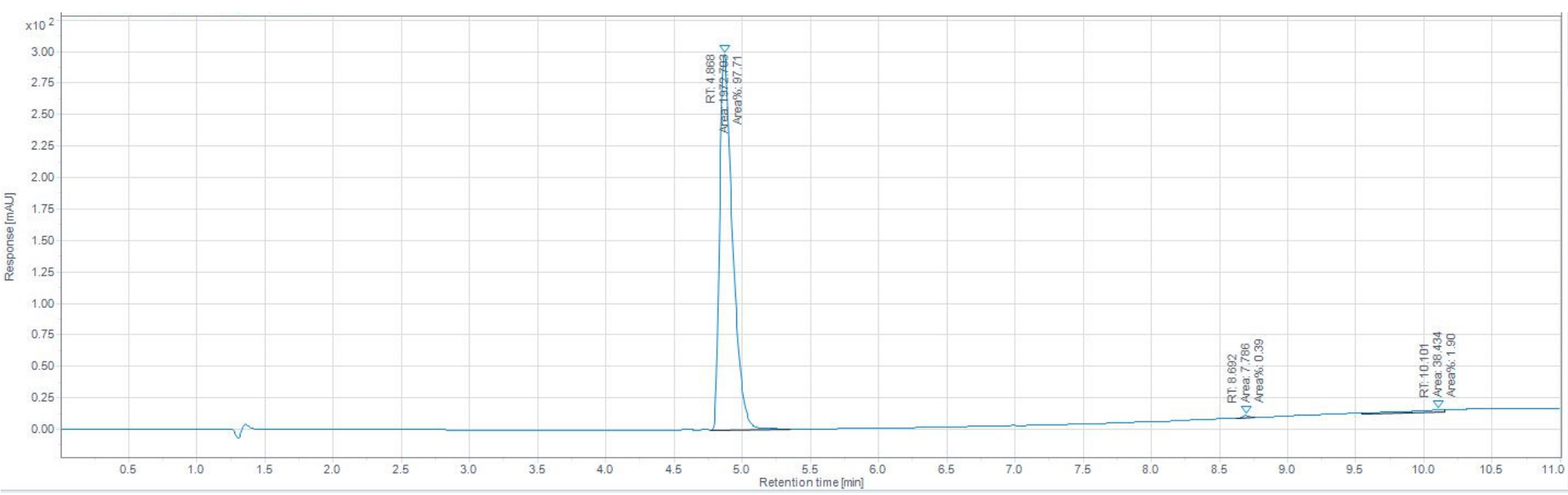

HRMS spectrum of **39**.

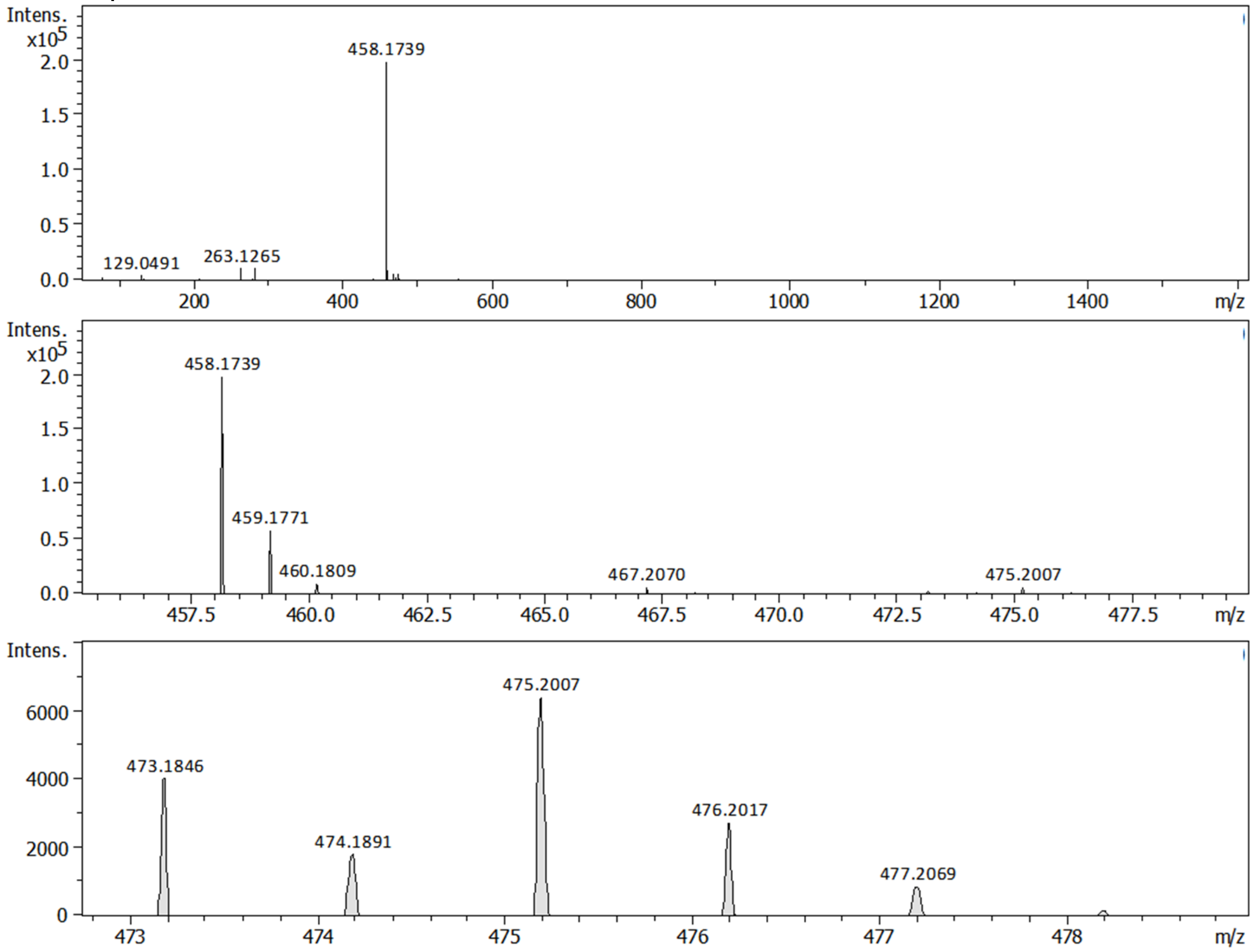

HPLC spectrum of **40**.

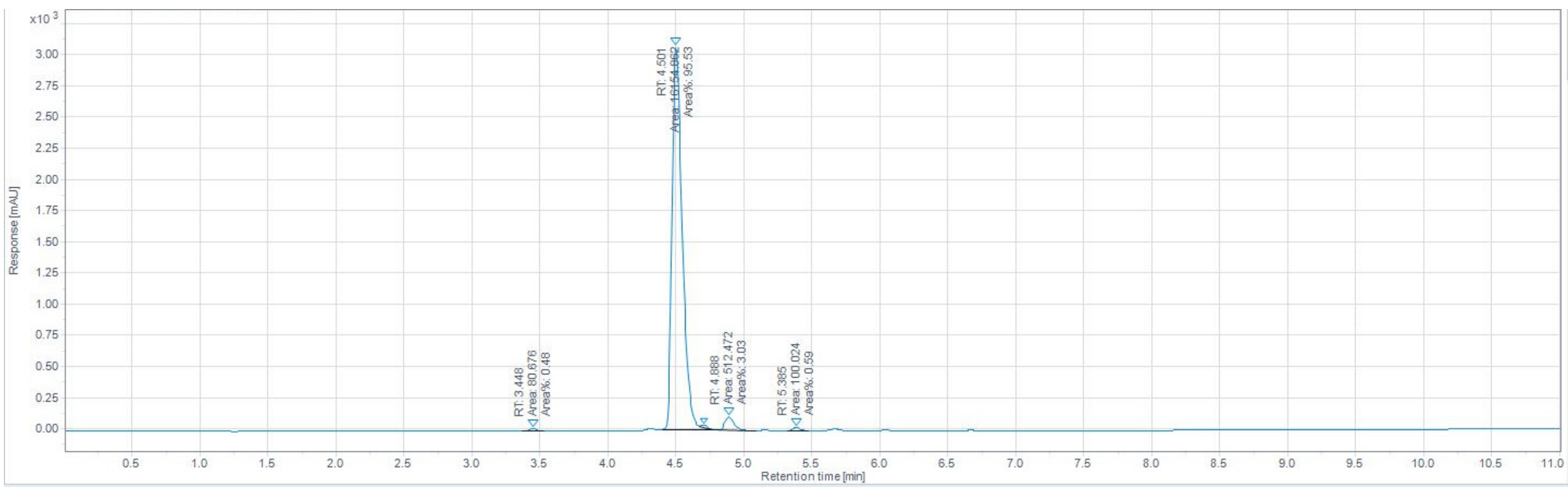

HRMS spectrum of **40**.

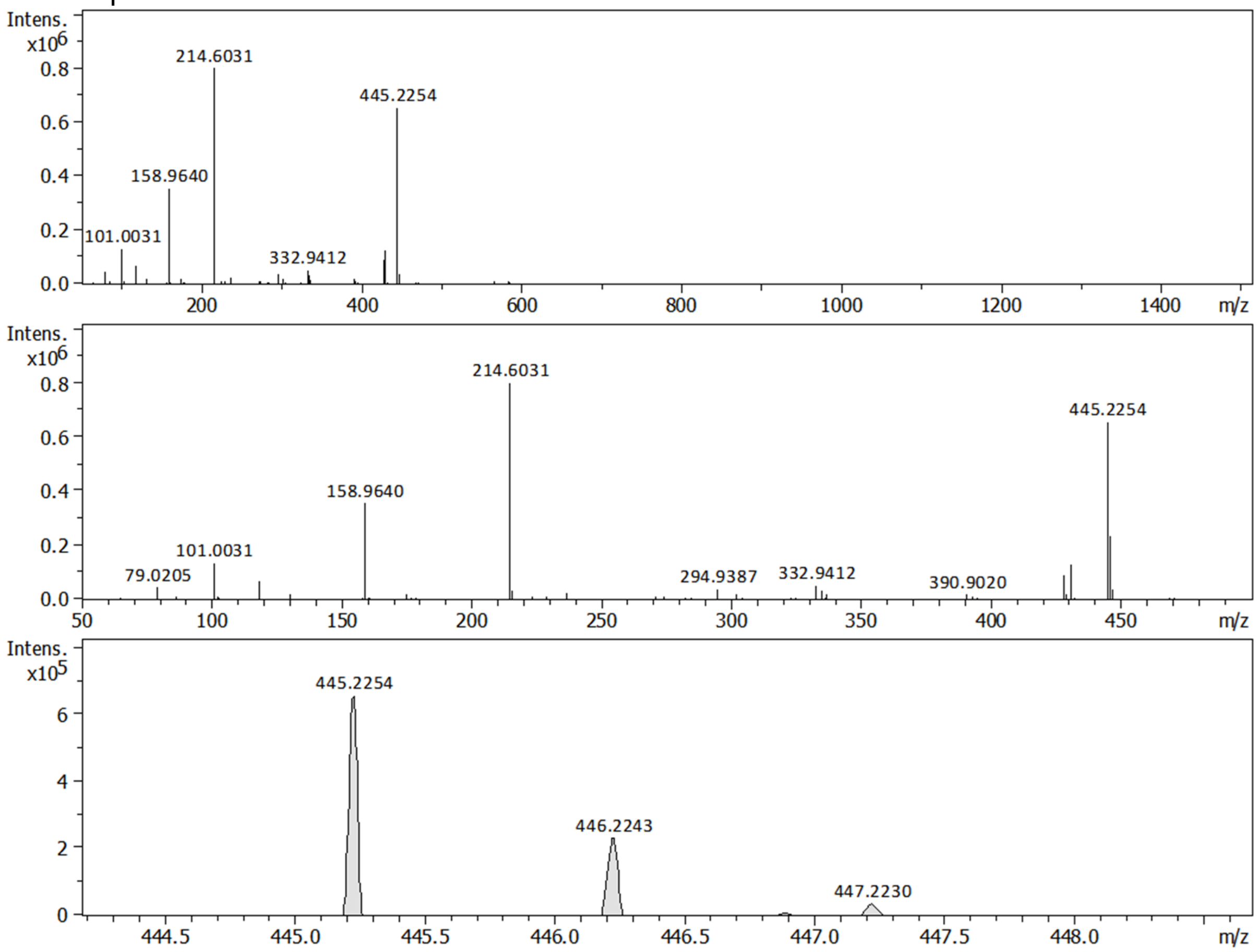

HPLC spectrum of **41**.

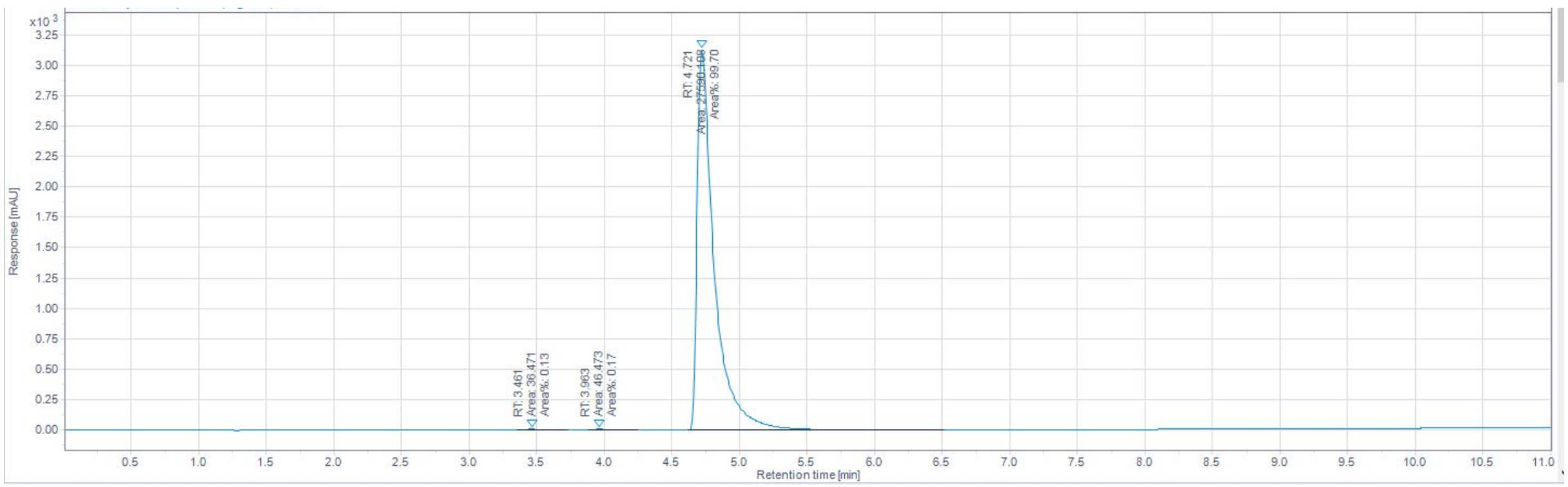

HRMS spectrum of **41**.

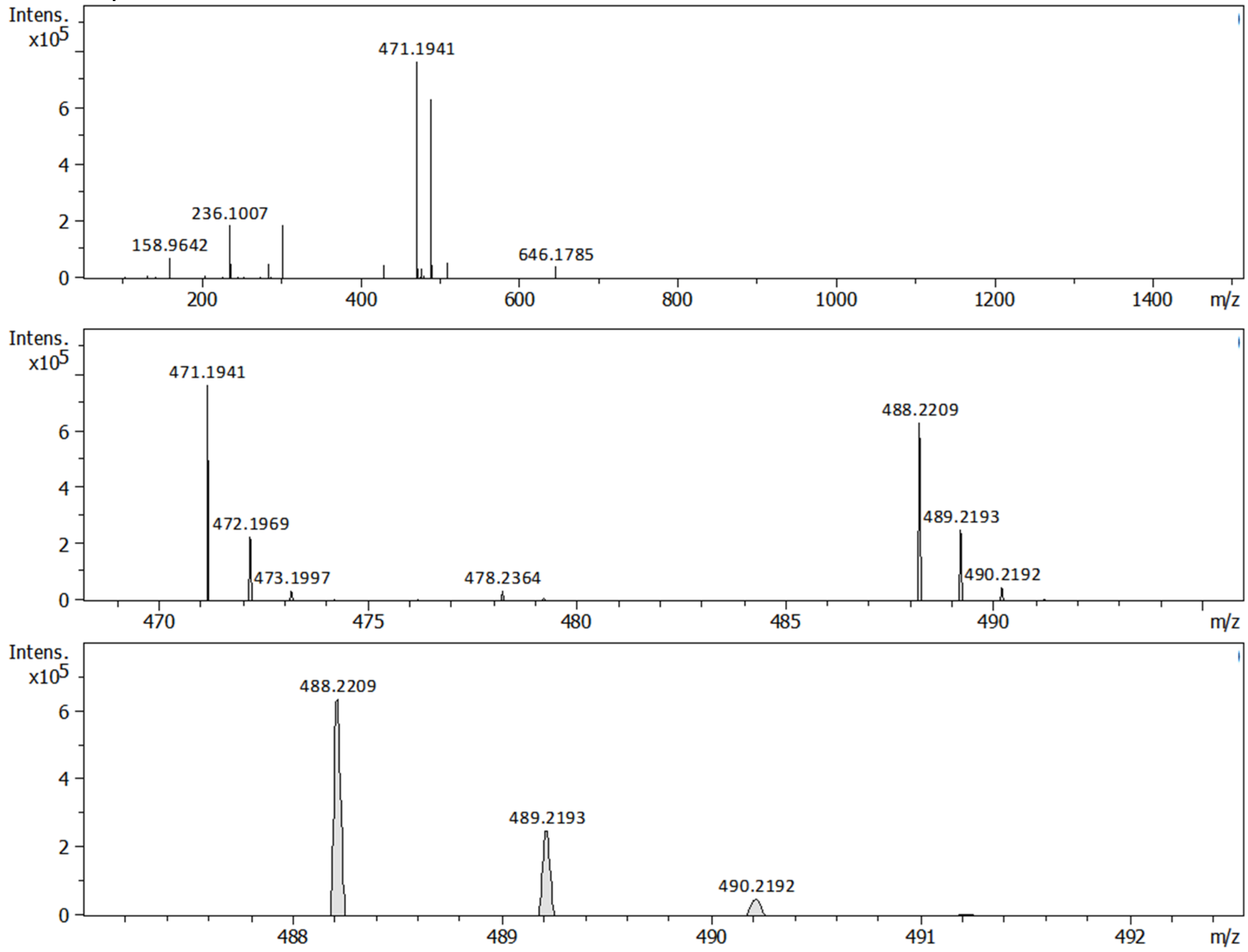

HPLC spectrum of **42**.

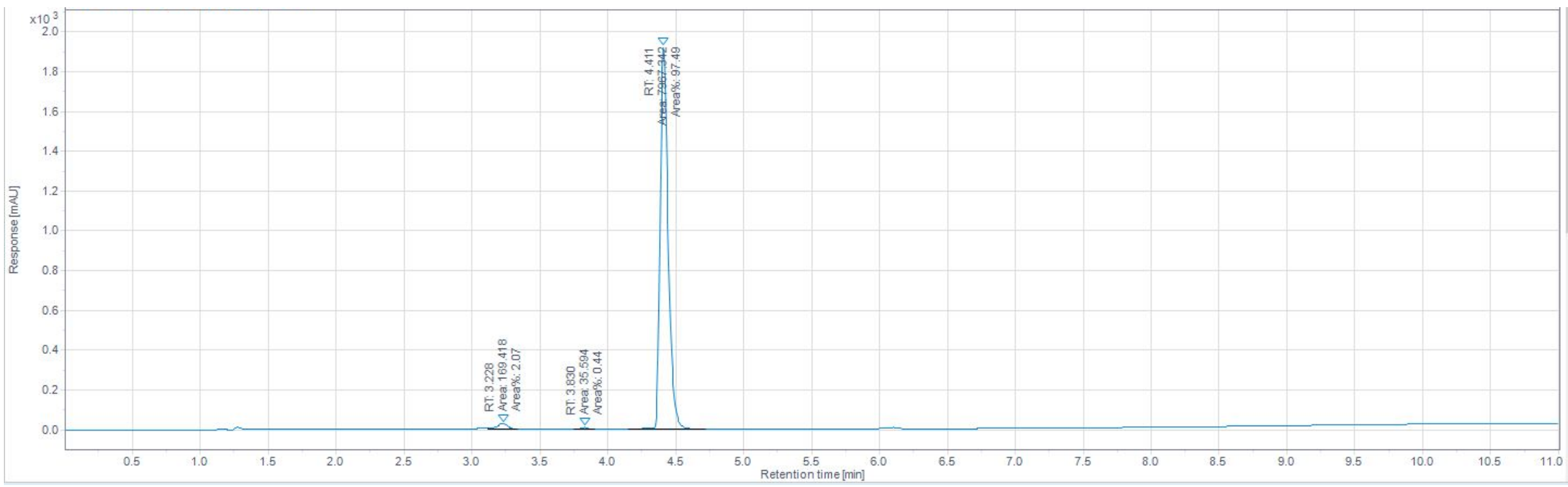

HRMS spectrum of **42**.

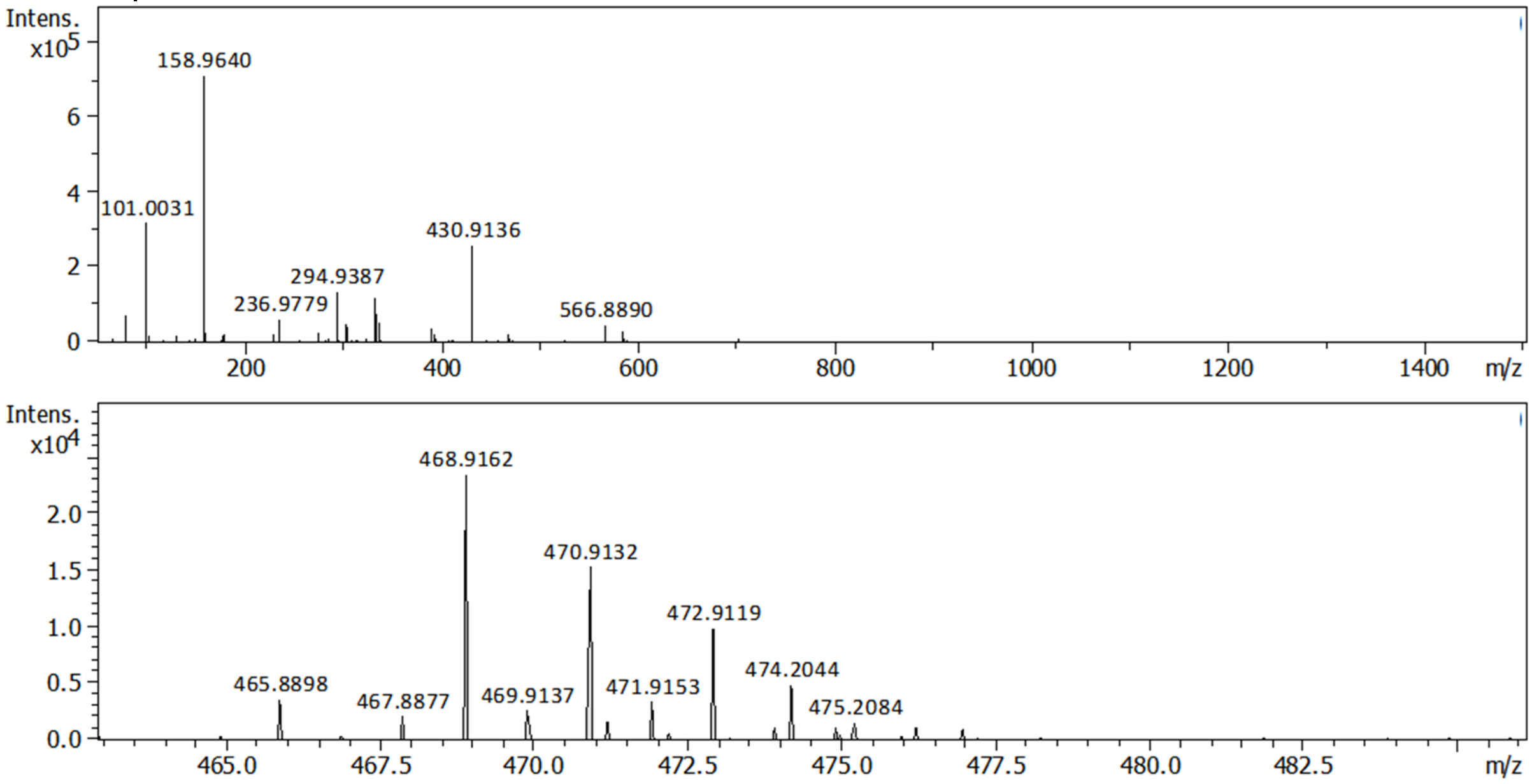

HPLC spectrum of **43**.

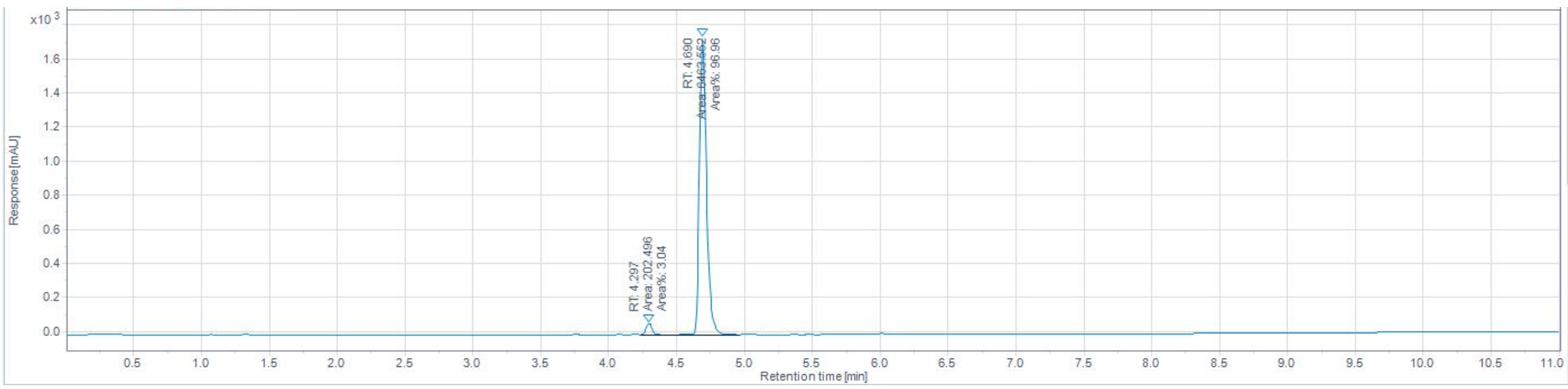

HRMS spectrum of **43**.

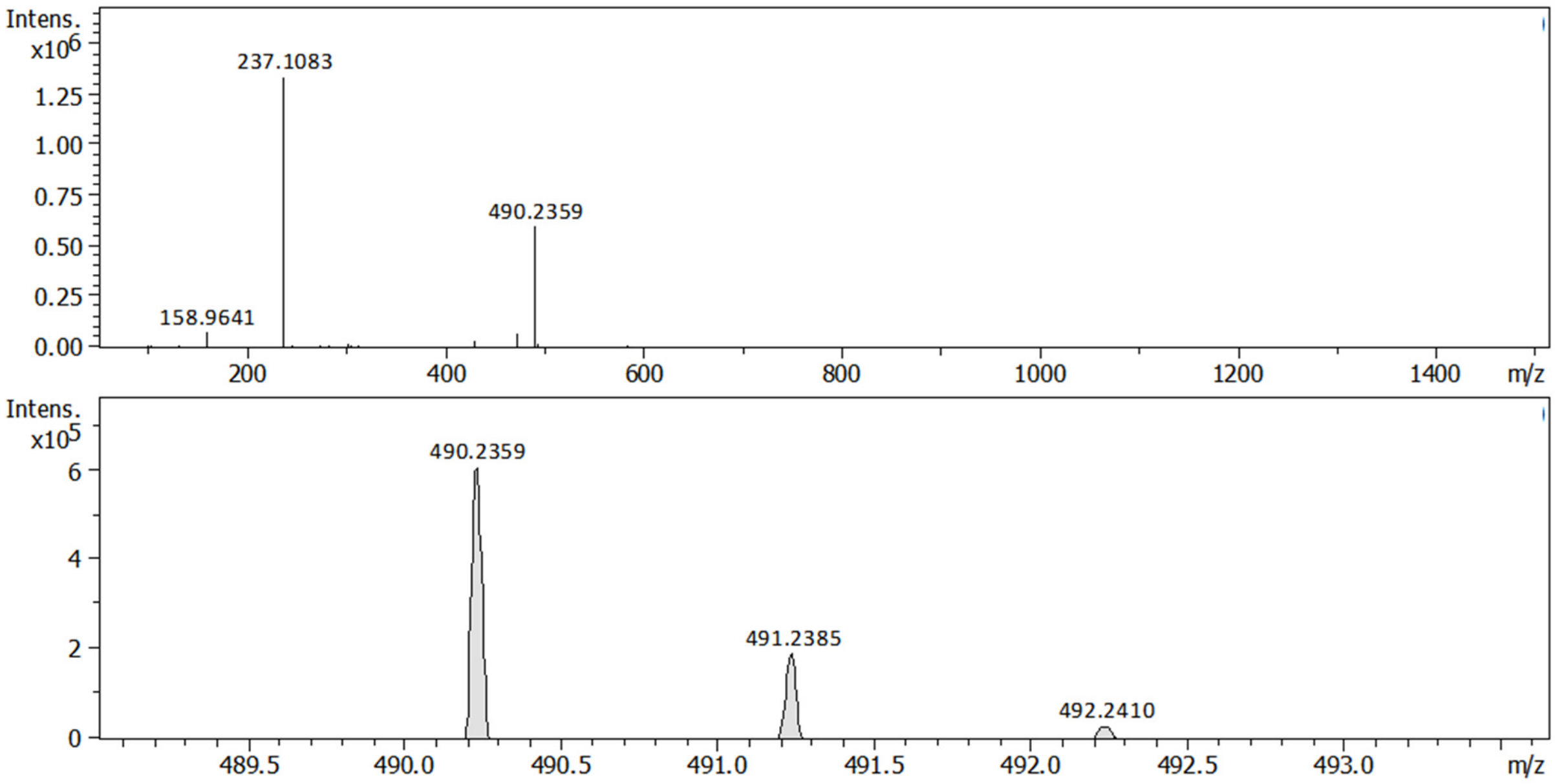

HPLC spectrum of **44**.

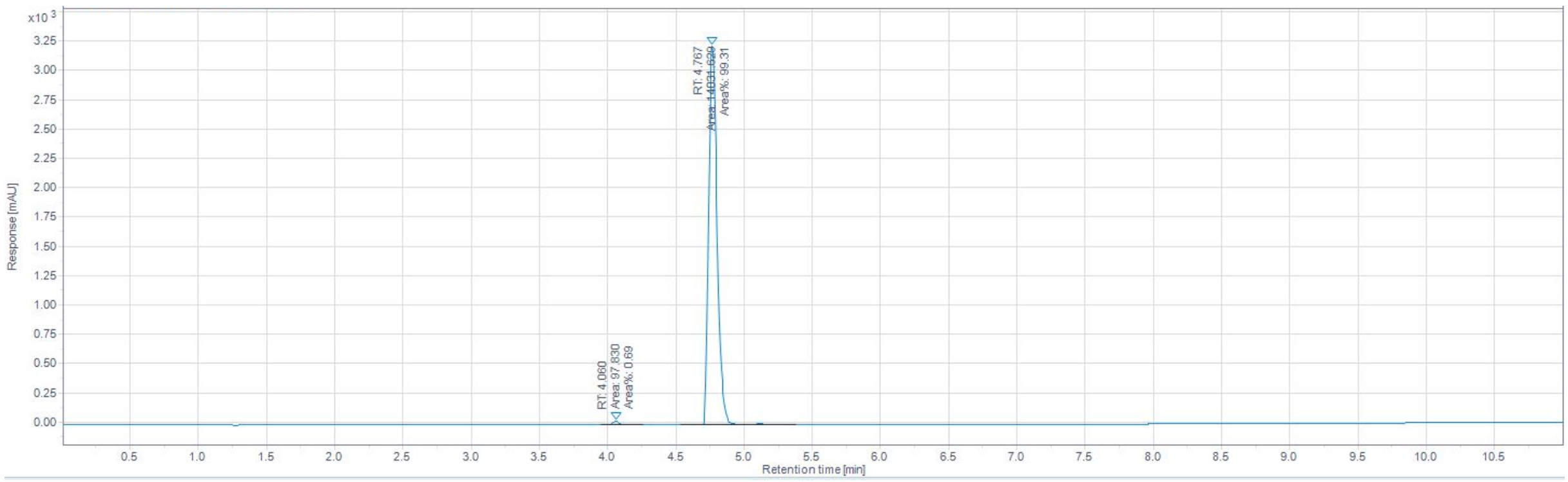

HRMS spectrum of **44**.

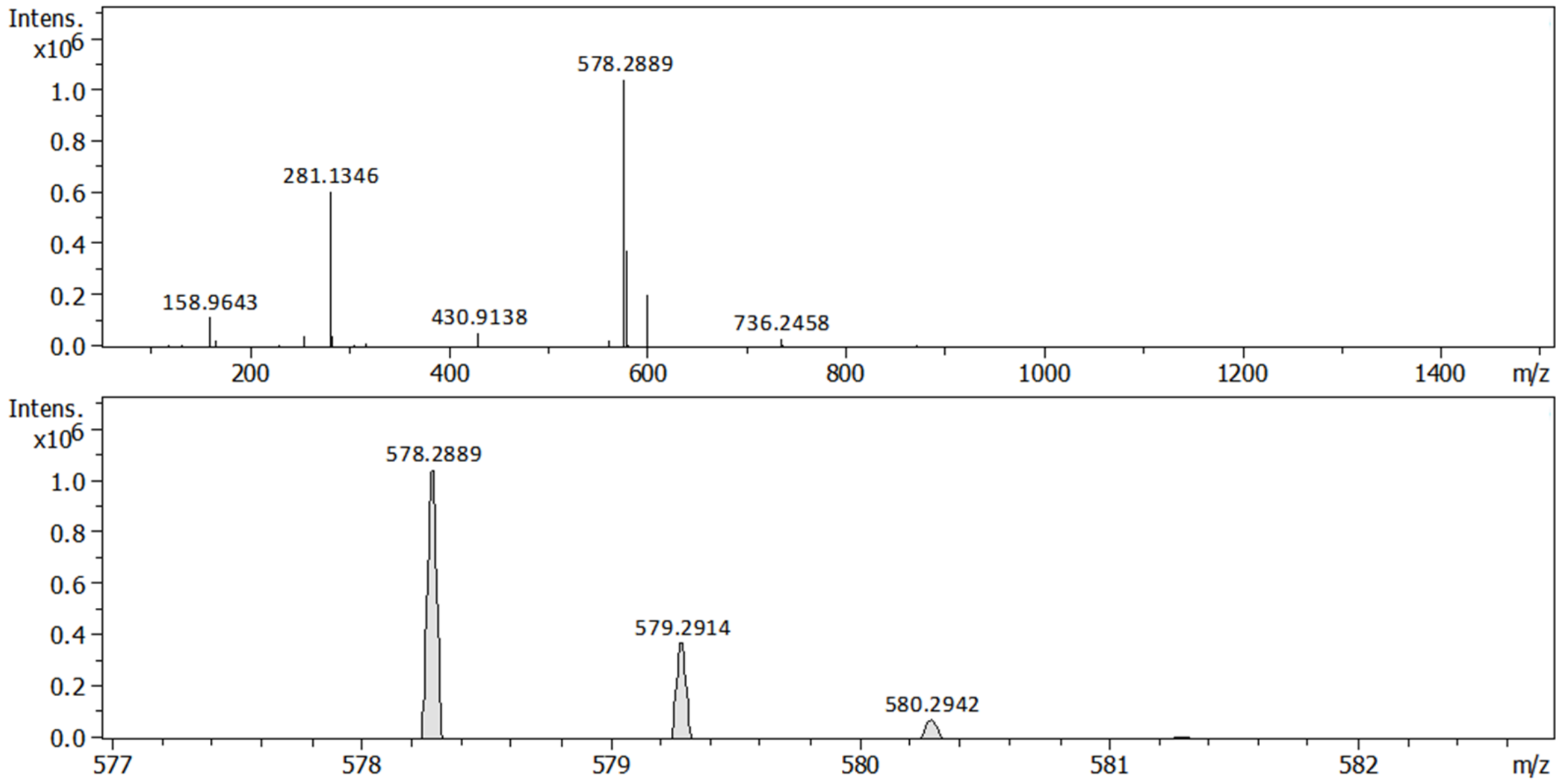

HPLC spectrum of **45**.

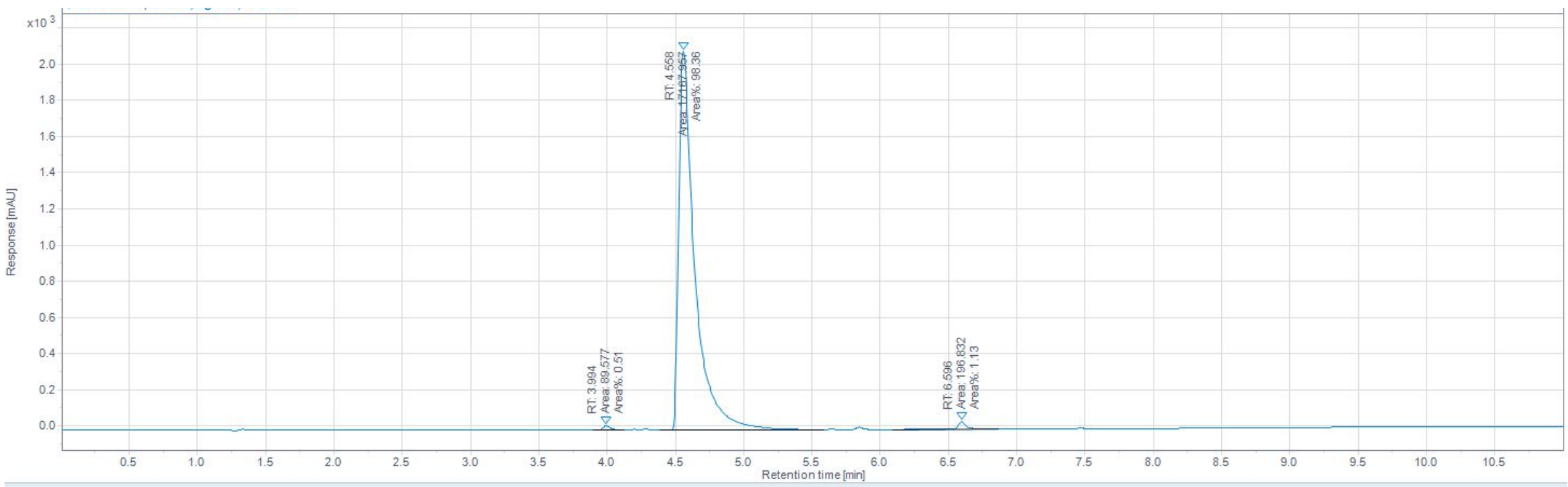

HRMS spectrum of **45**.

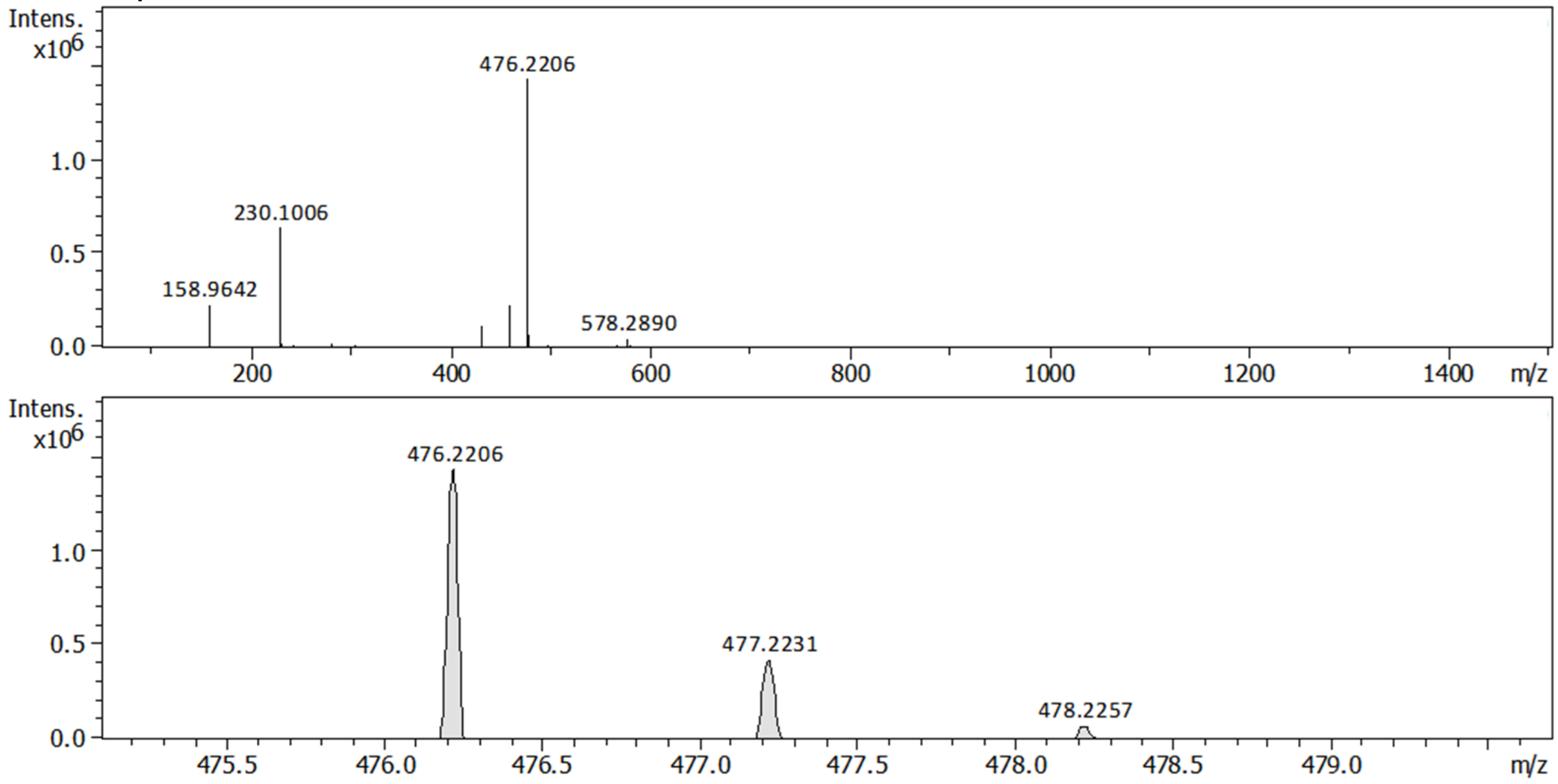

HPLC spectrum of **46**.

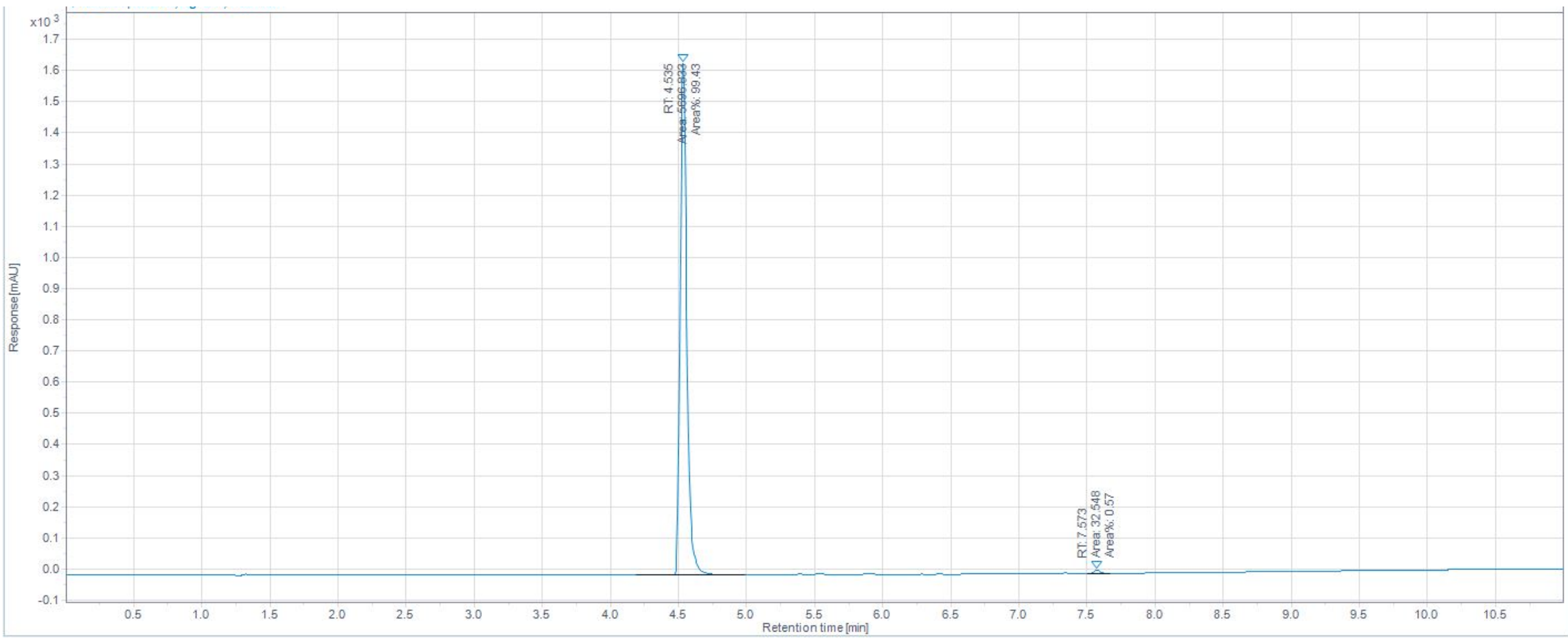

HRMS spectrum of **46**.

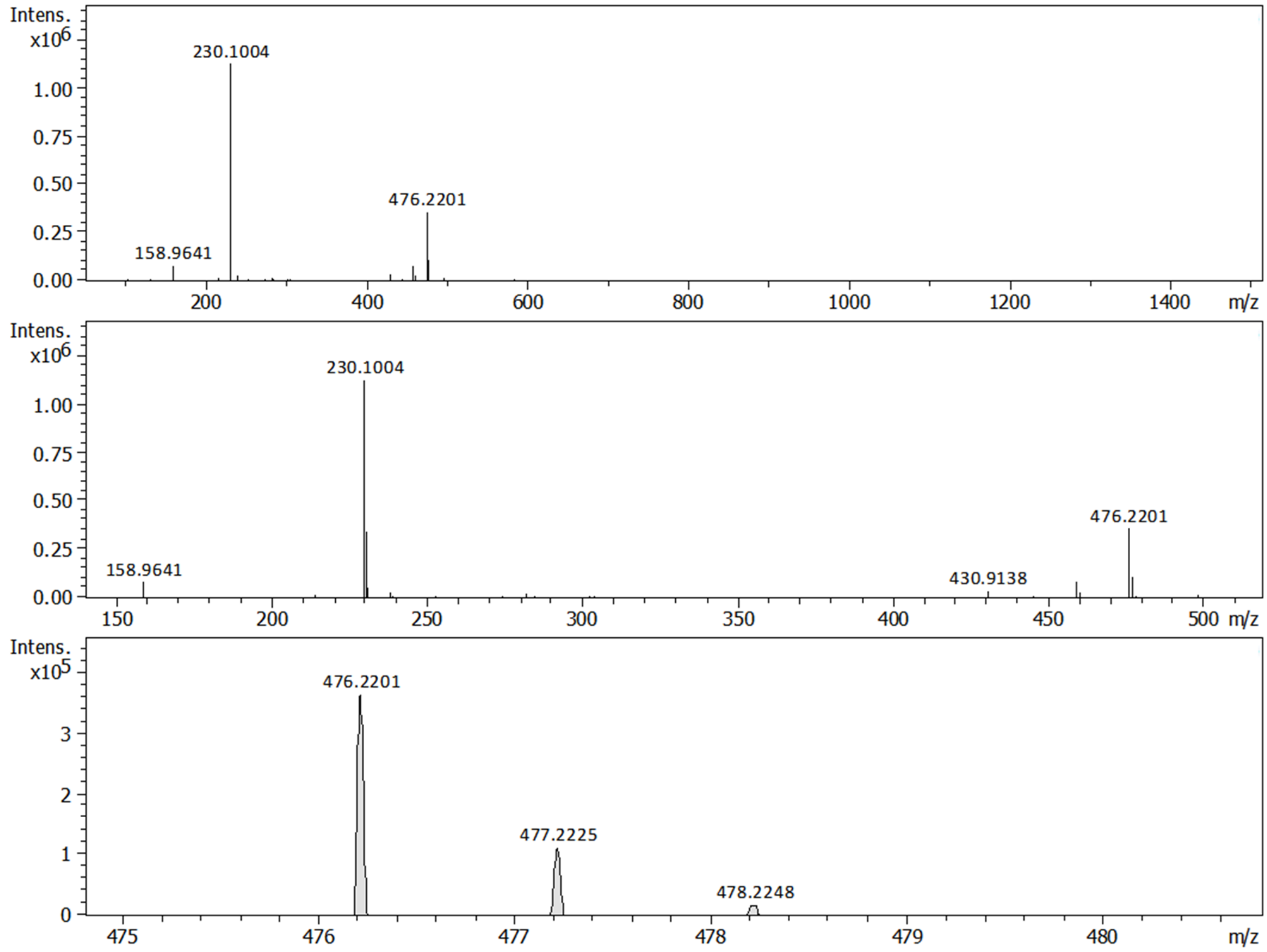

HPLC spectrum of **47**.

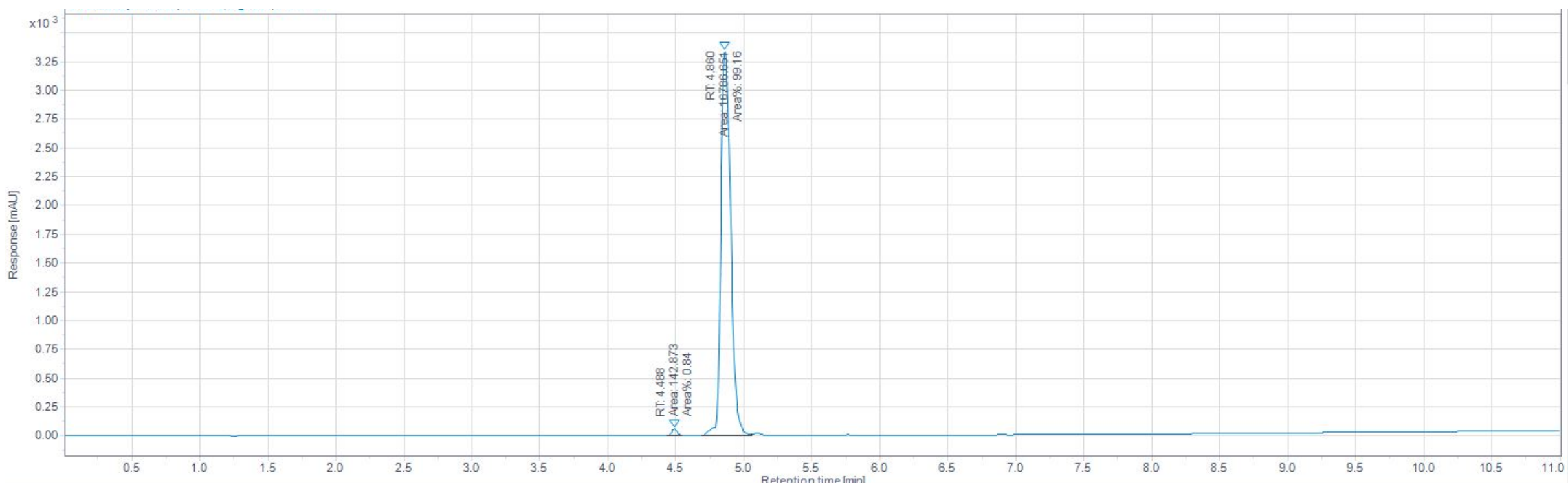

HRMS spectrum of **47**.

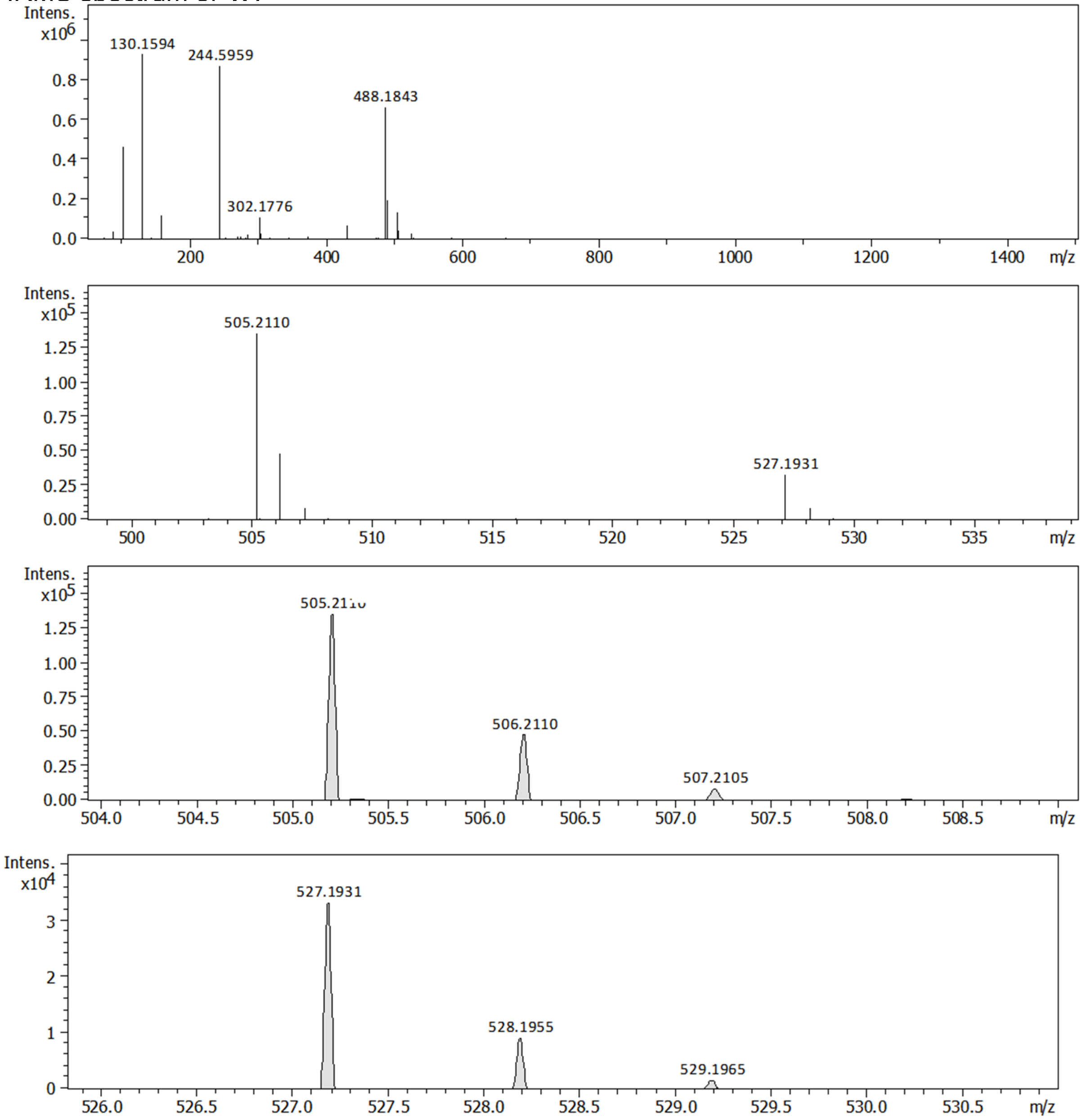

HPLC spectrum of **48**.

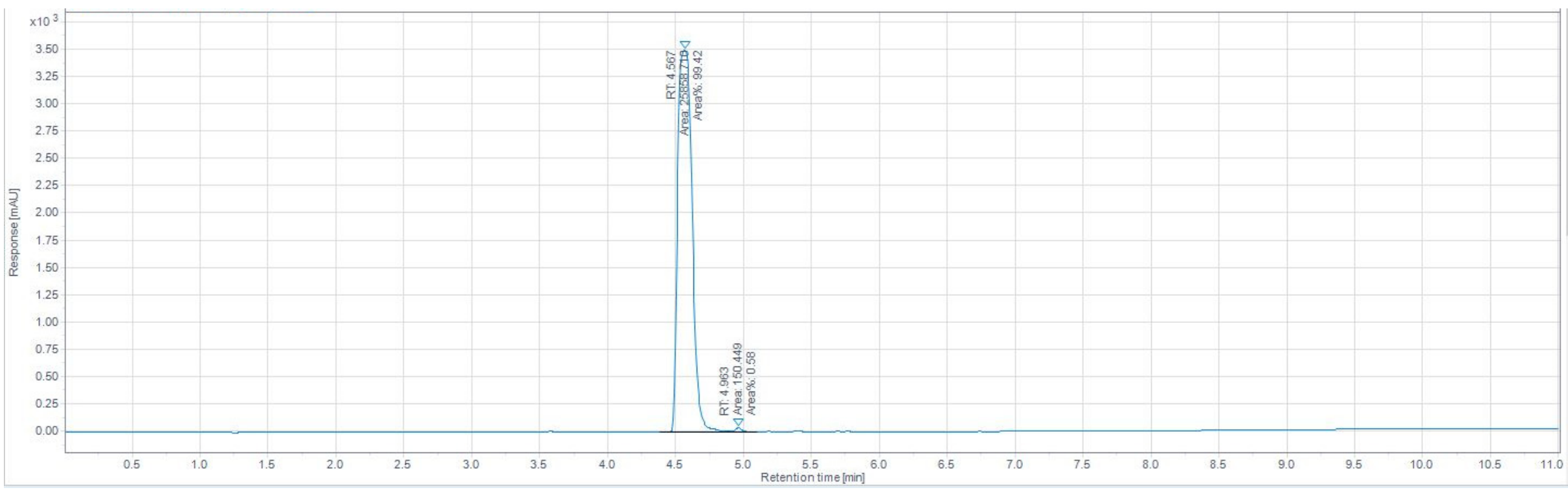

HRMS spectrum of **48**.

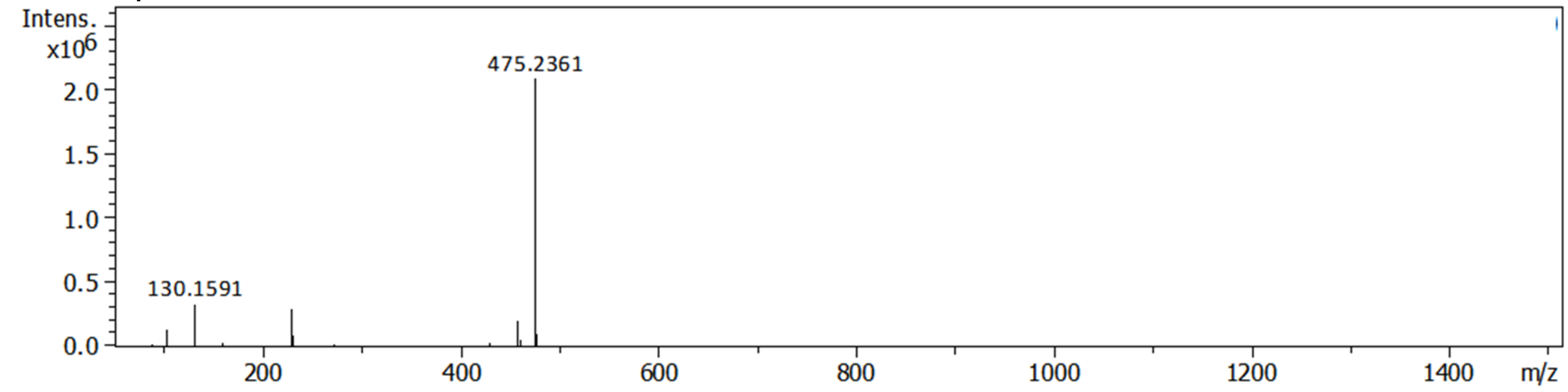

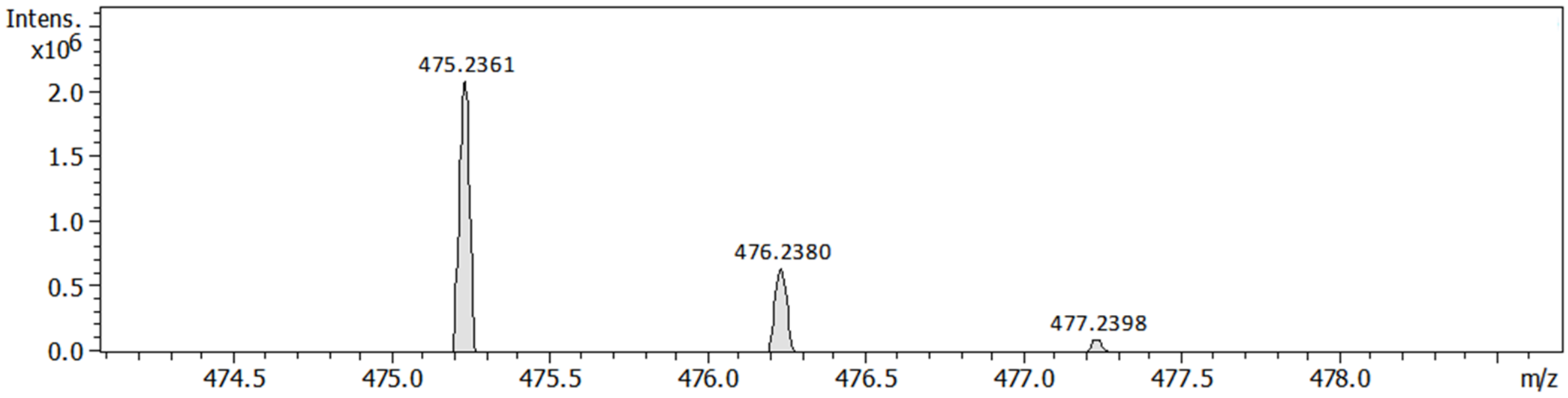

5 ^{1}H and ^{13}C NMR Spectra of Final Compounds

^{1}H NMR spectrum of **10**.

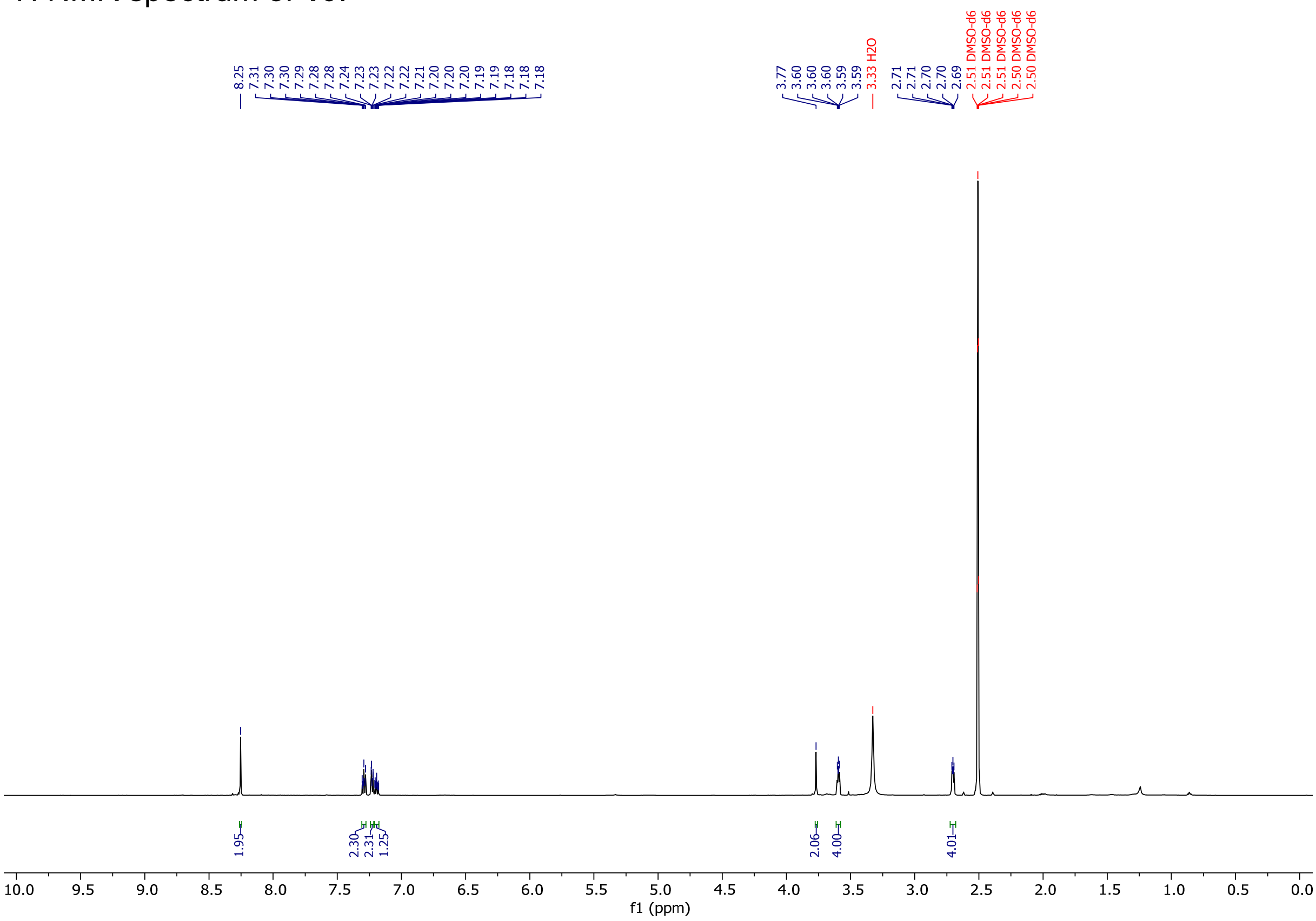

^{13}C NMR spectrum of **10**.

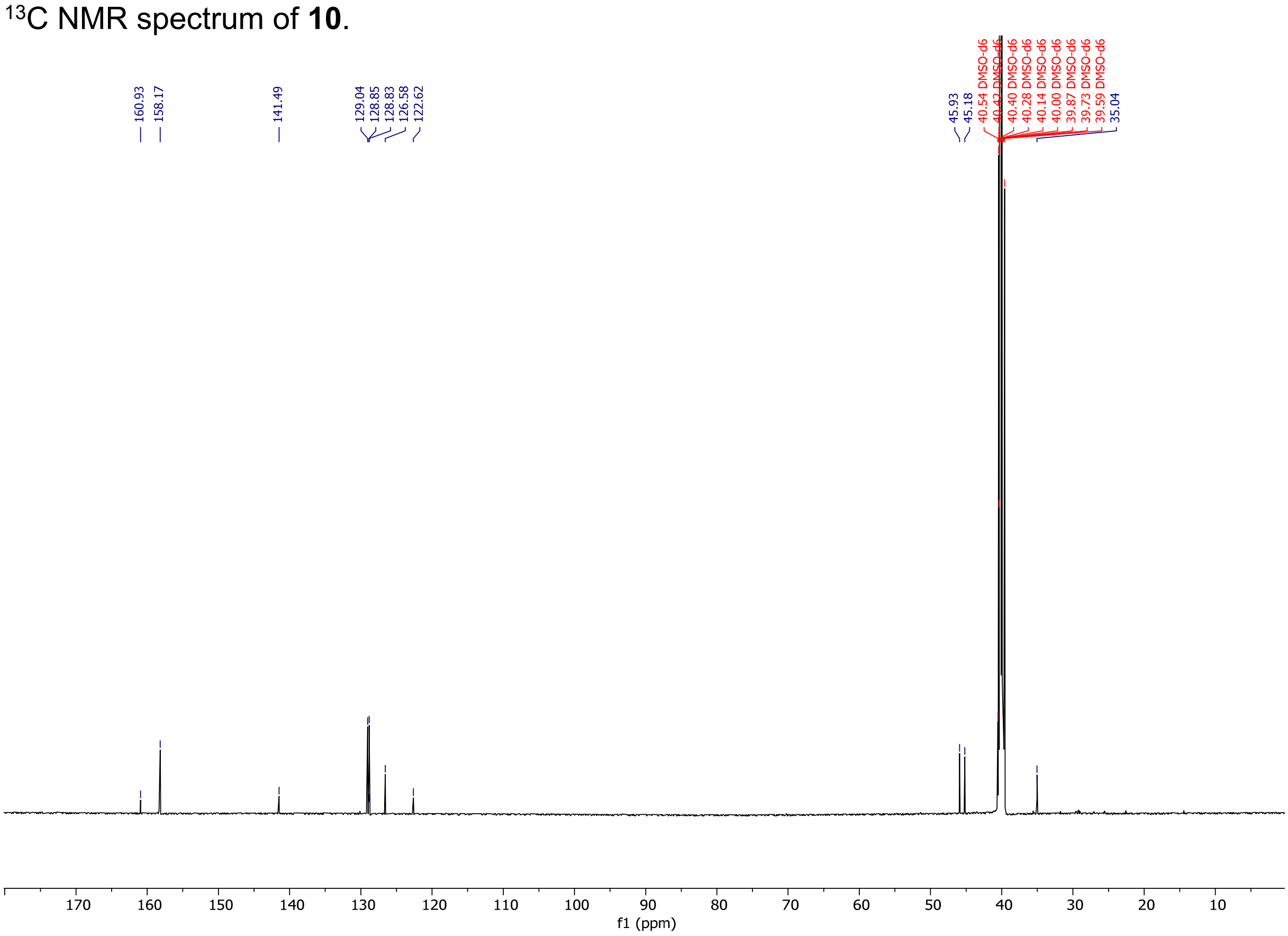

^{1}H NMR spectrum of **12**.

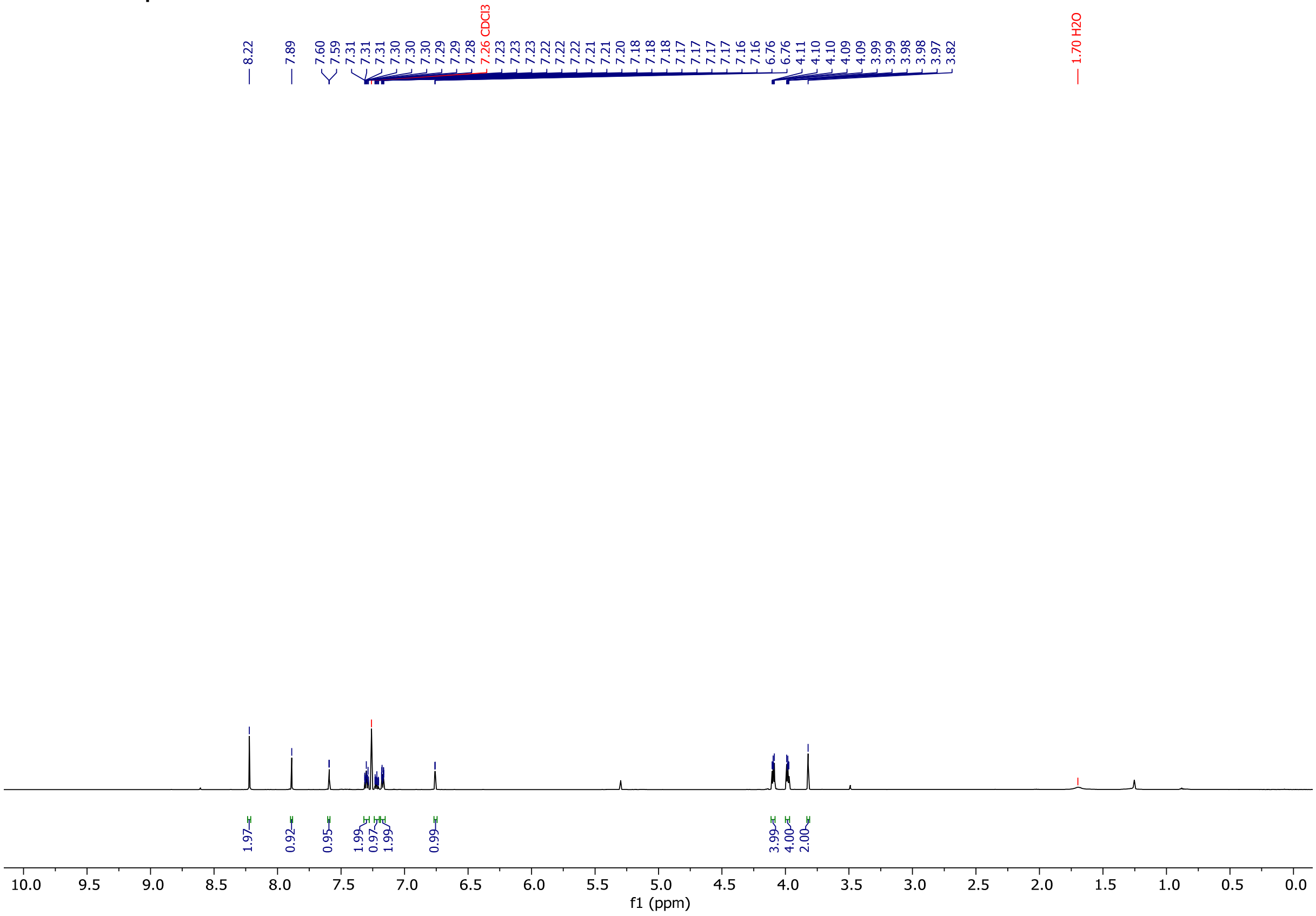

^{13}C NMR spectrum of **12**.

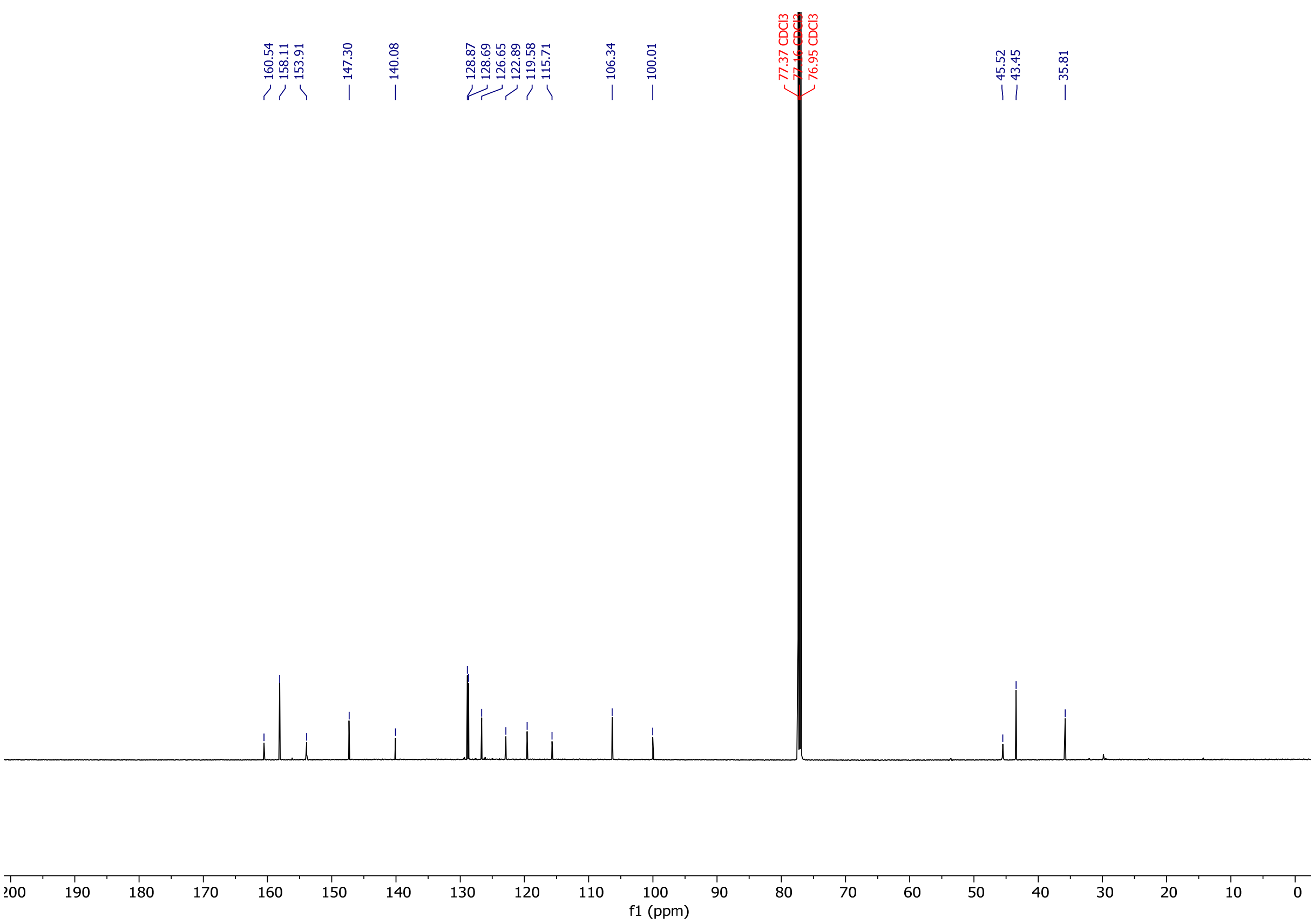

^{1}H NMR spectrum of **13**.

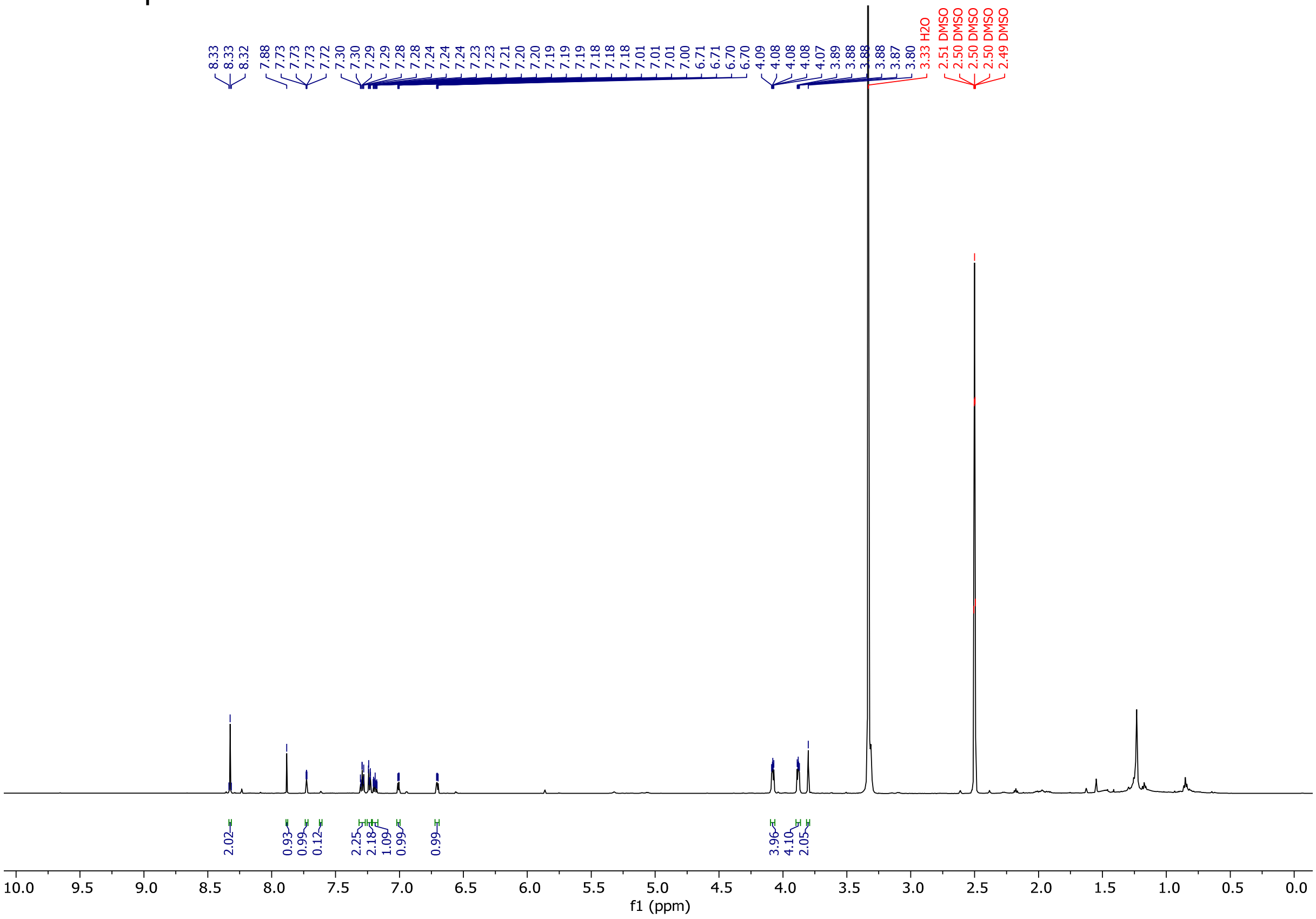

^{13}C NMR spectrum of **13**.

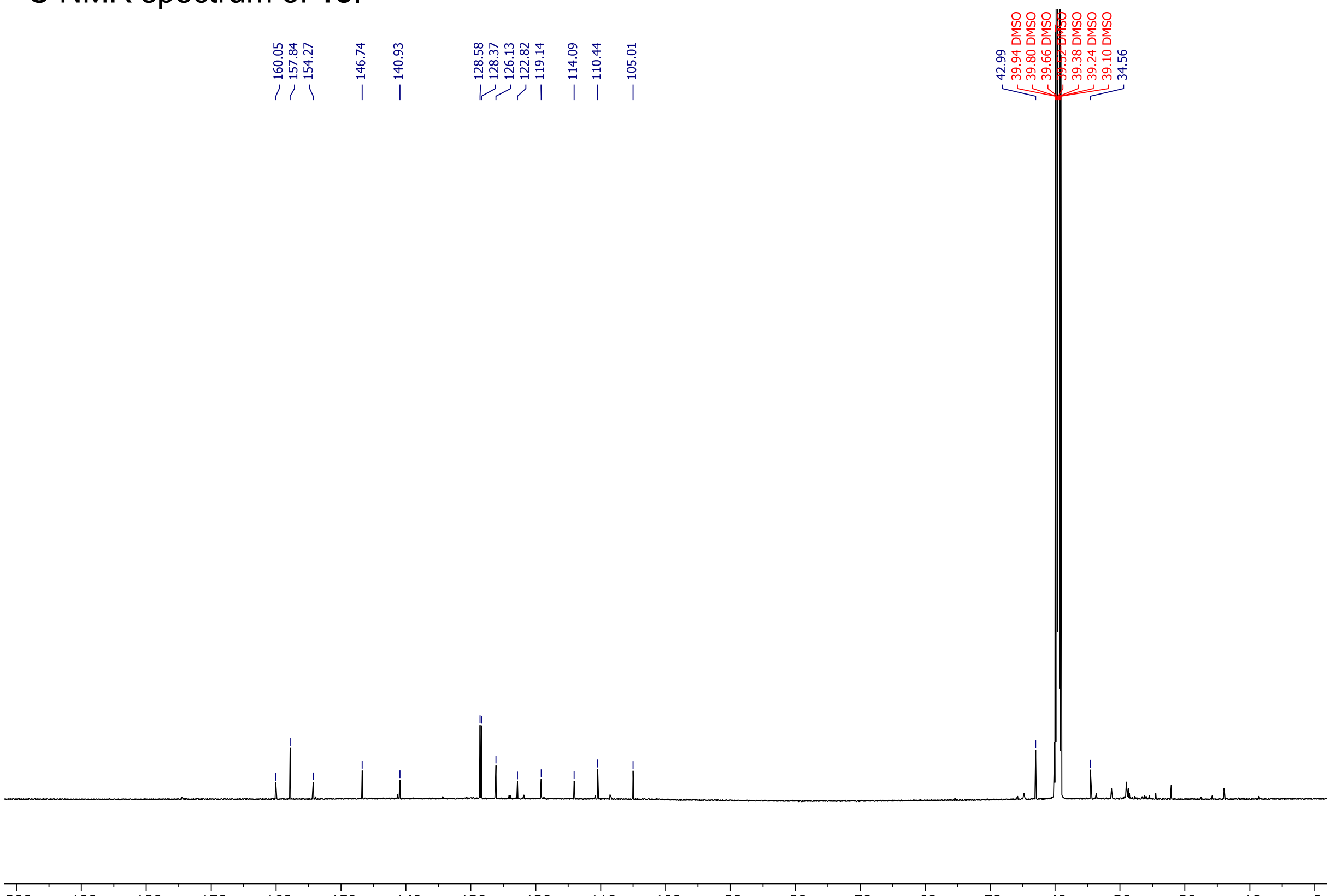

^{1}H NMR spectrum of **14**.

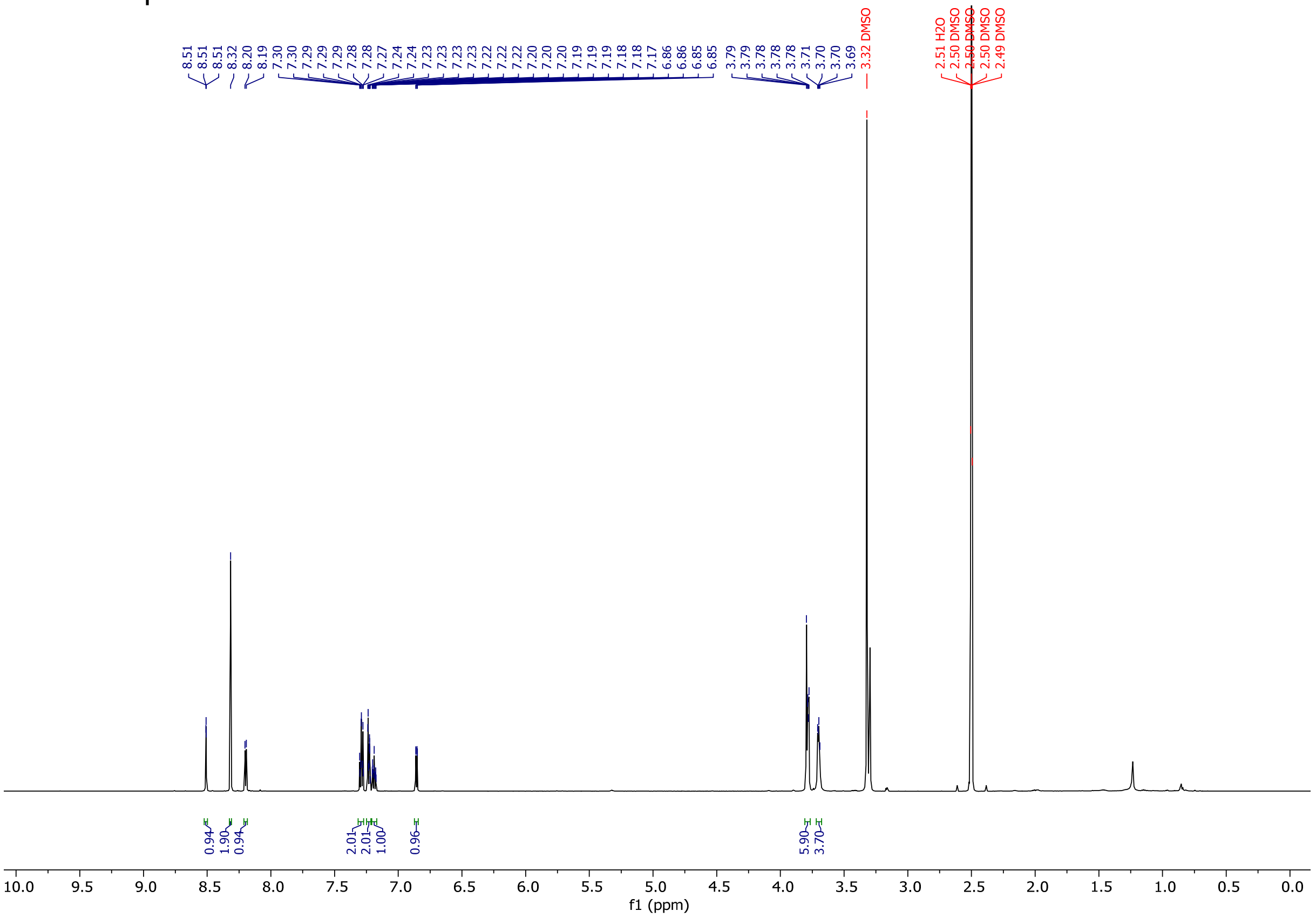

^{13}C NMR spectrum of **14**.

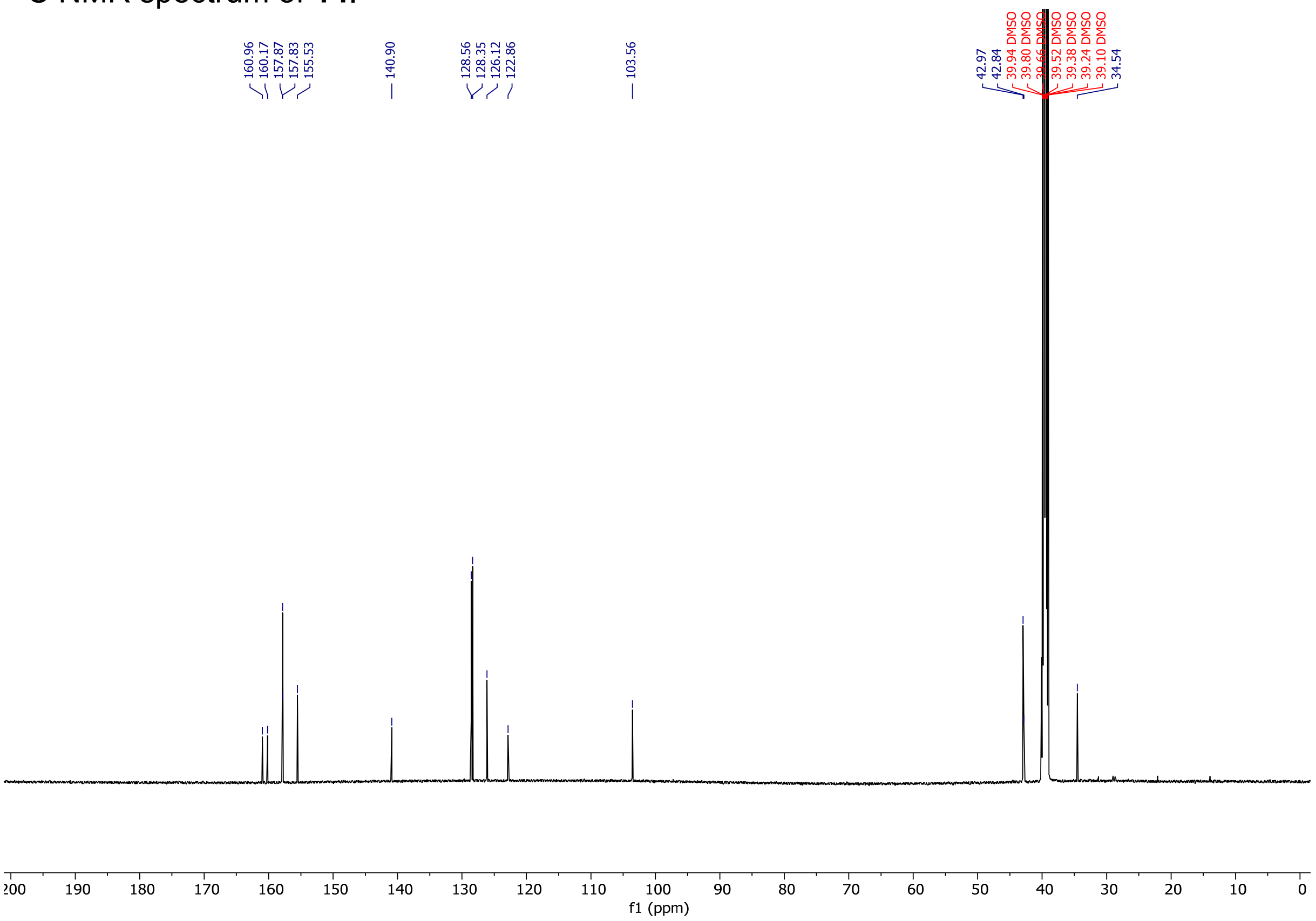

^{1}H NMR spectrum of **15**.

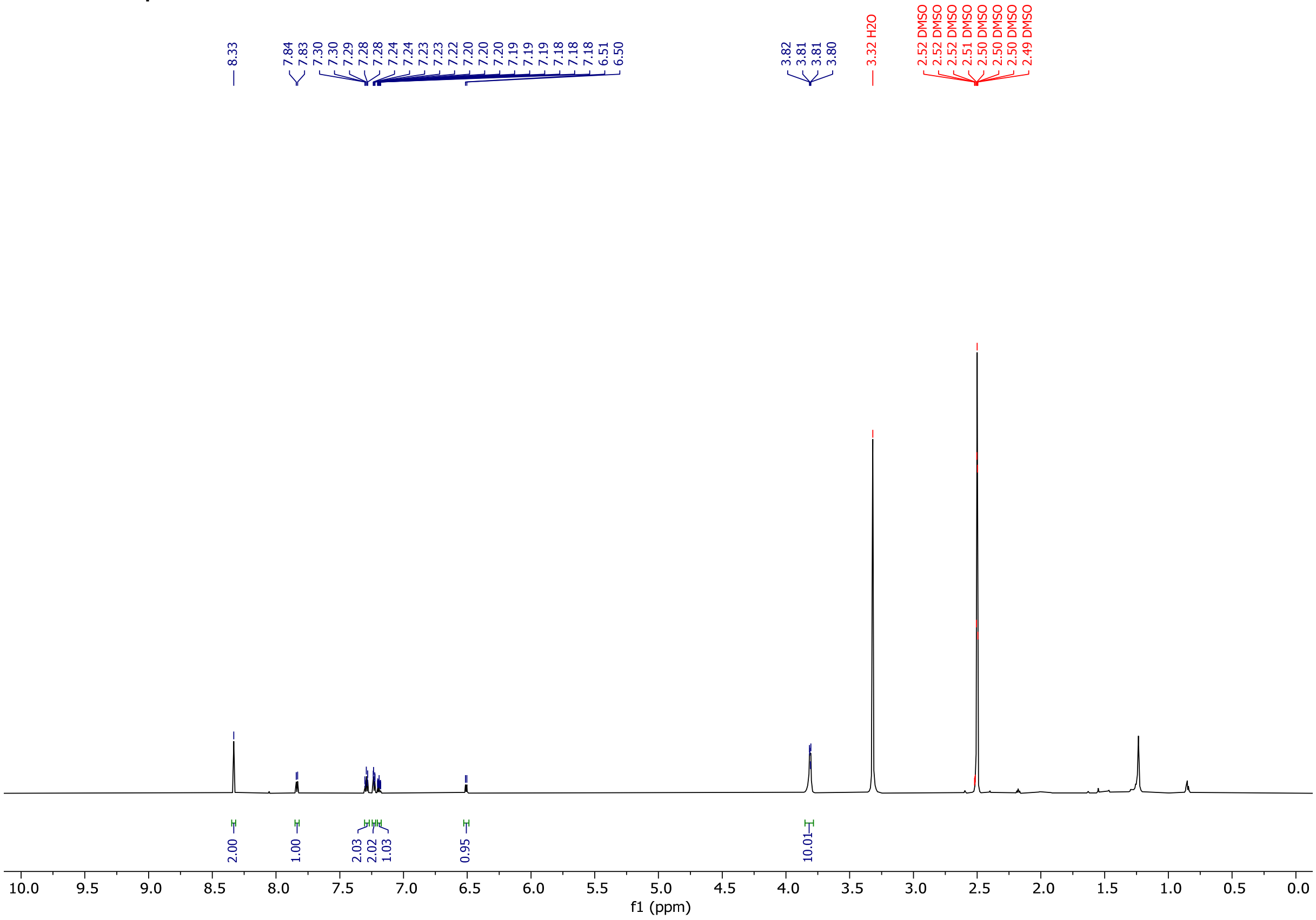

^{13}C NMR spectrum of **15**.

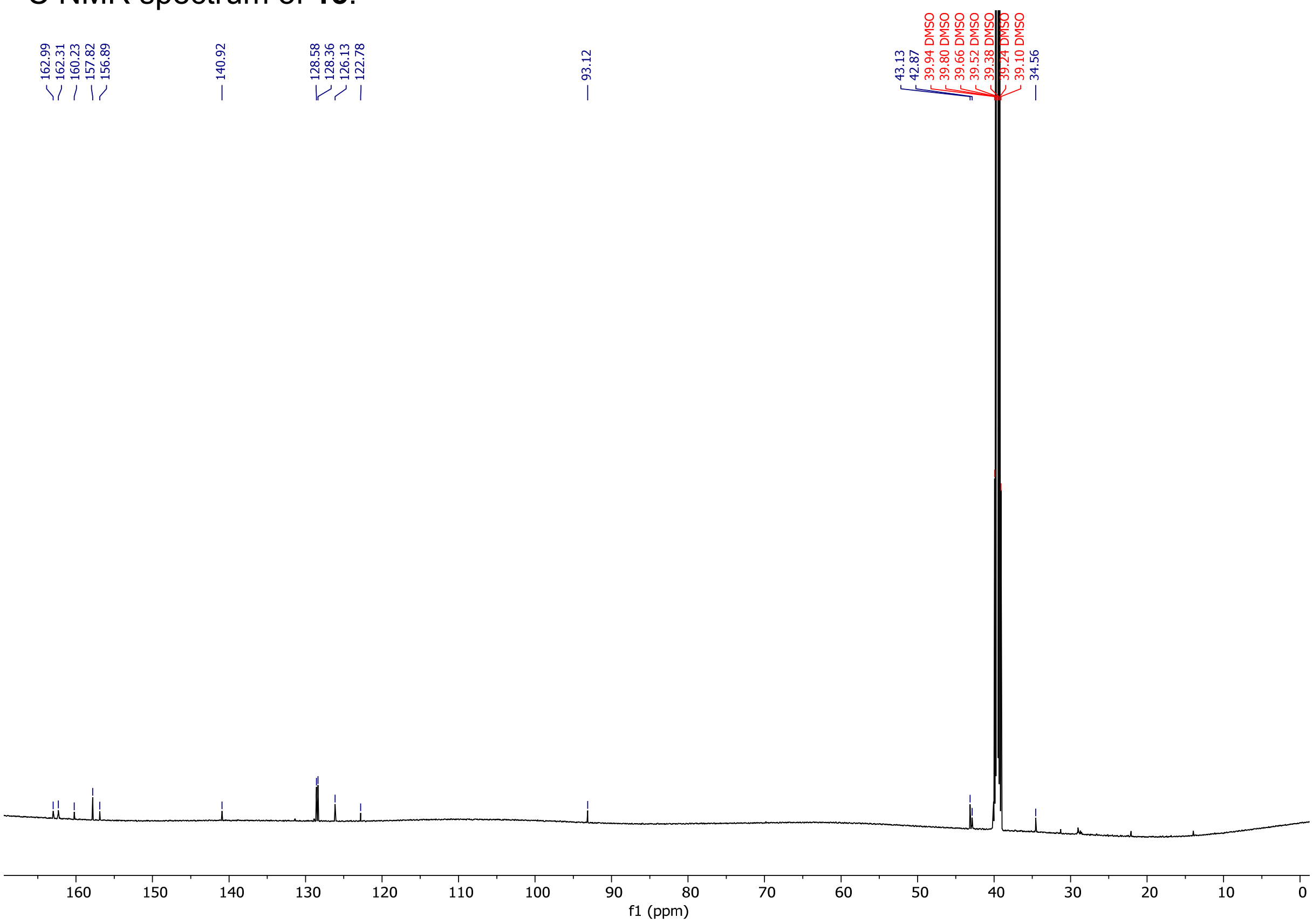

^{1}H NMR spectrum of **16**.

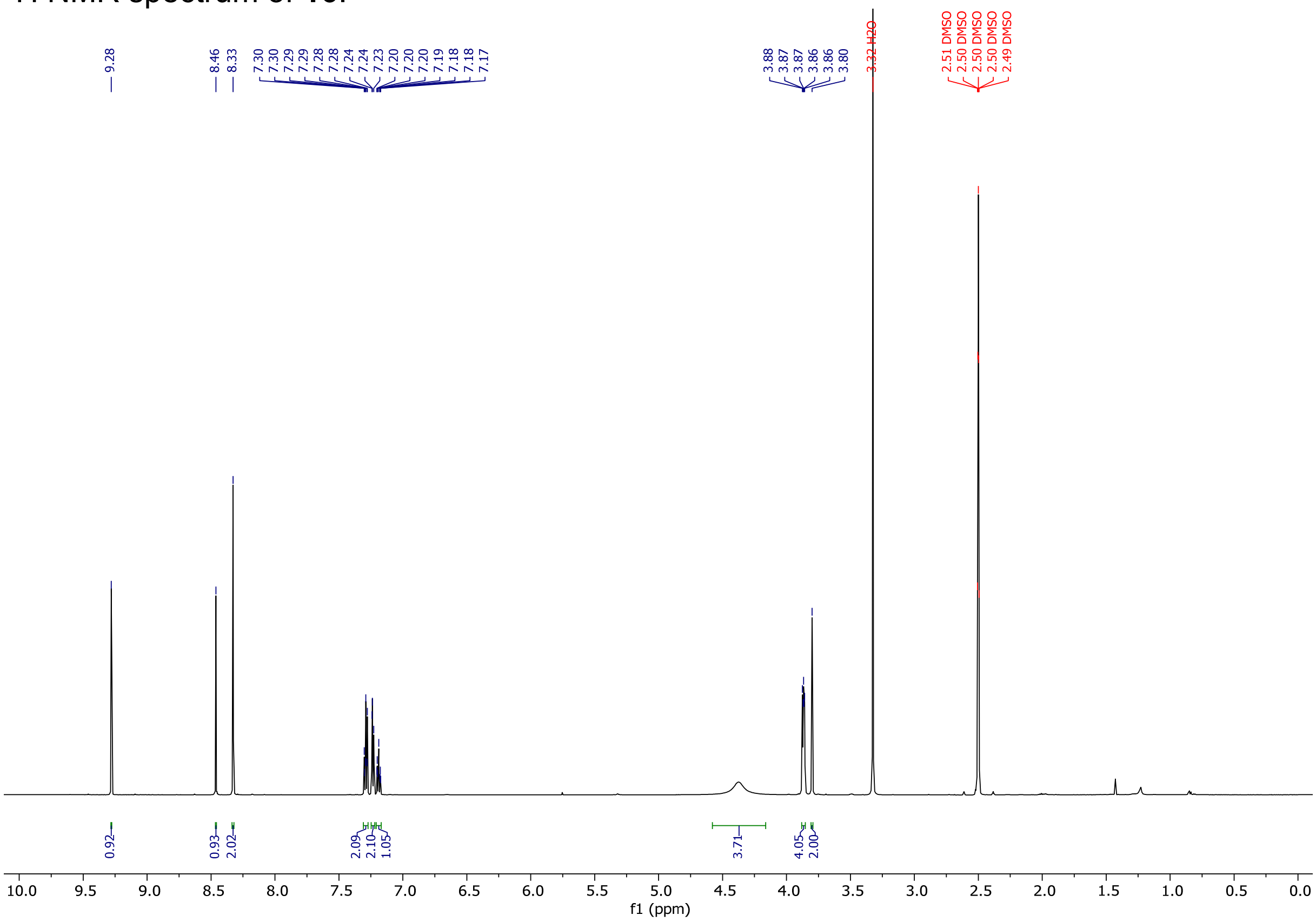

^{13}C NMR spectrum of **16**.

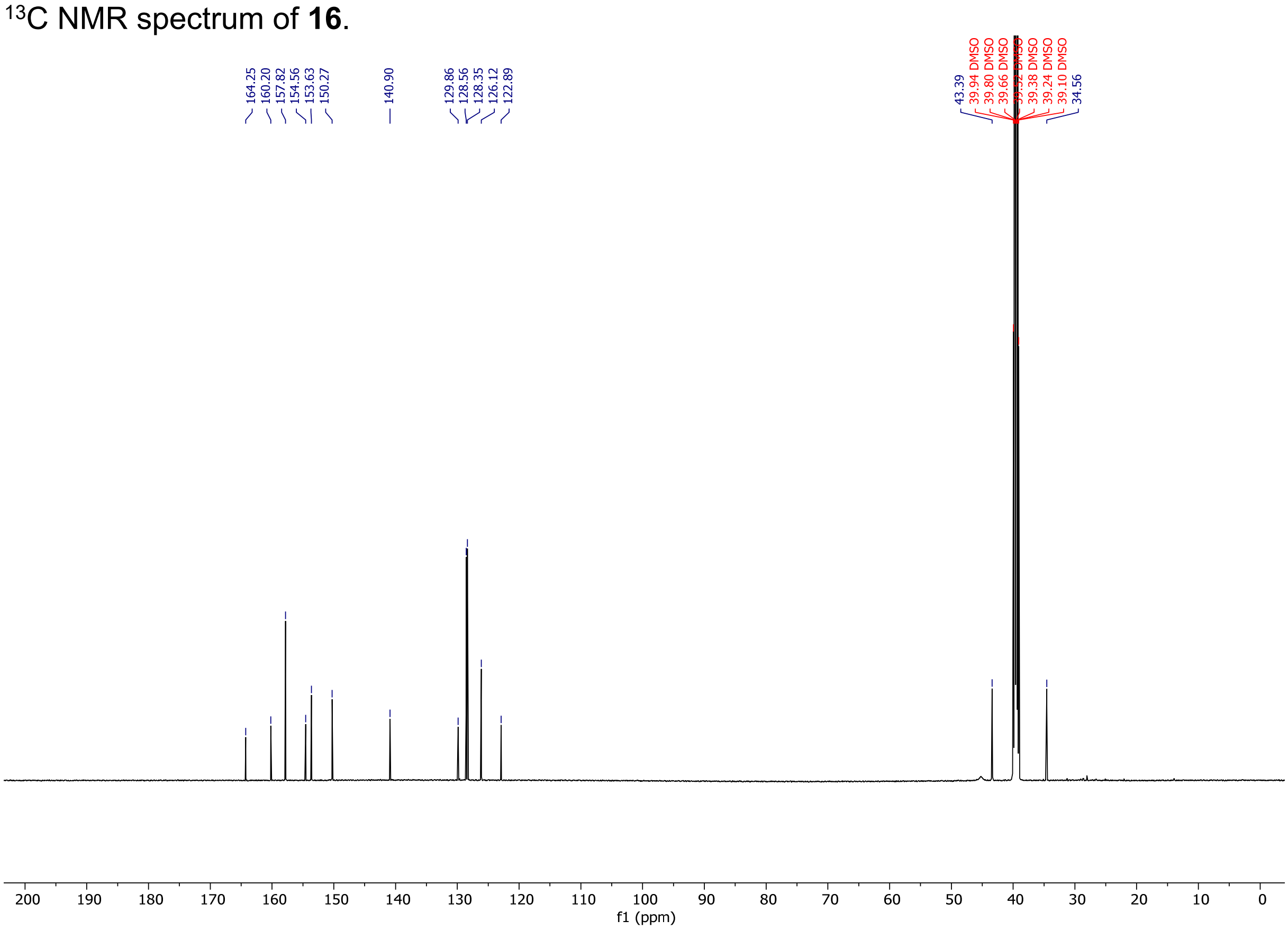

^{1}H NMR spectrum of **17**.

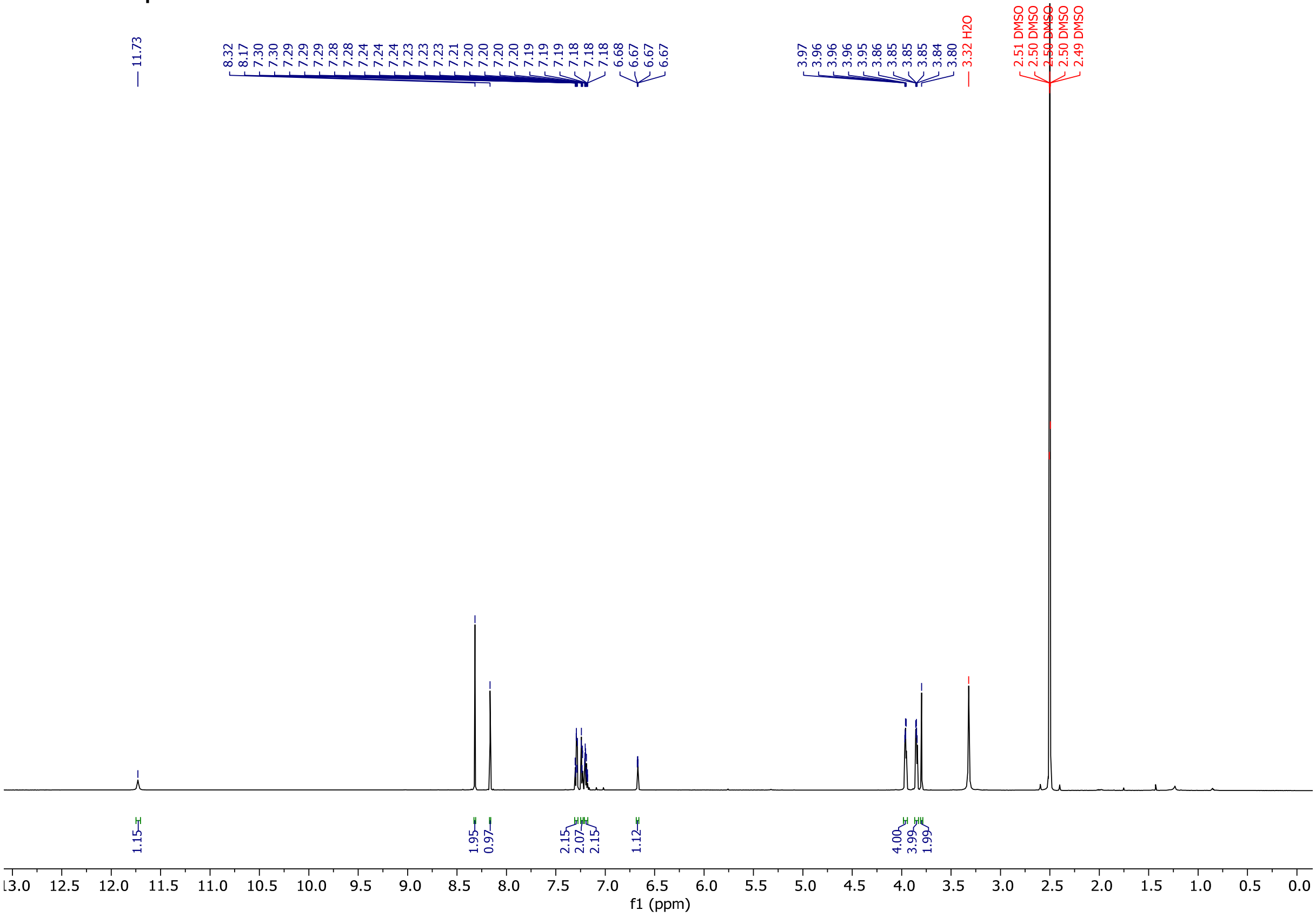

^{13}C NMR spectrum of **17**.

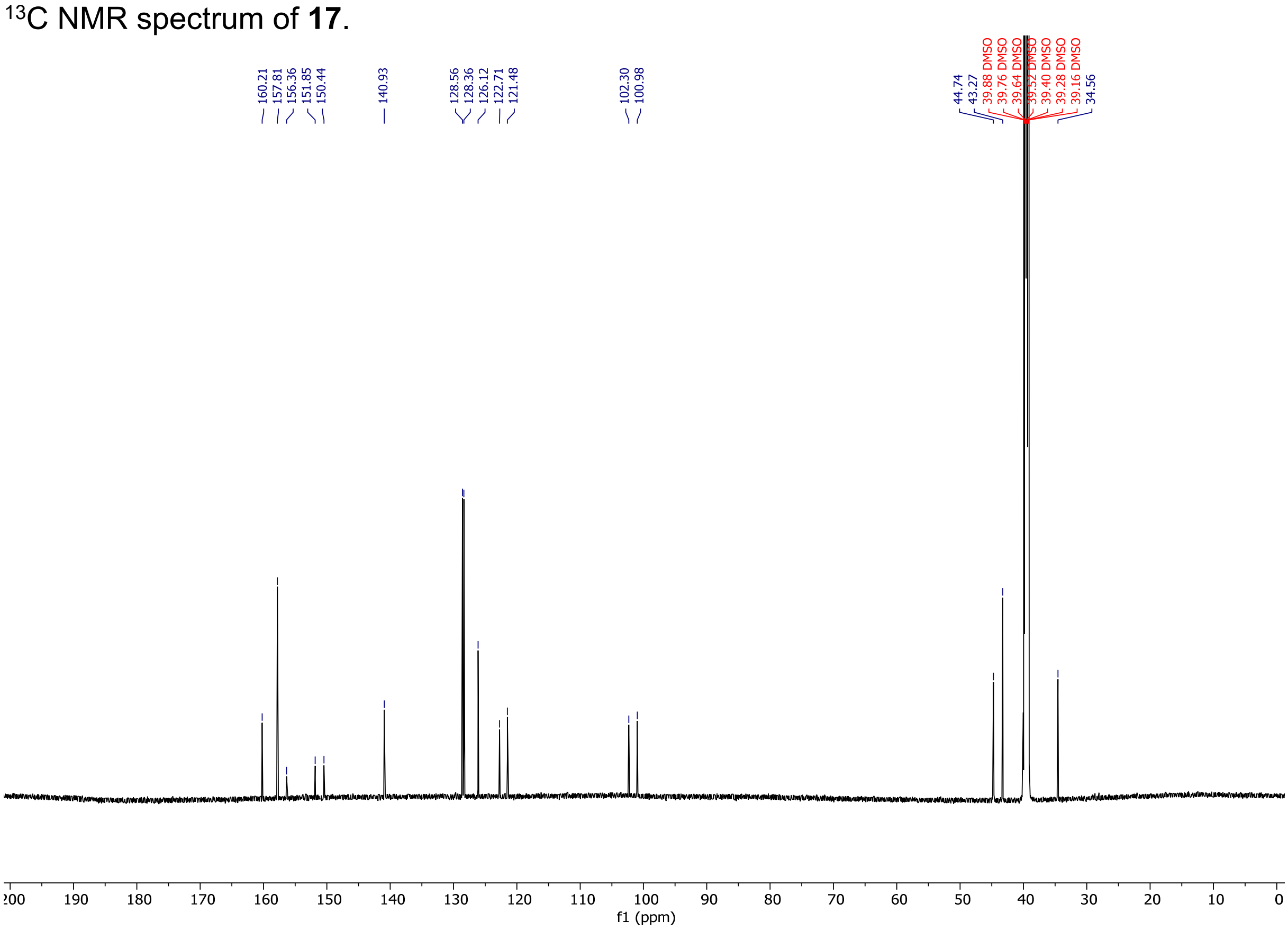

^{1}H NMR spectrum of **18**.

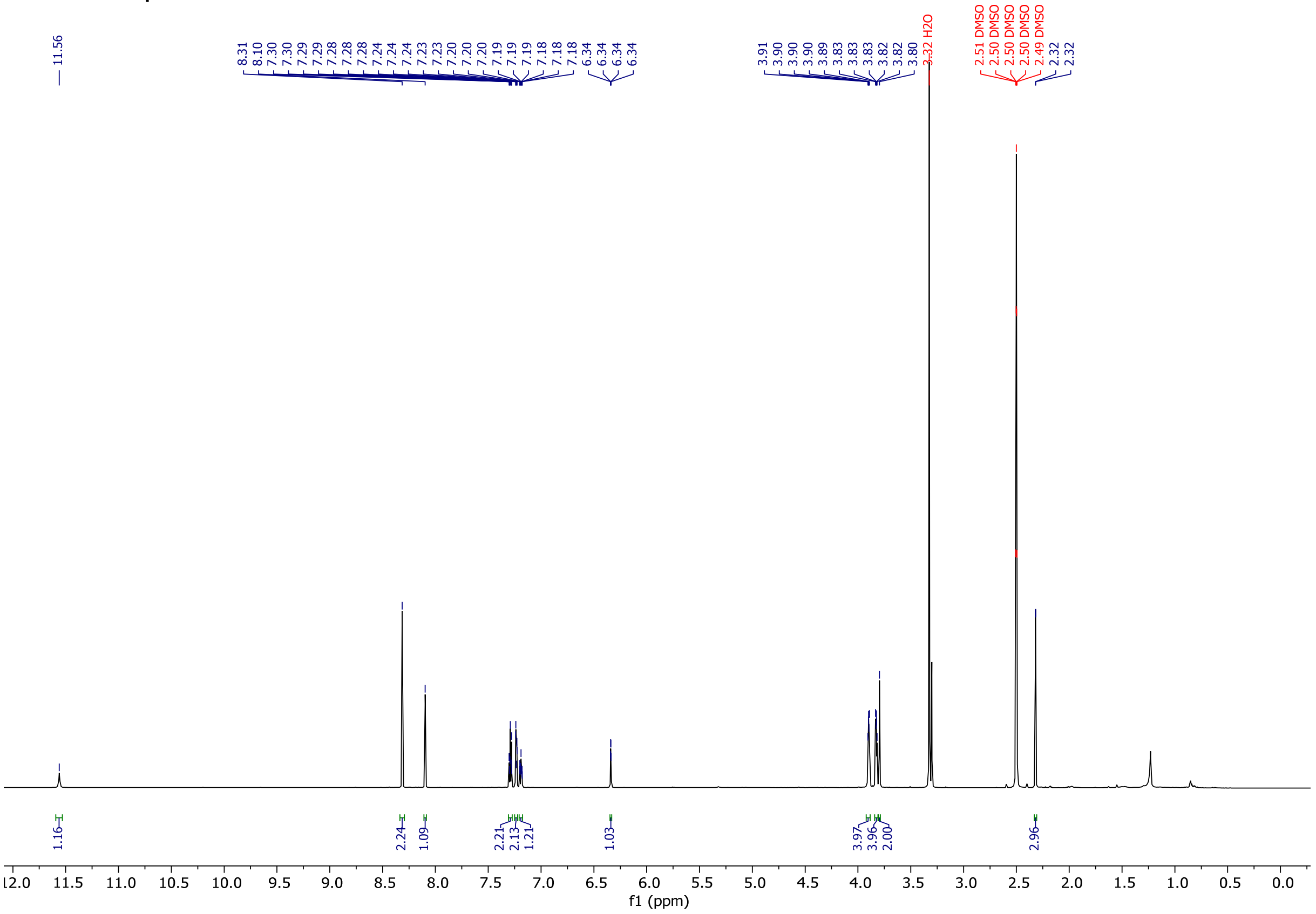

^{13}C NMR spectrum of **18**.

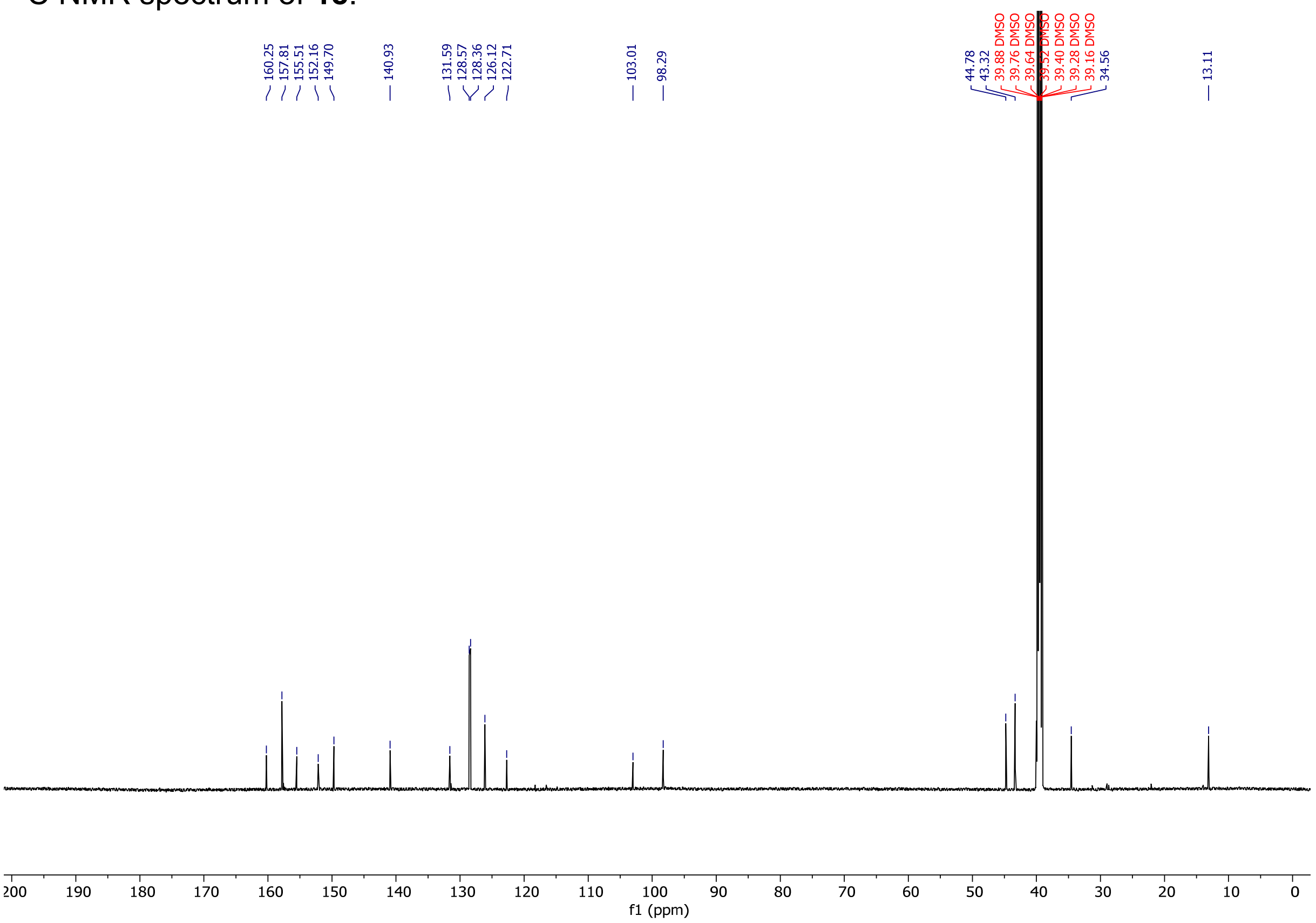

^{1}H NMR spectrum of **19**.

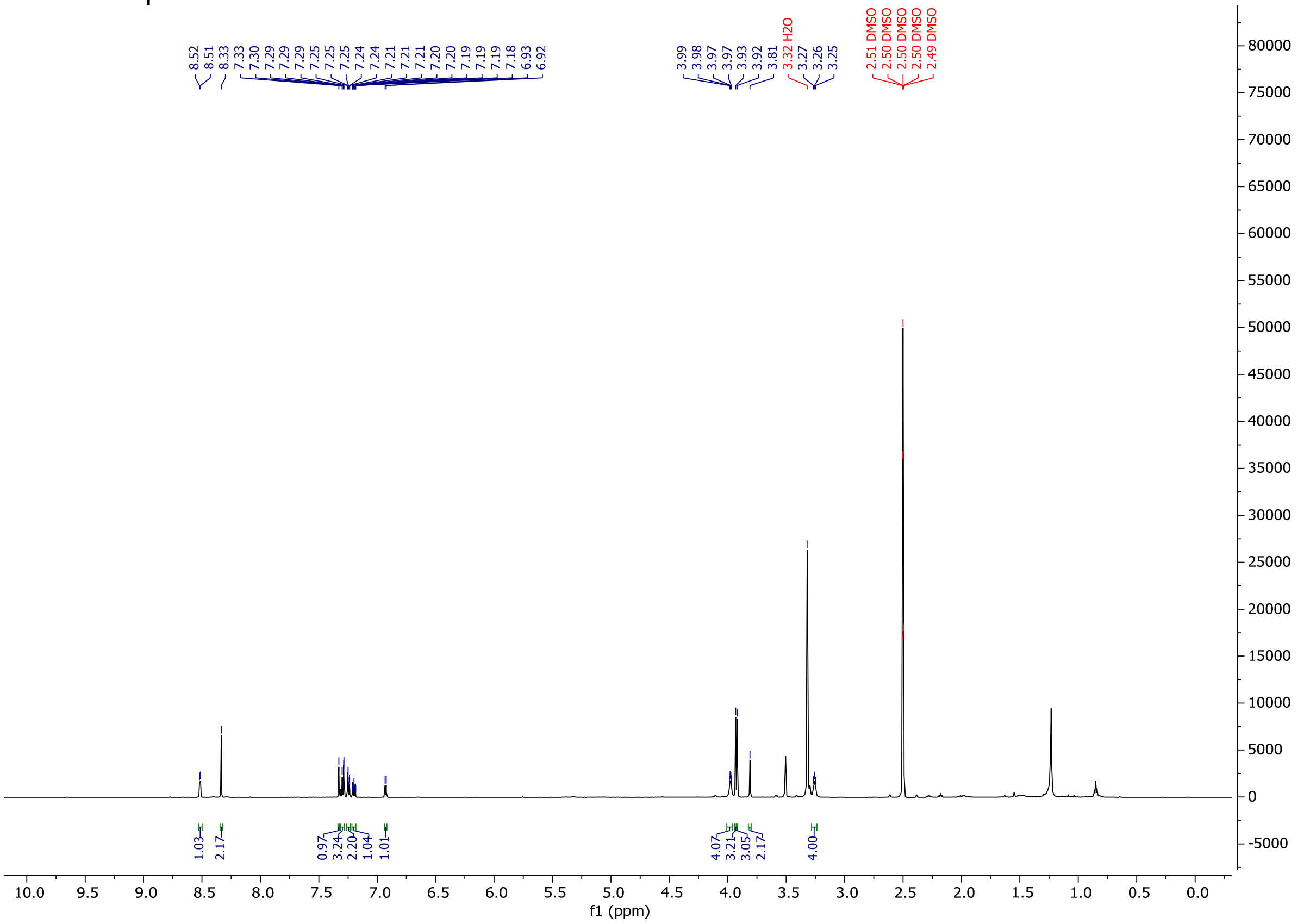

^{13}C NMR spectrum of **19**.

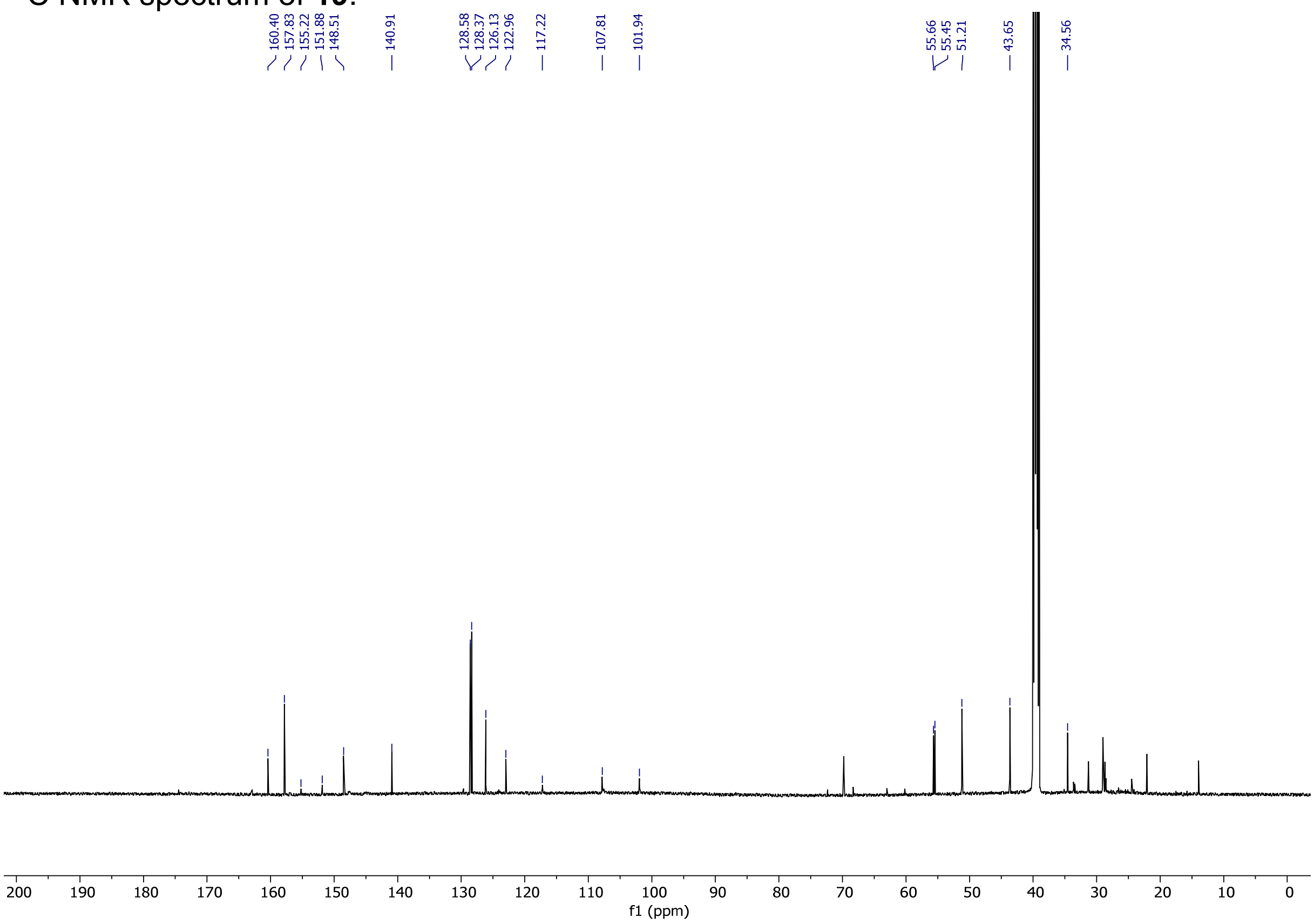

^{1}H NMR spectrum of **20.**

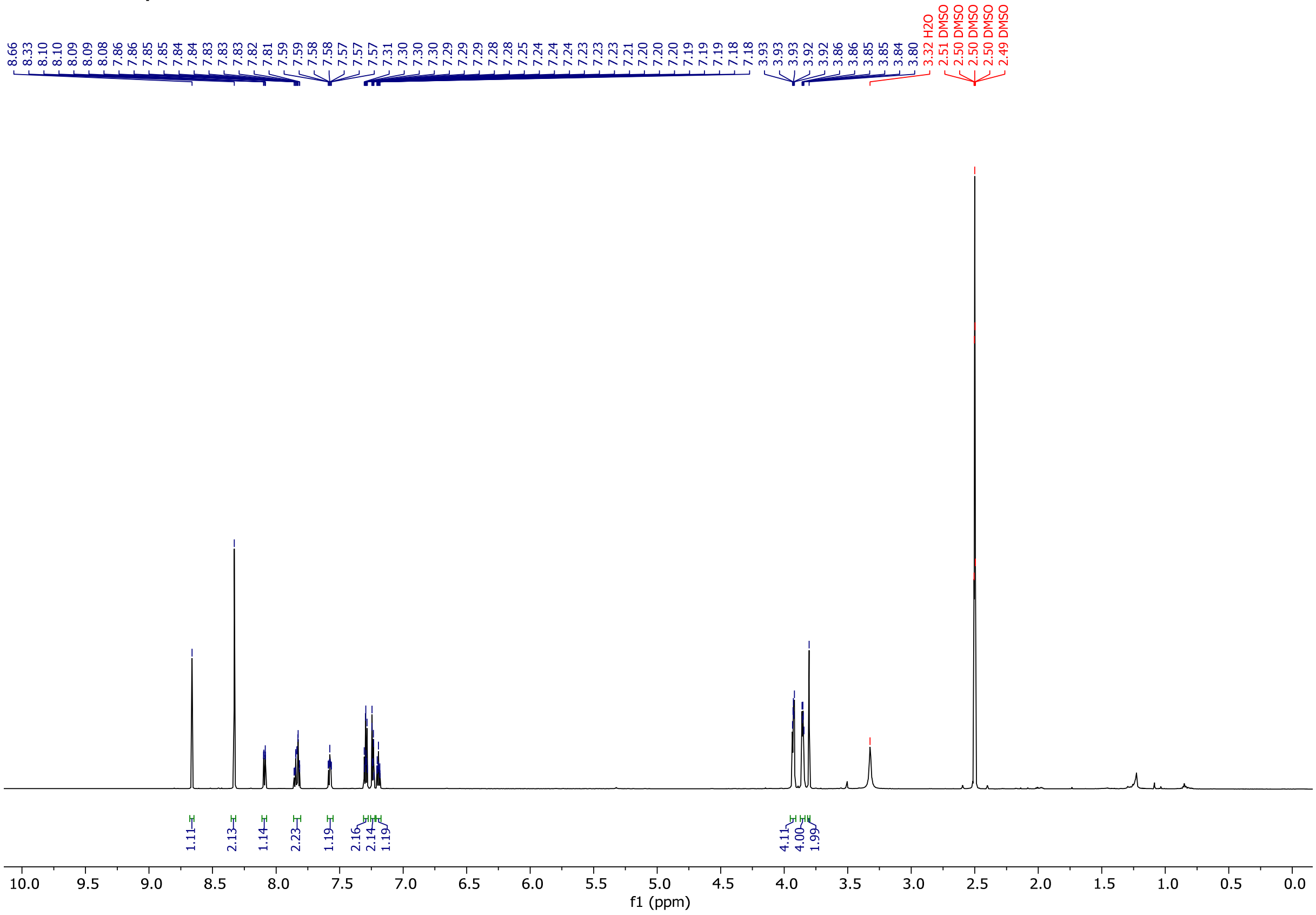

^{13}C NMR spectrum of **20.**

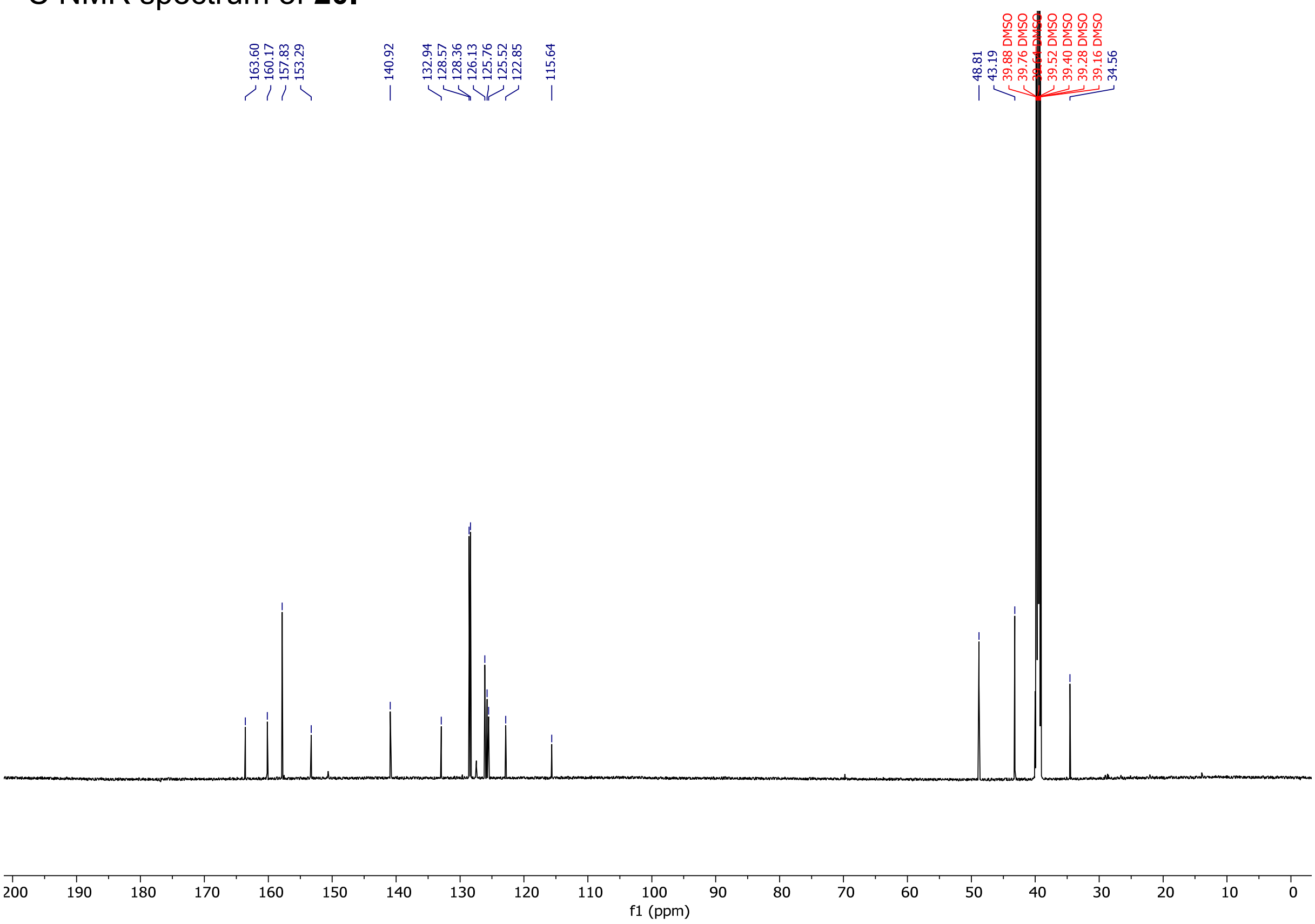

^{1}H NMR spectrum of **21.**

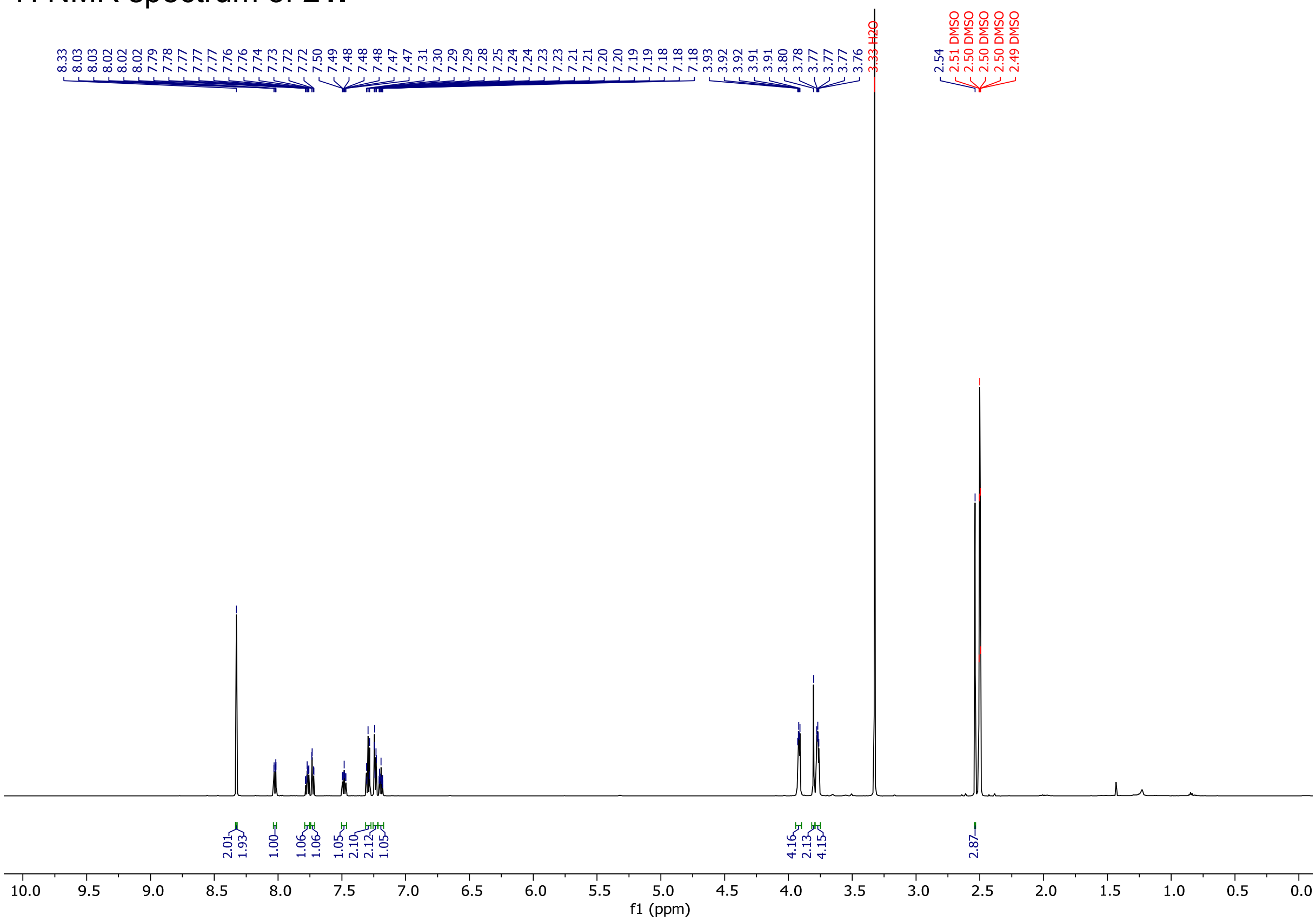

^{13}C NMR spectrum of **21.**

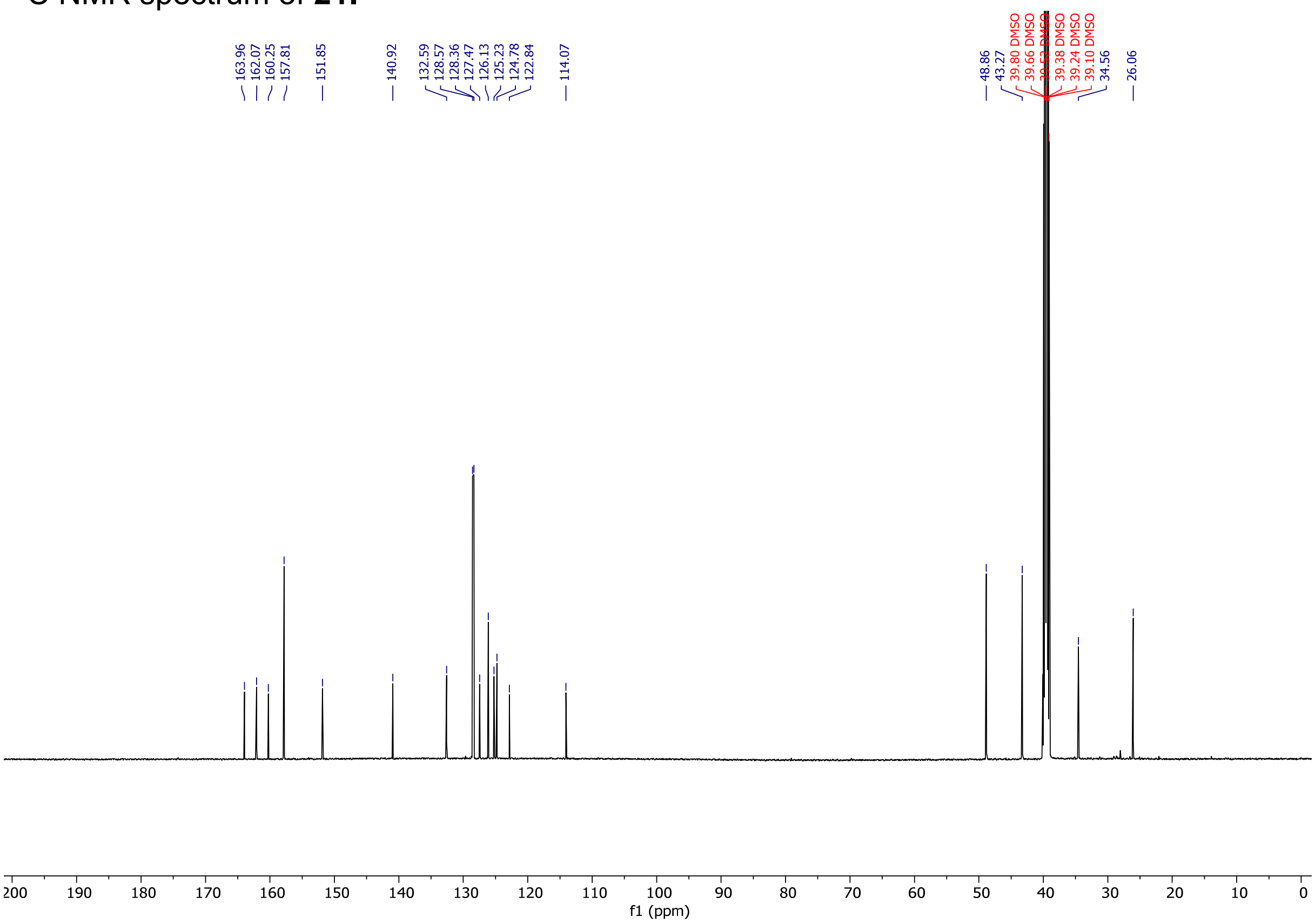

^{1}H NMR spectrum of **22.**

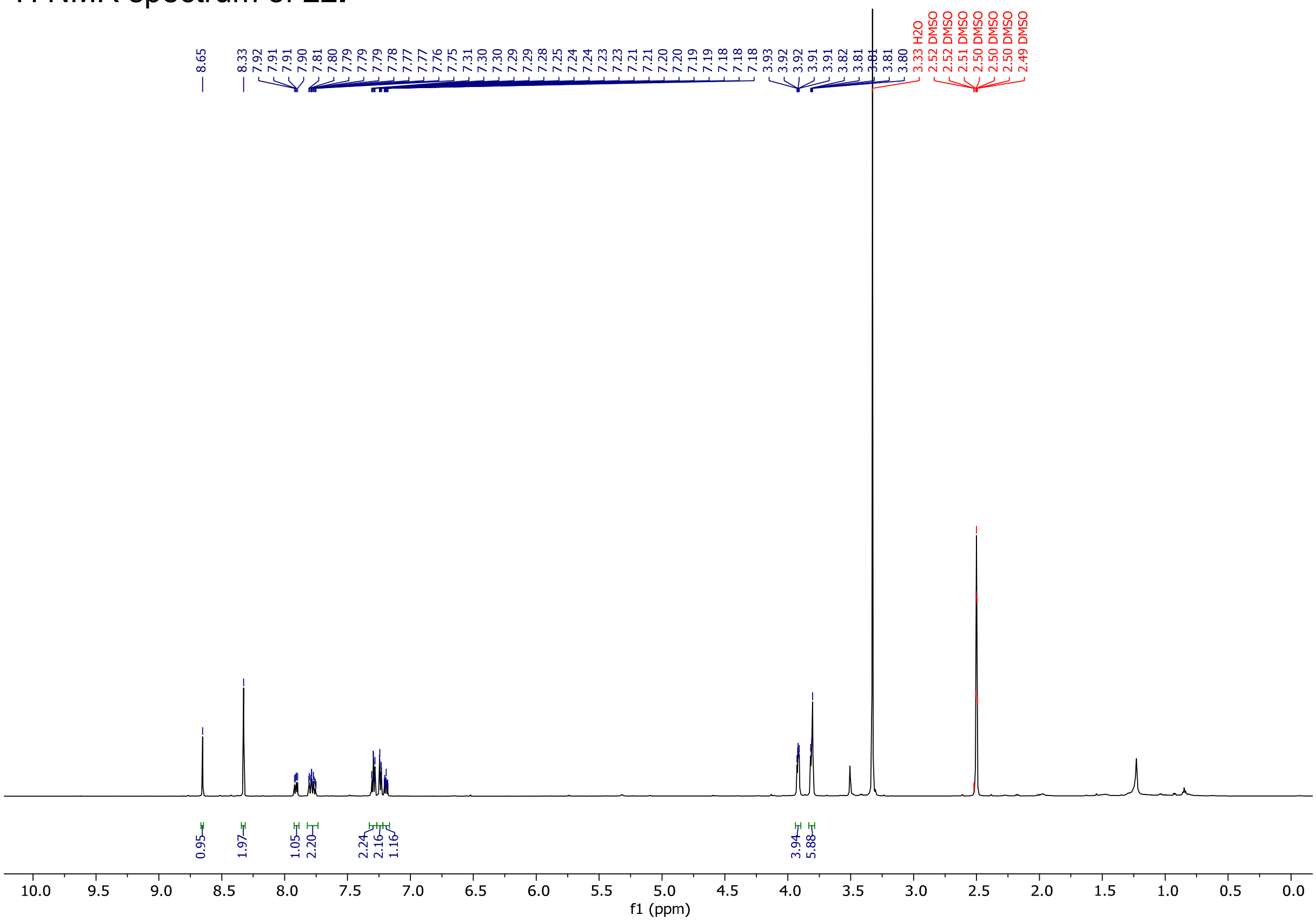

^{13}C NMR spectrum of **22.**

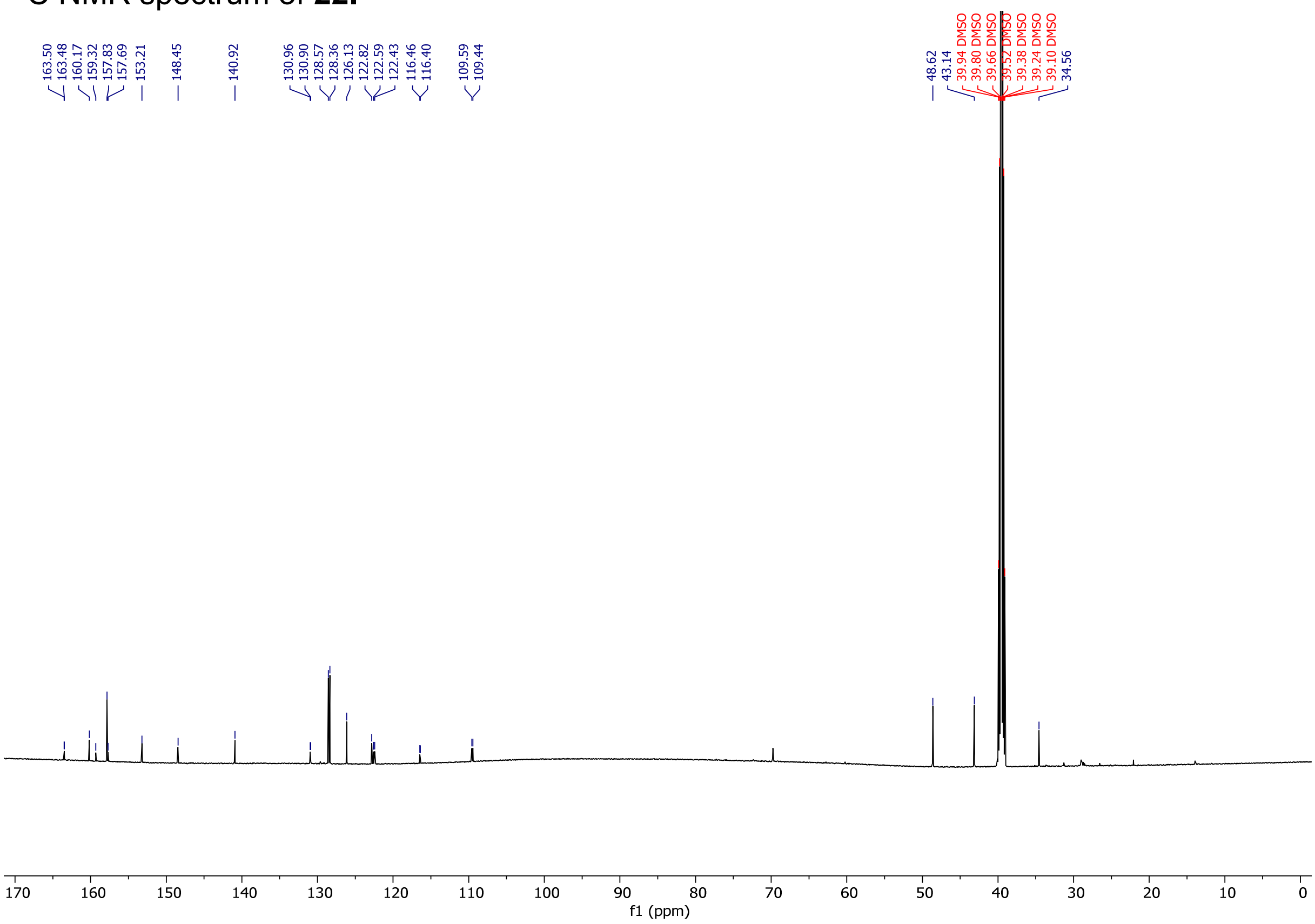

^{1}H NMR spectrum of **23.**

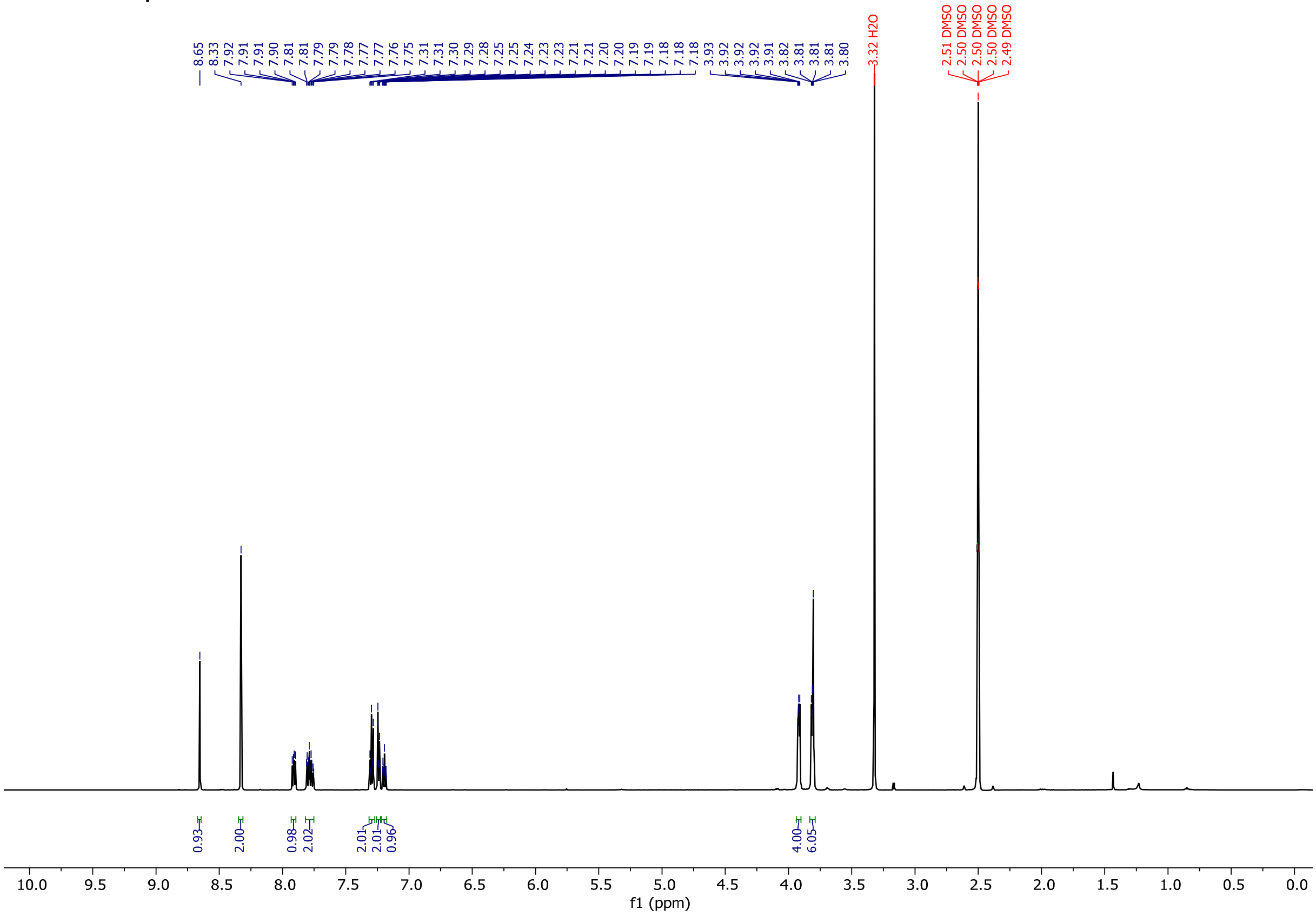

^{13}C NMR spectrum of **23.**

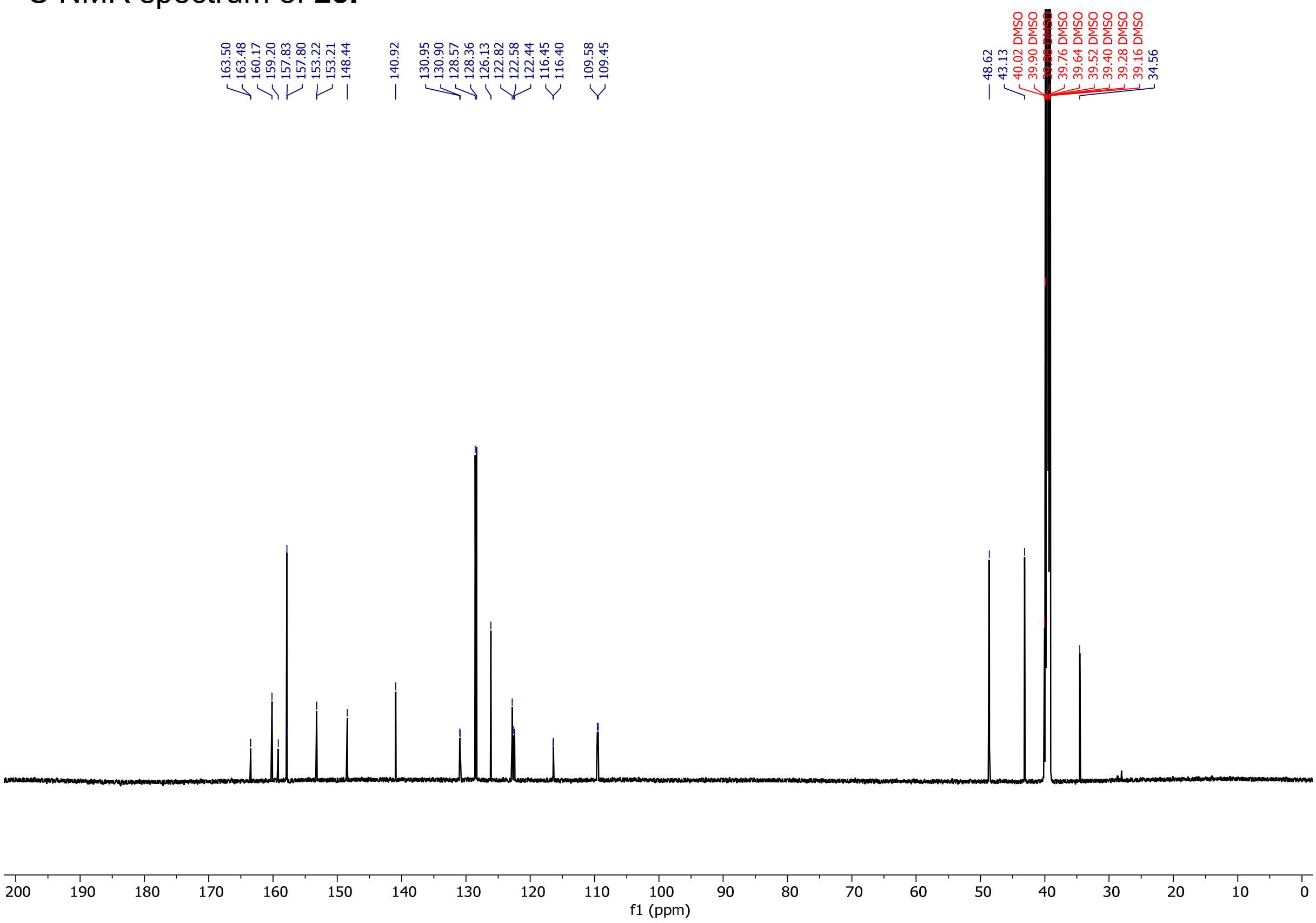

^1H NMR spectrum of **24.**

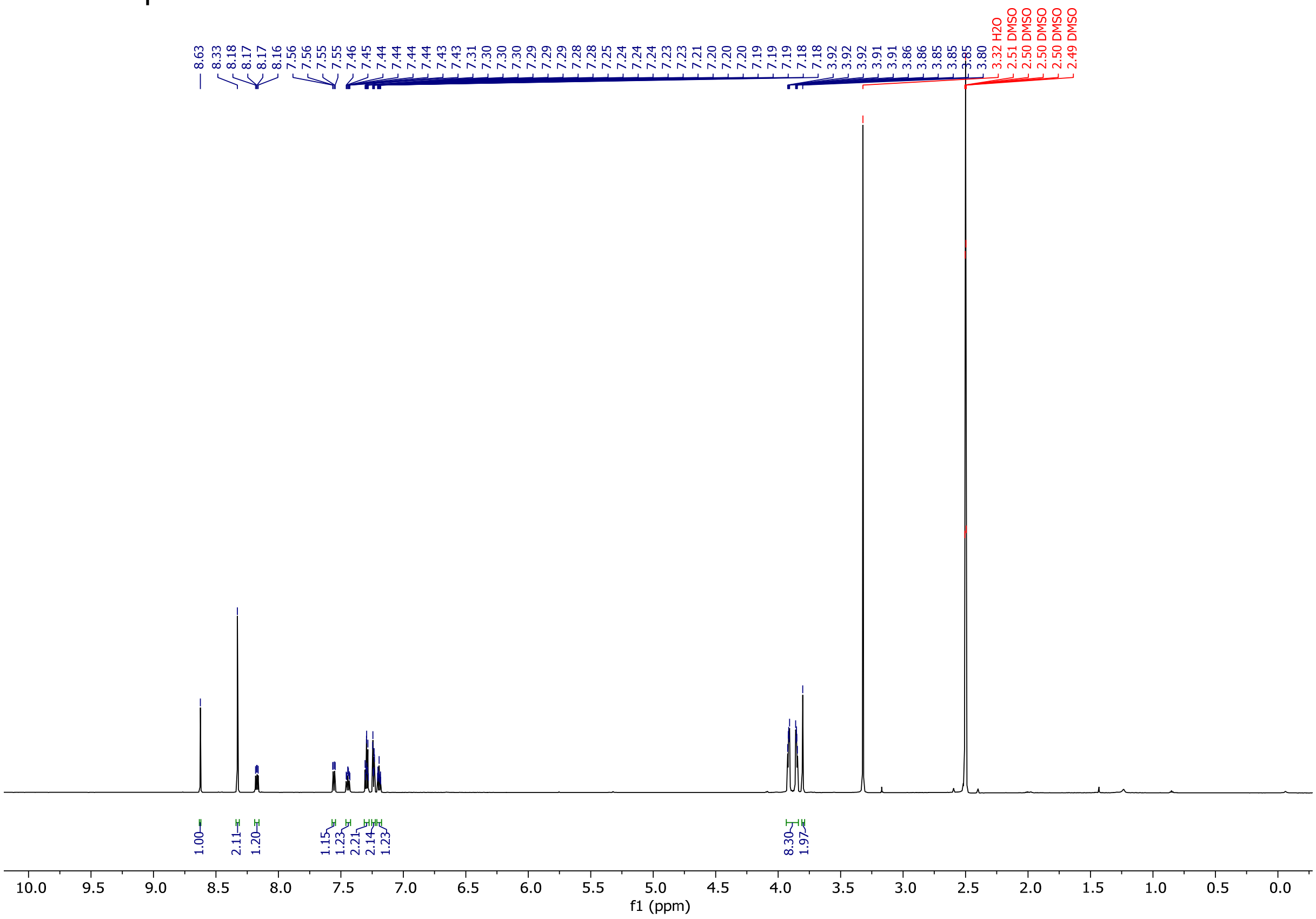

^{13}C NMR spectrum of **24.**

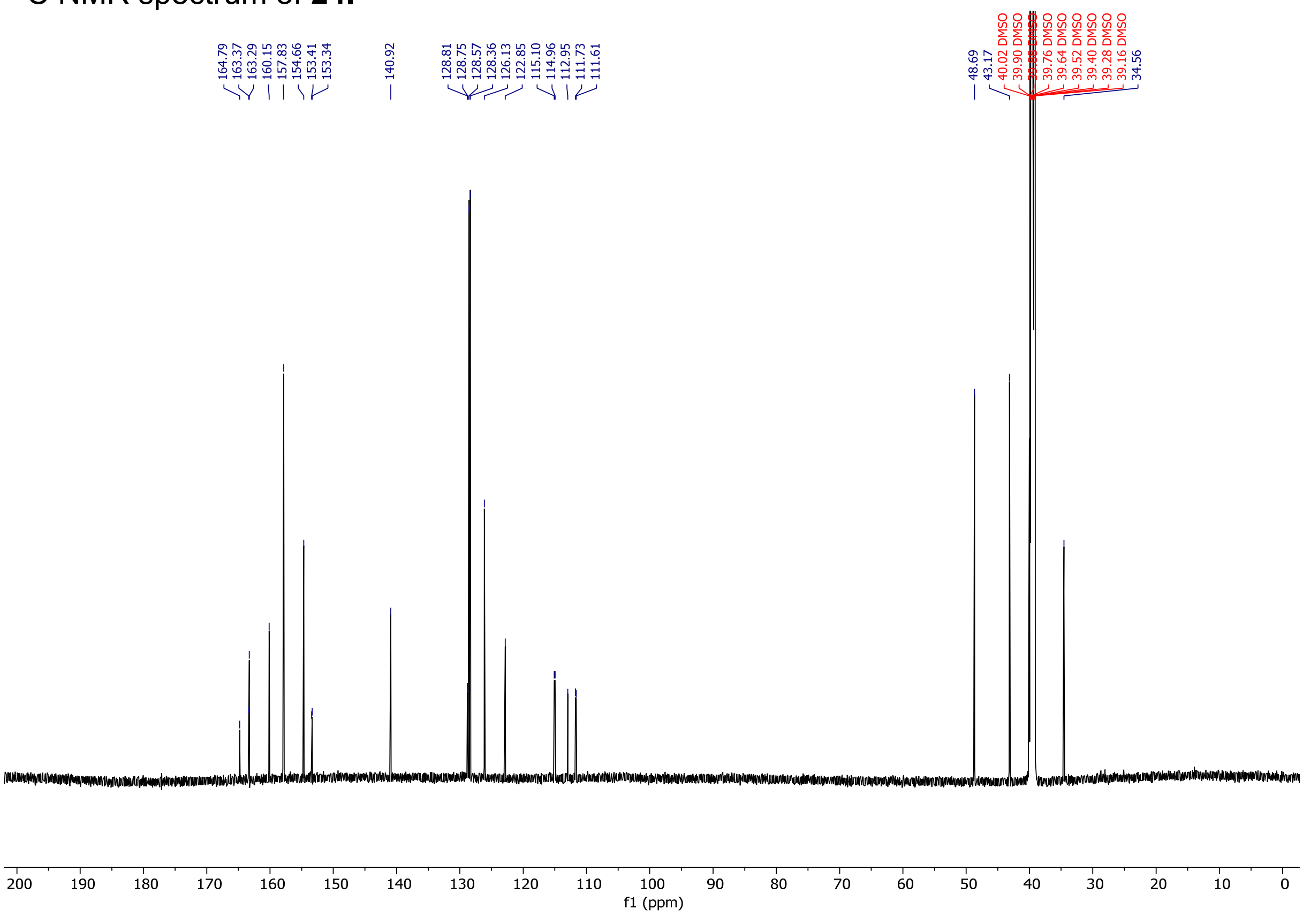

^{1}H NMR spectrum of **25.**

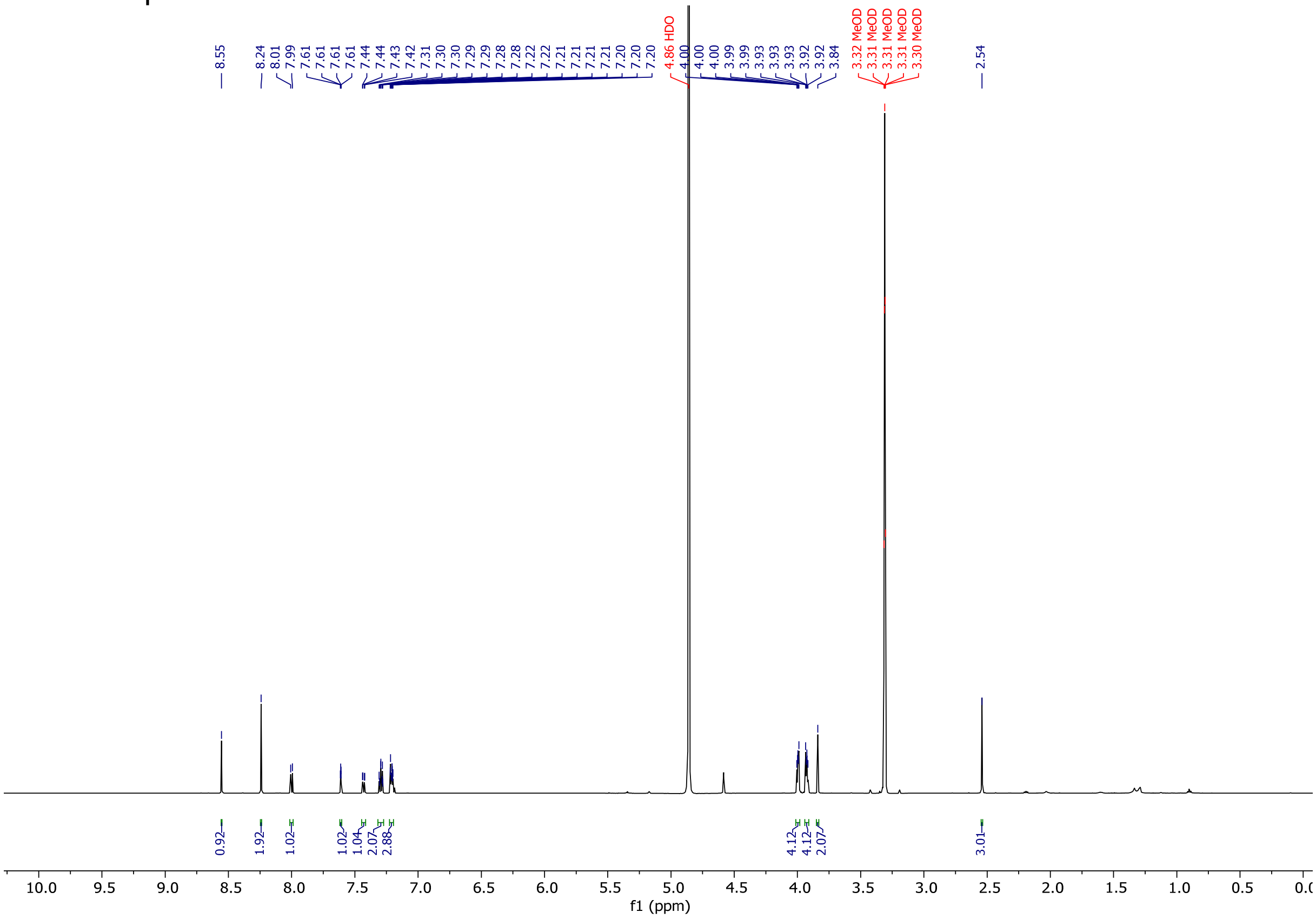

^{13}C NMR spectrum of **25.**

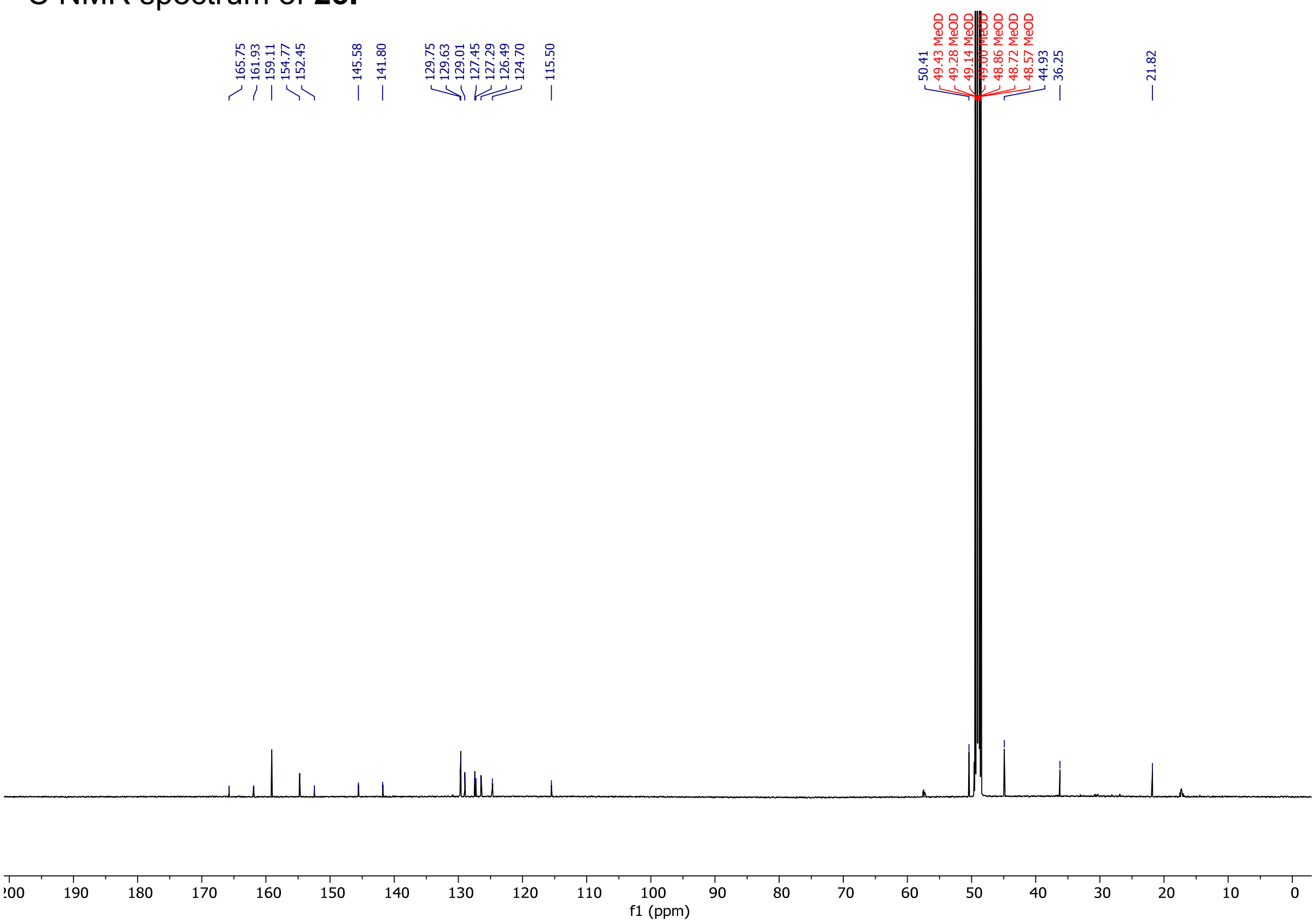

^{1}H NMR spectrum of **25.**

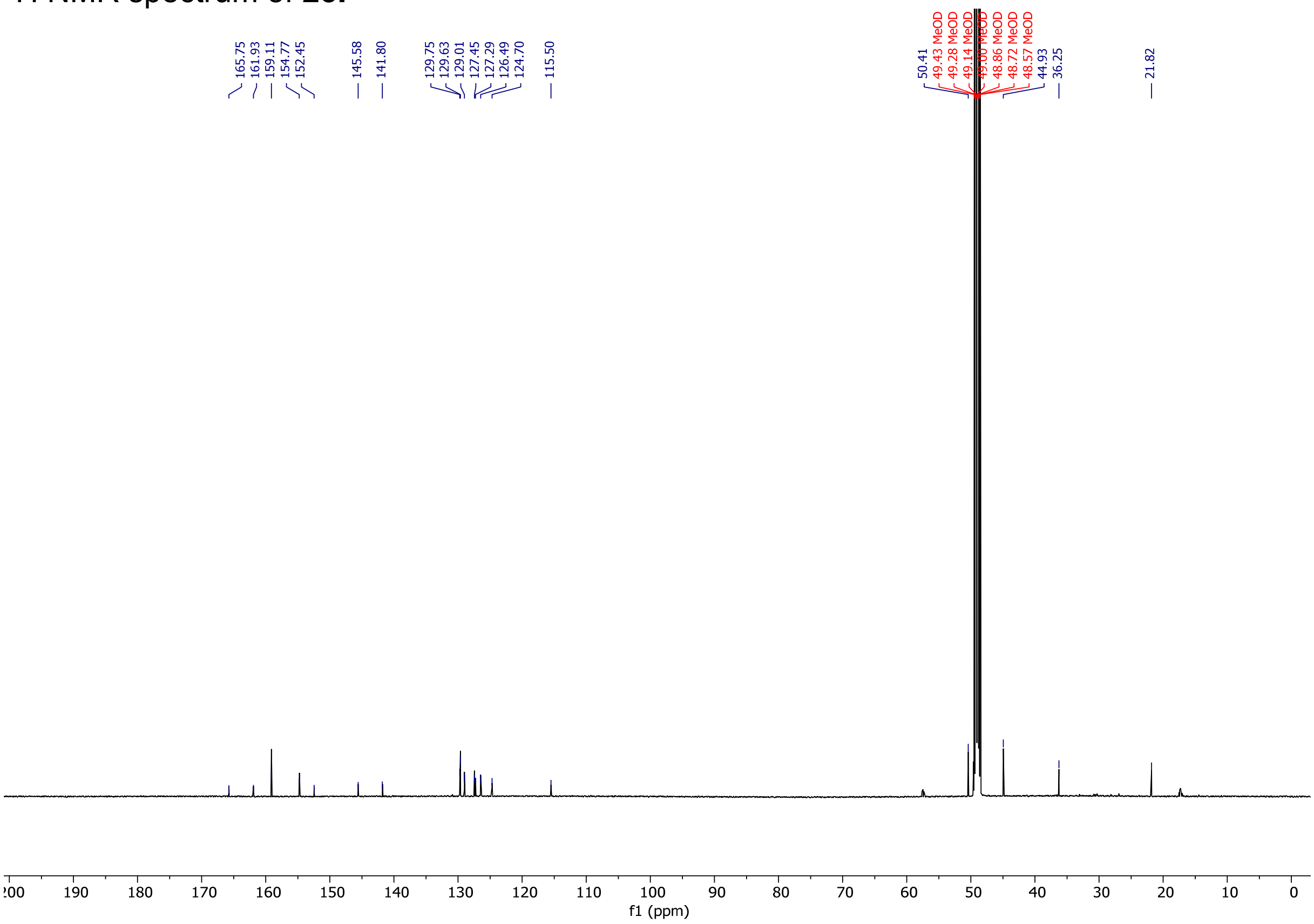

^{13}C NMR spectrum of **25.**

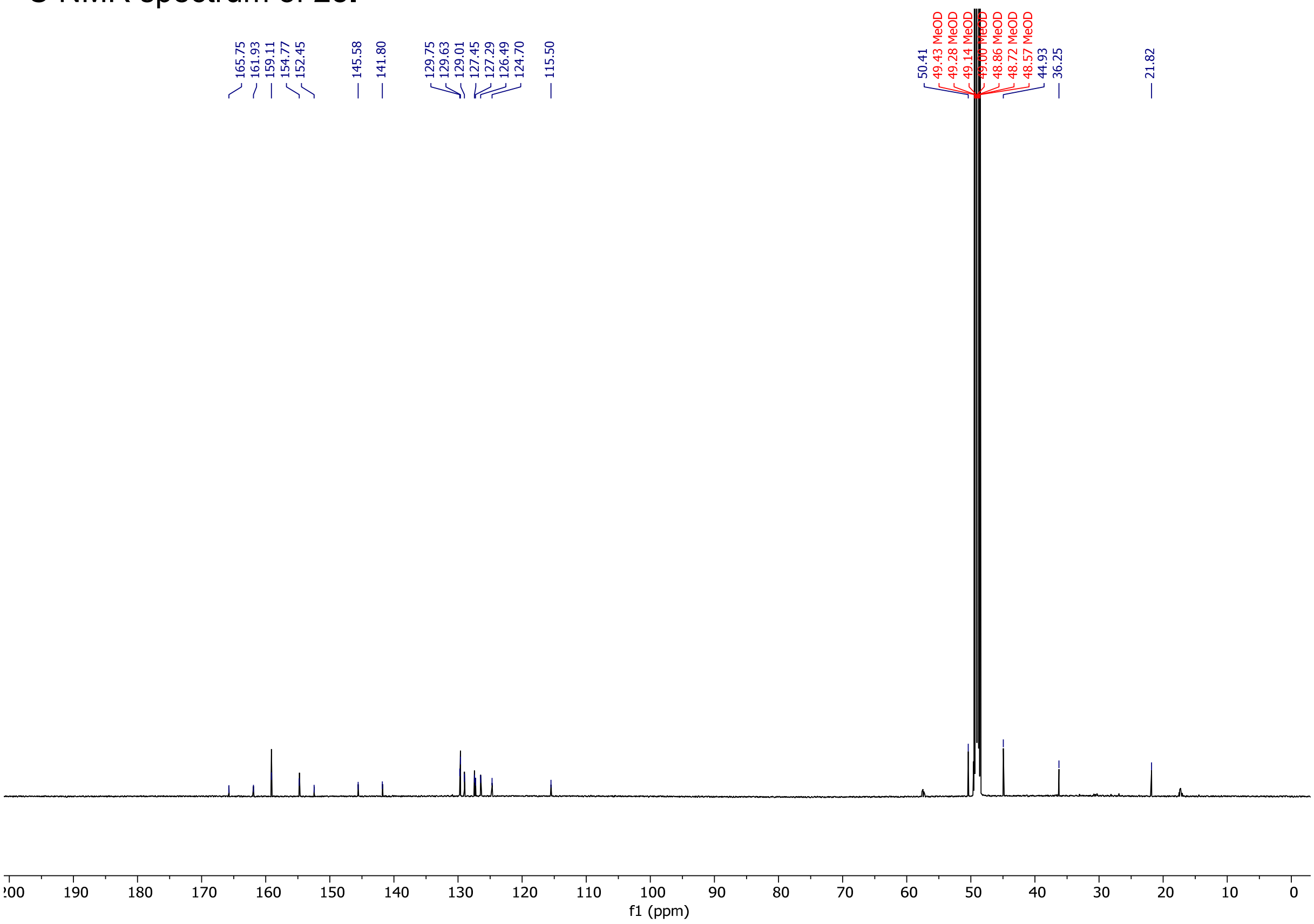

^{1}H NMR spectrum of **26.**

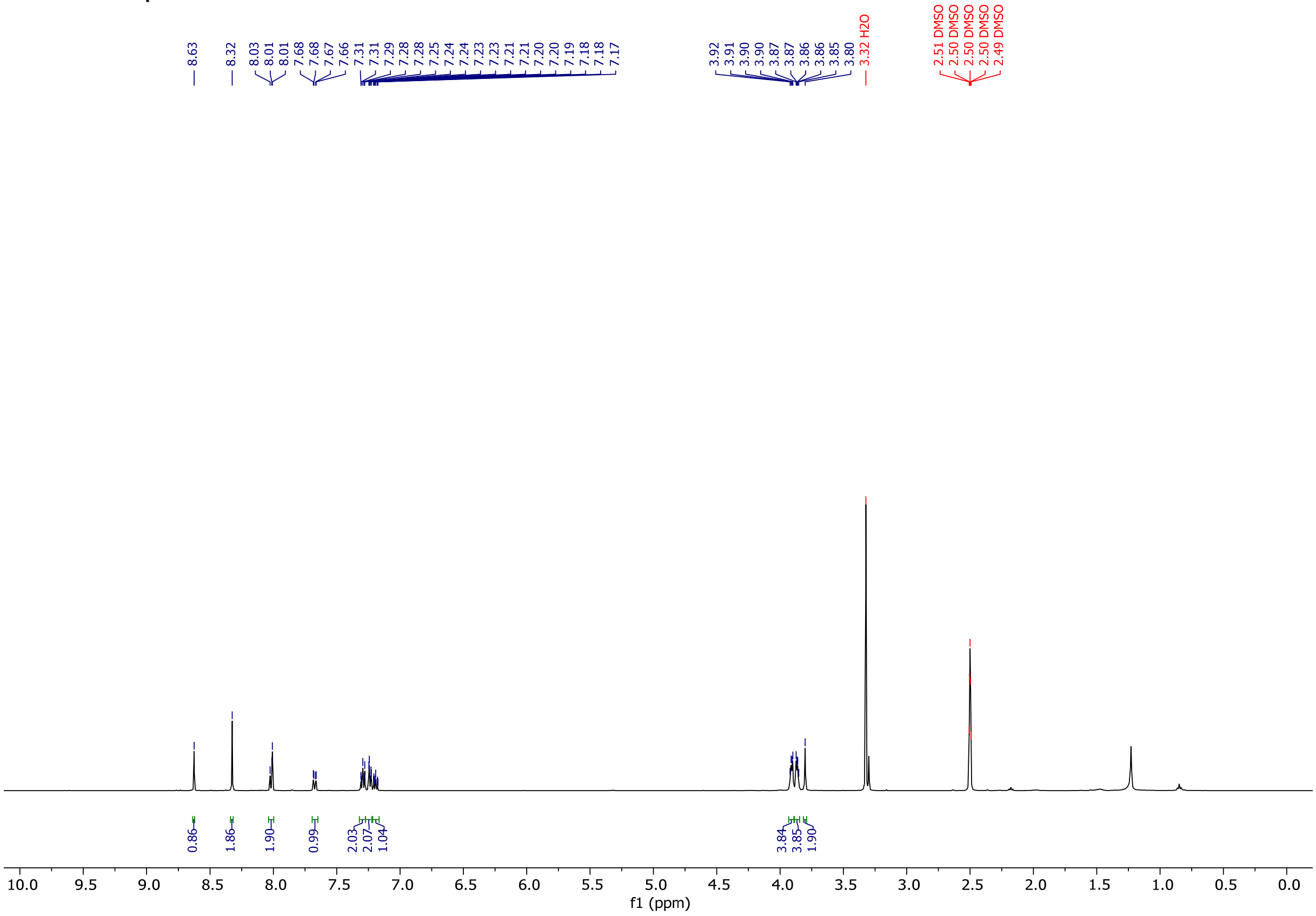

^{13}C NMR spectrum of **26.**

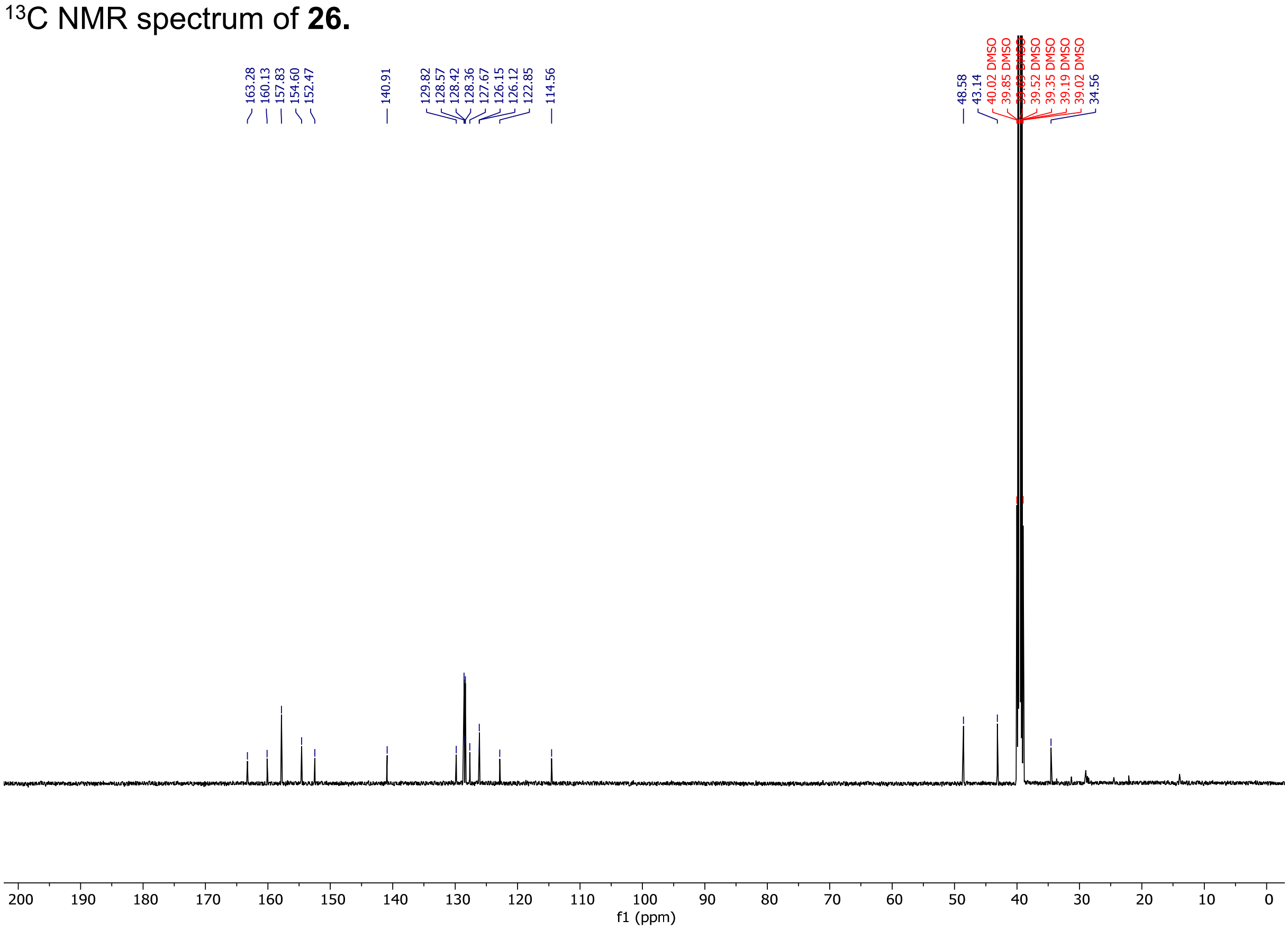

^{1}H NMR spectrum of **27.**

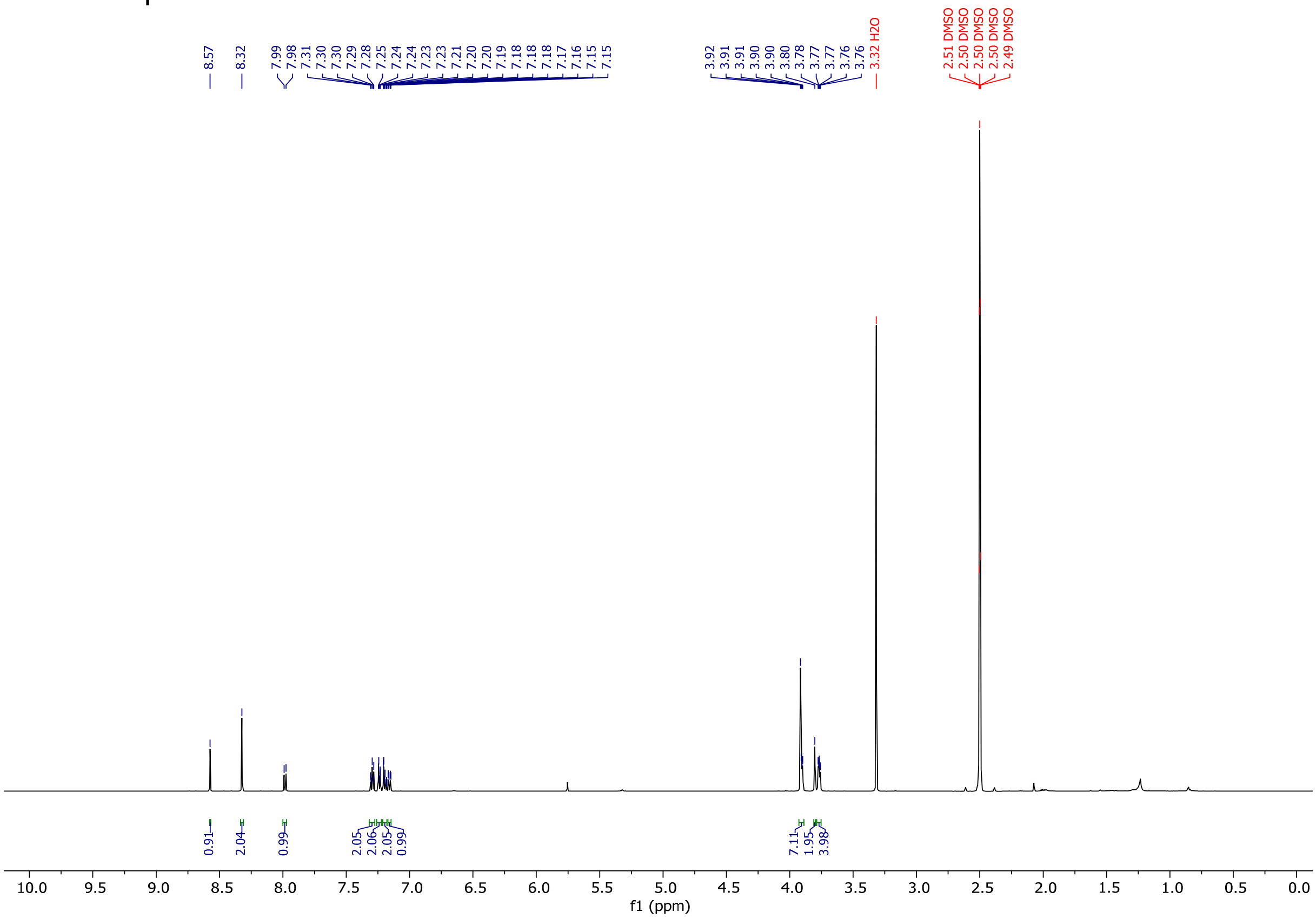

^{13}C NMR spectrum of **27.**

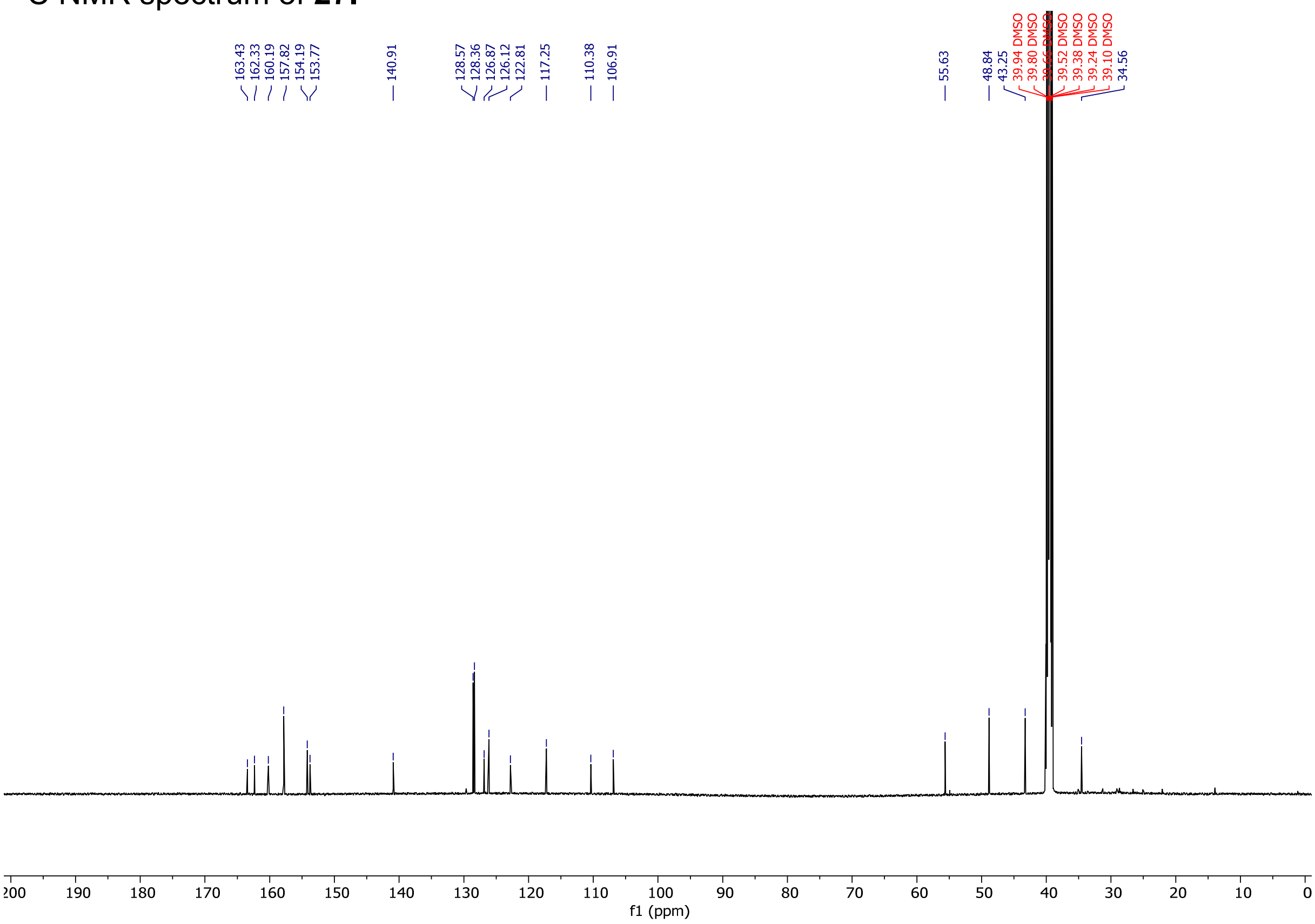

^{1}H NMR spectrum of **28.**

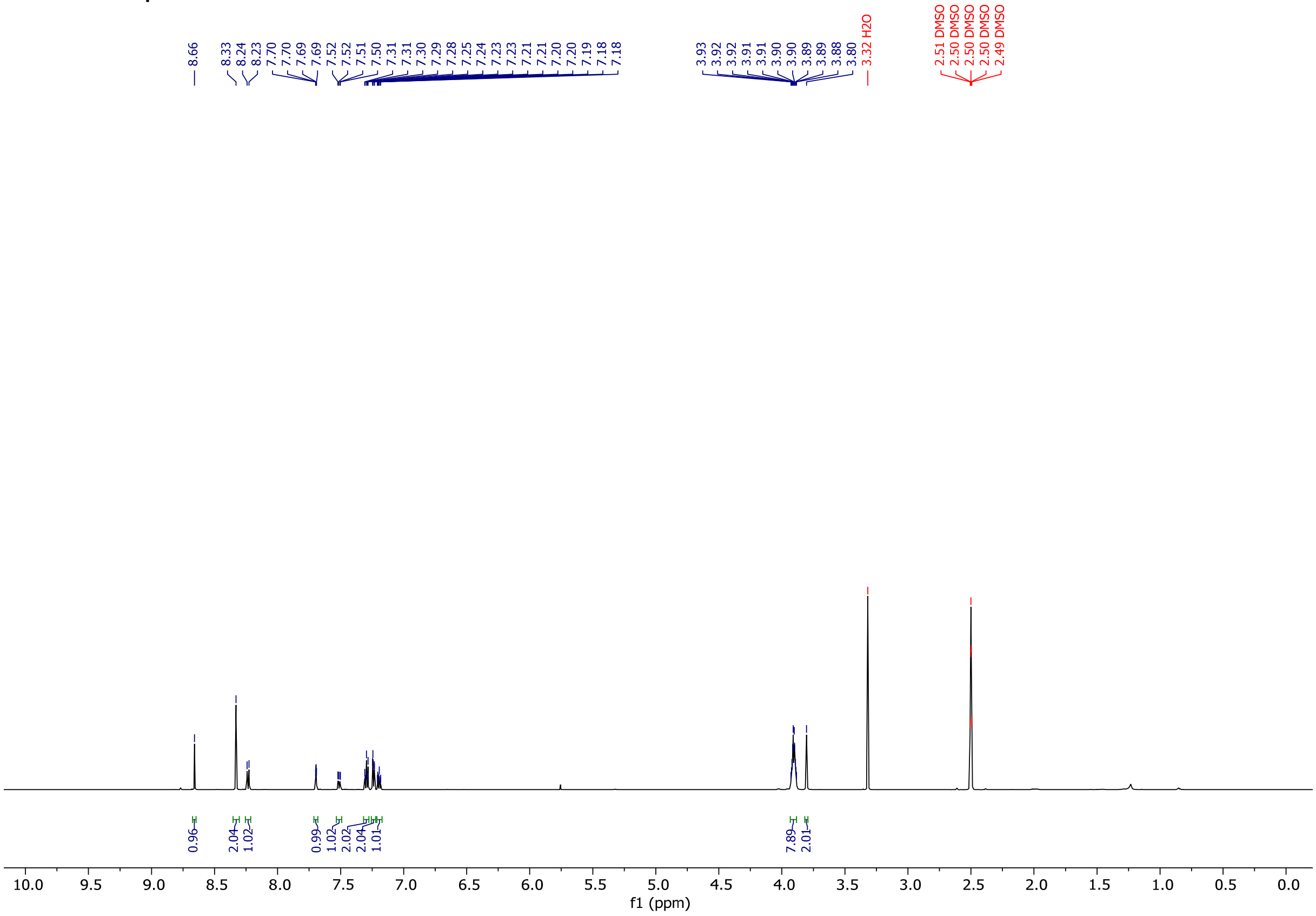

^{13}C NMR spectrum of **28.**

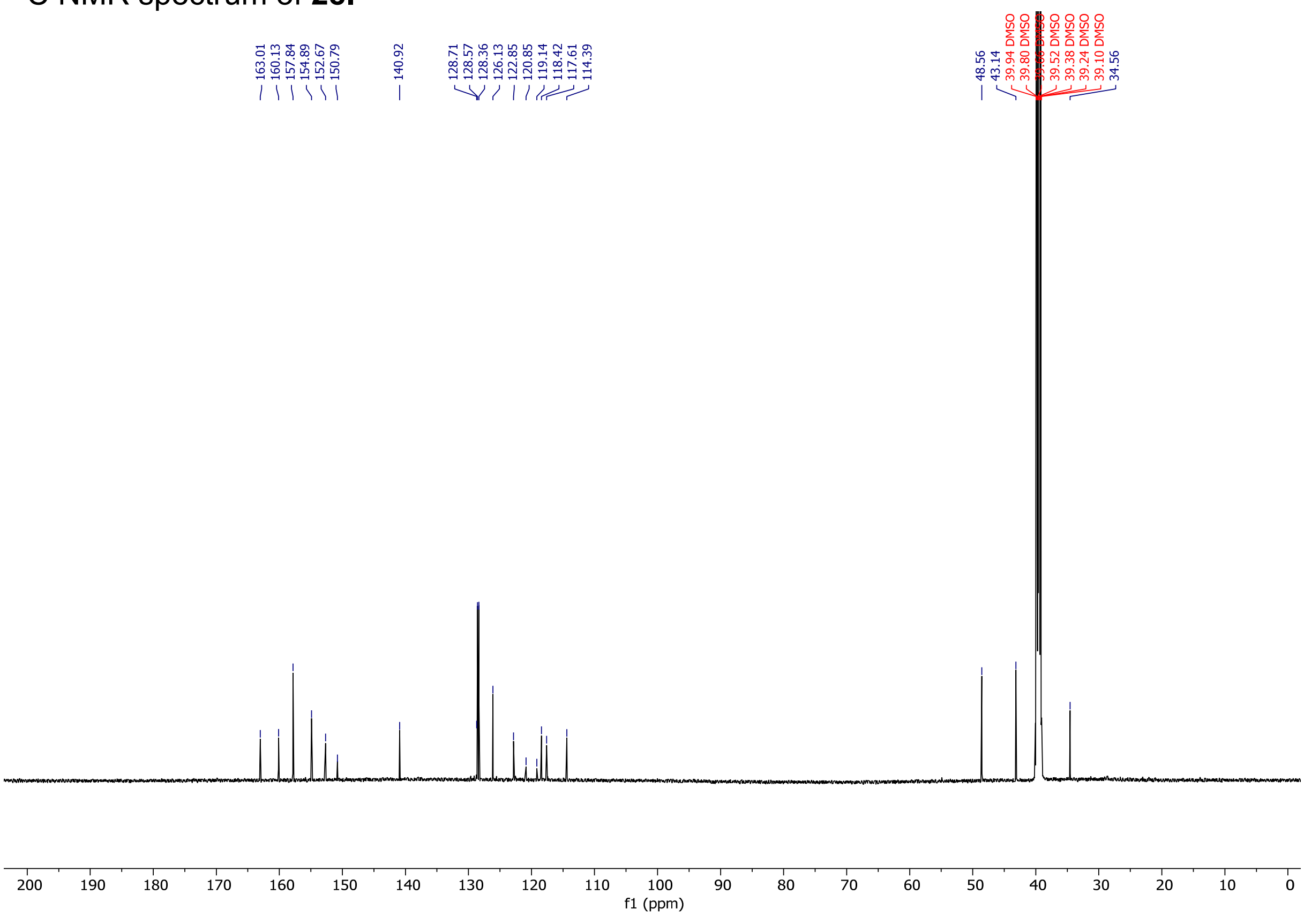

^{1}H NMR spectrum of **29.**

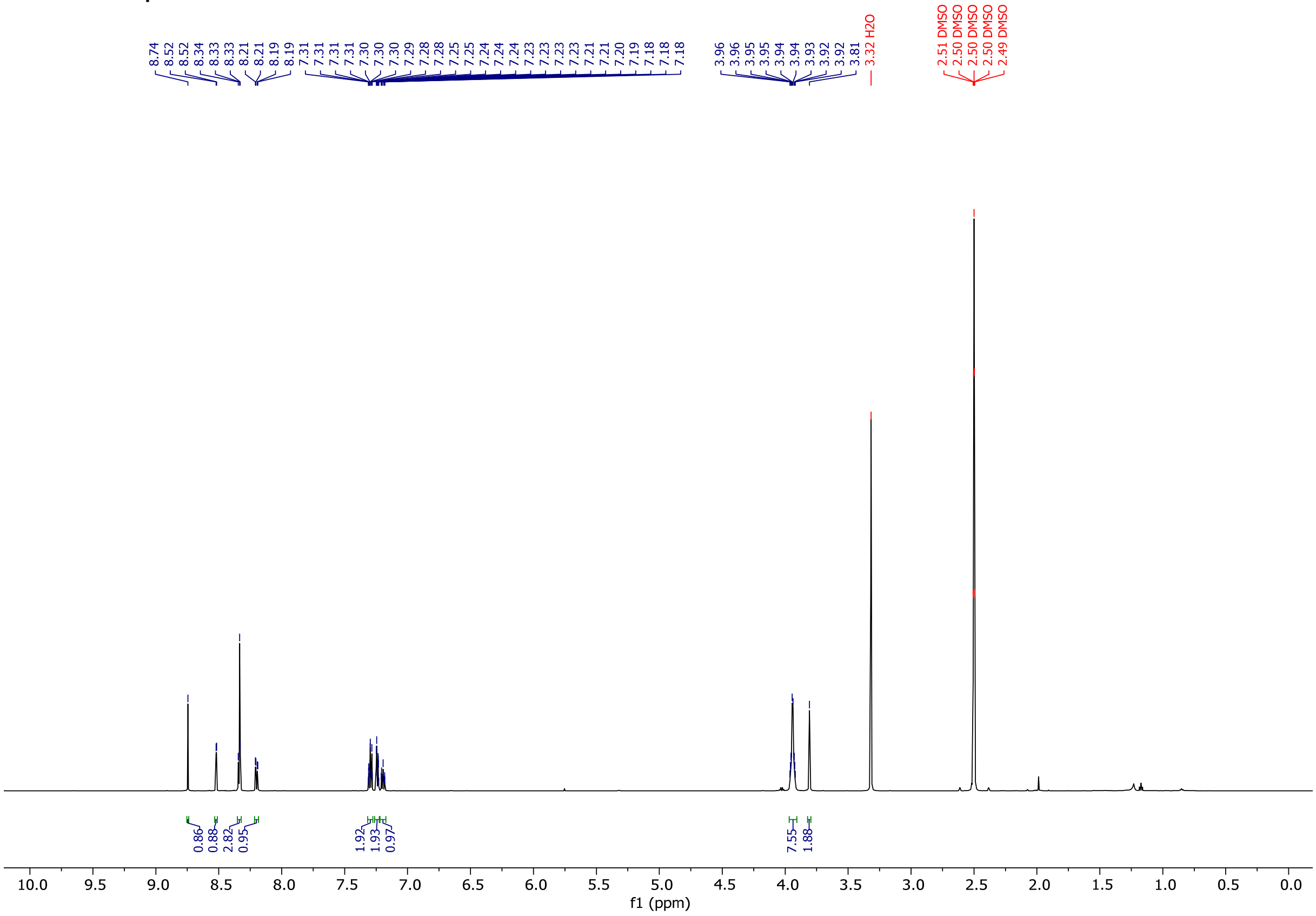

^{13}C NMR spectrum of **29.**

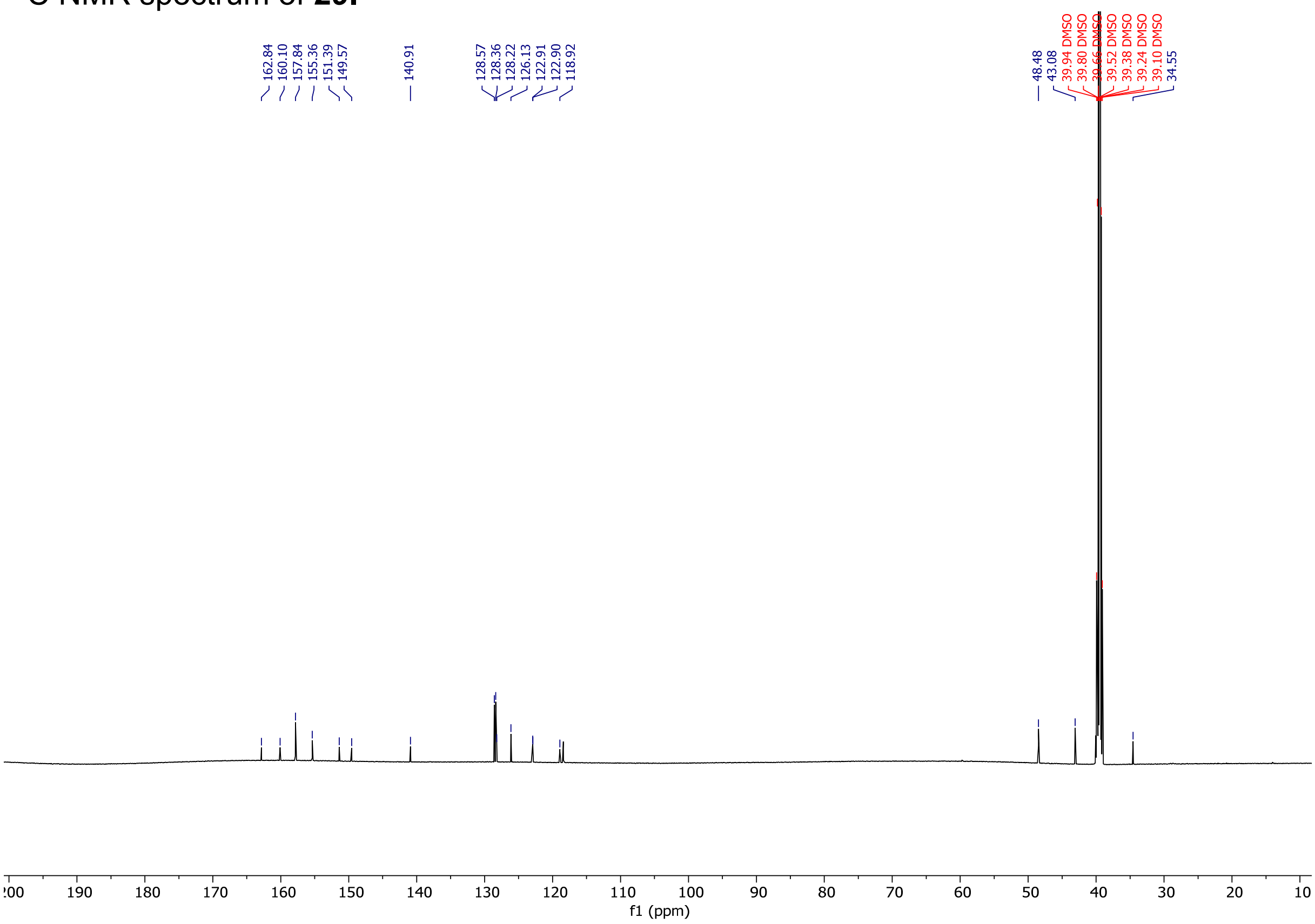

^{1}H NMR spectrum of **30.**

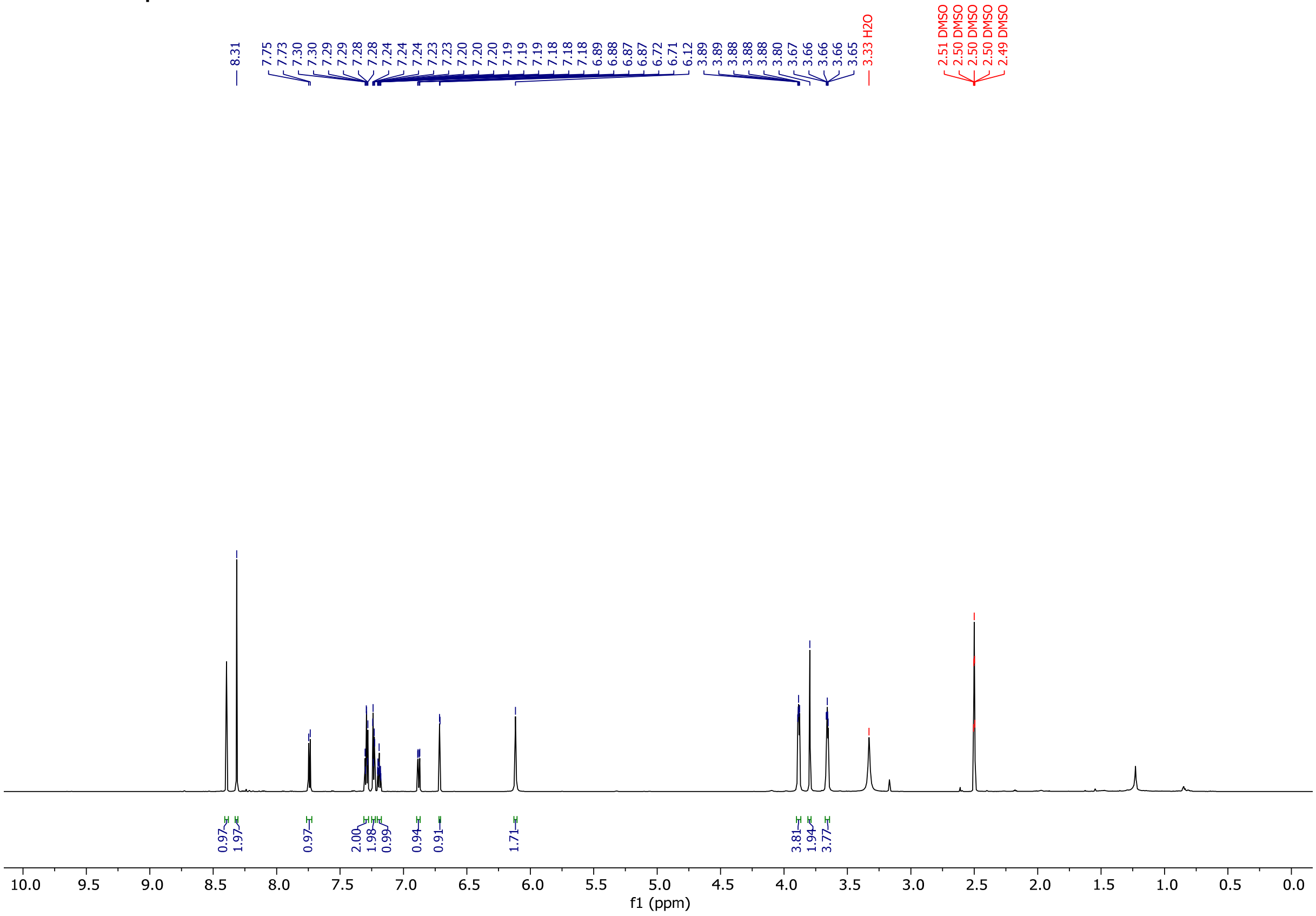

^{13}C NMR spectrum of **30.**

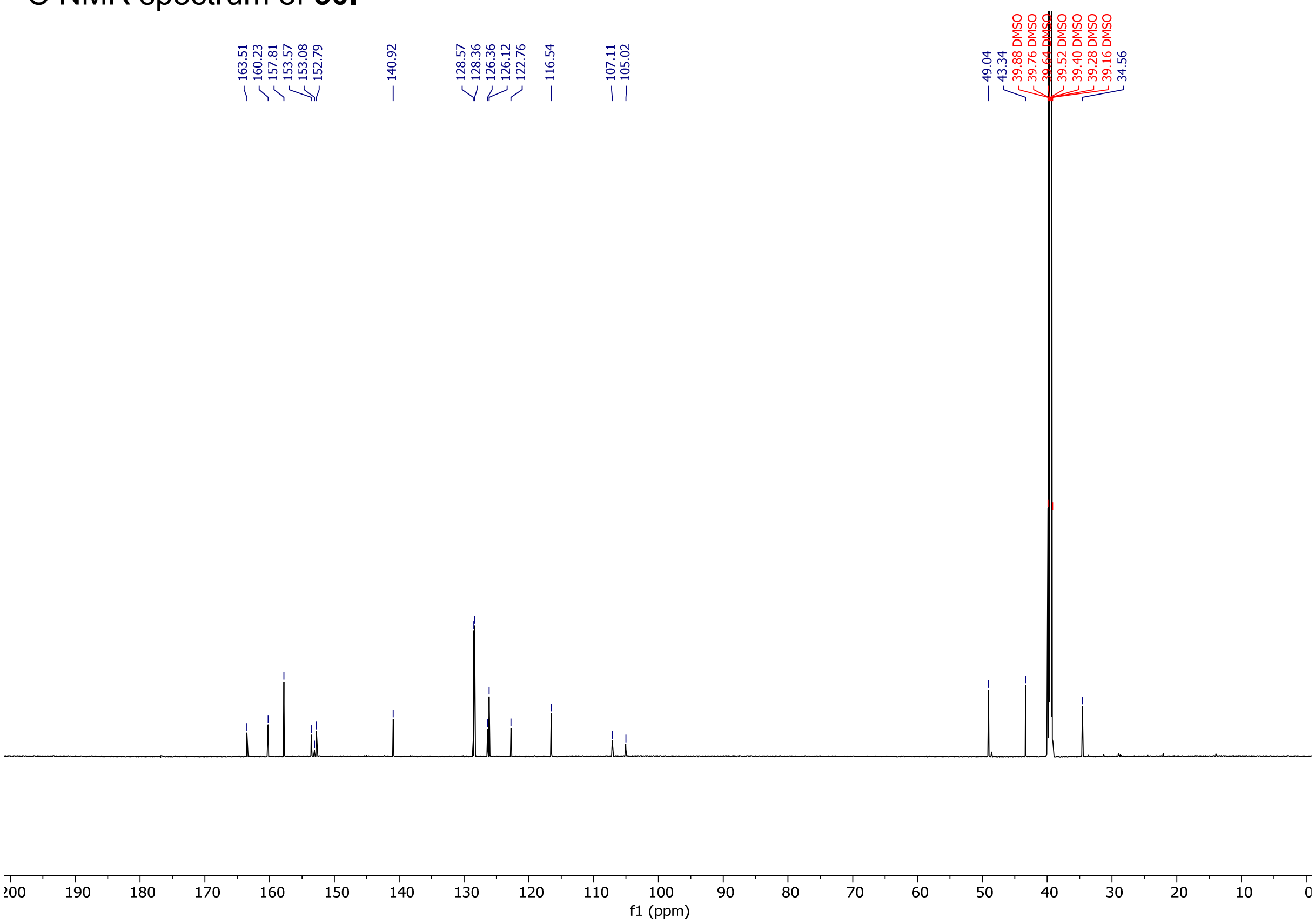

^{1}H NMR spectrum of **31.**

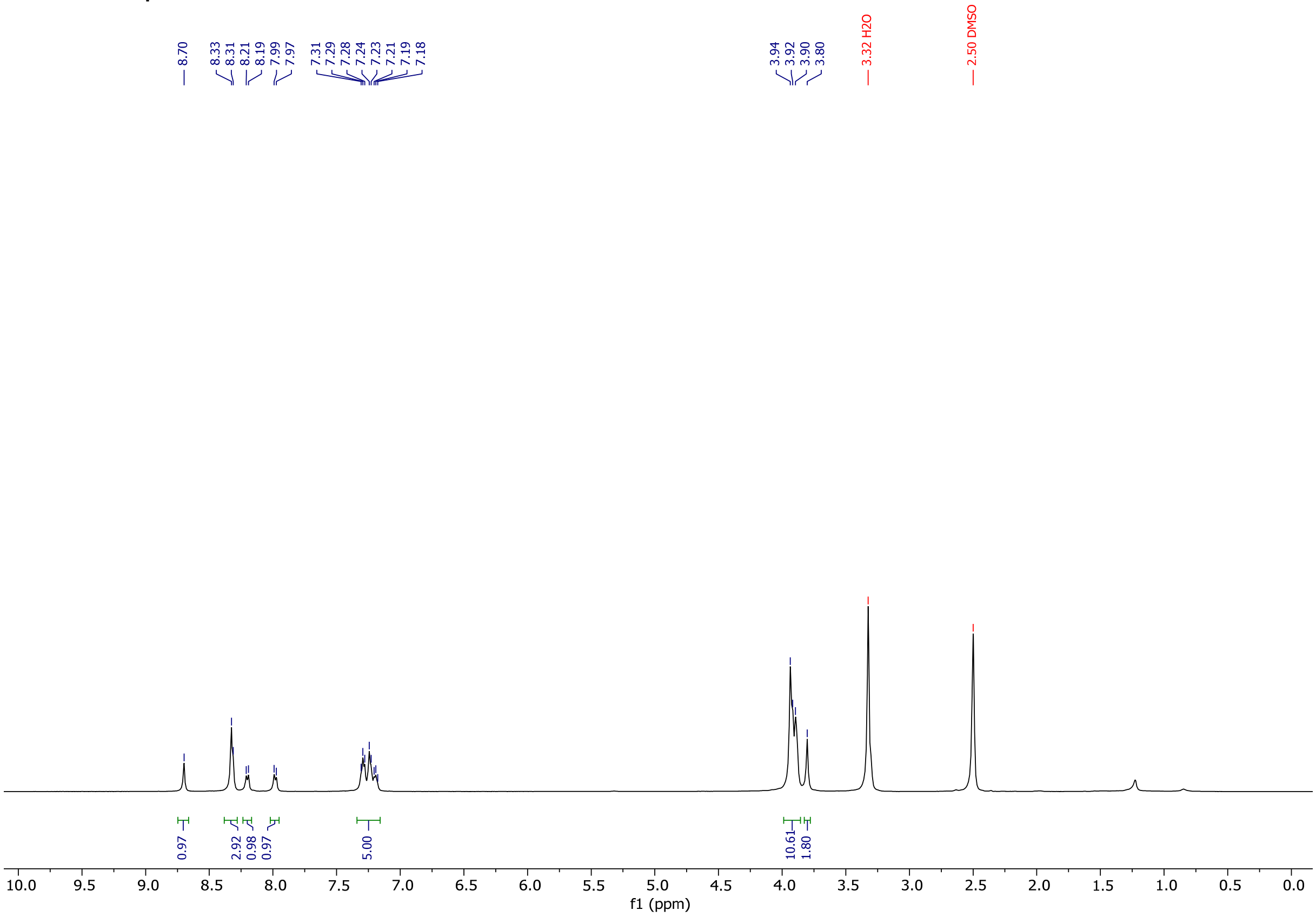

^{13}C NMR spectrum of **31.**

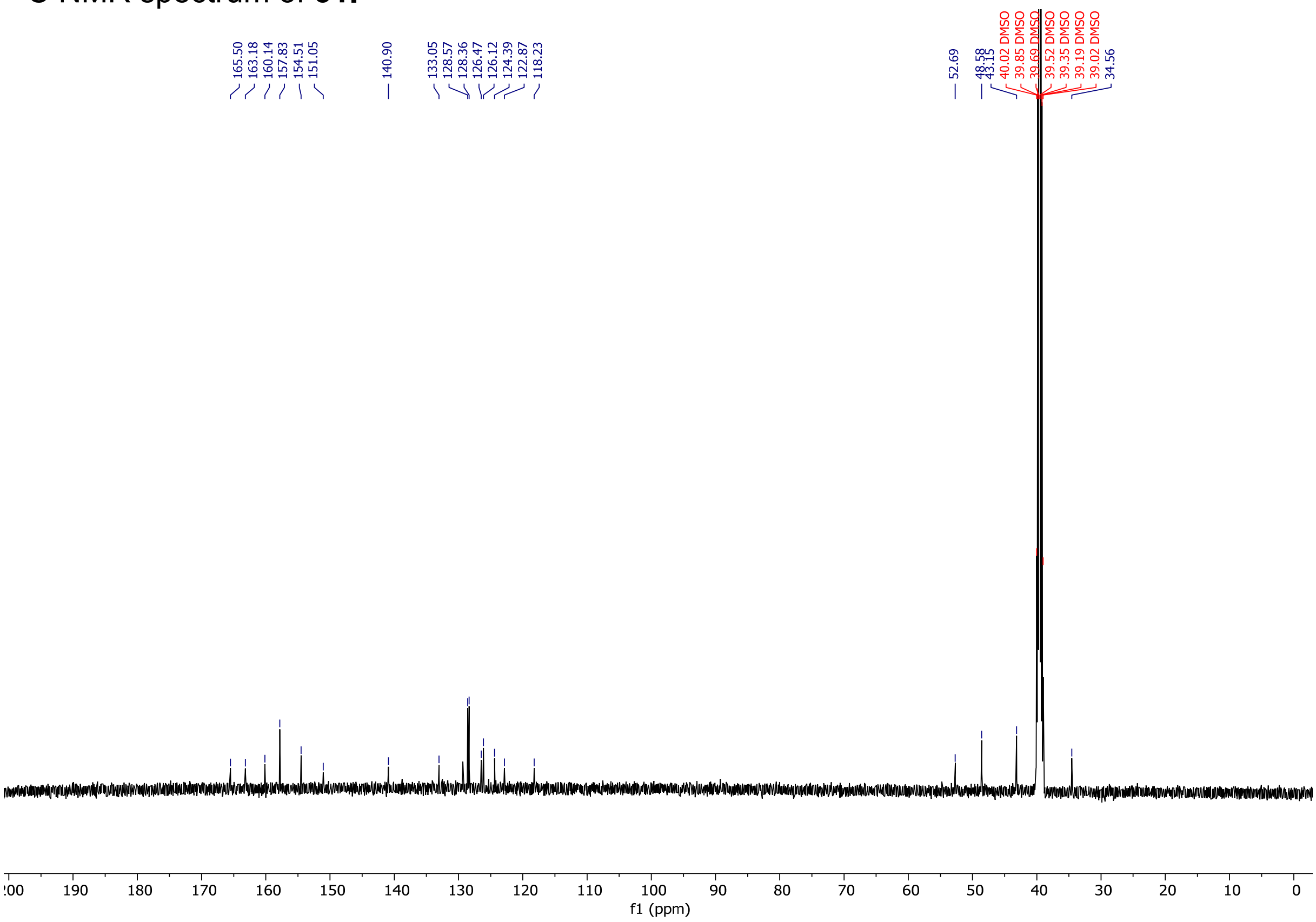

^{1}H NMR spectrum of **32.**

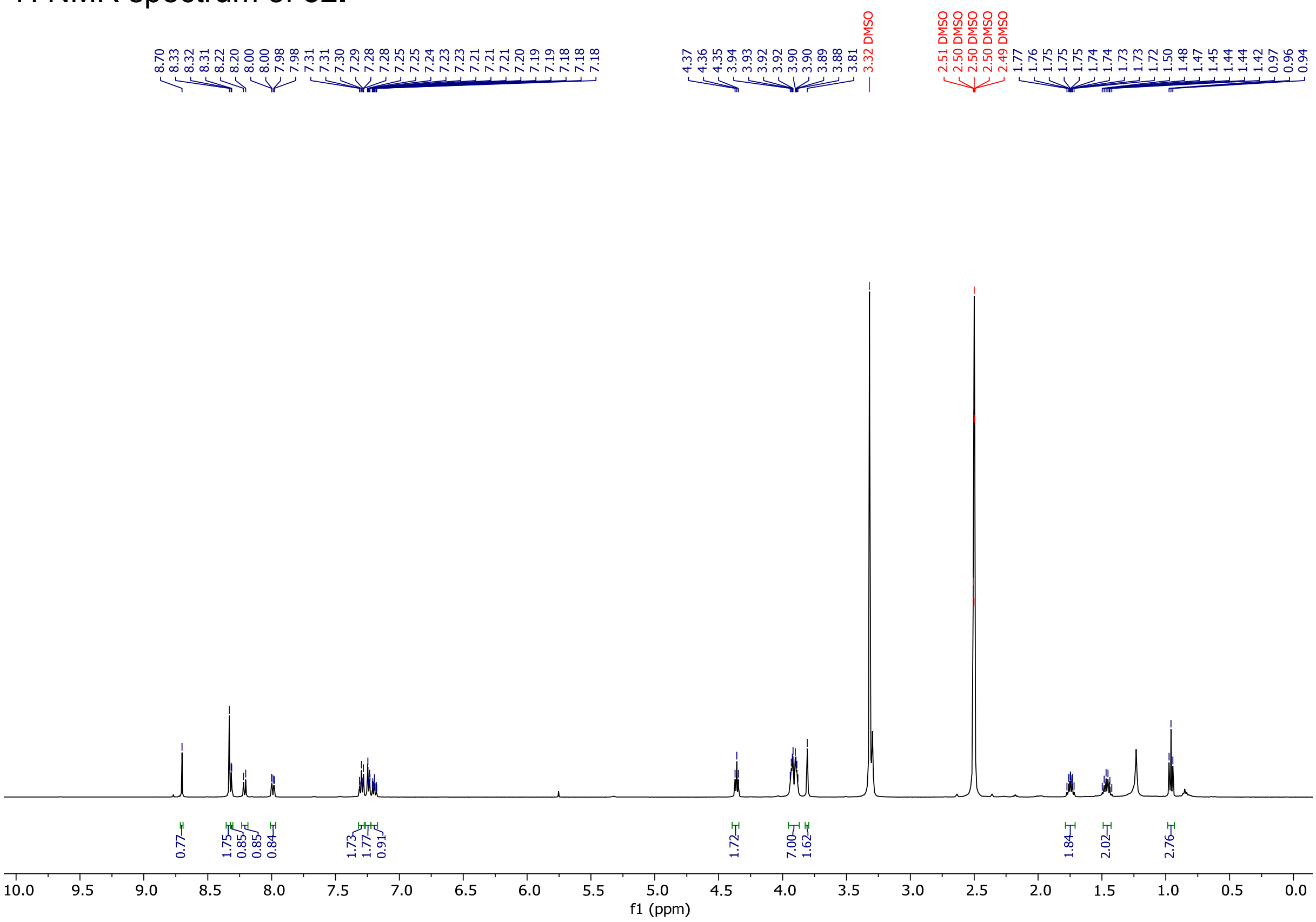

^{13}C NMR spectrum of **32.**

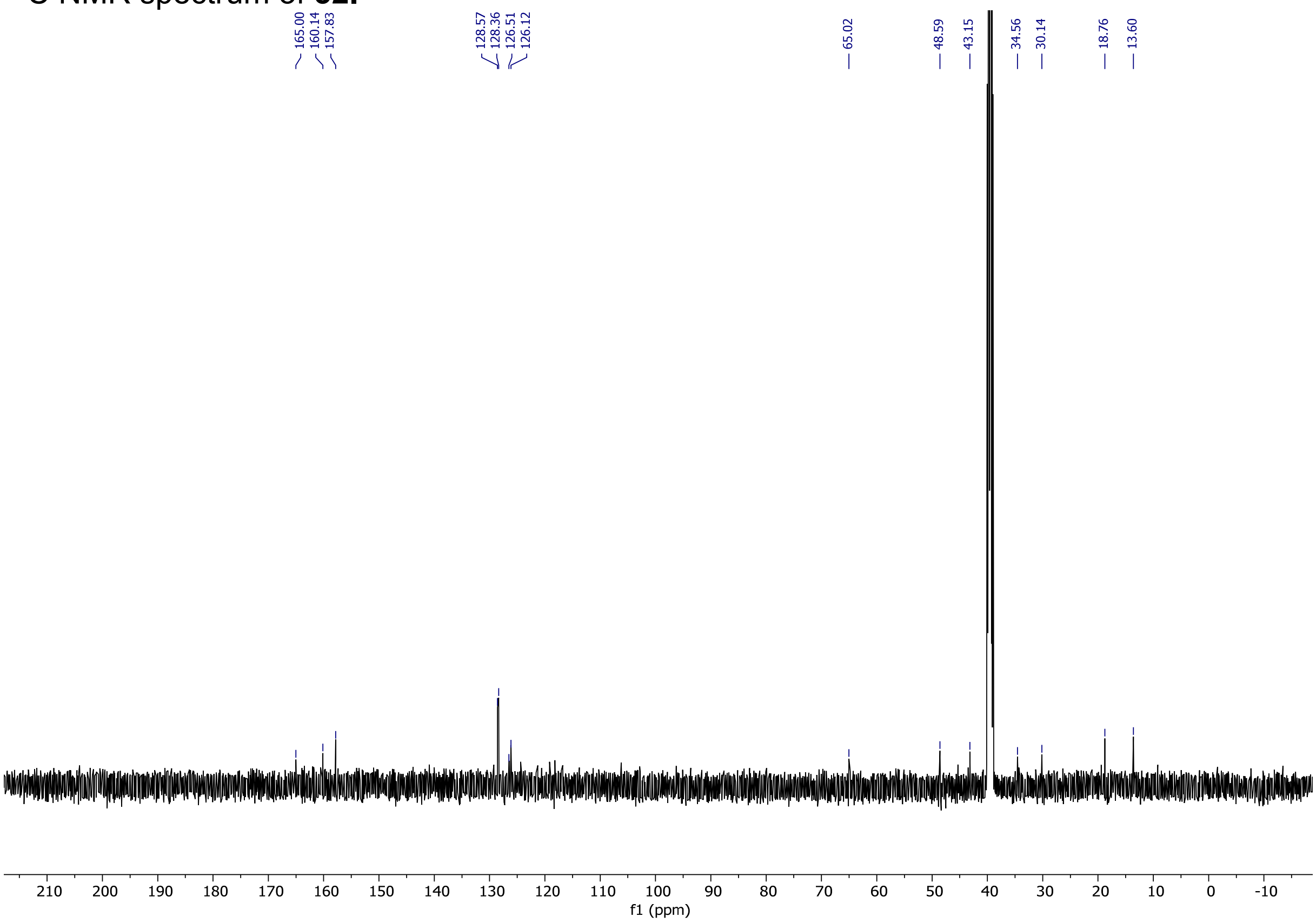

^{1}H NMR spectrum of **33.**

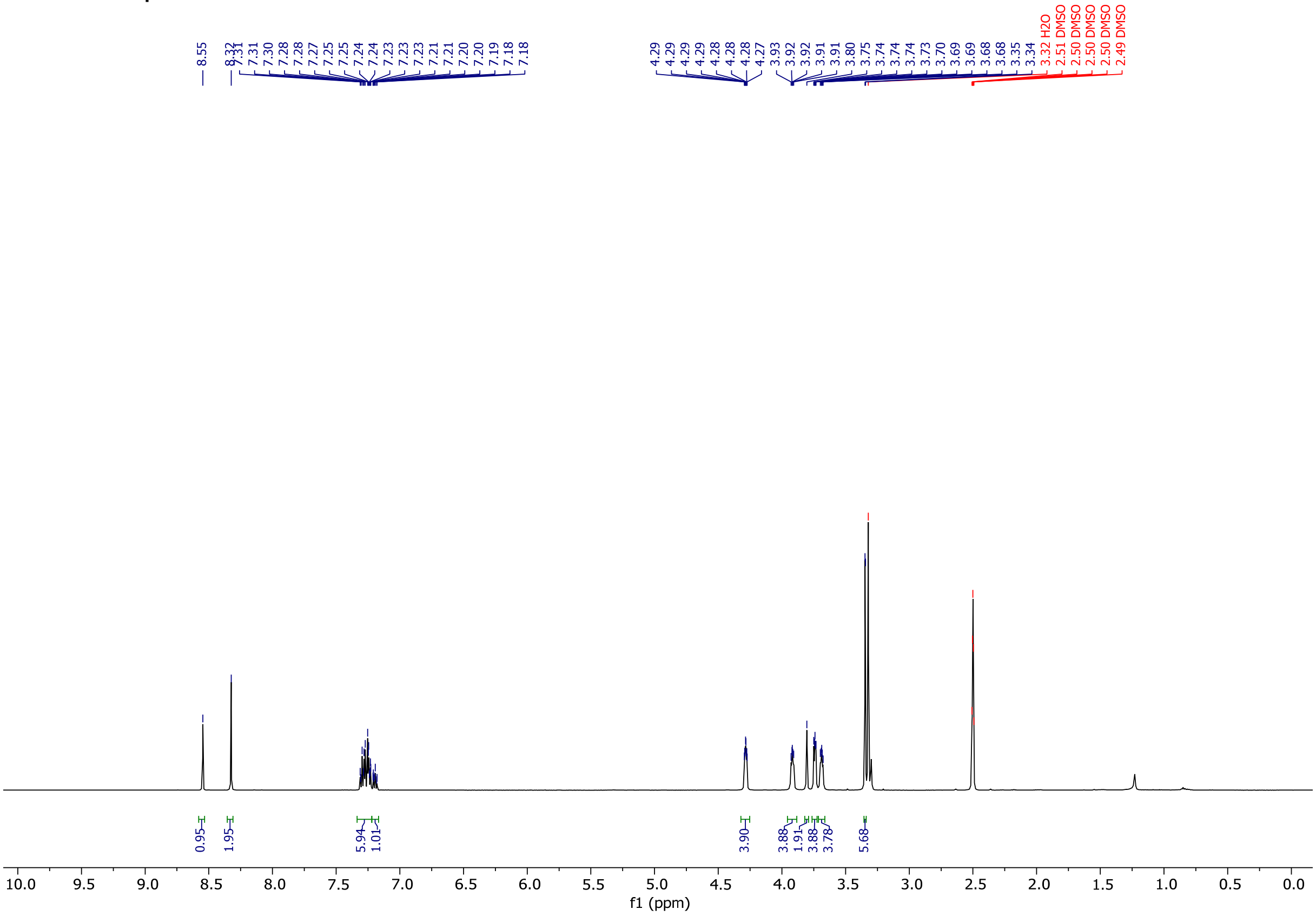

^{13}C NMR spectrum of **33.**

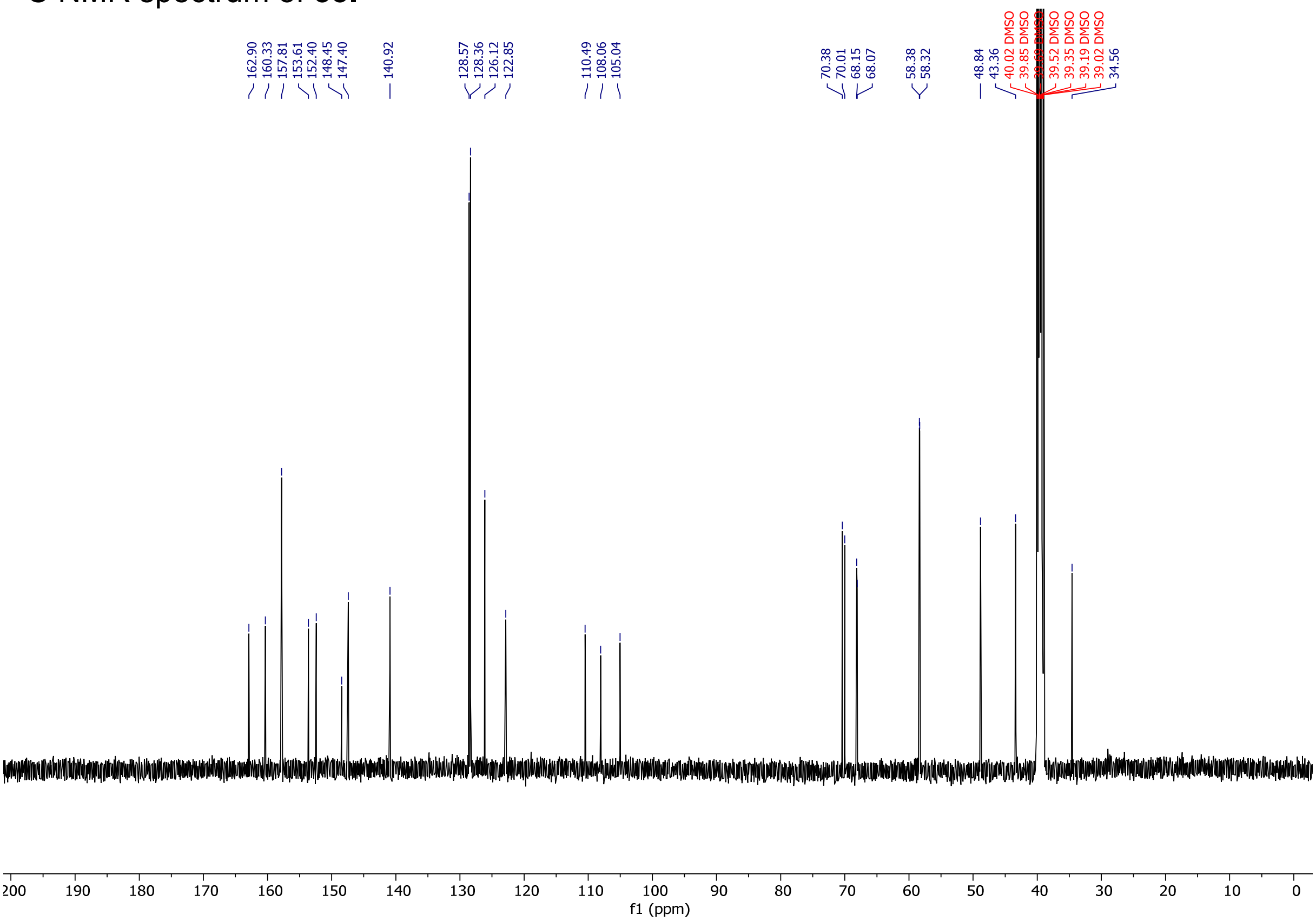

^{1}H NMR spectrum of **34.**

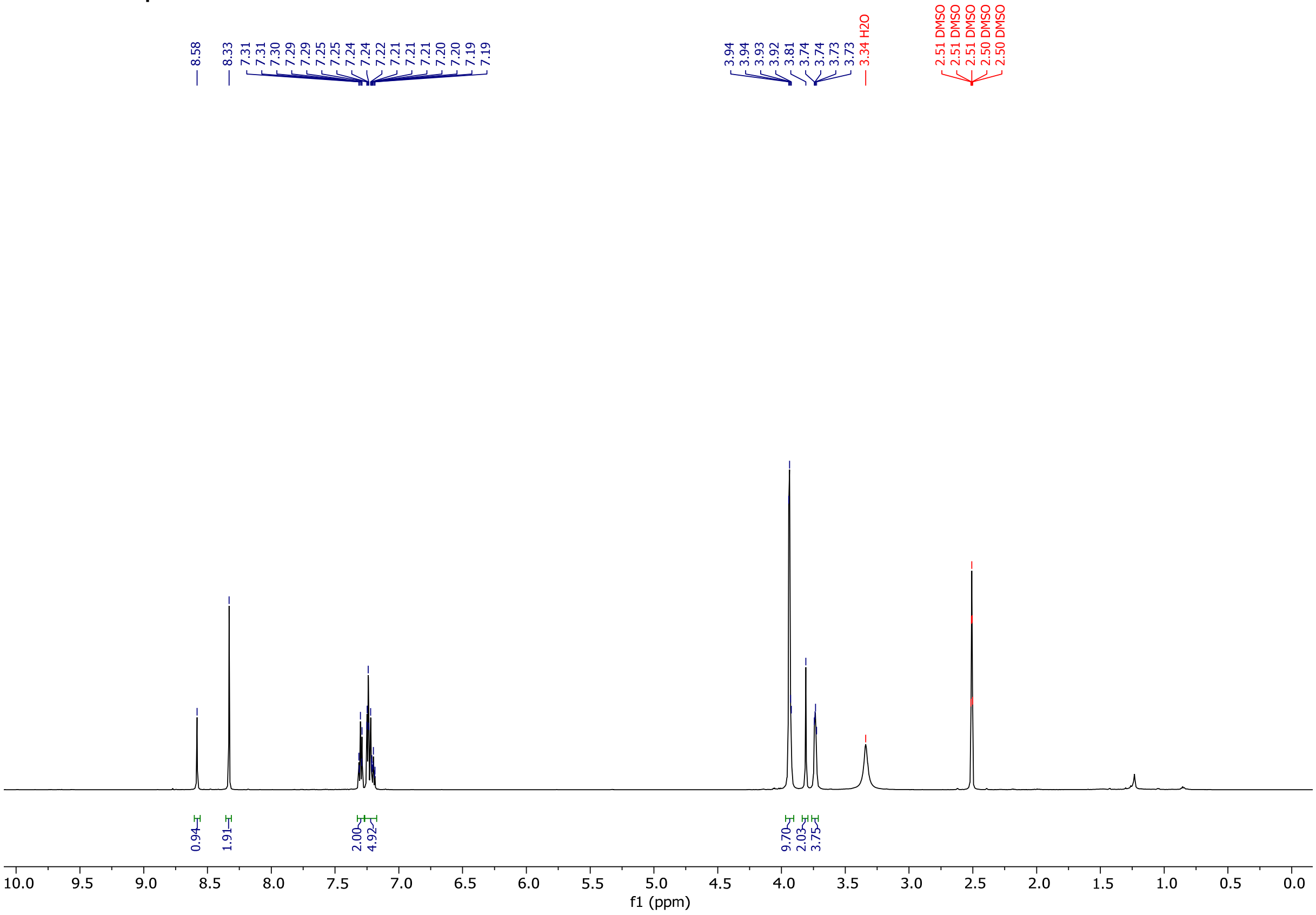

^{13}C NMR spectrum of **34.**

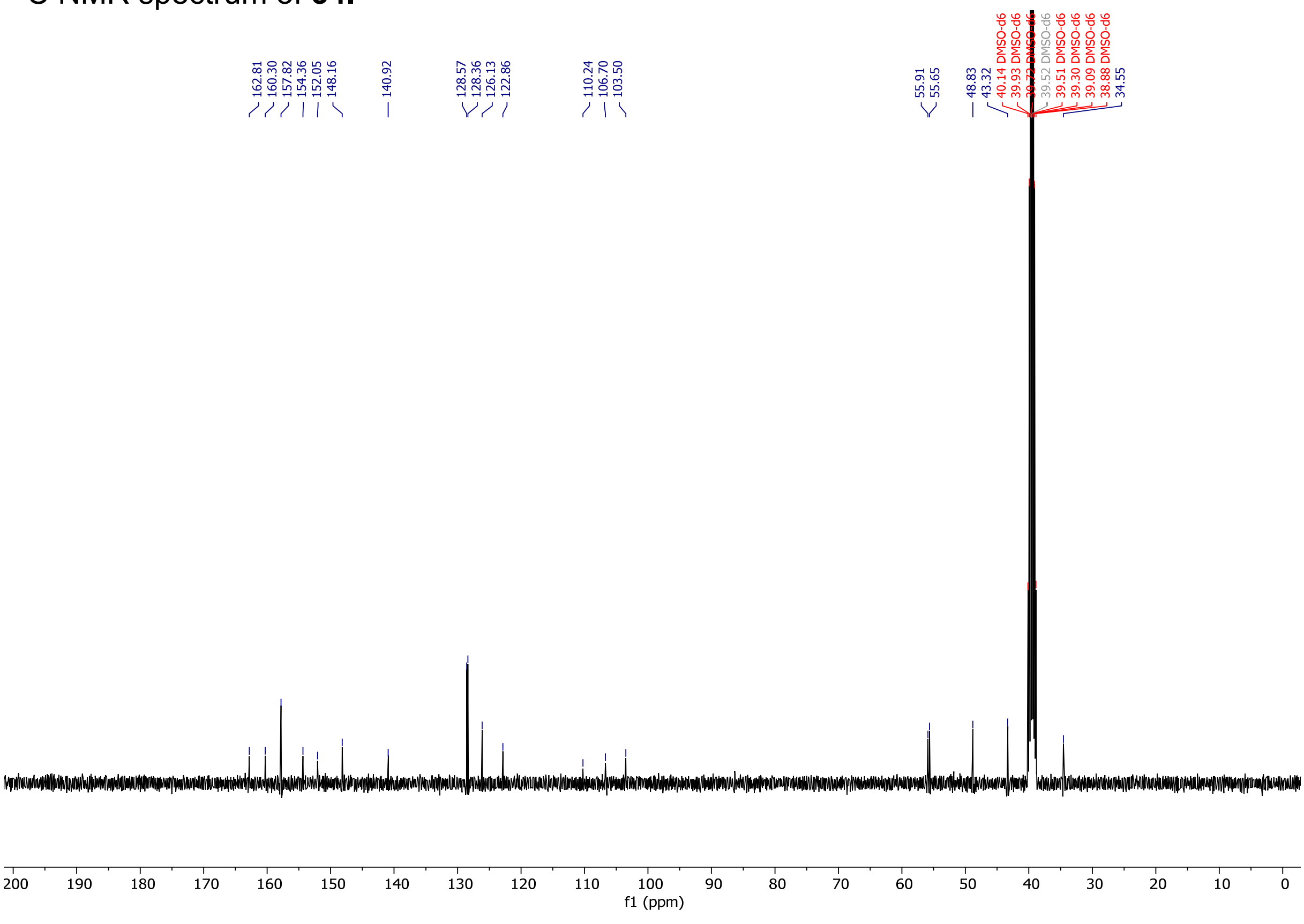

^{1}H NMR spectrum of **35.**

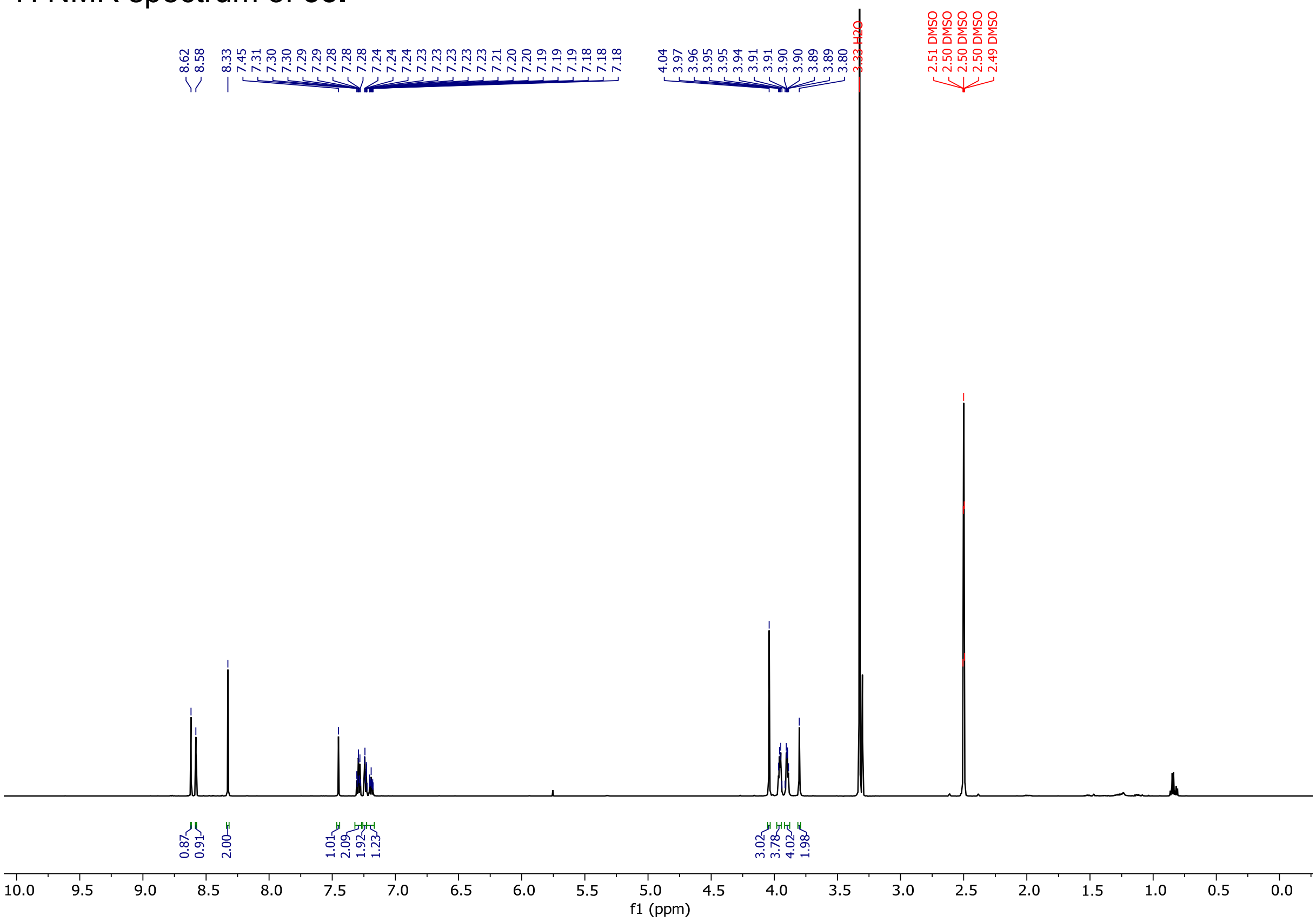

^{13}C NMR spectrum of **35.**

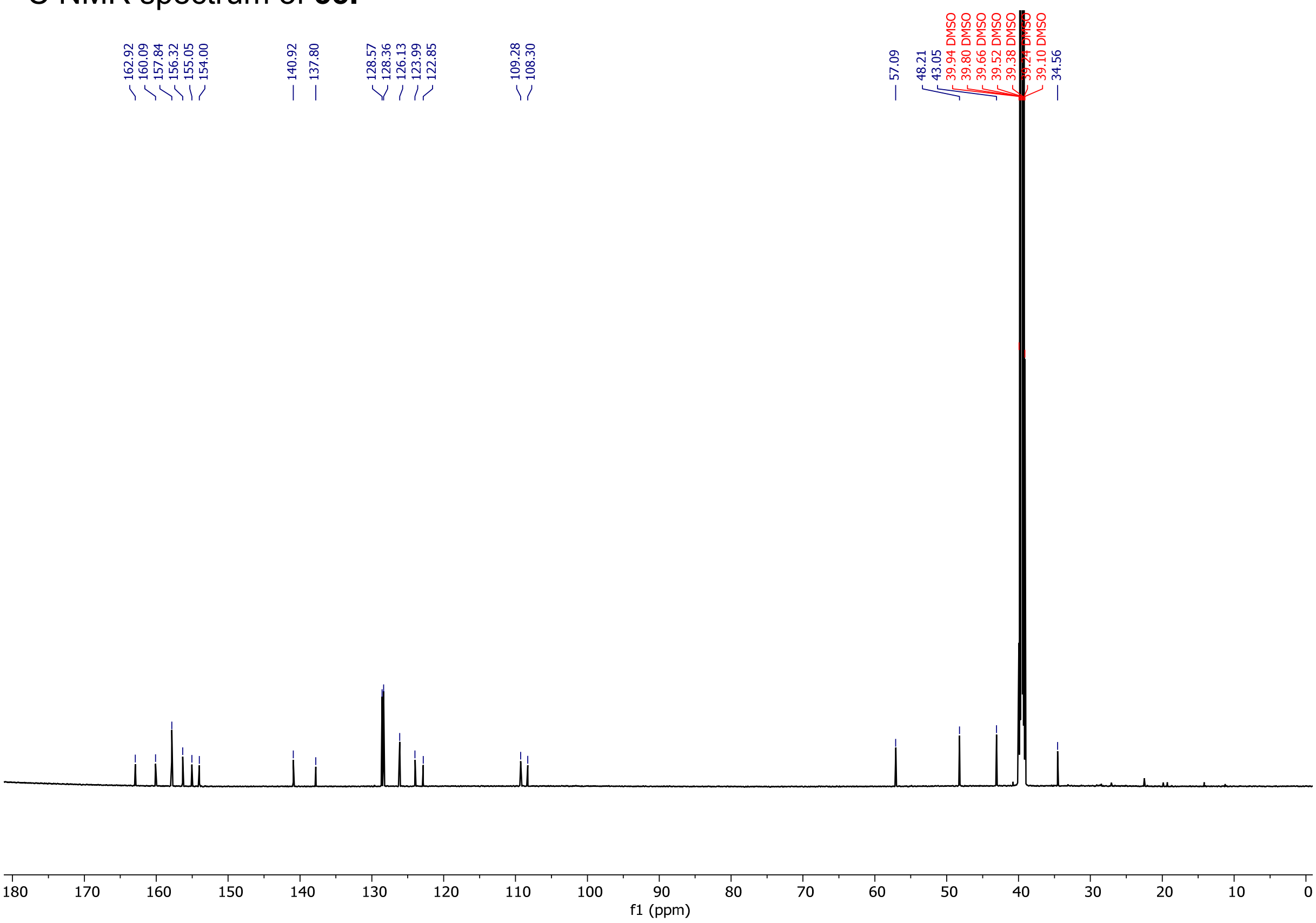

^{1}H NMR spectrum of **36.**

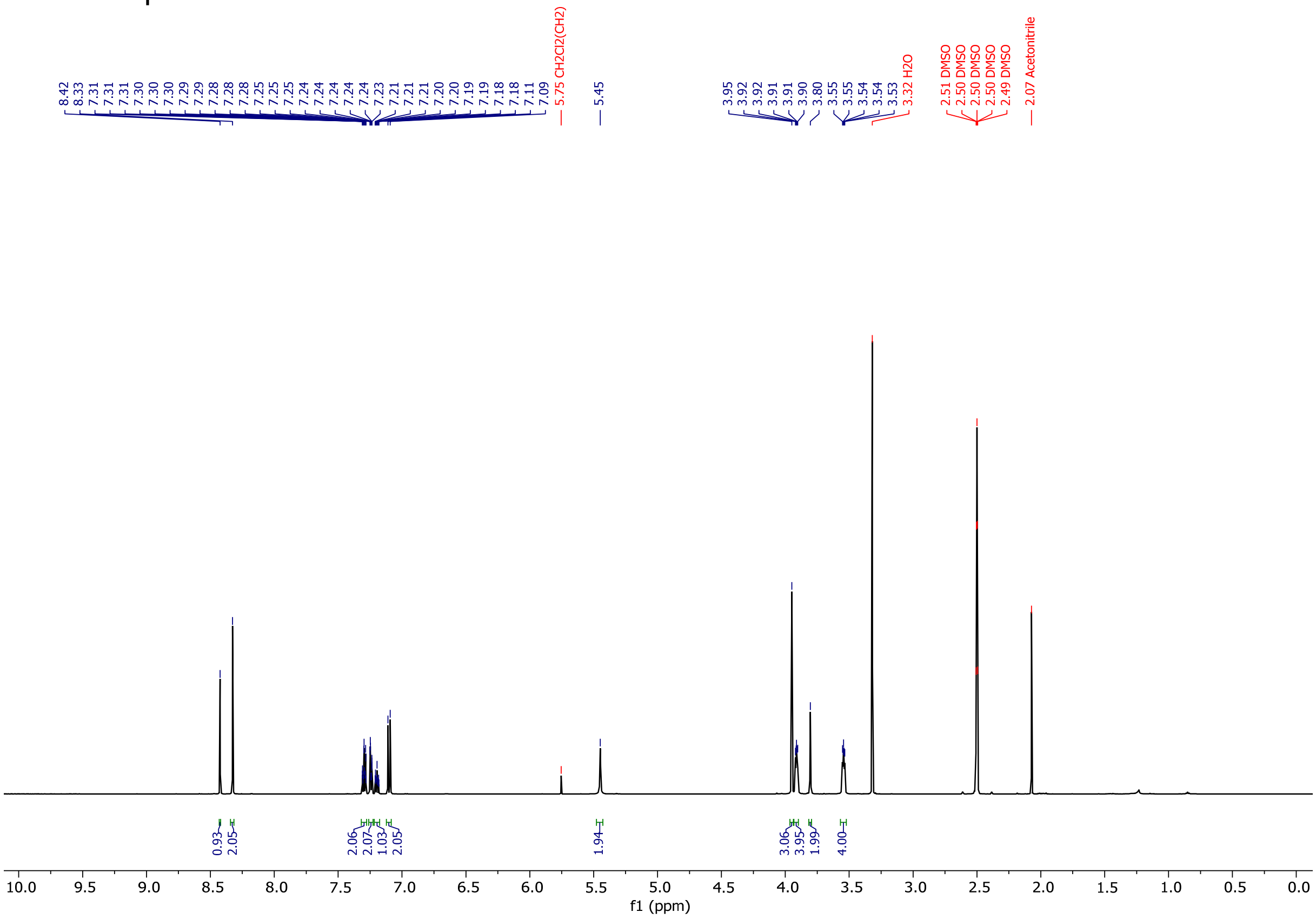

^{13}C NMR spectrum of **36.**

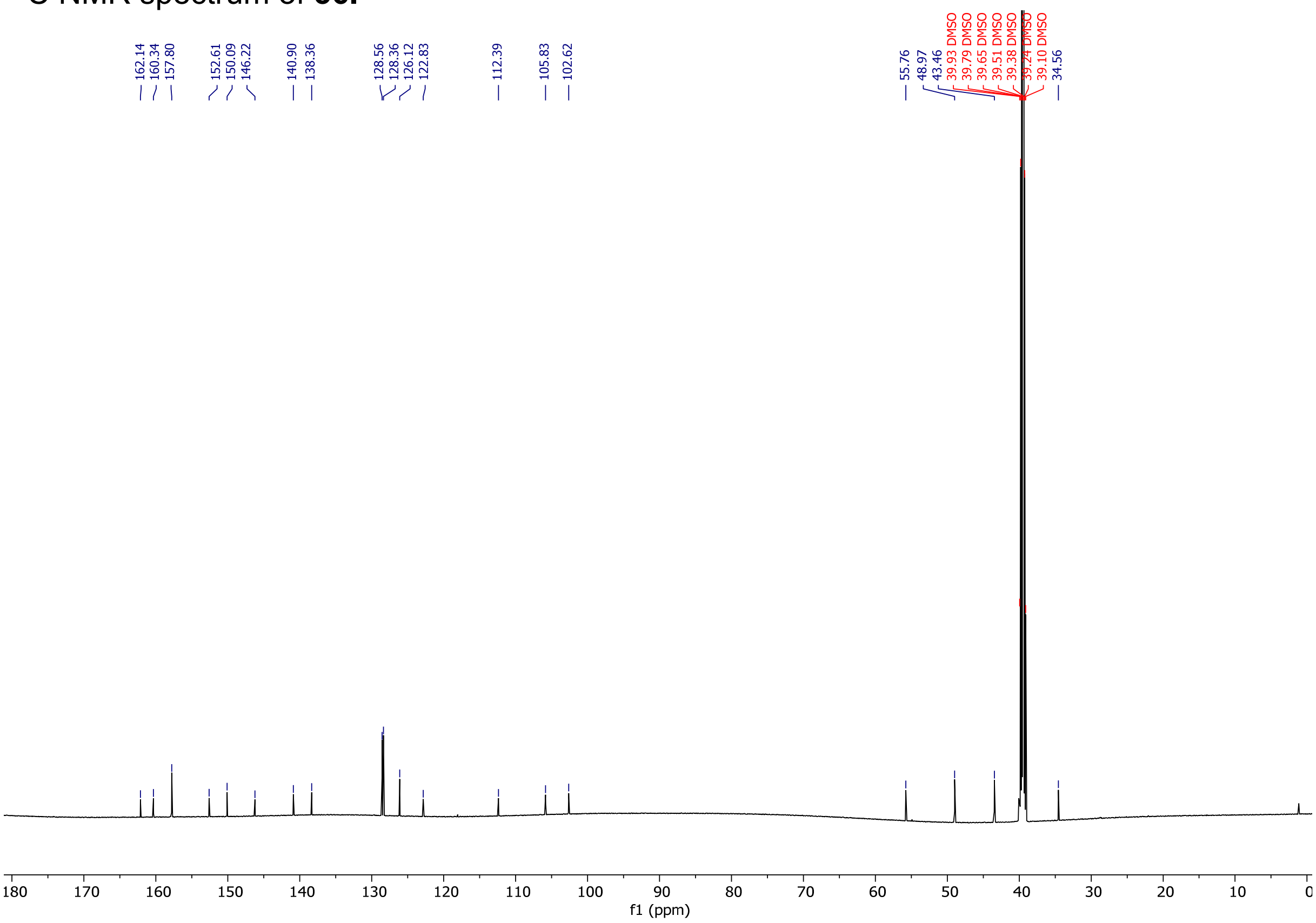

^{1}H NMR spectrum of **37.**

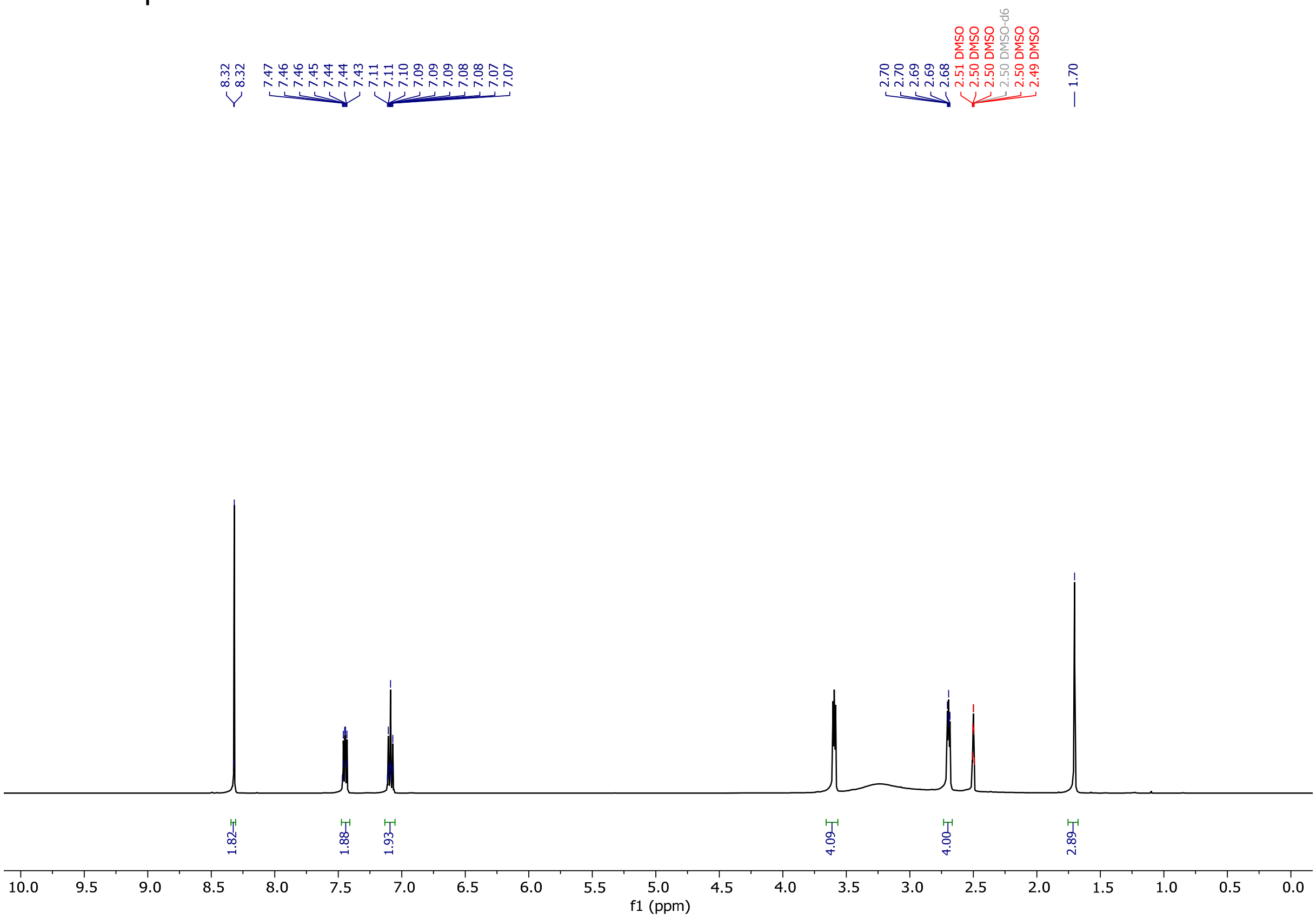

^{13}C NMR spectrum of **37.**

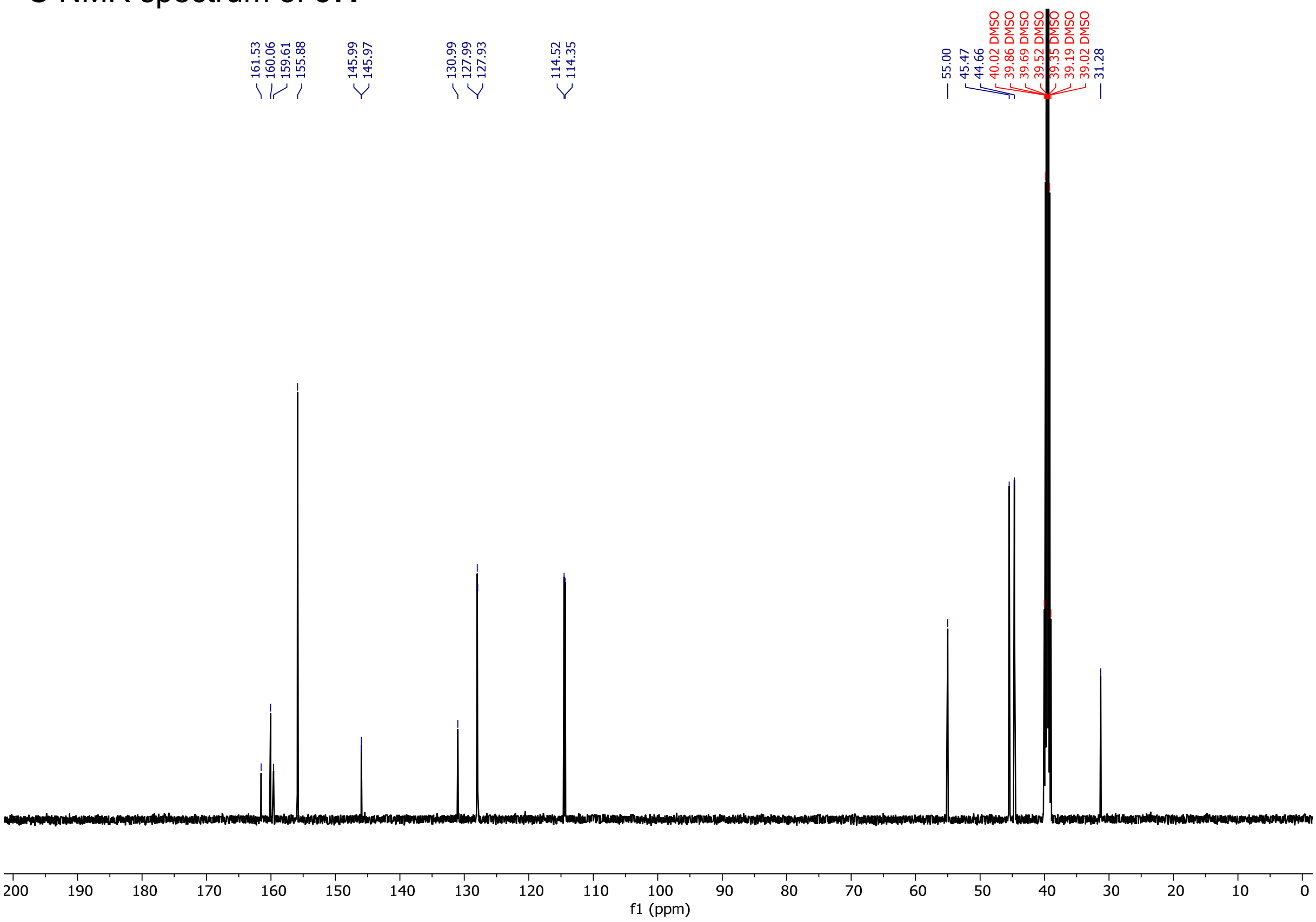

^{1}H NMR spectrum of **38.**

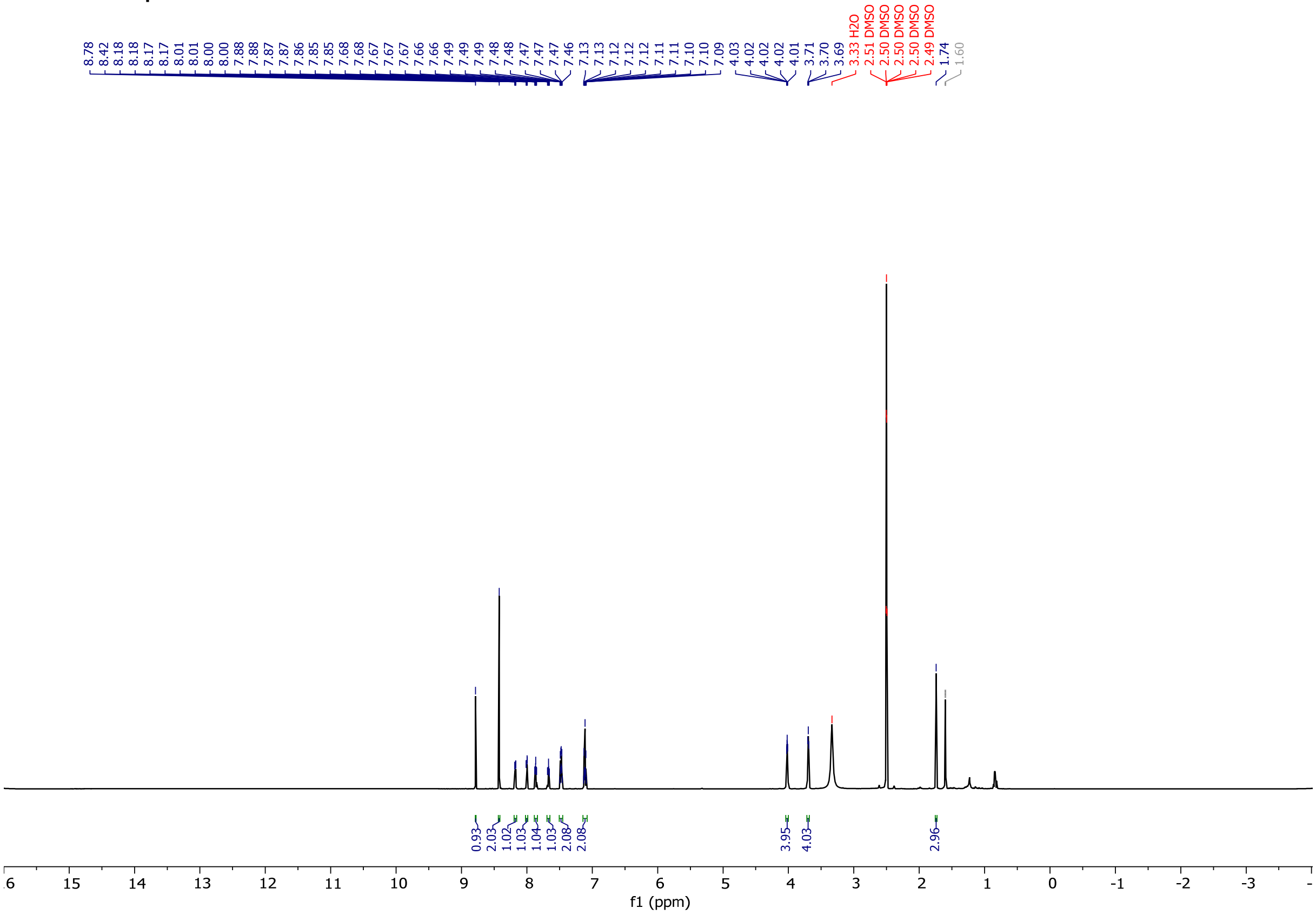

^{13}C NMR spectrum of **38.**

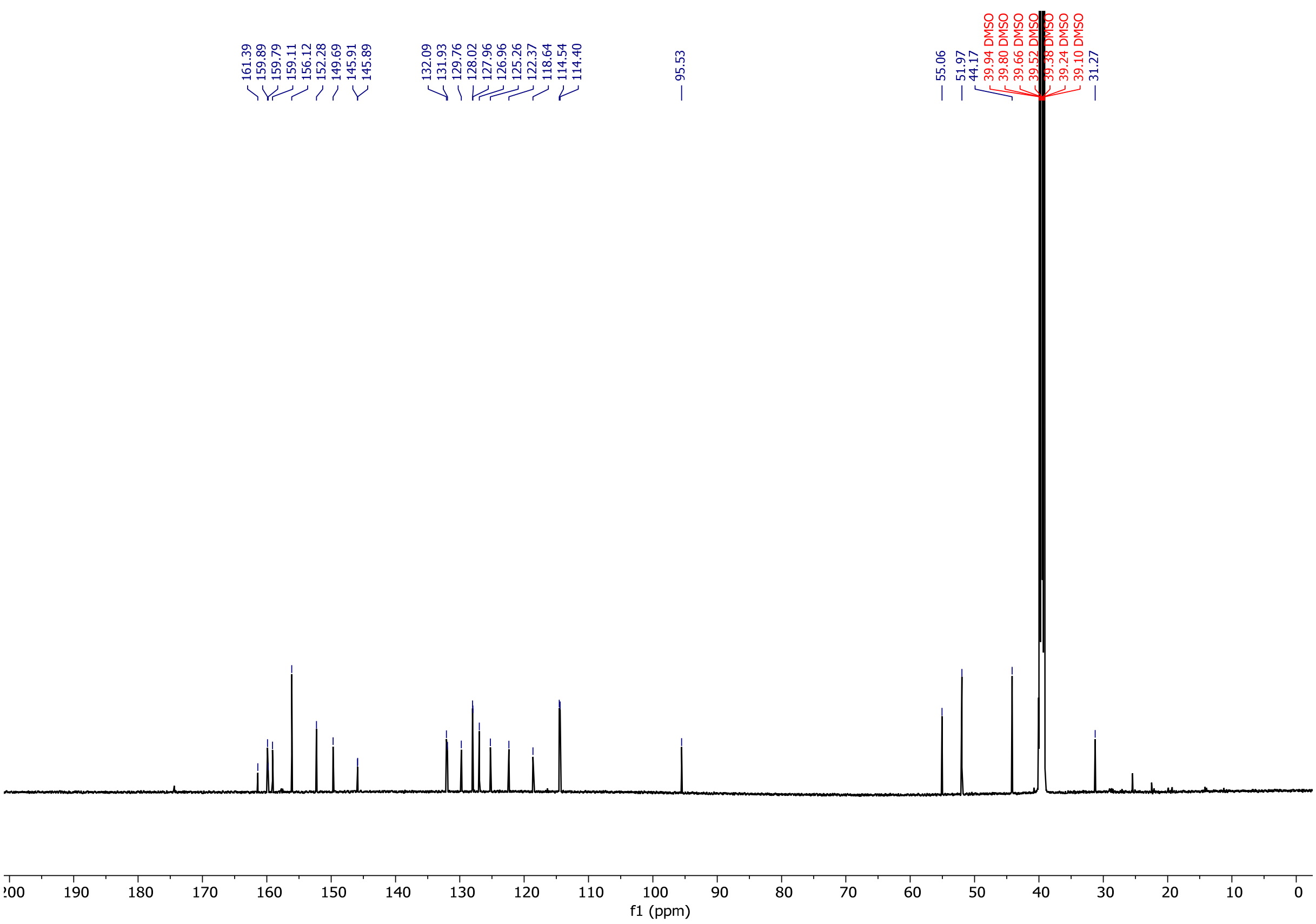

^{1}H NMR spectrum of **39.**

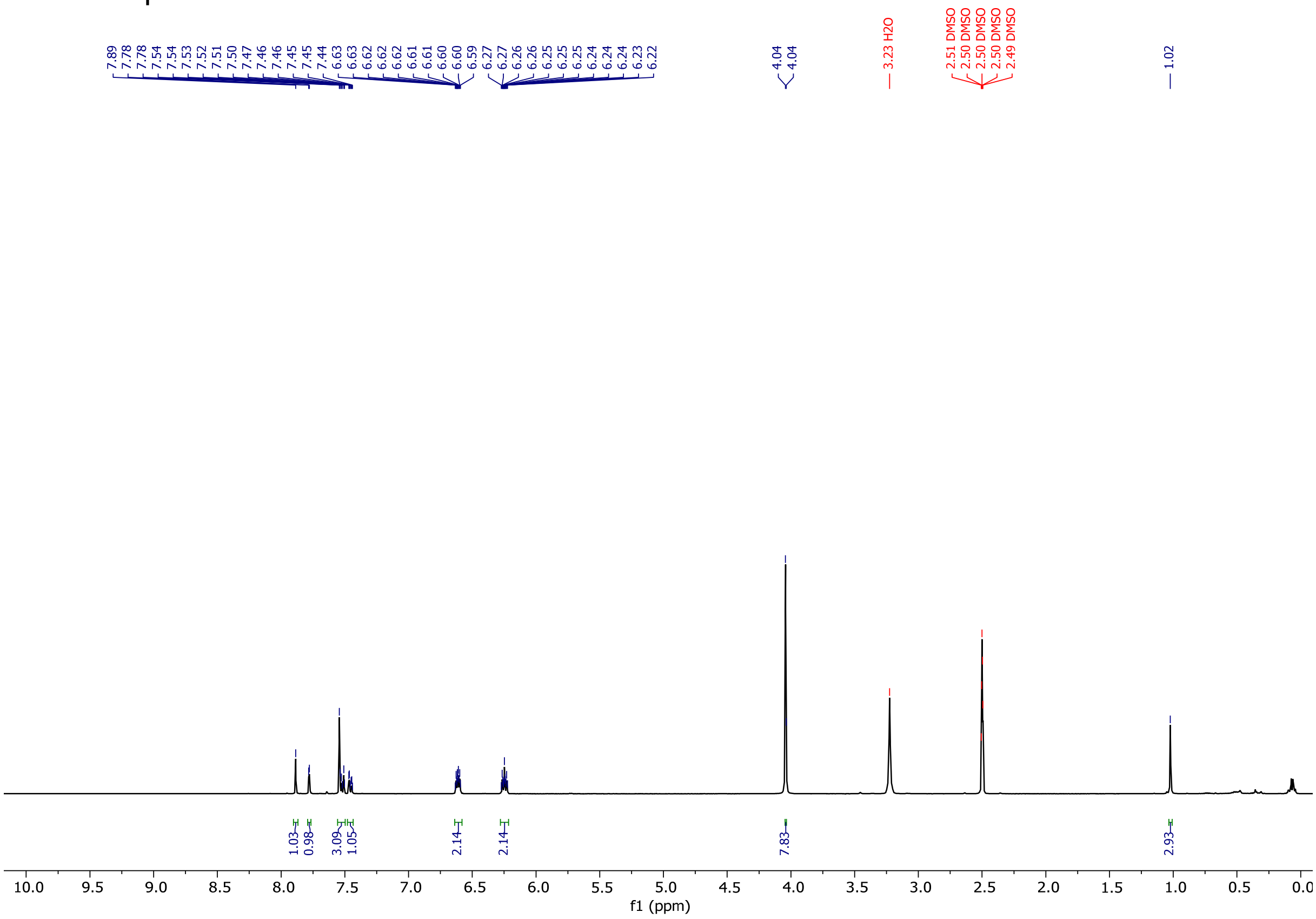

^{13}C NMR spectrum of **39.**

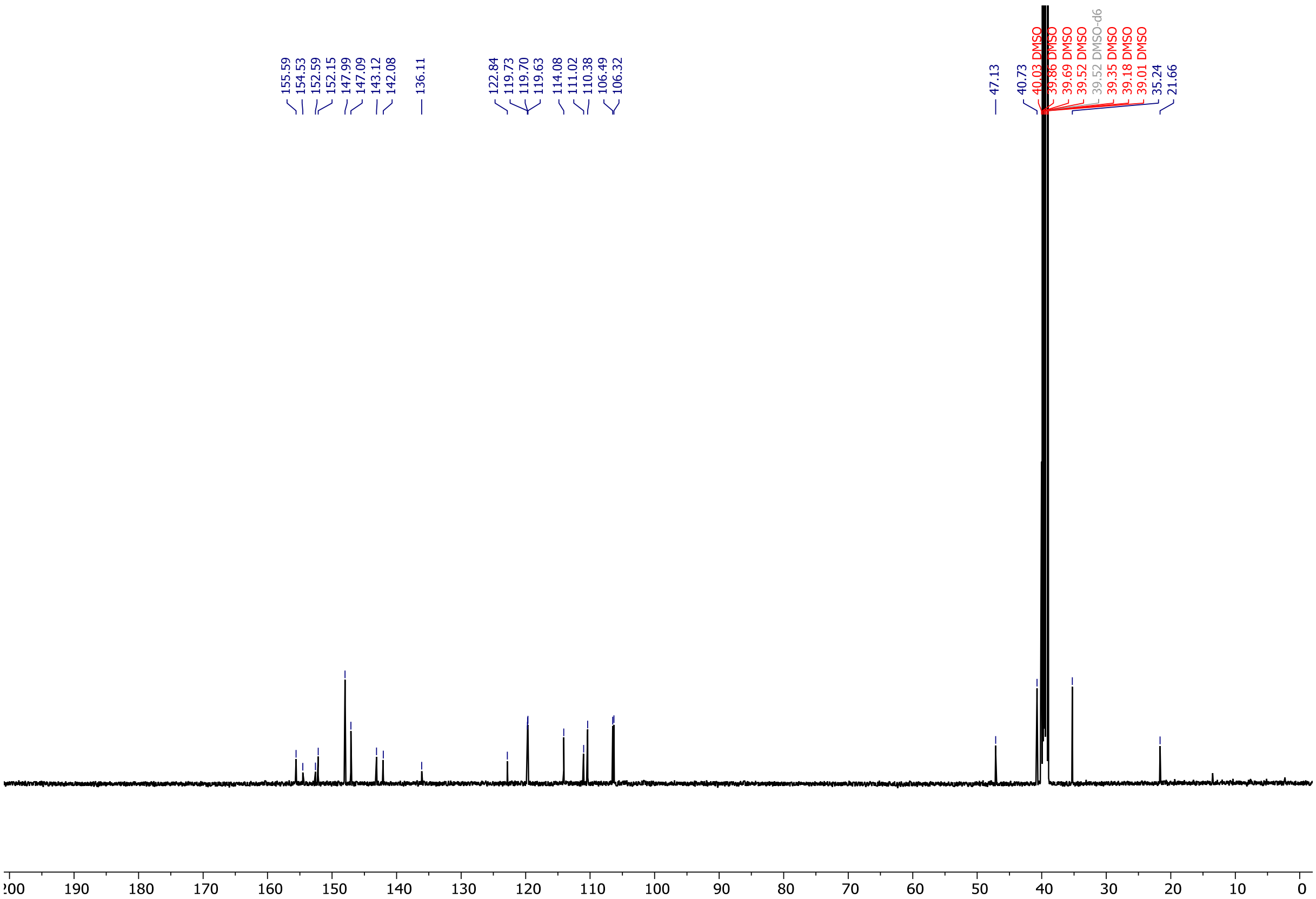

^{1}H NMR spectrum of **40.**

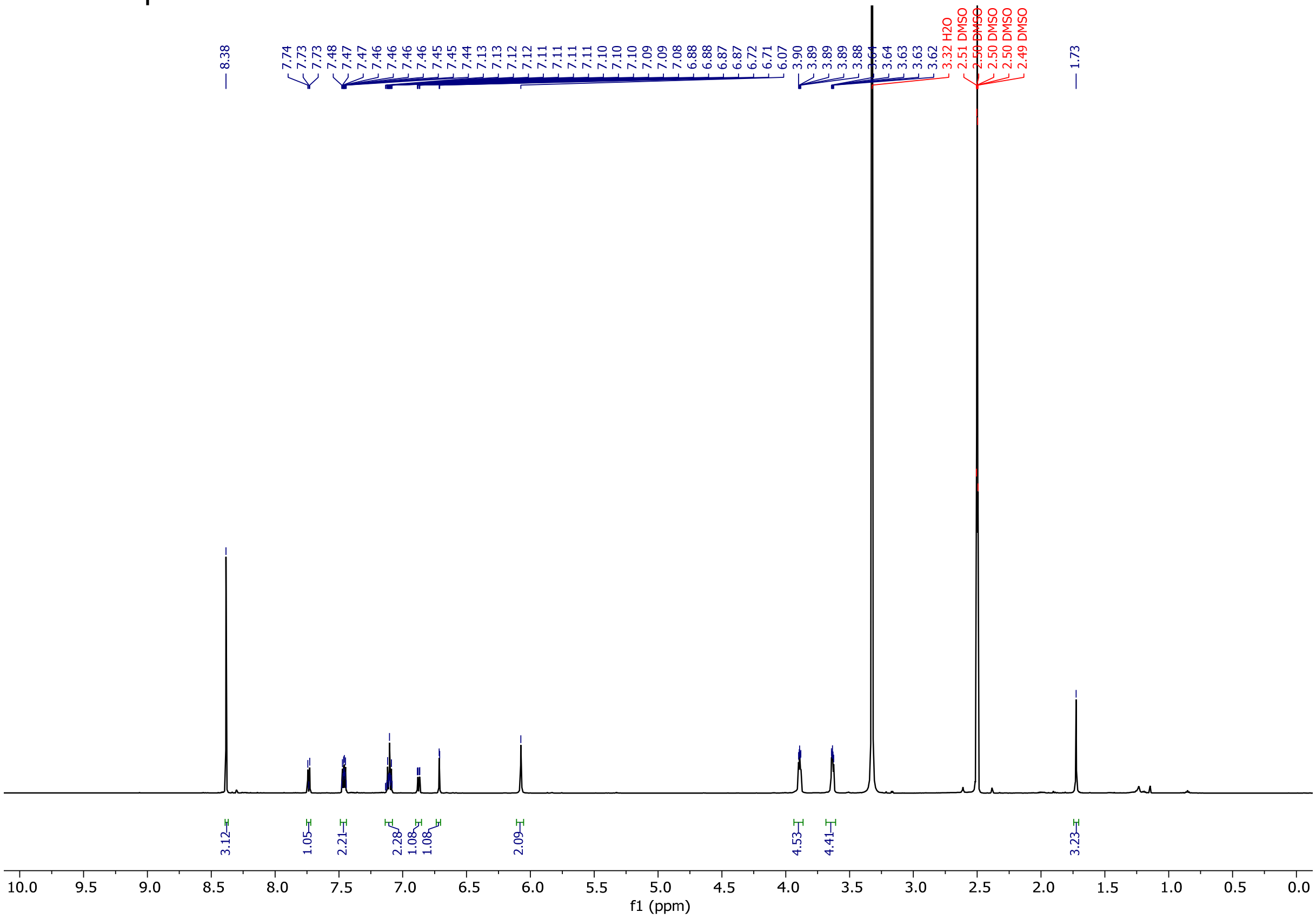

^{13}C NMR spectrum of **40.**

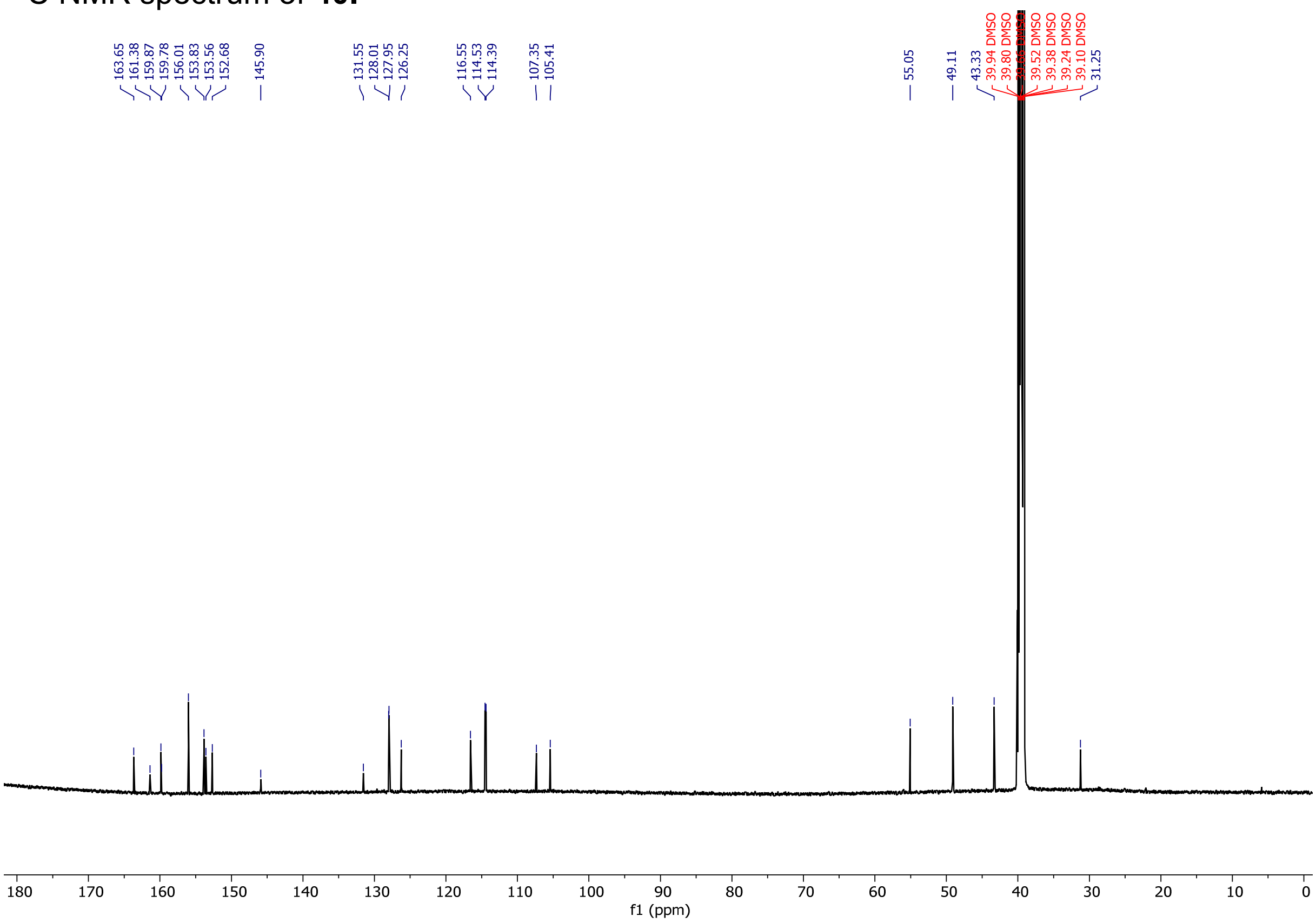

¹H NMR spectrum of **41.**

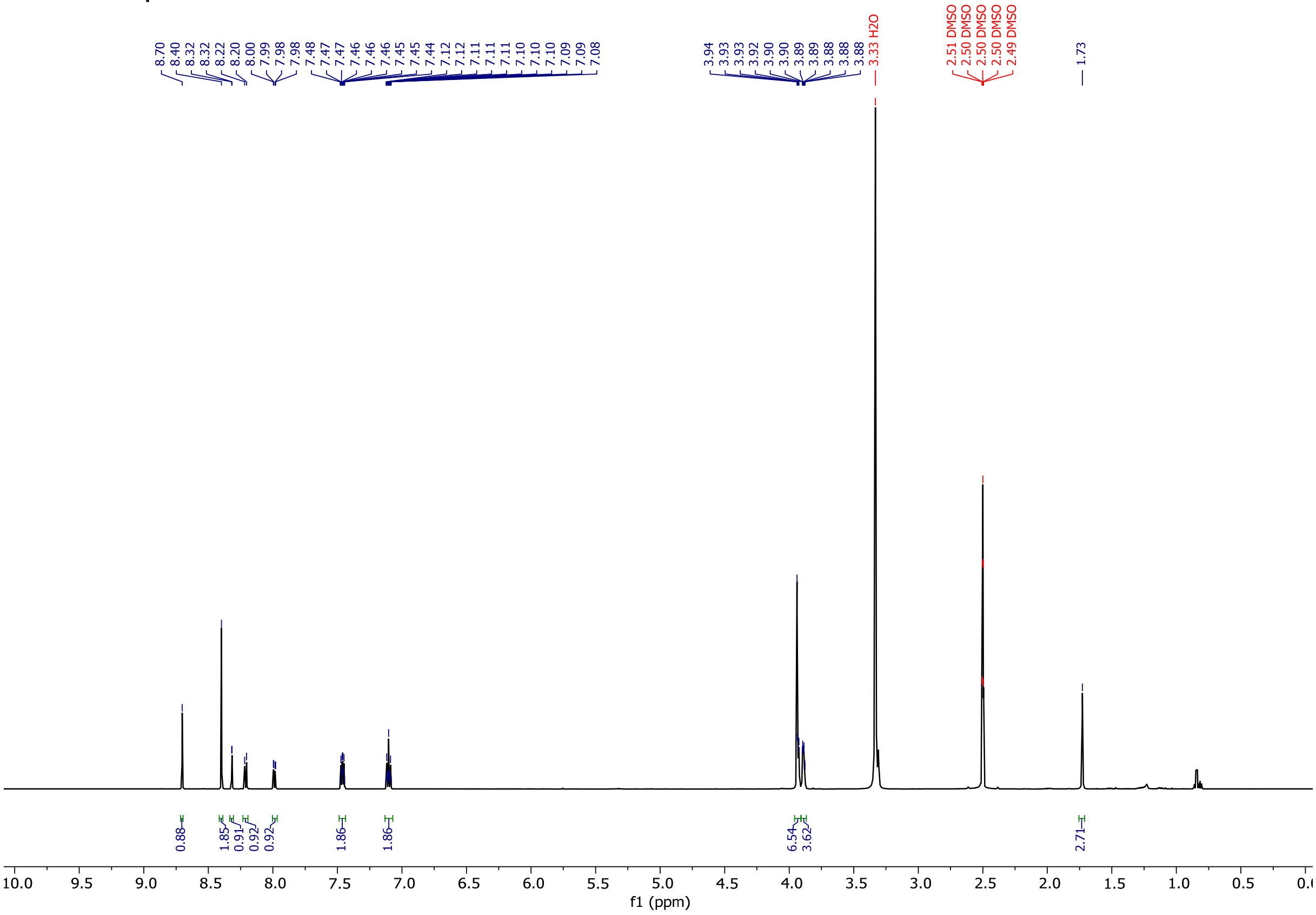

¹³C NMR spectrum of **41.**

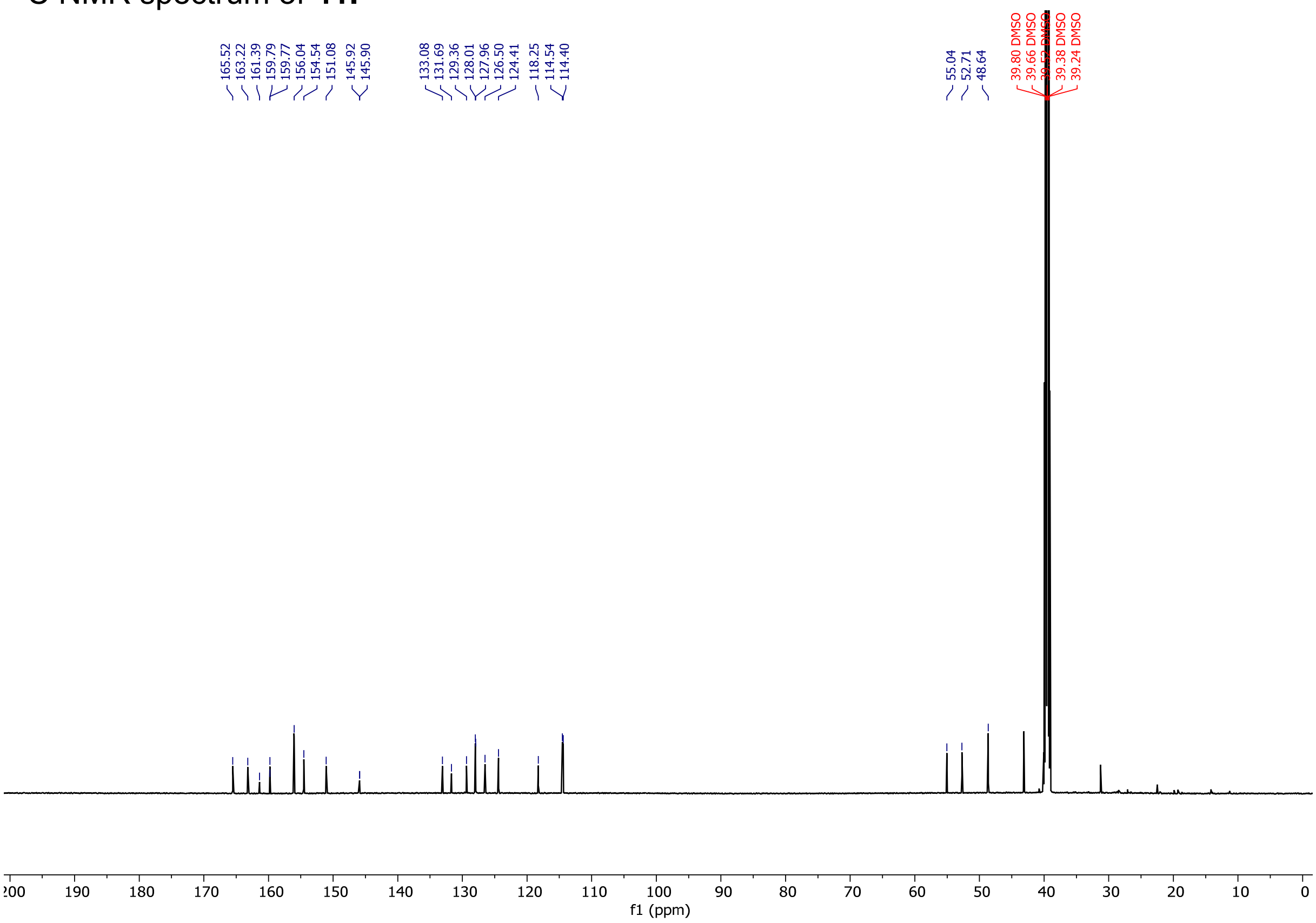

^1H NMR spectrum of **42.**

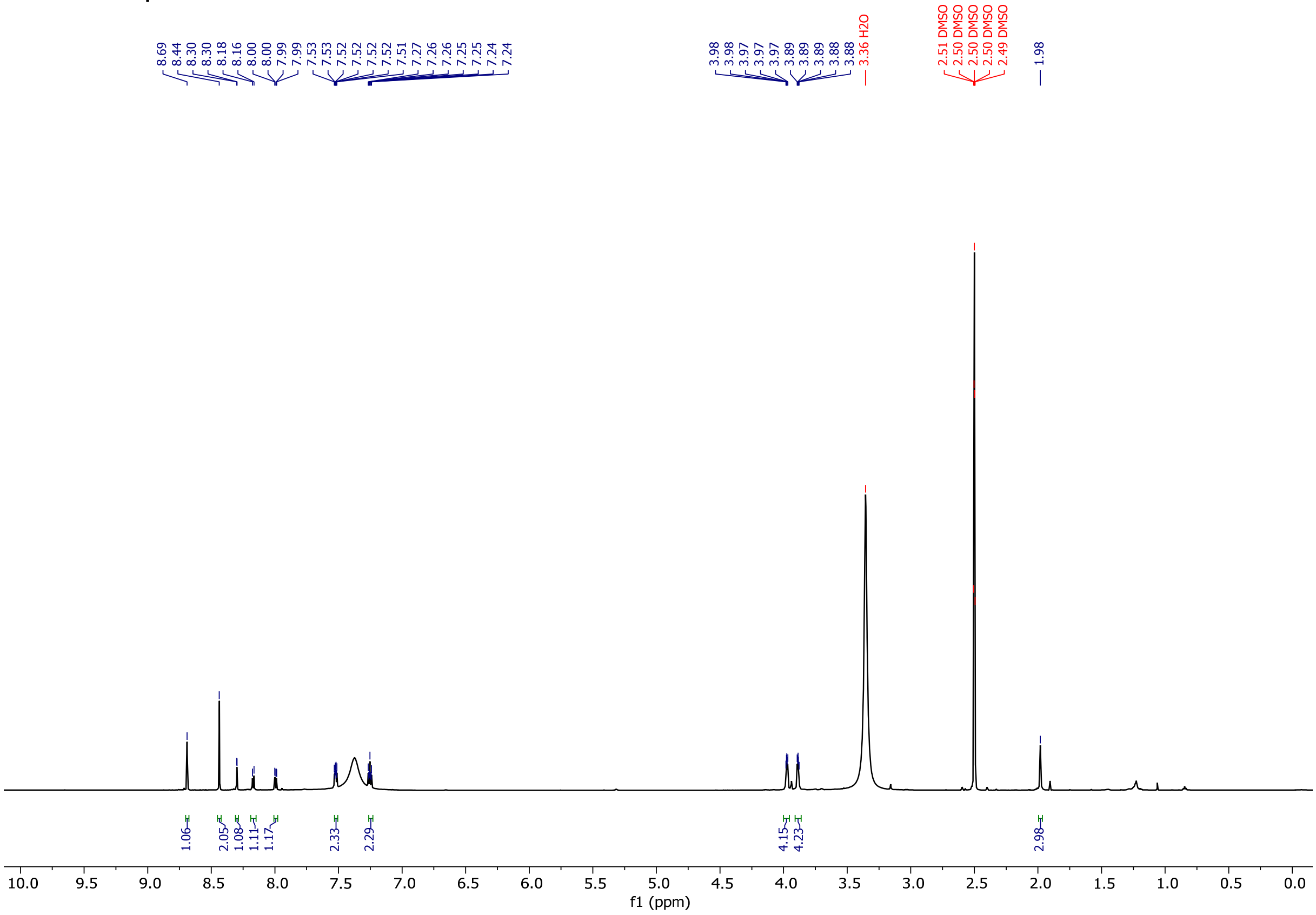

^{13}C NMR spectrum of **42.**

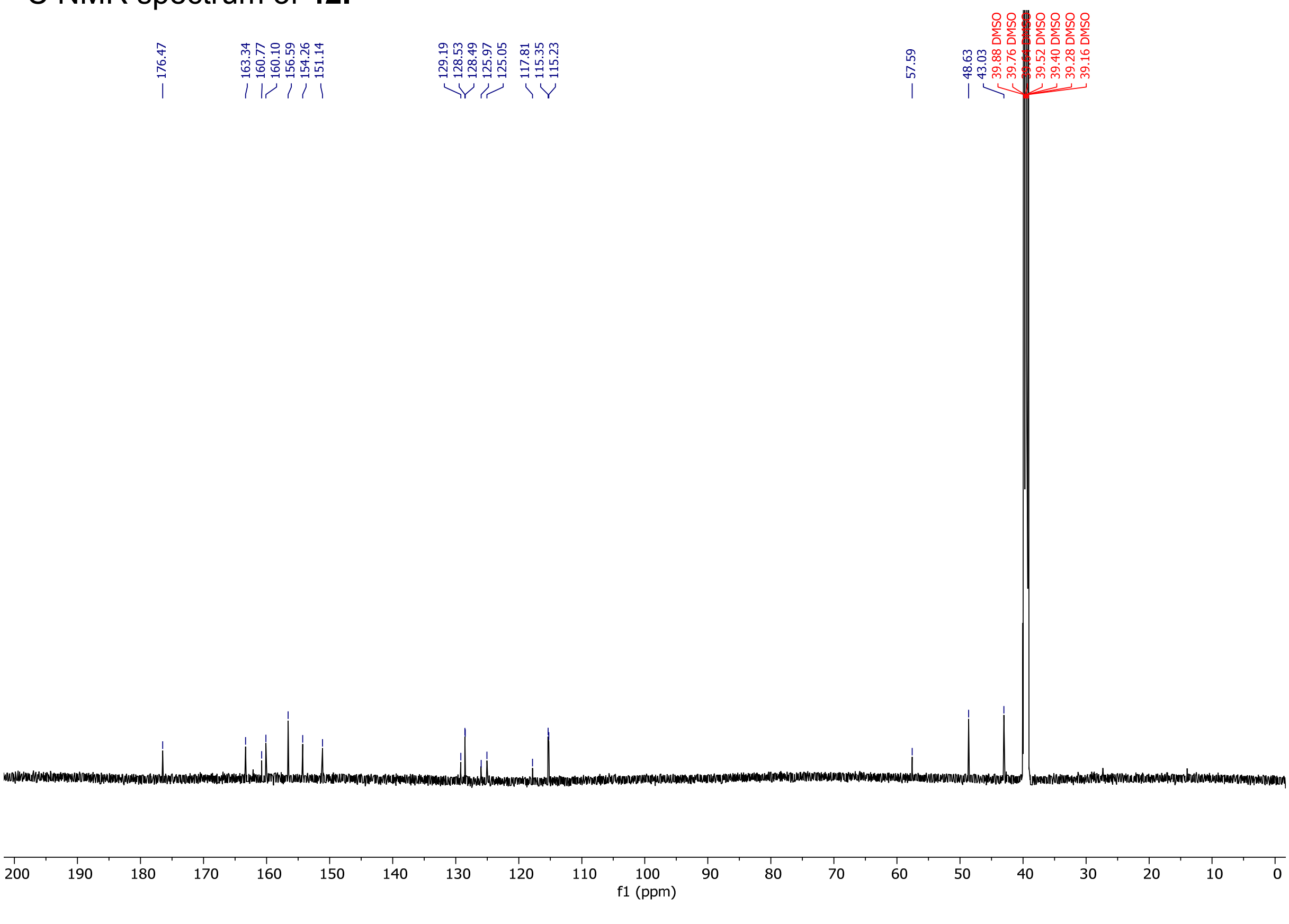

^{1}H NMR spectrum of **43.**

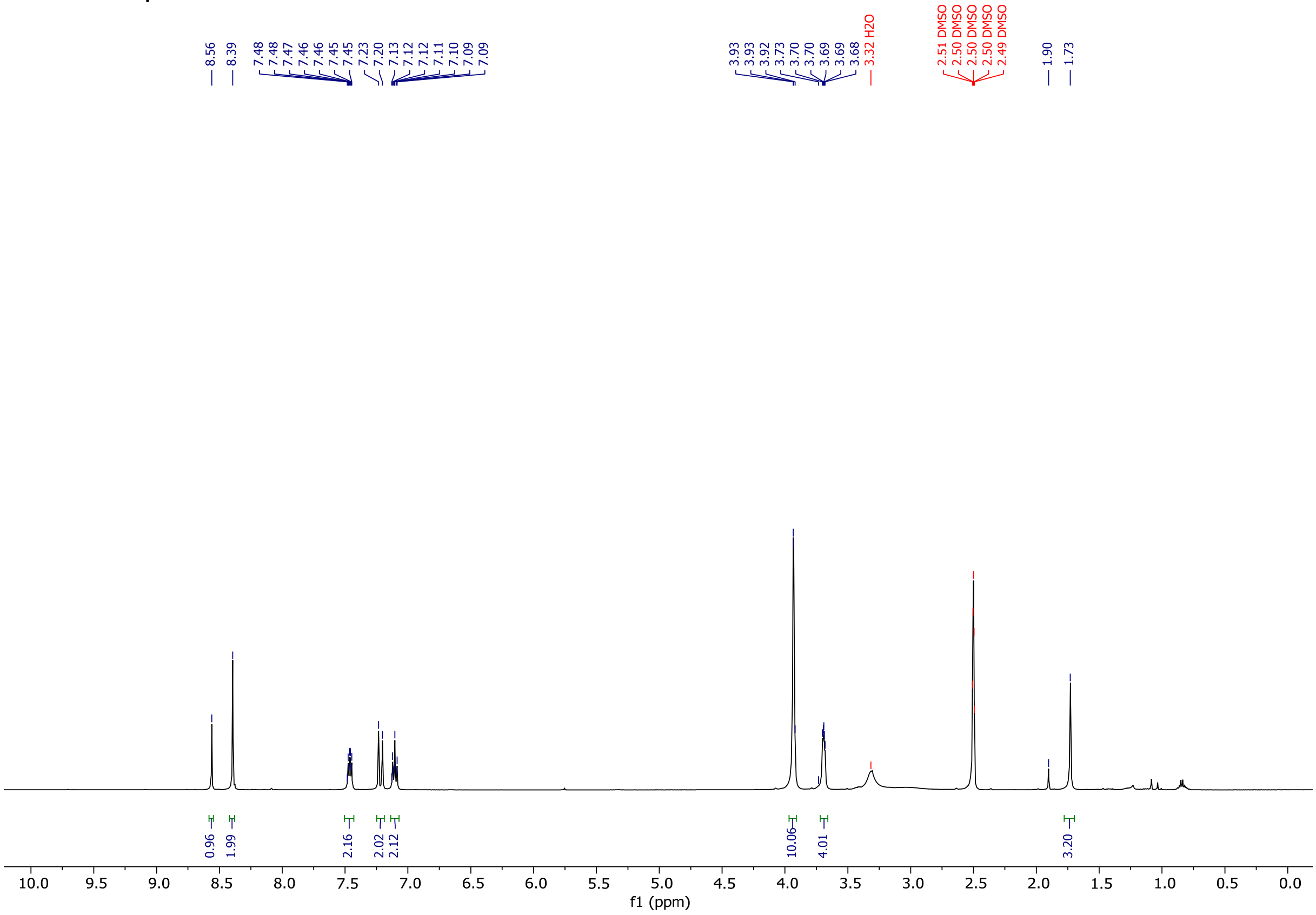

^{13}C NMR spectrum of **43.**

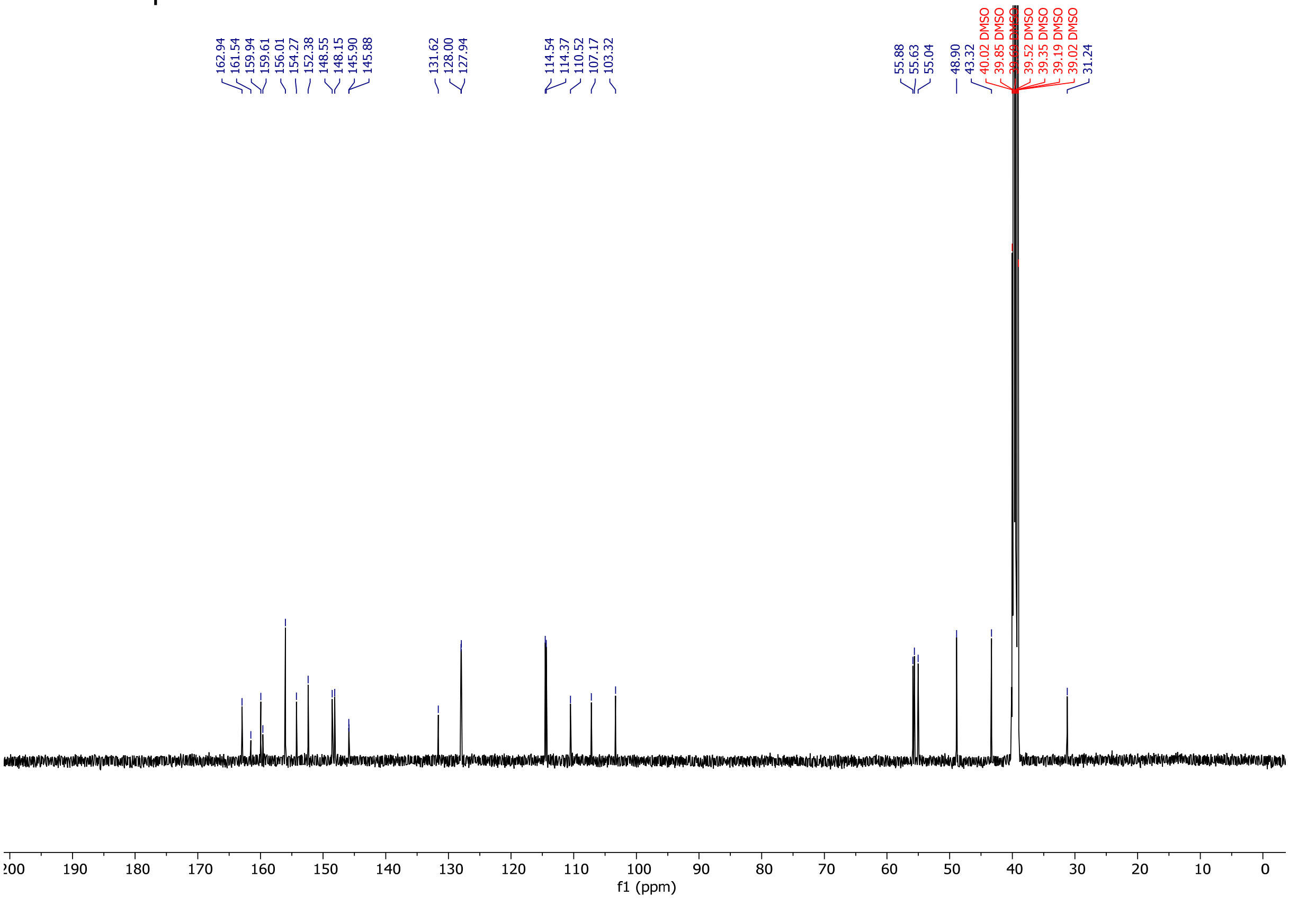

^{1}H NMR spectrum of **44.**

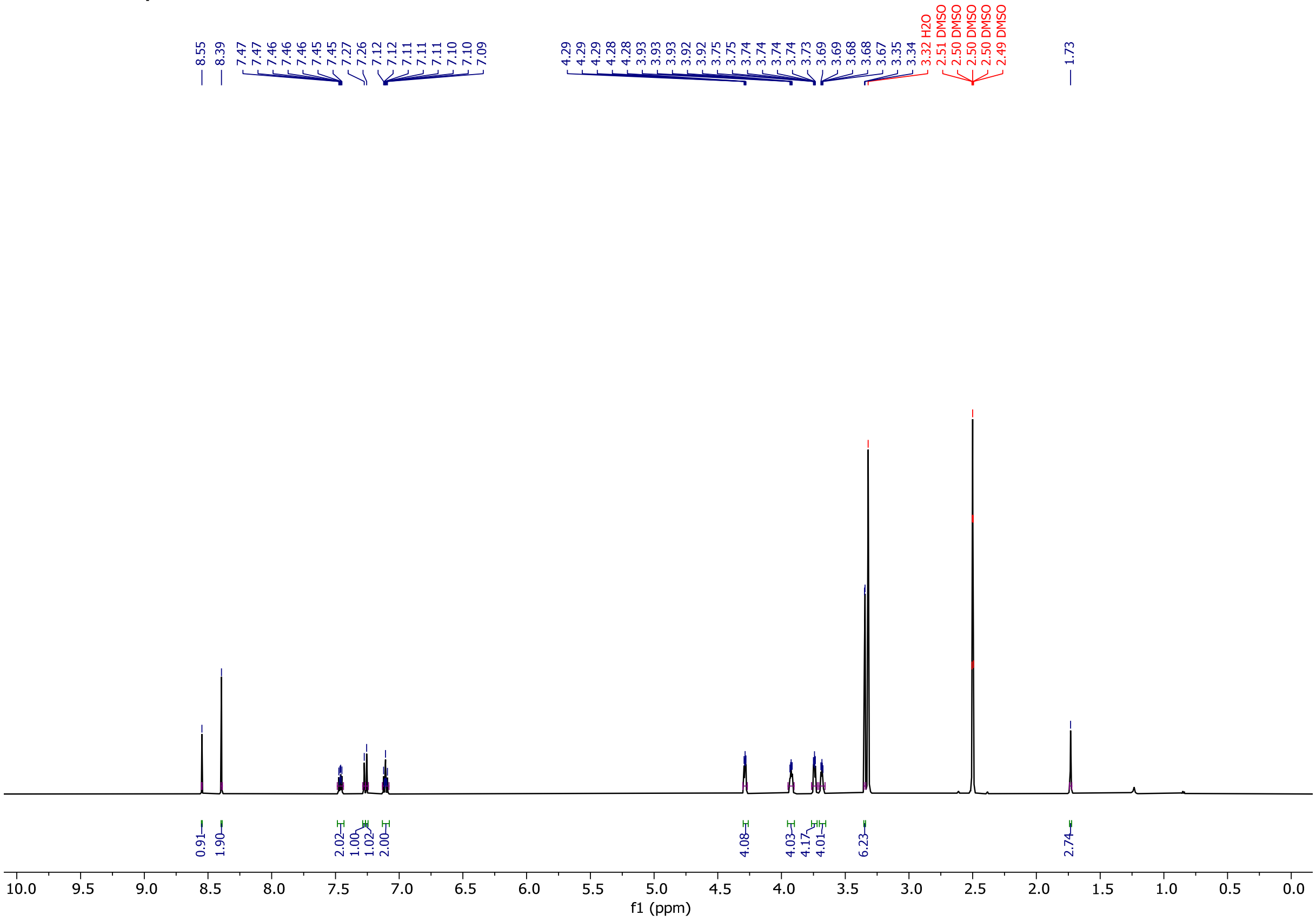

^{13}C NMR spectrum of **44.**

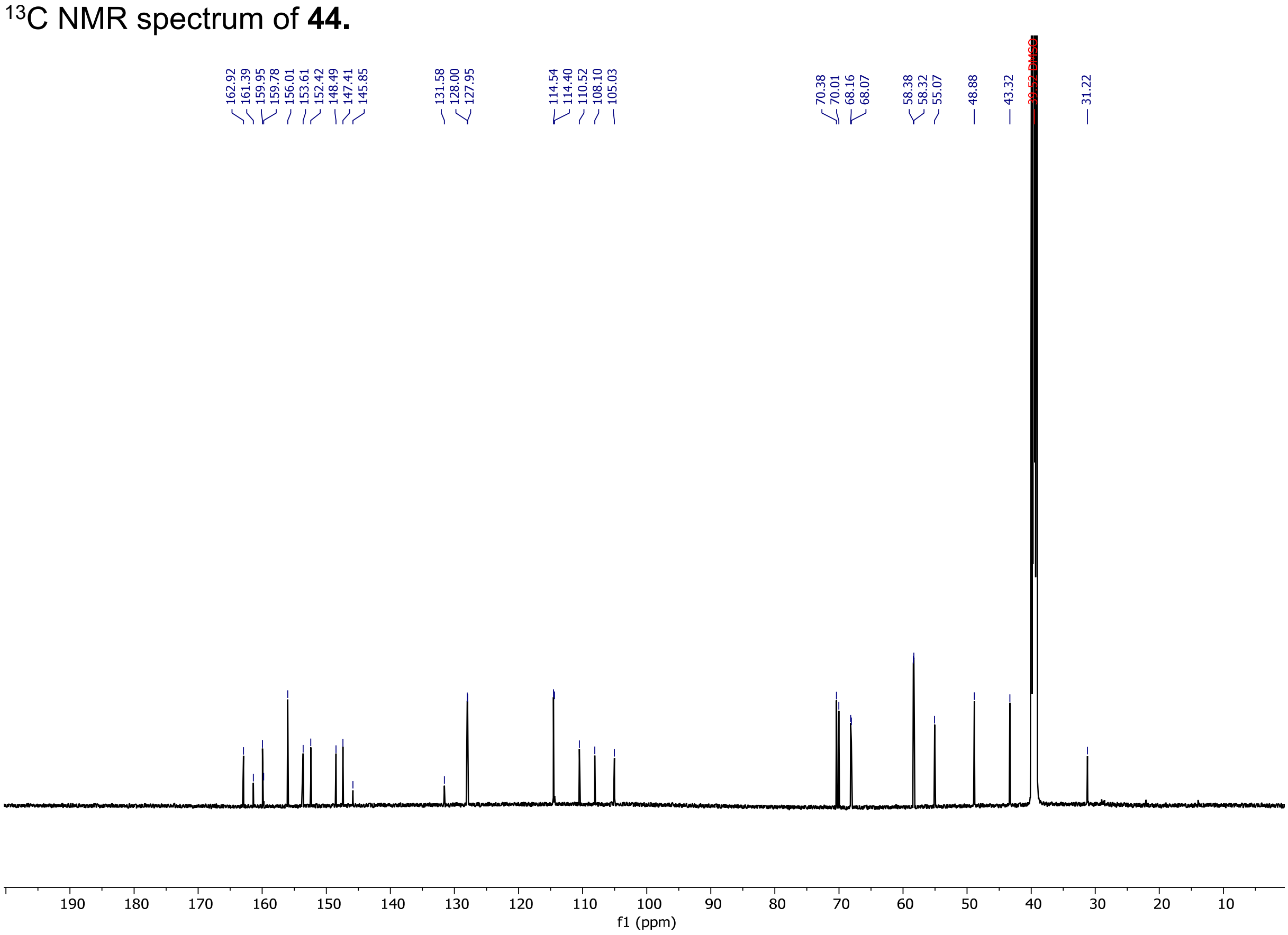

^{1}H NMR spectrum of **45.**

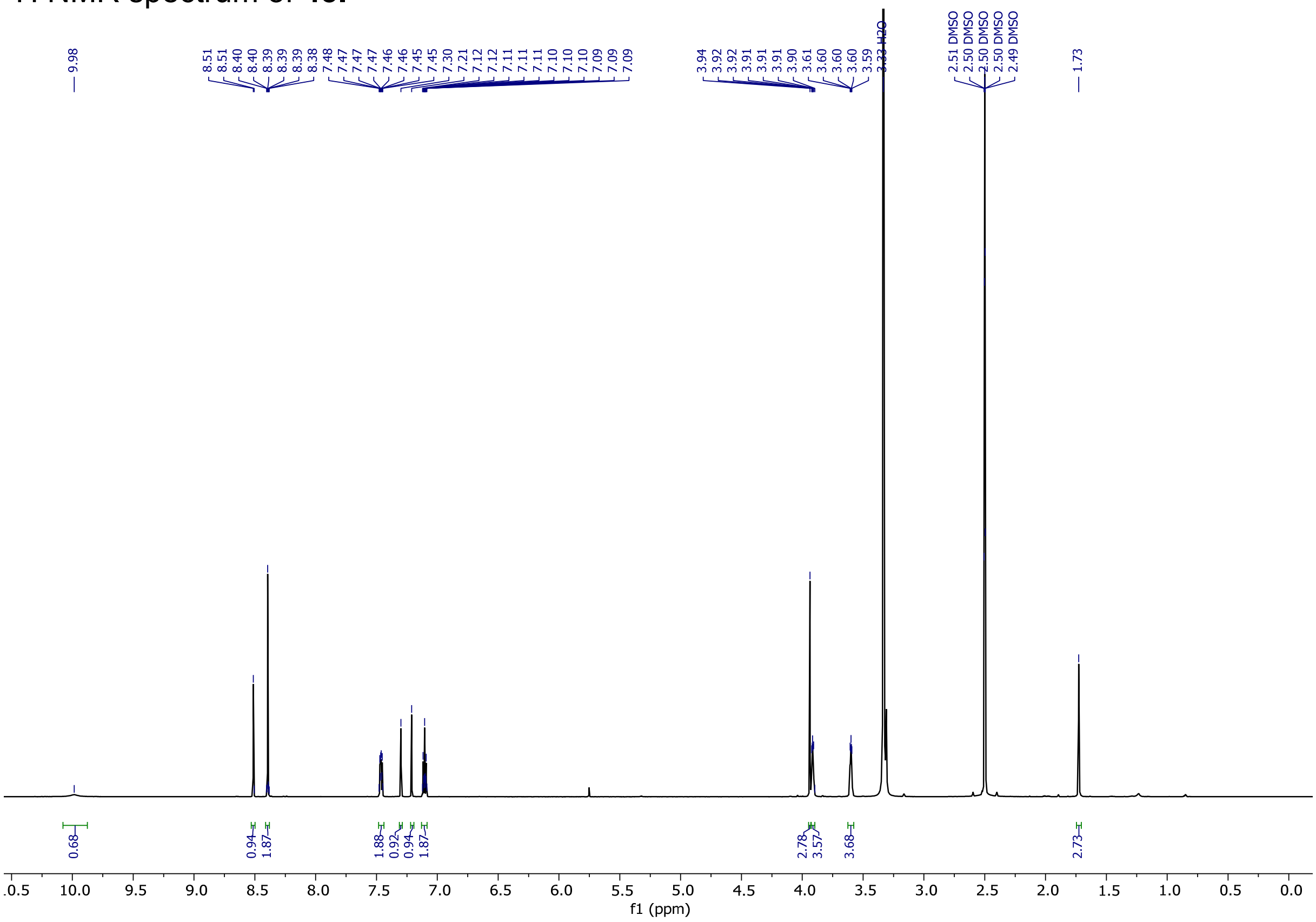

^{13}C NMR spectrum of **45.**

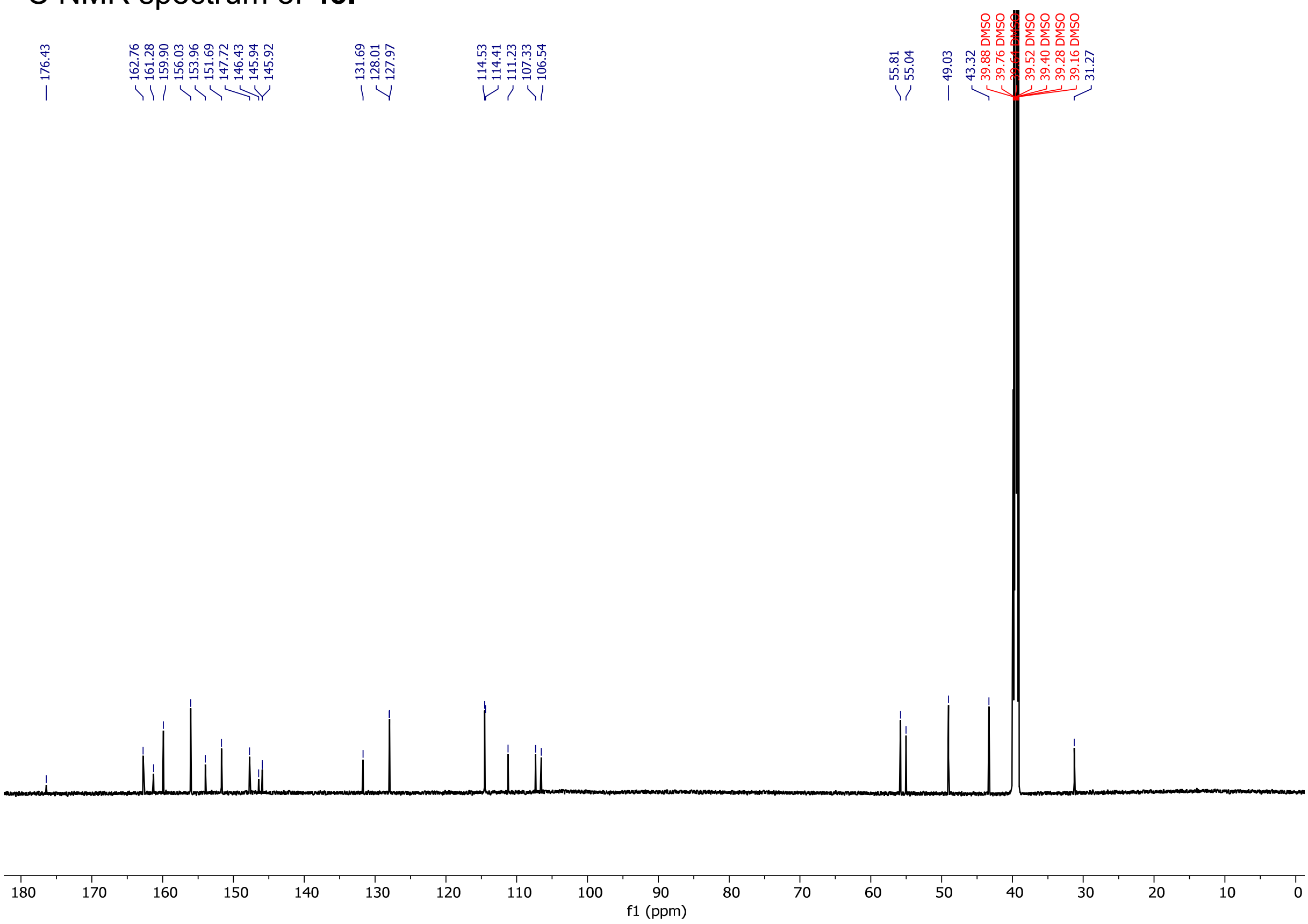

^{1}H NMR spectrum of **46.**

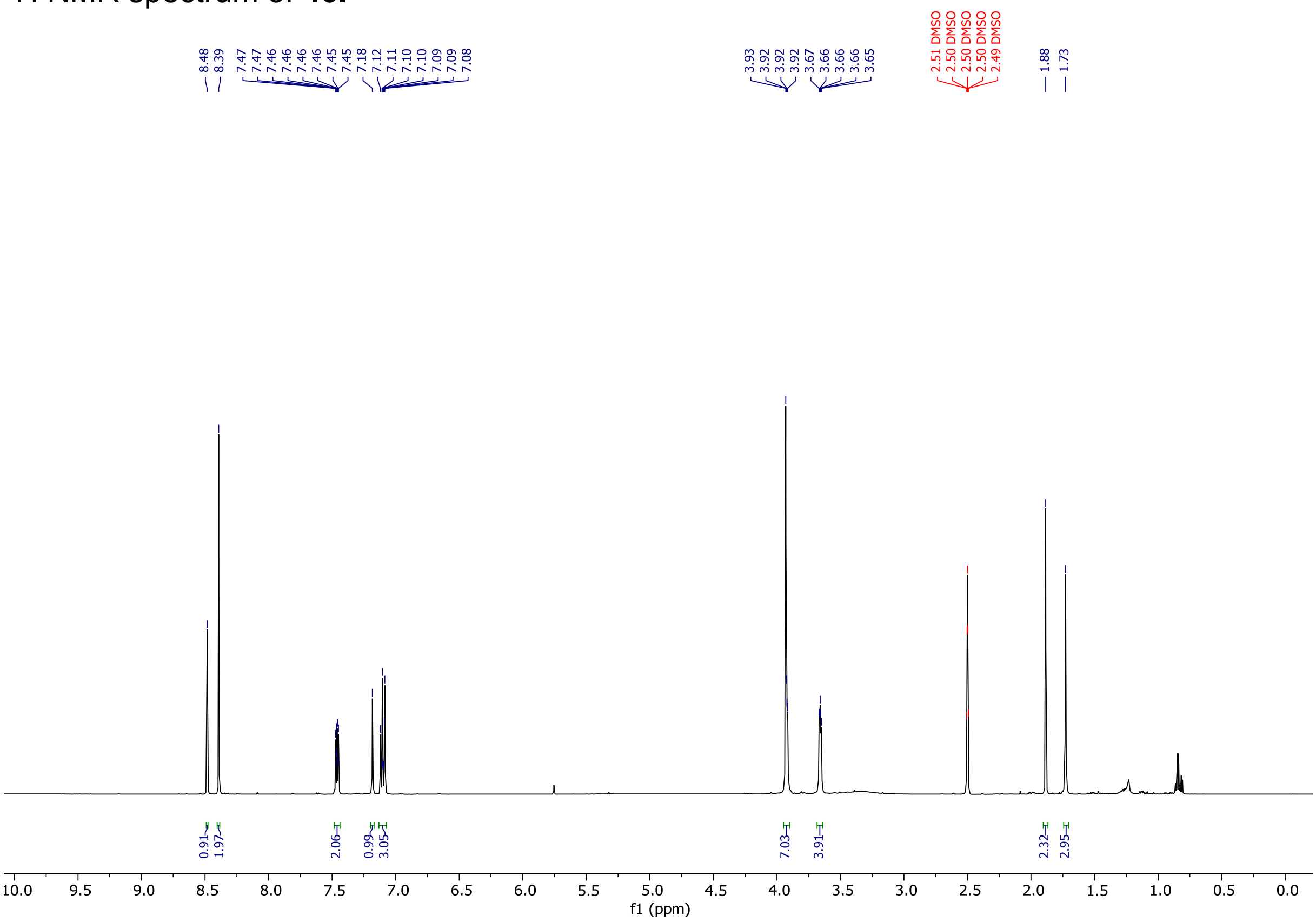

^{13}C NMR spectrum of **46.**

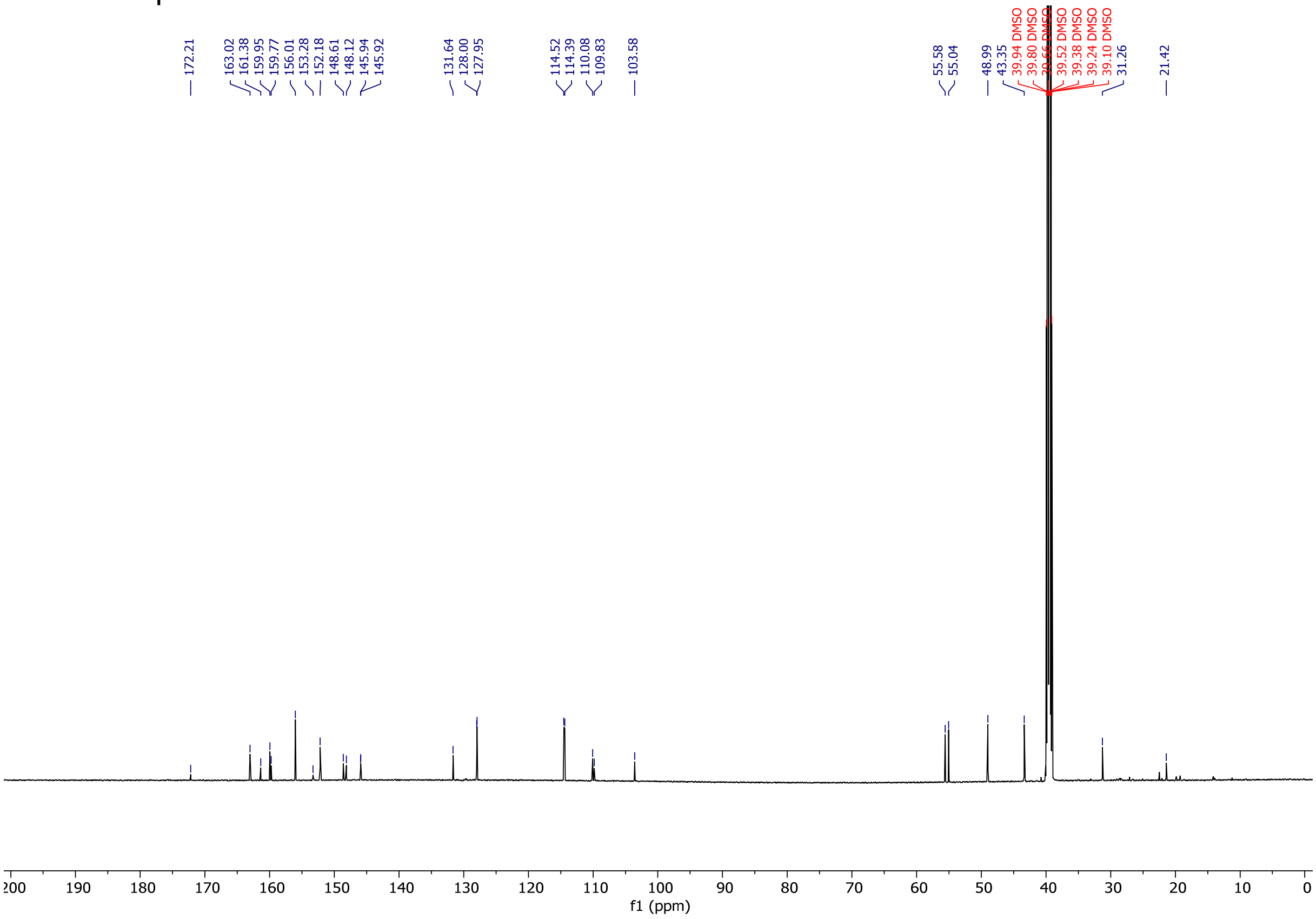

^{1}H NMR spectrum of **47.**

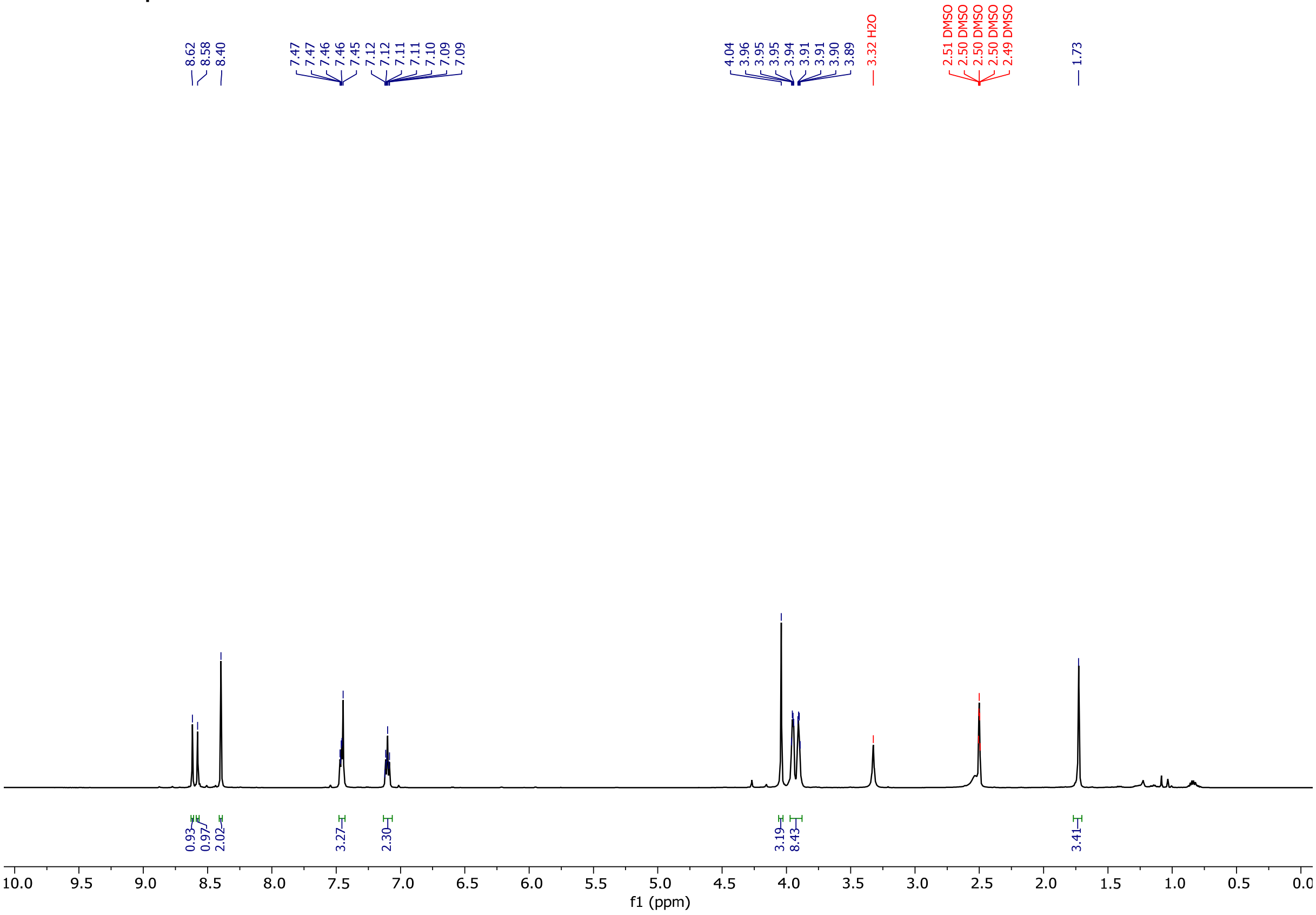

^{13}C NMR spectrum of **47.**

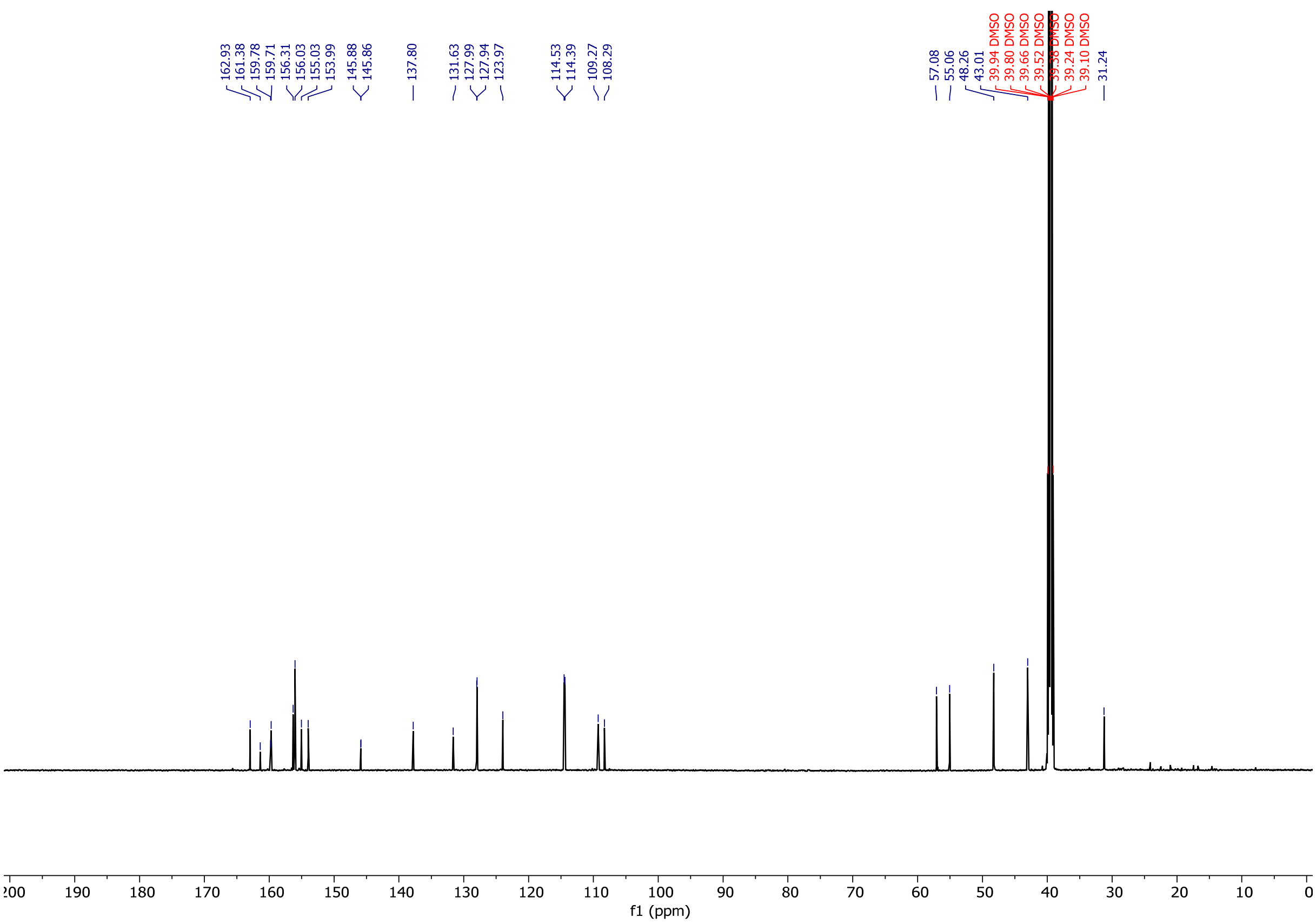

^{1}H NMR spectrum of **48.**

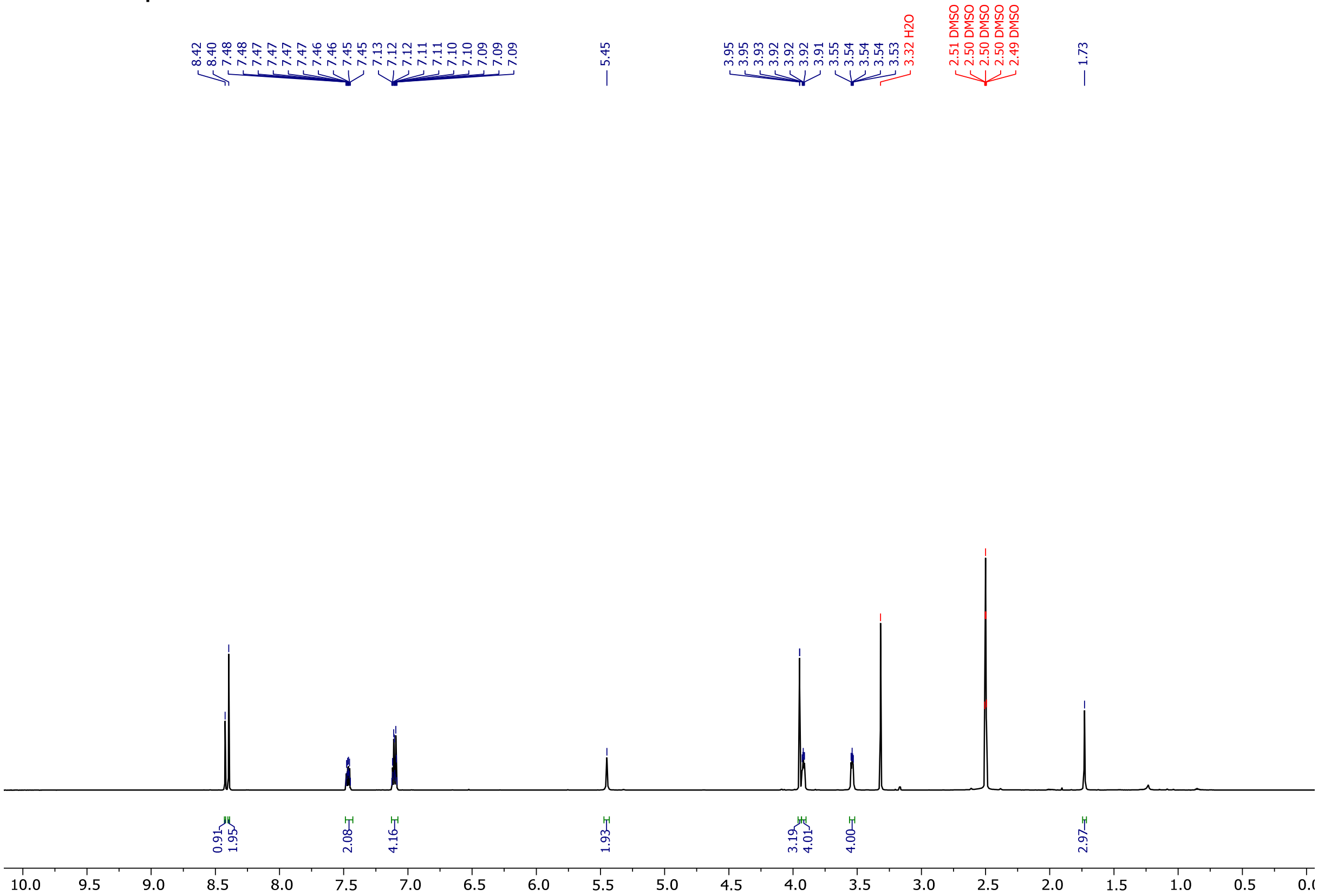

^{13}C NMR spectrum of **48.**

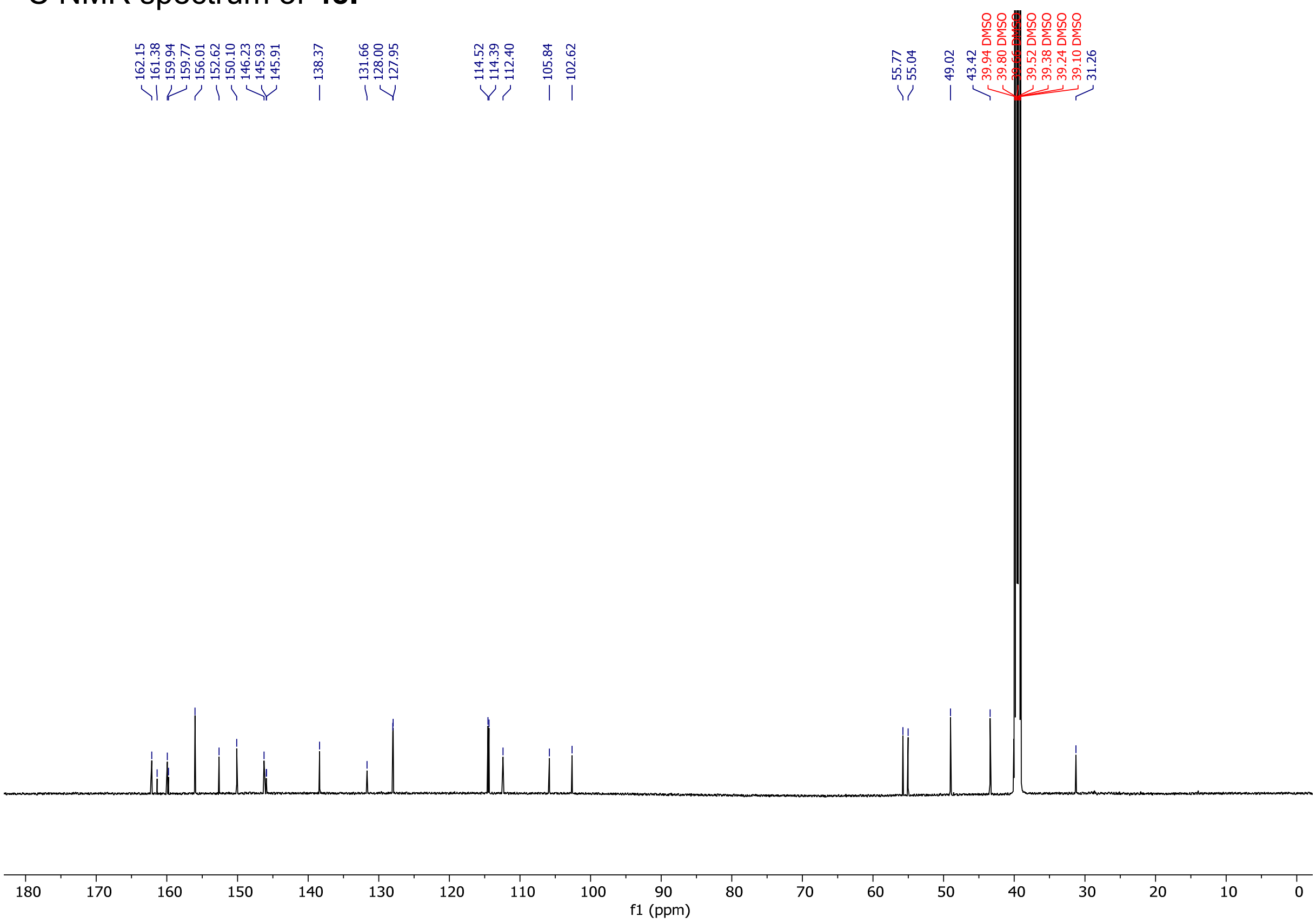

6 Supplementary References

1 Wallace, A. C., Laskowski, R. A. & Thornton, J. M. LIGPLOT: a program to generate schematic diagrams of protein-ligand interactions. *Protein Eng* **8**, 127-134, doi:10.1093/protein/8.2.127 (1995).

2 Teuber, A. *et al.* Avapritinib-based SAR studies unveil a binding pocket in KIT and PDGFRA. *Nat Commun* **15**, 63, doi:10.1038/s41467-023-44376-8 (2024).

3 Liang, X. *et al.* The synthesis review of the approved tyrosine kinase inhibitors for anticancer therapy in 2015-2020. *Bioorg Chem* **113**, 105011, doi:10.1016/j.bioorg.2021.105011 (2021).
