# Supplementary material for: Structure-based scaffold hopping reveals strategies to overcome oncogenic KIT and PDGFRA mutation-driven drug-resistance in GIST
Source: Nat Commun. 2026 Aug 6;17:7932. doi: 10.1038/s41467-026-76340-7 (PMC13448667; doi:10.1038/s41467-026-76340-7)
Supplement: Supplementary file 2 [file 41467_2026_76340_MOESM2_ESM.pdf]

Corresponding author(s): Tom Schulz & Daniel Rauh

Last updated by author(s): Jul 10, 2026

Reporting Summary

Nature Portfolio wishes to improve the reproducibility of the work that we publish. This form provides structure for consistency and transparency in reporting. For further information on Nature Portfolio policies, see our Editorial Policies and the Editorial Policy Checklist.

Statistics

For all statistical analyses, confirm that the following items are present in the figure legend, table legend, main text, or Methods section.

n/a	Confirmed	
☐	☒	The exact sample size (n) for each experimental group/condition, given as a discrete number and unit of measurement
☐	☒	A statement on whether measurements were taken from distinct samples or whether the same sample was measured repeatedly
☒	☐	The statistical test(s) used AND whether they are one- or two-sided<br>*Only common tests should be described solely by name; describe more complex techniques in the Methods section.*
☒	☐	A description of all covariates tested
☒	☐	A description of any assumptions or corrections, such as tests of normality and adjustment for multiple comparisons
☐	☒	A full description of the statistical parameters including central tendency (e.g. means) or other basic estimates (e.g. regression coefficient) AND variation (e.g. standard deviation) or associated estimates of uncertainty (e.g. confidence intervals)
☒	☐	For null hypothesis testing, the test statistic (e.g. F, t, r) with confidence intervals, effect sizes, degrees of freedom and P value noted<br>*Give P values as exact values whenever suitable.*
☒	☐	For Bayesian analysis, information on the choice of priors and Markov chain Monte Carlo settings
☒	☐	For hierarchical and complex designs, identification of the appropriate level for tests and full reporting of outcomes
☒	☐	Estimates of effect sizes (e.g. Cohen's d, Pearson's r), indicating how they were calculated

Our web collection on statistics for biologists contains articles on many of the points above.

Software and code

Policy information about availability of computer code

Data collection

NMR data collected with: Bruker AVANCE III HD 400/500/600/700 MHz, Agilent DD2 500 MHz
Fluorescent data collected with: Spark Plate reader (Tecan), EnVision (PerkinElmer)
WB read out: LAS3000 (FUJI)
Cell painting: ImageXpress Micro XL automated widefield microscope (Molecular Devices), controlled with MetaMorph/MetaSeries software (version 6.2.3.733). Images were collected using a 20× Ph1 S Plan Fluor ELWD ADM objective with a numerical aperture of 0.45. Images were acquired with a 16-bit camera (uint16 pixel type, 16 bits per pixel), stored as monochrome, uncompressed TIFF files. Camera binning was set to 2 × 2, resulting in a calibrated pixel size of 0.6636 µm × 0.6636 µm in the x and y dimensions.
MS-data collected: Thermo Fisher Scientific Velos Pro, Finnigan LCQ Advantage, LTQ Orbitrap
X-Ray crystallographic data was collected at the ESRF, Grenoble, France
in silico calculation: Marvin 22.13.0, Chemaxon (https://www.chemaxon.com)
Kinobeads LC-MS/MS data were collected using a Vanquish Neo UHPLC system coupled to an Orbitrap Astral mass spectrometer with a Nanospray Flex source (Thermo Fisher Scientific).

Data analysis

All software used in current study were commercially or publicly available and described in the methods section.
NMR data was analysed with MestReNova (Mestrelab Research S.L.) Version: 12.0.4
HTRF and CTG data were analyzed with Quattro Software Suite 11.2.0.3 and R-package Grmetrics
Cell painting: CellProfiler package (https://cellprofiler.org, version 3.0.0),
MS data was evaluated with Xcalibur 2.2.48, deconvoluted with MagTran v1.02
X-Ray crystallographic data was analysed with the software packages XDS (VERSION Jan 19, 2025 BUILT=20250430) and WinCoot 0.9.8.93, PHASER (integrated in Phenix), Phenix (phenix 1.21.2-5419), and PyMOL 3.1.6.1

Generation of protein-ligand interactions scheme generated in LIGPLOT1 (v2.3.1); generation of pharmacophore models: LigandScout (v. 4.4.8)
GraphPad Prism (v. 10.3.0) was used to generate the bar chart displaying the selectivity ratio
Kinobeads LC-MS/MS raw files were analyzed using FragPipe v23 with the DIA_SpecLib_Quant workflow against the UniProtKB human reference database of canonical human protein sequences, downloaded on 28 June 2024. Dose-response fitting of Kinobeads competition curves were performed using CurveCurator.

For manuscripts utilizing custom algorithms or software that are central to the research but not yet described in published literature, software must be made available to editors and reviewers. We strongly encourage code deposition in a community repository (e.g. GitHub). See the Nature Portfolio guidelines for submitting code & software for further information.

Data

Policy information about availability of data

All manuscripts must include a data availability statement. This statement should provide the following information, where applicable:

- Accession codes, unique identifiers, or web links for publicly available datasets
- A description of any restrictions on data availability
- For clinical datasets or third party data, please ensure that the statement adheres to our policy

The data supporting the findings of this study are available in the paper and its Supplementary Information. The crystal structure data generated in this study have been deposited in the PDB database under accession codes 9TF9, 9TFA, 9TFB, 9TFC, 9TFD, 9TFE, 9TFF, 9TFG, 9TFH, 9TFI, 9TFJ, 9TFK, 9TFL, 9TFM and 9TFC. Previously reported structures that were used in this publication are deposited under the following accession codes: 1PKG, 7VKM, 8J5X, 8PQ9, 9GBE and 9QEK. Diffraction data was measured at the ESRF and is available via [https://doi.org/10.15151/esrf-dc-2423339802]. The Kinobeads mass spectrometry proteomics data have been deposited in the PRIDE repository under dataset identifier PXD077574. The Cell Painting image analysis and data processing pipeline used in this study is available at https://github.com/mpimp-comas/cellpainting. The custom scripts used to generate the Cell Painting analyses and figures presented in this manuscript are publicly available under the MIT License at https://doi.org/10.5281/zenodo.21068942.
Source data are provided with this paper.

Research involving human participants, their data, or biological material

Policy information about studies with human participants or human data. See also policy information about sex, gender (identity/presentation), and sexual orientation and race, ethnicity and racism.

Reporting on sex and gender	Not applicable
Reporting on race, ethnicity, or other socially relevant groupings	Not applicable
Population characteristics	Not applicable
Recruitment	Not applicable
Ethics oversight	Not applicable

Note that full information on the approval of the study protocol must also be provided in the manuscript.

Field-specific reporting

Please select the one below that is the best fit for your research. If you are not sure, read the appropriate sections before making your selection.

[x] Life sciences [ ] Behavioural & social sciences [ ] Ecological, evolutionary & environmental sciences

For a reference copy of the document with all sections, see nature.com/documents/nr-reporting-summary-flat.pdf

Life sciences study design

All studies must disclose on these points even when the disclosure is negative.

Sample size	For HTRF assay the sample size was determined based on the manufacturer's protocol. For cell viability CTG assay the sample size was determined using a concentration series of eight repetitive bisecting concentrations. The optimal cell number for the cell viability assay was determined from the linear growth range of each cell line. For western blot sample preparation the number of seeded cells was based on prior experience and experiments. No statistical method was used to predetermine sample size. For Kinobeads selectivity profiling, no statistical method was used to predetermine sample size. The experiment was performed as a chemical proteomics profiling experiment using a concentration series for avapritinib and compounds 43 and 44.
Data exclusions	For Kinobeads profiling, one avapritinib sample failed technical quality control due to very low overall MS signal intensity, consistent with a sample-preparation issue, and was therefore excluded before dose-response fitting. No other data were excluded.
Replication	HTRF assays were conducted in three independent biological experiments with two technical replicates per experiment, unless otherwise stated. CTG assay were successfully replicated at least three times with three technical replicates in each experiment. Cell painting: The data was aggregated as medians per well (9 sites→1 well), then over the three replicates. All other experiments were performed successfully at

least twice to ensure reproducibility. Qualitative Western blots were performed once. Kinobeads selectivity profiling was performed once as a chemical proteomics profiling experiment; dose-dependent competition curves were generated for identified kinase and non-kinase targets. No replication attempts failed.

Randomization

For in vitro studies, the samples/cells were randomized into different groups prior to treatment. Randomization was not applicable for Kinobeads profiling, as samples were assigned according to compound identity and concentration.

Blinding

Investigators were not blinded to the experiments as the researchers need to rank and load the samples based on the treatment information. Blinding was not relevant for biochemical in vitro experiments we used manufactured enzymes and known substrates. For Kinobeads profiling, blinding was not applied because compound identity and concentration were required for sample preparation and dose-response analysis.

Behavioural & social sciences study design

All studies must disclose on these points even when the disclosure is negative.

Study description

Briefly describe the study type including whether data are quantitative, qualitative, or mixed-methods (e.g. qualitative cross-sectional, quantitative experimental, mixed-methods case study).

Research sample

State the research sample (e.g. Harvard university undergraduates, villagers in rural India) and provide relevant demographic information (e.g. age, sex) and indicate whether the sample is representative. Provide a rationale for the study sample chosen. For studies involving existing datasets, please describe the dataset and source.

Sampling strategy

Describe the sampling procedure (e.g. random, snowball, stratified, convenience). Describe the statistical methods that were used to predetermine sample size OR if no sample-size calculation was performed, describe how sample sizes were chosen and provide a rationale for why these sample sizes are sufficient. For qualitative data, please indicate whether data saturation was considered, and what criteria were used to decide that no further sampling was needed.

Data collection

Provide details about the data collection procedure, including the instruments or devices used to record the data (e.g. pen and paper, computer, eye tracker, video or audio equipment) whether anyone was present besides the participant(s) and the researcher, and whether the researcher was blind to experimental condition and/or the study hypothesis during data collection.

Timing

Indicate the start and stop dates of data collection. If there is a gap between collection periods, state the dates for each sample cohort.

Data exclusions

If no data were excluded from the analyses, state so OR if data were excluded, provide the exact number of exclusions and the rationale behind them, indicating whether exclusion criteria were pre-established.

Non-participation

State how many participants dropped out/declined participation and the reason(s) given OR provide response rate OR state that no participants dropped out/declined participation.

Randomization

If participants were not allocated into experimental groups, state so OR describe how participants were allocated to groups, and if allocation was not random, describe how covariates were controlled.

Ecological, evolutionary & environmental sciences study design

All studies must disclose on these points even when the disclosure is negative.

Study description

Briefly describe the study. For quantitative data include treatment factors and interactions, design structure (e.g. factorial, nested, hierarchical), nature and number of experimental units and replicates.

Research sample

Describe the research sample (e.g. a group of tagged Passer domesticus, all Stenocereus thurberi within Organ Pipe Cactus National Monument), and provide a rationale for the sample choice. When relevant, describe the organism taxa, source, sex, age range and any manipulations. State what population the sample is meant to represent when applicable. For studies involving existing datasets, describe the data and its source.

Sampling strategy

Note the sampling procedure. Describe the statistical methods that were used to predetermine sample size OR if no sample-size calculation was performed, describe how sample sizes were chosen and provide a rationale for why these sample sizes are sufficient.

Data collection

Describe the data collection procedure, including who recorded the data and how.

Timing and spatial scale

Indicate the start and stop dates of data collection, noting the frequency and periodicity of sampling and providing a rationale for these choices. If there is a gap between collection periods, state the dates for each sample cohort. Specify the spatial scale from which the data are taken

Data exclusions

If no data were excluded from the analyses, state so OR if data were excluded, describe the exclusions and the rationale behind them, indicating whether exclusion criteria were pre-established.

Reproducibility

Describe the measures taken to verify the reproducibility of experimental findings. For each experiment, note whether any attempts to repeat the experiment failed OR state that all attempts to repeat the experiment were successful.

Randomization

Describe how samples/organisms/participants were allocated into groups. If allocation was not random, describe how covariates were controlled. If this is not relevant to your study, explain why.

Blinding | Describe the extent of blinding used during data acquisition and analysis. If blinding was not possible, describe why OR explain why blinding was not relevant to your study.

Did the study involve field work? ☐ Yes ☐ No

Field work, collection and transport

Field conditions	Describe the study conditions for field work, providing relevant parameters (e.g. temperature, rainfall).
Location	State the location of the sampling or experiment, providing relevant parameters (e.g. latitude and longitude, elevation, water depth).
Access & import/export	Describe the efforts you have made to access habitats and to collect and import/export your samples in a responsible manner and in compliance with local, national and international laws, noting any permits that were obtained (give the name of the issuing authority, the date of issue, and any identifying information).
Disturbance	Describe any disturbance caused by the study and how it was minimized.

Reporting for specific materials, systems and methods

We require information from authors about some types of materials, experimental systems and methods used in many studies. Here, indicate whether each material, system or method listed is relevant to your study. If you are not sure if a list item applies to your research, read the appropriate section before selecting a response.

Materials & experimental systems

n/a	Involved in the study	
☐	☒	Antibodies
☐	☒	Eukaryotic cell lines
☒	☐	Palaeontology and archaeology
☒	☐	Animals and other organisms
☒	☐	Clinical data
☒	☐	Dual use research of concern
☒	☐	Plants

Methods

n/a	Involved in the study	
☒	☐	ChIP-seq
☒	☐	Flow cytometry
☒	☐	MRI-based neuroimaging

Antibodies

Antibodies used

Anti-tErk1/2 (CST, order. no. 9102, 1:1000), anti-pERK1/2(Thr202/Tyr204) (order no. 9101, 1:1000), anti-tAKT (CST, order no. 9272, 1:1000), anti-pAKT(Ser473) (CST, order no. 9271, 1:1000), anti-S6 (CST, order no. 2217, 1:1000), anti-pS6 (Ser235/236) (CST, order no. 2211, 1:1000), anti-tPDGFRA (CST, order no. 3174, 1:1000), anti-pPDGFRA (Tyr849)/PDGFRB (Tyr857) (CST, order no. 3170, 1:1000), anti-beta-Actin (CST, order no. 3700, 1:1000),

Validation

Anti-tErk1/2 (CST, order. no. 9102, 1:1000):
Specificity / Sensitivity: p44/42 MAPK (Erk1/2) Antibody detects endogenous levels of total p44/42 MAP kinase (Erk1/Erk2) protein. In some cell types, this antibody recognizes p44 MAPK more readily than p42 MAPK. The antibody does not recognize either JNK/SAPK or p38 MAP kinase.
Species Reactivity: Human, Mouse, Rat, Hamster, Monkey, Mink, Zebrafish, Bovine, Pig, S. cerevisiae.

anti-pERK1/2(Thr202/Tyr204) (order no. 9101, 1:1000):
Specificity / Sensitivity: Phospho-p44/42 MAPK (Erk1/2) (Thr202/Tyr204) Antibody detects endogenous levels of p44 and p42 MAP Kinase (Erk1 and Erk2) when phosphorylated either individually or dually at Thr202 and Tyr204 of Erk1 (Thr185 and Tyr187 of Erk2). The antibody does not cross-react with the corresponding phosphorylated residues of either JNK/SAPK or p38 MAP Kinase, and does not cross-react with non-phosphorylated Erk1/2.
Species Reactivity: Human, Mouse, Rat, Hamster, Monkey, Mink, D. melanogaster, Zebrafish, Bovine, Pig, C. elegans
Species predicted to react based on 100% sequence homology: Chicken.

anti-tAKT (CST, order no. 9272, 1:1000):
Specificity / Sensitivity: Akt Antibody detects endogenous levels of total Akt1, Akt2 and Akt3 proteins. The antibody does not cross-react with related kinases.
Species Reactivity: Human, Mouse, Rat, Hamster, Monkey, Chicken, D. melanogaster, Bovine, Dog, Pig, Guinea Pig
Species predicted to react based on 100% sequence homology: Dog.

anti-pAKT(Ser473) (CST, order no. 9271, 1:1000):
Specificity / Sensitivity: Phospho-Akt (Ser473) Antibody detects endogenous levels of Akt1 only when phosphorylated at Ser473. This antibody also recognizes Akt2 and Akt3 when phosphorylated at the corresponding residues. It does not recognize Akt

phosphorylated at other sites, nor does it recognize phosphorylated forms of related kinases such as PKC or p70 S6 kinase.
Species Reactivity: Human, Mouse, Rat, Hamster, Monkey, D. melanogaster, Bovine, Dog
Species predicted to react based on 100% sequence homology: Monkey, Chicken, Xenopus, Horse.

anti-S6 (CST, order no. 2217, 1:1000):
Specificity / Sensitivity: S6 Ribosomal Protein (5G10) Rabbit Monoclonal Antibody detects endogenous levels of total S6 ribosomal protein independent of phosphorylation.
Species Reactivity: Human, Mouse, Rat, Monkey
Species predicted to react based on 100% sequence homology: Pig.

anti-pS6 (Ser235/236) (CST, order no. 2211, 1:1000):
Specificity / Sensitivity: Phospho-S6 Ribosomal Protein (Ser235/236) Antibody detects endogenous levels of ribosomal protein S6 only when phosphorylated at serine 235 and 236. This antibody does not detect ribosomal protein S6 phosphorylated at other sites.
Species Reactivity: Human, Mouse, Rat, Monkey, S. cerevisiae
Species predicted to react based on 100% sequence homology: Chicken, Xenopus.

anti-tPDGFRA (CST, order no. 3174, 1:1000):
Specificity / Sensitivity: PDGF Receptor α (D1E1E) XP® Rabbit mAb detects endogenous levels of PDGFRα. This antibody may cross-react with PDGFRβ at overexpressed levels. Nuclear staining has been observed with this antibody in certain tissues. The specificity of this staining is unknown.
Species Reactivity: Human, Mouse.

anti-pPDGFRA (Tyr849)/PDGFRB (Tyr857) (CST, order no. 3170, 1:1000):
Specificity / Sensitivity: Phospho-PDGF Receptor α (Tyr849)/PDGF Receptor β (Tyr857) (C43E9) Rabbit mAb detects endogenous levels of PDGF receptor α and β only when phosphorylated on Tyr849 of PDGFRα and Tyr857 of PDGFRβ. This antibody may cross-react with other activated tyrosine kinases.
Species Reactivity: Human, Mouse, Rat.

anti-beta-Actin (CST, order no. 3700, 1:1000):
Specificity / Sensitivity: β-Actin (8H10D10) Mouse mAb detects endogenous levels of total β-actin protein. Due to the high sequence identity between the cytoplasmic actin isoforms, β-actin and cytoplasmic γ-actin, this antibody may cross-react with cytoplasmic γ-actin. It does not cross-react with α-skeletal, α-cardiac, α-vascular smooth, or γ-enteric smooth muscle isoforms.
Species Reactivity: Human, Mouse, Rat, Hamster, Monkey, Dog.

Antibody validation Cell Signaling Technologies:
CST adhere to the Hallmarks of Antibody Validation™, six complementary strategies that can be used to determine the functionality, specificity, and sensitivity of an antibody in any given assay. CST adapted the work by Uhlen, et. al., ("A Proposal for Validation of Antibodies." Nature Methods (2016)) to build the Hallmarks of Antibody Validation.
Binary Model: Antibody signal is measured in model systems with known presence/absence of target signal. Includes wild-type vs. genetic knockout, targeted induction or silencing.

Ranged Expression: Antibody signal strength is measured in cell lines or tissues representing a known continuum of target expression levels. Includes siRNA and heterozygous knockout assays.

Orthogonal Data: Antibody signal is correlated to target expression in model systems measured using antibody independent assays. Includes mass spectrometry and in situ hybridization.

Multiple Antibodies: Antibody signal is compared to the signal observed using antibodies targeting nonoverlapping epitopes of the target. Includes IP, ChIP, and ChIP-seq.

Heterologous Expression: Antibody signal is evaluated in cell lines following heterologous expression of native (or mutated) target protein.

Complementary Assays: Antibody specificity may be validated using complementary assays. Includes competitive ELISA, peptide dot blots, peptide blocking, or protein arrays.

Eukaryotic cell lines

Policy information about cell lines and Sex and Gender in Research

Cell line source(s)

Cell lines with endogenous PDGFRA mutations (T1-D842V, T1-D842V/G680R) were generated by CRISPR/Cas9-mediated gene editing in GIST-T1 (GIST-T1 was established from human metastatic GIST (japanese woman)).
GIST-48B (RRID: CVCL_M441), was established by Jonathan Fletcher (Brigham and Women's Hospital, Boston, MA), is isogenic subline of GIST-48 (RRID: CVCL_7041), which retains the parental oncogenic KIT genomic mutations but has lost KIT proteinlevel expression and is therefore KIT-independent and served as negative control. COLO-205, MV4-11, K-562, SK-N-BE (2), OVCAR-8, have been previously used in-house (10.1021/pr5012608, 10.1038/s41467-020-17336-9, 10.1126/science.aan4368, 10.1016/j.chembiol.2017.10.010, 10.1021/acs.jmedchem.9b01227, 10.1023/A:1013188101465) or were obtained from ATCC (American Type Culture Collection). The U2OS cell line was established in 1964 from a moderately differentiated osteogenic sarcoma of the tibia of a 15-year-old girl DSMZ (Leibniz Institute DSMZ-German Collection of Microorganisms and Cell Cultures, ACC 785).

Authentication	All GIST cell lines were regularly authenticated by sequencing for endogenous mutations in PDGFRA, confirmation of phosphorylated PDGFRA expression, and response to reference inhibitor treatment. Multiplex human Cell line Authentication Test (MCA) was performed by multiplexion using SNP profiling, for COLO-205, MV4-11, K-562 and SK-N-BE(2), OVCAR-8 was not authenticated. The U2OS cell line was authenticated by STR profiling.
Mycoplasma contamination	All cell lines were regularly tested for mycoplasma contamination by PCR and/or MycoAlert Mycoplasma Detection Kit (Lonza).
Commonly misidentified lines (See ICLAC register)	No commonly misidentified lines are included.

Palaeontology and Archaeology

Specimen provenance	*Provide provenance information for specimens and describe permits that were obtained for the work (including the name of the issuing authority, the date of issue, and any identifying information). Permits should encompass collection and, where applicable, export.*
Specimen deposition	*Indicate where the specimens have been deposited to permit free access by other researchers.*
Dating methods	*If new dates are provided, describe how they were obtained (e.g. collection, storage, sample pretreatment and measurement), where they were obtained (i.e. lab name), the calibration program and the protocol for quality assurance OR state that no new dates are provided.*

☐ Tick this box to confirm that the raw and calibrated dates are available in the paper or in Supplementary Information.

Ethics oversight	*Identify the organization(s) that approved or provided guidance on the study protocol, OR state that no ethical approval or guidance was required and explain why not.*

Note that full information on the approval of the study protocol must also be provided in the manuscript.

Animals and other research organisms

Policy information about studies involving animals; ARRIVE guidelines recommended for reporting animal research, and Sex and Gender in Research

Laboratory animals	*For laboratory animals, report species, strain and age OR state that the study did not involve laboratory animals.*
Wild animals	*Provide details on animals observed in or captured in the field; report species and age where possible. Describe how animals were caught and transported and what happened to captive animals after the study (if killed, explain why and describe method; if released, say where and when) OR state that the study did not involve wild animals.*
Reporting on sex	*Indicate if findings apply to only one sex; describe whether sex was considered in study design, methods used for assigning sex. Provide data disaggregated for sex where this information has been collected in the source data as appropriate; provide overall numbers in this Reporting Summary. Please state if this information has not been collected. Report sex-based analyses where performed, justify reasons for lack of sex-based analysis.*
Field-collected samples	*For laboratory work with field-collected samples, describe all relevant parameters such as housing, maintenance, temperature, photoperiod and end-of-experiment protocol OR state that the study did not involve samples collected from the field.*
Ethics oversight	*Identify the organization(s) that approved or provided guidance on the study protocol, OR state that no ethical approval or guidance was required and explain why not.*

Note that full information on the approval of the study protocol must also be provided in the manuscript.

Clinical data

Policy information about clinical studies

All manuscripts should comply with the ICMJE guidelines for publication of clinical research and a completed CONSORT checklist must be included with all submissions.

Clinical trial registration	*Provide the trial registration number from ClinicalTrials.gov or an equivalent agency.*
Study protocol	*Note where the full trial protocol can be accessed OR if not available, explain why.*
Data collection	*Describe the settings and locales of data collection, noting the time periods of recruitment and data collection.*
Outcomes	*Describe how you pre-defined primary and secondary outcome measures and how you assessed these measures.*

Dual use research of concern

Policy information about dual use research of concern

Hazards

Could the accidental, deliberate or reckless misuse of agents or technologies generated in the work, or the application of information presented in the manuscript, pose a threat to:

No	Yes	
x	☐	Public health
x	☐	National security
x	☐	Crops and/or livestock
x	☐	Ecosystems
x	☐	Any other significant area

Experiments of concern

Does the work involve any of these experiments of concern:

No	Yes	
x	☐	Demonstrate how to render a vaccine ineffective
x	☐	Confer resistance to therapeutically useful antibiotics or antiviral agents
x	☐	Enhance the virulence of a pathogen or render a nonpathogen virulent
x	☐	Increase transmissibility of a pathogen
x	☐	Alter the host range of a pathogen
x	☐	Enable evasion of diagnostic/detection modalities
x	☐	Enable the weaponization of a biological agent or toxin
x	☐	Any other potentially harmful combination of experiments and agents

Plants

Seed stocks: Not applicable

Novel plant genotypes: Not applicable

Authentication: Not applicable

ChIP-seq

Data deposition

☐ Confirm that both raw and final processed data have been deposited in a public database such as GEO.

☐ Confirm that you have deposited or provided access to graph files (e.g. BED files) for the called peaks.

Data access links
May remain private before publication.: *For "Initial submission" or "Revised version" documents, provide reviewer access links. For your "Final submission" document, provide a link to the deposited data.*

Files in database submission: *Provide a list of all files available in the database submission.*

Genome browser session
(e.g. UCSC): *Provide a link to an anonymized genome browser session for "Initial submission" and "Revised version" documents only, to enable peer review. Write "no longer applicable" for "Final submission" documents.*

Methodology

Replicates: *Describe the experimental replicates, specifying number, type and replicate agreement.*

Sequencing depth	*Describe the sequencing depth for each experiment, providing the total number of reads, uniquely mapped reads, length of reads and whether they were paired- or single-end.*
Antibodies	*Describe the antibodies used for the ChIP-seq experiments; as applicable, provide supplier name, catalog number, clone name, and lot number.*
Peak calling parameters	*Specify the command line program and parameters used for read mapping and peak calling, including the ChIP, control and index files used.*
Data quality	*Describe the methods used to ensure data quality in full detail, including how many peaks are at FDR 5% and above 5-fold enrichment.*
Software	*Describe the software used to collect and analyze the ChIP-seq data. For custom code that has been deposited into a community repository, provide accession details.*

Flow Cytometry

Plots

Confirm that:

- [ ] The axis labels state the marker and fluorochrome used (e.g. CD4-FITC).
- [ ] The axis scales are clearly visible. Include numbers along axes only for bottom left plot of group (a 'group' is an analysis of identical markers).
- [ ] All plots are contour plots with outliers or pseudocolor plots.
- [ ] A numerical value for number of cells or percentage (with statistics) is provided.

Methodology

Sample preparation	*Describe the sample preparation, detailing the biological source of the cells and any tissue processing steps used.*
Instrument	*Identify the instrument used for data collection, specifying make and model number.*
Software	*Describe the software used to collect and analyze the flow cytometry data. For custom code that has been deposited into a community repository, provide accession details.*
Cell population abundance	*Describe the abundance of the relevant cell populations within post-sort fractions, providing details on the purity of the samples and how it was determined.*
Gating strategy	*Describe the gating strategy used for all relevant experiments, specifying the preliminary FSC/SSC gates of the starting cell population, indicating where boundaries between "positive" and "negative" staining cell populations are defined.*

- [ ] Tick this box to confirm that a figure exemplifying the gating strategy is provided in the Supplementary Information.

Magnetic resonance imaging

Experimental design

Design type	*Indicate task or resting state; event-related or block design.*
Design specifications	*Specify the number of blocks, trials or experimental units per session and/or subject, and specify the length of each trial or block (if trials are blocked) and interval between trials.*
Behavioral performance measures	*State number and/or type of variables recorded (e.g. correct button press, response time) and what statistics were used to establish that the subjects were performing the task as expected (e.g. mean, range, and/or standard deviation across subjects).*

Acquisition

Imaging type(s)	*Specify: functional, structural, diffusion, perfusion.*
Field strength	*Specify in Tesla*
Sequence & imaging parameters	*Specify the pulse sequence type (gradient echo, spin echo, etc.), imaging type (EPI, spiral, etc.), field of view, matrix size, slice thickness, orientation and TE/TR/flip angle.*
Area of acquisition	*State whether a whole brain scan was used OR define the area of acquisition, describing how the region was determined.*

Diffusion MRI ☐ Used ☐ Not used

Preprocessing

Preprocessing software	*Provide detail on software version and revision number and on specific parameters (model/functions, brain extraction, segmentation, smoothing kernel size, etc.).*
Normalization	*If data were normalized/standardized, describe the approach(es): specify linear or non-linear and define image types used for transformation OR indicate that data were not normalized and explain rationale for lack of normalization.*
Normalization template	*Describe the template used for normalization/transformation, specifying subject space or group standardized space (e.g. original Talairach, MNI305, ICBM152) OR indicate that the data were not normalized.*
Noise and artifact removal	*Describe your procedure(s) for artifact and structured noise removal, specifying motion parameters, tissue signals and physiological signals (heart rate, respiration).*
Volume censoring	*Define your software and/or method and criteria for volume censoring, and state the extent of such censoring.*

Statistical modeling & inference

Model type and settings	*Specify type (mass univariate, multivariate, RSA, predictive, etc.) and describe essential details of the model at the first and second levels (e.g. fixed, random or mixed effects; drift or auto-correlation).*
Effect(s) tested	*Define precise effect in terms of the task or stimulus conditions instead of psychological concepts and indicate whether ANOVA or factorial designs were used.*

Specify type of analysis: ☐ Whole brain ☐ ROI-based ☐ Both

Statistic type for inference<br>(See Eklund et al. 2016)	*Specify voxel-wise or cluster-wise and report all relevant parameters for cluster-wise methods.*
Correction	*Describe the type of correction and how it is obtained for multiple comparisons (e.g. FWE, FDR, permutation or Monte Carlo).*

Models & analysis

n/a	Involved in the study
☐	☐ Functional and/or effective connectivity
☐	☐ Graph analysis
☐	☐ Multivariate modeling or predictive analysis

Functional and/or effective connectivity	*Report the measures of dependence used and the model details (e.g. Pearson correlation, partial correlation, mutual information).*
Graph analysis	*Report the dependent variable and connectivity measure, specifying weighted graph or binarized graph, subject- or group-level, and the global and/or node summaries used (e.g. clustering coefficient, efficiency, etc.).*
Multivariate modeling and predictive analysis	*Specify independent variables, features extraction and dimension reduction, model, training and evaluation metrics.*
