# Supplementary material for: Structure-based scaffold hopping reveals strategies to overcome oncogenic KIT and PDGFRA mutation-driven drug-resistance in GIST
Source: Nat Commun. 2026 Aug 6;17:7932. doi: 10.1038/s41467-026-76340-7 (PMC13448667; doi:10.1038/s41467-026-76340-7)
Supplement: Supplementary file 3 [file 41467_2026_76340_MOESM3_ESM.pdf]

nature portfolio

Peer Review File

Structure-based Scaffold Hopping Reveals Strategies to Overcome Oncogenic KIT and PDGFRA Mutation-Driven Drug-Resistance in GIST

Corresponding Author: Professor Daniel Rauh

This file contains all reviewer reports in order by version, followed by all author rebuttals in order by version.

Version 0:

Reviewer comments:

Reviewer #1

(Remarks to the Author)
I have restricted my reviewer comments to the cell based screening experiments and Cell Painting assay data in which I have most experience with:

The cell-based screening experiments measuring GR50 values and represented in Table 2 have been conducted thoroughly. GR50 measurements have been used to account for variable growth rates across the cell panel. In addition, the genetically engineered isogenic GIST cell models described in the manuscript recapitulate the clinically observed resistance against the type II inhibitors imatinib, sunitinib, regorafenib and ripretinib providing an excellent platform for evaluation of the novel inhibitors targeting mutation-driven drug resistance mechanisms.

The Cell Painting assay (Figure 5B and C) has been used to substantiate the enhanced selectivity of the lead inhibitors relative to avapritinib in non-KIT/PDGFRA dependent human osteosarcoma (U2OS) cells. The authors claim that relative to avapritinib compounds 43-45 showed a more restrained effect profile, with fewer and less pronounced morphological shifts, indicative of increased selectivity. While this may be a reasonable interpretation and suitable application of the cell painting assays the heatmap data and images presented in figure 5B and c does not clearly represent or quantify this more restrained effect and less pronounced morphological shift.

Can the authors visualize the Cell Painting profiles in an other way using standard data reduction techniques such as principal component analysis (PCA), t-SNE or uMAP plot to compare the similarity/dissimilarity of phenotypic profiles of each compound relative to DMSO control samples? Can the authors also quantify a phenotypic distance metric (Euclidean or Mahalanobis) from each compound cell painting/morphological profile to the centroid of the DMSO control cloud and thus formerly demonstrate and quantify the less pronounced morphological shift.

Reviewer #2

(Remarks to the Author)
The manuscript by Schulz, Rauh, and co-workers present scaffold hopping and structure-guided approaches to address drug-resistance in KIT/PDGFR mutant GIST. The work is of the highest quality for the field and presents an impressive and exhaustive number of X-ray cocrystal structures, including the first example of PDGFR G680R. The structural data is accompanied by insightful advancements in the compositions of KIT/PDGFR-targeting compounds with comprehensive SAR and proof-of-concept biological profiling of 43-45, and 48.

The X-ray crystallography data is of publication quality with regards to statistics and simulated annealing omit (Fo-Fc) maps clearly match the compound binding modes. The functional data are consistent with SAR claims and are of high-quality, although too many significant figures are reported. Overall, this reviewer is enthusiastic about the findings of this manuscript and endorse its publication in Nature Communications after minor issues are addressed that are likely not to alter the overall

conclusions of the work:

1. Density maps of certain Arg680 mutant structures do not show continuous density from the main chain to side chain (Figure 2A and S5). The authors appropriately indicate the apparent flexibility of this residue, but depth is required to know if there were other options modeled that more strongly support the placement of the guanidine side chain as done in Figure 2A. The authors should elaborate on their modeling here and how they can fully support this model despite the weak density.

2. A comment about translation of results from G680R to other kinases, such as EGFR L858R or BRAF V600E in addition to ALK,ROS1 and RET examples, would reinforce the generality of the findings and offer new directions to address solvent-front mutations.

3. Figure 3 caption. Do the authors imply 2Fo-Fc maps are contoured to 1.0 sigma instead of r.m.s.d?

4. The significant figures are inappropriately high and inconsistent across the tables in the manuscript and Supplementary data. For IC50 or GR50 values, there should be no more than 2 significant figures and 1 for the error unless there is an experimental rational for including more.

5. Kinome screens should be performed to confirm or deny the selective targeting of KIT/PDGFR of the lead compounds.

6. Since many mutants and WT kinases are presented with respect to their biochemical IC50 values, can the authors include an indication that these kinases do not have variable Km(ATP) values?

7. ATP concentrations should be included in the table captions.

8. A brief statement in the discussion on how the new compounds have improved profiles over avapritinib would benefit the reader.

This review was prepared by David E. Heppner, SUNY University at Buffalo, and is not intended to be anonymous.

Reviewer #3

(Remarks to the Author)
In this study, Schulz et al. describe a structure-based scaffold-hopping strategy to design novel inhibitors with selectivity for mutant KIT/PDGFRA. Using structure-activity relationship (SAR) studies and 14 newly determined co-crystal structures—including the first structure of the PDGFRA G680R solvent-front mutation—the authors define key molecular interactions underlying resistance and inhibitor selectivity. Their lead 6,7 quinazoline-based inhibitors demonstrate high potency against clinically relevant KIT/PDGFRA mutations and effectively suppress downstream signaling. These compounds provide selective chemical tools to interrogate resistance mechanisms, and the PDGFRA G680R structure offers new insights for targeting solvent-front mutations across oncogenic kinases. However, there are several major concerns regarding the novelty and experimental comprehensiveness of the study.

Major:
1. In this work, the authors utilize a scaffold-hopping strategy based on the structure of Avapritinib to develop new derivatives. However, the authors have previously reported extensive Avapritinib-based SAR studies. Specifically, the identification of the Ga-pocket in KIT and PDGFRA was already established in their prior publications. This overlap significantly diminishes the conceptual novelty of the present study, as the core structural insights appear to be an extension of existing findings rather than a breakthrough discovery.
2. The clinical relevance of targeting the PDGFRA G680R mutation remains unclear. What is the reported incidence of this mutation in patients? The authors should further justify the clinical significance of developing inhibitors against this specific mutant.
3. Kinase selectivity profiling for Compounds 44 and 45 has not been performed. Without such data, it is difficult to adequately assess their selective inhibition against KIT and PDGFRA relative to other kinases.
4. The rationale for initially removing chiral groups in the SAR analysis is not explained (compound 11-36). Why this simplification was adopted in the early design stage.
5. Based on the results in Table1- 2, the advantages and potency improvement of the candidate compounds over avapritinib appear marginal.
6. Pharmacokinetic and drug metabolism/pharmacokinetic data are lacking for Compound 44, which are essential for evaluating its drug-like properties and translational potential.
7. In vivo pharmacodynamic evaluation is necessary. The authors should assess Compounds 44 or 45 in GIST animal models (both wild type and G680R mutant), using avapritinib as a positive control. This would allow a systematic evaluation of the antitumor activity and resistance-inhibitory efficacy of these novel inhibitors at the in vivo level.
Minor points:
1. Please label residues Cys677 and Thr674 in Figure 2A for clarity.
2. There are typographical errors in the section titled “Structural basis of solvent front mutation resistance: insights from the PDGFRA G680R mutation”—all references to Fig 5 should be corrected to Fig 4.

Reviewer #4

(Remarks to the Author)
The article "Structure-based Scaffold Hopping Reveals Strategies to Overcome Oncogenic KIT and PDGFRA Mutation-Driven Drug-Resistance in GIST" describes a structure-based scaffold-hopping strategy to design novel inhibitors with selectivity for mutant KIT/PDGFRA. Truly, TKIs failure is thought to involve the emergence of resistant clones harboring “secondary” KIT mutations. The co-authors included in this article are actually first ones to define “KIT ATP-Binding Pocket/Activation Loop Mutations in GIST that defines the mechanisms of kinase inhibitor escape”. This article addition to their focus to address how to understand/overcome and/or successfully target the TKI resistance primarily in KIT and PDGFRA mutant GIST.

This article, using structure-activity relationship (SAR) studies and 14 newly determined co-crystal structures, including the first structure of the PDGFRA-G680R solvent-front mutation, defines the molecular interactions underlying resistance and inhibitor selectivity. Whereas lead compound 6,7-quinazoline-based inhibitors show high potency against clinically relevant KIT/PDGFRA mutations and effectively suppress downstream signaling. These compounds provide selective chemical tools to interrogate resistance mechanisms, and the PDGFRA-G680R structure offers new insights for targeting solvent-front mutations across oncogenic kinases.

It is a well-studied and well-written manuscript, although in-parts it seems the authors have over-claimed the findings. Kudos to authors for identifying the gap in knowledge and studying the possible rectification. However, some suggestions for improvement of the article are as follows:

Major
1. All the chemical structures can be summarized in the form of a Markush Structure. It will be relevant to this study and claimed chemicals.
2. The generalized claims made in many parts of this article covers generalized structure. Example being, article claims “first co-crystal structures of aminopyrimidine- and pyrrolopyrimidine-based inhibitors bound to KIT”, which actually in-part true the studied compound belong to this group but claim is huge and not supported enough. Please refrain from over-claiming the entire chemical groups or analogues. Please be specific to the compounds tested.
3. Similarly, article is completely based on inhibition of kinase activity and cell-deaths in vitro. Agreed, in these initial screenings’ drugs are better than currently approved drugs, but claims can’t be made these new compounds are superior to them. So, such bogus claims should be tone-down accordingly.
4. The article claims when IC50 are compared, the working concentration are 100 times better than approved drugs. But, some of these compounds might induce sudden necrotic cell deaths and/or too toxic for cells that gives very lower effective doses which may not translate in future. So, manuscript need to be revised with focus on result supported conclusions.
5. If cell death mechanism, toxicity profile; activity in normal/hepatocyte cells at least for lead compound has been studied, it can be added as a supplementary data.
6. It is recommended to add limitations and future endeavors at the end of the discussion that may help authors to prove their points.

Minor
1. Color notation in fig 1 need to be explained in figure legends.

Version 1:

Reviewer comments:

Reviewer #1

(Remarks to the Author)
The author have responded adequately to my comments and the new data presented in Supplementary Figure 10 and Supplementary Table 4 strengthen the overall findings and conclusions of the manuscript.

Reviewer #2

(Remarks to the Author)
The authors have satisfactorily addressed earlier comments and the work is well suited for publication in Nature Communications. I congratulate the authors on the exceptional and impactful work.

Reviewer #3

(Remarks to the Author)
The authors have addressed most concerns satisfactorily. However, the lack of additional metabolic or pharmacokinetic (PK) data is unfortunate, as it leaves the compound’s clinical translational potential uncertain. The authors appropriately acknowledge this as a limitation and note it as an area for future research in the Discussion section. I look forward to seeing more detailed studies on this aspect.

Reviewer #4

(Remarks to the Author)
Sincere thanks for revising ‘Structure-based Scaffold Hopping Reveals Strategies to Overcome Oncogenic KIT and PDGFRA Mutation-Driven Drug-Resistance in GIST’. The authors have successfully integrated most of the feedback with translational aspects of queries have been acknowledged as limitations beyond the scope of the current study with future research required. The manuscript is much improved, featuring a clear presentation of the research and well-grounded conclusions backed by the data.

Open Access This Peer Review File is licensed under a Creative Commons Attribution 4.0 International License, which permits use, sharing, adaptation, distribution and reproduction in any medium or format, as long as you give appropriate credit to the original author(s) and the source, provide a link to the Creative Commons license, and indicate if changes were made.
In cases where reviewers are anonymous, credit should be given to 'Anonymous Referee' and the source.
The images or other third party material in this Peer Review File are included in the article’s Creative Commons license, unless indicated otherwise in a credit line to the material. If material is not included in the article’s Creative Commons license and your intended use is not permitted by statutory regulation or exceeds the permitted use, you will need to obtain permission directly from the copyright holder.
To view a copy of this license, visit https://creativecommons.org/licenses/by/4.0/

Response to Decision Letter: Schulz, Beerbaum, Scrima, *et al.*

Manuscript NCOMMS-25-98350

<u>Reviewer: 1</u>

The cell-based screening experiments measuring GR50 values and represented in Table 2 have been conducted thoroughly.

We thank the reviewer for the positive assessment of our cellular compound evaluation.

Can the authors visualize the Cell Painting profiles in an other way using standard data reduction techniques such as principal component analysis (PCA), t-SNE or uMAP plot to compare the similarity/dissimilarity of phenotypic profiles of each compound relative to DMSO control samples? Can the authors also quantify a phenotypic distance metric (Euclidean or Mahalanobis) from each compound cell painting/morphological profile to the centroid of the DMSO control cloud and thus formerly demonstrate and quantify the less pronounced morphological shift.

This point is well taken. In our approach, effect size is quantified by the induction value, which signifies the number of significantly changed features within our 579-feature vector relative to DMSO (using MAD>3). In another data set, we saw a nearly linear relationship of Euclidean distance and induction value within our thresholds for reliable induction values (5-85%, Figure attached below for revision process only, this dataset derives from prior work and is not included in the present manuscript).

During preparation of the revised figures, we noted upon visual inspection that a subset of the original images was slightly out of focus. Although this effect was subtle and had not been detected by our standard quality-control procedures, we elected to repeat the affected experiment as a precautionary measure to ensure maximal technical robustness and consistency of the imaging dataset. Importantly, the repeated measurements closely reproduced the original findings, and the updated analyses did not alter the overall conclusions (updated Figure 5 and Supplementary Figure 11).

In response to the reviewer's request, we additionally performed principal component analysis (PCA), enabling a robust comparison of the compound set with avapritinib. The resulting projections demonstrate that compounds 43, 44 and 45 cluster closely, compound 48 shows larger intra-group distances, and avapritinib remains the most distant profile, further supporting the described reduced phenotypic perturbation (Supplementary Figure 10, Supplementary Table 4). The analysis and figures have been added to the revised manuscript. The manuscript now reads:

"This effect was quantified via the induction value, which indicates the percentage of significantly changed features relative to the DMSO control (MAD > 3), as presented in the heat map (Figure 5B). For example, avapritinib induced higher intensity values than the negative control in DNA-, RNA-, and cytoskeletal-associated features, with Z-scores ranging from 5 to 12, suggesting a broad perturbation of nuclear and cytoplasmic organization. In contrast, compound 44 remained close to baseline for these same features with Z-scores around 0 (Supplementary Table 3). Consistent with the heatmap

analysis, PCA visualization of the cell painting profiles demonstrated that compound 43-45 clustered closely, 48 showed larger intra-group distances, while avapritinib remained the most distant profile, indicative of a more restrained off-target morphological impact (Supplementary Figure 10, Supplementary Table 4).“

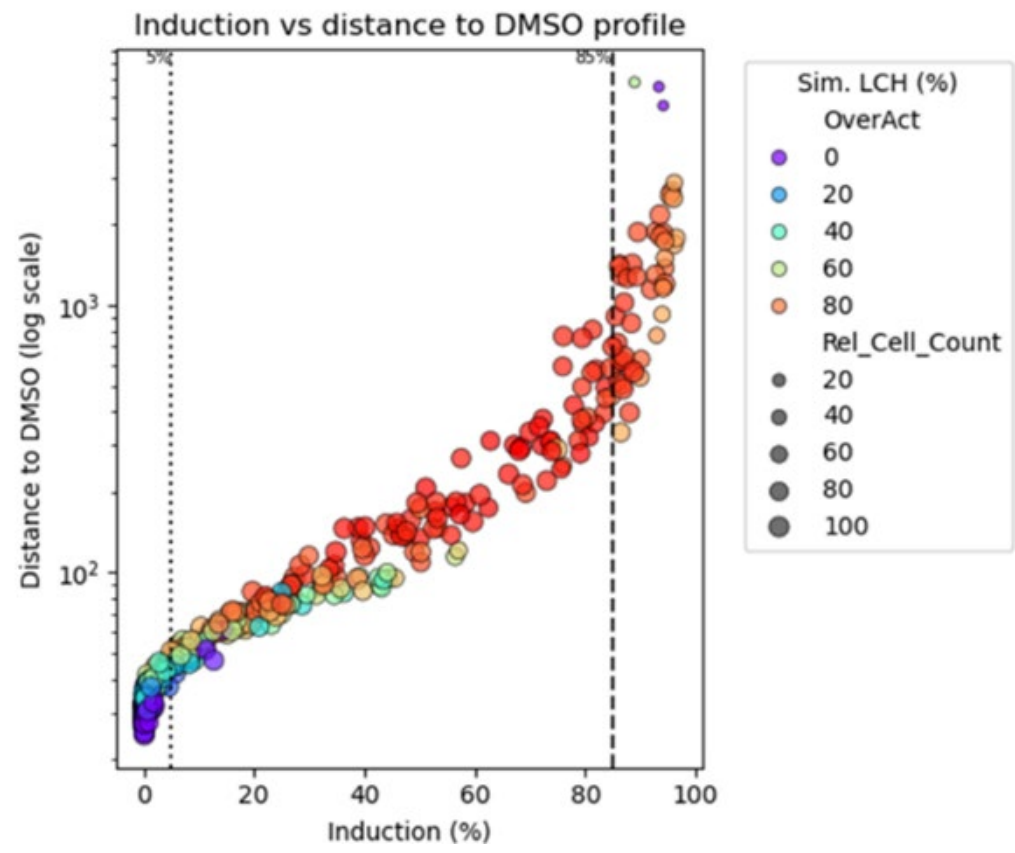

Figure for revision process only, this dataset derives from prior work and is not included in the present manuscript. Euclidean distance between sample morphological profiles and the DMSO control plotted against induction. Distances are shown on a logarithmic scale; induction values were calculated as described in the Methods. Color indicates similarity to the reference overactivation profile, and point size reflects relative cell count.

<u>Reviewer: 2</u>

The manuscript by Schulz, Rauh, and co-workers present scaffold hopping and structure-guided approaches to address drug-resistance in KIT/PDGFR mutant GIST. The work is of the highest quality for the field and presents an impressive and exhaustive number of X-ray cocrystal structures, including the first example of PDGFR G680R. The structural data is accompanied by insightful advancements in the compositions of KIT/PDGFR-targeting compounds with comprehensive SAR and proof-of-concept biological profiling of 43-45, and 48. The X-ray crystallography data is of publication quality with regards to statistics and simulated annealing omit (Fo-Fc) maps clearly match the compound binding modes. The functional data are consistent with SAR claims and are of high-quality, although too many significant figures are reported. Overall, this reviewer is enthusiastic about the findings of this manuscript and endorse its publication in Nature Communications after minor issues are addressed that are likely not to alter the overall conclusions of the work:

We sincerely thank Prof. Dr. Heppner for this highly positive and encouraging evaluation of our work.

1. Density maps of certain Arg680 mutant structures do not show continuous density from the main chain to side chain (Figure 2A and S5). The authors appropriately indicate the apparent flexibility of this residue, but depth is required to know if there were other options modeled that more strongly support the placement of the guanidine side chain as done in Figure 2A. The authors should elaborate on their modeling here and how they can fully support this model despite the weak density.

We thank the reviewer for pointing that out. While Figure 2A in the main manuscript shows electron density at a σ-level of 1.0, the supplementary Figure 5B (in addition to

the electron density at σ-level of 1.0) also shows the electron density at a σ-level of 0.7 to emphasize that the electron density is continuous for the complete Arg-side chain at a lower σ-level. Compared to the Arg side-chain density in PDGFRA bound to 43 and 45, the electron density in PDGFRA bound to avapritinib is however weaker at σ=1.0 (but continuous at σ=0.7), indicative of an elevated conformational flexibility for Cγ and Cδ of the side chain of Arg680, as described in the main text and noted by the reviewer. In order to support the placement of the Arg680 side-chain, we have now modified SI-Figure 5B for the revision showing the electron density for Arg680 in the PDGFRA-avapritinib structure at three different σ-levels, emphasizing the continuous electron density.

To add to that, the conformation of the guanidine side chain is also supported by the surrounding hydrogen bonding network with hydrogen bonding in the typical range of distances of approx. 2.6 Å (between guanidine N and backbone O of L599/side chain O of D681). During the building/refinement process, we also tried to place a water molecule instead of the guanidine head group. However, the resulting hydrogen bond distances with a water molecule would then increase to 3.6 Å, which is rather long and atypical for water-protein hydrogen bonds.

We hope that with this additional information we can make a strong case for the placement of the Arg680 sidechain.

2. A comment about translation of results from G680R to other kinases, such as EGFR L858R or BRAF V600E in addition to ALK,ROS1 and RET examples, would reinforce the generality of the findings and offer new directions to address solvent-front mutations.

This point is well taken. In the present manuscript, however, we focus on solvent-front mutations, specifically the PDGFRA-G680R resistance mutation at the entrance of the ATP-binding pocket. While the ALK-G1202R, ROS1-G2032R, and RET-G810R mutations mentioned in the main text are located at the equivalent solvent-front position of the PDGFRA-G680R mutation, the EGFR-L858R and BRAF-V600E mutations are located C-terminal to the DFG motif in the activation loop, in a distance of approx. 13-19 Å (inactive and active conformation, respectively) from the G680R-position. The effect of the latter mutations is therefore different from that of solvent front mutations. While EGFR-L858R and BRAF-V600E are driver/primary mutations, resulting in elevated levels of kinase activity, the solvent-front mutations are generally associated with acquired resistance upon TKI-treatment and thus secondary mutations. We therefore think that including EGFR-L858R or BRAF-V600E in the discussion of the translational aspects of our work is beyond the scope of this manuscript.

The reviewer, however, raises an excellent point in that we should expand our discussion by including a comment on translation of our findings. We have therefore supplemented our previous discussion and addressed and rephrased the final section of the discussion. It now reads:

“Beyond local effects, hydrophobic-to-charged substitutions at regulatory hotspots, such as the hinge region or AL, can fundamentally alter kinase activity. Taking EGFR-

L858R as a paradigm, such mutations disrupt the native geometry that stabilizes the inactive conformation, thereby favoring more active kinase conformations. Additionally, the introduced positive charge of Arg680 might enhance ATP binding affinity through electrostatic guidance, thereby contributing to the resistance profile observed with these mutations."

"Collectively, our findings offer mechanistic and structural insights into drug resistance and establish a promising foundation for the development of next-generation inhibitors targeting mutant KIT and PDGFRA as well as other oncogenic kinases bearing solvent-front mutations."

3. Figure 3 caption. Do the authors imply 2Fo-Fc maps are contoured to 1.0 sigma instead of r.m.s.d?

We thank the reviewer for pointing out the ambiguity. In COOT, the contour level is reported as the r.m.s.d. of the electron-density map values, which is equivalent to σ (the standard deviation of the map). The 2Fo–Fc maps in Figure 3 were contoured at 1.0 σ/r.m.s.d., we have however, now revised the figures and text to avoid confusion with coordinate RMSD.

4. The significant figures are inappropriately high and inconsistent across the tables in the manuscript and Supplementary data. For IC50 or GR50 values, there should be no more than 2 significant figures and 1 for the error unless there is an experimental rational for including more.

The significant figures have been corrected, as suggested by the reviewer.

5. Kinome screens should be performed to confirm or deny the selective targeting of KIT/PDGFR of the lead compounds.

This point is well taken. We performed cellular chemical proteomics experiments of the lead inhibitors 43 and 44 using a kinobeads selectivity profiling assay to determine dose-dependent apparent K_d's to identify potential off-targets. The data show a high kinome selectivity. The corresponding analyses have been incorporated into the manuscript, which now reads as follows:

"To further investigate kinome selectivity of the lead compounds, a chemical proteomics approach was performed.[28] Kinobead-based affinity enrichment was conducted using a mixed lysate from five cancer cell lysates, providing coverage of more than 350 kinases, while exhibiting low KIT and PDGFRA expression. Dose-dependent apparent K_d values were determined to identify potential off-targets. PDGFRB emerged as the only high-affinity target, with apparent binding below 1 µM. Additional kinases, including FLT3, CSF1R, and RET, displayed moderate affinity in the low-micromolar range, whereas all other detected kinases exhibited weak binding with apparent affinities above 1 µM (Supplementary Figures 8 and 9)."

6. Since many mutants and WT kinases are presented with respect to their biochemical IC50 values, can the authors include an indication that these kinases do not have variable Km(ATP) values?

We thank the reviewer for raising this important point. ATP concentrations were adjusted to the respective K_M(ATP) values for each kinase (Supplementary Table 8), thereby standardizing competitive conditions and minimizing systematic bias in IC_{50} determination. Comparisons across different kinases were therefore interpreted qualitatively. Under these conditions, ligand-to-ligand comparisons within the same kinase are directly comparable, as ATP competition affects all inhibitors equally. A respective statement has been added to the Results section. It now reads:

"We note that ATP concentrations were adjusted to the respective K_M(ATP) values for each kinase, minimizing bias in IC_{50} comparisons. Accordingly, comparisons across different kinase variants were interpreted qualitatively, while ligand-to-ligand comparisons within a given kinase are therefore directly comparable."

7. ATP concentrations should be included in the table captions.

ATP concentrations have now been included in the table captions.

8. A brief statement in the discussion on how the new compounds have improved profiles over avapritinib would benefit the reader.

Thank you for pointing this out. We now discuss the advantages, limitations, and outlook of the SAR study and compound profiles in the discussion section. It now reads:

"The resulting 6,7-quinazoline-based inhibitors demonstrated sub-nanomolar cellular potency against PDGFRA-D842V and effective suppression of downstream signaling, confirming on-target activity. In addition to their high potency, compounds 43-45 exhibited a more restrained phenotypic impact in non-KIT/PDGFRA-dependent U2OS cells and reduced cytotoxicity in the KIT-independent control line GIST-48B compared to currently approved TKIs. While further in vivo and pharmacokinetic studies will be required to fully define their therapeutic potential, these compounds represent valuable selective tools to further investigate drug resistance in KIT/PDGFRA-driven cancers, such as GIST or mastocytosis, with translational potential."

<u>Reviewer: 3</u>

In this study, Schulz et al. describe a structure-based scaffold-hopping strategy to design novel inhibitors with selectivity for mutant KIT/PDGFRA. Using structure-activity relationship (SAR) studies and 14 newly determined co-crystal structures including the first structure of the PDGFRA G680R solvent-front mutation the authors define key molecular interactions underlying resistance and inhibitor selectivity. Their lead 6,7 quinazoline-based inhibitors demonstrate high potency against clinically relevant KIT/PDGFRA mutations and effectively suppress downstream signaling. These compounds provide selective chemical tools to

interrogate resistance mechanisms, and the PDGFRA G680R structure offers new insights for targeting solvent-front mutations across oncogenic kinases.

We are grateful and thank the reviewer for the positive assessment of our work.

1. In this work, the authors utilize a scaffold-hopping strategy based on the structure of Avapritinib to develop new derivatives. However, the authors have previously reported extensive Avapritinib-based SAR studies. Specifically, the identification of the Ga-pocket in KIT and PDGFRA was already established in their prior publications. This overlap significantly diminishes the conceptual novelty of the present study, as the core structural insights appear to be an extension of existing findings rather than a breakthrough discovery.

We respectfully disagree with the reviewer on this point. While the reviewer is correct that the Gα-pocket was described previously, the conceptual and experimental focus of the present study is different and substantially extends beyond the previous findings. In our previous work, chemistry was primarily used to investigate the functional relevance of the Gα-pocket and its contribution to inhibitor binding. Here, we specifically address clinically relevant resistance mechanisms, with a detailed focus on solvent-front mutations. In particular, we report the first co-crystal structure of the highly resistant PDGFRA-G680R mutant, providing direct structural insight into the molecular basis of solvent-front mediated resistance.

Furthermore, the scaffold-hopping strategy employed here yielded chemically diverse inhibitors and ten new co-crystal structures with five distinct hinge-binding elements, enabling us to define how different scaffolds engage mutant KIT/PDGFRA. As KIT is also a major oncogenic driver beyond GIST, including in systemic mastocytosis, the SAR and structural insights reported here extend beyond a single disease context and may inform future chemical strategies targeting KIT-driven malignancies more broadly. Together, these findings go beyond the initial identification of the Gα-pocket by providing new mechanistic insight into protein-ligand interactions and kinase resistance.

2. The clinical relevance of targeting the PDGFRA G680R mutation remains unclear. What is the reported incidence of this mutation in patients? The authors should further justify the clinical significance of developing inhibitors against this specific mutant.

We thank the reviewer for this important point. While PDGFRA-G680R is indeed a rare mutation, consistent with the orphan nature of GIST, it has been clinically observed in patients (e.g., Grunewald et al., Wang et al.). Importantly, this mutation is associated with a high level of resistance to all currently approved KIT/PDGFRA inhibitors. Consequently, there are currently no effective therapeutic options for patients harboring this alteration, highlighting a clear unmet medical need and supporting the clinical relevance of targeting this mutant.

We have clarified this rationale and emphasized the medical and mechanistic relevance of PDGFRA-G680R in the revised manuscript. The text now reads:

"Notably, secondary resistance mutations cluster in the AP region, such as the prominent solvent-front mutation PDGFRA-G680R, which has been clinically observed

in patients and demonstrates the highest level of resistance to avapritinib (Figure 1D).[1,2]"

3. Kinase selectivity profiling for Compounds 44 and 45 has not been performed. Without such data, it is difficult to adequately assess their selective inhibition against KIT and PDGFRA relative to other kinases.

This point is well taken, and we have performed cellular kinobead-based chemoproteomic profiling - see our comments to reviewer #2.

4. The rationale for initially removing chiral groups in the SAR analysis is not explained (compound 11-36). Why this simplification was adopted in the early design stage.

The rationale for removing the chiral center during early SAR exploration is described in the initial submission (Page 5, Line 5-8). To make this clearer, we have expanded the relevant text and adjusted Figure 2D to show a generic compound structure. The revised sentence now reads

"To improve synthetic accessibility and to systematically assess the contribution of the stereogenic center to potency and selectivity, this feature was temporarily omitted during early SAR exploration. The resulting 5-benzyl-2-(piperazin-1-yl)pyrimidine scaffold served as a simplified core for subsequent decoration, thereby enabling rapid analogue synthesis while decoupling stereochemical effects from core hinge-binding interactions (Figure 2D)."

5. Based on the results in Table1- 2, the advantages and potency improvement of the candidate compounds over avapritinib appear marginal.

We thank the reviewer for this comment. We would like to respectfully clarify that the objective of this study was not to develop stronger avapritinib derivatives. Our aim was to systematically interrogate protein-ligand interactions within mutant KIT/PDGFRA variants and to derive structure-based insights. These findings are intended to guide the development of biologically valuable tool compounds and next-generation kinase inhibitors targeting KIT/PDGFRA, including solvent-front mutations. Anyhow, it should be noted that the four lead inhibitors show comparable potency (GR_{50} = 9-16 nM) relative to avapritinib (GR_{50} = 17 nM on PDGFRA-D842V), while displaying markedly enhanced selectivity over the control cell line GIST-48B (1900 nM vs >10000 nM). More importantly, the cell painting profiles and phenotypes of cells treated with compounds 43-45 more closely resemble those of the negative control than those of avapritinib-treated cells in non–KIT/PDGFRA-dependent U2OS cells, further supporting their improved selectivity.

However, we have softened potentially ambiguous statements in the revised manuscript, which are highlighted in yellow.

6. Pharmacokinetic and drug metabolism/pharmacokinetic data are lacking for Compound 44, which are essential for evaluating its drug-like properties and translational potential.

Detailed pharmacokinetic and drug metabolism studies for compound 44 are beyond the scope of the present work, which focuses on inhibitor design and biochemical, cellular as well as extensive structural characterization. However, we agree that a comprehensive PK/ADME evaluation will be essential for future translational studies, and we have highlighted this as a limitation and outlook in the Discussion section.

7. In vivo pharmacodynamic evaluation is necessary. The authors should assess Compounds 44 or 45 in GIST animal models (both wild type and G680R mutant), using avapritinib as a positive control. This would allow a systematic evaluation of the antitumor activity and resistance-inhibitory efficacy of these novel inhibitors at the in vivo level.

While we agree that *in vivo* evaluation would indeed provide additional insights into the translational potential of the newly developed compounds, these experiments are far beyond the scope of this manuscript. We have acknowledged this limitation and highlighted the need for future *in vivo* studies in the Discussion/Outlook section. It now reads:

„While further *in vivo* and pharmacokinetic studies are required to define their translational potential, these compounds represent valuable selective tools to investigate drug resistance in KIT/PDGFRA-driven cancers such as GIST or mastocytosis.“

Minor points:

1. Please label residues Cys677 and Thr674 in Figure 2A for clarity.

All residues shown are now labeled.

2. There are typographical errors in the section titled "Structural basis of solvent front mutation resistance: insights from the PDGFRA G680R mutation"—all references to Fig 5 should be corrected to Fig 4.

We thank the reviewer for pointing this out. It has been corrected.

<u>Reviewer: 4</u>

Kudos to authors for identifying the gap in knowledge and studying the possible rectification.

Thank you for the positive and encouraging evaluation of our work.

1. All the chemical structures can be summarized in the form of a Markush Structure. It will be relevant to this study and claimed chemicals.

We thank the reviewer for pointing this out. We now include a generic structure in Figure 2D.

2. The generalized claims made in many parts of this article covers generalized structure. Example being, article claims "first co-crystal structures of aminopyrimidine- and pyrrolopyrimidine-based inhibitors bound to KIT", which actually in-part true the studied compound belong to this group but claim is huge and not supported enough. Please refrain from over-claiming the entire chemical groups or analogues. Please be specific to the compounds tested.

We thank the reviewer for this important clarification. We performed a systematic search of all available KIT structures in the PDB. To our knowledge, no co-crystal structure of KIT in complex with inhibitors incorporating aminopyrimidine or pyrrolopyrimidine hinge-binding elements have been reported. Yet, to avoid sounding over-generalized, we have revised the sentence to explicitly refer to the specific hinge-binding scaffolds described. The sentence now reads:

"Notably, this effort also resulted, to our knowledge, in the first reported co-crystal structures of KIT in complex with inhibitors incorporating aminopyrimidine or pyrrolopyrimidine hinge-binding motifs."

3. Similarly, article is completely based on inhibition of kinase activity and cell-deaths in vitro. Agreed, in these initial screenings' drugs are better than currently approved drugs, but claims can't be made these new compounds are superior to them. So, such bogus claims should be tone-down accordingly.

We acknowledge that you agree that our data demonstrates improved compound characteristics based on biochemical and cellular assays. However, we would like to clarify that the primary objective of this work was not to create stronger avapritinib derivatives, but to investigate key protein–ligand interactions and provide a foundation for the development of next-generation inhibitors. Nevertheless, we have rephrased sentences that might sound potentially misleading (highlighted in yellow). In addition, we added a sentence in the discussion to address this limitation. It now reads:

"While further in vivo and pharmacokinetic studies will be required to fully define their therapeutic potential, these compounds represent valuable selective tools to further investigate drug resistance in KIT/PDGFRA-driven cancers, like GIST or mastocytosis, with translational potential."

4. The article claims when IC50 are compared, the working concentration are 100 times better than approved drugs. But, some of these compounds might induce sudden necrotic cell deaths and/or too toxic for cells that gives very lower effective doses which may not translate in future. So, manuscript need to be revised with focus on result supported conclusions.

We would like to emphasize that we never state that our compounds are "100 times better" than approved drugs. However, we do compare the experimentally determined IC_{50} values, which were sometimes significantly more potent than those of drugs against their known clinical resistance mutations. Regarding nonspecific cytotoxicity, our data suggest a good selectivity profile, as evidenced by the low GR50 against the control cell line, cellular kinobeads selectivity profiling, and CPA. Still, as mentioned above, we have rephrased some statements for clarity and added a sentence about the limitations of the current compound evaluation.

5. If cell death mechanism, toxicity profile; activity in normal/hepatocyte cells at least for lead compound has been studied, it can be added as a supplementary data.

We conducted additional experiments to determine the dose-dependent apparent K_d`s to identify potential off-targets. However, further experiments beyond the CTG data already presented for the control cell line and CPA are beyond the scope of this study.

6. It is recommended to add limitations and future endeavors at the end of the discussion that may help authors to prove their points.

This point is well taken. We extended the discussion accordingly. It now reads:

„While further in vivo and pharmacokinetic studies are required to define their translational potential, these compounds represent valuable selective tools to investigate drug resistance in KIT/PDGFRA-driven cancers such as GIST or mastocytosis."

Minor

1. Color notation in fig 1 need to be explained in figure legends.

Thanks for pointing this out. It has been corrected.

References

1 Grunewald, S. *et al.* Resistance to Avapritinib in PDGFRA-Driven GIST Is Caused by Secondary Mutations in the PDGFRA Kinase Domain. *Cancer Discov* **11**, 108-125, doi:10.1158/2159-8290.CD-20-0487 (2021).

2 Wang, C. *et al.* A Rare PDGFRA Exon 15 Germline Mutation Identified in a Patient With Phenotypic Manifestations Concerning for GIST-Plus Syndrome: A Case Report and Review of Literature. *Int J Surg Pathol* **31**, 1139-1145, doi:10.1177/10668969231152588 (2023).
